# Scaling up prevention and treatment towards the elimination of hepatitis C: a global mathematical model

**DOI:** 10.1016/S0140-6736(18)32277-3

**Published:** 2019-03-30

**Authors:** Alastair Heffernan, Graham S Cooke, Shevanthi Nayagam, Mark Thursz, Timothy B Hallett

**Affiliations:** a MRC Centre for Global Infectious Disease Analysis, Department of Infectious Disease Epidemiology, Faculty of Medicine, Imperial College London, London, UK; b Division of Infectious Diseases, St Mary's Hospital, Imperial College London, London, UK; c Division of Digestive Diseases, St Mary's Hospital, Imperial College London, London, UK

## Abstract

**Background:**

The revolution in hepatitis C virus (HCV) treatment through the development of direct-acting antivirals (DAAs) has generated international interest in the global elimination of the disease as a public health threat. In 2017, this led WHO to establish elimination targets for 2030. We evaluated the impact of public health interventions on the global HCV epidemic and investigated whether WHO's elimination targets could be met.

**Methods:**

We developed a dynamic transmission model of the global HCV epidemic, calibrated to 190 countries, which incorporates data on demography, people who inject drugs (PWID), current coverage of treatment and prevention programmes, natural history of the disease, HCV prevalence, and HCV-attributable mortality. We estimated the worldwide impact of scaling up interventions that reduce risk of transmission, improve access to treatment, and increase screening for HCV infection by considering six scenarios: no change made to existing levels of diagnosis or treatment; sequentially adding the following interventions: blood safety and infection control, PWID harm reduction, offering of DAAs at diagnosis, and outreach screening to increase the number diagnosed; and a scenario in which DAAs are not introduced (ie, treatment is only with pegylated interferon and oral ribavirin) to investigate the effect of DAA use. We explored the effect of varying the coverage or impact of these interventions in sensitivity analyses and also assessed the impact on the global epidemic of removing certain key countries from the package of interventions.

**Findings:**

By 2030, interventions that reduce risk of transmission in the non-PWID population by 80% and increase coverage of harm reduction services to 40% of PWID could avert 14·1 million (95% credible interval 13·0–15·2) new infections. Offering DAAs at time of diagnosis in all countries could prevent 640 000 deaths (620 000–670 000) from cirrhosis and liver cancer. A comprehensive package of prevention, screening, and treatment interventions could avert 15·1 million (13·8–16·1) new infections and 1·5 million (1·4–1·6) cirrhosis and liver cancer deaths, corresponding to an 81% (78–82) reduction in incidence and a 61% (60–62) reduction in mortality compared with 2015 baseline. This reaches the WHO HCV incidence reduction target of 80% but is just short of the mortality reduction target of 65%, which could be reached by 2032. Reducing global burden depends upon success of prevention interventions, implemention of outreach screening, and progress made in key high-burden countries including China, India, and Pakistan.

**Interpretation:**

Further improvements in blood safety and infection control, expansion or creation of PWID harm reduction services, and extensive screening for HCV with concomitant treatment for all are necessary to reduce the burden of HCV. These findings should inform the ongoing global action to eliminate the HCV epidemic.

**Funding:**

Wellcome Trust.

## Introduction

Globally, it is estimated that 71·1 million (95% uncertainty interval 62·5–79·4) individuals are chronically infected with hepatitis C virus (HCV),^1^ of whom 10–20% will develop liver complications including decompensated cirrhosis and hepatocellular carcinoma.^2^, ^3^ These complications were responsible for more than 475 000 deaths in 2015 and, in contrast with the malaria, tuberculosis, and HIV epidemics, the number of deaths from viral hepatitis infection has risen in recent years.^1^, ^4^ HCV transmission is most commonly associated with blood transfusions, health-care-related injections, and injection drug use.^5^ Transfusion transmissible infections and infections associated with lapses in injection safety have declined globally,^6^, ^7^ although these remain key risk factors in lower-income countries.^8^ Infection associated with injection drug use is the primary transmission route in countries where other transmission routes have mostly been eliminated.^8^, ^9^ Treatment for HCV infection used to comprise weekly subcutaneous injections of pegylated interferon and oral ribavirin,^10^ which had low success rates and was associated with a range of side-effects.^11^, ^12^ A watershed moment came in 2014 with the development of highly efficacious direct-acting antivirals (DAAs),^13^, ^14^ which allow interferon-free treatment, greatly improved cure rates, better side-effect profiles, and shorter duration of therapy more amenable to widespread use.^15^, ^16^

**Research in context**

**Evidence before this study**

In 2017, WHO set targets to eliminate hepatitis C virus (HCV) infection globally as a public health threat. We searched PubMed on Feb 23, 2018, for studies published in English that modelled the epidemiological impact of HCV interventions or modelled the global HCV epidemic, using the following search strategy: “(hepatitis C AND model* AND (global OR intervention*))”. We found 33 relevant studies. Mathematical models have been developed to investigate the effectiveness of harm reduction strategies in high-risk groups but these have not examined impact at the population level. Models of the population impact of screening and treatment have been developed and used to investigate WHO mortality targets at both the national and regional levels. These studies, however, could not investigate incidence targets because they did not utilise dynamic transmission models that would allow the impact of prevention interventions and the effect of treatment on incidence to be captured. A recent study used a dynamic transmission model to estimate the population-level impact of both prevention and treatment interventions in Pakistan. This work found that only with extremely high coverage of interventions can elimination targets be met. No study has modelled HCV interventions at the global scale.

**Added value of this study**

To our knowledge, our study is the first to estimate the impact of interventions on the global HCV epidemic and to address WHO HCV elimination targets. We explicitly model the transmission process within and between population groups, allowing us to model prevention as well as screening and treatment interventions. The model is calibrated to all global epidemiological and programme data in a statistically principled way. We quantify the extent to which prevention, screening, and treatment could reduce the burden of HCV globally. Our results highlight the key role prevention has in reducing the burden of disease, the bottleneck screening places on the impact of HCV interventions, and the importance of expanding access to direct-acting antivirals.

**Implications of all the available evidence**

Across the globe, coverage of key prevention, screening, and treatment interventions is currently well below the levels we estimate are needed to have a major impact on the HCV epidemic. Operations research into how to improve coverage in all settings, as well as increased funding for effective strategies, will be required to make progress towards HCV elimination targets.

Advances in HCV therapeutics have led to a commitment from all 194 member states of WHO to eliminate viral hepatitis as a public health threat.^1^, ^17^ WHO HCV elimination targets are defined as a 65% reduction in mortality and an 80% reduction in incidence by 2030 from 2015 baseline (the HCV incidence reduction target combines with a 95% reduction target for viral hepatitis B incidence to produce the overall viral hepatitis incidence reduction target of 90%).^17^ This is to be achieved through a combination of preventing transmission by improving blood safety and infection control measures, extending harm reduction services aimed at reducing transmission among people who inject drugs (PWID), and expanding testing and DAA treatment for those already infected.^1^

Although these targets were formulated by WHO through extensive consultations,^17^ the feasibility of achieving WHO targets globally is not known. Given the current focus on these targets, it is imperative we understand the full effect of HCV interventions at the global scale. We sought, therefore, to use mathematical modelling to provide the first estimates of the impact of combined prevention, diagnosis, and treatment programmes on the global HCV epidemic and to determine the achievability of WHO elimination targets.

## Methods

### Model structure

**Figure 1:**
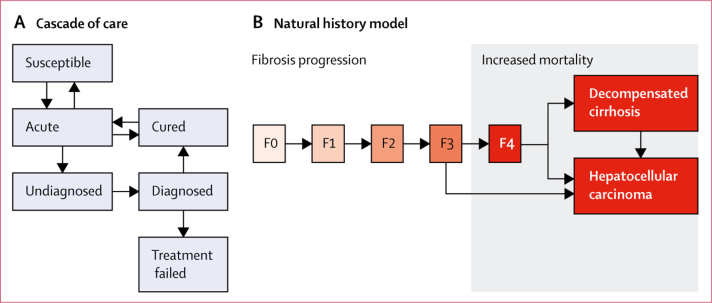
Model structure Boxes represent compartments of the model and arrows denote annual transition rates that can depend upon age, sex, risk group, or duration of infection that can vary over time. At any given point, every person is in one compartment of the cascade of care (A). These compartments are further subdivided by age, sex, and risk group. Spontaneous clearance occurs in a fraction of those acutely infected who return to the susceptible population. Treatment can result in cure and reinfection can occur after successful treatment. Infection results in people entering the natural history model (B): HCV disease progresses through five METAVIR^18^ fibrosis stages, from F0 (no fibrosis) to F4 (compensated cirrhosis). The potential effects of age and male sex on progression and mortality rates are accounted for in calibration. People in the cured compartment of the treatment cascade have reduced or zero disease progression rates depending on disease stage. See appendix for full details, including a list of parameter values with dependencies and the complete model structure diagram. HCV=hepatitis C virus.

We developed a mathematical model to project the future course of the HCV epidemic country by country. We analysed the impact of a set of intervention packages at the global scale by combining the results for 190 individual countries. Our compartmental model simulates the population of a country from 1950 to 2100, grouped according to infection and treatment status (see the treatment cascade in figure 1). Compartments are further stratified by age, sex, and risk group (PWID and non-PWID). Infection (the transition from susceptible to acute compartments) is specified according to HCV prevalence and a risk group-dependent, age-dependent, and time-dependent risk of transmission that is calibrated in model fitting; perinatal infection can also occur. Background age-specific, sex-specific, and time-specific mortality is simulated according to UN projections.^19^ PWID experience an additional mortality risk compared with other groups.^20^, ^21^ HCV infection results in additional risk of liver-related mortality (denoted HCV-related mortality); increased non-liver-related mortality in those infected with HCV is not simulated because it is a much less substantial cause of death in HCV-positive individuals than is liver-related mortality^22^, ^23^ and WHO viral hepatitis targets focus on liver-related mortality.^24^ It should be noted that neglecting non-liver-related mortality will tend to underestimate the impact of interventions.^25^, ^26^

Past treatment and diagnosis is modelled as follows: we increase rates of diagnosis and treatment such that the proportion who are diagnosed, and the proportion treated, match WHO estimates for these quantities in 2015.^1^ DAAs are implemented from 2016 in countries where their use is reported.^27^, ^28^ In these countries, the rate of treatment is calculated in the model such that the reported number of DAA treatment courses is matched by the model.^27^, ^28^ All other countries continue to implement treatment with pegylated interferon and ribavirin after 2015. This approach results in 1·76 million HCV treatment courses being delivered in 2016, of which 86% consist of DAAs.^28^ After 2016 (or 2015 for countries without 2016 data), the future rates of both diagnosis and treatment are fixed unless altered through an intervention. Treatment success depends on the type of treatment used (ie, pegylated interferon and ribavirin or DAAs). Reinfection following cure is also simulated: as a conservative assumption, reinfection risk is set to be equal to primary infection risk.^29^

Following infection with HCV, disease progression occurs according to a widely recognised natural history (figure 1B).^2^, ^30^, ^31^ Progression occurs through the five METAVIR^18^ fibrosis stages. Increased HCV-related mortality occurs from compensated cirrhosis (stage F4), decompensated cirrhosis, and hepatocellular carcinoma. Disease progression and mortality rates are reduced (to zero, in some cases) following cure. All parameters in the natural history model are taken from the literature (appendix); where values are uncertain, parameters are drawn from prior distributions informed by the literature.

### Model calibration

All uncertain parameters were allowed to vary in calibration. These parameters include those of the natural history model and the risk group-dependent, age-dependent, and time-dependent risks of infection. These risks of infection are modelled as flexible splines, where the knot values are drawn from suitable prior distributions; for full details of the modelling of transmission and a complete list of parameter values and prior distributions, see the appendix.

We calibrated the model, in a Bayesian framework, to three sets of information: overall HCV prevalence,^1^, ^32^ PWID HCV prevalence,^33^ and HCV-attributable mortality (according to age, time, and sex).^4^, ^34^ 1000 samples were drawn from the parameter posterior distributions using incremental mixture importance sampling.^35^ This self-monitoring approach to approximating the posterior distribution proceeds by calculating the importance weight for a given parameter set and then drawing new parameter values until the expected fraction of unique parameter sets in the final resample corresponds to the case where all parameter weights are equal—ie, such that no one parameter set dominates the resampled values (appendix).^35^ The parameter sets drawn from the posterior distribution were used to make forward projections of the epidemic under a range of intervention scenarios.

### Intervention scenarios

Six scenarios were constructed to assess the impact of differing levels of prevention, screening, and treatment intervention scale-up (table). The status quo scenario, which represents our best estimate of what the HCV epidemic will look like with no changes made to diagnosis or treatment, maintains diagnosis and treatment rates at their 2016 (or 2015 where 2016 data were not available) values and assumes no reduction in general or PWID population risk. We inferred diagnosis and treatment rates by scaling up these quantities to match the proportion diagnosed or treated where data were available,^40^, ^41^, ^42^, ^43^ or using regional averages where data were lacking, with DAAs introduced in relevant countries in 2016 (appendix);^28^ at the global scale, this results in 20% of people diagnosed in 2015, of whom 7% are treated.^1^

**Table:** Details of intervention scenarios modelled

		**Risk of infection in non-PWID population**	**Risk of infection in PWID population**	**Treatment**	**Treatment coverage**	**Diagnosis coverage**
No-DAA scenario		As per 2015 risk	As per 2015 risk	Pegylated interferon and oral ribavirin	Treatment rate fixed at 2015 or 2016 value	Diagnosis rate fixed at 2015 or 2016 value
Status quo		As per 2015 risk	As per 2015 risk	DAAs from 2016	Treatment rate fixed at 2015 or 2016 value	Diagnosis rate fixed at 2015 or 2016 value
Interventions added cumulatively to the status quo scenario						
	(1) Blood safety and infection control	80% reduction by 2020*	As per 2015 risk	DAAs from 2016	Treatment rate fixed at 2015 or 2016 value	Diagnosis rate fixed at 2015 or 2016 value
	(2) PWID harm reduction	80% reduction by 2020*	75% reduction at 40% coverage by 2020†	DAAs from 2016	Treatment rate fixed at 2015 or 2016 value	Diagnosis rate fixed at 2015 or 2016 value
	(3) Offer DAAs at diagnosis	80% reduction by 2020*	75% reduction at 40% coverage by 2020†	DAAs from 2016 and in all countries from 2017	Treatment offered within 1 year of diagnosis; those previously diagnosed are treated at original treatment rates	Diagnosis rate fixed at 2015 or 2016 value
	(4) Outreach screening	80% reduction by 2020*	75% reduction at 40% coverage by 2020†	DAAs from 2016 and in all countries from 2017	Treatment offered within 1 year of diagnosis; those previously diagnosed are linked back into care for treatment‡	Rate increased linearly such that 90% of people infected are diagnosed by 2030

PWID=people who inject drugs. DAA=direct-acting antiviral. OST=opioid substitution therapy. NSP=needle and syringe programmes.

* General population risk reduced linearly from 2016 to 2020.

† PWID risk reduction simulated as combined OST and NSP, the method of harm reduction with the strongest evidence of reducing HCV transmission among PWID.^36^, ^37^ A suitable coverage for this intervention, therefore, is the so-called WHO high target for OST of 40%,^38^ beyond that reached in most countries to date.^39^ PWID intervention coverage is increased linearly from 2017 to 2020. Coverage only includes opioid-dependent PWID as suitable for OST.^33^

‡ Outreach diagnosis campaign facilitates return to care for those already diagnosed, leading to 10% returning annually for treatment.

Four intervention strategies were sequentially added to the status quo scenario, starting in 2017: (1) blood safety and infection control, leading to an 80% reduction in HCV infection risk in the non-PWID population by 2020; (2) PWID harm reduction, whereby 40%^38^ of the PWID population are reached with a combined package of opioid substitution therapy (OST) and needle and syringe programmes (NSP), leading to a 75% reduction in infection risk in those covered;^36^, ^37^ (3) offering DAAs at diagnosis (regardless of disease stage), with status quo rates of diagnosis maintained (90% accept treatment); and (4) outreach screening, resulting in 90% of the HCV-infected population being diagnosed by 2030 (table). Each intervention builds in the features of the previous strategies; intervention 4 comprises all interventions and is referred to as the comprehensive intervention package.

Finally, a no-DAA scenario, which is equivalent to status quo but incorporates only treatment with pegylated interferon and ribavirin, was included to analyse the scope of the epidemic if the recent adoption^44^ of DAA treatment is not maintained.

### Sensitivity analysis

Uncertainty in the natural history model is accounted for in calibration by drawing 1000 samples from the parameter posterior distributions. The model is run 1000 times with these parameter sets and medians and credible intervals (CrIs; 2·5th and 97·5th percentiles) for all quantities of interest are evaluated. The effects of other notable model assumptions (ie, reinfection rate, effectiveness of PWID harm reduction interventions, and delay in possible retreatment after reinfection) are investigated through one-way sensitivity analyses (appendix). We investigated the sensitivity of the outcomes in the comprehensive package of interventions by doing one-way sensitivity analyses on the three key intervention parameters: risk reduction in the non-PWID population, coverage of harm reduction in the PWID population, and proportion diagnosed by 2030. For each of these parameters, we explored the range from no improvement to 95% (the upper limit is chosen to represent a highly ambitious increase).^45^ We did another set of sensitivity analyses in the same manner as this but using the status quo as our starting point (as opposed to the comprehensive package of interventions) to explore the effects of each programme element. Finally, we evaluated which countries contributed most to infections and deaths averted under the comprehensive package of interventions and then re-ran the model without these countries included in the intervention package, with all values set to the status quo scenario, to quantify the sensitivity of our global results to progress made in key countries.

### Role of the funding source

The funders of the study had no role in the study design, data collection, data analysis, data interpretation, or writing of the report. The corresponding author had full access to all data in the study, and all authors had final responsibility for the decision to submit for publication.

## Results

**Figure 2:**
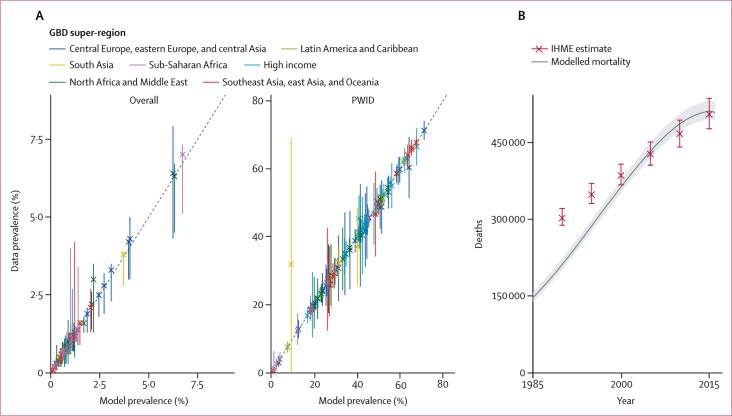
Calibration results (A) Comparison of the model's simulated prevalence of HCV with prevalence data for all 190 countries simulated,^40^, ^41^, ^42^, ^43^ colour-coded by GBD super-region (see appendix for the full list of regions). Overall prevalence values are from 2015 whereas PWID values come from a range of years.^18^ The diagonal lines mark equivalent estimates. (B) Comparison of modelled HCV mortality numbers with IHME HCV mortality estimates.^4^, ^34^ Countries were calibrated to age-stratified and sex-stratified mortality estimates; modelled outputs were summed into overall cirrhosis and hepatocellular carcinoma mortality numbers and compared with aggregated IHME estimates for the years 1990, 1995, 2000, 2005, 2010, and 2015. GBD=Global Burden of Disease Study. HCV=hepatitis C virus. IHME=Institute for Health Metrics and Evaluation. PWID=people who inject drugs.

**Figure 3:**
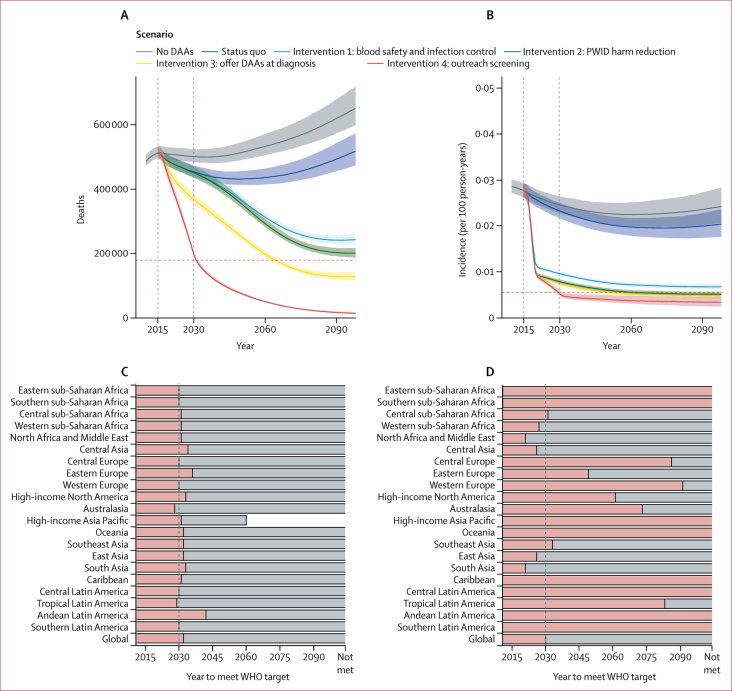
Global intervention results (A) Number of deaths due to HCV by scenario. Note that due to the high impact of the comprehensive package of interventions (intervention 4) on reducing the number of deaths, the credible intervals are extremely narrow around the median. (B) Incidence of viraemic HCV infection (number of incident chronic infections divided by number of people susceptible) by scenario. Note that the incidence curves for interventions 2 and 3 are almost coincident. Vertical dashed lines indicate baseline (2015) and the year for targeted elimination (2030). Horizontal dashed lines indicate elimination targets: 65% reduction of mortality and 80% reduction in incidence. In panels (C) and (D), data are shown by GBD region. The end of each bar represents the median year of mortality (C) or incidence (D) elimination. Grey bars show the status quo scenario; the red portion of each bar shows the comprehensive package of interventions (intervention 4). Where a red bar extends to the edge of the graph, elimination was not achieved in any scenario before 2100. The dashed vertical line indicates the WHO elimination year target of 2030. DAA=direct-acting antivirals. GBD=Global Burden of Disease Study. HCV=hepatitis C virus. PWID=people who inject drugs.

We found 69 million (95% CrI 67–71) active viraemic infections and 512 000 deaths (497 000–533 000) in 2015, with 277 incident infections (262–294) per 1 million people, in line with WHO estimates for that year (figure 2).^1^ The projected proportion of new infections expected in PWID between 2016 and 2030 was 29% (95% CrI 27–32) in our model, in keeping with WHO estimates in 2015 that 22% of new infections were in PWID.^1^ Projecting the epidemic forwards in the status quo scenario, we find that the number of active infections will slowly decrease to 58 million (54–62) by 2050 but could rise by the end of the century. Likewise, incidence would gradually decrease to 198 infections (179–218) per 1 million people by 2060 but might increase thereafter. Mortality from HCV gradually decreases for more than three decades but can increase thereafter in line with the possible increasing numbers of active infections (figure 3). These results reflect our assumptions in the status quo scenario that risk of infection does not decrease in any group after 2015 and that numbers of PWID will not change. Not implementing DAAs at all results in worse outcomes than in the status quo scenario, with considerably higher mortality and incidence (figure 3).

Programmes aimed at curbing the spread of blood-borne infections among the non-PWID population (intervention 1) can dramatically reduce incidence (figure 3). Global improvements in blood safety and infection control (lowering the general population risk of HCV infection by 80%) reduce the annual number of new infections in 2030 by 58% (95% CrI 56–60) compared with the status quo scenario in the same year. Along with these improvements in blood and infection safety, extending OST and NSP harm reduction services to 40% of the opioid-dependent PWID population (intervention 2) will reduce the number of new infections in 2030 by a further 7 percentage points (figure 3). Taken together, these prevention interventions could avert 14·1 million (95% CrI 13·0–15·2) cumulative infections by 2030. Due to the long incubation period of HCV infection, however, such reductions in incidence will not immediately translate into reductions in mortality: by 2030, the decrease in mortality will be small in either scenario (figure 3) and the combination of both interventions will only reduce mortality in 2050 by 18% (17–19).

Expanding access to DAAs, in addition to the prevention interventions, is projected to cut future mortality more substantially. Replacing pegylated interferon and ribavirin with DAAs in all countries where they have not been rolled out, and offering these at time of diagnosis (intervention 3), has substantial short-term impact: we estimate 640 000 fewer deaths (95% CrI 620 000–670 000) from liver cancer and cirrhosis by 2030. However, compared with intervention 2, there is no additional effect on incidence (figure 3). This scenario illustrates that improvements in outcomes can be attained by ensuring access to DAAs without otherwise changing diagnosis or treatment programmes. However, such gains fall well short of WHO mortality targets (figure 3) and these programmes also offer only minimal prevention benefits.

Adding outreach screening (intervention 4), such that the proportion diagnosed reaches 90% by 2030, further reduces mortality and incidence (figure 3). This scenario incorporates all prevention, screening, and treatment elements of the previous strategies. With this comprehensive package of interventions, there would be a 61% (95% CrI 60–62) reduction in mortality by 2030 compared with the baseline 2015 value—corresponding to 1·5 million (95% CrI 1·4–1·6) deaths prevented. Our estimates show a treatment-as-prevention benefit: compared with prevention interventions alone (intervention 2), the comprehensive package of interventions averts an average of 950 000 additional infections by 2030, resulting in 15·1 million (13·8–16·1) new infections averted in total. Achieving such reductions requires a massive screening programme and demands a rapid increase in new treatment courses in the short term—namely, 51·8 million (50·5–53·3) courses of DAA treatment by 2030 (appendix). In the following 20 years, by contrast, the total number required is a much more modest 12·0 million (11·5–12·7) courses. The reduced treatment requirement after 2030 indicates that rapid testing and treatment scale-up is a means to control the epidemic in the long term.

Interventions do not have a uniform impact across different countries; projections by country are presented in the appendix. Upon all countries rolling out DAAs and offering these at diagnosis (intervention 3), most countries in Africa, along with south Asia, east Asia, and southeast Asia, experience smaller reductions in mortality than do countries in other regions (appendix). This reflects the lower existing diagnosis coverage in these countries. The effect of prevention interventions on incidence is also dependent on local epidemiology. Certain countries, such as Egypt, Mongolia, and Pakistan, benefit greatly from improvements in blood safety and infection control (intervention 1) because these reduce risk of infection in the entire population (appendix). Countries that have a large proportion of the HCV epidemic concentrated in PWID—eg, the USA, Australia, and Spain—show more improvement upon expansion of PWID harm reduction services (intervention 2; appendix). All countries experience reductions in both mortality and incidence upon implementation of the comprehensive package of interventions (appendix), reflecting the high impact a multifaceted approach to tackling the epidemic can have.

WHO mortality and incidence elimination targets are narrowly missed in 2030 with the comprehensive package of interventions: the WHO-defined mortality elimination target occurs in this model by 2032, whereas the incidence elimination target is met by 2030 (figure 3). The comprehensive package of interventions does, however, lead to a 61% (95% CrI 60–62) reduction in mortality in 2030 compared with the status quo scenario (figure 3). The mortality elimination targets could be reached by 2030 if the coverage of diagnosis were increased to 95% rather than the 90% assumed above (appendix). Further incidence reduction is possible, but this is primarily achieved by increasing PWID harm reduction coverage: if coverage is increased to more than 80%, incidence rates can be reduced by nearly 90% compared with 2015 baseline values (appendix).

We also examined year of elimination by Global Burden of Disease Study (GBD) regions. All regions reach the mortality elimination target at similar times after implementing the comprehensive package of interventions (figure 3). Although global incidence targets are met, eight regions do not reach the incidence elimination target before 2100. A key determinant of whether incidence elimination is achieved is the proportion of infections by risk group: the regions that reach incidence elimination before 2030 are characterised by low numbers of infections in PWID relative to the rest of the population (appendix).

**Figure 4:**
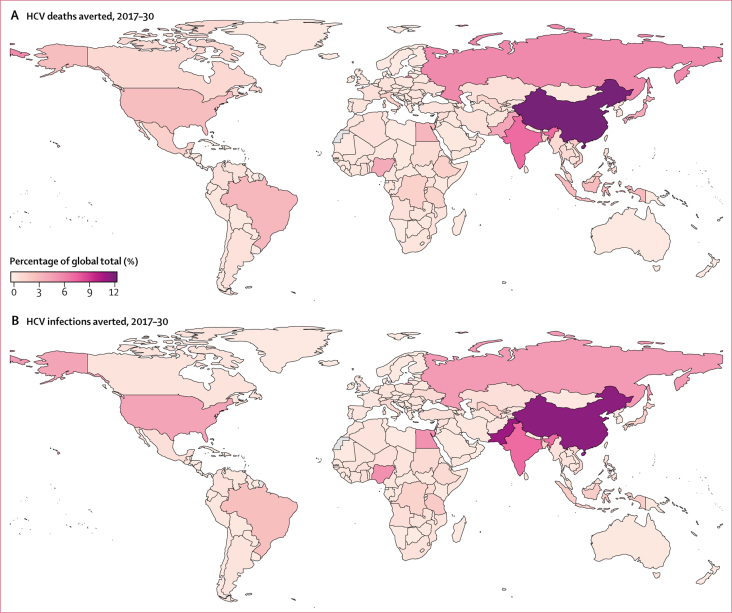
Distribution of global deaths averted and infections averted when implementing the comprehensive package of interventions compared with the status quo scenario Cumulative deaths and infections averted in 2017–30 are shown by country as percentages of the total global number averted.

Reducing the global burden of hepatitis C depends on the progress made in just a few countries. Most infections and deaths averted, after implementation of the comprehensive package of interventions, are concentrated in a small number of countries, in particular China, India, Pakistan, and Egypt, which are the countries that contribute most to projected new infections by 2030 (figure 4). If China, India, or Pakistan do not implement the comprehensive package of interventions, the year in which global incidence elimination is reached is pushed back to at least 2047 (appendix), whereas not implementing the comprehensive package of interventions in these countries would result in global incidence reductions in 2030 of 69% (95% CrI 66–74), as opposed to an 81% (78–82) reduction when all countries implement the interventions (appendix).

**Figure 5:**
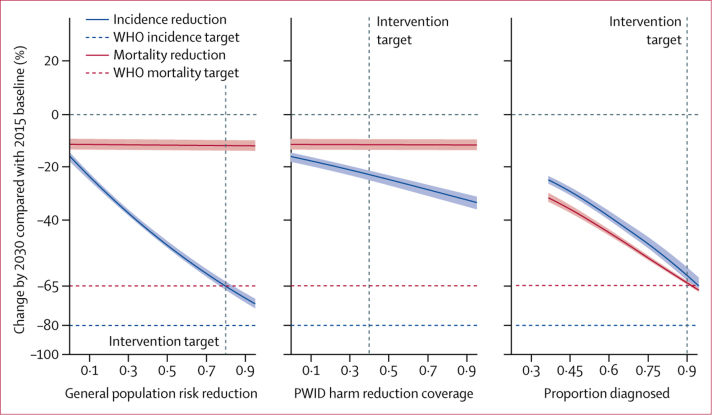
Reduction in mortality and incidence in the status quo scenario upon improving prevention or screening intervention coverage Changes in mortality and incidence by 2030 upon increasing the reduction in general population (ie, non-PWID) risk, the coverage of PWID harm reduction interventions, and the proportion diagnosed by 2030, from status quo values. The vertical dashed lines show the values targeted in the intervention scenarios (see table).

We found that improving blood safety and infection control alone (ie, starting from the status quo scenario), such that the risk of infection in the general population decreases by 95%, can reduce incidence in 2030 by 72% (95% CrI 70–73; figure 5). Although HCV-positive PWID comprise only a minority of global HCV-positive individuals, programmes that can reduce PWID transmission have a global impact upon incidence: expanding PWID harm reduction coverage to 95% (in the absence of any other intervention scale-up) could reduce global incidence by 33% (31–36) in 2030 (figure 5). Screening and treatment interventions carry a double benefit: increasing the proportion diagnosed and treated (without scaling up the prevention interventions) reduces both mortality and incidence through a treatment-as-prevention effect (figure 5). Yet, treatment alone has less impact than when combined with prevention interventions: the comprehensive package of interventions reduces incidence in 2030 by 81% (78–82) compared with 60% (57–62) when prevention interventions are not implemented.

Because the degree of effectiveness of PWID harm-reduction programmes is more uncertain than that of DAA treatment, and the effectiveness of such programmes probably varies substantially between settings, we did sensitivity analyses to investigate the dependence of the results on this aspect of the model. Reducing the effectiveness of OST and NSP interventions from 75% to 20% (within the context of the comprehensive package of interventions, scenario 4) pushed back the year of incidence elimination to 2052 (appendix). As shown by the regional breakdown, this is driven by delays in elimination in regions in which HCV-positive PWID comprise a large proportion of the HCV epidemic (such as western Europe, high-income North America, and Australasia; appendix). By contrast, simulations in which PWID harm reduction effectiveness is taken to be 90% resulted in a median estimate of 470 000 fewer courses of treatment being delivered by 2030 in intervention 4 than when the effectiveness is assumed to be 75% (appendix).

Year of incidence elimination is sensitive to the extent of intervention scale-up (appendix). Reducing coverage of any intervention, within the context of the comprehensive package, predictably pushes elimination further into the future. However, the relationship between the level of diagnosis and impact on incidence (and year of achieving the elimination target) is found to be parabolic; that is, intermediate levels of diagnosis led to lesser reductions in incidence than either no increase or enormous increases in diagnosis rates. This is because we have assumed that cured people are at risk of reinfection and, at lower levels of diagnosis coverage, infection risk for PWID remains high and so there can be more infections than with no intervention. At high levels of diagnosis, this effect is overwhelmed by the reduction in infection risk that is brought about by the reduction in the numbers of infectious people. The risk of reinfection is, however, not known, and if it were lower than the risk of initial infection then the year of elimination could be sooner than we have estimated and thus the non-linear, parabolic effect described disappears (appendix). Further sensitivity analyses showed that the delay in accessing retreatment after reinfection had no impact on the results (appendix).

## Discussion

A dramatic decrease in both mortality and incidence in HCV could be possible through implementation of a comprehensive package of prevention, screening, and treatment interventions. Even though it narrowly falls short of the WHO targets for 2030, such an impact would be a tremendous stride forwards, averting 15·1 million new infections and 1·5 million HCV-related deaths by 2030.

Several important challenges must be met and this analysis raises points of direct relevance to policy and programme development. First, the benefits of DAAs will only be fully reaped with an exceptional increase in diagnosis coverage to 90% by 2030. The treatment of only those already in care will not translate into substantial reductions in HCV deaths or incidence. However, Malta is the only country in which the diagnosis coverage is estimated to be at such a high level.^27^ Progress could be made in different ways in the coming years: innovative means of increasing awareness and encouraging HCV testing in a range of settings are being explored^46^ and new technologies, such as point-of-care viral load finger-stick tests,^47^ should soon be available.^48^ Both awareness raising and simpler diagnostics could facilitate large increases in knowledge of HCV status as has occurred in the HIV arena.

Second, the HCV epidemic among PWID has a deciding role in determining whether incidence elimination targets are met. The modelled strategy that resulted in incidence elimination being met by 2032 relied upon coverage of OST with NSP increasing to 40%: however, only 1% of PWID live in countries with such high coverage of these harm reduction services.^39^ If the effectiveness of those programmes is lower than has been estimated in some settings,^36^, ^37^ then elimination becomes a much more remote prospect, with elimination not being reached until after 2050, even with high coverage of other interventions. This result, along with the finding that reinfection plays a key role in delaying the year of incidence elimination, highlights that PWID prevention must be central to HCV policy. Targeted treatment-as-prevention approaches among PWID might reduce incidence,^49^ although this must be done in the context of enhanced harm reduction interventions and community involvement.^50^ Eliminating structural and systemic barriers such as the criminalisation of PWID or treatment restrictions on active drug users can improve access to health services.^51^, ^52^ A Hepatitis C Action Plan programme in Scotland has shown that national reductions in incidence are possible through an integrated approach involving scaling up of OST with NSP along with increasing awareness and provision of HCV testing for PWID (including ever-PWID).^53^ Such progress relies on political will and reliable sources of funding that, in low-income and middle-income countries in particular, are often lacking—this is a serious challenge HCV programmes often face.^54^

Third, continued improvements in blood safety and infection control are key components of the global elimination intervention package and drive a large reduction in new infections. Although proven safety and control measures exist and have played a major part in reducing incidence in many settings,^55^, ^56^ only 39% of countries worldwide operate haemovigilance systems,^55^ and unsafe (often unnecessary) injections continue to be a major source of HCV infection.^7^, ^57^ The reasons for the persistence of unsafe injections as a transmission route are complex,^58^ and context-specific management methods are necessary if there are to be continued reductions in the risk of HCV transmission via these routes.^59^

Finally, the global HCV epidemic is concentrated in a set of countries that could face myriad challenges in implementing the PWID harm reduction, infection control, and outreach screening initiatives required. This hurdle might make talk of elimination seem more tenuous but should also focus attention while illustrating that major progress can be made with policy changes in just a few places. In terms of global HCV epidemiology, and given the stated aims of WHO and the Sustainable Development Goals to “combat hepatitis”,^60^ progress made in these settings should be of primary concern.

These policy points reinforce and augment the conclusions drawn by other studies. Several modelling analyses have highlighted the challenge of reducing transmission among PWID and have advocated simultaneously scaling up prevention and treatment interventions to reduce prevalence.^61^, ^62^ Our work extends this to show that, even on the global scale where incidence is dominated by non-PWID transmission, reducing incidence among PWID plays a key part in determining whether elimination targets are met. A recent study^63^ has incorporated dynamic modelling of PWID into a full population model of the HCV epidemic in Pakistan. The authors find that only with extremely high coverage of interventions can elimination targets be met. This result agrees with what we have shown on a global scale that even with exceptionally high intervention coverage, elimination targets are difficult to meet. An EU modelling study^27^ has suggested that mortality elimination targets can be met in this region. Similarly, we find that, provided there are ambitious increases in screening and treatment, mortality elimination targets are met in most regions by around 2030. The EU study did not, however, model incidence dynamically and could not draw conclusions regarding incidence targets. Our analysis has shown that even with extensive scale-up of prevention interventions, several regions do not meet incidence elimination targets before 2100, driven in large part by ongoing PWID transmission.

To produce a global analysis, we have had to manage a variety of data and modelling limitations. We used modelled estimates of mortality from the GBD project;^4^, ^34^ these have been produced for most countries and allow basic epidemic trends to be inferred even where other data are lacking. To manage the uncertainty this introduces, we built the model to be flexible when calibrating to these inputs because they are estimates rather than data. To simulate the treatment cascade, we used regional estimates to extrapolate to countries without data. This process necessarily smooths out country-level differences in diagnosis and treatment coverage, introducing error in some individual country projections. Country-level cascade information is, however, available for countries that account for about 60% of the global viraemic population, giving good resolution on the treatment cascade for most of the globally infected population. Furthermore, low overall treatment numbers compared with the size of the epidemic mean that error introduced through extrapolating to the remaining 40% of the viraemic population will have only a small impact on projections.

There is considerable uncertainty about the source of infections among the general population, which makes it impossible to be sure about the extent to which this route of transmission is being, and can be, reduced. Although WHO calls for no unsafe injections and 100% of blood donations screened with quality assurance,^1^ both the difficulty in reaching these targets and the many ways of being infected with HCV beyond these two routes (appendix) led us to specify only an 80% risk reduction among the general population in intervention 1; this is to be conservative regarding what is a necessarily vague intervention strategy. We varied this in sensitivity analysis to explore the impact this programme parameter had on outcomes. Similarly, the impact of PWID harm reduction interventions is uncertain outside of the primarily North American and European settings in which OST and NSP initiatives have been studied.^36^, ^37^ We varied this quantity in sensitivity analysis to account for this limitation, but in implementing PWID harm reduction globally we are assuming that such programmes can have equal success in all regions. Lastly, we did not model changes in the proportion of the population who are PWID. There is growing concern about potential increases in the number of PWID in the USA,^64^ for instance, yet the relationship between non-medical use of prescription opioids and initiation of injection drug use is not well understood.^65^ Conversely, in other regions such as Europe, the proportion of the population who are PWID might have decreased,^66^ such as in Scotland, which has reported a decline in injecting drug use over the past decade.^67^ With such uncertainty regarding possible changes in the proportion of the population who are PWID, we kept this quantity fixed and did not simulate possible increases or decreases in the future. Nevertheless, future structural interventions could reduce the number of PWID^68^ and so potentially limit the ongoing spread of HCV.

In conclusion, reaching WHO elimination targets is an extremely challenging aim that requires a multifaceted approach combining screening, prevention, and treatment with a focus on those countries in which burden is greatest. Such efforts will entail considerable practical challenges and have large cost implications—running into the tens of billions of US dollars by 2030 for a complete viral hepatitis strategy^17^—but many countries have made substantial progress despite this: Egypt empowered local facilities, created numerous opportunities for HCV screening, and treated 700 000 HCV-infected individuals with DAAs in 2016;^28^, ^69^ Australia has negotiated a volume-based pricing model for DAAs that encourages, rather than rations, the prescription of expensive DAA treatment courses;^70^ and Scotland has successfully coordinated national expansion of harm reduction services with HCV testing and treatment provision resulting in a sharp increase in people achieving sustained virological response.^71^ By using new tools and the examples of relevant countries to devise ambitious, integrated interventions, this modelling work has shown that substantial progress towards global elimination can be made while greatly reducing the burden of new infections and premature deaths.
