# Supplementary material for: Scaling up prevention and treatment towards the elimination of hepatitis C: a global mathematical model
Source: Lancet. 2019 Mar 30;393(10178):1319–29. doi: 10.1016/S0140-6736(18)32277-3 (PMC6484702; doi:10.1016/S0140-6736(18)32277-3)
Supplement: Supplementary appendix [file mmc1.pdf]

THE LANCET

Supplementary appendix

This appendix formed part of the original submission and has been peer reviewed. We post it as supplied by the authors.

Supplement to: Heffernan A, Cooke GS, Nayagam S, Thursz M, Hallett TB. Scaling up prevention and treatment towards the elimination of hepatitis C: a global mathematical model. *Lancet* 2019; published online Jan 28. http://dx.doi.org/10.1016/S0140-6736(18)32277-3.

Supplementary Appendix: Scaling up prevention and treatment towards the elimination of hepatitis C: a global mathematical model

Alastair Heffernan, Graham S. Cooke, Shevanthi Nayagam, Mark Thursz, Timothy B. Hallett
Correspondence to: a.heffernan14@imperial.ac.uk

Table of contents

Materials and Methods **1**
Model structure 1
Natural history model 4
Transmission 6
Calibration 8
Modelling treatment and prevention 10
Intervention strategies 12
One-way sensitivity analyses 13
Model inputs 14
Calibration data 15
List of countries by region 16

Supplementary figures **18**

Supplementary equations and tables **30**

Supplementary results **41**

References **231**

Materials and Methods

A model was constructed to make projections of the hepatitis C epidemic, country-by-country, and to analyse the impact of a set of intervention packages at the global scale by combining individual country results. To simulate the full course of the hepatitis C epidemic in a given country, a mathematical model was constructed that incorporates: population dynamics (birth, natural death and migration); age and sex stratification; dynamic infection, by genotype, with hepatitis C virus (HCV); increased risk of infection among people who inject drugs (PWID); disease progression leading to cirrhosis; increased hepatitis C specific mortality due to complications following onset of cirrhosis; historical rates of diagnostic screening; historical rates of treatment; treatment success and treatment failure; reduced rates of disease progression and mortality following treatment success; and possible reinfection following cure. The model is calibrated to HCV viraemic prevalence in the overall population, HCV viraemic prevalence among PWID and HCV-attributable mortality estimates. Historical and future demographic information, HCV genotype distributions, PWID data, and past coverage of harm reduction and HCV interventions are used as model inputs. Uncertainty is accounted for in a statistical framework allowing disease burden projections to be made by sampling from calibrated parameter posterior distributions.

Model structure

Overview. The mathematical model used to simulate the HCV epidemic in a given country is shown in Figure S10 (the associated equations are given in Supplementary equations and tables

Equations S1) and reflects broad consensus regarding the natural history of the disease.[1–3] Parameter symbols are defined, and values given where appropriate, in Table S1. The compartments of the model are denoted by the state variables S (susceptible to HCV infection), A (acute HCV infection), U (infected, undiagnosed), D (diagnosed, pre-treatment), T (diagnosed, on treatment), Q (diagnosed, failed treatment) and C (cured). All compartments are further subdivided according to the subscripts i (age: stratified into five-year age bands from 0 to 100 years old), j (sex: female and male) and l (risk group: general population, PWID or former PWID). All infectious compartments (A, U, D, T, Q) are also subdivided according to the subscript g (genotype: 1-6[4]). Superscripts denote disease stage: 0 to 4 refer to METAVIR[5] fibrosis progression stages F^0 to F^4, DC to decompensated cirrhosis and HCC to hepatocellular carcinoma. Compartments DC and HCC are further divided according to the subscript d (duration in stage: a binary division indicating presence of a complication for less than, or more than, one year). Subsequent references to a compartment refer to the set of compartments of a given letter (S, A, U, D, T, Q, C) with all relevant subscripts and superscripts. The term

"people" is used to indicate the average number in a particular compartment; as this is a deterministic compartmental model, individuals are not tracked.

People enter the model at birth. Infants join either the susceptible (S), or acutely infected (A) compartments, according to the proportion perinatally infected $p^{inf.}$ (for values see Table S1). Those susceptible can be infected according to a force of infection, $\Lambda_{il}(t)$, that depends on HCV prevalence, and a calibrated risk of transmission that varies over time (t), by age (i), and according to risk group (l), see Transmission section. Having been infected, people either progress to undiagnosed chronic infection (U) at rate $\lambda_i^{chronic}$, or clear infection and return to the susceptible compartment according to an age-specific clearance rate λ_i^{clear}. Unless a person dies from other causes, migrates or is treated, those that develop chronic HCV infection move through the five METAVIR fibrosis stages F^0 to F^4 (compensated cirrhosis), and may ultimately progress to DC and/or hepatocellular carcinoma HCC. Progression rates between compartments A and compartment B depend (in general) on age and sex: $\lambda_{ij}^{A\to B}$ (see

Natural history model section). Natural mortality and migration (not explicitly shown on Figure S10) remove/add people from all compartments at the same rates. Mortality from compensated cirrhosis, DC and HCC are increased above background mortality rates to rates μ_{ij}^4, μ_{ijd}^{DC} and μ_{ijd}^{HCC} respectively. Those that die from F4 or DC are recorded as dying from a cirrhosis-attributable death; those from HCC from an HCC-attributable death.

People may be diagnosed and move to compartment D. This occurs at historical rates of diagnosis or at chosen rates in the interventions (corresponding to programmatic aims). The relative likelihood of seeking diagnosis increases with disease severity and is incorporated into the diagnosis rates $\delta^0(t)$ to $\delta^{HCC}(t)$, capturing the fact that individuals usually present for diagnosis after onset of symptoms. Once diagnosed, individuals may be treated with pegylated interferon plus ribavirin (PEG-IFN+RBV) or with direct-acting antivirals (DAAs); the rate of treatment $\tau^k(t)$ at a particular disease stage k is either determined historically or else is a programmatic aim of the particular intervention being simulated. Treatment lasts a mean duration dependent on genotype (in the PEG-IFN+RBV era) given by $1/\phi_g$. The proportion successfully treated (dependent on disease stage, k, and genotype, g: ζ_g^k) enter the cured compartment (C) and those that fail treatment ($\bar{\zeta}_g^k = 1 - \zeta_g^k$) enter the failed treatment compartment (Q). Those cured progress through the disease stages at reduced rates: the rate between compartment A and compartment B is reduced to a factor $\alpha^{A\to B}$ of the original progression rate, giving a rate of disease progression $\rho_{ij}^{A\to B} = \alpha^{A\to B}\lambda_{ij}^{A\to B}$. Mortality rates from the end stages of the disease are similarly reduced in those cured to a fraction α^4, α^{DC} and α^{HCC} of the original rates (for full details see the section Modelling treatment and prevention). Once cured, people can be reinfected according to the same force of infection $\Lambda_{il}(t)$.

Risk groups. The risk structure of the model reflects the increased risk of infection in PWID.[6,7] Published estimates for the proportion of the population active PWID are generally quoted relative to the size of the 15- to 64-year-old population;[8] injecting careers are considerably shorter than this, however, with survey estimates reporting injection careers ranging from 11 to 27 years in length.[9] Taking the upper end of this estimate, and acknowledging the fact that younger PWID are believed to engage in riskier behaviour,[10] the model simulates increased risk of HCV infection between the ages of 15 and 40 years old.

The remaining people in the model, termed the general population (comprising never PWID and former PWID), experience a lower risk of infection. This risk of infection is calibrated to HCV prevalence estimates in the population as a whole and incorporates all non-PWID modes of HCV transmission.[6]

It should be noted that HIV-positive men who have sex with men (MSM) have been identified as a key HCV risk group:[11–13] HCV antibody prevalence among HIV-positive MSM (who are not injection drug users) is estimated at 6·7%,[14] higher than 1·4% in the population as a whole.[15] Though sexual transmission among monogamous, heterosexual couples is rare,[13,16] there is an increased risk of HCV infection in HIV-positive MSM.[13] On a global scale, however, the overall number of HIV-positive MSM means that this group comprises a much smaller fraction of the HCV epidemic than HCV-infected PWID. Using an estimate of the proportion of men in the USA who engage in same-sex behaviour (as reported in the previous year) of 2·9%,[17] multiplying by regional estimates of HIV-positive prevalence in MSM[18] and adult male population size[19] results in an estimated 7·4 million HIV-positive MSM. This, in turn, yields an estimated 495 000 HIV-HCV coinfected MSM globally,[14] or 6% of the estimated global number of HCV antibody positive PWID.[20] Due to the difference in size of these risk groups globally, and the fact that overall HCV prevalence is much higher in PWID (over 50% HCV antibody positive[20]) than in HIV-positive MSM, the latter are not modelled as a separate risk group in our analysis.

Mortality. We distinguish between three modes of death in the model:

i. Background mortality.

That risk of death having nothing to do with HCV or being a PWID, and pertaining to all persons (in a sex-, age- and time-specific manner), simulated according to UN projections.[19]

ii. HCV mortality.

The additional risk of death that is caused by infection with HCV. This is the additional risk of mortality that stems from chronic liver disease, decompensated cirrhosis and liver cancer.

It is acknowledged that while HCV is associated with various non-liver conditions,[21,22] the impact these conditions have on mortality is much smaller than liver-related disease: a large US study of over 9000 people followed for an average of 14·8 years calculated mortality rate ratios between HCV-positive and HCV-negative individuals for liver-related and non-liver-related mortality.[23] This found a 26-fold increase in liver-related mortality ($p < 0{\cdot}001$) among HCV-positive individuals, compared to a 1·8-fold increase in non-liver-related mortality, which was not statistically significant ($p = 0{\cdot}18$). An analysis in Scotland did find a statistically significant reduction in the hazard ratio of non-liver-related mortality following HCV cure, but there was no absolute risk reduction (ARR) in non-liver-related mortality considered over a 7·5 year period (liver-related mortality, by contrast, saw a highly statistically significant ARR in the same analysis).[24] These considerations led us only to consider an increase in liver-related mortality as an outcome of HCV infection and not to simulate an increase in other non-liver-related mortality; doing so accords with the WHO definition of mortality attributable to HCV as those deaths from "hepatocellular carcinoma (HCC), cirrhosis and chronic liver diseases".[25] It should be stressed, however, that the narrow focus on liver-related disease ignores the considerable morbidity associated with non-liver-related conditions arising as a consequence of HCV and, by underestimating mortality as well, ultimately underestimates the potential impact of HCV interventions.[21,26]

iii. PWID related mortality.

The additional risk of death observed in PWID.

PWID have a higher risk of death than the non-PWID population and this is accounted for by multiplying the background rate of mortality by a standardised mortality ratio (SMR). To estimate this value, we examined two key sources that take a global perspective on the excess mortality risk for PWID. Firstly, a systematic review by Mathers et al. in 2013 that compiled estimates of SMRs for PWID compared to non-PWID from 67 studies and produced an overall pooled estimate of the PWID SMR;[27] and secondly estimates published by the United Nations Office on Drugs and Crime (UNODC) on the global number of drug-related deaths in 2015 (derived from reports by 86 countries).[28] SMRs were produced from these UNODC estimates by fitting an SMR in our model to account for the extra 190 900 deaths (range: 115 900 – 230 100) in PWID.[28]

Both sources have drawbacks: Mathers et al. suffer from bias since the pooled SMR relies on data from primarily high-income settings, in particular Italy, USA and UK. Additionally, the SMR reported in the analysis will include HCV liver-related mortality (no disaggregation was attempted to produce an estimate of the additional mortality due to causes other than HCV). A previous iteration of this study (that finds almost the same pooled SMR) suggested that the majority of PWID deaths were due to overdose, while liver-related mortality (of which HCV-specific liver-related mortality is only a part) contributed around 10% (range: 4·2-15·1%) to deaths among PWID in those studies that reported this measure.[29] The UNODC estimates suffer from being non-systematic in the collection of results and due to countries applying different definitions of the term drug-related deaths (which may include any of: "fatal drug overdoses; deaths due to HIV acquired through injecting drug use; suicide; and unintentional deaths and trauma due to illicit drug use"[30]). This suggests that the numbers reported by UNODC probably underestimate the burden of drug related deaths. These estimates are, however, an

established and endorsed source for such information and include more countries than the Mathers et al. meta-analysis.

The limitations in both sources, and the high variability of SMR values reported both between countries and between studies in the same country,[27] indicates that the most appropriate approach to estimating the PWID SMR is to take a wide range of values for this quantity. To that end, the upper SMR value reported in Mathers et al. was combined with the lower value derived from UNODC to produce a PWID SMR range of 5-16.

Natural history model

The structure of the natural history model has been described above (see also Figure S10). There is a broad consensus regarding the natural history of HCV infection,[1–3] which is reflected in the similarity of mathematical models used in various cost-effectiveness analyses[31] and epidemiological analyses.[32,33] One notable difference distinguishing published models is between those that simulate explicit decompensation events (variceal haemorrhage, ascites, encephalopathy) and those that group these events into one decompensated cirrhosis category.[34] As treatment outcomes and intervention strategies are not influenced by the specific manner of decompensation, and since the progression rates to (and between) specific episodes of decompensation are less studied than the rate to the decompensated stage as a whole, the model presented here simply amalgamates all decompensation events into one DC health state. We do not model HIV coinfection explicitly in this model; while there is evidence that HIV coinfection increases fibrosis progression rates,[35] it is also argued that antiretroviral therapy (ART) mitigates this effect.[35,36] Since the global number on ART is high in many places and increasing,[37] we make the assumption that the impacts of HIV coinfection on the HCV epidemic are small and will, furthermore, decrease over the course of the model's projections. Similarly, HCV spontaneous clearance rates may be slightly lower in HIV-positive individuals (around 15%[38]) but this is only marginally different from our assumption regarding spontaneous clearance in the population as a whole and so again we assume that the effects are small.[38] Where HIV prevalence is high and ART coverage low among PWID, we will have slightly underestimated the impact of the HCV epidemic and potentially underestimated the impact of the HCV interventions.

The following section details the parameter values, or ranges of values where parameters are varied in calibration, used in the model (for a full list of all parameters and derived quantities see Table S1).

Fibrosis progression rates. The starting point for parameterising fibrosis progression rates is a meta-analysis by Thein et al.[39] that synthesised data from 111 studies (33 121 individuals) investigating hepatitis C progression. This meta-analysis used the distribution of METAVIR stages, combined with known (or approximately known) durations of infection, to infer the annual transition probability between fibrosis stages using maximum likelihood methods.[40] This analysis presents results for both clinical (diagnosed in tertiary care) and non-clinical (diagnosed as part of routine screening) settings; since the model simulates the HCV epidemic across an entire country (and not just the epidemic among those presenting for care) we utilise the non-clinical results. These rates are lower than those derived from the clinical results, since they are less influenced by the sample bias inherent in deriving fibrosis progression rates only from patients presenting at tertiary care centres.

The results taken from the meta-analysis are, accordingly, the non-clinical setting random effects model transition probabilities, quoted between fibrosis stage k and $k+1$ ($k = 0$, 1, 2, 3): $f_{l/u}^{k\to k+1}$, where l and u indicate the lower and upper 95% confidence intervals. We allow the model to explore the full range of the 95% confidence intervals in fitting by introducing a scaling factor ($\alpha^{fibr.} \in [0,1]$), varied in calibration (see Reinfection**.** Work in the PEG-IFN+RBV era suggested that reinfection occurs at an equal rate to primary infection.[69,70] Other work in this period indicated that reinfection may be less likely to result in viraemic infection.[71] More recent articles after the development of DAAs illustrate that PWID who have been treated and relapsed to injecting behaviour are often reinfected, though in these studies comparisons with non-SVR individuals were not given preventing a formal assessment of the relative risks of reinfection versus primary infection following cure.[72,73] Searching for information regarding potentially lower rates of reinfection among PWID, as suggested by this press release reporting on a 2017 presentation,[74] did not return robust evidence demonstrating such an effect. With such a range of views regarding relative risk of reinfection, and no clear evidence one way or the other, a conservative assumption was adopted, viz. that reinfection risk is equal to primary infection risk (a relative risk of one), an assumption followed by other modelling analyses for largely the same reasons.[75,76]

Calibration section for details of how the model is fit to data). This transition probability is then converted to a rate:

$$r^{k\to k+1} = -\ln\left\{1-\left(p_l^{k\to k+1} + \alpha^{fibr.}\left(f_u^{k\to k+1} - f_l^{k\to k+1}\right)\right)\right\}.$$

The effects of age and sex are incorporated as part of model calibration. This leads to the specification of age- and sex-dependent rates of progression, denoted $\lambda_{ij}^{k\to k+1}$. The average age at liver tissue sample of the individuals in the meta-analysis was 40 years old and the proportion male was close to 50% (the exact figure was 57%). Accordingly, the sex-averaged progression rate (average of rates for sex $j =$ f and m) at age 40 is set equal to the meta-analysis derived value:

$$\frac{1}{2}\left(\lambda_{40,f}^{k\to k+1} + \lambda_{40,m}^{k\to k+1}\right) = r^{k\to k+1}.$$

To build in the possible effects of sex and age, two calibrated parameters are introduced: one that controls the difference in progression rates between males and females and one that controls the increase of progression rates by age. This is done because previous modelling studies have shown that allowing progression rates to increase with age and by male sex is necessary to account for observed trends in mortality[32,41] and previous statistical analyses also indicate the significance of sex and age (specifically age at liver tissue biopsy) on progression rates.[42]

The impact of sex on fibrosis progression rates (that male progression is faster than female) is incorporated by requiring that male progression rates are up to twice as fast as female, denoted by the relative risk $h_{sex}^{fibr.} \in [1,2]$.[42] The impact of age is incorporated by allowing the progression rates to vary for people between the ages of 20 and 70 years old; specifically, the 70-year-old progression rates can be equal to the 20-year-old progression rates (no difference by age), or up to ten times greater, denoted by the relative risk $h_{age}^{fibr.} \in [1,10]$.

The fibrosis progression rates are, therefore, specified according to three calibrated quantities, the relative progression rate by age ($h_{age}^{fibr.}$), the relative rate by sex ($h_{sex}^{fibr.}$) and the speed of progression ($\alpha^{fibr.}$), in addition to the meta-analysis-derived rates of fibrosis progression ($r^{k\to k+1}$):

$$\lambda_{ij}^{k\to k+1} \propto r^{k\to k+1} \times \begin{Bmatrix} 1 & j=f \\ h_{sex}^{fibr.} & j=m \end{Bmatrix} \times \begin{cases} 1 & i<20 \\ \dfrac{(h_{age}^{fibr.}-1)}{50}(i-20)+1 & 20\le i<70, \\ h_{age}^{fibr.} & i\ge 70 \end{cases}$$

where the constant of proportionality is

$$\frac{2}{1+h_{sex}^{fibr.}}\frac{5}{2h_{age}^{fibr.}+3}.$$

This function is illustrated in Figure S11.

End stage disease progression rates. The remaining natural history model progression rates are for the transitions to HCC ($\lambda_{ij}^{3\to HCC}$, $\lambda_{ij}^{4\to HCC}$, $\lambda_{ij}^{DC\to HCC}$) and the transition to DC ($\lambda_{ij}^{4\to DC}$). Values are taken from the literature, see Table S1. In general, annual transition probabilities (p) are published, which are transformed to exponential rates (λ) according to $\lambda = -\ln(1-p)$.

End stage disease progression rates are varied in calibration across a range of probabilities: where multiple sources of a parameter were available, these were used to inform the endpoints of uniform prior distributions for that parameter; where only one suitable value could be found, approximate 95% confidence intervals were constructed (assuming binomial distributions) and these were used as endpoints on the range of parameter values. The aim of this procedure was to be as conservative as possible. This was partly to reflect uncertainty even in countries where information was available regarding the relevant disease transition probabilities, but primarily this was to acknowledge lack of insight into many of these transition rates on a global scale.

The transition rate to HCC varies with age and sex,[3] and there is no prior reason to assume that the other end-stage disease progression rates would not vary with these cofactors as well. Accordingly, these progression rates are varied individually by age and sex in an analogous manner to the fibrosis progression rates. The following age and sex relative risk parameters are, therefore, included in the calibration procedure (with the same ranges as their fibrosis analogues): $h_{age}^{fibr.}$, $h_{age}^{j\to HCC}$, $h_{age}^{j\to DC}$, $h_{sex}^{fibr.}$, $h_{sex}^{j\to HCC}$, $h_{sex}^{j\to DC}$, where $j \to HCC$ indicates any transition to HCC.

Mortality rates. Increased mortality rates due to HCV infection are specified from compensated cirrhosis (μ_{ij}^{4}), DC (μ_{ijd}^{DC}) and HCC (μ_{ijd}^{HCC}). Values are taken from the literature according to duration of infection (less than, or greater than, one year), see Table S1. Mortality rates vary by age and sex[43,44] and this

is accounted for in calibration in the same way as for other parameters, see above. This results in calibrating the following parameters: $h_{age}^{cirr.\to\mu}$, $h_{sex}^{cirr.\to\mu}$, $h_{age}^{HCC\to\mu}$, $h_{sex}^{HCC\to\mu}$, where cirr. refers to both cirrhosis stages.

Transmission

The model incorporates two forces of infection (the per-capita rate at which those in the susceptible compartment become infected[45]): the one affecting the entire population is termed the general population (or GP) force of infection. Epidemiologically this can be considered to comprise (primarily) transfusion transmissible infections with infected blood and exposure to HCV through inadequate infection control.[6,7] These two routes are the dominant routes of infection but are not the only means of transmission. Other routes of infection include tattooing, body piercing, beauty treatments, non-injection drug use and high-risk sexual behaviours in HIV-positive MSM.[13,46–49] The relative contributions of the different transmission routes have rarely been investigated: one US study of blood donors suggested piercings and religious scarification were significant risk factors for HCV infection.[50] Other studies have led to opposite conclusions, suggesting at most a minor role for these transmission routes.[7] Transmission among HIV-positive MSM is an increasingly important transmission route in some settings,[51–53] but is at present a small part of the global HCV epidemic (see Martin et al.[54] and risk groups paragraph of the section **Error! Reference source not found.** for further discussion).

The PWID force of infection arises through the sharing of drug paraphernalia.[55] Both transmission routes can be considered to be driven through "engagement" with a particular activity, either injection drug use on the one hand or blood transfusion/medical procedures on the other.

The force of infection in a mass action model consists of the probability of transmission multiplied by the rate of infectious encounters.[56] In the model of HCV, susceptible individuals have a rate of engagement with a particular activity $c_{il}(t)$, assumed to vary by age i, calendar time t (see below) and risk group l. It is assumed that the rate of engagement with the activity is equal in all individuals (for a particular risk group), regardless of disease status. As such, the rate at which susceptible individuals (of age i and risk group l) are exposed to infection is equal to their engagement rate multiplied by the proportion of engagements that are with infectious individuals (the effective prevalence), denoted according to risk group as $p_l^{eff.}(t)$:

$$\text{rate of exposure}_{il} = c_{il}(t)p_l^{eff.}(t) = c_{il}(t)\frac{\sum_i c_{il}(t)\, I_{il}(t)}{\sum_i c_{il}(t)\, N_{il}(t)},$$

where $I_{il}(t)$ is the number of infectious individuals in age band i and risk group l, and $N_{il}(t)$ is the number of all individuals in age band i and risk group l (sum over all other indices and all relevant model compartments). Denoting the probability of transmission in an engagement between susceptible and infectious individuals as $p^{transmit}$, then the force of infection can be written:

$$\Lambda_{il}(t) = p^{transmit}c_{il}(t)p_l^{eff.}(t) = p^{transmit}c_{il}(t)\frac{\sum_i c_{il}(t)\, I_{il}(t)}{\sum_i c_{il}(t)\, N_{il}(t)}.$$

The quantity $p^{transmit}c_{il}(t)$ is the transmission risk $\beta_{il}(t)$ for a particular age and risk group. Since the probability of transmission is not assumed to vary by age, we can multiply inside the sum by the transmission probability in the numerator and denominator to rewrite the above expression as:

$$\Lambda_{il}(t) = \beta_{il}(t)\frac{\sum_i \beta_{il}(t)\, I_{il}(t)}{\sum_i \beta_{il}(t)\, N_{il}(t)}.$$

General population force of infection. Above it was assumed that transmission risk varied by age and through time. In the case of the general population risk, this is known to be true, since age-specific prevalence varies by country, indicating different levels of exposure at different ages across time.[57] Mechanistically, the variation of risk by age may be considered a result of different engagements with healthcare over the course of a lifetime; the variation over time may be a consequence of changes in infection control and blood screening measures.[57] The general population force of infection can be written as:

$$\Lambda_{i,GP}(t) = \beta_{i,GP}(t)\, p_{all}^{eff}(t),$$

where $p_{all}^{eff.}(t) = \sum_i \beta_{i,GP}(t)\, I_i(t) / \sum_i \beta_{i,GP}(t)\, N_i(t)$ is the effective prevalence among all people, $I_i(t)$ is the number of all infectious people of a given age and $N_i(t)$ is the number of all people of a particular age (implicit sum over all risk groups and other indices, for all relevant compartments). The risk of transmission is decomposed into age and time dependent terms:

$$\beta_{i,GP}(t) = \gamma_i \beta_{GP}(t).$$

These quantities are fit in calibration (see Reinfection**.** Work in the PEG-IFN+RBV era suggested that reinfection occurs at an equal rate to primary infection.[69,70] Other work in this period indicated that reinfection may be less likely to result in viraemic infection.[71] More recent articles after the development of DAAs

illustrate that PWID who have been treated and relapsed to injecting behaviour are often reinfected, though in these studies comparisons with non-SVR individuals were not given preventing a formal assessment of the relative risks of reinfection versus primary infection following cure.[72,73] Searching for information regarding potentially lower rates of reinfection among PWID, as suggested by this press release reporting on a 2017 presentation,[74] did not return robust evidence demonstrating such an effect. With such a range of views regarding relative risk of reinfection, and no clear evidence one way or the other, a conservative assumption was adopted, viz. that reinfection risk is equal to primary infection risk (a relative risk of one), an assumption followed by other modelling analyses for largely the same reasons.[75,76]

Calibration section). γ_i, the age-dependent general population risk multiplier (constant in time) is constructed as an interpolated spline with knot values at ages 33, 66 and 100 years old (ξ_{33}^{GP}, ξ_{66}^{GP}, ξ_{100}^{GP}), with uniform prior distributions specified for each of these knots.

$\beta_{GP}(t)$, the time-varying contribution to the general population risk of infection, is constructed as an interpolated spline with knots at years 1930, 1950, 1970, 1990 and 2005 (corresponding to the values: ξ_{1930}^{GP}, ξ_{1950}^{GP}, ξ_{1970}^{GP}, ξ_{1990}^{GP}, ξ_{2005}^{GP}). Before 1930, access to healthcare was limited and it is assumed that the spread of disease through medical procedures was minimal. Countries that experienced early HCV epidemics did so from the 1930s onwards.[58] The prior distribution on the 1930 knot (ξ_{1930}^{GP}) is, therefore, a sharply decreasing exponential distribution (rate = 1·00) implying low transmission before this time. The prior distributions on the knots for 1950, 1970 and 1990 (ξ_{1950}^{GP}, ξ_{1970}^{GP}, ξ_{1990}^{GP}) are chosen to be as uninformative as possible without permitting impossibly high rates (this was found to be an exponential prior with rate = 0·05). In the period before 1990, universal health care expanded but application of infection control and screening of blood transfusions were less than adequate, hence the decision not to constrain the rate of infection in the general population over this period.

After 1990, countries began implementing blood transfusion screening. In addition, infection control continued to improve with technological and educational advances. It is a reasonable assumption, therefore, that general population risk of infection decreased after 1990. However, the roll-out of blood safety programmes and the magnitude of improvements in infection control were not uniform at the global scale. As a proxy for the improvement in iatrogenic procedure safety after 1990, the average per capita health spending was calculated for all the countries in the model[59] and the top quartile were assumed to have significantly reduced general population incidence. This was achieved by setting the 2005 general population risk of infection proportional to the 1990 value, $\xi_{2005}^{GP} = q^{country}\xi_{1990}^{GP}$, but drawing the multiplicative factor $q^{country}$ from a high rate exponential distribution (rate = 50). Conversely, for countries in the bottom three quartiles, it is still assumed that some improvements have been made in terms of general population transmission, however, the prior assumption about the scale of improvement is weaker (specifically $q^{country}$ is drawn from a lower rate exponential distribution, with rate = 5). The general population risk of infection is held constant at this reduced rate at all later times; we do not model any further improvements in infection control in the status quo scenario.[60]

PWID force of infection. The PWID force of infection is assumed to vary over time. This encapsulates the known changes of injection drug use within the injection drug population. While PWID, an individual's risk of infection is assumed to be elevated for the duration of injecting career, but not to vary with age. The PWID force of infection is, therefore, the sum of the general population risk (through engagements with healthcare as per the non-PWID population) and the age-constant increased risk of infection due to injection drug use:

$$\Lambda_{i,PWID}(t) = \beta_{i,GP}(t)\, p_{all}^{eff.}(t) + \beta_{PWID}(t)\, p_{PWID}(t).$$

The final term incorporates the prevalence (not effective prevalence) because of the simplifying assumption that riskiness of behaviour does not vary with age over the course of an injecting career ($c_{i,PWID}(t) \equiv c_{PWID}(t)$).

The time dependent risk of transmission in PWID, $\beta_{PWID}(t)$, is composed of interpolated splines with knots at 1950 and 1980 (values ξ_{1950}^{PWID} and ξ_{1980}^{PWID}). The value of the knot at 1950 is drawn from a sharply peaked exponential prior, reflecting the low levels of injection drug use before 1980 (exponential rate = 1) and the knot at 1980 is chosen to be as uninformative as possible (exponential rate = 0·05).

The impact of recent expansion of PWID harm reduction services is taken into account as follows. It has been suggested that needle and syringe programmes (NSP) and opioid substitution therapy (OST), when implemented individually, do not significantly reduce the risk of HCV infection among PWID, but can reduce transmission by a factor of 0·75 when implemented in combination.[61,62] Furthermore, it is recognised that "the higher infectivity of HCV compared to HIV and greater prevalence demands ... higher coverage and greater scale-up" [63] of harm reduction interventions to reduce HCV transmission among PWID than required

to reduce HIV transmission (see also Grebely & Dore (2011)[64] and Gore & Bird (1998)[65]). This is primarily because there is much higher prevalence of HCV than HIV in this group.[20] To capture both of these effects, we require a country not only to have implemented both OST and NSP programmes, but require NSP coverage to be high in order for there to be a harm reduction effect. High coverage of NSP is defined following WHO targets as at least 200 syringes distributed per PWID per year (indicator NSP.C.1c[66]). Provided this coverage of NSP is reached, coverage of effective harm reduction is then defined as the percentage of opioid-dependent PWID reached by OST programmes. This subset of PWID are, therefore, in receipt of both OST and high-coverage NSP (due to the high coverage NSP requirement) and so can be considered to experience a reduced risk of HCV transmission.[61] In 2010, only three countries (Norway, Australia and Moldova) reported high coverage of NSP services, rising to nine in 2017;[67,68] as global coverage is so low in 2010, we model no coverage before 2010, rising to coverage in nine countries in 2015 (the year in which those countries reported). Details on the proportion of the PWID population opioid-dependent (and so eligible for OST) and the proportion of this group receiving OST in 2015 is described in Model inputs.

Reinfection. Work in the PEG-IFN+RBV era suggested that reinfection occurs at an equal rate to primary infection.[69,70] Other work in this period indicated that reinfection may be less likely to result in viraemic infection.[71] More recent articles after the development of DAAs illustrate that PWID who have been treated and relapsed to injecting behaviour are often reinfected, though in these studies comparisons with non-SVR individuals were not given preventing a formal assessment of the relative risks of reinfection versus primary infection following cure.[72,73] Searching for information regarding potentially lower rates of reinfection among PWID, as suggested by this press release reporting on a 2017 presentation,[74] did not return robust evidence demonstrating such an effect. With such a range of views regarding relative risk of reinfection, and no clear evidence one way or the other, a conservative assumption was adopted, viz. that reinfection risk is equal to primary infection risk (a relative risk of one), an assumption followed by other modelling analyses for largely the same reasons.[75,76]

Calibration

The model is calibrated to three sets of information: HCV viraemic prevalence estimates in the overall population, data on prevalence of viraemic HCV infection in the PWID population and estimates of mortality due to HCV-attributable cirrhosis/decompensated cirrhosis (termed a cirrhosis death) or HCV-attributable HCC (an HCC death). While HCV incidence data are available in select instances, they have not been systematically combined to produce a reliable dataset that could be utilised within a global model; as such the model is not fit to incidence estimates and instead relies in calibration on the relatively stronger mortality and prevalence information available. References to prevalence in the following refer only to viraemic prevalence. The data are described in detail in Model inputs, see below. This section describes how the likelihood functions are constructed and how we sample from the parameter posterior distributions.

According to Bayes' theorem:

$$p(\phi|\mathcal{D}) = \frac{p(\phi)\mathcal{L}(\phi|\mathcal{D})}{\int p(\phi)\mathcal{L}(\phi|\mathcal{D})d\phi},$$

where $p(\phi|\mathcal{D})$ is the posterior distribution of the set of parameters ϕ conditional on data $\mathcal{D}$, $p(\phi)$ is the prior distribution of the set of parameters, $\mathcal{L}(\phi|\mathcal{D})$ is the likelihood of the parameters conditional upon the data, and the denominator is a normalisation constant that does not need to be explicitly evaluated.[77] The prior distributions are defined using ranges of values and distributions from the literature, see Table S2. The likelihood function is equivalent to the probability of observing the data conditional on a particular set of model parameters, ϕ: $\mathcal{L}(\phi|\mathcal{D}) \equiv p(\mathcal{D}|\phi)$[78]. Once evaluated, samples are drawn from the posterior distribution using Incremental Mixture Importance Sampling (IMIS), described in Raftery and Bao (2010)[79] and below.

Calculating the likelihood. There are three contributions to the value of the likelihood: overall viraemic HCV prevalence, PWID HCV viraemic prevalence and mortality: $\mathcal{L} = \mathcal{L}^{overall\,prev.} \times \mathcal{L}^{PWID\,prev.} \times \mathcal{L}^{mortality}$. The calculation of the likelihood in respect of each set of information is as follows.

Overall viraemic prevalence values are 2015 country-by-country estimates, used by the WHO in formulating their global targets (see Model inputs). The likelihood in respect of the overall viraemic prevalence is assumed to follow a beta distribution, Beta(α, β), defined between the upper ($p_{high}^{overall}$) and lower ($p_{low}^{overall}$) uncertainty intervals (the likelihood is assumed zero outside this range). The parameters are chosen to ensure the mode is located at the central estimate of prevalence $p_{central}^{overall}$, $\beta = (\alpha - 1 - p_{central}^{overall}(\alpha - 2))/p_{central}^{overall}$, and α is chosen such that 95% of the probability density function lies within 20% of the central prevalence estimate (the exact value of α varies by country). This value was chosen to produce simulated median global prevalence values that were in accord with WHO estimates (since it is WHO

elimination targets that were being investigated by our model). The likelihood of a particular parameter set ϕ is calculated from the modelled prevalence, $p^{overall}(\phi)$, by computing the value of the probability density function of the above-defined beta distribution at $p^{overall}(\phi)$:

$$\mathcal{L}^{overall\ prev.} = \begin{cases} \text{Beta}(p^{overall}(\phi);\ \alpha,\beta) & p_{low}^{overall} \le p^{overall}(\phi) \le p_{high}^{overall} \\ 0 & \text{otherwise} \end{cases}.$$

Where overall prevalence values are lacking, we make no assumption regarding overall prevalence other than requiring that the modelled prevalence is not less than the lower estimate for regional average prevalence (see Model inputs for information sources).

The likelihood in respect of the PWID viraemic prevalence data is calculated as follows. Where data are available, they comprise lower, middle and upper values. Such data are compiled from minimum and maximum prevalence values found in systematic reviews (see Model inputs). The constituent studies in the ranges of values might be conducted at different locations within a particular country, at different times or with limited subgroups within the population. To avoid applying unjustified statistical assumptions to these heterogeneous data (which nevertheless represents the best resource for modelling the global HCV epidemic in PWID), we constructed a triangular likelihood, such that simulated values must lie between the minimum and maximum values and are maximised at the central value:

$$\mathcal{L}^{PWID\ prev.} = \begin{cases} \dfrac{C(p^{PWID}(\phi) - p_{low}^{PWID})}{p_{central}^{PWID} - p_{lower}^{PWID}} & p_{low}^{PWID} \le p^{PWID}(\phi) \le p_{central}^{PWID} \\ \dfrac{C\left(p_{upper}^{PWID} - p^{PWID}(\phi)\right)}{p_{upper}^{PWID} - p_{central}^{PWID}} & p_{central}^{PWID} \le p^{PWID}(\phi) \le p_{upper}^{PWID} \\ 0 & \text{otherwise} \end{cases},$$

where $C = 2/(p_{upper}^{PWID} - p_{low}^{PWID})$ is a normalisation constant and $p^{PWID}(\phi)$ is the modelled prevalence. Where PWID prevalence values are lacking, no contribution to the likelihood in respect of PWID prevalence data is added.

Mortality information consists of estimates of numbers of deaths by year y, age-band a, sex j and death type t (cirrhosis or HCC), $M_{a,j,y,t}$. 95% uncertainty intervals are provided in the mortality estimates. The number of deaths is assumed to follow a log-normal distribution,[80] with mean located at $\ln(M_{a,j,y,t})$ and variance denoted $\sigma_{a,j,y,t}^2$: $\text{Lognormal}(\ln(M_{a,j,y,t}), \sigma_{a,j,y,t}^2)$. The variance is chosen such that the cumulative distribution function is equal to 0·975 at the upper mortality estimate.

The contribution to the likelihood in respect of a particular mortality estimate is the value of the log-normal probability distribution function at the modelled value of mortality (in the particular age-band, sex, year and death-type), for parameter set ϕ, $M_{a,j,y,t}(\phi)$:

$$\mathcal{L}_{a,j,y,t}^{mortality} = \frac{1}{M_{a,j,y,t}(\phi)} \frac{1}{\sqrt{2\pi\sigma_{a,j,y,t}^2}} \exp\left(-\frac{\left(\ln(M_{a,j,y,t}) - \ln\left(M_{a,j,y,t}(\phi)\right)\right)^2}{2\sigma_{a,j,y,t}^2}\right),$$

All such points can be combined to produce the total contribution to the likelihood in respect of the mortality information:

$$\mathcal{L}^{mortality} = \prod_{a,j,y,t} \mathcal{L}_{a,j,y,t}^{mortality}.$$

The overall likelihood is then the product of the three likelihoods defined above. To reduce numerical overflow the quantity actually calculated is the log-likelihood, $l^{mortality} = \sum_{a,j,y,t} \ln \mathcal{L}_{a,j,y,t}^{mortality}$ and similarly for the other terms, giving the final expression for the log-likelihood:

$$l(\phi|\mathcal{D}) = \frac{1}{24} l^{mortality}(\phi|\mathcal{D}) + l^{PWID\ prev.}(\phi|\mathcal{D}) + l^{overall\ prev.}(\phi|\mathcal{D}),$$

where the numerical factor reduces the weight of the mortality information (for which each country has 96 values) relative to the prevalence values in order to improve the model fit. A second adjustment to the likelihood is the requirement that incidence not be increasing after 2015; this criterion was implemented to prevent runaway simulations occurring, whereby an acceptable calibration was achieved, but the choice of risk values was such that very soon after 2015 an epidemic explosion occurred. As found in model development, such simulations were very uncommon, contributing to raising the upper 95% uncertainty interval but not altering the posterior median in a noticeable way compared to simulations in which incidence was not allowed to take off after 2015 (results not shown).

Sampling from the posterior distributions. The model is calibrated using Incremental Mixture Importance Sampling (IMIS), since it was designed to fit country-level HIV models and is known to be robust even in situations with multimodal posterior distributions.[79] Implementation in C++ was written for a previously published model[81] and was adapted for use here. The fitting approach is summarised briefly.

N_0 = 1 million initial samples are drawn from the prior distributions. The model is run and the likelihood calculated. The weight is calculated for all simulations (the ratio of individual likelihood to the sum of all likelihoods). The maximum weight point is chosen and a multivariate Gaussian distribution is constructed around this point from which B = 10 000 new parameter sets are sampled (the covariance of the multivariate Gaussian distribution is calculated using the B nearest points according to their Mahalanobis distance[82] from the maximum weight parameter). The likelihood of the newly sampled points is calculated. The weights of all points sampled are recalculated according to the prior distribution and the sum of all previously constructed multivariate Gaussian distributions. This defines the mixture sampling distribution. The maximum weight point is chosen, Gaussian distributions constructed and B new parameter sets are sampled and so on.

This process is continued until a stopping criterion is reached, specifically that all points have equal importance weights. At a given iteration of the model, this is estimated by calculating the expected number of unique points that would result were resampling (from a multinomial distribution defined by all the parameter sets sampled so far) to take place at that point. To assess whether the stopping criterion has been reached it is noted that, given a set of parameters labelled i with weight w_i, the probability of resampling parameter set i from a J-dimensional multinomial distribution is $1-(1-w_i)^J$, where $J=1000$ is the aimed-for number of posterior samples. During a particular iteration of the model fit, therefore, the expected fraction of unique parameter sets sampled is calculated as $E=\sum_i(1-(1-w_i)^J)/J$. This quantity is compared to the case where all points have equal weight, i.e. $w_i=1/J$, in which case it can be shown that the expected fraction of unique points is $1-1/e\approx 0{\cdot}63$. In other words, the algorithm terminates once $E>$ $0{\cdot}63$ and resamples (with replacement) from all the parameter sets according to their associated importance weights. Due to this self-monitoring nature, there is no stopping criterion to report, while convergence is reached by definition in this process.

Modelling treatment and prevention

Treatment regimens. Two treatment regimens are implemented: pegylated interferon plus ribavirin (PEG-IFN+RBV) and direct-acting antivirals (DAAs). Rates of sustained virologic response (SVR) when treated with PEG-IFN+RBV vary by genotype: genotype 1 - 44%, genotype 2 and 3 - 73% and genotype 4 - 53%.[83] Genotypes 5 and 6 are less well studied. The SVR rate in genotype 5 is believed to be similar to genotypes 2 and 3 - 73%.[84] SVR in genotype 6 is estimated to be 75%.[85] Treatment durations are 48 weeks for genotype 1,[86] 24 weeks for genotypes 2 and 3,[86] 48 weeks for genotype 4,[87] 24 weeks for genotype 5, based on similarity to genotypes 2 and 3,[84] and 48 weeks for genotype 6, as a conservative assumption.[84,87] Note we do not simulate treatment from either DC or HCC: PEG-IFN+RBV is contraindicated in patients with DC[88] and the impact of SVR on patients with HCC is contested therefore, to be conservative, we assume no impact on survival so do not model HCC antiviral treatment.[89]

Many DAA combinations are available. Instead of modelling a specific combination, we make the assumption that the best DAAs for the particular genotype, disease stage and treatment experience history are used in individual cases, leading to the best possible outcomes for the patient. Given the assumption of optimal regimens, recent work has shown equivalently high values of SVR in compensated cirrhotic and pre-cirrhotic patients, regardless of genotype, treatment experience or age.[90–93] Accordingly, we adopt the value 98% for the proportion achieving SVR in all patients in stages F^0 to F^4, regardless of genotype. In addition, studies have shown equivalent rates of SVR in active drug use patients[94,95] and we assume that PWID receiving DAAs have the same outcomes as the general population. Lastly we do not explicitly model HIV coinfection since coinfected patients receiving ART have equivalent SVR to mono-infected patients;[96–98] assuming high ART coverage, we are justified in not modelling differential treatment efficacy in HIV coinfected patients. DAAs can be used to treat patients in DC with 85% achieving SVR.[93] We do not model DAA treatment for patients in the HCC compartment as described above.[89]

Duration of treatment is assumed to be 12 weeks up to and including compensated cirrhosis, and to be 24 weeks in DC.[90,93] Treatment duration is uniform by genotype.[99] Options for even shorter duration of treatment, for example through detection of a sufficiently low viral load,[100,101] are not modelled as the requisite nucleic acid tests would add to cost and would not be available for a large proportion of the global HCV infected population.

Once SVR has been achieved, disease progression and mortality rates are reduced or set equal to zero. Studies have suggested fibrosis regression can take place if SVR occurs before cirrhosis, however, as a conservative measure we set disease progression rates to zero if SVR is achieved before compensated cirrhosis,[102,103] but do not model regression. If SVR is achieved in compensated cirrhosis, progression rates to DC and HCC are reduced and direct mortality is eliminated.[104,105] Those who have achieved SVR and are in the DC compartment have no decrease in mortality but have a lower incidence of HCC.[106] Direct treatment of HCC is not possible; however, the impact of SVR on survival in HCC must be taken into account since those who have already achieved SVR can progress to HCC (though at a reduced rate). A reduced rate of mortality for those with SVR in HCC is modelled, following.[107]

Historical diagnosis and treatment. The model uses estimates of the proportion of HCV infected people diagnosed and the proportion of those diagnosed who are treated to simulate historical standards of care. 2015 values are available for select countries[41,108–110] and regional averages[111] used to impute the remainder (see Model inputs below for more details). Additionally information on DAA use in 2016 is added into the model for select countries, see below. It should be noted that the proportion diagnosed is defined in WHO reports as the "number of persons with chronic infection diagnosed out of the total number of persons with chronic infection".[25,111] This definition is used when scaling up diagnosis to meet historical estimates of this quantity. This approach means that individuals who are cured are not included in calculations of the proportion diagnosed. At the current modest levels of cure, this definition is stable (since the number who are cured following diagnosis is small). In interventions simulated here, particularly the outreach screening intervention (intervention IV) this definition becomes unusable because, in aiming to reach up to 90% diagnosis proportions, the proportion diagnosed never asymptotes to a stable quantity. For this reason, an alternative definition of the proportion diagnosed is proposed, whereby cured (but not reinfected) individuals are considered as diagnosed as well, allowing targeted diagnosis proportions to be reached. In terms of the model quantities, therefore, the proportion diagnosed can be written as

$$\frac{\sum(D+T+Q+C)}{\sum(A+U+D+T+Q+C)}$$

where the summations are over all indices of the given compartment. The definition of the proportion treated is retained: "number of persons initiating treatment during a given year out of the total number diagnosed".[25,111]

Diagnosis is started in 1990, approximately the time the first HCV specific antibody assays were introduced.[112,113] The rate of diagnosis is chosen such that the proportion diagnosed increases linearly from 0% in 1990 to the country value (or regional average value where data are lacking) in 2015. Care seeking behaviour is assumed to vary with disease stage: those with stage equal to or less than F^3 have a relative rate of seeking diagnosis of 0·0025, compared to rates of 0·1 for compensated cirrhosis, and 0·4 in DC or HCC compartments. This captures the trend that individuals present late for treatment, usually only after the onset of symptoms (taken here to occur from the onset of compensated cirrhosis). Unless altered in intervention, the diagnosis rate reached in 2015 is kept constant, modelling constant care seeking behaviour in the population. The bias towards end-stage diagnosis is also retained unless altered through outreach screening programmes which facilitate people entering care regardless of disease stage.

Historical PEG-IFN+RBV treatment is simulated as follows: after the introduction of PEG-IFN+RBV as the standard treatment for HCV infection,[86,114] the rate of treatment is chosen such that the annual proportion of those diagnosed who are treated rises linearly from 0% in the initial year of treatment, to the country (or regional) estimate of the fraction treated in 2015. The initial year of treatment is chosen, region-by-region, such that the cumulative number of treatments is approximately equal to the cumulative number of treated patients by region.[111]

DAAs are implemented from 2016 in countries in which their use is reported. In these countries, the rate of treatment is calculated such that the reported number of DAA treatment courses is matched by the model.[115,116] All other countries continue to implement PEG-IFN+RBV after 2015. This approach results in 1·76 million HCV treatment courses being delivered in 2016, of which 86% consist of DAAs.[116] The rate reached in 2015 or 2016 (depending on whether DAA information is available for a given country) is kept constant unless altered in intervention as described in the following section. As in the case of diagnosis, this simulates a constant treatment seeking behaviour among those diagnosed.

A list of all countries with estimates for the percentage diagnosed and treated, or the regional estimates where data are lacking, is given in Table S3; values for the additional DAA treatment courses given are reported in Table S4.

Retreatment. Previous models have not allowed retreatment after reinfection (following SVR).[117–119] In the age of well-tolerated DAAs this is not considered a necessary or equitable restriction.[120] However, to

prevent the possible (presumed unlikely) cycling on and off treatment (due to rapid retreatment following cure), an average delay of five years before potential retreatment was implemented in the model.

Historical harm reduction. Prior to 2016, blood and injection safety improvements are modelled in countries with high health expenditure (see Transmission and Model inputs for details). Reduced transmission among opioid-dependent PWID is simulated in countries with high coverage NSP programmes plus OST programmes, with coverage equal to OST programme coverage (see Transmission and Model inputs for details).

Intervention strategies

Four intervention strategies are simulated from 2017 above a status quo scenario (described below). We also simulated a scenario in which DAAs were not rolled out from 2016 in any countries, called the no-DAA scenario. Interventions simulated atop the status quo are cumulative: each incorporates the features of the previous interventions. If not otherwise stated, parameter values not changed in an intervention are equivalent to their values in the previous scenario. An overview of the parameter changes scenario-by-scenario is given in the table in the main text.

No DAA scenario. DAAs are implemented in our status quo scenario, but we simulated a scenario in which they are not implemented at all and all treatment courses in the past and future are PEG-IFN+RBV. This was included to investigate the benefit of DAA use even at current rates of diagnosis and treatment and provides an estimate of the future HCV epidemic were the recent rapid scale up of DAA use not to be continued.

Status quo. In the model, DAAs are introduced in 2016 in those countries where use has been reported: this results in 86% of 1·76 million treatment courses in that year consisting of DAAs instead of PEG-IFN+RBV,[116] see Modelling treatment and prevention section above for details. In the status quo, it is assumed that all these countries in which DAAs are implemented continue to use DAAs, and diagnosis and treatment rates are fixed at 2016 values. All other countries continue to use PEG-IFN+RBV at rates set to match 2015 proportion treated of those diagnosed as described in the previous section. Risk of transmission in the general population and PWID is kept fixed at 2015 values and no other changes are made. This scenario represents our best estimate of what the HCV epidemic will look like with no changes made to improve rates of diagnosis and treatment or to reduce numbers of HCV infections.

Intervention I: + blood safety and infection control. Two of the key transmission routes in the general population are blood transfusions and unsafe injections.[6] Accordingly, blood and injection safety have been targeted for elimination or reduction by WHO: by 2020 the aim is for 95% of blood donations to be screened in a quality assured manner and by 2030 for 100% of donations to be screened.[111] At the same milestones, it is intended that 50% and 90% of injections respectively are given "with safety-engineered devices in and out of health facilities";[121] the most recent report goes further and aims for a 0% "proportion of unsafe injections" by 2030.[111] Published estimates of the source of infections in the general population suggests uncertainty regarding the precise transmission route of a significant fraction of infections in the general population.[111] Accordingly, focusing only on blood and injection safety targets alone may not eliminate HCV infection. Yet such infections have been all but eliminated in high income countries through rigorous infection control in all settings. This demonstrates that overall improvements in health systems, as well as targeted interventions regarding blood and injection safety, can prevent the majority of infections resulting from medical and non-medical procedures.

These considerations make the case for simulating significant reductions in general population risk by 2030 as a plausible public health target, through improvements in infection control across all settings. By contrast, the existence of HIV-positive MSM in particular as an increasingly significant at-risk group, as well as the clear challenges in eliminating medical-procedure related infection on a global scale, lead us to propose a more conservative 80% reduction in general population risk globally from 2015 values by 2020 (reached by linear scale up). As an assumption, this value is varied in our analysis (between the values of 0% and 95%), see main text figure 5A and Figure S4A.

Intervention II: + PWID harm reduction. OST in combination with high coverage NSP has the most consistent evidence of reducing HCV incidence in PWID: meta-analyses suggest that such programmes could reduce the risk of infection by 75%[61] or 71-74% (two analyses are performed that arrive at relative risks of 0·26 and 0·29 respectively based on whether adjusted or unadjusted outcomes were available).[62] The latter study analyses data from 3241-3356 participants and no one study has a weight in the meta-analysis of more than 40%, suggesting that risk reductions of approximately 75% are appropriate values to incorporate when considering high quality OST+NSP programmes.

Our intervention aims to increase all countries to high coverage NSP (defined as at least 200 syringes distributed per PWID per year), and expand OST provision to 40% of the opioid-dependent PWID

population, using both of the WHO high targets for intervention coverage.[66] Countries with higher initial coverage are assumed to remain at those values (see Model inputs for details on the calculation of historical OST coverage). As such, 40% (or higher where appropriate) of the opioid-dependent PWID population are covered by an intervention that reduces the risk of HCV infection: $\beta_{PWID}(t)$ is reduced by 75% for 40% of the opioid-dependent PWID population. Reducing risk of transmission according to risk reductions reported in meta-analyses has been used in other studies to explore the impacts of harm reduction interventions in PWID, see for example Martin et al. (2013).[9]

The OST coverage parameter and, therefore, the coverage of effective harm reduction, is varied in our analysis between the status quo value of less than 1% globally[68] and 95%, see main text figure 5B. Following recent commentary on the challenge of reducing HCV transmission among PWID, no impact of expanded NSP or OST in isolation is simulated.[61,63,117]

Intervention III: + offer DAAs at diagnosis. In this scenario, we assume that all countries introduce DAAs from 2017 so no PEG-IFN+RBV is used anywhere. Additionally, because patients engage with the healthcare system in order to be diagnosed, a logistically achievable approach to reducing the burden of HCV would be to offer DAA treatment at the time of diagnosis regardless of disease stage. In this scenario, those recently diagnosed are offered treatment and 90% accept and adhere to treatment. The rate of seeking treatment in the remainder of the population is maintained at the 2015/2016 values as described above.

Intervention IV: + outreach screening. In order to fully realise the potential of DAA therapies, diagnosis efforts must be stepped up in order to minimise the number of people unaware of their status. The model simulates scaling up rates of diagnosis through a countrywide programme of outreach screening such that, by 2030, 90% of the infected population is diagnosed.[111] This value is reached through linear scale up from the initial number diagnosed. As in intervention III, people are offered treatment upon diagnosis (with 90% accepting and adhering to treatment). In addition such an expansive outreach screening strategy would result in those previously diagnosed but untreated returning back into the care cascade to receive the new DAAs. This is simulated as 10% per year of the previously diagnosed population returning for treatment.

<u>One-way sensitivity analyses</u>

One-way sensitivity analyses were performed on a number of parameters that were fixed in the primary analysis and are described here. These were either parameters affected by the nature of the intervention programme (effectiveness of PWID intervention) or else parameters about which the evidence for their value was mixed or limited (relative rates of reinfection, delay in retreatment following reinfection). In the primary analysis best estimates were used for these values or where this was lacking an approach was taken with the aim of being conservative. To make it computationally feasible to perform several sensitivity analyses, these simulations were performed with a random sample of 100 posteriors (as opposed to 1000 used in the primary analyses). Experience working with the model has shown that this has minimal impact on outcomes. The discussion here is intended to augment conclusions drawn in the main text.

Effectiveness of PWID harm reduction interventions. Evidence for the impact of OST+NSP (the harm reduction intervention simulated in this analysis) is of uncertain quality.[61,62] This is reflected in the wide confidence intervals for the risk reduction of PWID on OST+NSP programmes reported in these papers; taking the end-points of these values suggests a range of plausible effectiveness values from a minimum impact of a 20% reduction to a maximum impact of a 90% reduction (the primary analysis utilises a 75% reduction). Figure 3 from the main text has been reproduced using the lower value of effectiveness to illustrate how the outcomes change with this large reduction in impact of PWID harm reduction programmes, see Figure S8. The impact on key outcomes by varying the effectiveness from the minimum to maximum values is shown in Table S5.

Delay in retreatment after reinfection. To test sensitivity to the assumption of a five year average delay before possible retreatment, the model was rerun with a one-year delay implemented instead. We considered only intervention IV (the comprehensive strategy) since this involves the most treatments being delivered and so a choice regarding treatment delay would make the most difference in this setting. Reducing the delay to one year does not alter the global incidence or mortality elimination years (still 2030 and 2032 respectively), while by 2050 the difference in incidence rate between the 5 year delay and 1 year delay simulations is only 3·5%, i.e. there is a very small increase in incidence with the smaller treatment delay time due to the greater rate at which people are offered retreatment and so subsequently available to be reinfected. This effect only marginally increases the number of treatments: by 2050 there are an additional 200 000 treatments delivered on a total number of 64 million courses. In other words, a possible cycling effect does not manifest noticeably in the results and the choice of delay has a minimal impact on outcomes.

Relative risk of reinfection. The impact of altering our assumptions regarding the relative risk of reinfection was investigated by running the model with relative risks of 0·2 and 0·5 (compared to a primary

analysis value of 1). Additionally, a sensitivity analysis was performed in which the comprehensive intervention strategy was simulated but with varying diagnosis coverages reached in 2030. This analysis was repeated for the three values of relative risk of reinfection (0·2, 0·5 and 1), see Figure S9.

Model inputs

Model inputs are taken from a variety of sources discussed below. The key factor determining the choice of sources was the requirement, as this is a global model, to find comprehensive sources that provided information (data or best estimates where necessary) for as many countries as possible. Given these sources, all countries are simulated in the same way for consistency in producing global results.

Demographic inputs. UN population prospects estimates (2017 update) are used to simulate the population, country-by-country, from 1950-2100.[19] The following information was used: population size in five-year age bands by sex, age-specific fertility rates in five-year age bands (between the ages of 15 and 50 years old), sex ratio at birth, migration rates and mortality rates in the ranges 0-1, 1-5, 5-10 years old and so on (calculated from life tables). Population sizes were available annually; all other values were available as five-year averages.

PWID inputs. Values of the proportion of the population PWID (denoted the proportion PWID) in the 15- to 64-year-old population were from a recent review by Degenhardt et al.[20]. We recalculated regional averages (see the following) in order to be consistent with the population data we used, following the description in the aforementioned paper and previous iterations of the work.[8,122] Countries with data comprise 81·1% of the global adult population. To extrapolate these country-level values to estimate the proportion PWID in the remaining countries, the procedure for producing regional and global PWID proportion estimates described in detail in Mathers et al.[8] and related papers is followed. Countries are classified by region (Eastern Europe, Western Europe, East and southeast Asia, South Asia, Central Asia, Caribbean, Latin America, Canada and USA, Pacific Island states and territories, Australia and New Zealand, Middle East and north Africa and Sub-Saharan Africa). Within each region an average proportion PWID is calculated, weighted by the 15- to 64-year-old population size. This value is used for countries in that region without data. One exception is made for Poland. This country lacks data, but applying the proportion PWID calculated in the above way results in too many HCV-positive PWID relative to the estimates of HCV-positive individuals in the entire population. The regional average is dominated by data from Russia. To be able to calibrate Poland we removed Russia and recalculated the regional average to produce a revised estimate of the proportion PWID. This results in a value (0·37%) closer to countries with similar drug use profiles as noted by examining data reported by the European Monitoring Centre for Drugs and Addiction.[123] In the Pacific Islands there are no data, so global averages are constructed in the same way as regional averages and these values are used for all the countries in the region. In the Caribbean, where only Puerto Rico has data, this value is combined with the global average for the remaining countries in the region to produce an adjusted regional estimate. Estimates derived using our population data are only marginally different from the ones reported in the original paper,[20] but we retain our recalculated values for consistency.

The proportion of PWID who are female and the proportion of PWID who are opioid-dependent (required for modelling the impact of PWID harm reduction interventions) are reported in the same paper and we recalculate the regional and global averages according to the same procedure described above.[20]

Harm reduction inputs. Countries with OST programmes, and in which at least 200 needles and syringes per PWID per year were distributed, are simulated as having a reduction in HCV transmission risk in opioid-dependent PWID. Larney et al. report the number of needle-syringes distributed in a given year (primarily 2014, 2015 and 2016) for 158 territories.[68] Using up-to-date population estimates we followed their procedure in calculating estimates of numbers of needle-syringes per PWID and also recalculated the regional averages to account for our use of 2017 population prospects population estimates (see above). This provided a binary measure of whether a country had greater than, or less than, 200 needle-syringes per PWID per year by 2015 (the year in which most data are available).

To construct OST coverage, the number of OST recipients by country was found for 72 countries, as reported in Larney et al.[68]. This number includes those on substitution therapy who are both primary injection drug users and those who are not (a group which includes sniffing and smoking as primary routes of administration[123]). To derive an approximate coverage of OST requires estimating the number of opioid users in the population (of whom a subset are opioid-dependent PWID); the number on OST at a particular time can then be divided by this value to arrive at a coverage in opioid-dependent people (indicator OST.C.1c in the WHO technical guide[66]). We then assume equal coverage in opioid-dependent PWID (indicator OST.C.1d[66]).

To estimate the number of opioid users by country, data were extracted from the UNODC website on prevalence of opioid and opiate use. Opiates are drugs derived from opium while opioids include these as

well as synthetic drugs created to emulate the properties of opiates. As such, opioid use prevalence is higher than opiate use. Noting that many more countries have estimates of opiate than opioid use, we used those countries that had estimates of prevalence of both (51 countries) to calculate regional estimates of opioid to opiate use. We followed the procedure of weighting by population size as per the estimates of regional global PWID estimates in Mathers et al. and related papers.[8,20,67,68,122] This allowed the calculation of regional estimates of the ratio of opioid to opiate use in all regions (using the same regions as the aforementioned paper) except the Pacific Islands for which a global average was used. Having done this, estimates of opioid use were calculated for all countries with either estimates of opioid prevalence or values inferred using the above ratios from opiate use, resulting in opioid use prevalence in 128 countries. Regional averages were constructed from these data for opioid use prevalence and applied to countries lacking either opioid or opiate data. Lastly, the number on OST was divided by the estimated number of opioid users in the relevant year to calculate a coverage of OST among all opioid users. We capped coverage at 80%, the highest coverage achieved in Europe (see this European Monitoring Centre for Drugs and Addiction report[123]), as two countries with inferred opioid prevalence values had higher reported coverage. Lastly, we assume that the coverage among opioid-dependent PWID is equivalent to the coverage among opioid users in general. In Europe, 38% of those entering treatment inject as their primary route of administration,[123] while our estimates suggest that 30% of the region's opioid-dependent people are PWID, justifying equating the coverage of OST among opioid-dependent people with that among opioid-dependent PWID. According to this procedure, 16 countries had coverage of OST higher than 40 per 100 opioid-dependent PWID, defined as a high target coverage by WHO,[66] while globally 5% of opioid-dependent PWID received OST.

Genotype distributions. Genotype distributions were taken from Messina et al. (2015).[4] GBD regional averages calculated in the paper were used where data were not available for a given country. An average genotype distribution for Oceania was not calculated for lack of data; for these countries the distribution for Australasia was used.

Historical diagnosis and treatment. 67 country values for the proportion diagnosed and the proportion subsequently treated (for one of the years 2013, 2014 or 2015) were extracted from published reviews.[41,108–110] We updated 2013 and 2014 proportion diagnosed values to 2015 values by adding the published estimate of number diagnosed per year to the then current number diagnosed, taking into account estimates of new infections and deaths in the intervening years. We assumed that the treatment rate in 2015 was equal to the 2013 or 2014 value where necessary. In two cases (Egypt and India), significant numbers starting treatment in 2015 were reported (greater than the treatment numbers implied in our approach).[99] We, therefore, added these treatments to the original projections of treatment numbers to estimate updated treatment rates. To impute values for the remaining countries and territories, regional averages were used.[111] Specifically, we calculated regional estimates for the remaining countries such that the overall treatment cascade numbers in 2015 matched WHO regional estimates.[111]

In countries with reported DAA use in 2016[116], the rate of treatment in 2016 is chosen so that the number reported as starting DAA treatment in that year is matched in the model. No PEG-IFN+RBV is simulated in those countries that have transitioned to DAAs.

Whether or not countries experienced a reduction in general population risk was based on average healthcare spending, 1995-2014.[59] The small number of countries lacking such data were assumed not to be in the top quartile of health spending.

Initialising the model and technical details. The model is fit to data starting in 1990 (see Calibration data); we run the model from 1890, such that a full 100-year cycle of ageing has been simulated before calibration is carried out, allowing the age distribution of HCV-infected people to stabilise. The model is run with 1950's demographic information from 1890 to 1950, with a seed value of 0·1% HCV infected. The population size is rescaled in 1950 to correct the population size and the simulation proceeds from this date with the appropriate demographic parameters.

The model was coded in C++14,[124] with figures produced using ggplot2[125] and rworldmap[126] in R version 3·4·3[127] (additional packages[128–136]). The equations were solved using an Euler scheme, with a time-step of $dt = 0{\cdot}2$ years. This introduces minimal error (result not shown) as would be expected by the slow nature of both the natural history of the disease and the epidemic itself.

<u>Calibration data</u>

Mortality. Estimates of the number of deaths due to compensated/decompensated cirrhosis (defined as "liver cirrhosis, chronic viral hepatitis infections and hepatic decompensation events"[137]) and HCC are taken from Global Burden of Disease (GBD) group estimates (2016 update, see this website[138]) see also Stanaway et al. (2016)[139] for further details on methods. Mortality estimates were downloaded for the years 1990 to 2015 in five-year intervals (2015 value replaced with a 2013 value for Macao Special Administrative

Region (SAR) and Hong Kong SAR as these regions were not included in the 2016 GBD update so numbers from the 2013 updates were used). Estimates were grouped into the age ranges 5-15, 15-50, 50-70 and over 70 years old. These outputs were chosen to enable the model to capture the broad trends of the GBD estimates both temporally and by age. As HCV prevalence information is more limited than GBD estimates, all countries that were simulated were required to have GBD estimates.

Prevalence. HCV viraemic prevalence data for the overall population are 2015 estimates, used by the WHO in formulating their targets.[15,111] Such estimates, based on individual country-level data, cover 86·1% of the global population. Where data are lacking, the only constraint on prevalence is that it must be in excess of the lower bound prevalence for that country's GBD region.

HCV antibody positive prevalence data in PWID are taken from Degenhardt et al. (2017).[20] These values are combined with the percentage HCV viraemic (the viraemic rate) to calculate a viraemic HCV prevalence. The viraemic rate is assumed equal in the population as a whole and the PWID population, and is from a review of published estimates.[15] Countries with data comprise 90·2% of the global PWID population. Where data are lacking, PWID prevalence is not included in the likelihood calculation.

<u>List of countries by region</u>

190 countries and territories were simulated. The only regions with populations greater than 100 000 not modelled were French and Dutch overseas territories. These were not simulated because there are no GBD mortality estimates for these regions. GBD mortality numbers for China include Macao SAR and Hong Kong SAR; to simulate mainland China independently the China mortality rates were combined with population sizes to produce mortality numbers for the Chinese mainland only. Mortality information from the 2013 update of GBD was used for Hong Kong SAR and Macao SAR, since this update included mortality estimates specific to these regions (the 2016 update does not).

For the purposes of calculating the year of elimination, simulated countries were categorised according to the 21 regions used by the GBD group:[140]

- Central Asia: Armenia, Azerbaijan, Georgia, Kazakhstan, Kyrgyzstan, Mongolia, Tajikistan, Turkmenistan and Uzbekistan.
- Central Europe: Albania, Bosnia and Herzegovina, Bulgaria, Croatia, Czech Republic, Hungary, Macedonia, Montenegro, Poland, Romania, Serbia, Slovakia and Slovenia.
- Eastern Europe: Belarus, Estonia, Latvia, Lithuania, Moldova, Russia and Ukraine.
- Australasia: Australia and New Zealand.
- High-income Asia Pacific: Brunei, Japan, Singapore and South Korea.
- High-income North America: USA and Canada.
- Southern Latin America: Argentina, Chile and Uruguay.
- Western Europe: Austria, Belgium, Cyprus, Denmark, Finland, France, Germany, Greece, Iceland, Ireland, Israel, Italy, Luxembourg, Malta, Netherlands, Norway, Portugal, Spain, Sweden, Switzerland and United Kingdom.
- Andean Latin America: Bolivia, Ecuador and Peru.
- Caribbean: Antigua and Barbuda, The Bahamas, Barbados, Belize, Cuba, Dominican Republic, Grenada, Guyana, Haiti, Jamaica, Puerto Rico, Saint Lucia, Saint Vincent and the Grenadines, Suriname, Trinidad and Tobago and US Virgin Islands.
- Central Latin America: Colombia, Costa Rica, El Salvador, Guatemala, Honduras, Mexico, Nicaragua, Panama and Venezuela.
- Tropical Latin America: Brazil and Paraguay.
- North Africa and Middle East: Afghanistan, Algeria, Bahrain, Egypt, Iran, Iraq, Jordan, Kuwait, Lebanon, Libya, Morocco, Palestine, Oman, Qatar, Saudi Arabia, Sudan, Syria, Tunisia, Turkey, United Arab Emirates and Yemen.
- South Asia: Bangladesh, Bhutan, India, Nepal and Pakistan.
- Southeast Asia: Cambodia, Indonesia, Laos, Malaysia, Maldives, Mauritius, Myanmar, Philippines, Sri Lanka, Seychelles, Thailand, Timor-Leste and Viet Nam.
- East Asia: China, Hong Kong SAR, Macao SAR, Taiwan and North Korea.
- Oceania: Federated States of Micronesia, Fiji, Guam, Kiribati, Papua New Guinea, Samoa, Solomon Islands, Tonga and Vanuatu.
- Central Sub-Saharan Africa: Angola, Central African Republic, Congo, Democratic Republic of the Congo, Equatorial Guinea and Gabon.

- Eastern Sub-Saharan Africa: Burundi, Comoros, Djibouti, Eritrea, Ethiopia, Kenya, Madagascar, Malawi, Mozambique, Rwanda, Somalia, South Sudan, Tanzania, Uganda and Zambia.
- Southern Sub-Saharan Africa: Botswana, Lesotho, Namibia, South Africa, Swaziland and Zimbabwe.
- Western Sub-Saharan Africa: Benin, Burkina Faso, Cameroon, Cape Verde, Chad, Côte d'Ivoire, The Gambia, Ghana, Guinea, Guinea-Bissau, Liberia, Mali, Mauritania, Niger, Nigeria, São Tomé and Príncipe, Senegal, Sierra Leone and Togo.

Other studies have utilised different regional groupings. One recent study analyses the European Union (EU)[115], which comprises the following 28 countries (as of that study's publication date):

- EU: Austria, Italy, Belgium, Latvia, Bulgaria, Lithuania, Croatia, Luxembourg, Cyprus, Malta, Czech Republic, Netherlands, Denmark, Poland, Estonia, Portugal, Finland, Romania, France, Slovakia, Germany, Slovenia, Greece, Spain, Hungary, Sweden, Ireland and United Kingdom.

Lastly, we utilised WHO data on the percentage diagnosed and treated. These values are reported according to WHO office region. The constituent countries of the six regions (of those included in the model) are:

- African Region – AFRO: Algeria, Angola, Benin, Botswana, Burkina Faso, Burundi, Cameroon, Cape Verde, Central African Republic, Chad, Comoros, Congo, Côte d'Ivoire, Democratic Republic of the Congo, Equatorial Guinea, Eritrea, Ethiopia, Gabon, Gambia, Ghana, Guinea, Guinea-Bissau, Kenya, Lesotho, Liberia, Madagascar, Malawi, Mali, Mauritania, Mauritius, Mozambique, Namibia, Niger, Nigeria, Rwanda, São Tomé and Príncipe, Senegal, Seychelles, Sierra Leone, South Africa, Swaziland, Tanzania, Togo, Uganda, Zambia and Zimbabwe.
- Region of the Americas – PAHO: Antigua and Barbuda, Argentina, Bahamas, Barbados, Belize, Bolivia, Brazil, Canada, Chile, Colombia, Costa Rica, Cuba, Dominican Republic, Ecuador, El Salvador, Grenada, Guatemala, Guyana, Haiti, Honduras, Jamaica, Mexico, Nicaragua, Panama, Paraguay, Peru, Puerto Rico, Saint Lucia, Saint Vincent and the Grenadines, Suriname, Trinidad and Tobago, USA, Uruguay, Venezuela and US Virgin Islands.
- Eastern Mediterranean Region – EMRO: Afghanistan, Bahrain, Djibouti, Egypt, Iran, Iraq, Jordan, Kuwait, Lebanon, Libya, Morocco, Oman, Pakistan, Qatar, Saudi Arabia, Somalia, South Sudan, Sudan, Syria, Tunisia, United Arab Emirates and Yemen.
- European Region – EURO: Albania, Armenia, Austria, Azerbaijan, Belarus, Belgium, Bosnia and Herzegovina, Bulgaria, Croatia, Cyprus, Czech Republic, Denmark, Estonia, Finland, France, Georgia, Germany, Greece, Hungary, Iceland, Ireland, Israel, Italy, Kazakhstan, Kyrgyzstan, Latvia, Lithuania, Luxembourg, Macedonia, Malta, Moldova, Montenegro, Netherlands, Norway, Palestine, Poland, Portugal, Romania, Russia, Serbia, Slovakia, Slovenia, Spain, Sweden, Switzerland, Tajikistan, Turkey, Turkmenistan, Ukraine, United Kingdom and Uzbekistan.
- South-East Asia Region – SEARO: Bangladesh, Bhutan, North Korea, Hong Kong SAR, India, Indonesia, Macao SAR, Maldives, Myanmar, Nepal, Sri Lanka, Taiwan, Thailand and Timor-Leste.
- Western Pacific Region – WPRO: Australia, Brunei Darussalam, Cambodia, China, Fiji, Guam, Japan, Kiribati, Laos, Malaysia, Micronesia, Mongolia, New Zealand, Papua New Guinea, Philippines, Samoa, Singapore, Solomon Islands, South Korea, Tonga, Vanuatu and Viet Nam.

Supplementary figures

Figure S1: Annual number of treatments

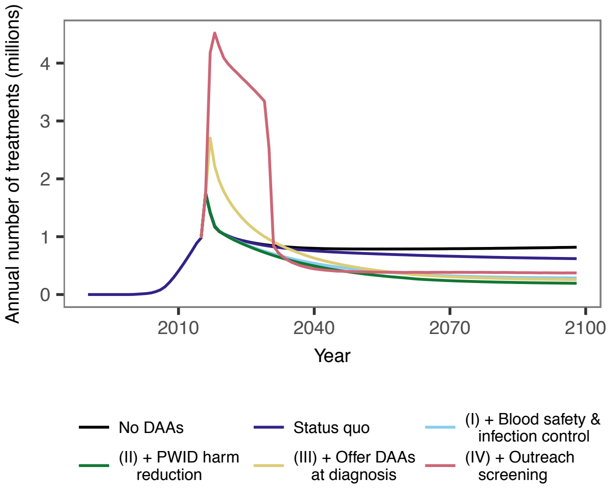

Annual treatment numbers for all intervention scenarios.

Figure S2: Mortality reductions by country and intervention in 2030

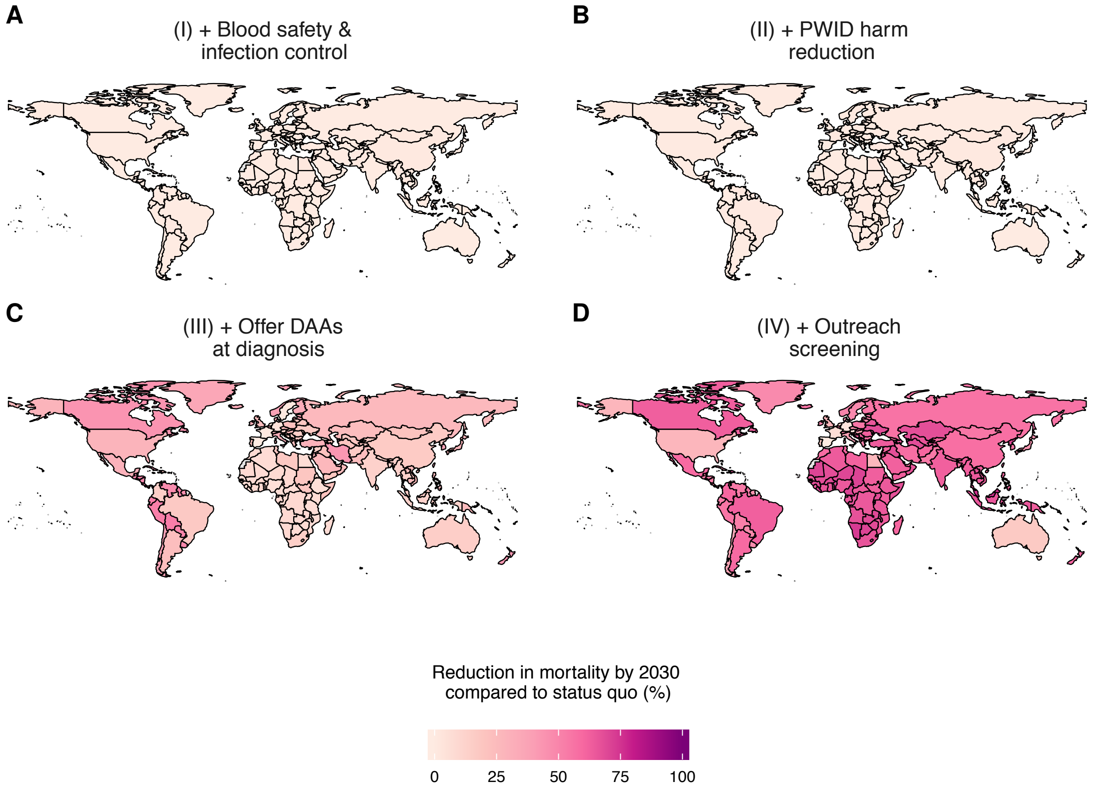

Reduction in annual mortality, calculated for 190 countries by intervention, as compared to the status quo in the same year. Each graph shows the mortality reduction comparing the status quo to (A) intervention I – status quo + blood safety and infection control, (B) intervention II – status quo + blood safety and infection control + PWID harm reduction, (C) intervention III – status quo + blood safety and infection control + PWID harm reduction + DAAs in all countries and offer DAAs at diagnosis, (D) intervention IV – status quo + blood safety and infection control + PWID harm reduction + DAAs in all countries and offer DAAs at diagnosis + outreach screening.

Figure S3: Incidence reductions by country and intervention in 2030

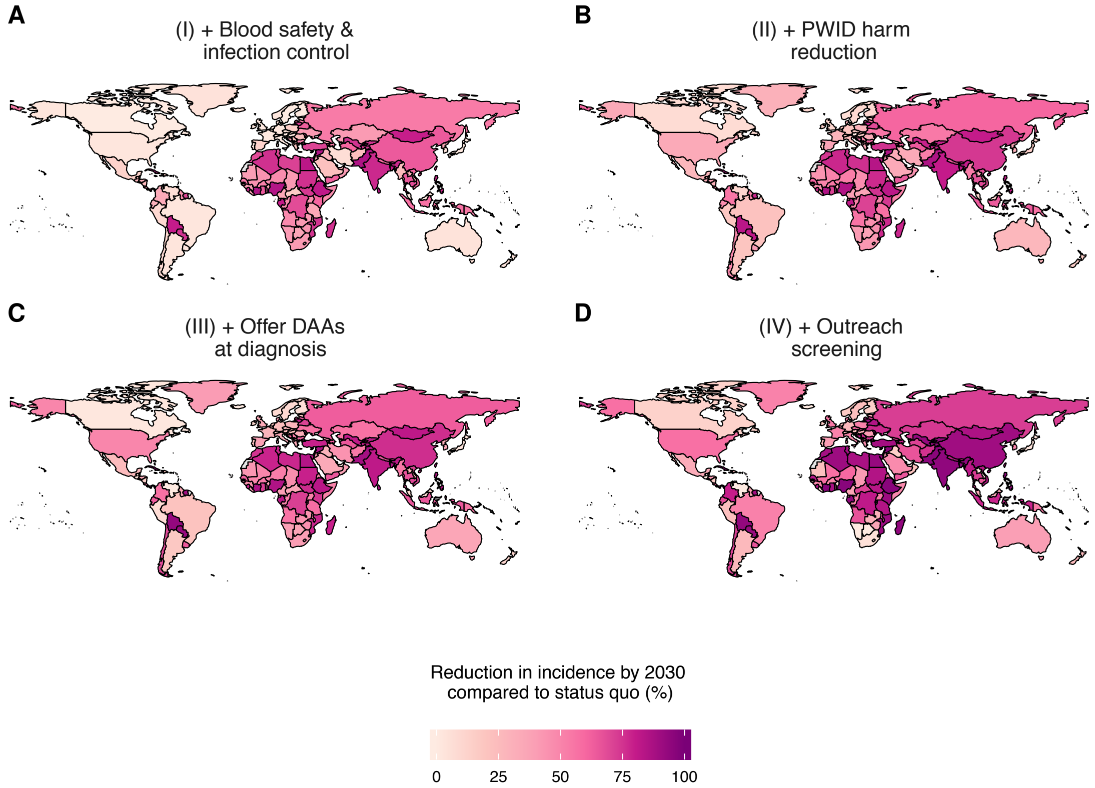

Reduction in annual incidence, calculated for 190 countries by intervention, as compared to the status quo in the same year. Each graph shows the incidence reduction comparing the status quo to: (A) intervention I – status quo + blood safety and infection control, (B) intervention II – status quo + blood safety and infection control + PWID harm reduction, (C) intervention III – status quo + blood safety and infection control + PWID harm reduction + DAAs in all countries and offer DAAs at diagnosis, (D) intervention IV – status quo + blood safety and infection control + PWID harm reduction + DAAs in all countries and offer DAAs at diagnosis + outreach screening. In a small number of instances, projected incidence is greater in an intervention than in status quo (shown as a country coloured grey). One example is Namibia under intervention IV. The countries in which interventions can potentially have a negative impact are all characterised by having variable numbers of HCV-positive PWID (since all have no data on PWID HCV prevalence which is not, therefore, constrained in fitting). As such, the simulations explore epidemics that are concentrated in PWID; upon curing a greater number of individuals with DAAs in intervention IV, there is a sudden increase in the susceptible PWID population who are then reinfected, causing an increase in absolute number of new infections and thus an increase in overall population incidence. Notably incidence within PWID alone actually decreases in these simulations, and the rise in overall incidence pertains to the increase in the size of the susceptible pool as a result of curing more individuals with DAAs.

Figure S4: Sensitivity of reaching WHO elimination targets to changes in programme parameters when implementing the comprehensive package of interventions

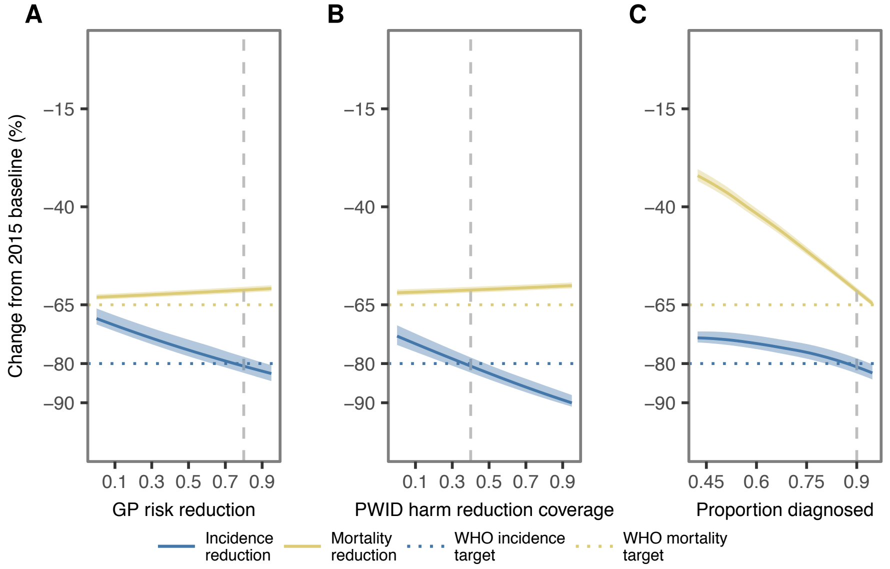

Sensitivity of reaching elimination targets in 2030, expressed as change in 2030 value from 2015 baseline, by (A) reduction in general population risk, (B) coverage of PWID harm reduction intervention and (C) proportion diagnosed. The values of all variables aside from those varied in sensitivity are equal to their original outreach screening intervention values, see the vertical dashed lines on each plot (and the table in the main text).

Figure S5: Sensitivity of reaching WHO elimination targets to changes in programme parameters with the comprehensive package of interventions, as a function of year of elimination

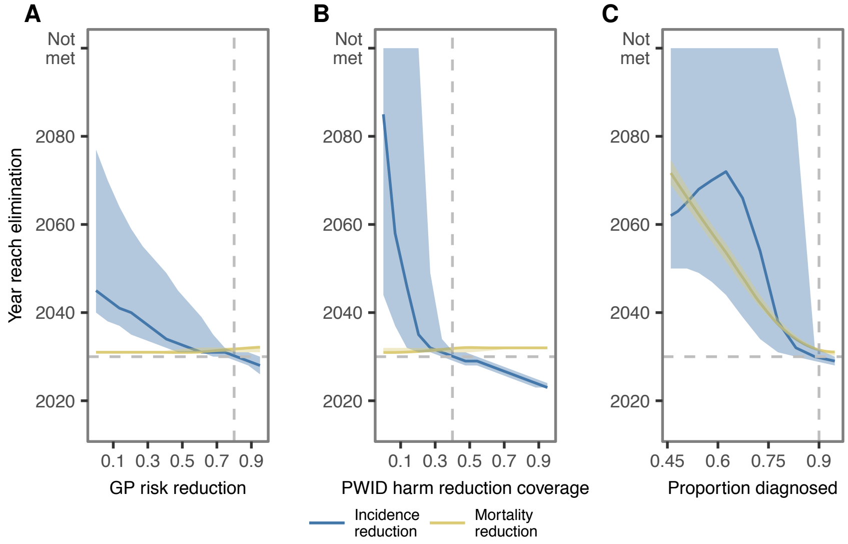

Sensitivity of reaching elimination targets before 2100 by (A) reduction in general population risk, (B) coverage of PWID harm reduction intervention and (C) proportion diagnosed. The values of all variables aside from those varied in sensitivity are equal to their original intervention IV values, see the vertical dashed lines on each plot (and the table in the main text). Horizontal dashed line shows 2030 (the elimination-year target).

Figure S6: Proportion of infections in PWID

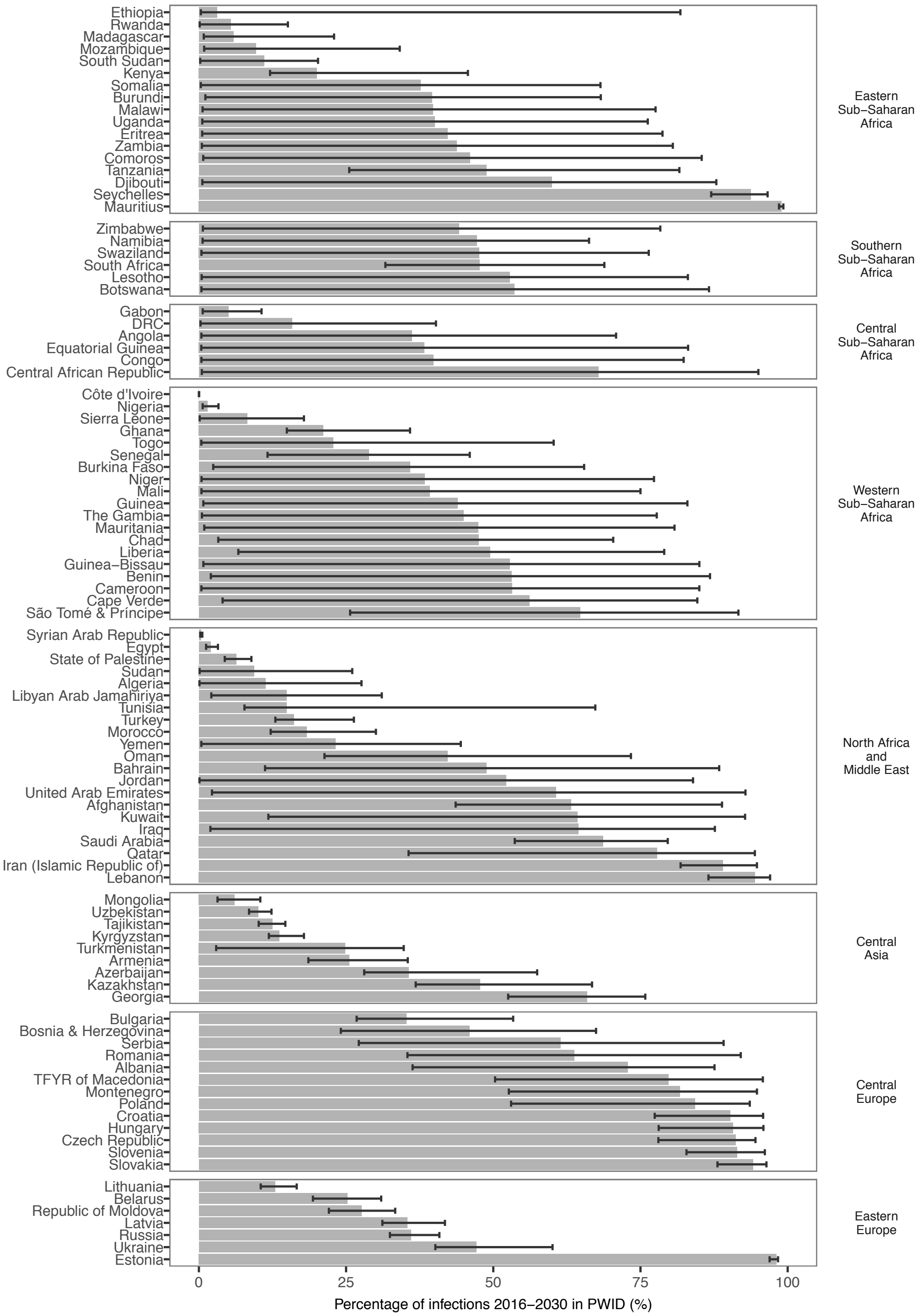

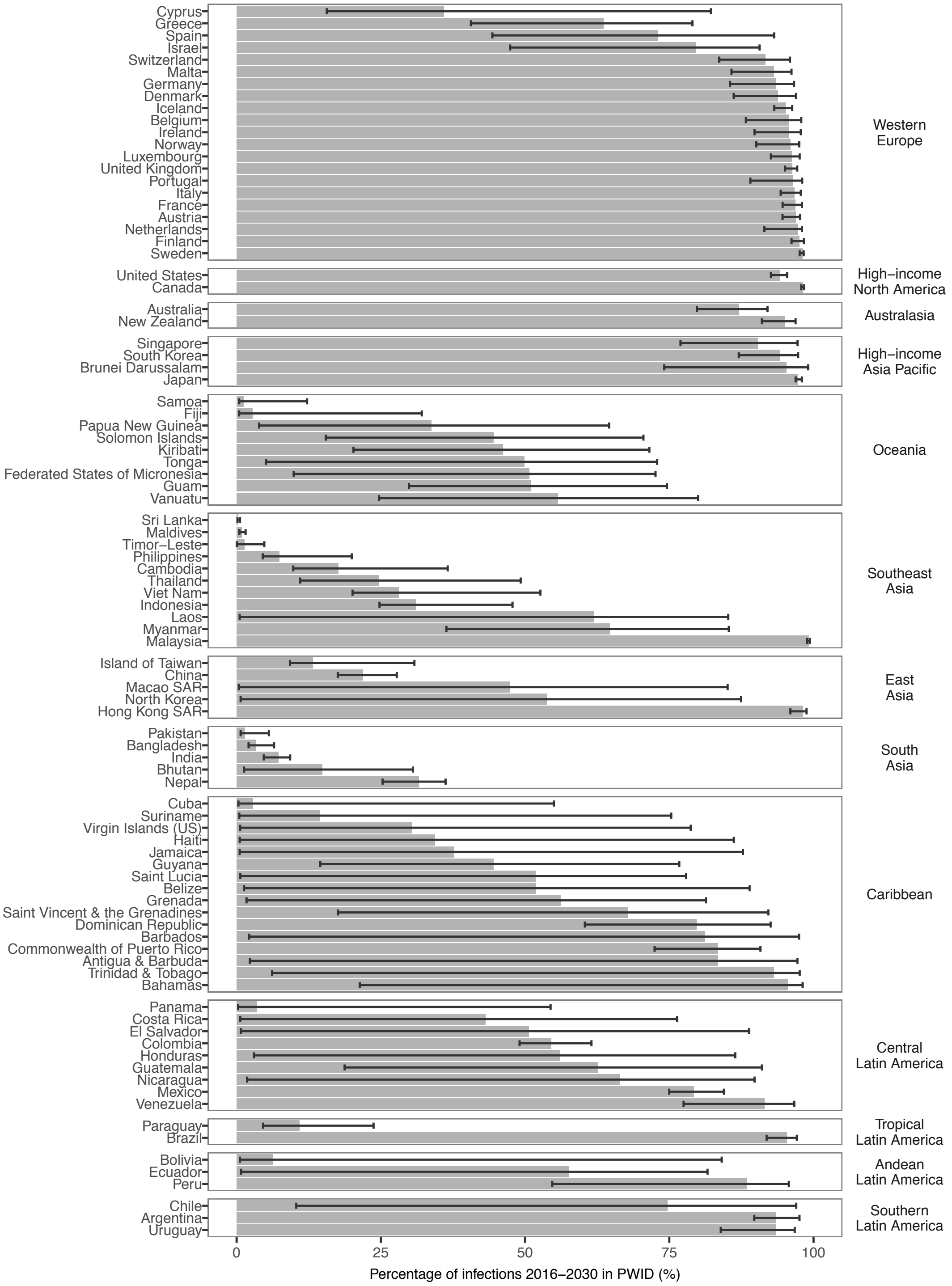

Bars represent the median proportion of new infections in PWID (2016-2030) in the status quo, relative to the overall number of new infections. Error bars are 95% credible intervals.

Figure S7: Sensitivity of reaching WHO elimination targets upon removal of systemically important countries

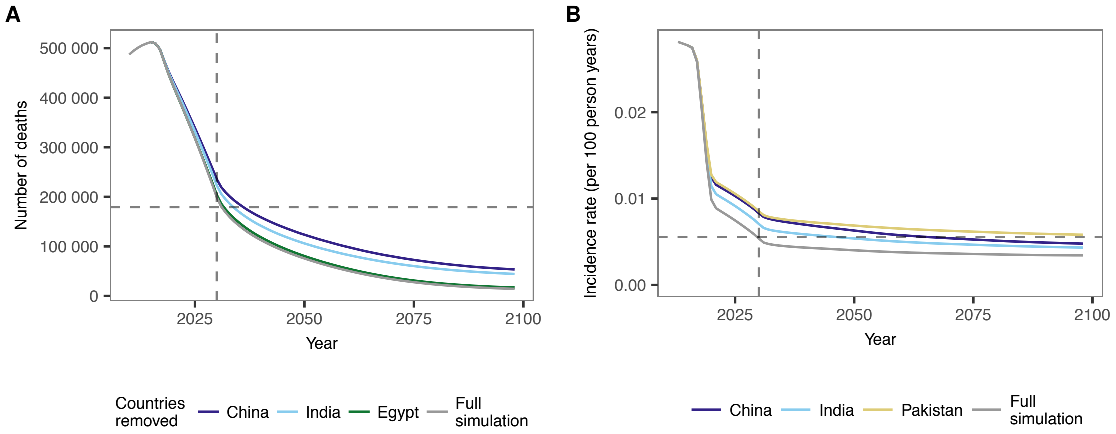

Number of deaths (A) and incidence (B) for the comprehensive package of interventions and for the comprehensive package of interventions upon removal of the three countries that are projected to contribute most to mortality or number of infections in the status quo (2016-2030). Credible intervals not shown for ease of interpretation.

Figure S8: Impact of all interventions on global HCV epidemic with assumption of lower PWID programme harm reduction effectiveness

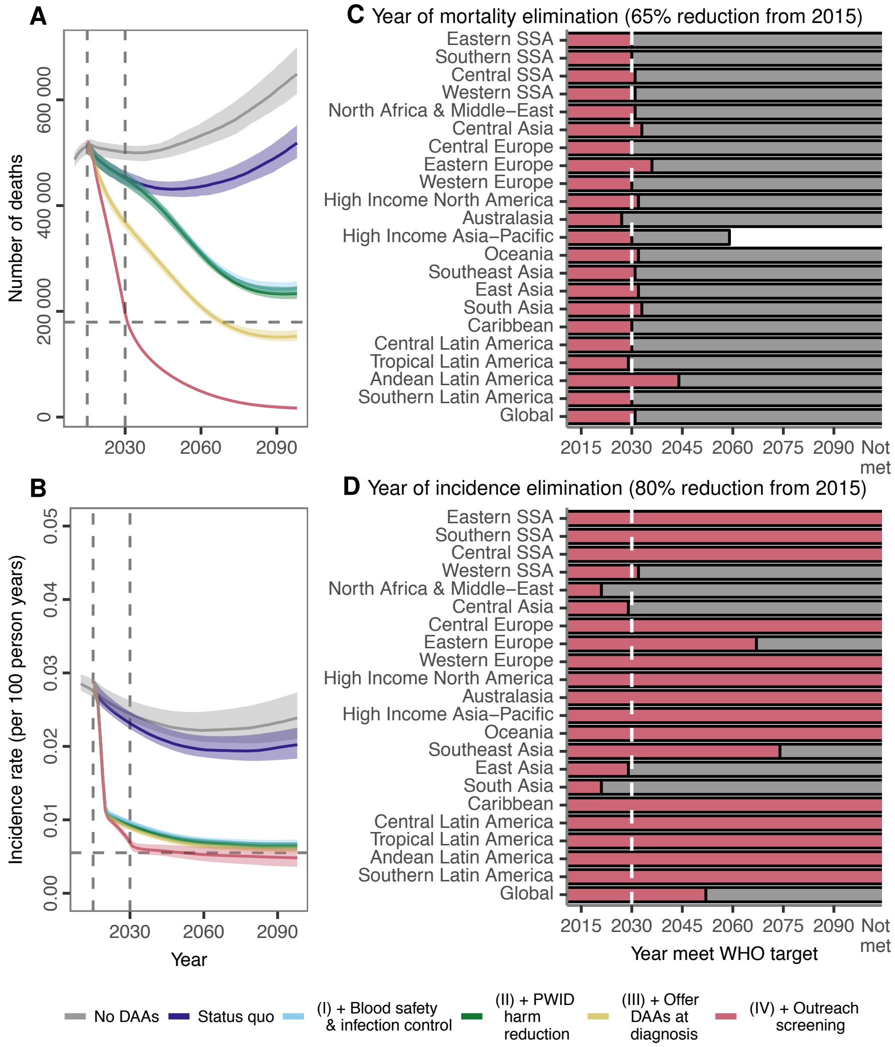

Impact of interventions when assuming that PWID harm reduction programmes reduce risk by 20%. Note that the green and blue curves in figures (A) and (B) are mostly coincident reflecting the fact that the impact of add PWID harm reduction is much lower as would be expected. (A) Number of deaths due to HCV by scenario. (B) Incidence of viraemic HCV infection (number of chronic infections divided by number susceptible) by scenario. Note that the incidence curves for interventions II and III are almost coincident. Vertical dashed lines indicate baseline (2015) and the year for targeted elimination (2030). Horizontal dashed line indicates elimination targets: 65% reduction of mortality and 80% reduction in incidence. (C and D) End of each bar represents the median year of (C) mortality and (D) incidence elimination. Grey bars show no DAA scenario; red bars show the comprehensive package of interventions (intervention IV). Where a red bar extends to the edge of the graph, elimination was not achieved in any scenario before 2100. The dashed white vertical line indicates the WHO elimination year target (2030).

Figure S9: Impact on elimination year in comprehensive package of interventions upon varying the relative risk (RR) of reinfection.

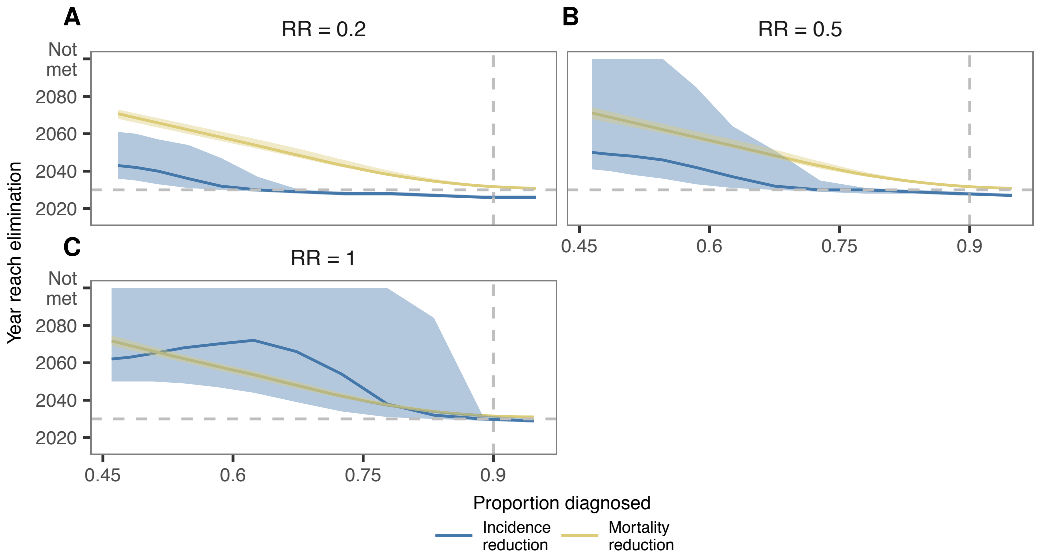

Shown are the times to elimination in the comprehensive package of interventions at varying diagnosis coverages (in 2030) with a reinfection relative risk (RR) of (A) 0·2, (B) 0·5 and (C) 1 which is the value used in the primary analysis (this is subfigure C in Figure S5).

Figure S10: Compartmental model diagram

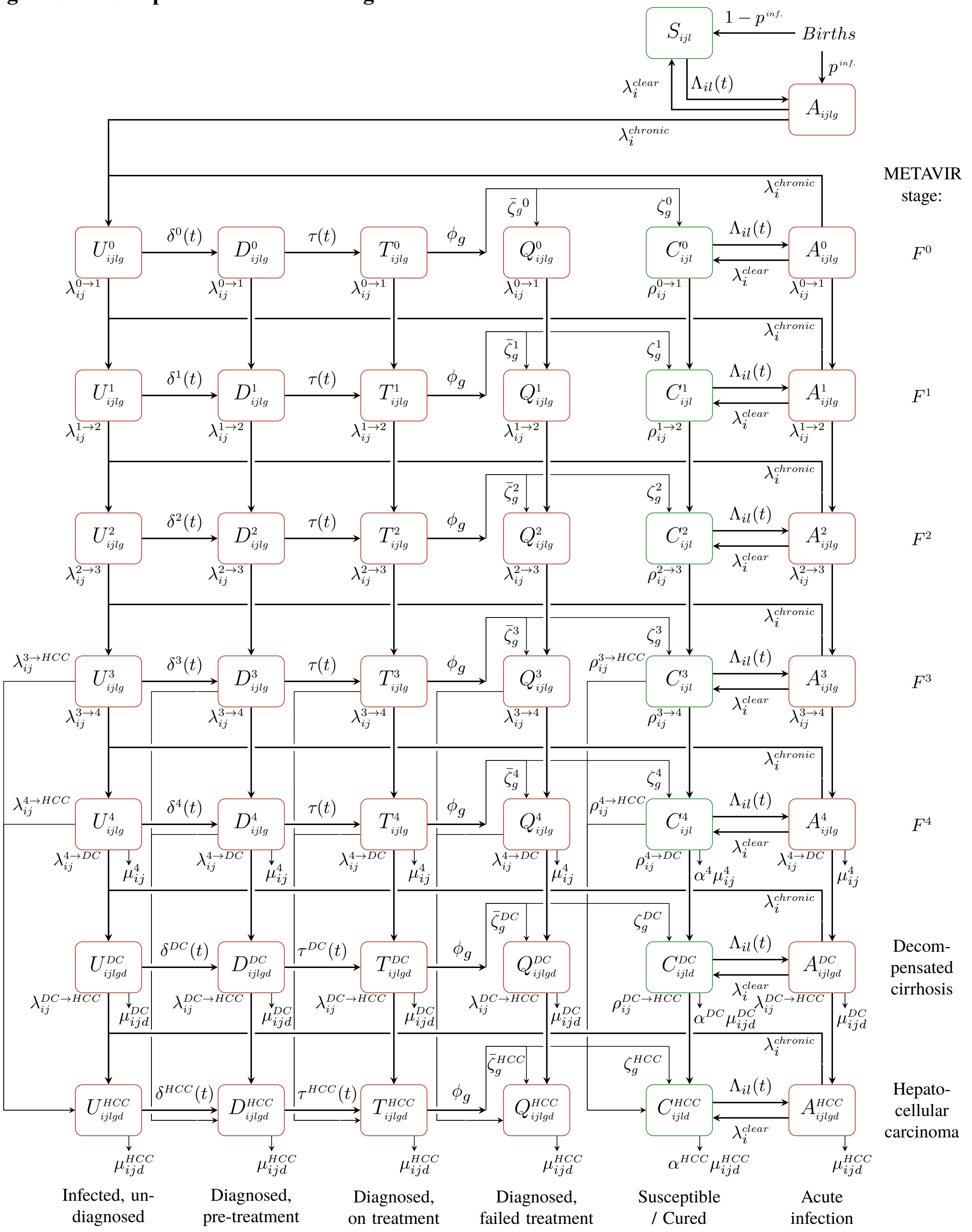

Boxes (compartments) represent people. These are subdivided into age – i, sex -j, risk group - l, genotype - g (where appropriate) and duration of infection - d (DC and HCC only). Arrows and associated symbols represent annual transition rates; functional dependence is shown where necessary. Green boxes do not contribute to the force of infection. Background mortality – $\mu_{ijl}^{nat.}(t)$ - and migration – $\nu(t)$ - occur from all compartments. All symbols are defined in Table S1.

Figure S11: Example of fibrosis progression rates by age and sex

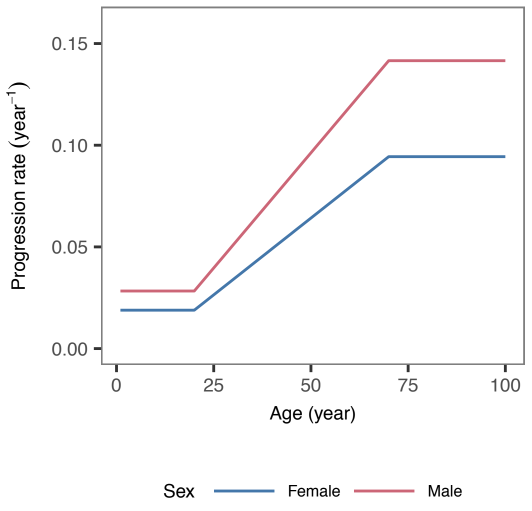

Shown is the F1 to F2 transition rate graphed with the mean value of the annual transition probability chosen (i.e. $\alpha_{age}^{fibr.} = 0{\cdot}5$), a male to female relative rate of $h_{sex}^{fibr.} = 1{\cdot}5$ and a 70- to 20-year-old relative probability of $h_{age}^{fibr.} = 5$.

Supplementary equations and tables

Equations S1: Partial differential equations defining the model

$$\frac{\partial S}{\partial t}+\frac{\partial S}{\partial a}=\left(1-p_{inf.}\right)b(t)+\sum_{g}\lambda_i^{clear}\,A-\left(\Lambda_{il}(t)+\mu_{ijl}^{nat.}(t)-\nu(t)\right)S$$

$$\frac{\partial A}{\partial t}+\frac{\partial A}{\partial a}=p_{inf.}b(t)+\Lambda_{il}(t)S-\left(\lambda_i^{clear}+\lambda_i^{chronic}+\mu_{ijl}^{nat.}(t)-\nu(t)\right)A$$

$$\frac{\partial A^k}{\partial t}+\frac{\partial A^k}{\partial a}+\frac{\partial A^k}{\partial d}=\theta_{reinf}\Lambda_{il}(t)C^k+\sum_{k'\to k}\lambda_{ij}^{k'\to k}A^{k'}$$
$$-\left(\lambda_i^{clear}+\lambda_i^{chronic}+\sum_{k\to k''}\lambda_{ij}^{k\to k''}+\mu_{ijd}^{k}+\mu_{ijl}^{nat.}(t)-\nu(t)\right)A^k$$

$$\frac{\partial U^k}{\partial t}+\frac{\partial U^k}{\partial a}+\frac{\partial U^k}{\partial d}=\lambda_i^{chronic}\left(A^k+\delta_{k,0}A\right)+\sum_{k'\to k}\lambda_{ij}^{k'\to k}U^{k'}$$
$$-\left(\delta^k(t)+\sum_{k\to k''}\lambda_{ij}^{k\to k''}+\mu_{ijd}^{k}+\mu_{ijl}^{nat.}(t)-\nu(t)\right)U^k$$

$$\frac{\partial D^k}{\partial t}+\frac{\partial D^k}{\partial a}+\frac{\partial D^k}{\partial d}=\delta^k(t)U^k+\sum_{k'\to k}\lambda_{ij}^{k'\to k}D^{k'}$$
$$-\left(\tau^k(t)+\sum_{k\to k''}\lambda_{ij}^{k\to k''}+\mu_{ijd}^{k}+\mu_{ijl}^{nat.}(t)-\nu(t)\right)D^k$$

$$\frac{\partial T^k}{\partial t}+\frac{\partial T^k}{\partial a}+\frac{\partial T^k}{\partial d}=\tau^k(t)D^k+\sum_{k'\to k}\lambda_{ij}^{k'\to k}T^{k'}$$
$$-\left(\phi_g+\sum_{k\to k''}\lambda_{ij}^{k\to k''}+\mu_{ijd}^{k}+\mu_{ijl}^{nat.}(t)-\nu(t)\right)T^k$$

$$\frac{\partial Q^k}{\partial t}+\frac{\partial Q^k}{\partial a}+\frac{\partial Q^k}{\partial d}=\bar{\zeta}_g^k\phi_g T^k+\sum_{k'\to k}\lambda_{ij}^{k'\to k}Q^{k'}$$
$$-\left(\sum_{k\to k''}\lambda_{ij}^{k\to k''}+\mu_{ijd}^{k}+\mu_{ijl}^{nat.}(t)-\nu(t)\right)Q^k$$

$$\frac{\partial C^k}{\partial t}+\frac{\partial C^k}{\partial a}+\frac{\partial C^k}{\partial d}=\zeta_g^k\phi_g T^k+\lambda_i^{clear}A^k+\sum_{k'\to k}\rho_{ij}^{k'\to k}C^{k'}$$
$$-\left(\Lambda_{il}(t)+\sum_{k\to k''}\rho_{ij}^{k\to k''}+\alpha^k\mu_{ijd}^{k}+\mu_{ijl}^{nat.}(t)-\nu(t)\right)C^k$$

The following shorthand notations are used: i, j, l, g, d indices dropped from state vectors for ease of reading, so A stands for A_{ijlgd} and so forth; $\sum_{k'\to k}\lambda_{ij}^{k'\to k}$ implies a sum over all the disease stages k' that can move into stage k; $\sum_{k\to k''}\lambda_{ij}^{k\to k''}$ is a sum over all disease stages k'' that can be moved into from stage k (Figure S8 shows all possible connections between the disease stages of a particular model compartment); μ_{ijd}^{k} refers to the excess mortality at disease stage k – this is zero for the majority of compartments for which there is no excess mortality due to HCV infection; the rate of change of a compartment with respect to duration, $\partial U^k/\partial d$ and so on, is zero for all disease stages except $k=$ DC/HCC since only in these stages is duration of the condition monitored; $\delta_{k,0}=0$ for $k\neq 0$ and $\delta_{k,0}=1$ for $k=0$.

Table S1: Full list of parameter symbols and derived quantities with values and prior distributions

Parameter	Description	Value(s) or relation to other parameters	Justification and references
Demographic parameters			
$b(t)$	Number of births	Calculated from sex ratio, fertility rate and number of females; depends on country and time	Historical values and future projections from UN population prospects[141]
$\mu_{ijl}^{nat.}(t)$	Background mortality rate	$\sigma_l \mu_{ij}^{nat.}(t)$	See below
σ_l	Standardised mortality ratio	$l = 1$: 5-16	PWID SMR range from UNODC World Drug Report (2017)[28] and Mathers et al. (2013)[27]
		Otherwise: 1	Non-PWID have mortality rate given by background rate
$\mu_{ij}^{nat.}(t)$	Background mortality rate	Depends on country, age, sex and time	Historical values and future projections from UN population prospects[141]
$\nu(t)$	Migration	Depends on country	Historical values and future projections from UN population prospects[141]
π^{PWID}, π_{female}^{PWID}, $\pi_{opioid-dept.}^{PWID}$	Proportion PWID	Depends on country	Country-specific, regional or global value where appropriate from Degenhardt et al. (2017)[20]
	Proportion PWID women		
	Proportion PWID opioid-dependent		
Transmission parameters			
$p^{inf.}$	Probability of perinatal infection	0·027	Ferrero et al. (2003) [142]
$\Lambda_{il}(t)$	Force of infection; dependent on age – i - and risk group – l = GP, PWID	GP: $\beta_{i,GP}(t)\, p_{all}^{eff}(t)$	See below
		PWID: $\beta_{i,GP}(t)\, p_{all}^{eff}(t) + \beta_{PWID}(t)\, p_{PWID}(t)$	
$p_{all}^{eff}(t)$	Effective HCV prevalence; $I_i(t)$ is number infected by age, $N_i(t)$ is population size by age	$\sum_i \beta_{i,GP}(t)\, I_i(t) / \sum_i \beta_{i,GP}(t)\, N_i(t)$	Anderson et al. (1992)[56]
$p_{PWID}(t)$	HCV prevalence among PWID	$I^{pwid}(t)/N^{pwid}(t)$	Anderson et al. (1992)[56]
$\beta_{i,GP}(t)$	Time and age dependent risk of infection in general population	$\gamma_i \beta_{GP}(t)$	See below
$\beta_{GP}(t)$	Time-varying contribution to general population risk of infection	Interpolated spline with risk values at years 1930, 1950, 1970, 1990 and 2005: ξ_{1930}^{GP}, ξ_{1950}^{GP}, ξ_{1970}^{GP}, ξ_{1990}^{GP}, ξ_{2005}^{GP}	See below
γ_i	Age-dependent general population risk multiplier	Interpolated spline with multiplicative values at ages 33, 66, 100 years old: ξ_{33}^{GP}, ξ_{66}^{GP}, ξ_{100}^{GP}	See below
$\beta_{PWID}(t)$	Risk of infection in PWID	Interpolated spline with risk values at years 1950, 1980: ξ_{1950}^{PWID}, ξ_{1980}^{PWID}	See below
$\xi_{i/t}^{l}$		ξ_{1930}^{GP}: ≥ 0, exponential prior (rate = 1·00)	

	Values of spline knots by risk group l, at time t or age i	ξ_{1950}^{GP}: ≥ 0, exponential prior (rate = 0·05)	Pre-1930 risk of infection low so greater prior weight at lower values; risks at other times drawn from effectively uninformative prior distributions; age risks completely uninformative
		ξ_{1970}^{GP}: ≥ 0, exponential prior (rate = 0·05)	
		ξ_{1990}^{GP}: ≥ 0, exponential prior (rate = 0·05)	
		$\xi_{2005}^{GP} = q^{country}\xi_{1990}^{GP}$	
		ξ_{1950}^{PWID}: ≥ 0, exponential prior (rate = 1·00)	
		ξ_{1980}^{PWID}: ≥ 0, exponential prior (rate = 0·05)	
		ξ_{33}^{GP}: 0 – 1	
		ξ_{66}^{GP}: 0 – 1	
		ξ_{100}^{GP}: 0 – 1	
$q^{country}$	Reduction in GP risk after 1990, by country, quantified using average country healthcare spending from 1995	Spending in top quartile – $q^{country} \geq 0$, exponential prior (rate=50)	Healthcare expenditure per capita from World Bank[59]; greater reduction assumed with greater expenditure
		Otherwise – $q^{country} \geq 0$, exponential prior (rate=5)	
Natural history model parameters			
λ_i^{clear}	Rate of spontaneous clearance	$p^{clear}r^{clear/chronic}$	See below
$\lambda_i^{chronic}$	Rate of progression to chronic disease	$\left(1 - p_i^{clear}\right) r^{clear/chronic}$	See below
p_i^{clear}	Proportion clearing HCV	$i \geq 15$ years old: 0·25	Grebely et al. (2014)[143] Micallef et al. (2006)[144]
		$i < 15$ years old: 0·55	Vogt et al. (1999)[145]
$r^{clear/chronic}$	Rate of moving from acute to either chronic disease or susceptible	2·18 years $^{-1}$	Grebely et al. (2014)[143]
$\lambda_{ij}^{0\to1}$, $\lambda_{ij}^{1\to2}$, $\lambda_{ij}^{2\to3}$, $\lambda_{ij}^{3\to4}$, $\lambda_{ij}^{3\to HCC}$, $\lambda_{ij}^{4\to HCC}$, $\lambda_{ij}^{DC\to HCC}$, $\lambda_{ij}^{4\to DC}$, μ_{ij}^{4}, μ_{ijd}^{DC}, μ_{ijd}^{HCC}	Age and sex dependent fibrosis progression rates and end stage disease progression rates	Calibrated; determined by age via h_{age}^{k}, sex via h_{sex}^{k} and annual transition probabilities $f^{k\to k+1}$ where k denotes disease stage	See below and methods for precise relationship between calibration quantities and literature-derived annual transition probabilities
$f_{l/u}^{0\to1}$	Annual transition probability	lower = 0·05, upper = 0·12	Thein et al. (2008)[39]
$f_{l/u}^{1\to2}$		lower = 0·05, upper = 0·07	
$f_{l/u}^{2\to3}$		lower = 0·08, upper = 0·13	
$f_{l/u}^{3\to4}$		lower = 0·05, upper = 0·13	
$\alpha^{fibr.}$	Scalar controlling rate of fibrosis progression	0-1: 0 gives lower value of all fibrosis progression probabilities in above row; 1 gives upper values	Full range of values
$f^{3\to HCC}$	Annual transition probability: F_3 to HCC	0-0·02	Dienstag et al. (2011)[146]
$f^{4\to HCC}$	Annual transition probability: F_4 to HCC	0·01-0·09	Fattovich et al. (1997)[43] Kato et al. (1994)[147]
$f^{DC\to HCC}$	Annual transition probability: DC to HCC	0·03-0·10	Planas et al. (2004)[148]
$f^{4\to DC}$	Annual transition probability: F_4 to DC	0·02-0·06	Fattovich et al. (1997)[43]
$f^{4\to\mu}$	Annual probability of mortality from F_4	0·02-0·04	D'Amico et al. (2006)[44]

$f^{DC\rightarrow\mu}$	Annual probability of mortality from DC	1st year: 0·07-0·25	Fattovich et al. (1997)[43]
		Later years: 0·07-0·18	
$f^{HCC\rightarrow\mu}$	Annual probability of mortality from HCC	1st year: 0·53-0·75	Altekruse et al. (2009)[149]<br>Shiratori et al. (1995)[150]
		Later years: 0·09-0·38	
$h_{age}^{fibr.}$, $h_{age}^{j\rightarrow HCC}$, $h_{age}^{4\rightarrow DC}$, $h_{age}^{cirr.\rightarrow\mu}$, $h_{age}^{HCC\rightarrow\mu}$	Proportional difference 70- to 20-year-old disease progression rates	1-10	Sweeting et al. (2006)[42]
$h_{sex}^{fibr.}$, $h_{sex}^{j\rightarrow HCC}$, $h_{sex}^{4\rightarrow DC}$, $h_{sex}^{cirr.\rightarrow\mu}$,$h_{sex}^{HCC\rightarrow\mu}$	Proportional difference male to female disease progression rates	1-2	Sweeting et al. (2006)[42]
Treatment, prevention and intervention parameters			
ζ_g^k (PEG-IFN + RBV)	Proportion achieving SVR by genotype g (no treatment DC or HCC) with PEG-IFN+RBV	ζ_1^k = 0·44	Yee et al. (2015)[83]
		ζ_2^k = 0·73	Yee et al. (2015)[83]
		ζ_3^k = 0·73	Yee et al. (2015)[83]
		ζ_4^k = 0·53	Yee et al. (2015)[83]
		ζ_5^k = 0·73	Yee et al. (2015)[83]<br>Nguyen et al. (2005)[84]
		ζ_6^k = 0·75	Bunchorntavakul (2013)[85]
ζ_g^k (DAAs)	Proportion achieving SVR by disease stage (no treatment HCC) with DAAs	F_0 to F_4: 0·98	Forns et al. (2017)[90] - lower confidence interval to be conservative; genotype 3 assumed equal to others
		DC: 0·85	Lens et al. (2017)[93]
$\bar{\zeta}_g^k$	Proportion not achieving SVR	$\bar{\zeta}_g^k = 1 - \zeta_g^k$	Definition
ϕ_g (PEG-IFN + RBV)	Mean rate of treatment course by genotype g with PEG-IFN+RBV	ϕ_1 = 1/48 weeks	Strader et al. (2004)[86]<br>WHO (2014)[87]
		ϕ_2 = 1/24 weeks	Strader et al. (2004)[86]<br>WHO (2014)[87]
		ϕ_3 = 1/24 weeks	Strader et al. (2004)[86]<br>WHO (2014)[87]
		ϕ_4 = 1/48 weeks	WHO (2014)[87]
		ϕ_5 = 1/24 weeks	Nguyen et al. (2005)[84]<br>Strader et al. (2004)[86]
		ϕ_6 = 1/48 weeks	Nguyen et al. (2005)[84]<br>Strader et al. (2004)[86]
ϕ_g (DAAs)	Mean rate of treatment course with DAAs	F^0 to F^4: 1/12 weeks	Forns et al. (2017)[90]
		DC: 1/24 weeks	Lens et al. (2017)[93]
a_{min} (PEG-IFN + RBV)	Minimum age of treatment with PEG-IFN+RBV	15 years old	Approximate minimum age used in guidelines
a_{min} (DAAs)	Minimum age of treatment with DAAs	0 years old	Assume ongoing clinical trials will lead to all-age treatment, see Ohmer et al. (2016)[151]
$\delta_{delay}^{PWID\ re-treat}$	Average time to possible retreatment in PWID after cure	5 years (varied in one way SA: 1-5 years)	Likelihood of multiple retreatments in PWID has not been established; we assume a 5 year average waiting time before retreatment after reinfection

θ_{reinf}	Relative risk of reinfection compared to primary infection	1 (varied in one way SA: 0·2-1)	Evidence on relative risk of reinfection mixed;[69–74] conservative value of one chosen (conservative as increases incidence so worsens outcomes)
$\rho_{ij}^{0\to1}$, $\rho_{ij}^{1\to2}$, $\rho_{ij}^{2\to3}$, $\rho_{ij}^{3\to4}$, $\rho_{ij}^{3\to HCC}$, $\rho_{ij}^{4\to HCC}$, $\rho_{ij}^{DC\to HCC}$, $\rho_{ij}^{4\to DC}$	Age and sex dependent fibrosis progression rates after achieving SVR	$\rho_{ij}^{k\to k+1} = \alpha^{k\to k+1}\lambda_{ij}^{k\to k+1}$	See below
$\alpha^{0\to1}$, $\alpha^{1\to2}$, $\alpha^{2\to3}$, $\alpha^{3\to4}$, $\alpha^{3\to HCC}$	Hazard ratio SVR vs. non-SVR: progression rates before compensated cirrhosis	0	George et al. (2009)[102] and Lee et al. (2014)[103] – as a conservative measure no regression is modelled
$\alpha^{4\to HCC}$	Hazard ratio SVR vs. non-SVR: HCC rate from F4	0·29	Nahon et al. (2017)[104]
$\alpha^{DC\to HCC}$	Hazard ratio SVR vs. non-SVR: HCC rate from DC	0·33	Cheung et al. (2016)[106]
$\alpha^{4\to DC}$	Hazard ratio SVR vs. non-SVR: DC rate from F4	0·26	Nahon et al. (2017)[104]
α^{4}, α^{DC}, α^{HCC}	Hazard ratio SVR vs. non-SVR: mortality rates	$\alpha^{4} = 0$	Bruno et al. (2016)[105]
		$\alpha^{DC} = 1$	Cheung et al. (2016)[106]
		$\alpha^{HCC} = 1$ (SVR after development of HCC – i.e. no treatment effect so treatment not modelled after onset of HCC)	Pol et al. (2016)[89]
		$\alpha^{HCC} = 0{\cdot}41$ (SVR before development of HCC)	Bruno et al. (2017)[107]
δ^{k}	Diagnosis rates	Without outreach screening: $\omega^{k}\Delta$	See below
		With outreach screening: Δ	
ω^{k}	Relative probability of diagnosis by stage k, without outreach screening	$\omega^{0} = 0{\cdot}01/4$	Assume majority of people come for diagnosis at the end stages of disease
		$\omega^{1} = 0{\cdot}01/4$	
		$\omega^{2} = 0{\cdot}01/4$	
		$\omega^{3} = 0{\cdot}01/4$	
		$\omega^{4} = 0{\cdot}1$	
		$\omega^{DC} = 0{\cdot}4$	
		$\omega^{HCC} = 0{\cdot}4$	
Δ	Overall rate of diagnosis without outreach screening	Chosen such that proportion diagnosed linearly scales (from 1990) to 2015 proportion diagnosed	Razavi et al. (2014)[41]<br>Hatzakis et al. (2015)[109]<br>Sibley et al. (2015)[108]<br>Chan et al. (2017)[110]<br>WHO (2017)[111]<br>Kuo et al. (1989)[112]<br>Choo et al. (1989)[113]
	Rate of diagnosis with outreach screening	Chosen such that a proportion $p_{diag}^{max.}$ are diagnosed five years after intervention start	Programmatic aim
$p_{diag}^{max.}$	Maximum proportion diagnosed in outreach screening strategies	Intervention IV - outreach screening: 90%<br>Sensitivity analysis: 20-95%	Programmatic aim. Lower bound in sensitivity analysis is the approximate current global proportion diagnosed
$\tau(t)$	Historical treatment rate and treatment rate in scenarios with no increase in treatment coverage	Chosen such that the annual proportion treated (of those then diagnosed) matches 2015 values or WHO regional estimates	Razavi et al. (2014)[41]<br>Hatzakis et al. (2015)[109]<br>Sibley et al. (2015)[108]<br>Chan et al. (2017)[110]<br>WHO (2017)[111]

	Treatment rate in scenarios with outreach screening	$t < 2016$: as above<br>$t \geq 2016$: Chosen such that 90% of those diagnosed are treated plus 10% of those previously diagnosed but untreated	Programmatic aim
$\tau^{DC}(t)$, $\tau^{HCC}(t)$	Treatment rate in DC and HCC	DC: no treatment under PEG-IFN as contraindicated. Under DAAs, treatment rates as for $\tau(t)$, see above<br>HCC: impact of SVR on patients with HCC under any treatment is contested so zero treatment in this group	Gambato et al. (2014)[88]<br>Pol et al. (2016)[89]
$\Omega_{GP\ risk\ red.}^{intervention}$	Reduction in GP risk in blood and infection control intervention	80%	Programmatic aim; see Intervention strategies for discussion
$\Omega_{PWID\ harm\ red.}$	Reduction in PWID risk in those covered by combination NSP and OST	75%	Hagan et al. (2011)[61]
$\kappa_{PWID\ harm\ red.}^{historical}$	Coverage of historical harm reduction interventions	Transmission risk reduced in presence of > 200 needles & syringes per PWID per year plus OST; percentage coverage defined as coverage of OST provided NSP condition met	NSP values from Larney et al. (2017);[68] OST coverage calculated from data in Larney et al. (2017),[68] European Monitoring Centre for Drugs and Addiction (2017)[123] and United Nations Office on Drugs and Crime database[152]
$\kappa_{PWID\ harm\ red.}^{intervention}$	Coverage of NSP + OST combination in PWID harm reduction intervention	40%	WHO target, see WHO (2012)[66]

Ranges indicate the quantity is varied in calibration or sensitivity analysis using a uniform prior distribution; single values indicated the quantity is fixed, except where an explicit prior distribution is specified.

Table S2: List of calibrated parameters with prior distributions

Parameter	Description	Prior distribution	Values and details
π^{PWID}	Proportion PWID	Uniform	Values drawn from country-specific low-high values or region-specific low-high values as reported in Degenhardt et al. (2017)[20]
σ_l	Standardised mortality ratio among PWID	Uniform	5-16[27,28]
ξ^{GP}_{1930}	Values of spline knots by risk group l, at time t or age i	Exponential	Rate = 1·00
ξ^{GP}_{1950}		Exponential	Rate = 0·05
ξ^{GP}_{1970}		Exponential	Rate = 0·05
ξ^{GP}_{1990}		Exponential	Rate = 0·05
ξ^{GP}_{2005}		$q^{country}\xi^{GP}_{1990}$	NA: $q^{country}$ distribution given below
ξ^{PWID}_{1950}		Exponential	Rate = 1·00
ξ^{PWID}_{1980}		Exponential	Rate = 0·05
ξ^{GP}_{33}		Uniform	0-1
ξ^{GP}_{66}		Uniform	0-1
ξ^{GP}_{100}		Uniform	0-1
$q^{country}$	Reduction in GP risk after 1990, by country, quantified using average country healthcare spending from 1995	Healthcare quartile 1: Exponential	Rate = 50
		Otherwise: Exponential	Rate = 5
$\alpha^{fibr.}$	Scalar controlling rate of fibrosis progression	Uniform	0-1 (0 gives lower value of all fibrosis progression probabilities; 1 gives upper values[39])
$f^{3\rightarrow HCC}$	Annual transition probability: F_3 to HCC	Uniform	0-0·02[146]
$f^{4\rightarrow HCC}$	Annual transition probability: F_4 to HCC	Uniform	0·01-0·09[43,147]
$f^{DC\rightarrow HCC}$	Annual transition probability: DC to HCC	Uniform	0·03-0·10[148]
$f^{4\rightarrow DC}$	Annual transition probability: F_4 to DC	Uniform	0·02-0·06[43]
$f^{4\rightarrow \mu}$	Annual probability of mortality from F_4	Uniform	0·02-0·04[44]
$f^{DC\rightarrow \mu}$, 1st year	Annual probability of mortality from DC	Uniform	0·07-0·25[43]
$f^{DC\rightarrow \mu}$, later years		Uniform	0·07-0·18[43]
$f^{HCC\rightarrow \mu}$, 1st year	Annual probability of mortality from HCC	Uniform	0·53-0·75[149,150]
$f^{HCC\rightarrow \mu}$, later years		Uniform	0·09-0·38[149,150]
$h^{fibr.}_{age}$, $h^{j\rightarrow HCC}_{age}$, $h^{j\rightarrow DC}_{age}$, $h^{cirr.\rightarrow \mu}_{age}$, $h^{HCC\rightarrow \mu}_{age}$	Proportional difference 70- to 20-year-old disease progression rates	Uniform	1-10[42]
$h^{fibr.}_{sex}$, $h^{j\rightarrow HCC}_{sex}$, $h^{j\rightarrow DC}_{sex}$, $h^{cirr.\rightarrow \mu}_{sex}$, $h^{HCC\rightarrow \mu}_{sex}$	Proportional difference male to	Uniform	1-2[42]

	female disease progression rates		

Table S3: Percentage diagnosed and treated in 2015

Country	Percentage diagnosed	Percentage treated of diagnosed	Reference
Country-specific values			
Austria	33	29	Razavi et al. (2017)[115]
Belgium	44	5	
Bulgaria	19	4	
Croatia	24	2	
Cyprus	10	7	
Czech Republic	31	7	
Denmark	63	5	
Estonia	50	5	
Finland	77	2	
France	74	14	
Germany	57	20	
Greece	29	6	
Hungary	48	5	
Ireland	40	7	
Italy	42	10	
Latvia	45	5	
Lithuania	13	23	
Luxembourg	54	7	
Malta	92	1	
Netherlands	60	20	
Poland	18	12	
Portugal	34	17	
Slovakia	9	11	
Slovenia	52	5	
Spain	34	29	
Sweden	85	7	
United Kingdom	39	12	
Bahrain	17	2	
Cameroon	4	8	
Colombia	13	2	
Dominican Republic	10	7	
Ethiopia	3	10	
Ghana	7	0	

Hong Kong	23	6	Chan et al. (2017)[110]
Jordan	21	3	
Kazakhstan	17	3	
Malaysia	8	2	
Morocco	10	10	
Nigeria	4	0	
Oman	19	3	
Qatar	46	28	
Taiwan	43	4	
Iceland	83	3	Sibley et al. (2015)[108]
Indonesia	11	0	
Iran	35	7	
Japan	74	4	
Lebanon	22	11	
Pakistan	16	14	
Romania	17	4	
Saudi Arabia	23	2	
South Korea	42	5	
United Arab Emirates	42	3	
Argentina	37	1	Hatzakis et al. (2015)[109]
India	6	12	
Israel	28	4	
Mexico	35	2	
Mongolia	30	1	
New Zealand	42	5	
Norway	63	5	
Russia	38	0	
South Africa	15	0	
Australia	88	1	Razavi et al. (2014)[41]
Brazil	16	6	
Canada	73	2	
Egypt	18	17	
Switzerland	41	3	
Turkey	18	5	
Adjusted regional values used for remaining countries			
African region - AFRO	6	3	Values calculated such that, in combination with country-specific values above, the global diagnosed and treated percentages
Region of the Americas – PAHO	45	14	
Eastern Mediterranean Region - EMRO	21	0	

European Region - EURO	13	2	match WHO global estimates (see below) as reported in World Hepatitis Report (2017)[111]
South-East Asia Region - SEARO	9	7	
Western Pacific Region - WPRO	16	6	
Global	20	7	World Hepatitis Report (2017)[111]

Countries with treatment cascade estimates reported for 2013 or 2014 had estimates of 2015 treatment cascade values produced using available information in the relevant papers regarding new diagnosis and treatments combined with estimates of new infections and mortality. In the case of India, Pakistan and Egypt, significant treatment expansion was reported in 2015. Treatment numbers were altered by adding 100 000 extra treatments for Egypt,[116] 65 000 for Pakistan,[116] and 42 000 for India[99] when producing updated 2015 percentage treated values; not doing this led to extremely high estimates for the adjusted regional SEARO and EMRO percentage treated values.

Table S4: Number of DAAs in countries with DAAs introduced in 2016

Country	Number of DAA courses	Reference
Argentina	1 200	WHO progress on access report (2018)[116]
Australia	40 000	
Brazil	41 200	
China	133 000	
Egypt	700 000	
France	45 000	
Georgia	21 700	
Indonesia	400	
Mongolia	6 500	
Morocco	6 500	
Pakistan	161 000	
Romania	6 000	
Rwanda	1 000	
South Africa	160	
Spain	72 000	
Ukraine	2 500	
United Kingdom	12 000	

In addition to the above countries, DAAs are implemented in countries noted as introducing DAAs in the WHO progress report[116] or in a recent study of DAA expansion in the European Union but for which precise 2016 figures were not given. The proportion treated in 2015 is assumed to be equal to the 2016 value and these numbers are used for the number of DAA treatment courses in 2016. The countries modelled as offering DAAs in 2016 that are not listed in the above table are: Austria, Belgium, Bulgaria, Canada, Croatia, Czech Republic, Denmark, Estonia, Finland, Germany, Greece, Hungary, Ireland, Italy, Latvia, Lithuania, Luxembourg, Malta, Netherlands, Poland, Portugal, Slovakia, Slovenia, Sweden, Switzerland and USA. As noted above, this prescription results in approximately 1·8 million treatment courses being delivered of which around 86% (1·5 million) are with DAAs.

Table S5: One-way sensitivity analysis on select outcomes upon varying PWID harm reduction programme effectiveness

Outcome	Lower (20%)	Primary (75%)	Upper (90%)
Infections averted by 2030 in intervention II	12·8 million	14·1 million	14·3 million
Additional infections averted by 2030 in intervention IV	780 000	950 000	960 000

Deaths averted by 2050 in intervention II	510 000	570 000	590 000
Treatment courses required by 2030 in intervention IV	52·9 million	51·8 million	51·4 million
Additional treatment courses required by 2050 in intervention IV	14·6 million	12·0 million	11·3 million

Median values shown. Intervention II refers to the general population risk reduction + PWID harm reduction intervention; intervention IV is the comprehensive strategy involving these elements as well as DAA treatment worldwide and screening scale up such that 90% are diagnosed by 2030.

Supplementary Results

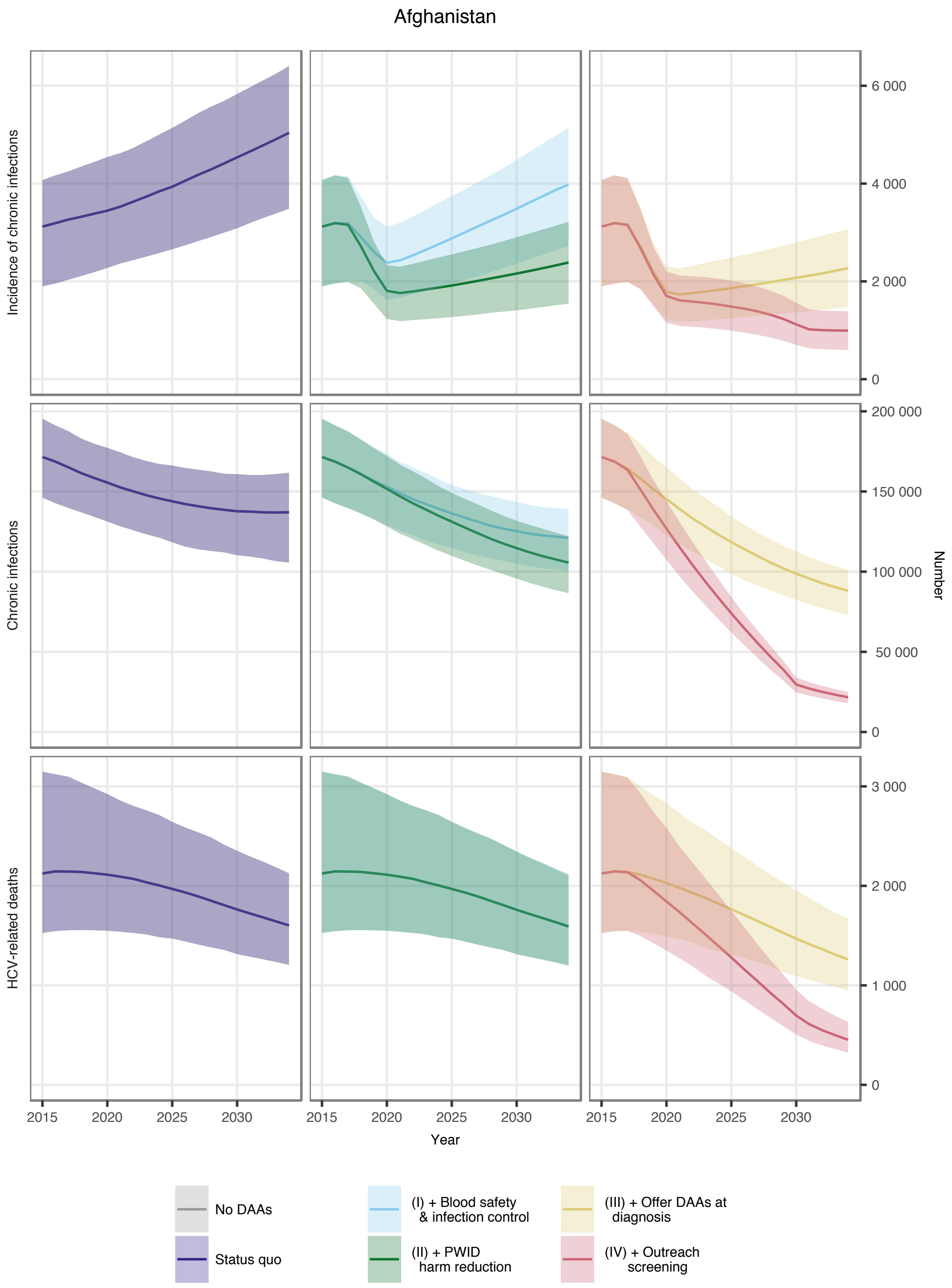

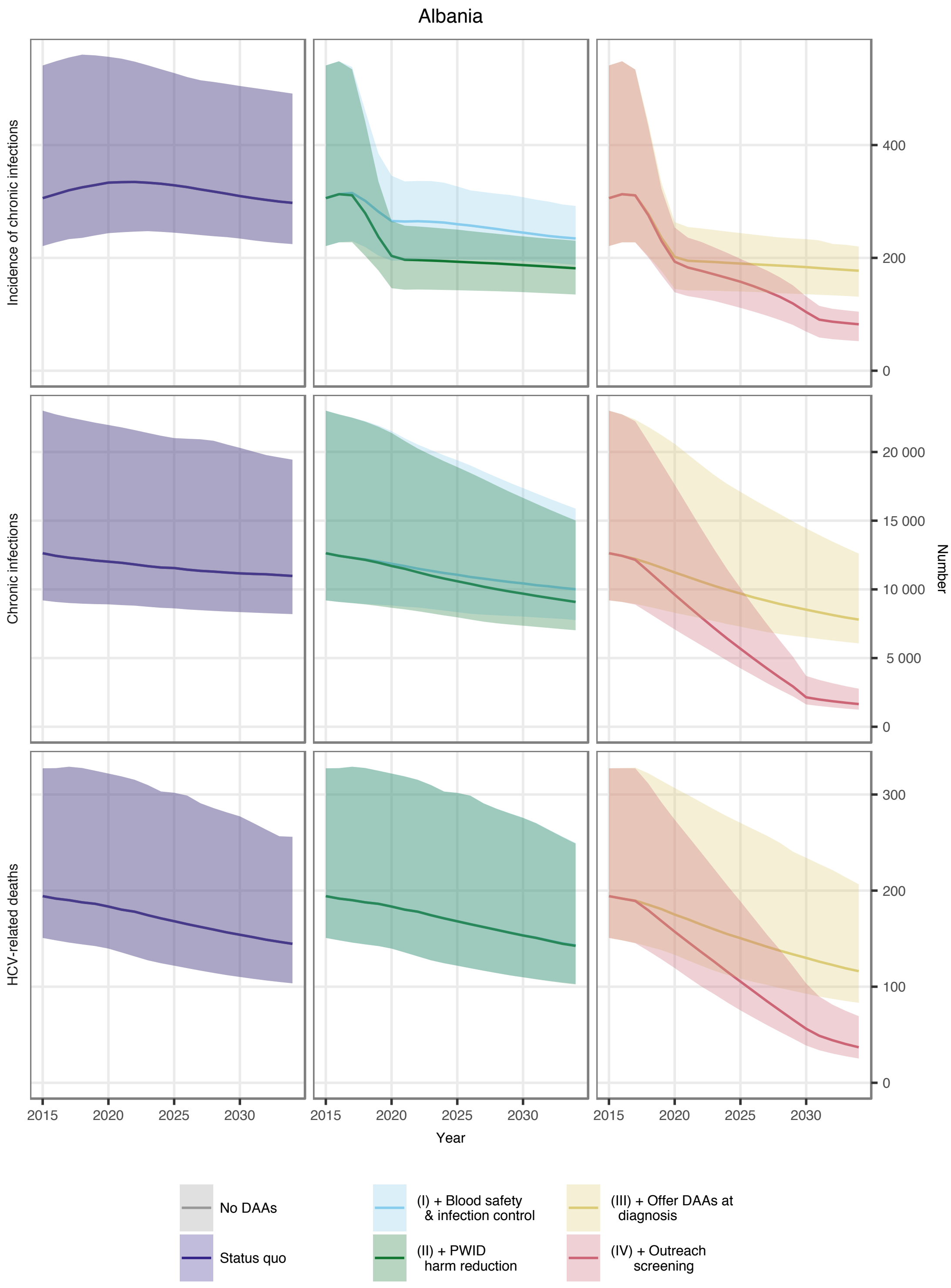

Albania
Incidence of chronic infections
Chronic infections
HCV-related deaths
Number
Year
0
200
400
0
5 000
10 000
15 000
20 000
0
100
200
300
2015
2020
2025
2030
No DAAs
Status quo
(I) + Blood safety & infection control
(II) + PWID harm reduction
(III) + Offer DAAs at diagnosis
(IV) + Outreach screening

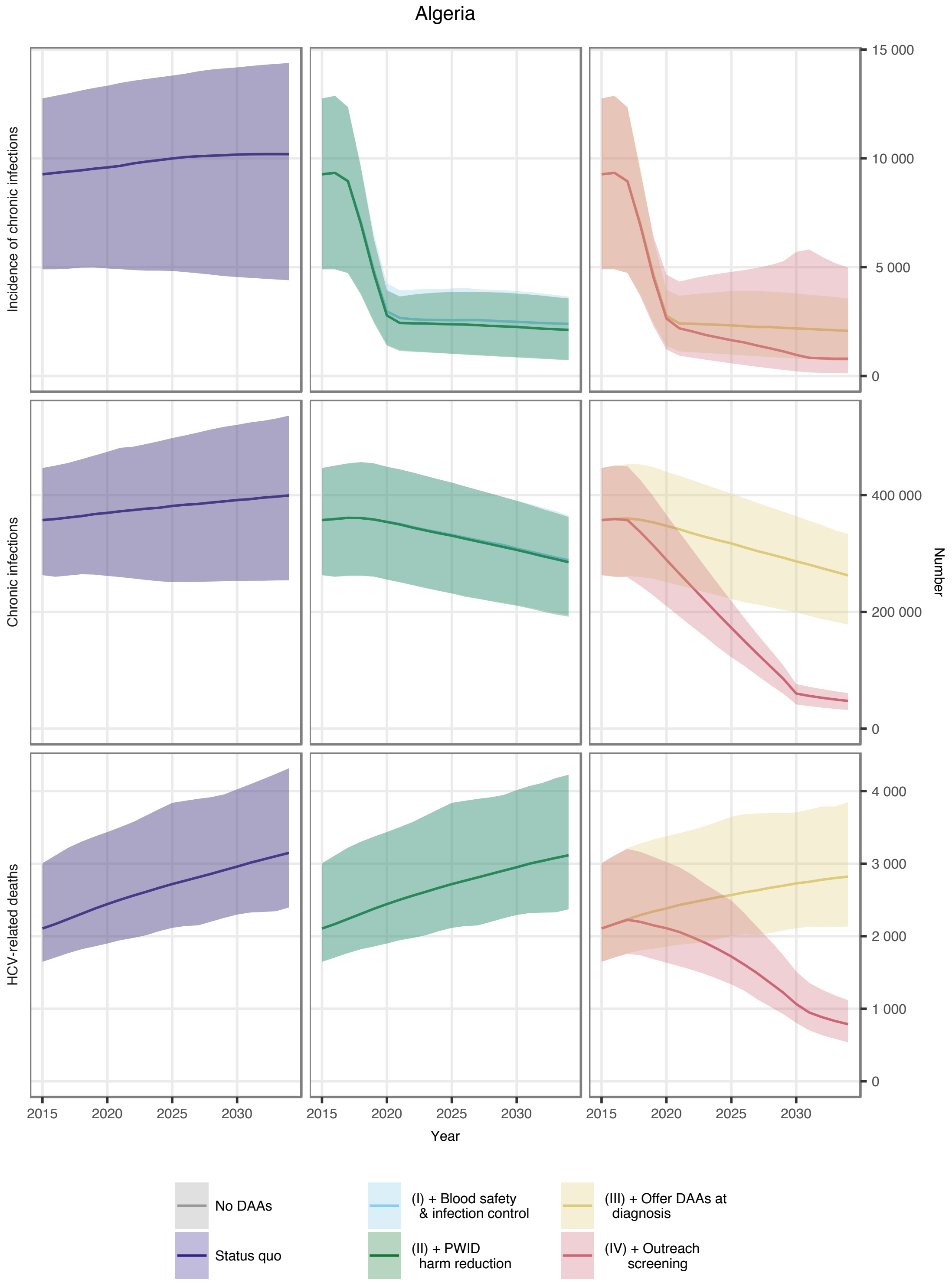
Algeria
Incidence of chronic infections
Chronic infections
HCV-related deaths
15 000
10 000
5 000
0
400 000
200 000
0
4 000
3 000
2 000
1 000
0
Number
2015
2020
2025
2030
Year
No DAAs
Status quo
(I) + Blood safety & infection control
(II) + PWID harm reduction
(III) + Offer DAAs at diagnosis
(IV) + Outreach screening

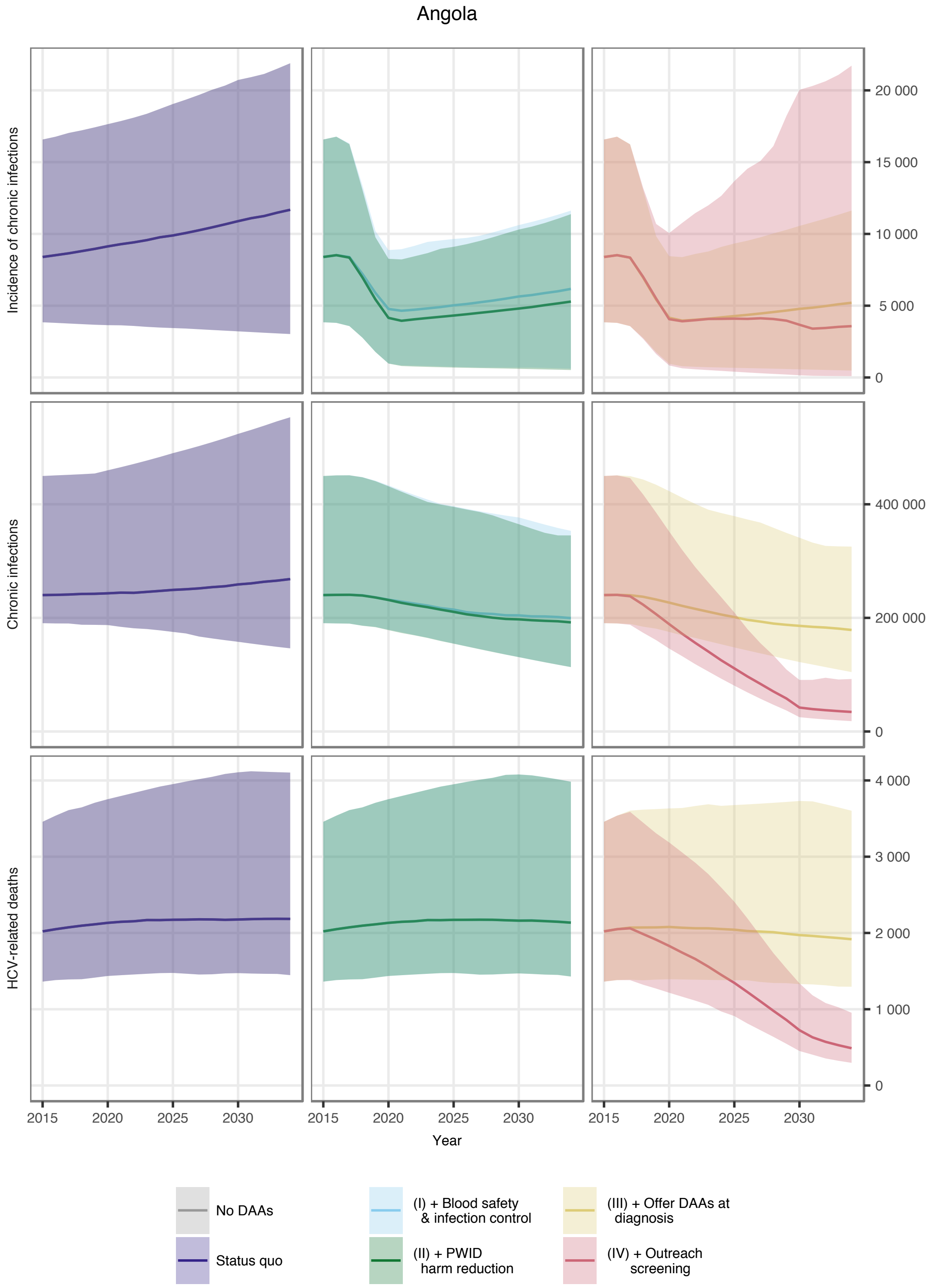

Angola
Incidence of chronic infections
Chronic infections
HCV-related deaths
Number
20 000
15 000
10 000
5 000
0
400 000
200 000
0
4 000
3 000
2 000
1 000
0
2015
2020
2025
2030
Year
No DAAs
Status quo
(I) + Blood safety & infection control
(II) + PWID harm reduction
(III) + Offer DAAs at diagnosis
(IV) + Outreach screening

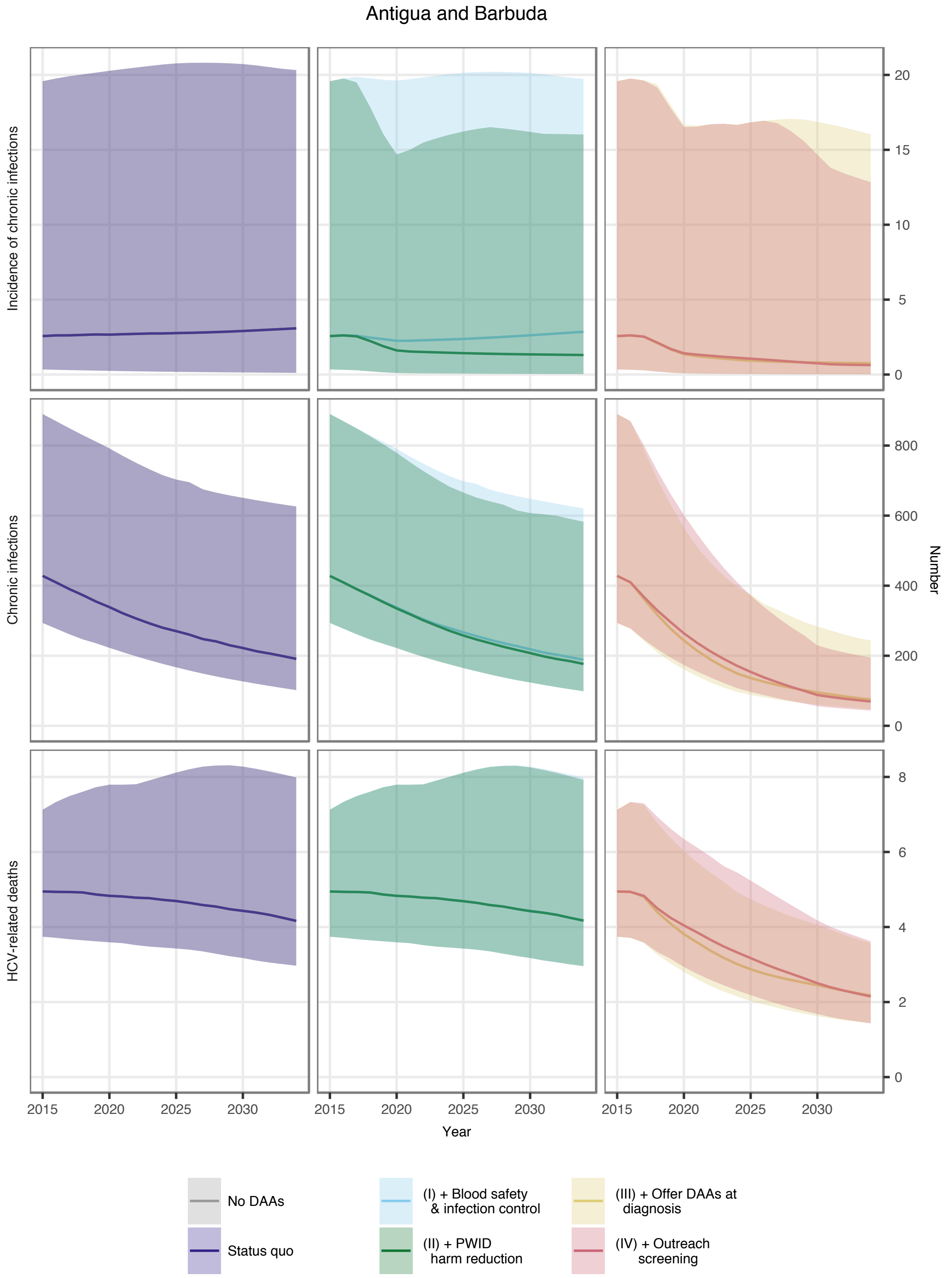
Antigua and Barbuda
Incidence of chronic infections
Chronic infections
HCV-related deaths
Number
Year
0
5
10
15
20
0
200
400
600
800
0
2
4
6
8
2015
2020
2025
2030
No DAAs
Status quo
(I) + Blood safety & infection control
(II) + PWID harm reduction
(III) + Offer DAAs at diagnosis
(IV) + Outreach screening

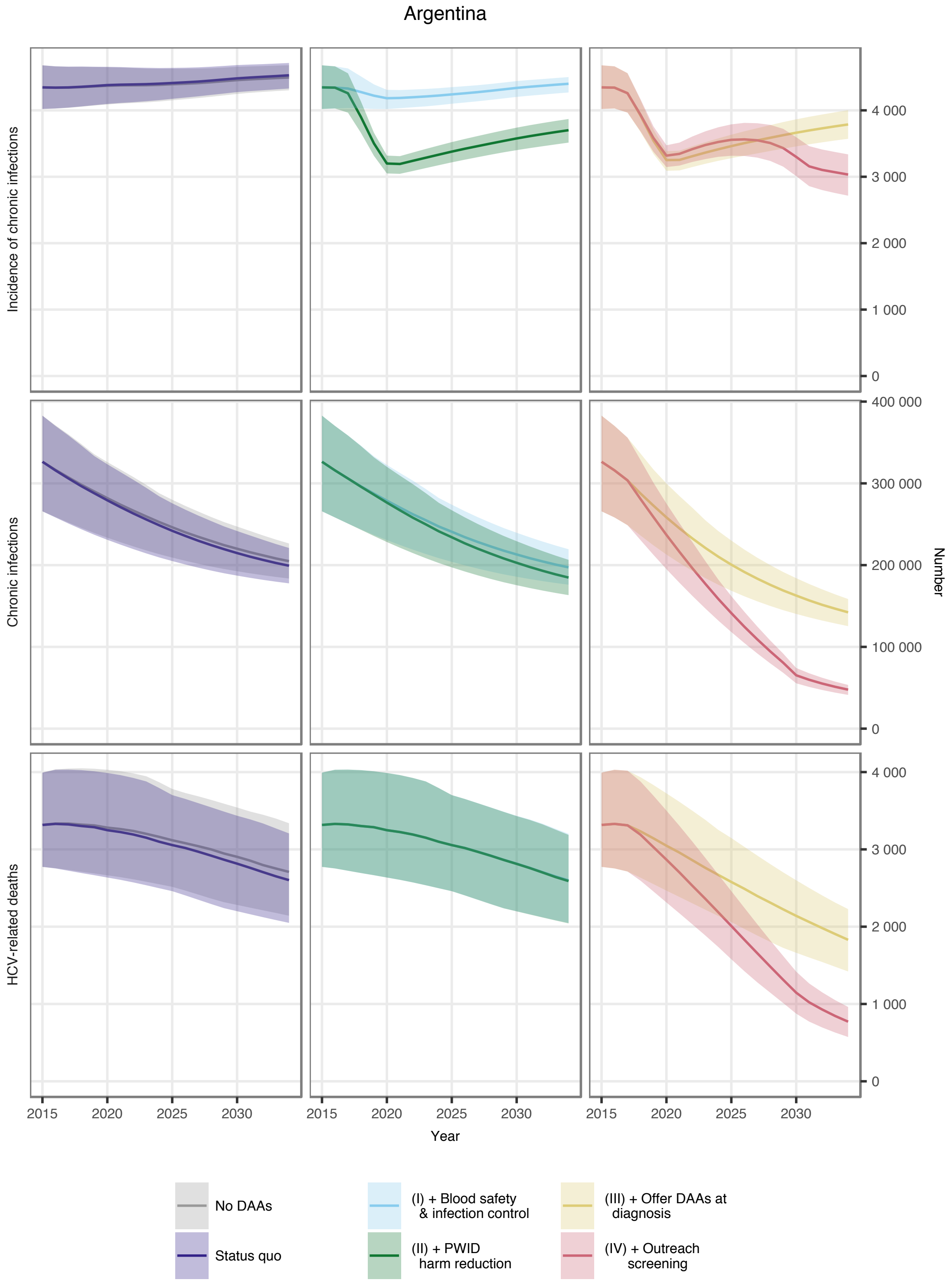
Argentina
Incidence of chronic infections
Chronic infections
HCV-related deaths
Number
Year
2015
2020
2025
2030
0
1 000
2 000
3 000
4 000
100 000
200 000
300 000
400 000
No DAAs
Status quo
(I) + Blood safety & infection control
(II) + PWID harm reduction
(III) + Offer DAAs at diagnosis
(IV) + Outreach screening

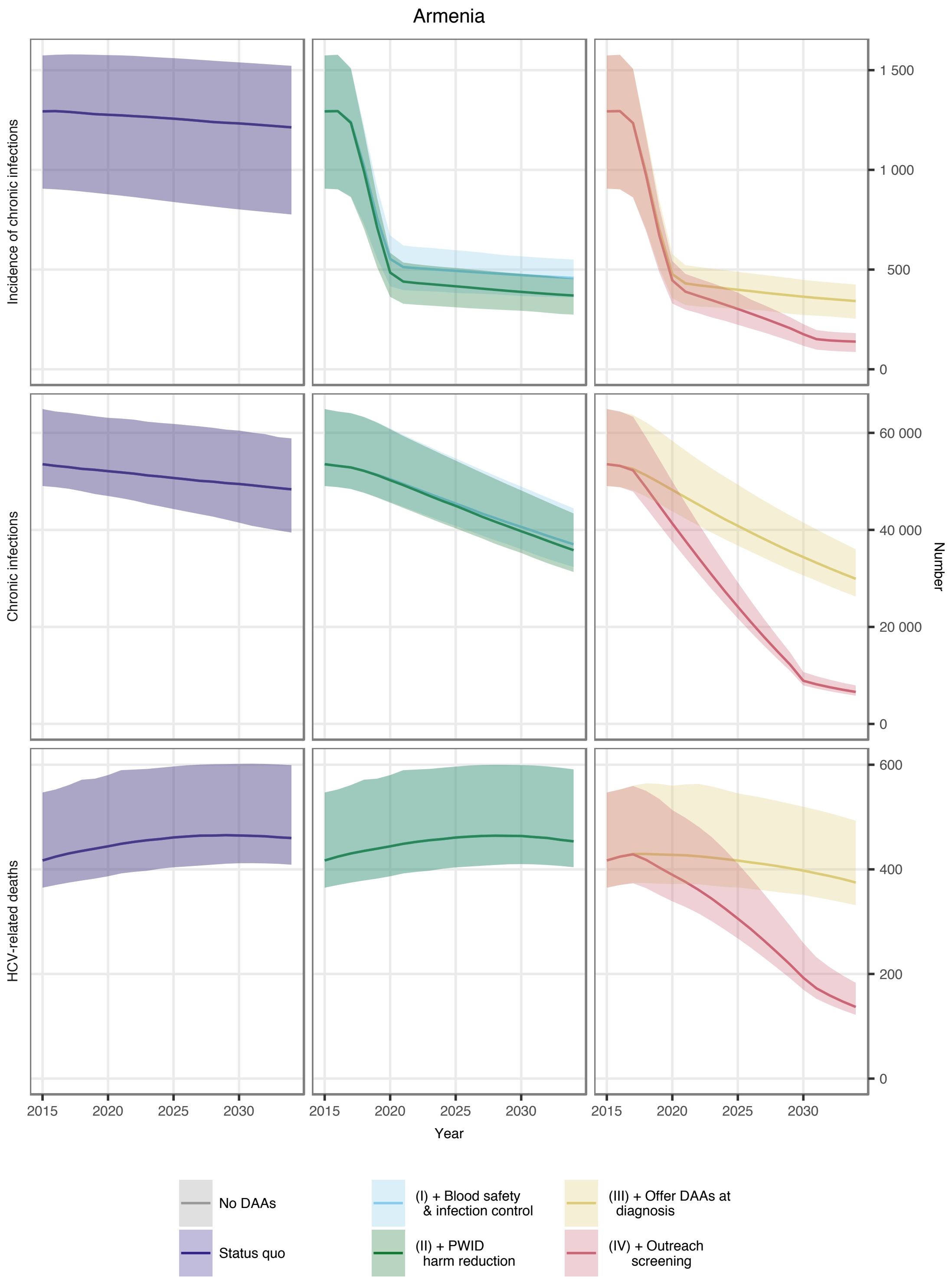
Armenia
Incidence of chronic infections
Chronic infections
HCV-related deaths
Number
1 500
1 000
500
0
60 000
40 000
20 000
0
600
400
200
0
2015
2020
2025
2030
Year
No DAAs
Status quo
(I) + Blood safety & infection control
(II) + PWID harm reduction
(III) + Offer DAAs at diagnosis
(IV) + Outreach screening

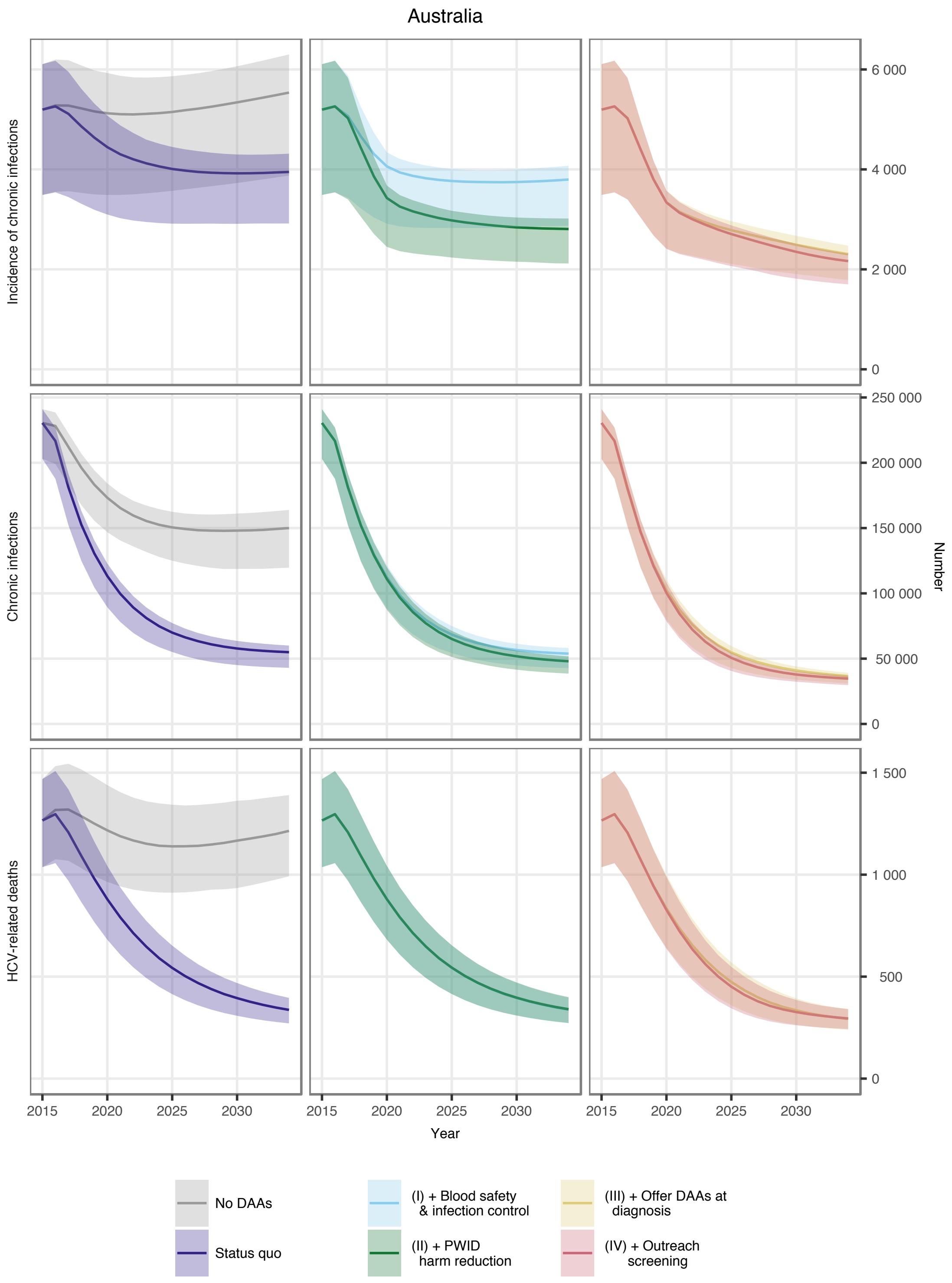

Australia
Incidence of chronic infections
Chronic infections
HCV-related deaths
Number
6 000
4 000
2 000
0
250 000
200 000
150 000
100 000
50 000
0
1 500
1 000
500
0
2015
2020
2025
2030
Year
No DAAs
Status quo
(I) + Blood safety & infection control
(II) + PWID harm reduction
(III) + Offer DAAs at diagnosis
(IV) + Outreach screening

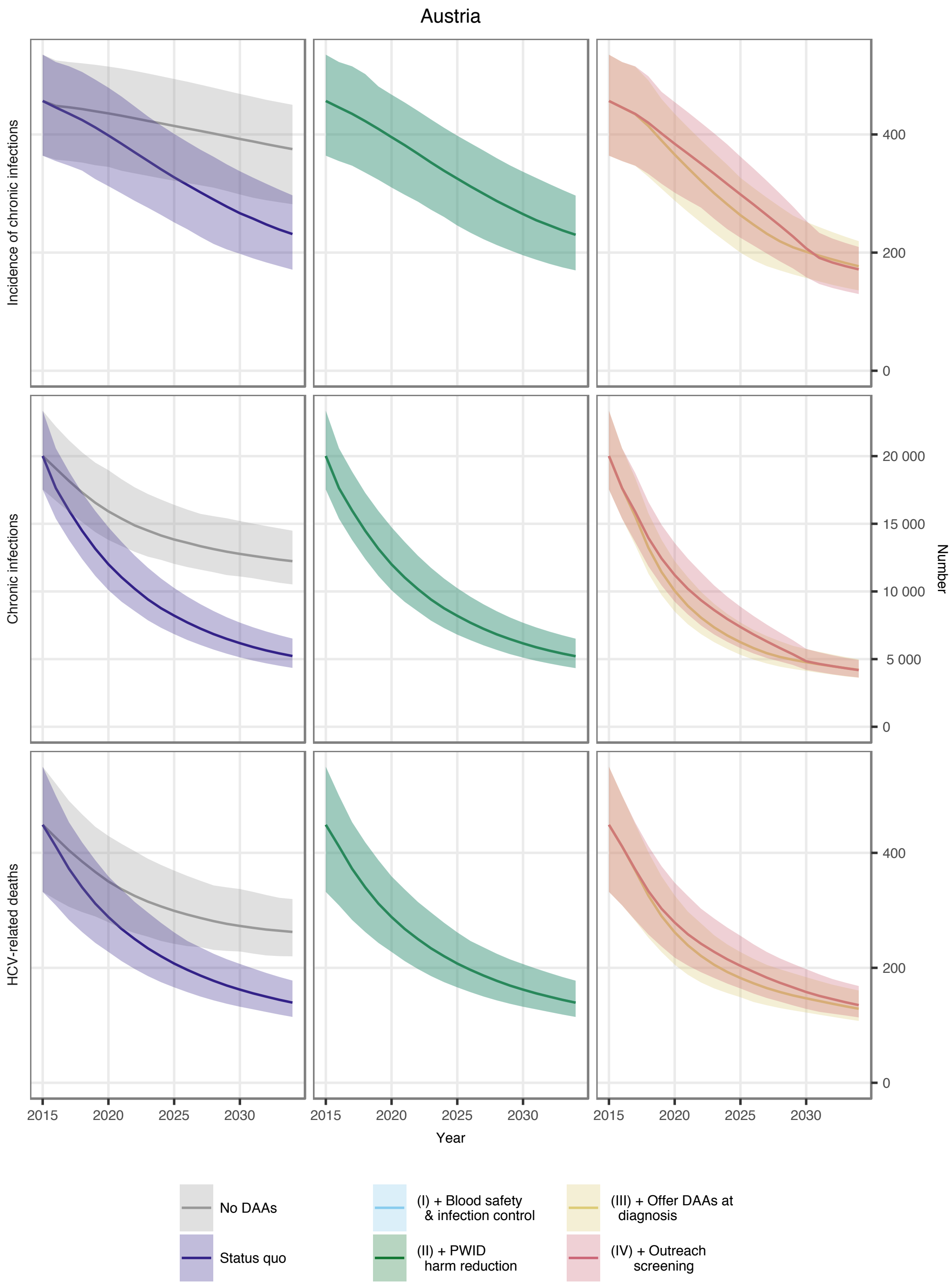

Austria
Incidence of chronic infections
Chronic infections
HCV-related deaths
Number
0
200
400
0
5 000
10 000
15 000
20 000
0
200
400
2015
2020
2025
2030
Year
No DAAs
Status quo
(I) + Blood safety & infection control
(II) + PWID harm reduction
(III) + Offer DAAs at diagnosis
(IV) + Outreach screening

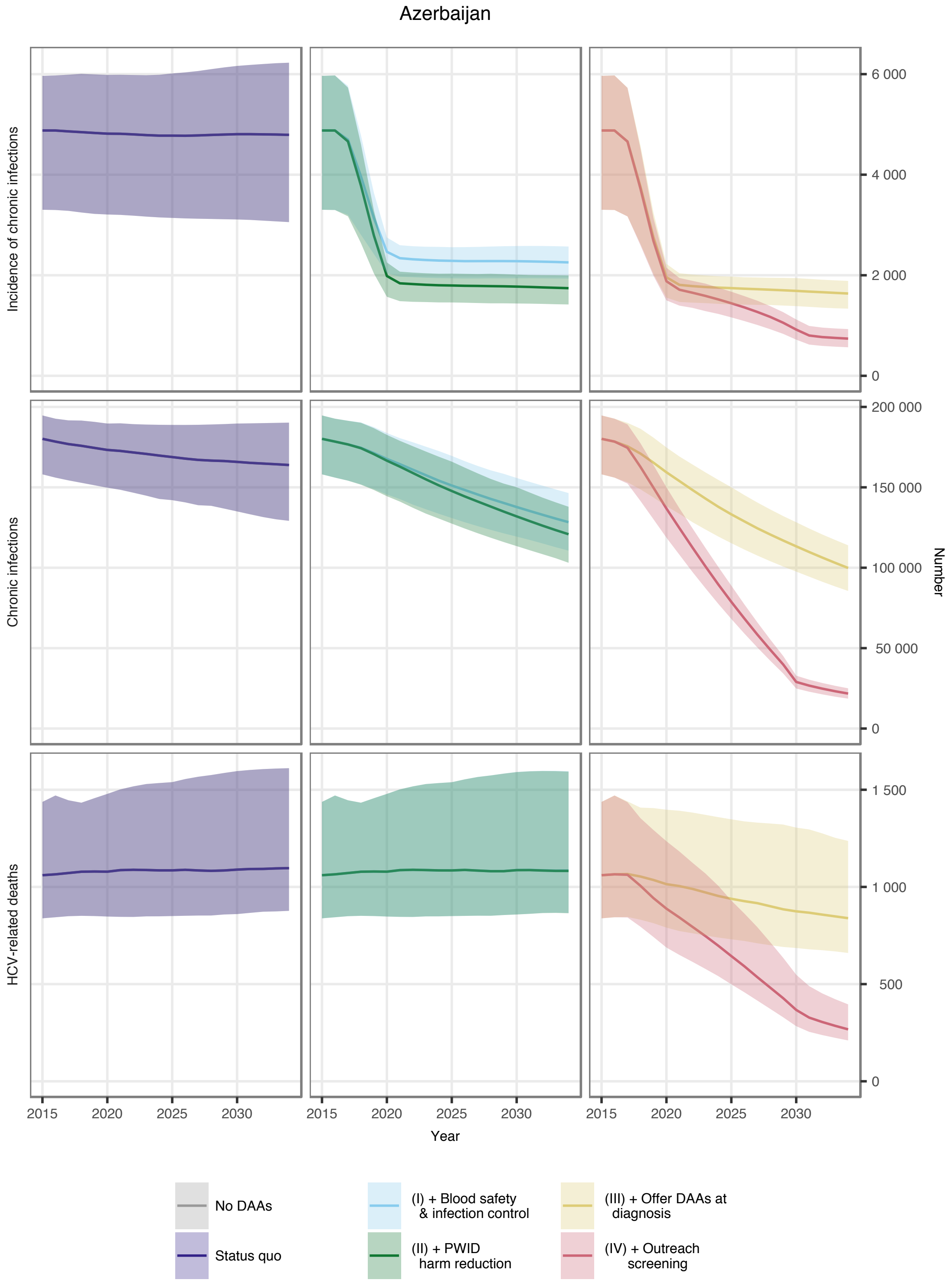

Azerbaijan
Incidence of chronic infections
Chronic infections
HCV-related deaths
6 000
4 000
2 000
0
200 000
150 000
100 000
50 000
0
1 500
1 000
500
0
Number
2015
2020
2025
2030
Year
No DAAs
Status quo
(I) + Blood safety & infection control
(II) + PWID harm reduction
(III) + Offer DAAs at diagnosis
(IV) + Outreach screening

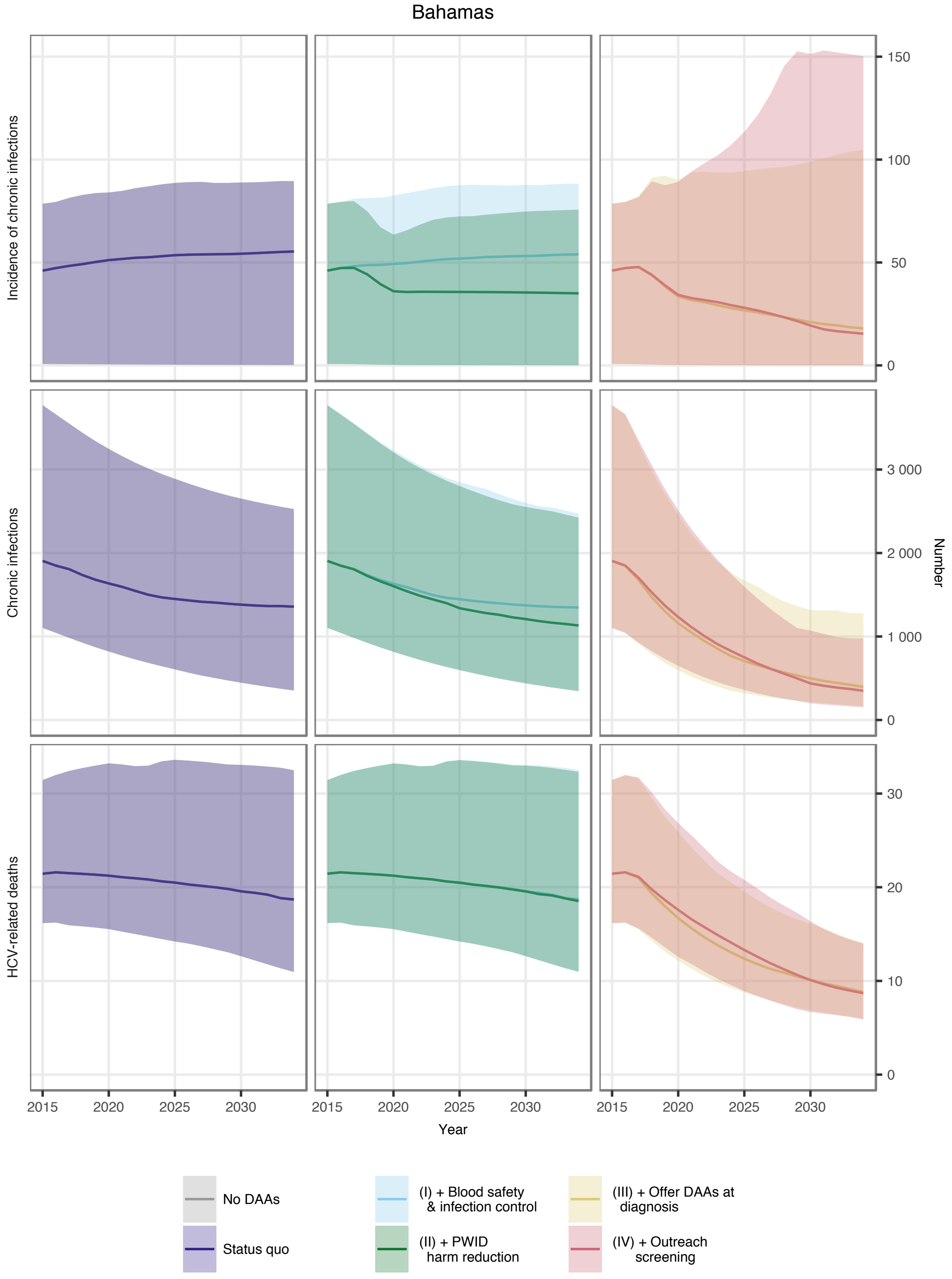
Bahamas
Incidence of chronic infections
Chronic infections
HCV-related deaths
Number
Year
2015
2020
2025
2030
0
50
100
150
0
1 000
2 000
3 000
0
10
20
30
No DAAs
Status quo
(I) + Blood safety & infection control
(II) + PWID harm reduction
(III) + Offer DAAs at diagnosis
(IV) + Outreach screening

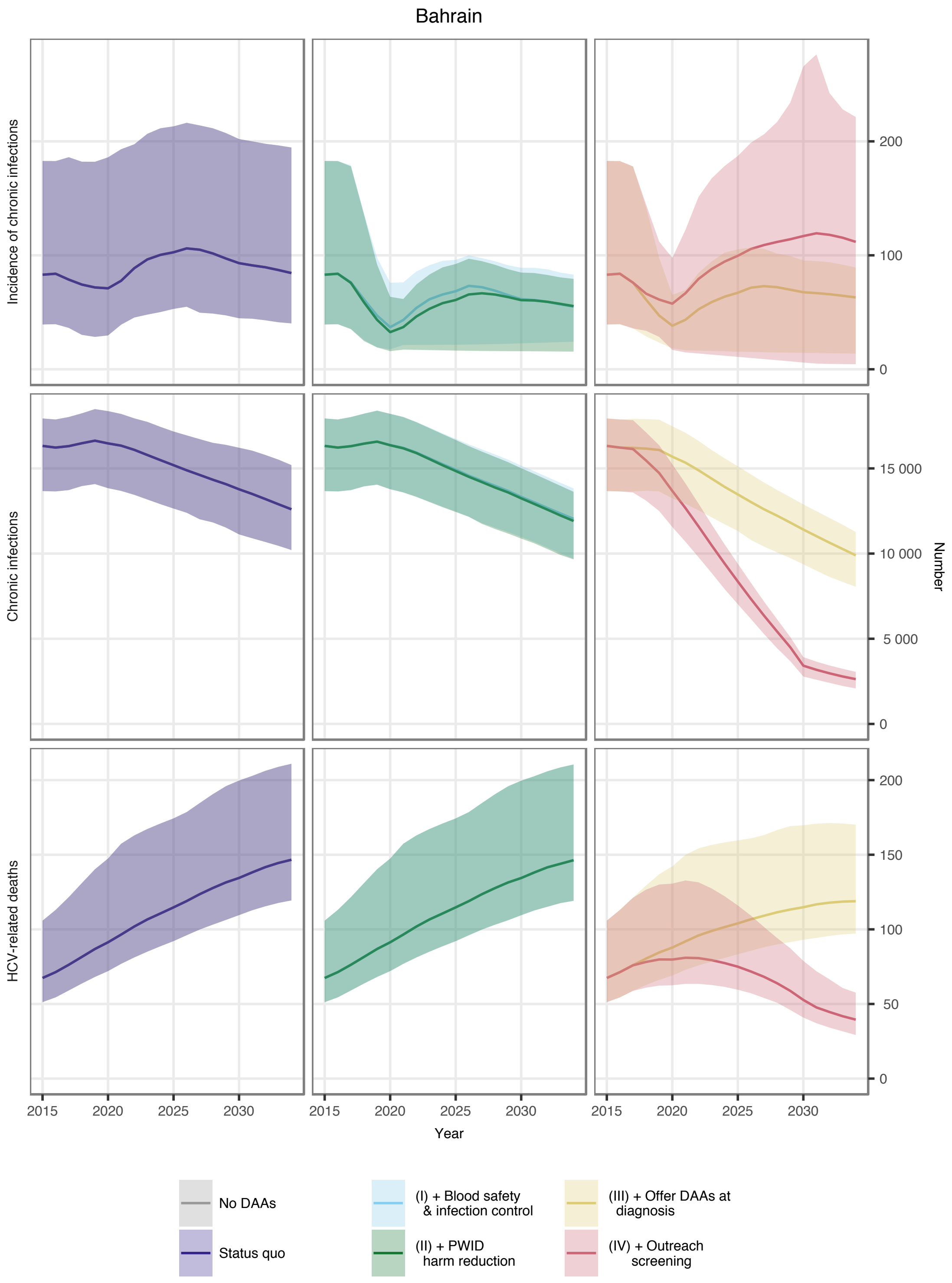
Bahrain
Incidence of chronic infections
Chronic infections
HCV-related deaths
Number
Year
200
100
0
15 000
10 000
5 000
0
200
150
100
50
0
2015
2020
2025
2030
No DAAs
Status quo
(I) + Blood safety & infection control
(II) + PWID harm reduction
(III) + Offer DAAs at diagnosis
(IV) + Outreach screening

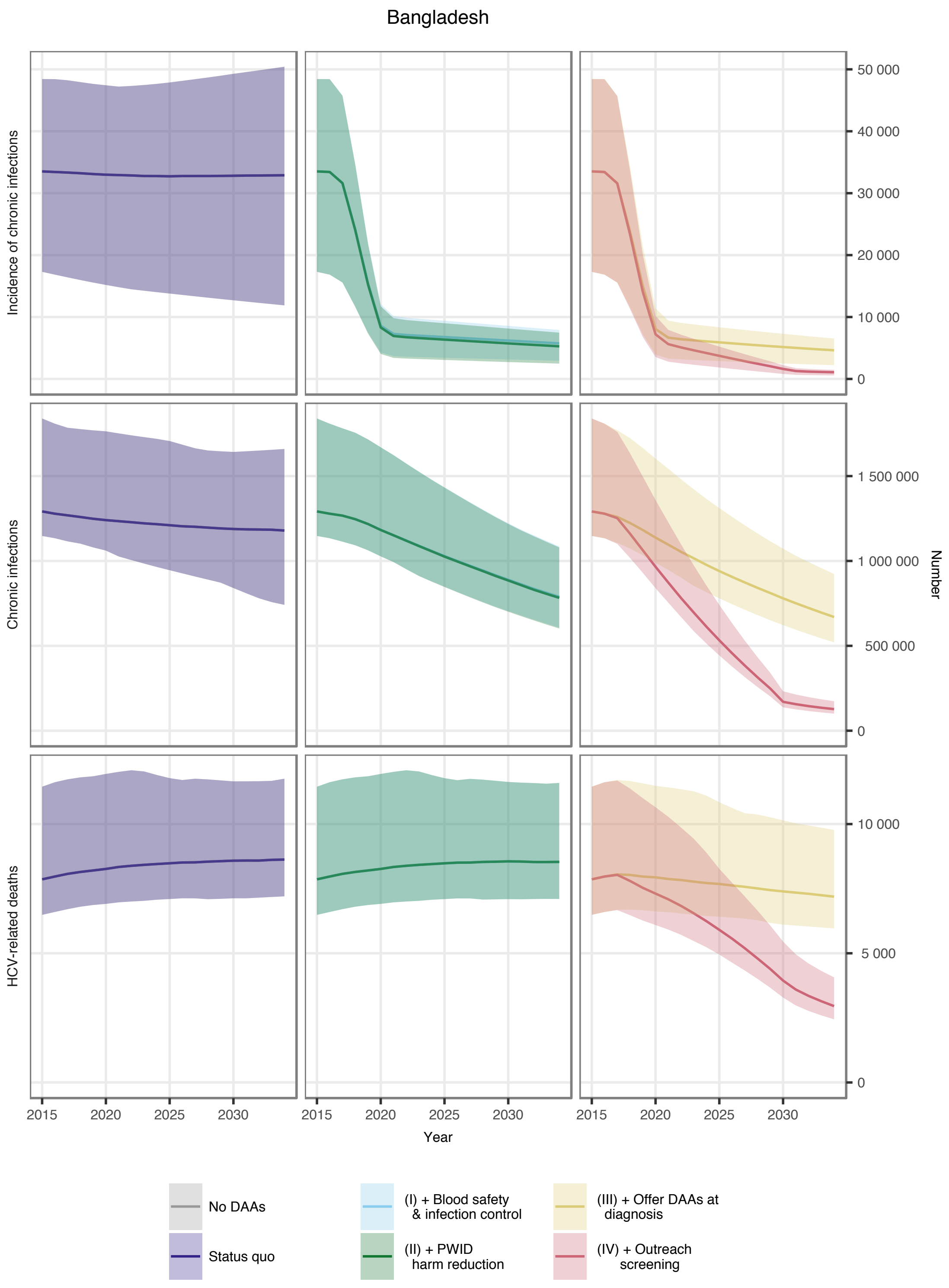

Bangladesh
Incidence of chronic infections
Chronic infections
HCV-related deaths
50 000
40 000
30 000
20 000
10 000
0
1 500 000
1 000 000
500 000
0
10 000
5 000
0
Number
2015
2020
2025
2030
Year
No DAAs
Status quo
(I) + Blood safety & infection control
(II) + PWID harm reduction
(III) + Offer DAAs at diagnosis
(IV) + Outreach screening

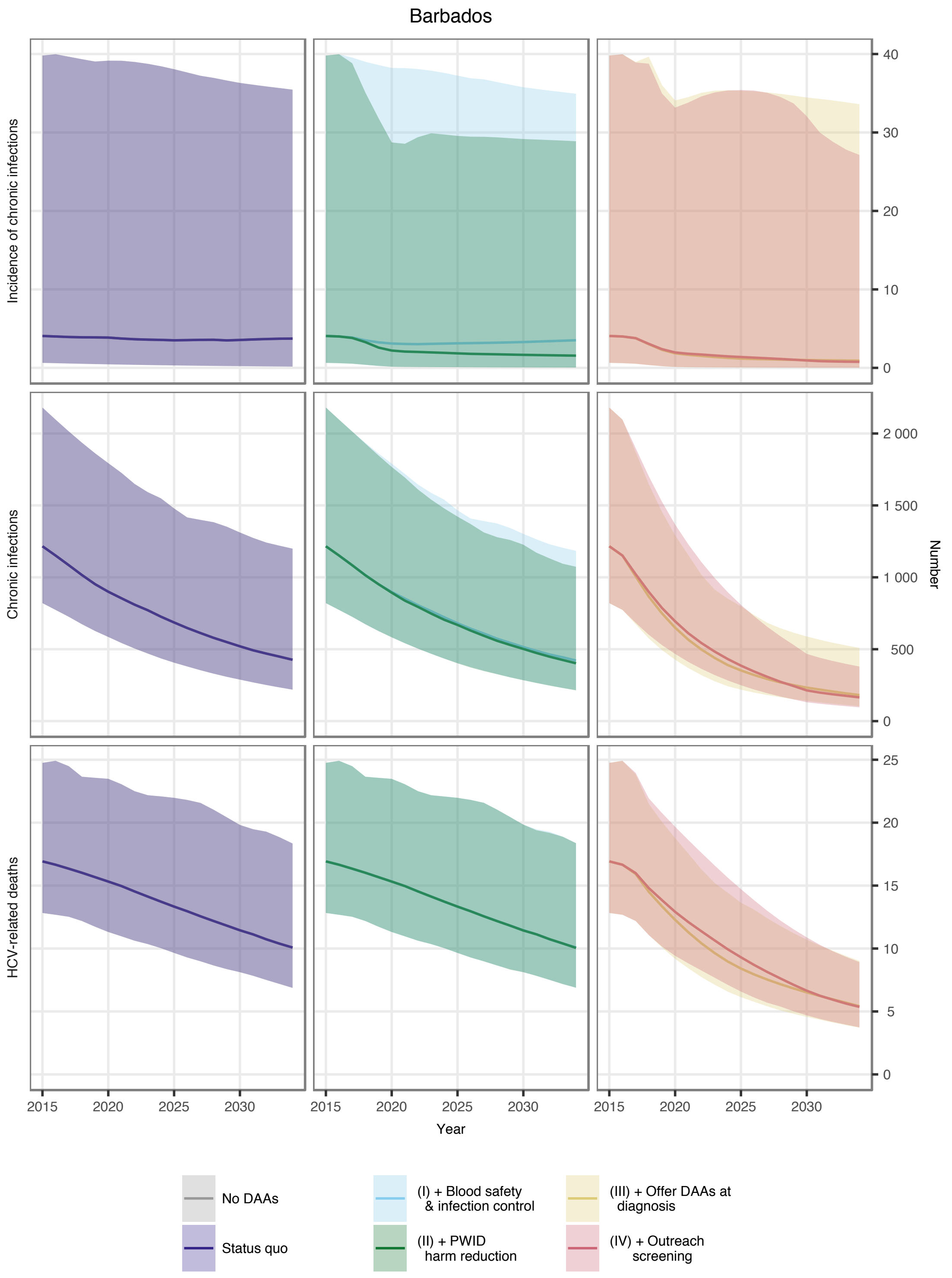

Barbados
Incidence of chronic infections
Chronic infections
HCV-related deaths
Number
Year
2015
2020
2025
2030
40
30
20
10
0
2 000
1 500
1 000
500
25
20
15
5
No DAAs
Status quo
(I) + Blood safety & infection control
(II) + PWID harm reduction
(III) + Offer DAAs at diagnosis
(IV) + Outreach screening

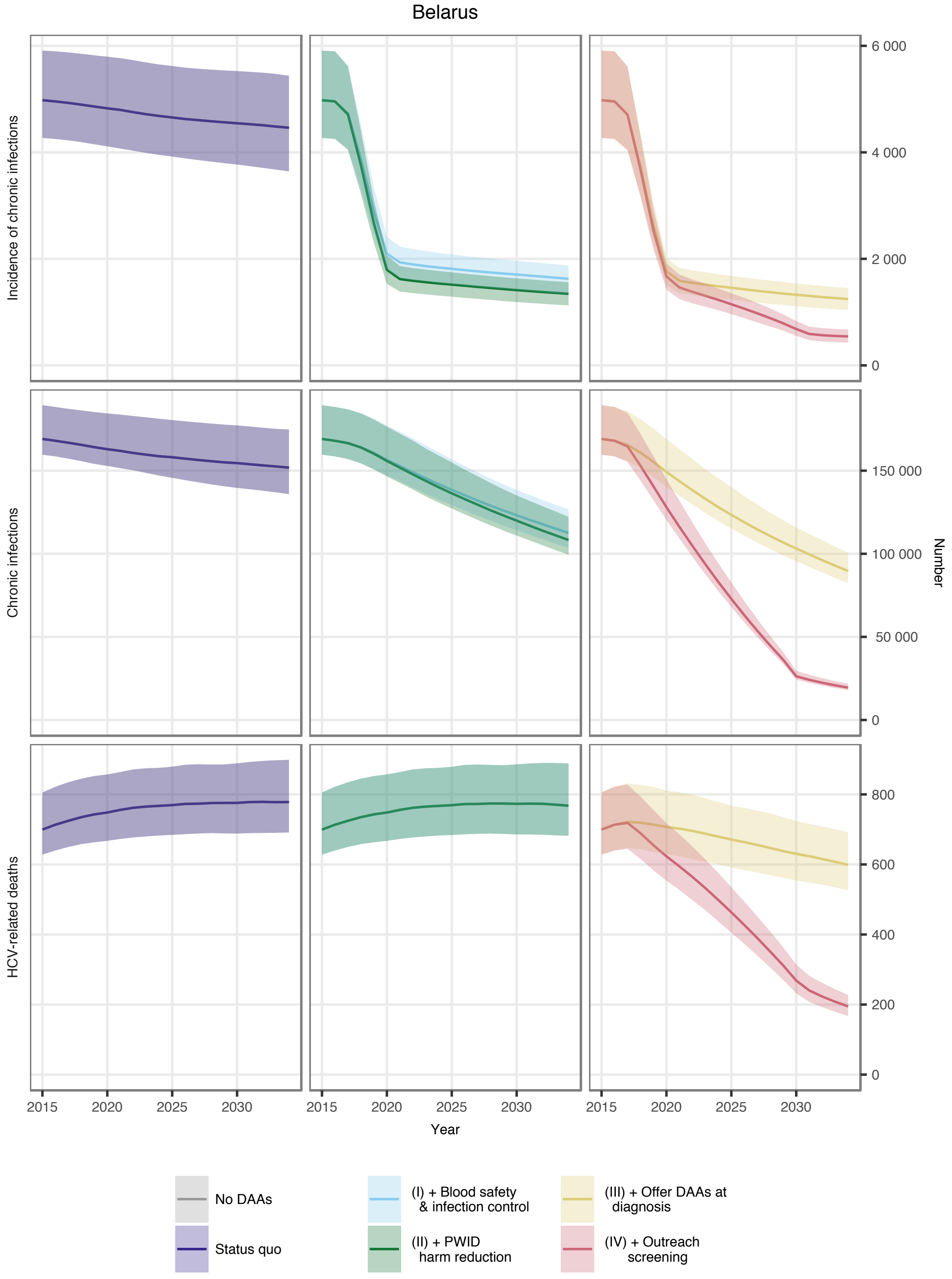
Belarus
Incidence of chronic infections
Chronic infections
HCV-related deaths
Number
6 000
4 000
2 000
0
150 000
100 000
50 000
0
800
600
400
200
0
2015
2020
2025
2030
Year
No DAAs
Status quo
(I) + Blood safety & infection control
(II) + PWID harm reduction
(III) + Offer DAAs at diagnosis
(IV) + Outreach screening

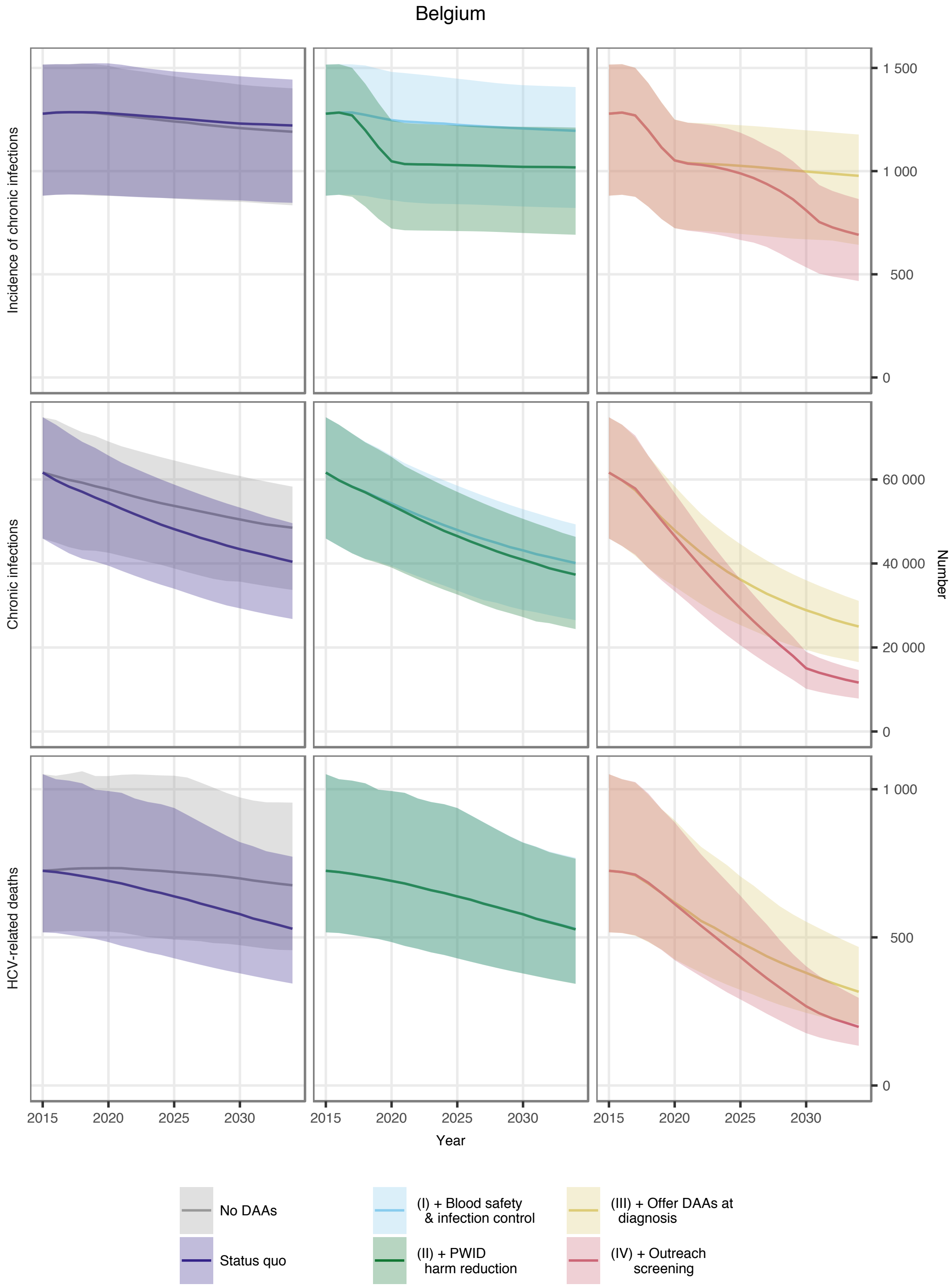

Belgium
Incidence of chronic infections
Chronic infections
HCV-related deaths
Number
Year
1 500
1 000
500
0
60 000
40 000
20 000
0
1 000
500
0
2015
2020
2025
2030
No DAAs
Status quo
(I) + Blood safety & infection control
(II) + PWID harm reduction
(III) + Offer DAAs at diagnosis
(IV) + Outreach screening

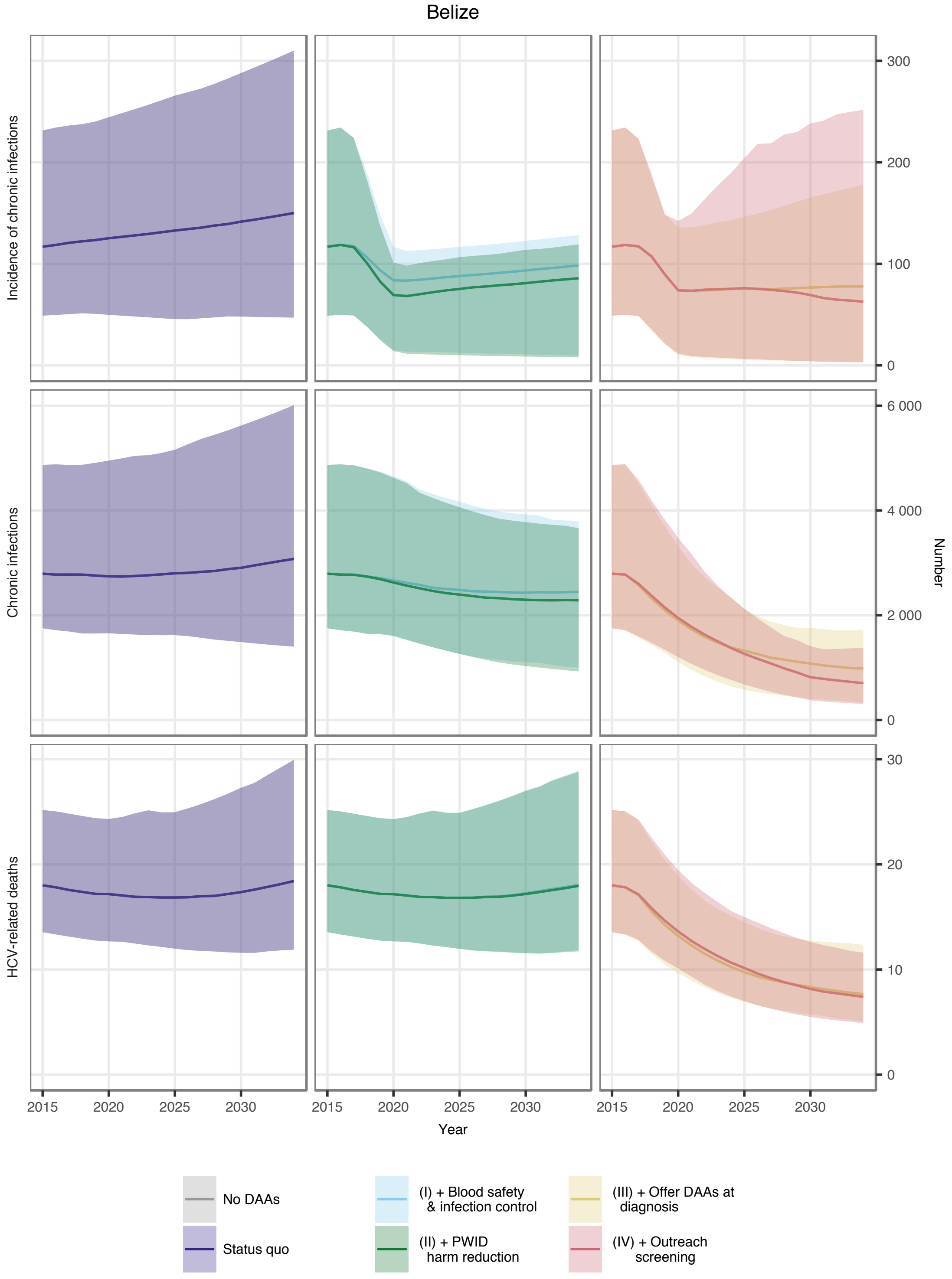
Belize
Incidence of chronic infections
Chronic infections
HCV-related deaths
Number
Year
2015
2020
2025
2030
0
100
200
300
0
2 000
4 000
6 000
0
10
20
30
No DAAs
Status quo
(I) + Blood safety & infection control
(II) + PWID harm reduction
(III) + Offer DAAs at diagnosis
(IV) + Outreach screening

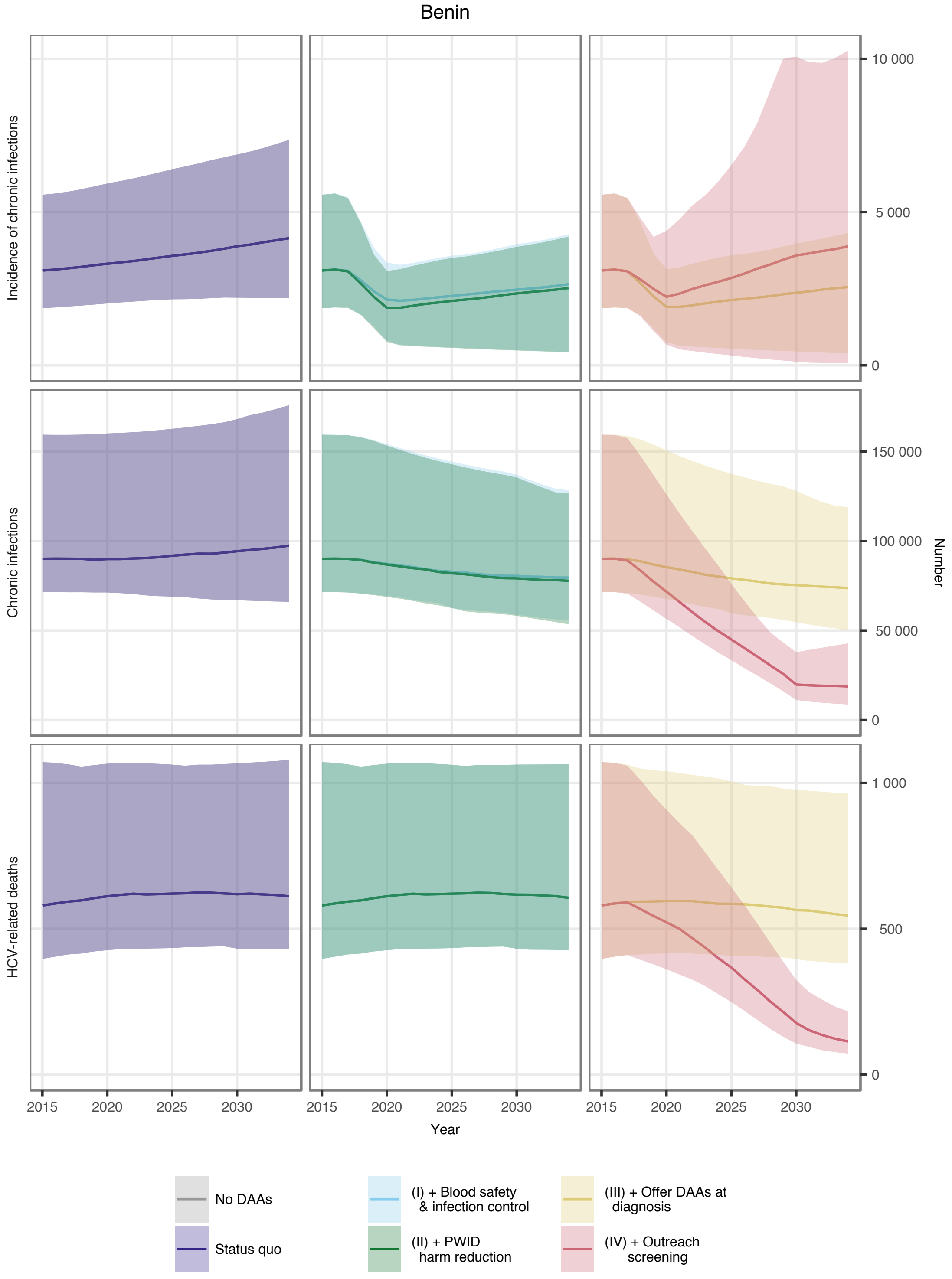

Benin
Incidence of chronic infections
Chronic infections
HCV-related deaths
10 000
5 000
0
150 000
100 000
50 000
0
1 000
500
0
Number
2015
2020
2025
2030
Year
No DAAs
Status quo
(I) + Blood safety & infection control
(II) + PWID harm reduction
(III) + Offer DAAs at diagnosis
(IV) + Outreach screening

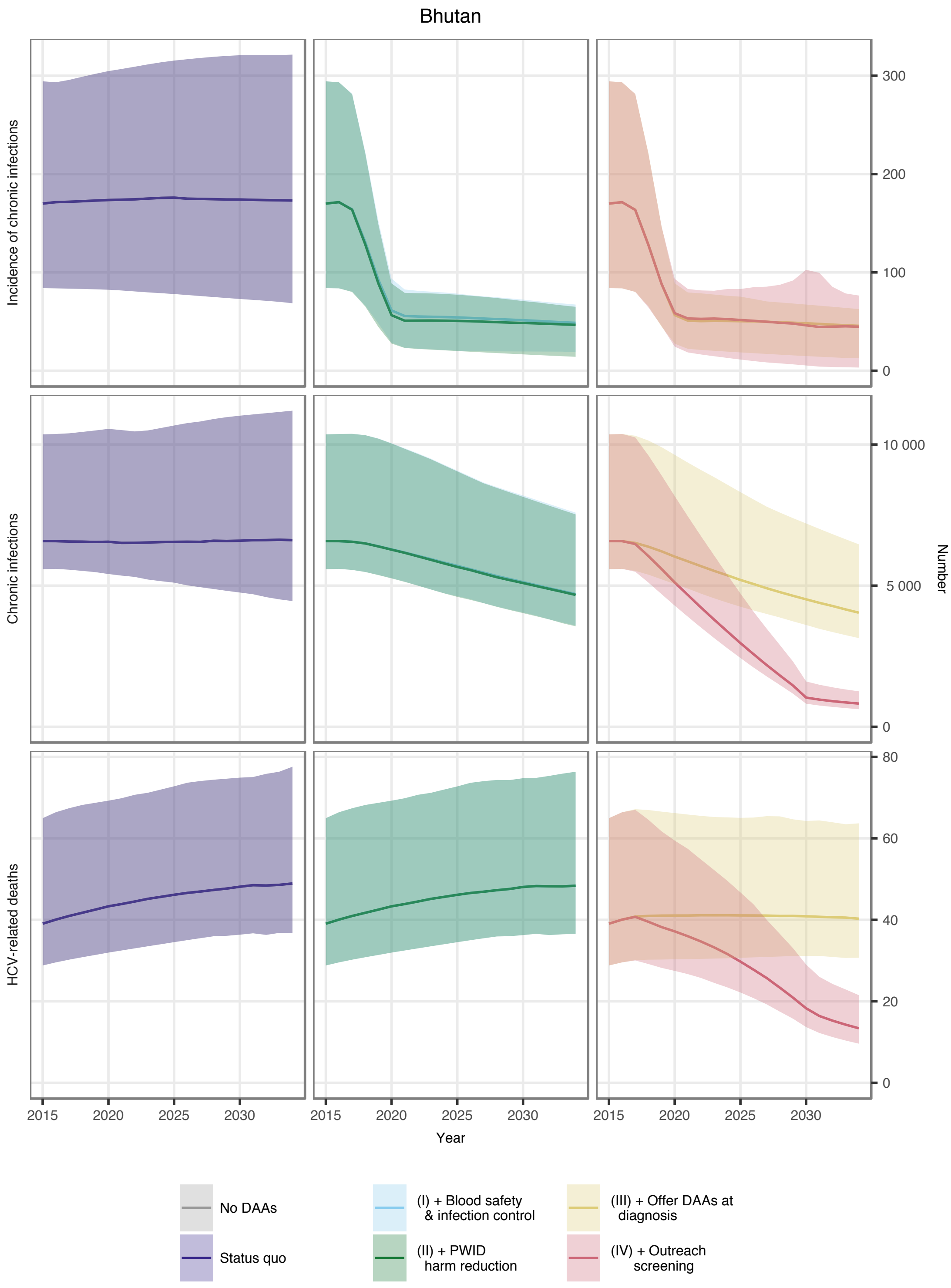

Bhutan
Incidence of chronic infections
Chronic infections
HCV-related deaths
Number
300
200
100
0
10 000
5 000
0
80
60
40
20
0
2015
2020
2025
2030
Year
No DAAs
Status quo
(I) + Blood safety & infection control
(II) + PWID harm reduction
(III) + Offer DAAs at diagnosis
(IV) + Outreach screening

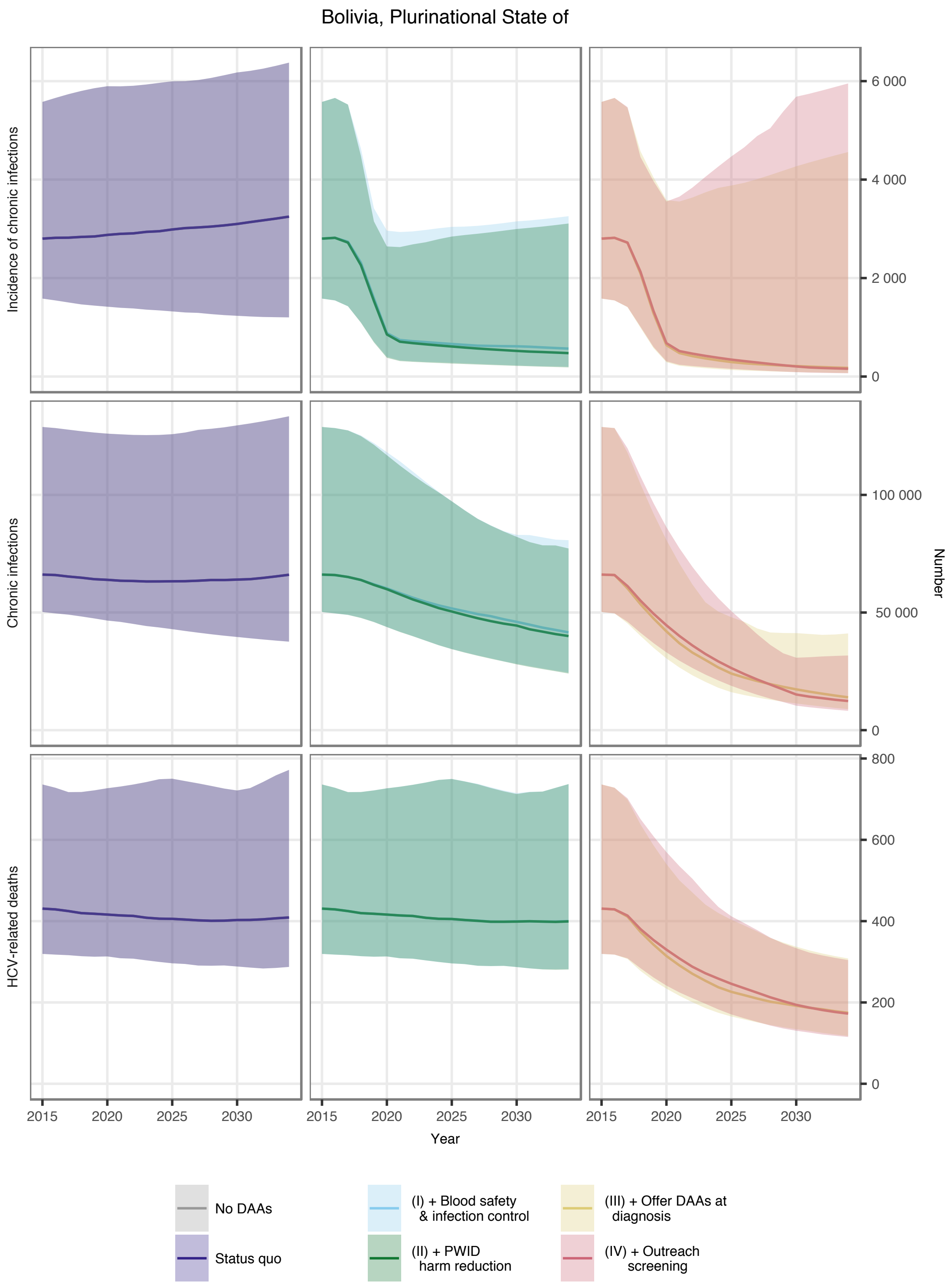
Bolivia, Plurinational State of
Incidence of chronic infections
Chronic infections
HCV-related deaths
Number
Year
6 000
4 000
2 000
0
100 000
50 000
0
800
600
400
200
0
2015
2020
2025
2030
No DAAs
Status quo
(I) + Blood safety & infection control
(II) + PWID harm reduction
(III) + Offer DAAs at diagnosis
(IV) + Outreach screening

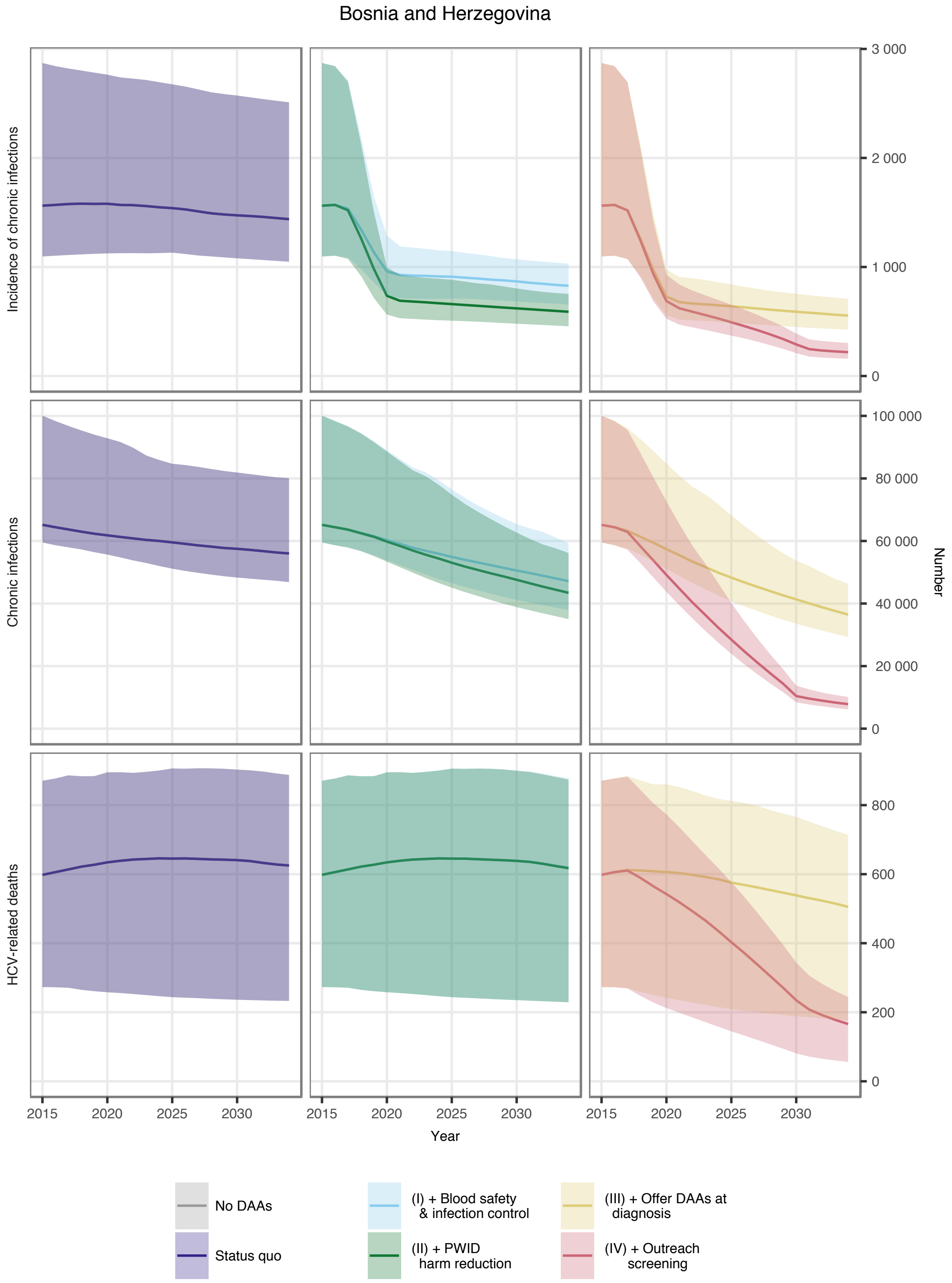
Bosnia and Herzegovina
Incidence of chronic infections
Chronic infections
HCV-related deaths
Number
3 000
2 000
1 000
0
100 000
80 000
60 000
40 000
20 000
0
800
600
400
200
0
2015
2020
2025
2030
Year
No DAAs
Status quo
(I) + Blood safety & infection control
(II) + PWID harm reduction
(III) + Offer DAAs at diagnosis
(IV) + Outreach screening

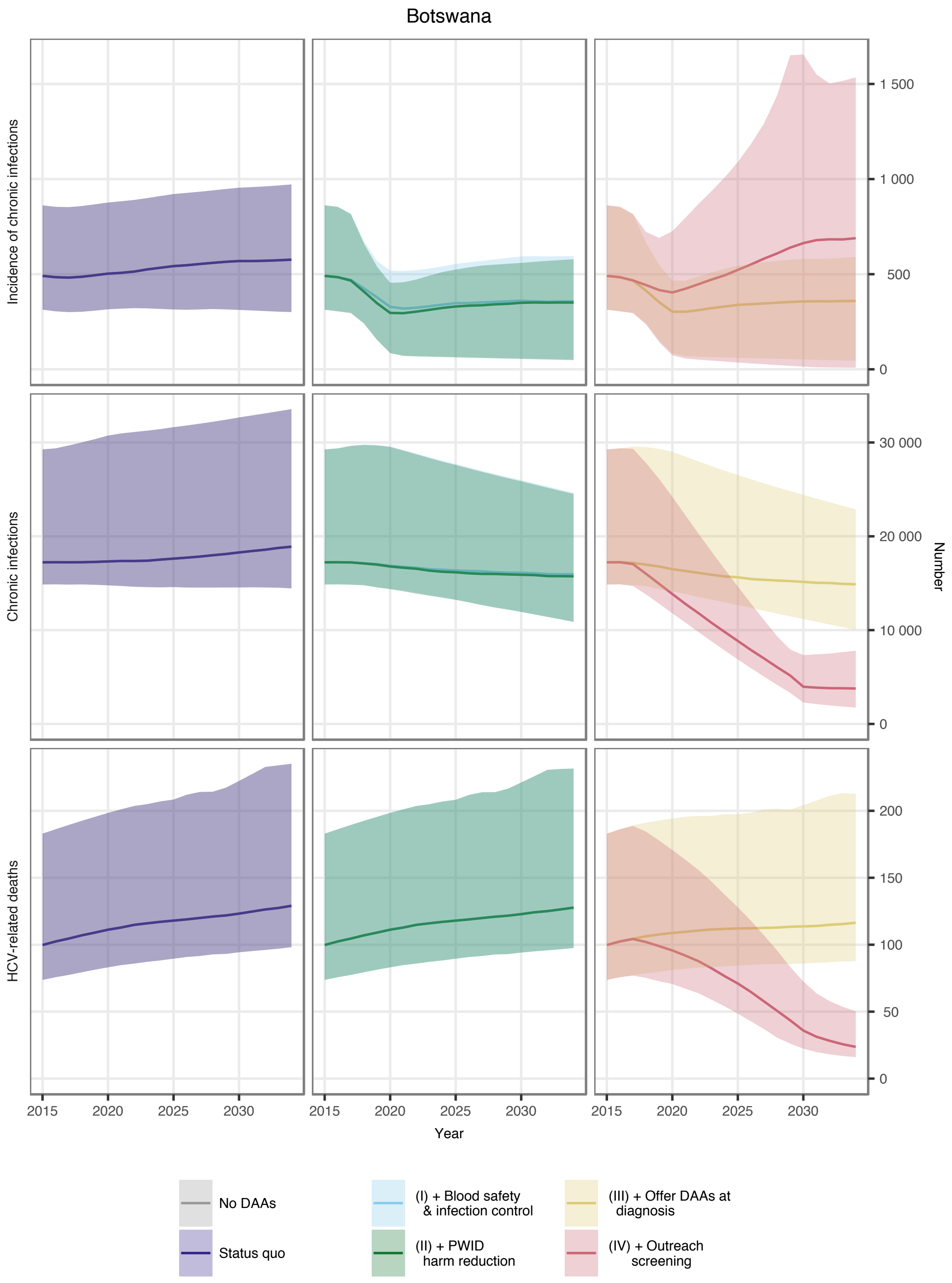
Botswana
Incidence of chronic infections
Chronic infections
HCV-related deaths
Number
Year
2015
2020
2025
2030
0
500
1 000
1 500
10 000
20 000
30 000
50
100
150
200
No DAAs
Status quo
(I) + Blood safety & infection control
(II) + PWID harm reduction
(III) + Offer DAAs at diagnosis
(IV) + Outreach screening

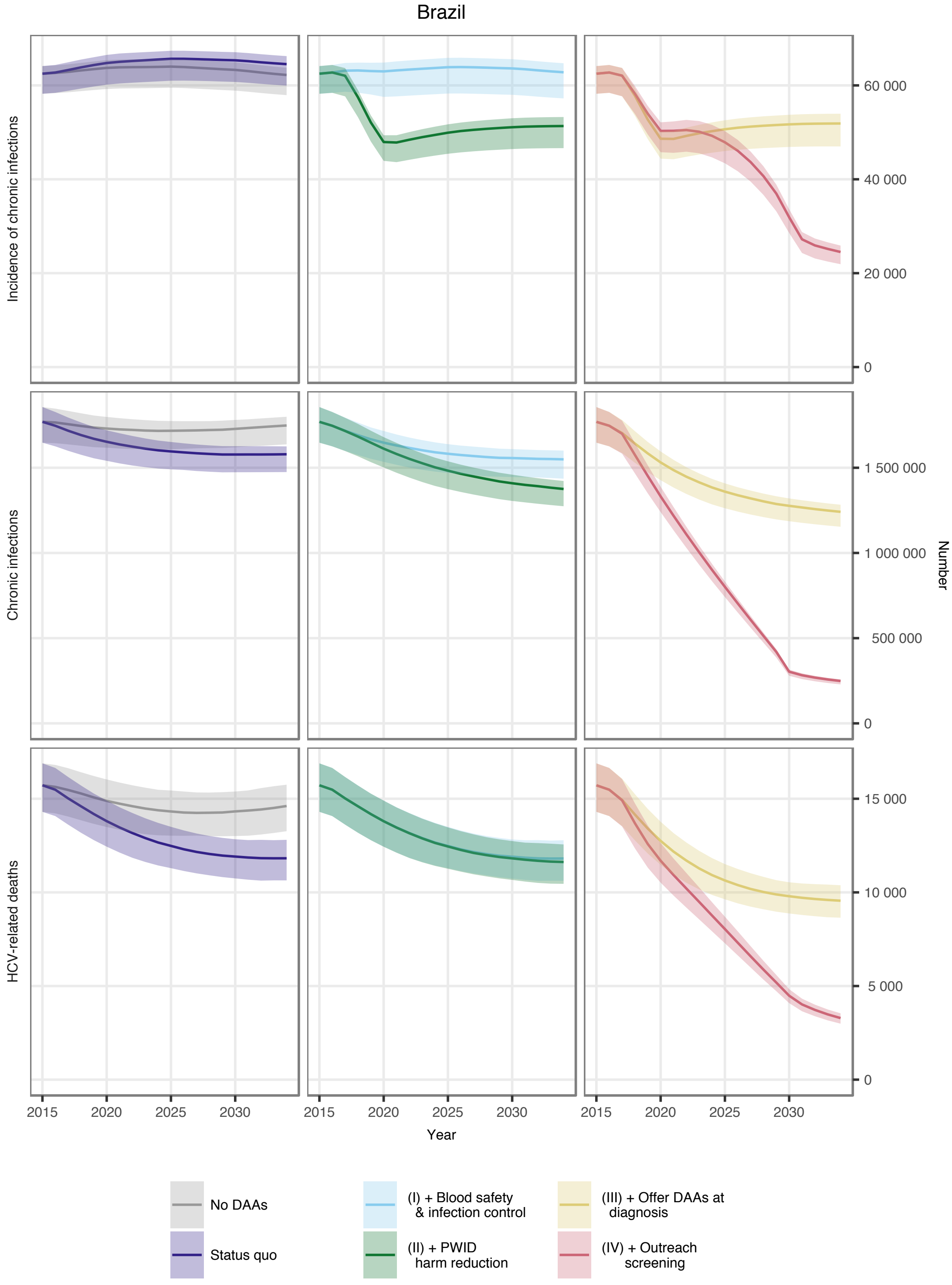
Brazil
Incidence of chronic infections
Chronic infections
HCV-related deaths
Number
60 000
40 000
20 000
0
1 500 000
1 000 000
500 000
0
15 000
10 000
5 000
0
2015
2020
2025
2030
Year
No DAAs
Status quo
(I) + Blood safety & infection control
(II) + PWID harm reduction
(III) + Offer DAAs at diagnosis
(IV) + Outreach screening

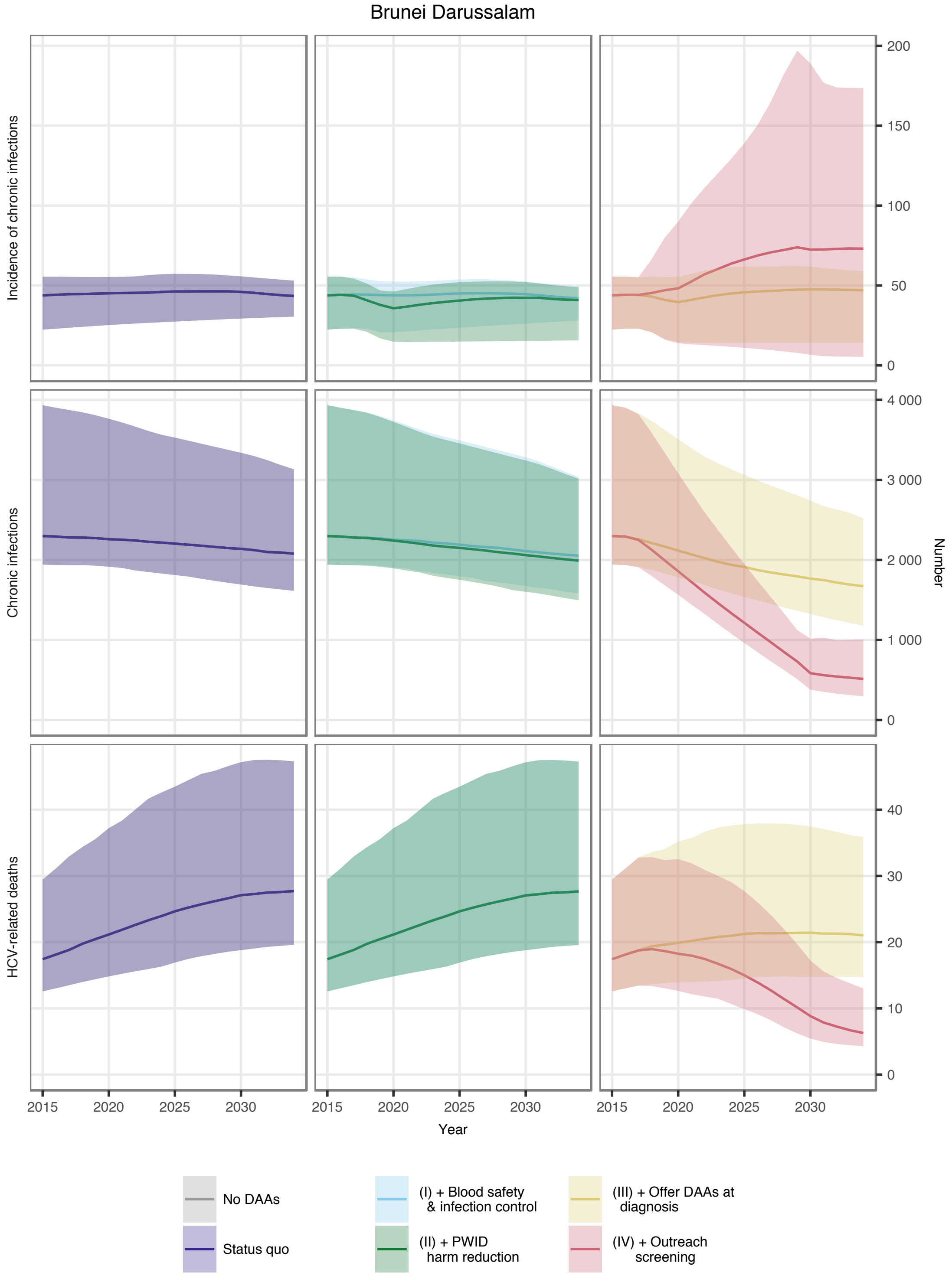
Brunei Darussalam
Incidence of chronic infections
Chronic infections
HCV-related deaths
Number
Year
0
50
100
150
200
0
1 000
2 000
3 000
4 000
0
10
20
30
40
2015
2020
2025
2030
No DAAs
Status quo
(I) + Blood safety & infection control
(II) + PWID harm reduction
(III) + Offer DAAs at diagnosis
(IV) + Outreach screening

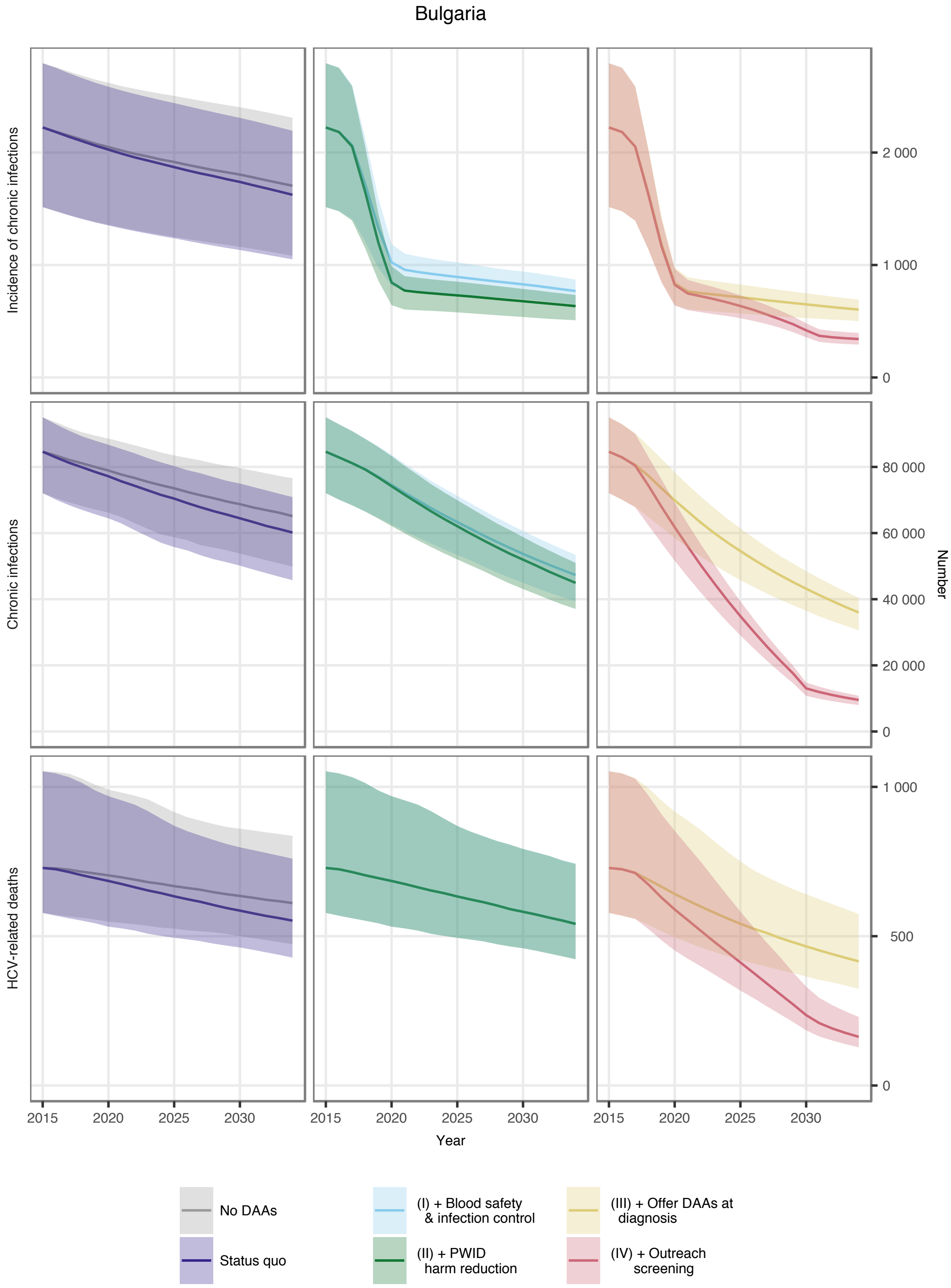
Bulgaria
Incidence of chronic infections
Chronic infections
HCV-related deaths
Number
2 000
1 000
0
80 000
60 000
40 000
20 000
0
1 000
500
0
2015
2020
2025
2030
Year
No DAAs
Status quo
(I) + Blood safety & infection control
(II) + PWID harm reduction
(III) + Offer DAAs at diagnosis
(IV) + Outreach screening

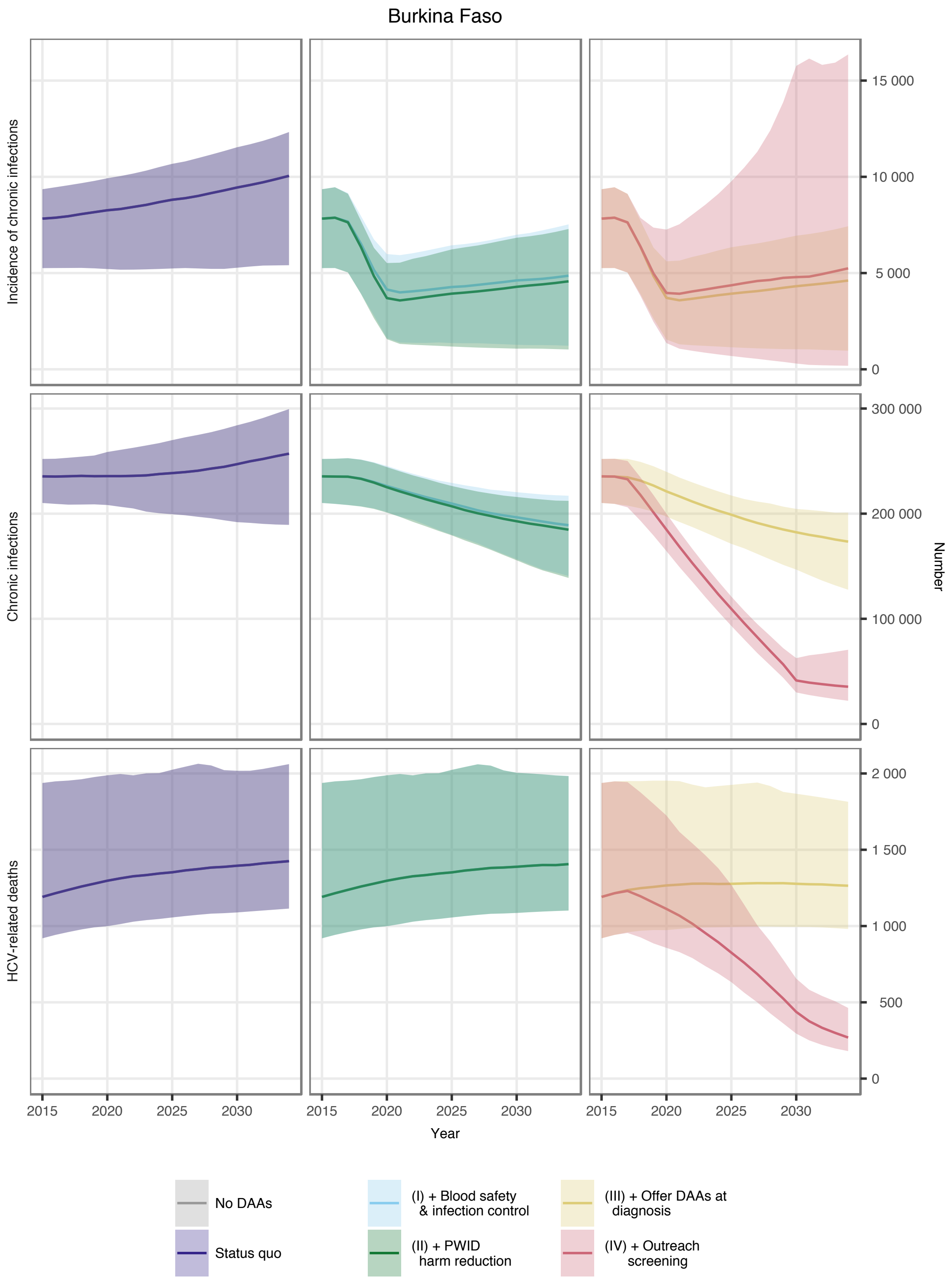
Burkina Faso
Incidence of chronic infections
Chronic infections
HCV-related deaths
Number
15 000
10 000
5 000
0
300 000
200 000
100 000
0
2 000
1 500
1 000
500
0
2015
2020
2025
2030
Year
No DAAs
Status quo
(I) + Blood safety & infection control
(II) + PWID harm reduction
(III) + Offer DAAs at diagnosis
(IV) + Outreach screening

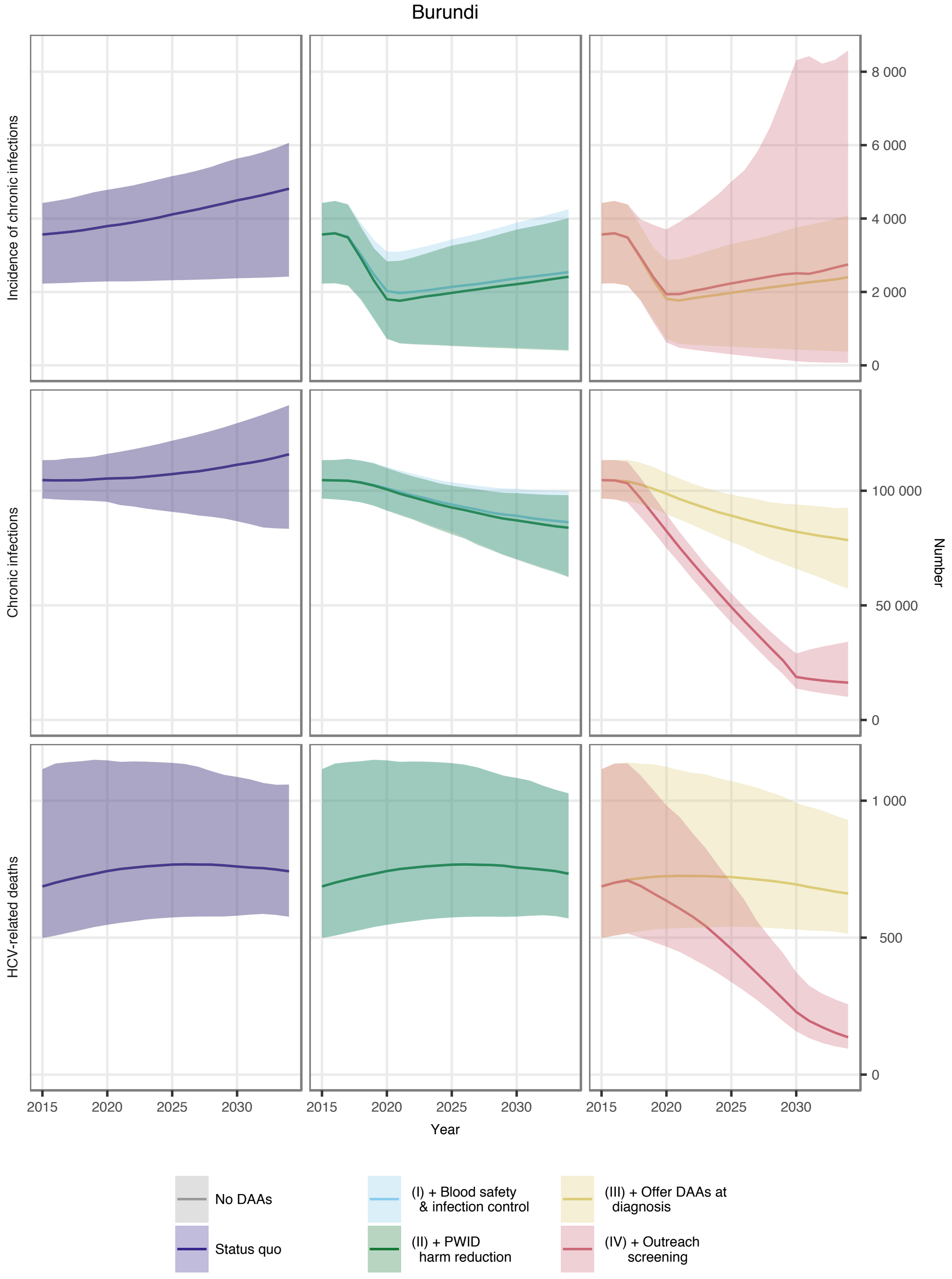
Burundi
Incidence of chronic infections
Chronic infections
HCV-related deaths
Number
8 000
6 000
4 000
2 000
0
100 000
50 000
0
1 000
500
0
2015
2020
2025
2030
Year
No DAAs
Status quo
(I) + Blood safety & infection control
(II) + PWID harm reduction
(III) + Offer DAAs at diagnosis
(IV) + Outreach screening

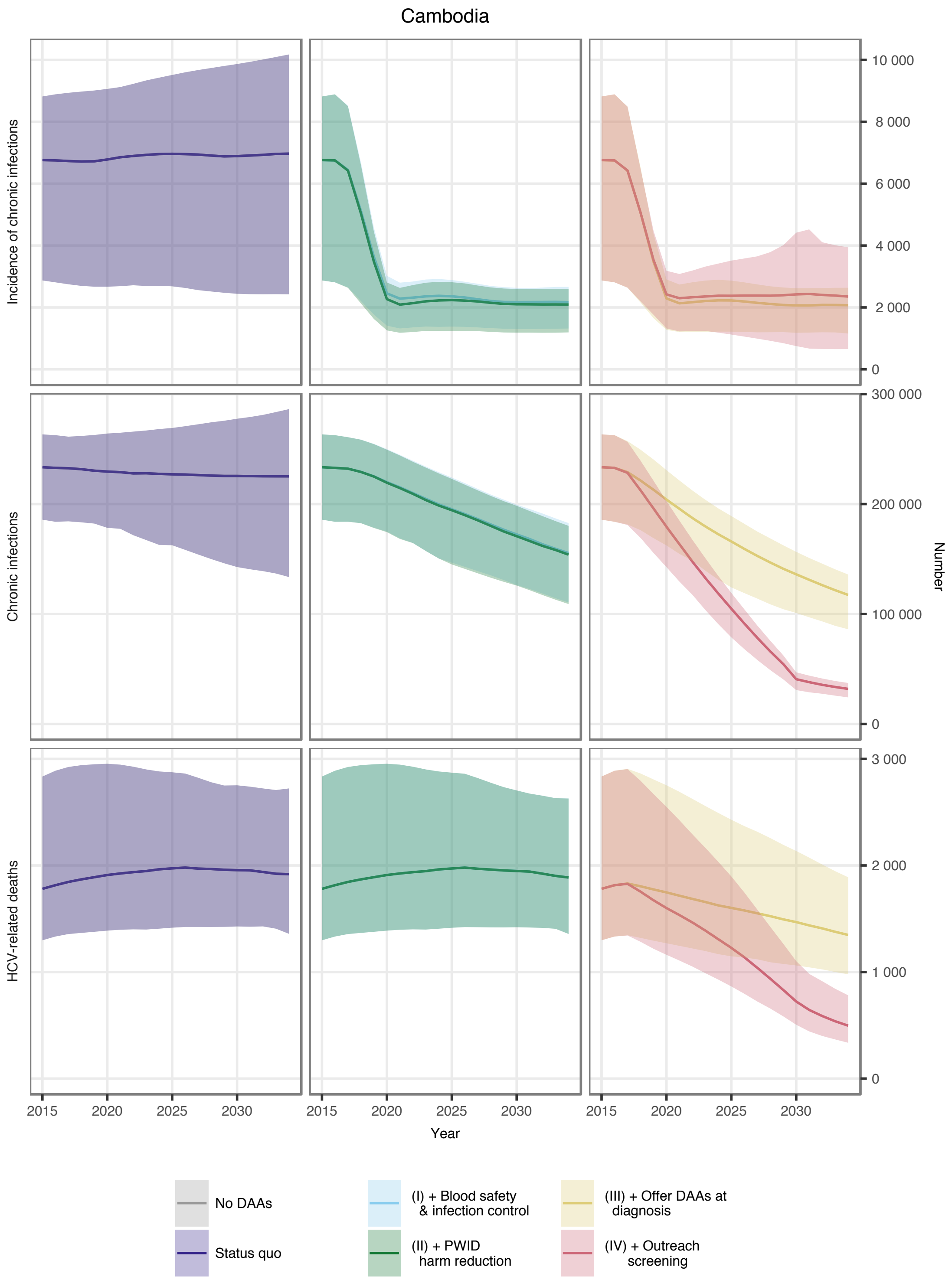

Cambodia
Incidence of chronic infections
Chronic infections
HCV-related deaths
Number
Year
0
2 000
4 000
6 000
8 000
10 000
0
100 000
200 000
300 000
0
1 000
2 000
3 000
2015
2020
2025
2030
No DAAs
Status quo
(I) + Blood safety & infection control
(II) + PWID harm reduction
(III) + Offer DAAs at diagnosis
(IV) + Outreach screening

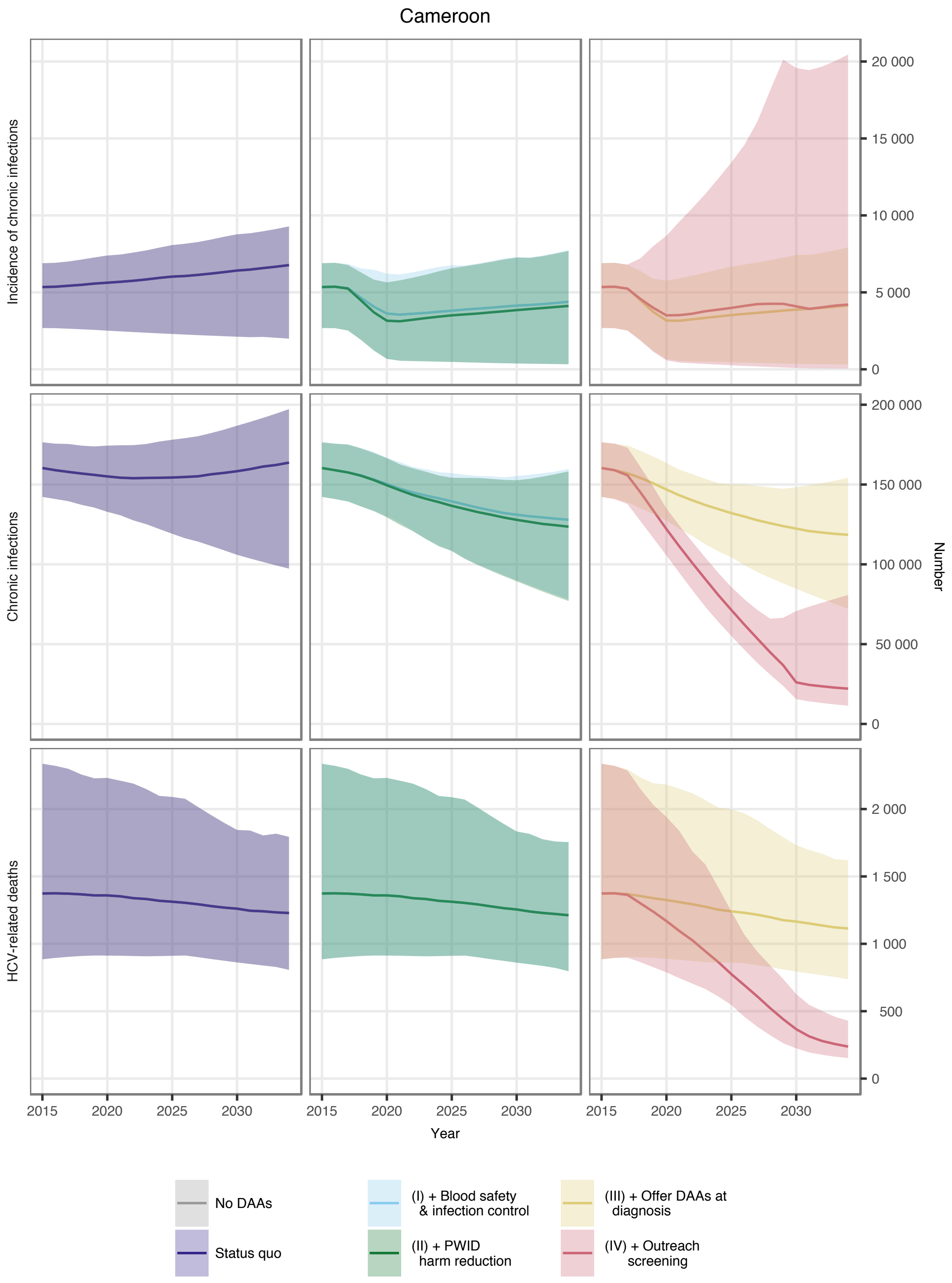
Cameroon
Incidence of chronic infections
Chronic infections
HCV-related deaths
Number
20 000
15 000
10 000
5 000
0
200 000
150 000
100 000
50 000
0
2 000
1 500
1 000
500
0
2015
2020
2025
2030
Year
No DAAs
Status quo
(I) + Blood safety & infection control
(II) + PWID harm reduction
(III) + Offer DAAs at diagnosis
(IV) + Outreach screening

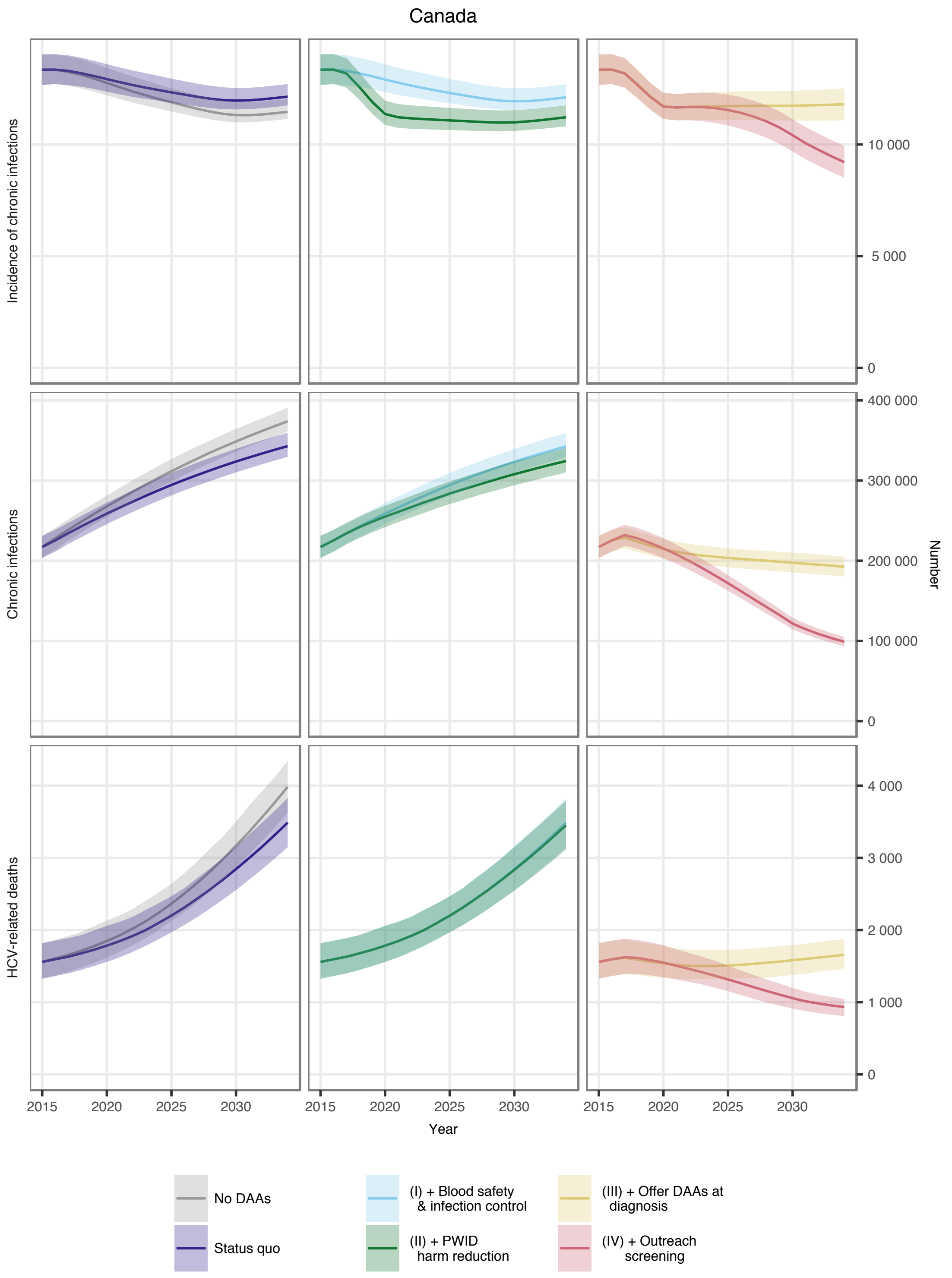
Canada
Incidence of chronic infections
Chronic infections
HCV-related deaths
10 000
5 000
0
400 000
300 000
200 000
100 000
0
4 000
3 000
2 000
1 000
0
Number
2015
2020
2025
2030
Year
No DAAs
Status quo
(I) + Blood safety & infection control
(II) + PWID harm reduction
(III) + Offer DAAs at diagnosis
(IV) + Outreach screening

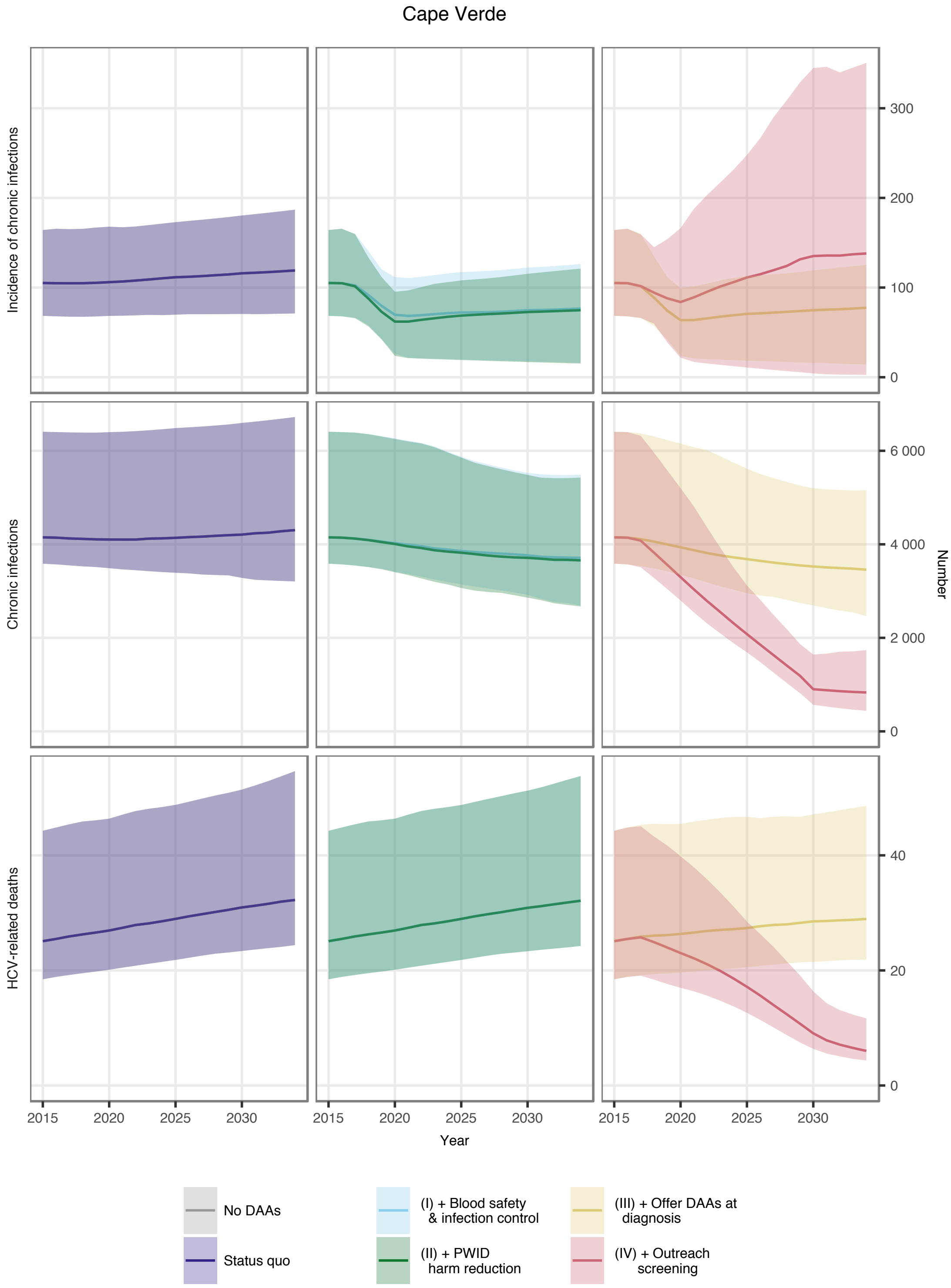
Cape Verde
Incidence of chronic infections
Chronic infections
HCV-related deaths
Number
Year
300
200
100
0
6 000
4 000
2 000
0
40
20
0
2015
2020
2025
2030
No DAAs
Status quo
(I) + Blood safety & infection control
(II) + PWID harm reduction
(III) + Offer DAAs at diagnosis
(IV) + Outreach screening

Central African Republic

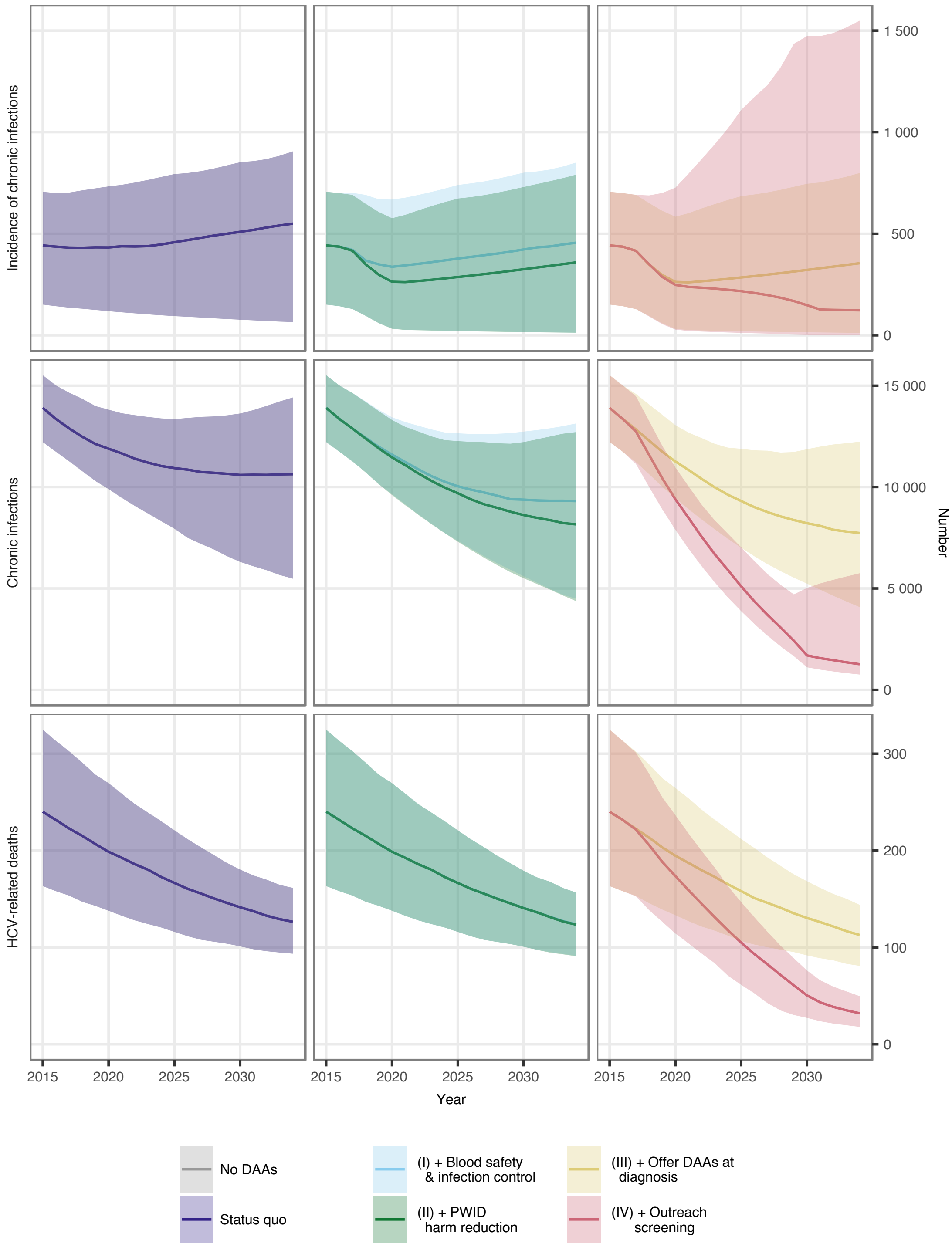

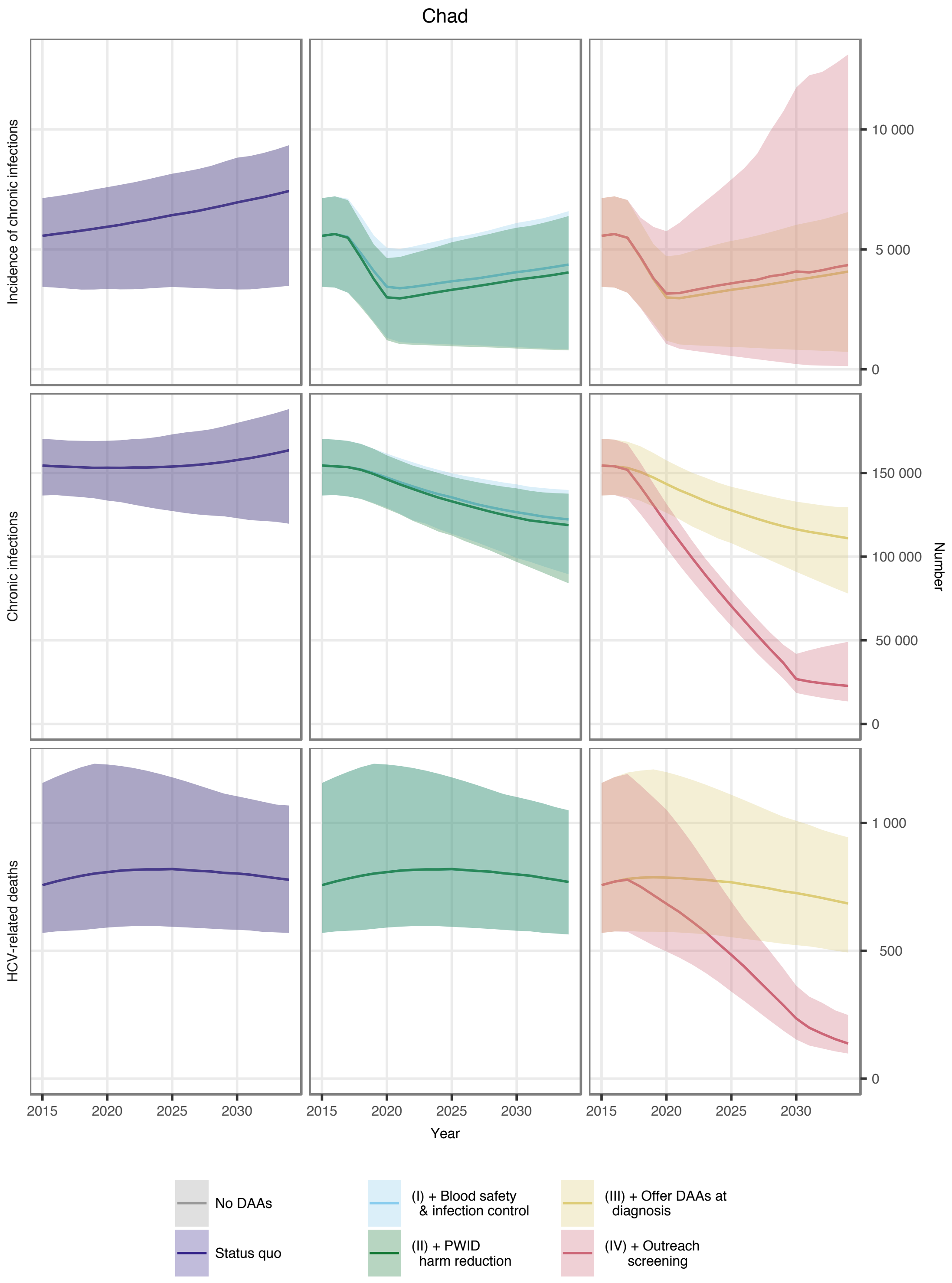
Chad
Incidence of chronic infections
Chronic infections
HCV-related deaths
Number
10 000
5 000
0
150 000
100 000
50 000
0
1 000
500
0
2015
2020
2025
2030
Year
No DAAs
Status quo
(I) + Blood safety & infection control
(II) + PWID harm reduction
(III) + Offer DAAs at diagnosis
(IV) + Outreach screening

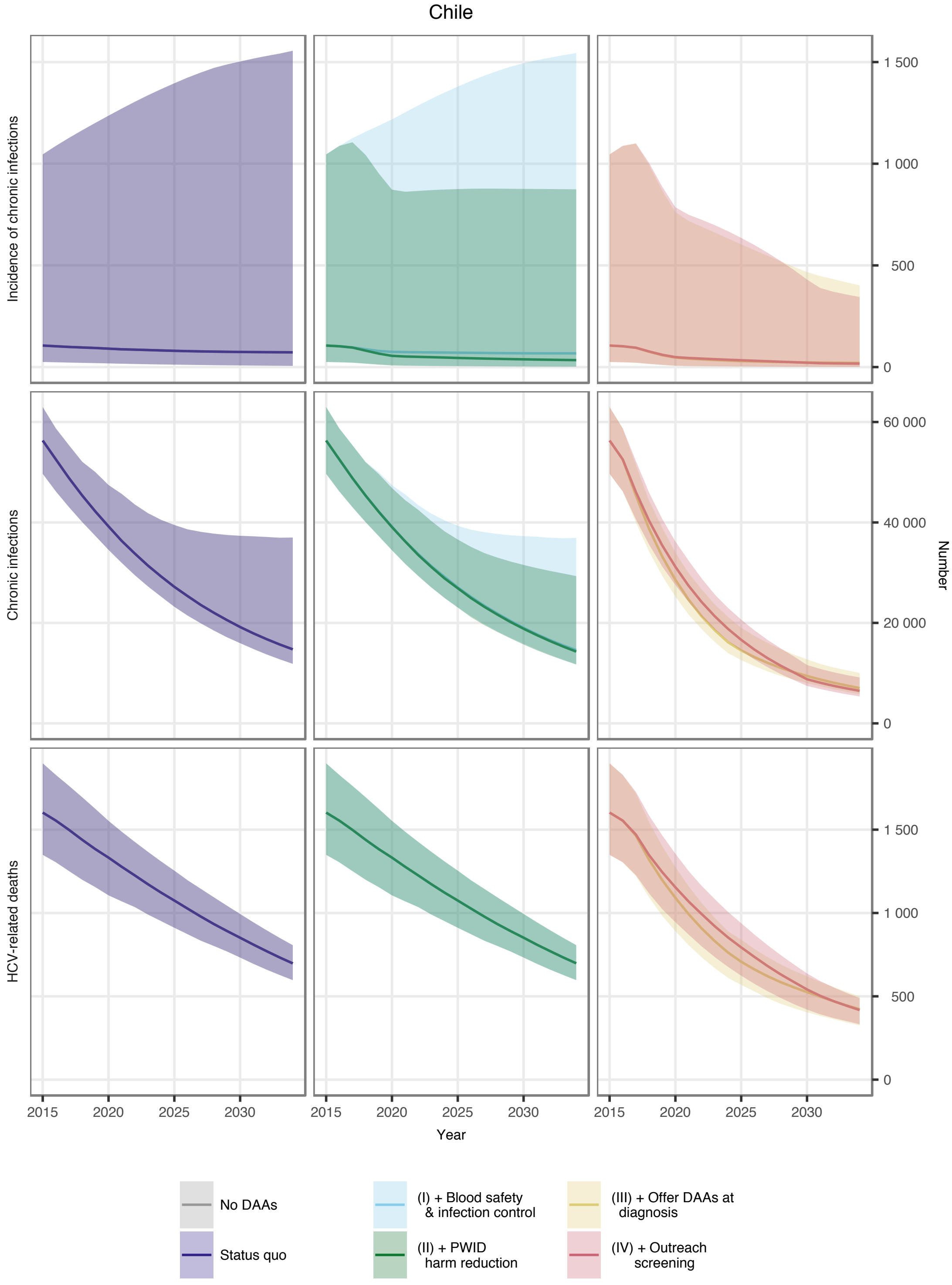
Chile
Incidence of chronic infections
Chronic infections
HCV-related deaths
Number
Year
2015
2020
2025
2030
0
500
1 000
1 500
20 000
40 000
60 000
No DAAs
Status quo
(I) + Blood safety & infection control
(II) + PWID harm reduction
(III) + Offer DAAs at diagnosis
(IV) + Outreach screening

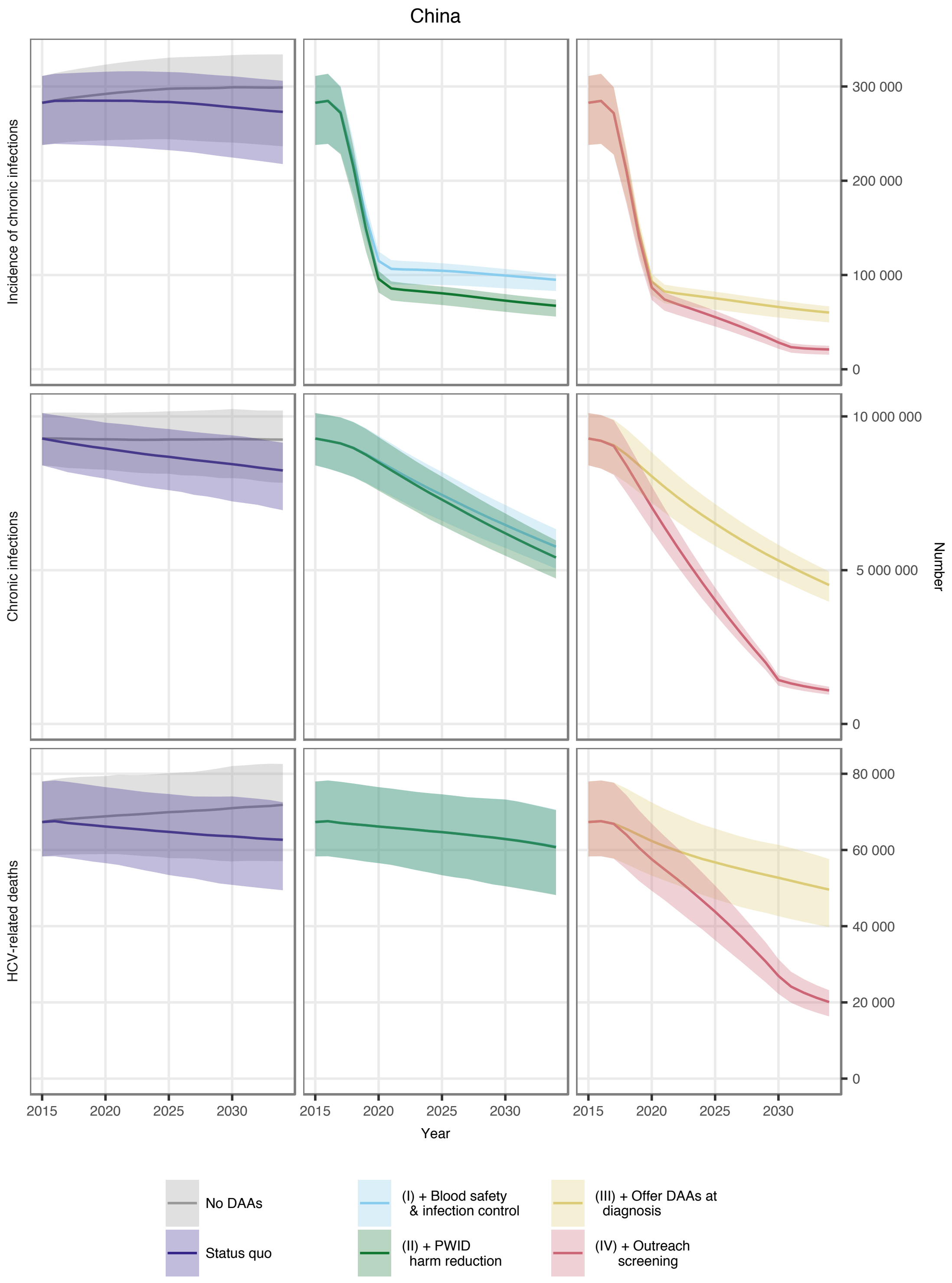
China
Incidence of chronic infections
Chronic infections
HCV-related deaths
300 000
200 000
100 000
0
10 000 000
5 000 000
0
80 000
60 000
40 000
20 000
0
Number
2015
2020
2025
2030
Year
No DAAs
Status quo
(I) + Blood safety & infection control
(II) + PWID harm reduction
(III) + Offer DAAs at diagnosis
(IV) + Outreach screening

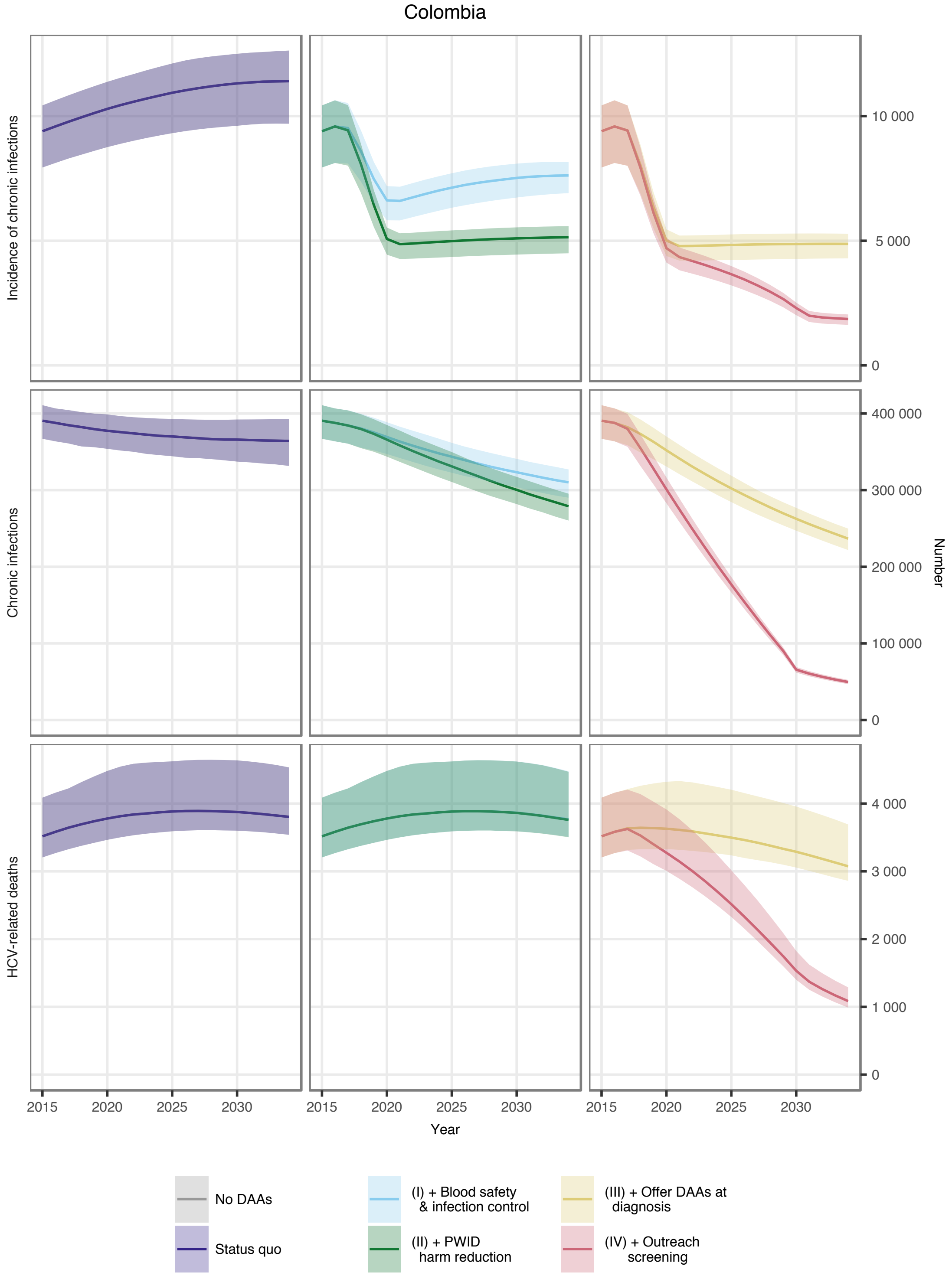

Colombia
Incidence of chronic infections
Chronic infections
HCV-related deaths
10 000
5 000
0
400 000
300 000
200 000
100 000
0
4 000
3 000
2 000
1 000
0
Number
2015
2020
2025
2030
Year
No DAAs
Status quo
(I) + Blood safety & infection control
(II) + PWID harm reduction
(III) + Offer DAAs at diagnosis
(IV) + Outreach screening

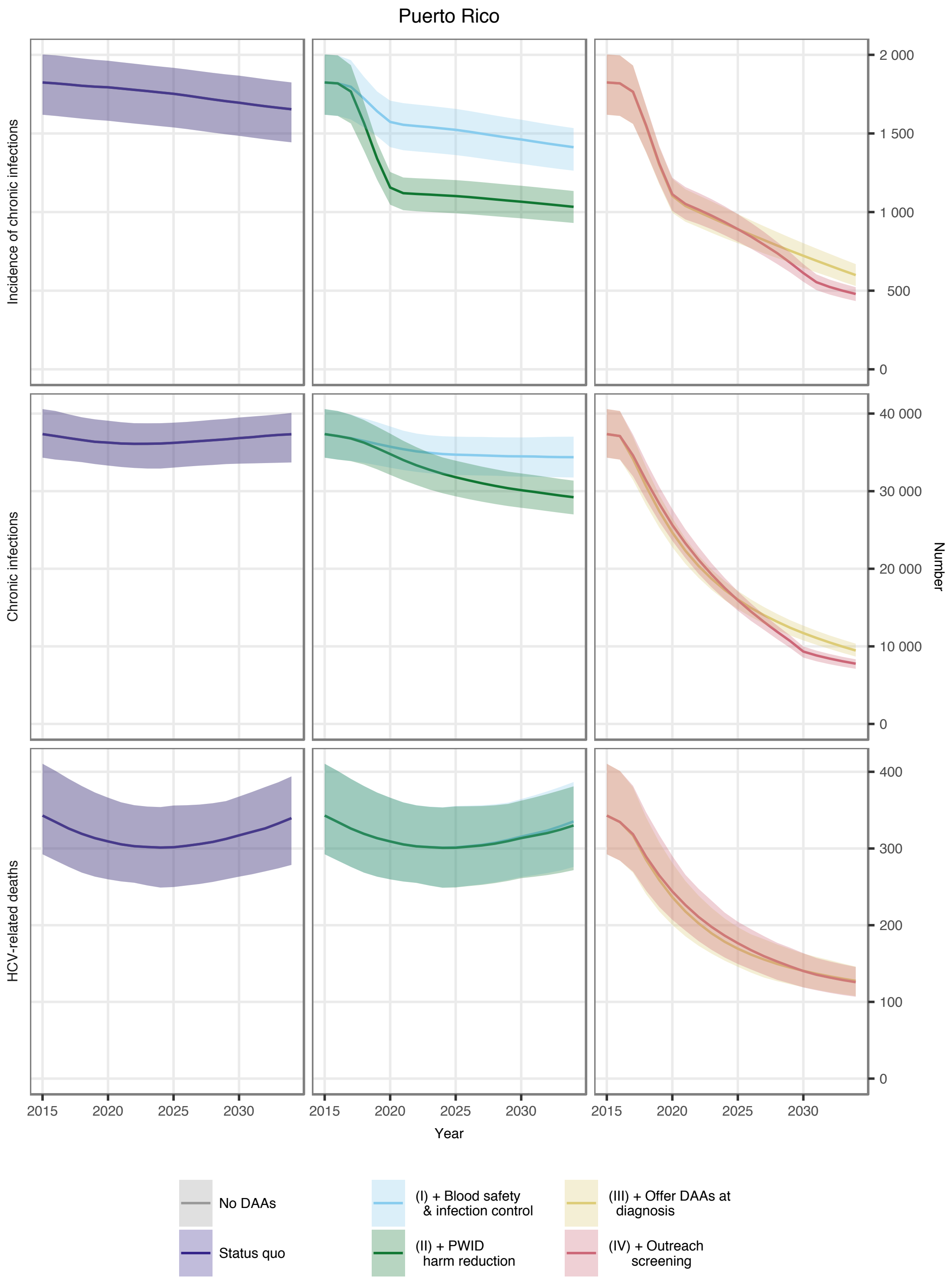
Puerto Rico
Incidence of chronic infections
Chronic infections
HCV-related deaths
Number
2 000
1 500
1 000
500
0
40 000
30 000
20 000
10 000
0
400
300
200
100
0
2015
2020
2025
2030
Year
No DAAs
Status quo
(I) + Blood safety & infection control
(II) + PWID harm reduction
(III) + Offer DAAs at diagnosis
(IV) + Outreach screening

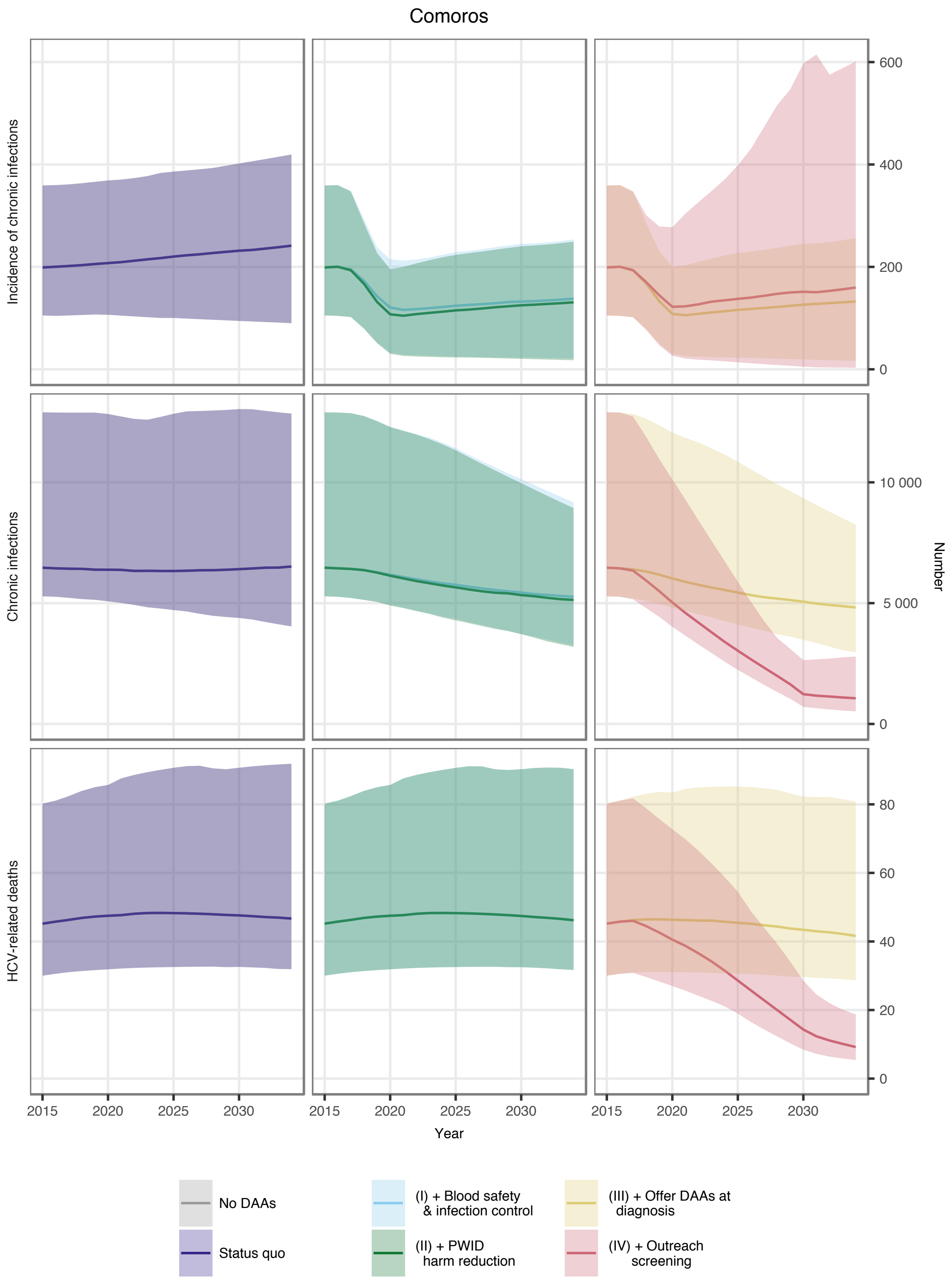

Comoros
Incidence of chronic infections
Chronic infections
HCV-related deaths
Number
Year
600
400
200
0
10 000
5 000
0
80
60
40
20
0
2015
2020
2025
2030
No DAAs
Status quo
(I) + Blood safety & infection control
(II) + PWID harm reduction
(III) + Offer DAAs at diagnosis
(IV) + Outreach screening

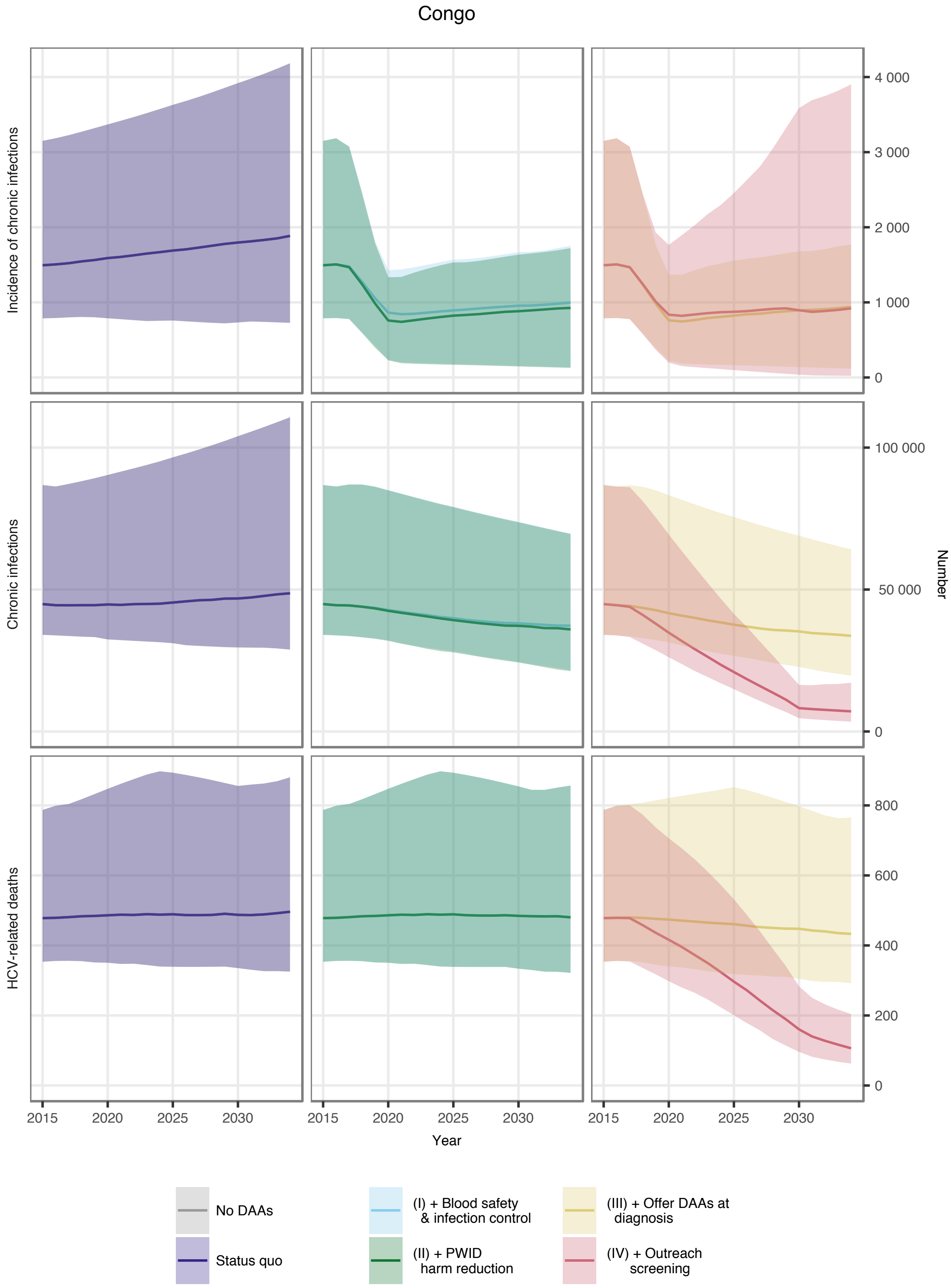

Congo
Incidence of chronic infections
Chronic infections
HCV-related deaths
Number
4 000
3 000
2 000
1 000
0
100 000
50 000
0
800
600
400
200
0
2015
2020
2025
2030
Year
No DAAs
Status quo
(I) + Blood safety & infection control
(II) + PWID harm reduction
(III) + Offer DAAs at diagnosis
(IV) + Outreach screening

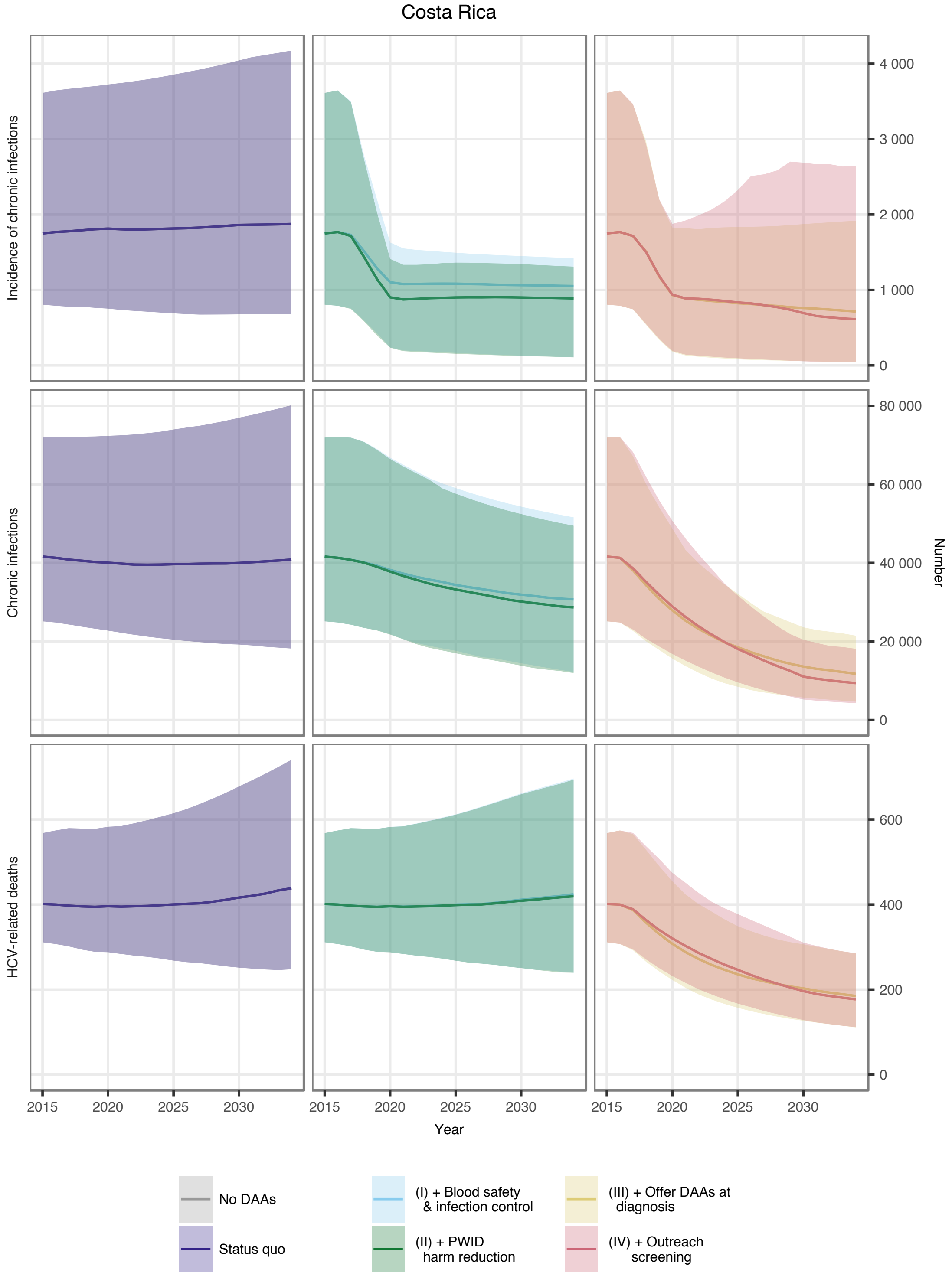
Costa Rica
Incidence of chronic infections
Chronic infections
HCV-related deaths
Number
Year
0
1 000
2 000
3 000
4 000
20 000
40 000
60 000
80 000
200
400
600
2015
2020
2025
2030
No DAAs
Status quo
(I) + Blood safety & infection control
(II) + PWID harm reduction
(III) + Offer DAAs at diagnosis
(IV) + Outreach screening

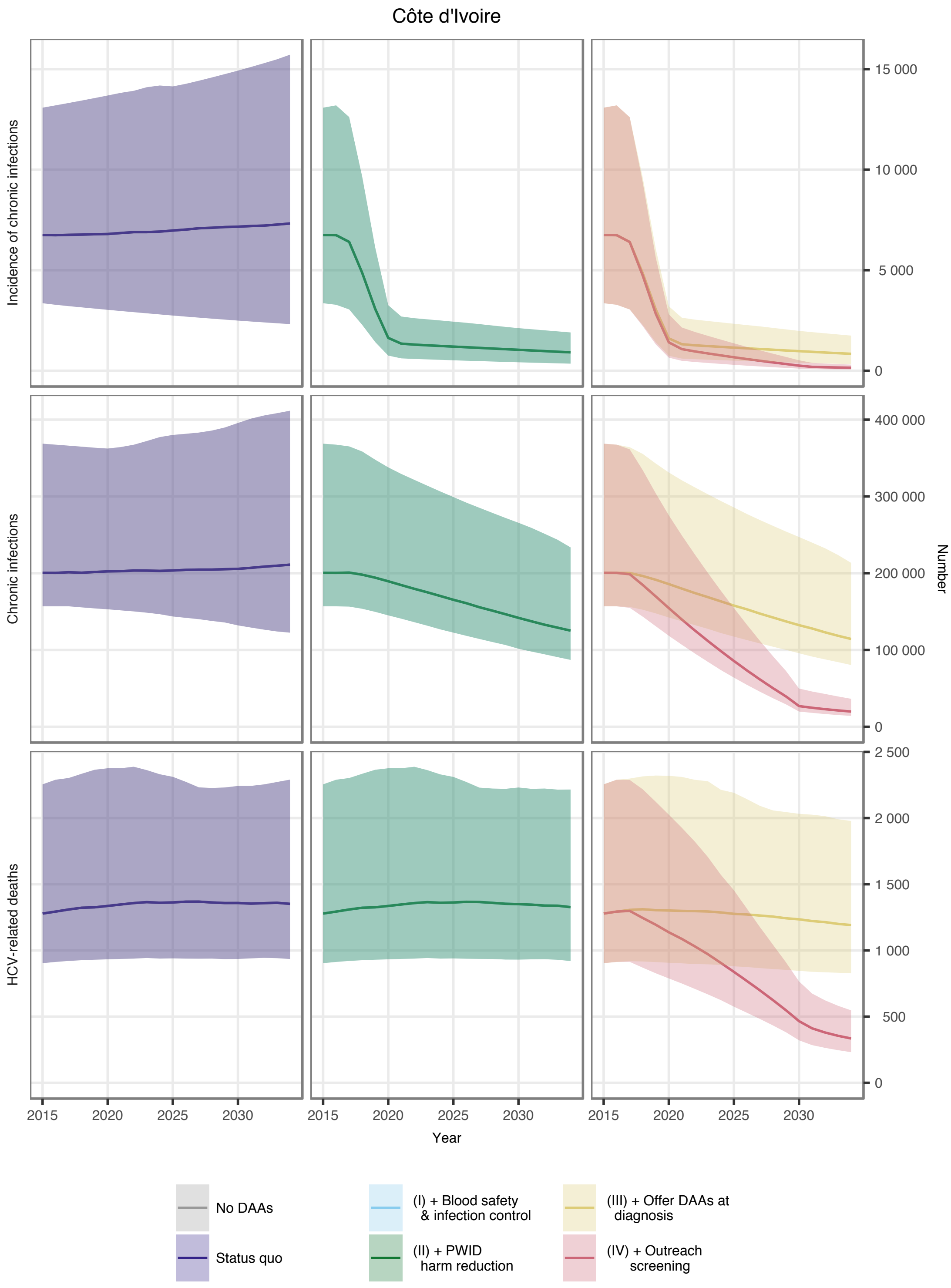

Côte d'Ivoire
Incidence of chronic infections
Chronic infections
HCV-related deaths
Number
15 000
10 000
5 000
0
400 000
300 000
200 000
100 000
0
2 500
2 000
1 500
1 000
500
0
2015
2020
2025
2030
Year
No DAAs
Status quo
(I) + Blood safety & infection control
(II) + PWID harm reduction
(III) + Offer DAAs at diagnosis
(IV) + Outreach screening

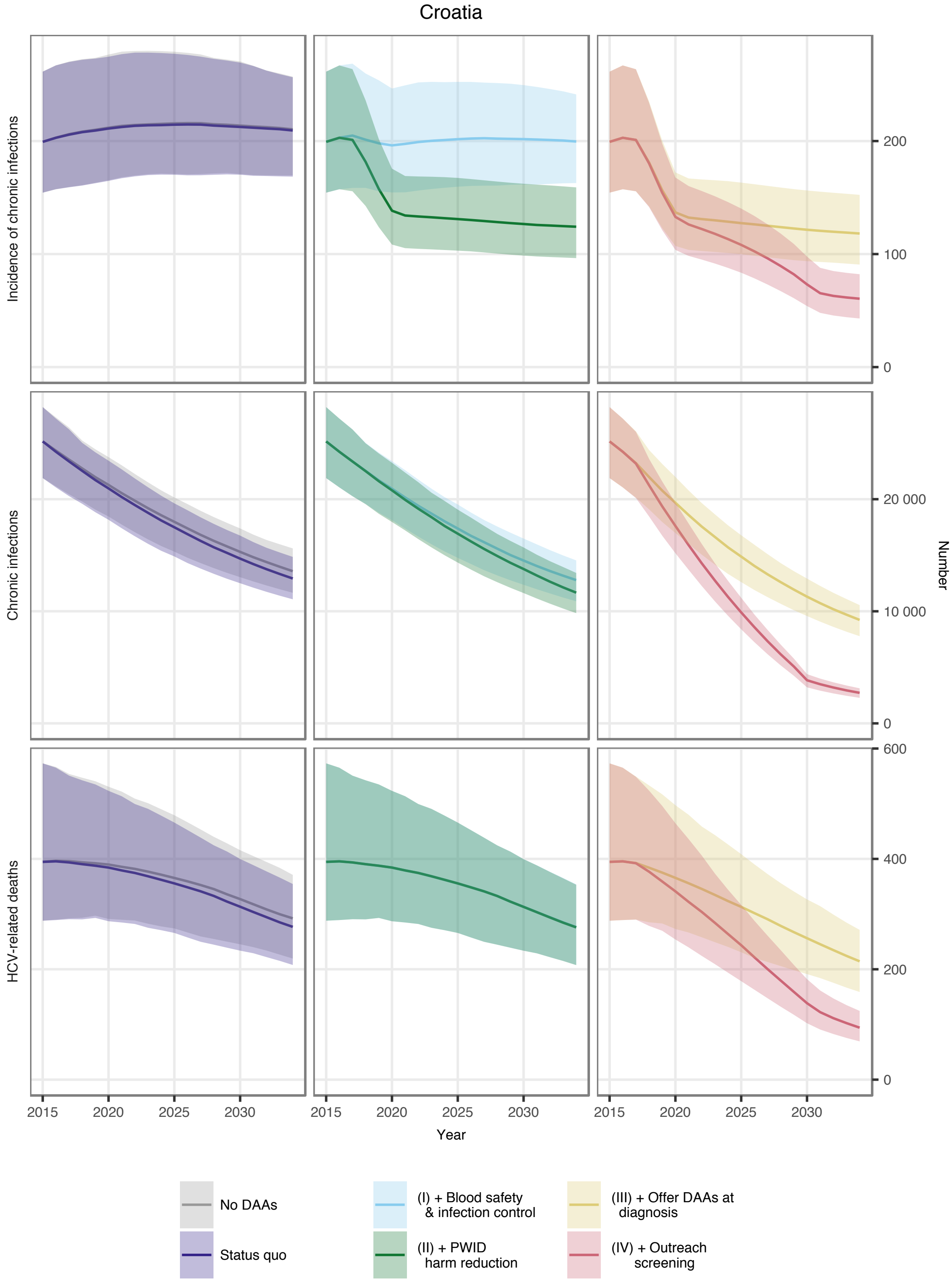

Croatia
Incidence of chronic infections
Chronic infections
HCV-related deaths
Number
0
100
200
0
10 000
20 000
0
200
400
600
2015
2020
2025
2030
Year
No DAAs
Status quo
(I) + Blood safety & infection control
(II) + PWID harm reduction
(III) + Offer DAAs at diagnosis
(IV) + Outreach screening

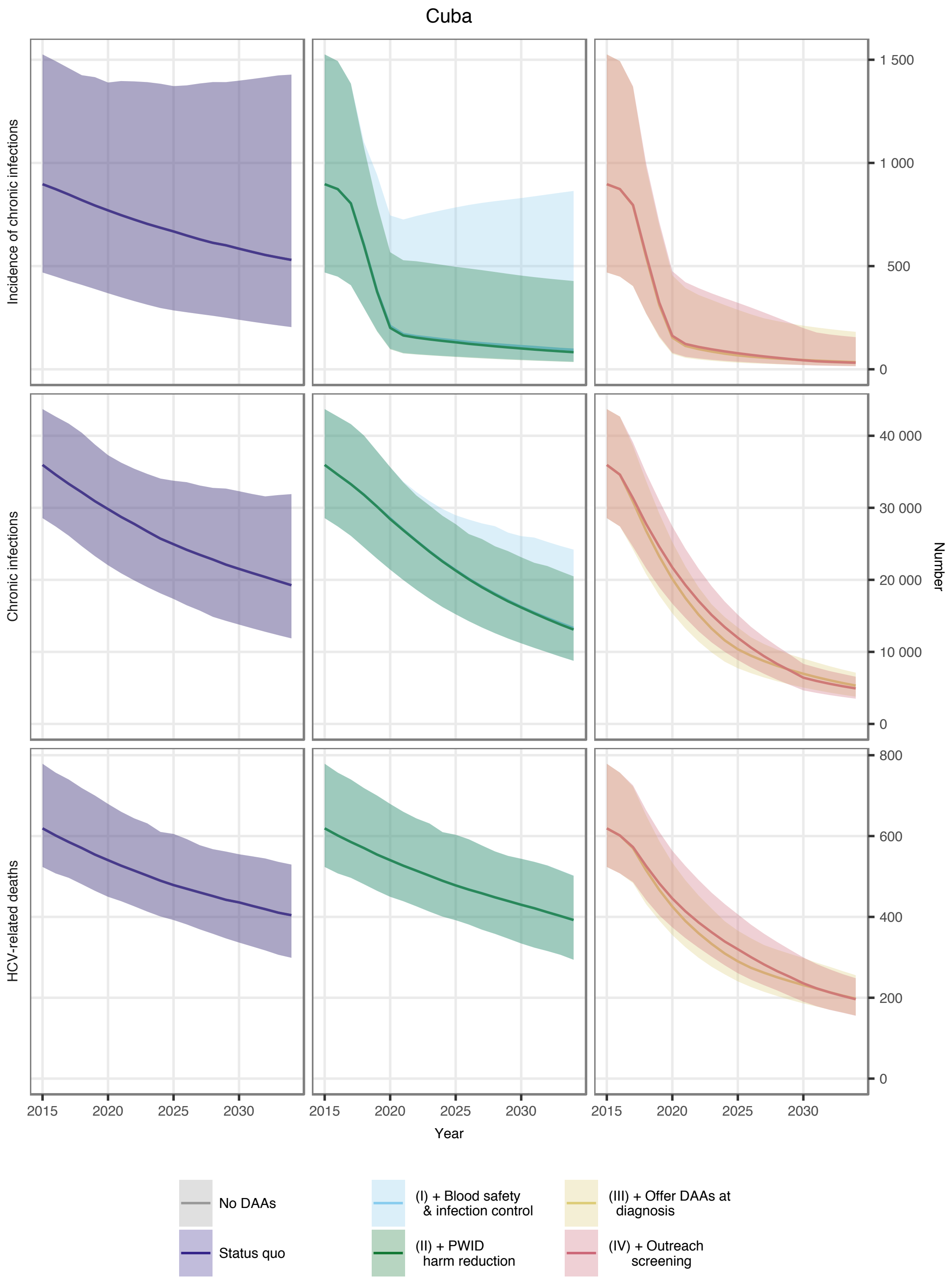
Cuba
Incidence of chronic infections
Chronic infections
HCV-related deaths
Number
Year
2015
2020
2025
2030
1 500
1 000
500
0
40 000
30 000
20 000
10 000
0
800
600
400
200
0
No DAAs
Status quo
(I) + Blood safety & infection control
(II) + PWID harm reduction
(III) + Offer DAAs at diagnosis
(IV) + Outreach screening

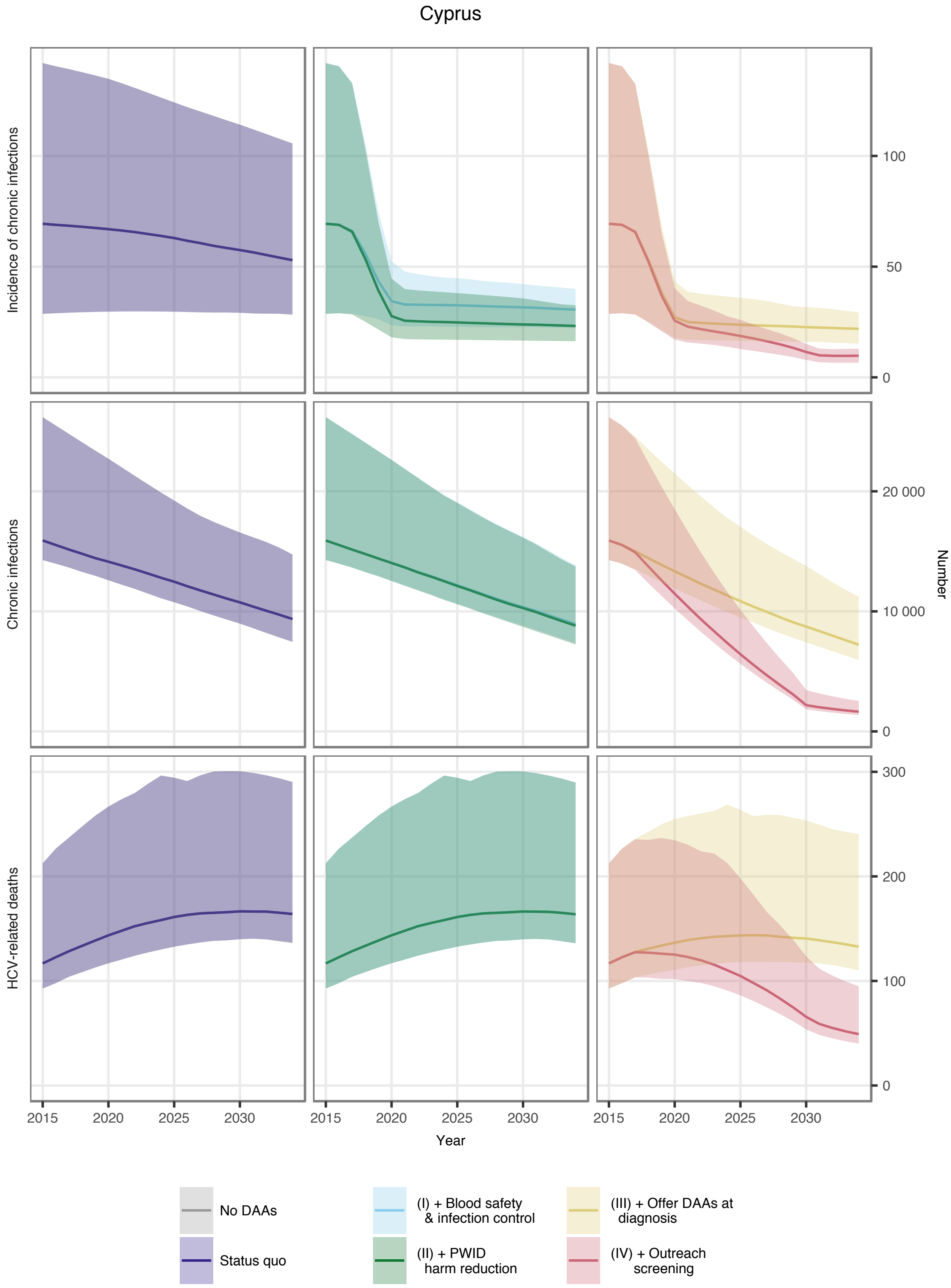
Cyprus
Incidence of chronic infections
Chronic infections
HCV-related deaths
Number
Year
0
50
100
0
10 000
20 000
0
100
200
300
2015
2020
2025
2030
No DAAs
Status quo
(I) + Blood safety & infection control
(II) + PWID harm reduction
(III) + Offer DAAs at diagnosis
(IV) + Outreach screening

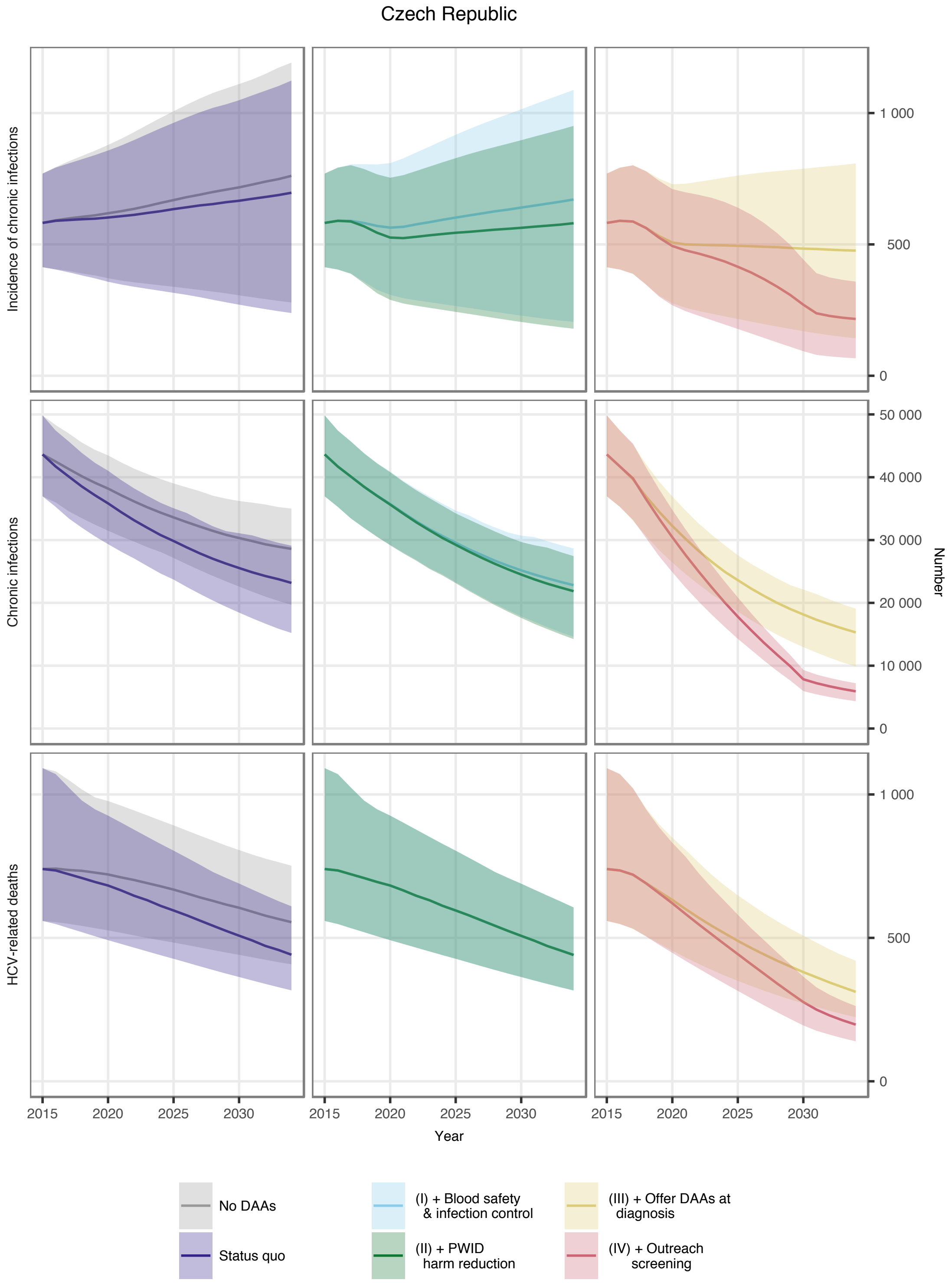
Czech Republic
Incidence of chronic infections
Chronic infections
HCV-related deaths
Number
1 000
500
0
50 000
40 000
30 000
20 000
10 000
0
1 000
500
0
2015
2020
2025
2030
Year
No DAAs
Status quo
(I) + Blood safety & infection control
(II) + PWID harm reduction
(III) + Offer DAAs at diagnosis
(IV) + Outreach screening

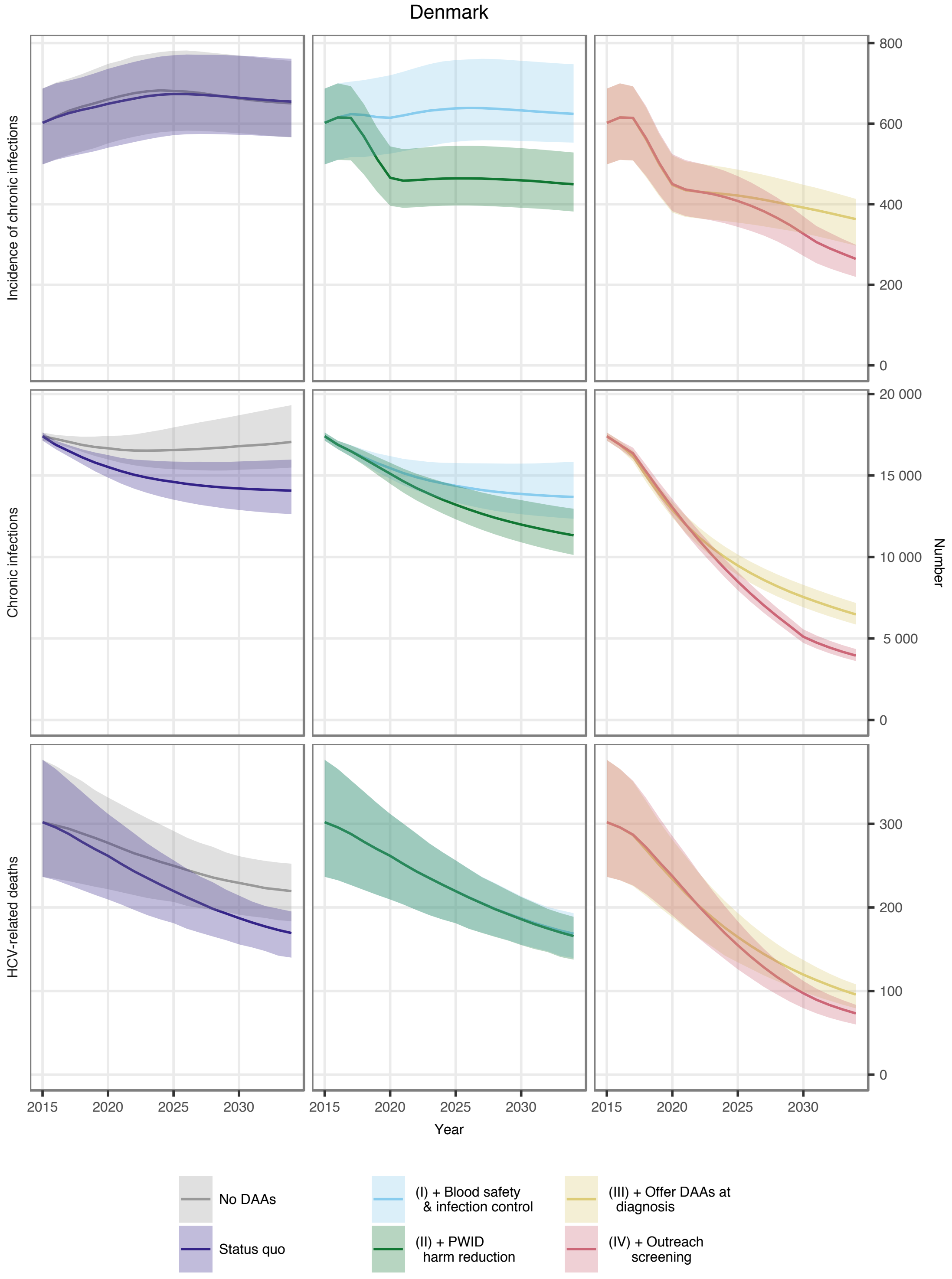
Denmark
Incidence of chronic infections
Chronic infections
HCV-related deaths
800
600
400
200
0
20 000
15 000
10 000
5 000
0
300
200
100
0
Number
2015
2020
2025
2030
Year
No DAAs
Status quo
(I) + Blood safety & infection control
(II) + PWID harm reduction
(III) + Offer DAAs at diagnosis
(IV) + Outreach screening

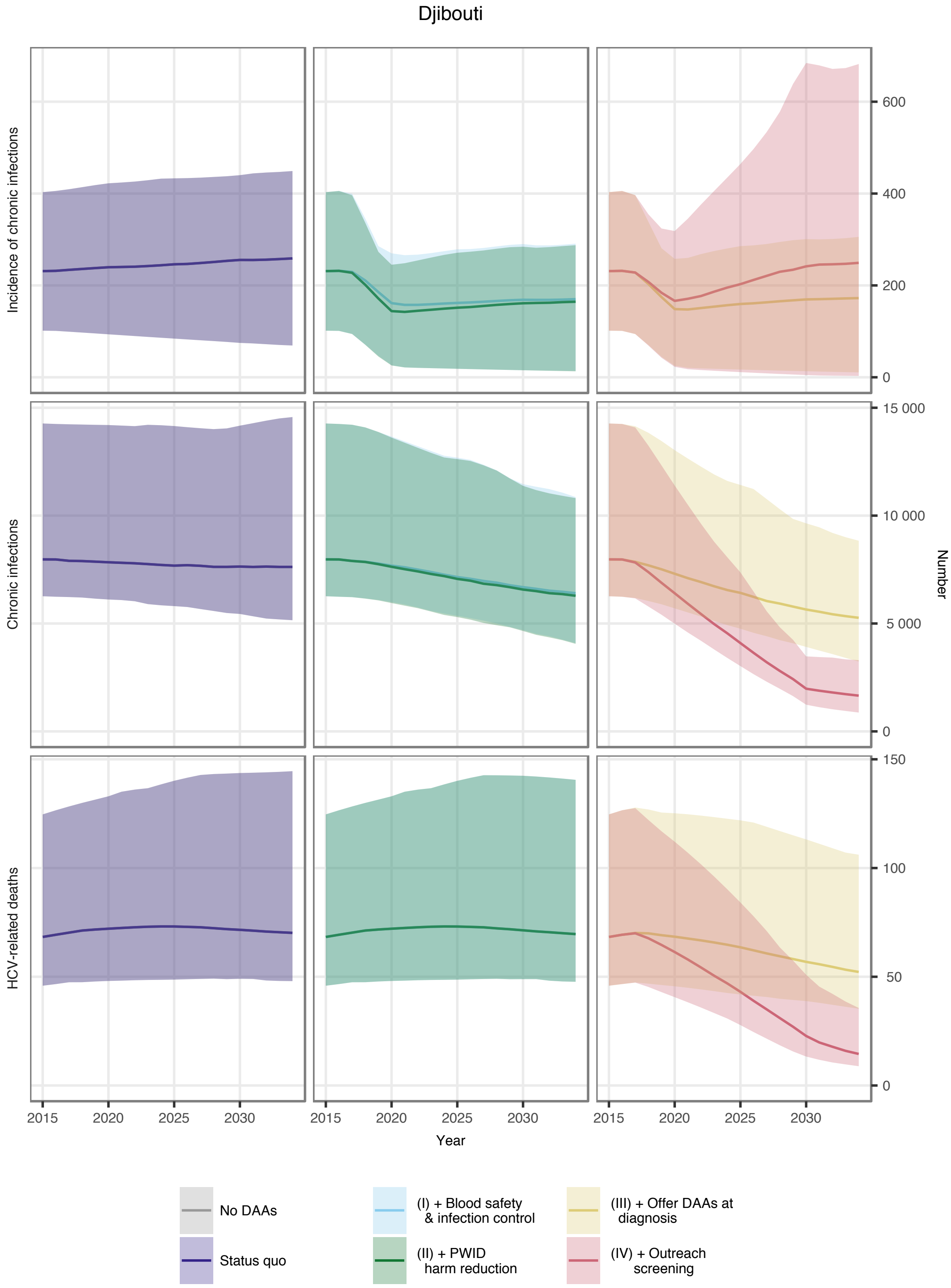
Djibouti
Incidence of chronic infections
Chronic infections
HCV-related deaths
Number
0
200
400
600
0
5 000
10 000
15 000
0
50
100
150
2015
2020
2025
2030
Year
No DAAs
Status quo
(I) + Blood safety & infection control
(II) + PWID harm reduction
(III) + Offer DAAs at diagnosis
(IV) + Outreach screening

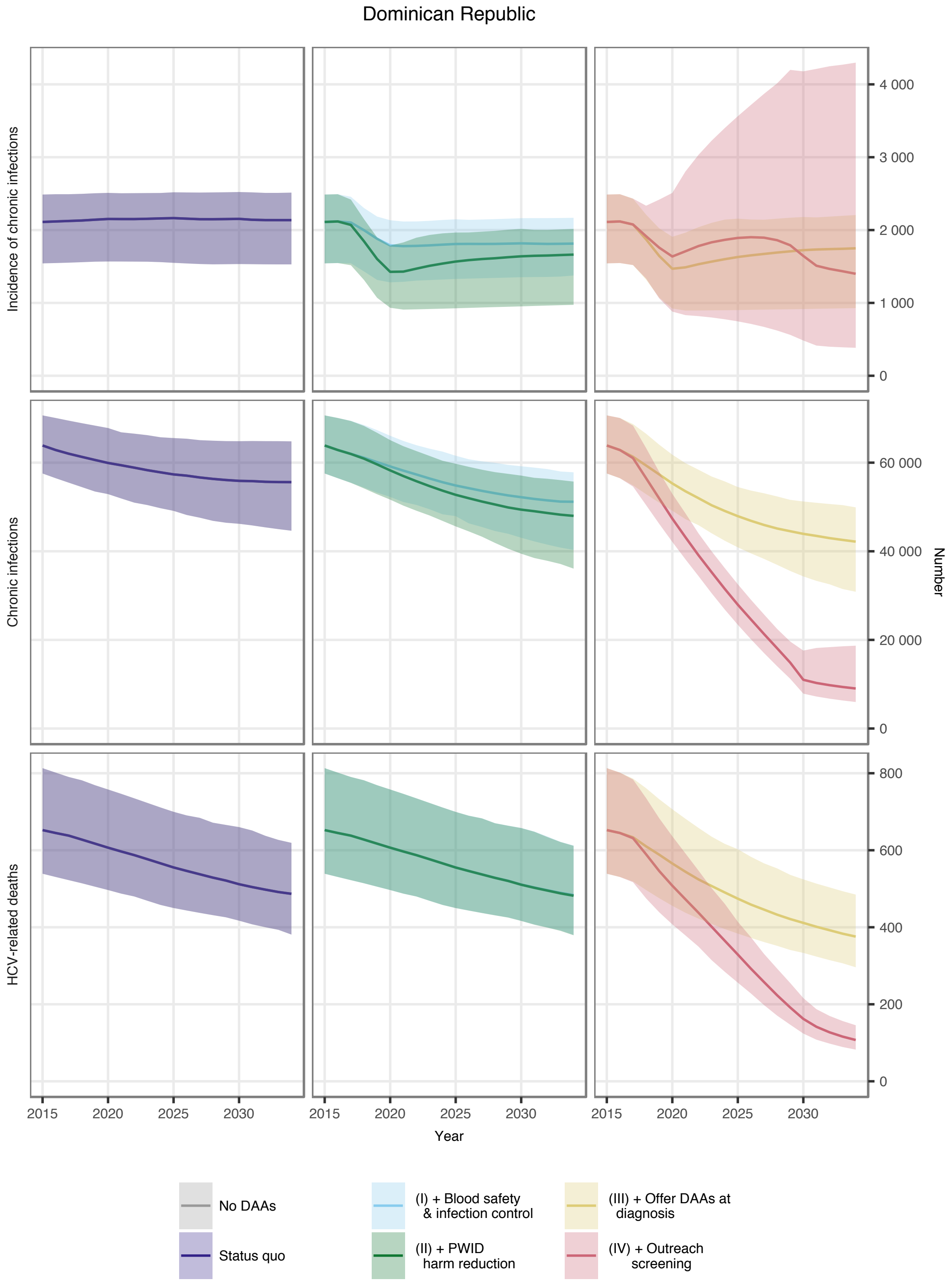
Dominican Republic
Incidence of chronic infections
Chronic infections
HCV-related deaths
Number
4 000
3 000
2 000
1 000
0
60 000
40 000
20 000
0
800
600
400
200
0
2015
2020
2025
2030
Year
No DAAs
Status quo
(I) + Blood safety & infection control
(II) + PWID harm reduction
(III) + Offer DAAs at diagnosis
(IV) + Outreach screening

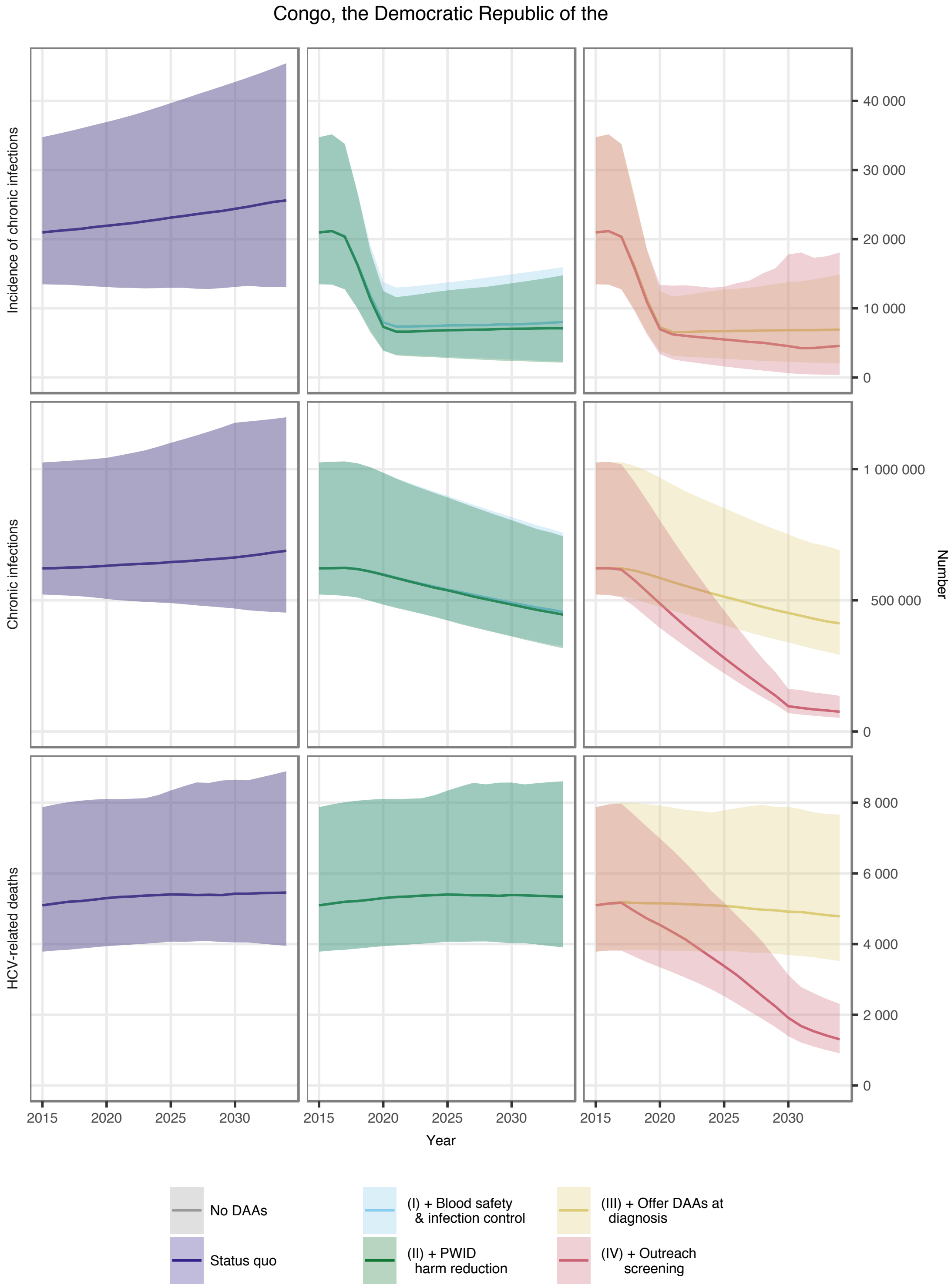

Congo, the Democratic Republic of the
Incidence of chronic infections
Chronic infections
HCV-related deaths
Number
40 000
30 000
20 000
10 000
0
1 000 000
500 000
0
8 000
6 000
4 000
2 000
0
2015
2020
2025
2030
Year
No DAAs
Status quo
(I) + Blood safety & infection control
(II) + PWID harm reduction
(III) + Offer DAAs at diagnosis
(IV) + Outreach screening

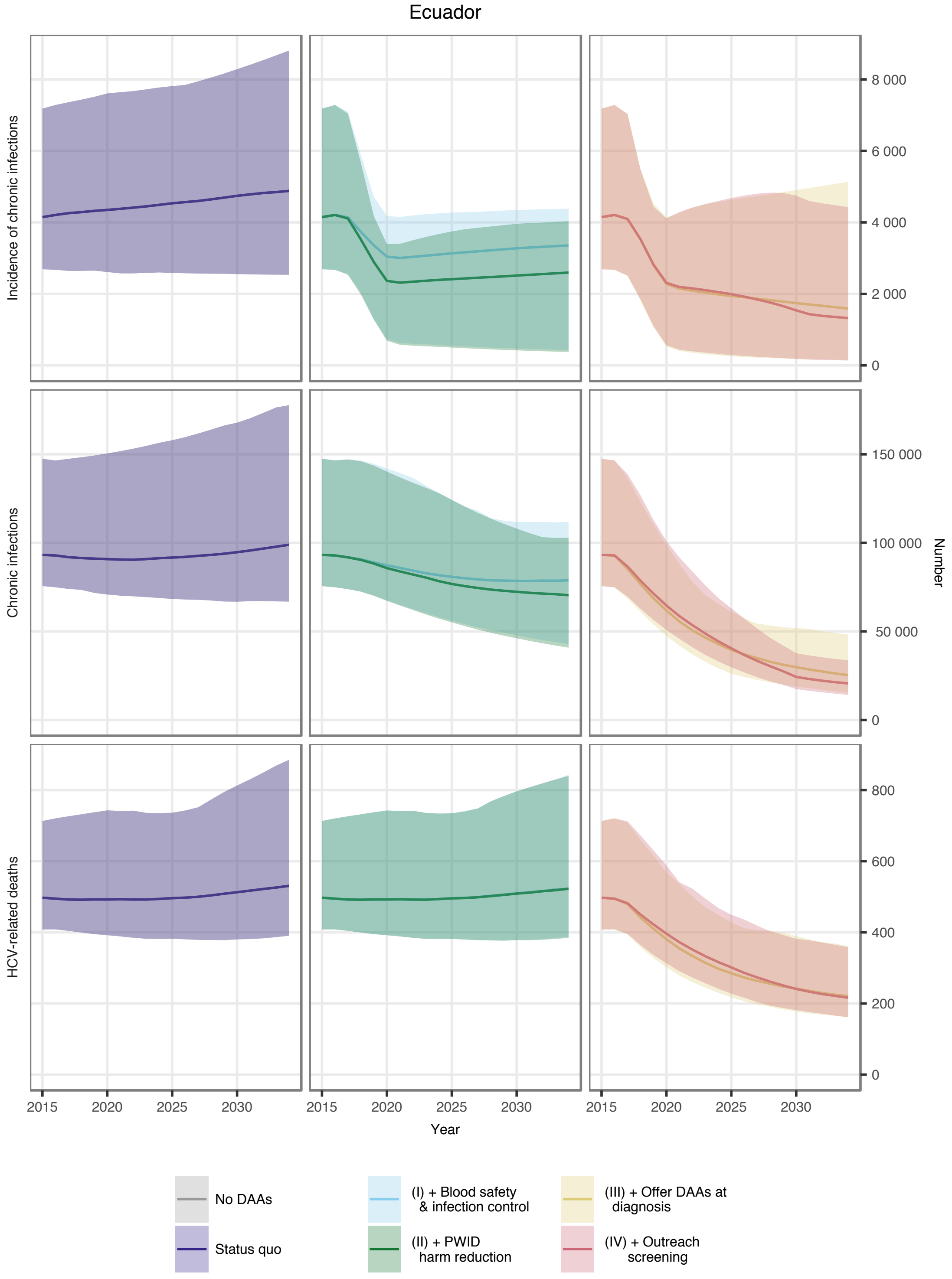
Ecuador
Incidence of chronic infections
Chronic infections
HCV-related deaths
Number
8 000
6 000
4 000
2 000
0
150 000
100 000
50 000
0
800
600
400
200
0
2015
2020
2025
2030
Year
No DAAs
Status quo
(I) + Blood safety & infection control
(II) + PWID harm reduction
(III) + Offer DAAs at diagnosis
(IV) + Outreach screening

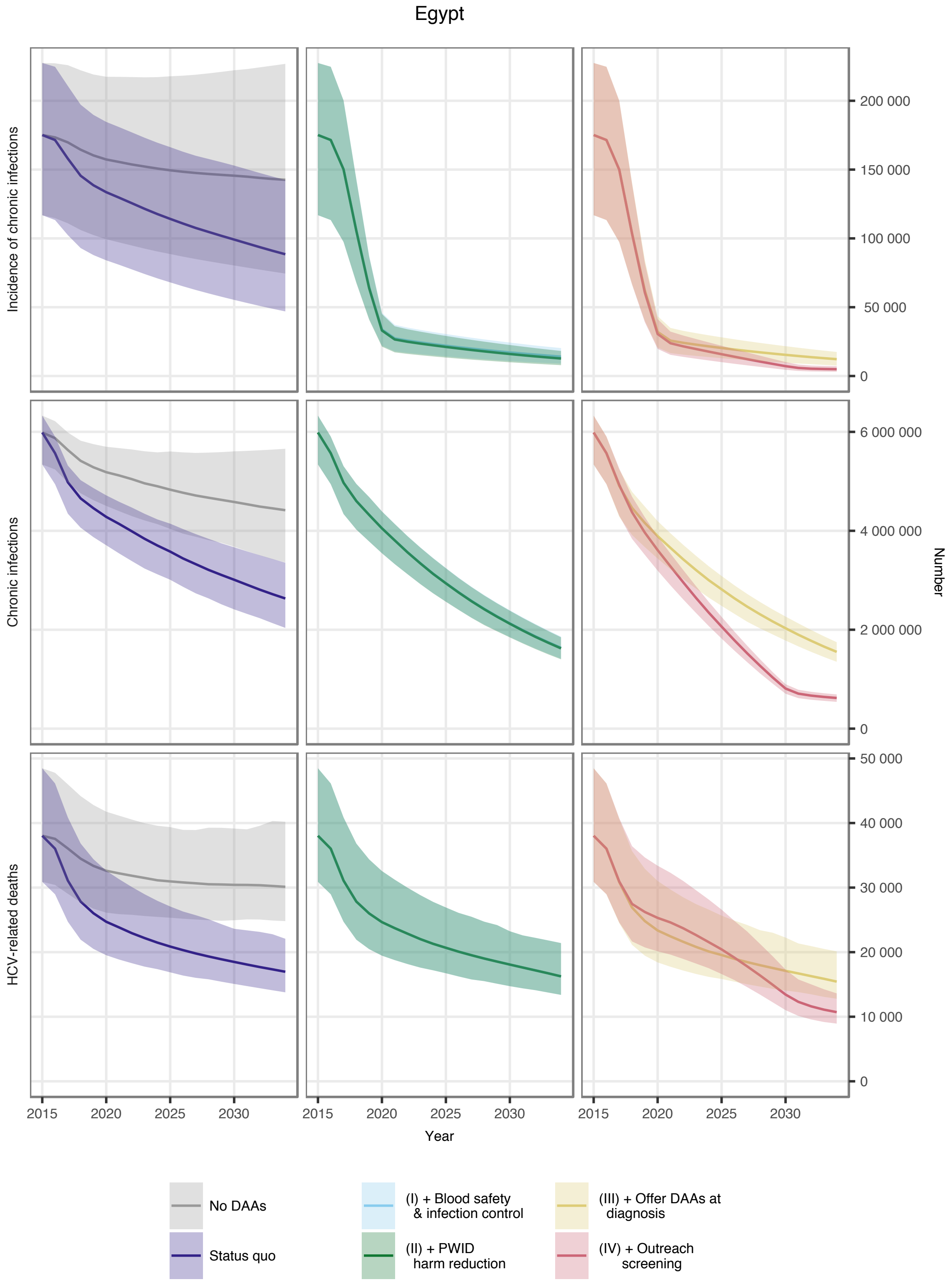

Egypt
Incidence of chronic infections
Chronic infections
HCV-related deaths
Number
200 000
150 000
100 000
50 000
0
6 000 000
4 000 000
2 000 000
0
50 000
40 000
30 000
20 000
10 000
0
2015
2020
2025
2030
Year
No DAAs
Status quo
(I) + Blood safety & infection control
(II) + PWID harm reduction
(III) + Offer DAAs at diagnosis
(IV) + Outreach screening

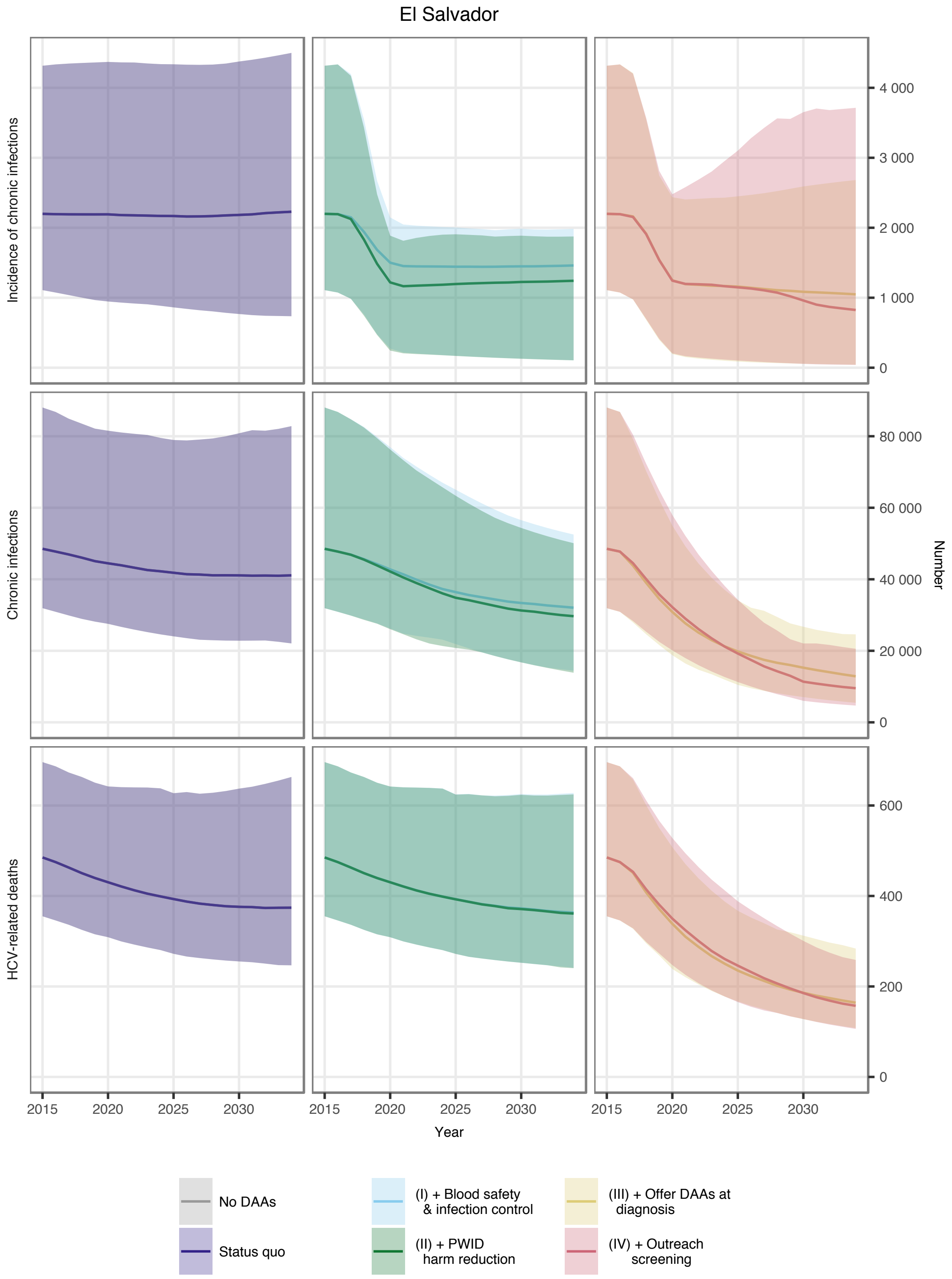

El Salvador
Incidence of chronic infections
Chronic infections
HCV-related deaths
Number
Year
0
1 000
2 000
3 000
4 000
20 000
40 000
60 000
80 000
200
400
600
2015
2020
2025
2030
No DAAs
Status quo
(I) + Blood safety & infection control
(II) + PWID harm reduction
(III) + Offer DAAs at diagnosis
(IV) + Outreach screening

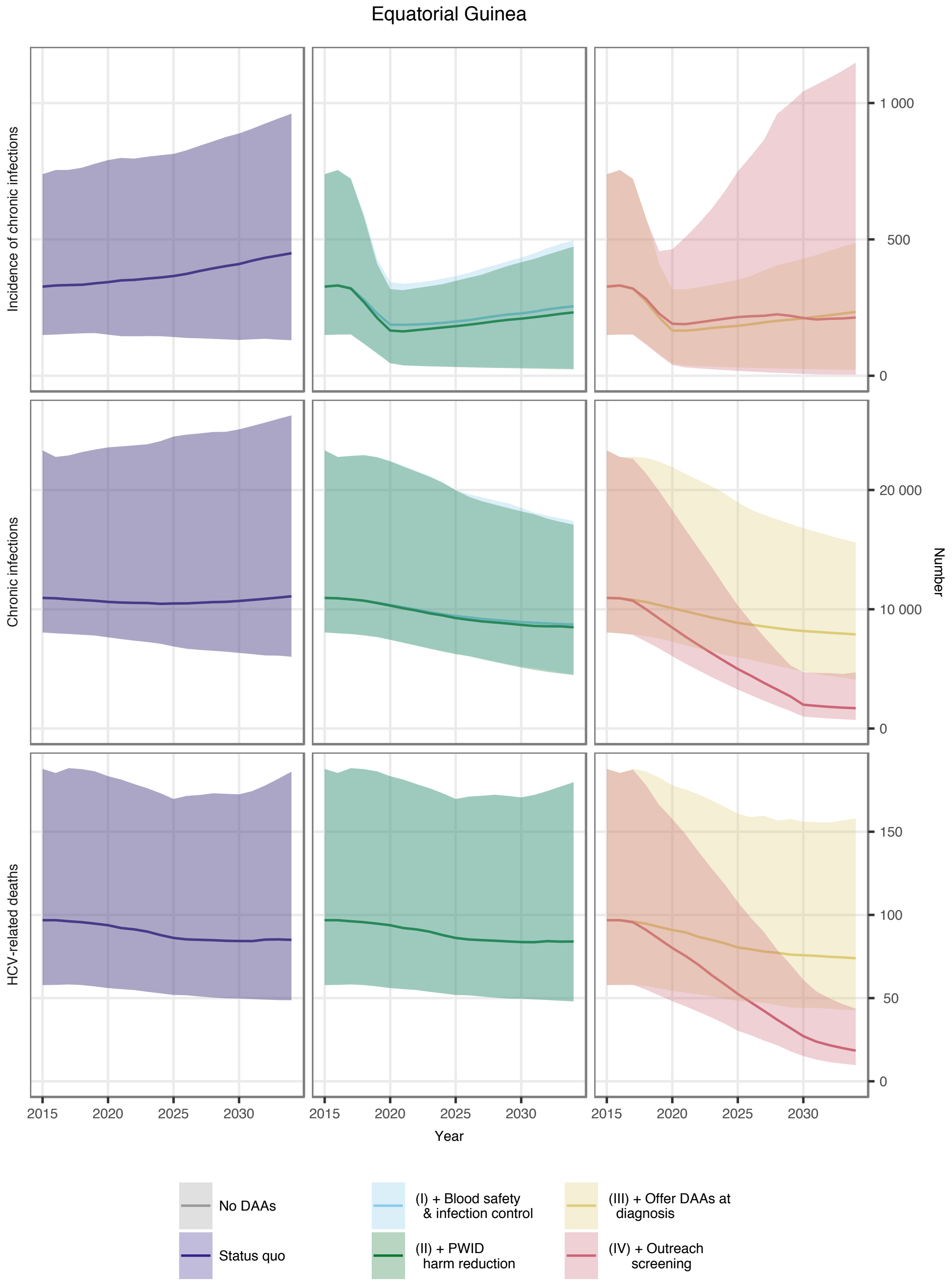
Equatorial Guinea
Incidence of chronic infections
Chronic infections
HCV-related deaths
Number
1 000
500
0
20 000
10 000
0
150
100
50
0
2015
2020
2025
2030
Year
No DAAs
Status quo
(I) + Blood safety & infection control
(II) + PWID harm reduction
(III) + Offer DAAs at diagnosis
(IV) + Outreach screening

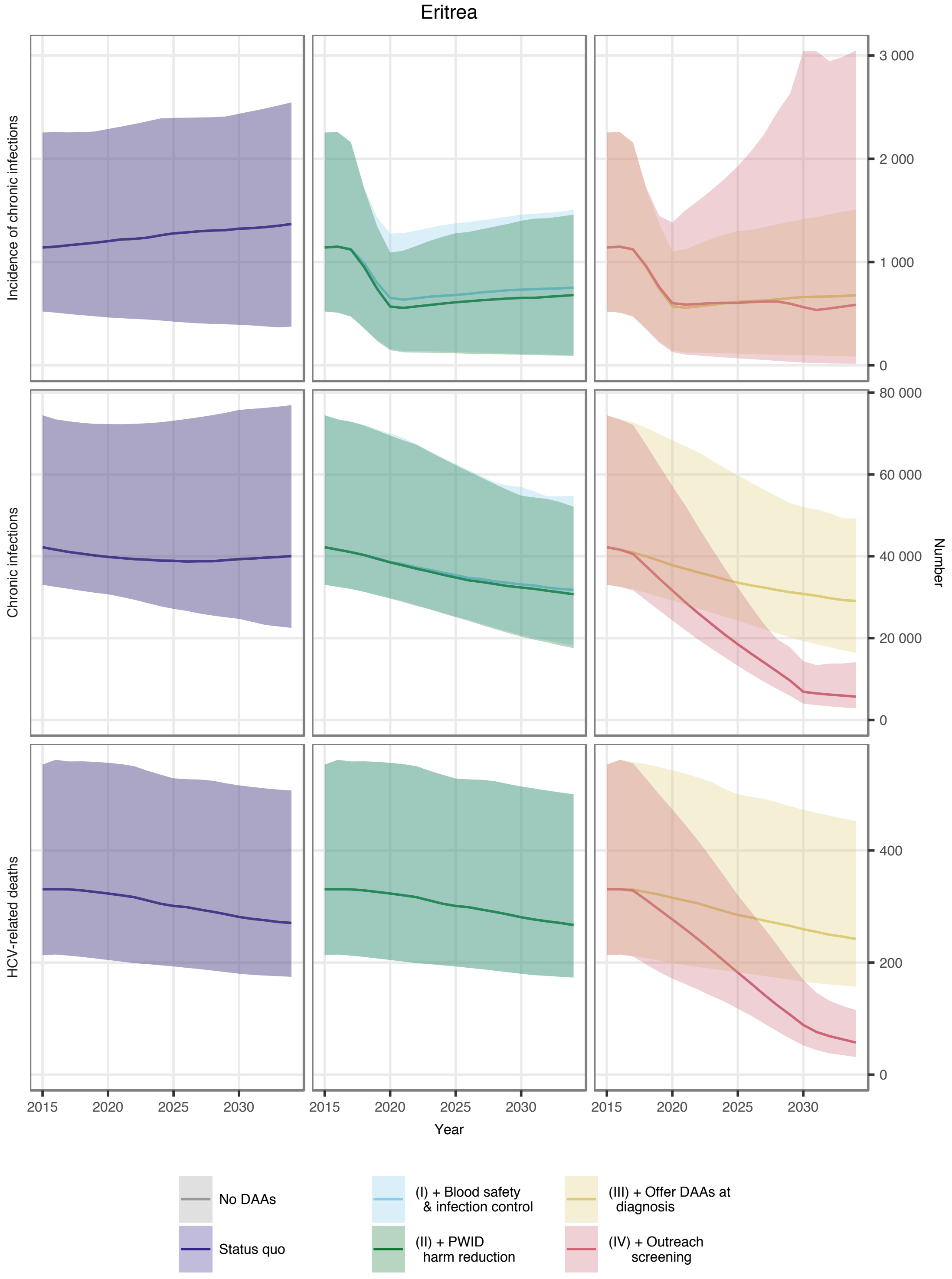
Eritrea
Incidence of chronic infections
Chronic infections
HCV-related deaths
Number
Year
2015
2020
2025
2030
3 000
2 000
1 000
0
80 000
60 000
40 000
20 000
0
400
200
0
No DAAs
Status quo
(I) + Blood safety & infection control
(II) + PWID harm reduction
(III) + Offer DAAs at diagnosis
(IV) + Outreach screening

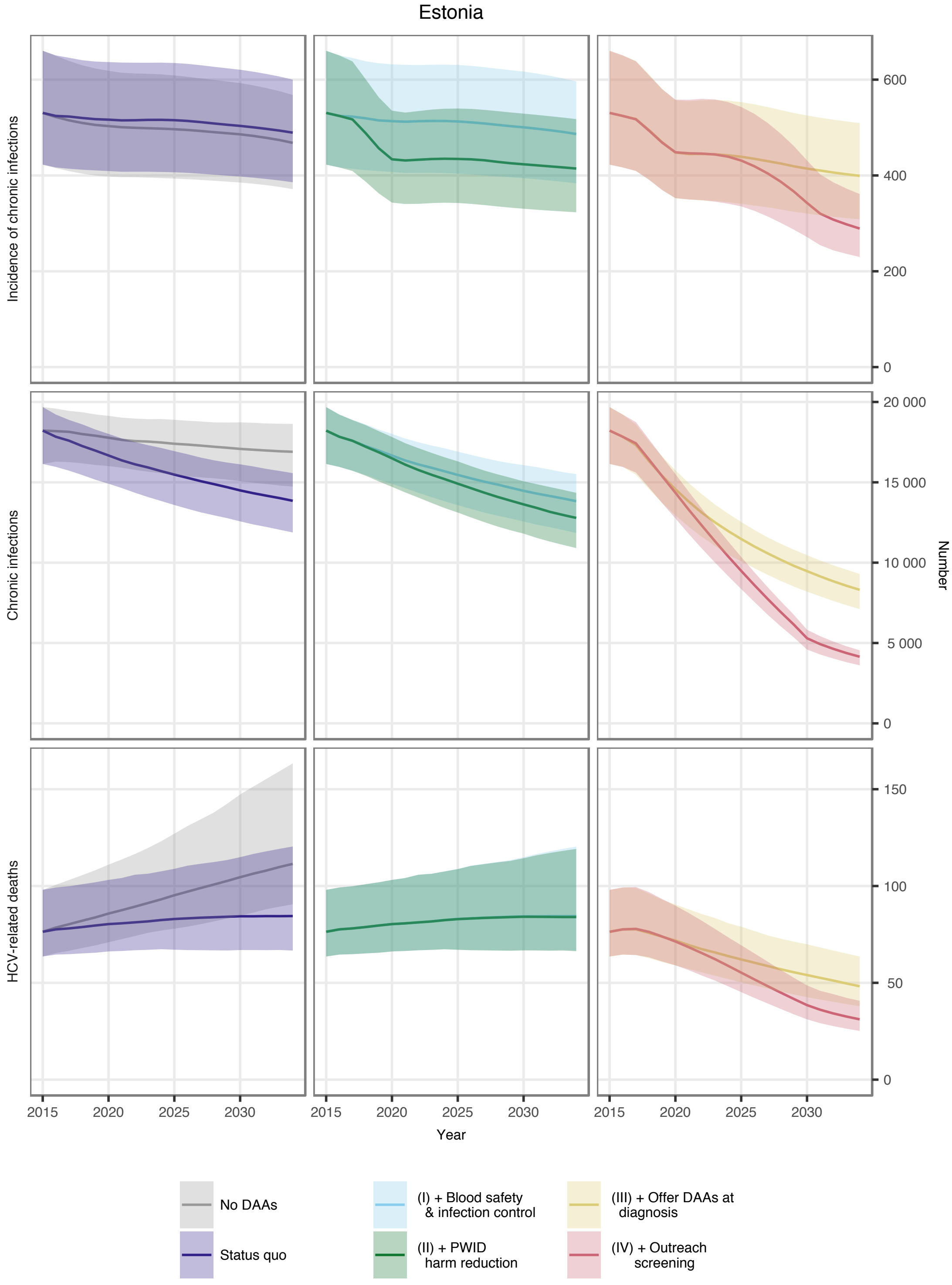
Estonia
Incidence of chronic infections
Chronic infections
HCV-related deaths
Number
Year
600
400
200
0
20 000
15 000
10 000
5 000
0
150
100
50
0
2015
2020
2025
2030
No DAAs
Status quo
(I) + Blood safety & infection control
(II) + PWID harm reduction
(III) + Offer DAAs at diagnosis
(IV) + Outreach screening

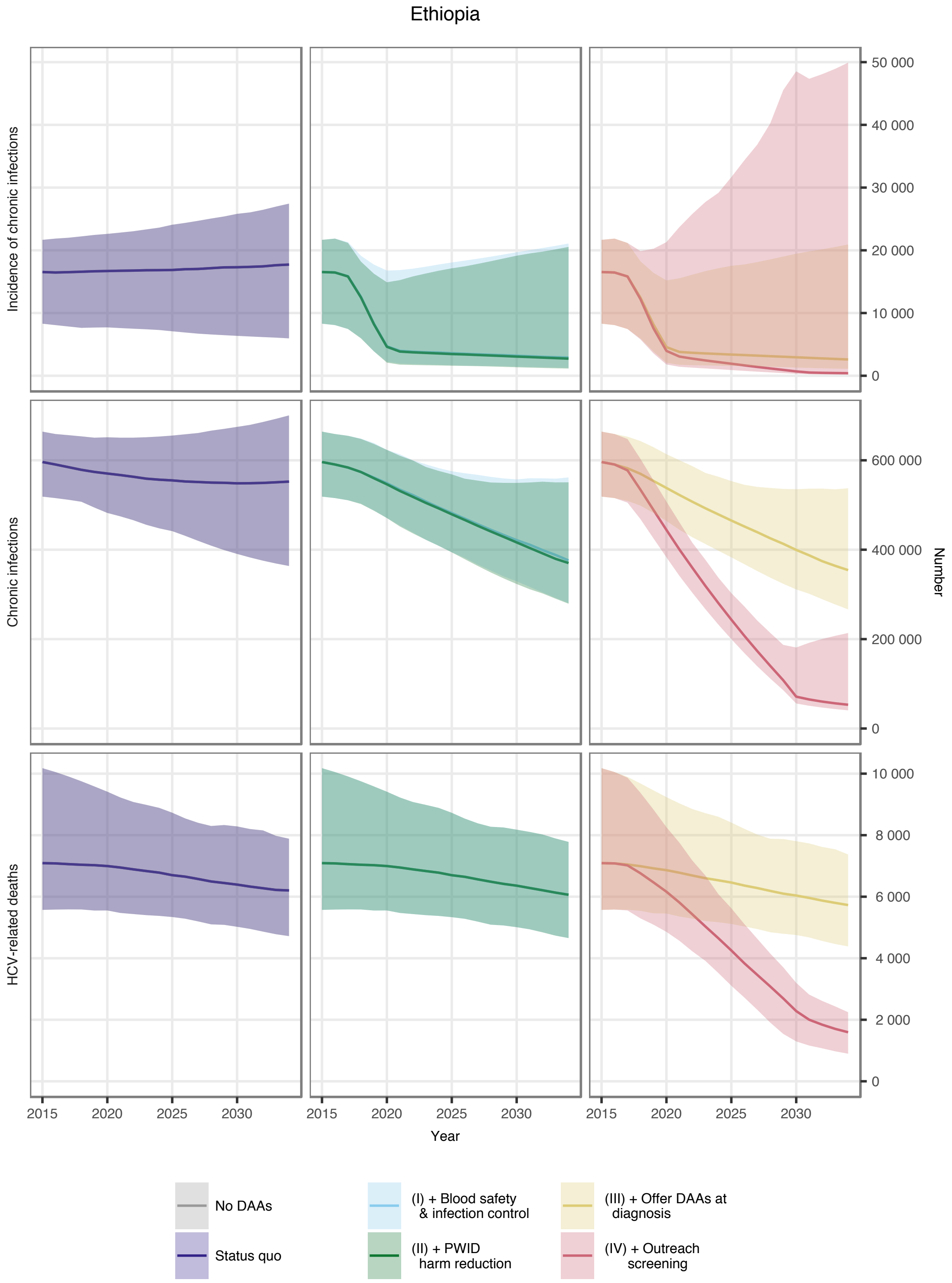

Ethiopia
Incidence of chronic infections
Chronic infections
HCV-related deaths
Number
Year
2015
2020
2025
2030
50 000
40 000
30 000
20 000
10 000
0
600 000
400 000
200 000
0
10 000
8 000
6 000
4 000
2 000
0
No DAAs
Status quo
(I) + Blood safety & infection control
(II) + PWID harm reduction
(III) + Offer DAAs at diagnosis
(IV) + Outreach screening

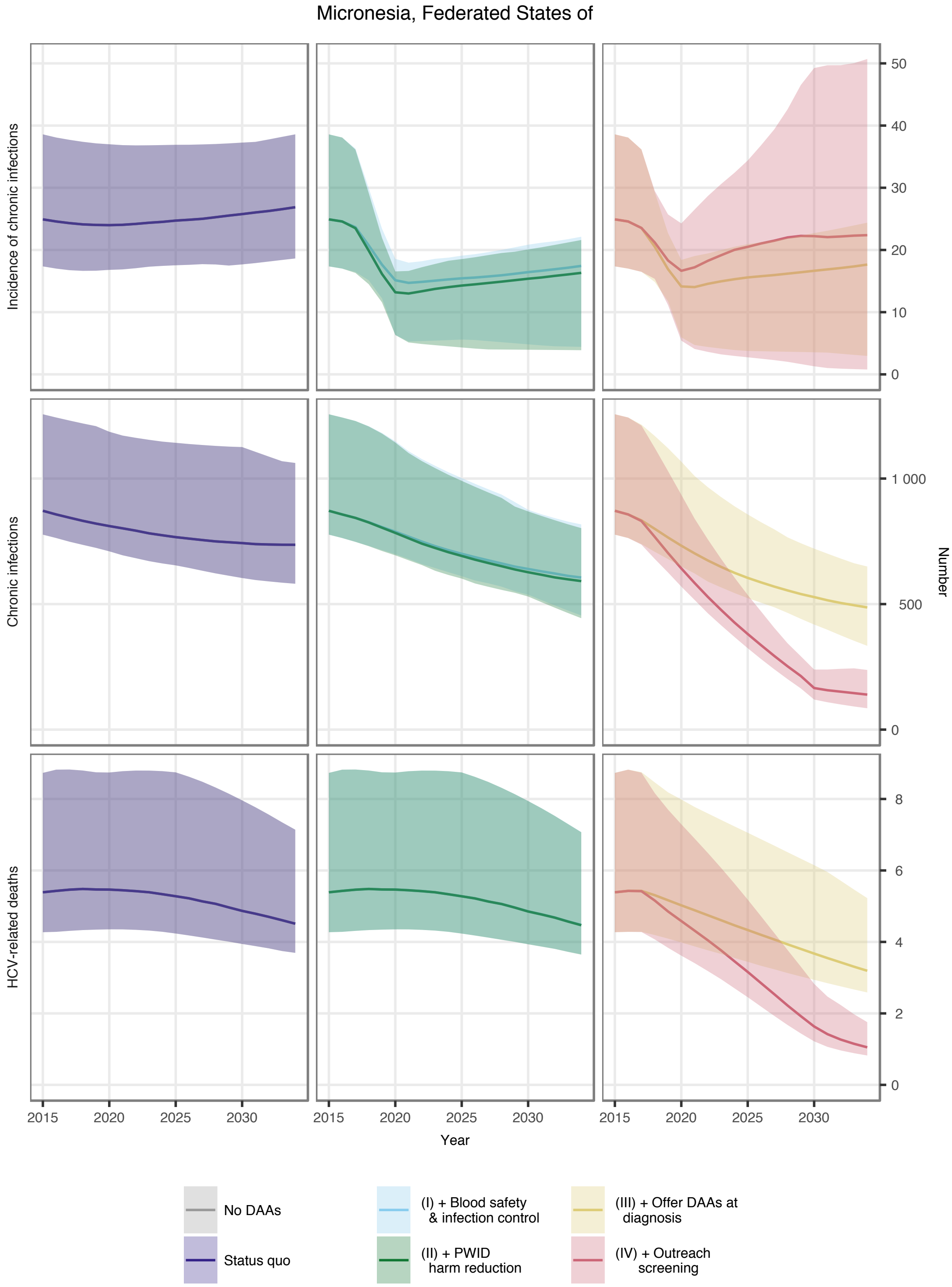

Micronesia, Federated States of
Incidence of chronic infections
Chronic infections
HCV-related deaths
Number
Year
2015
2020
2025
2030
0
10
20
30
40
50
500
1 000
2
4
6
8
No DAAs
Status quo
(I) + Blood safety & infection control
(II) + PWID harm reduction
(III) + Offer DAAs at diagnosis
(IV) + Outreach screening

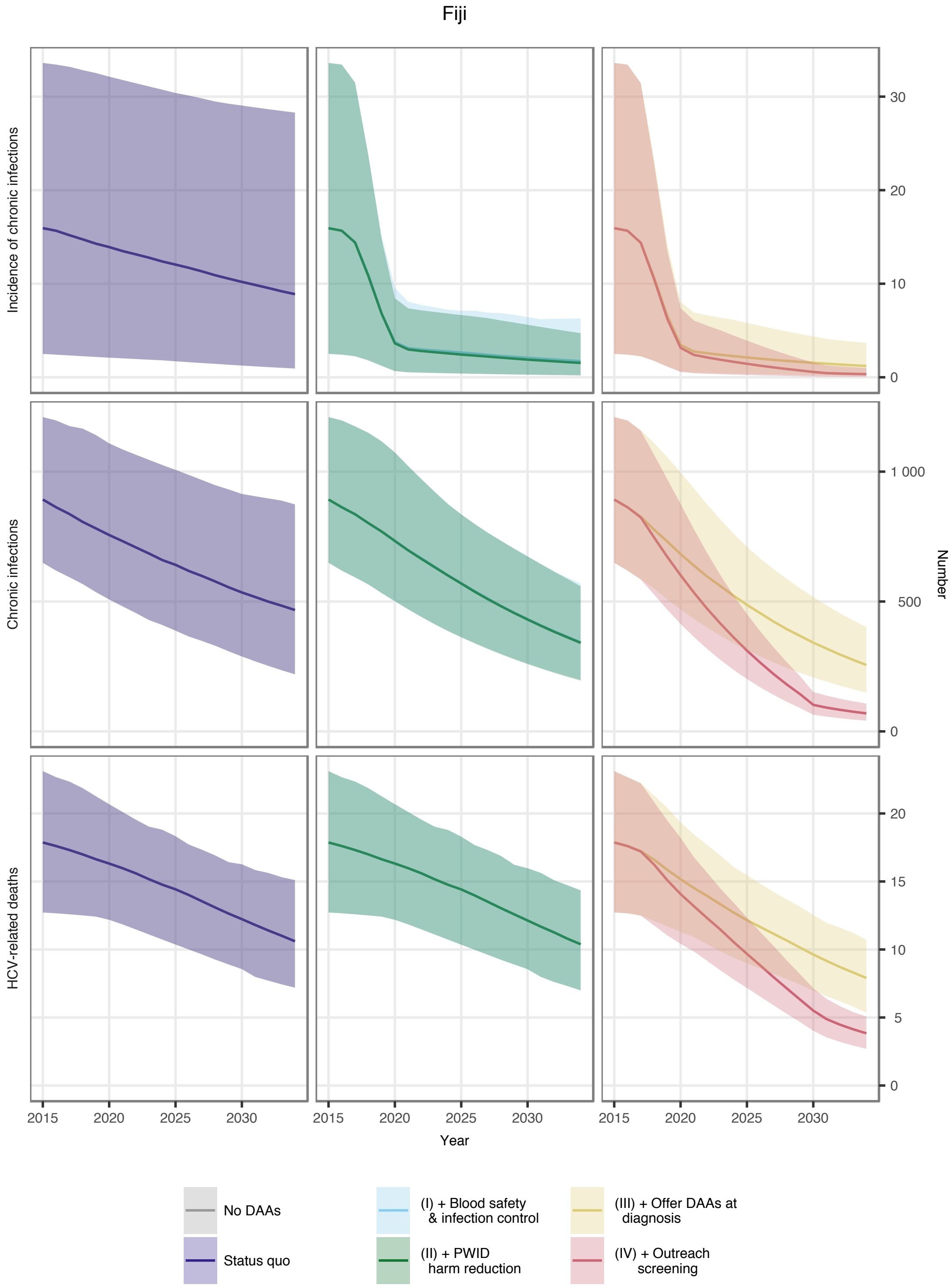
Fiji
Incidence of chronic infections
Chronic infections
HCV-related deaths
Number
Year
2015
2020
2025
2030
0
10
20
30
500
1 000
5
15
No DAAs
Status quo
(I) + Blood safety & infection control
(II) + PWID harm reduction
(III) + Offer DAAs at diagnosis
(IV) + Outreach screening

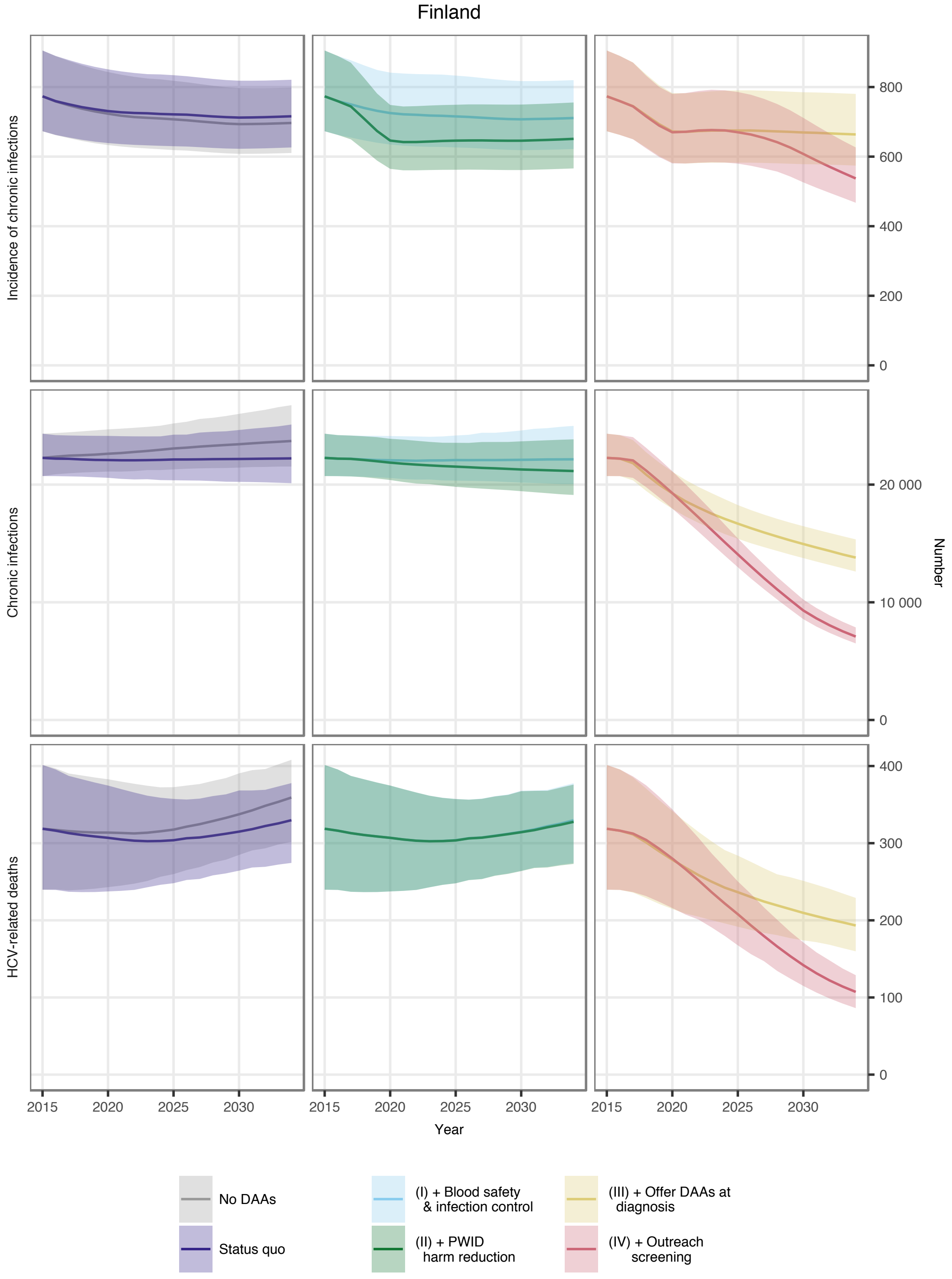

Finland
Incidence of chronic infections
Chronic infections
HCV-related deaths
800
600
400
200
0
20 000
10 000
0
400
300
200
100
0
Number
2015
2020
2025
2030
Year
No DAAs
Status quo
(I) + Blood safety & infection control
(II) + PWID harm reduction
(III) + Offer DAAs at diagnosis
(IV) + Outreach screening

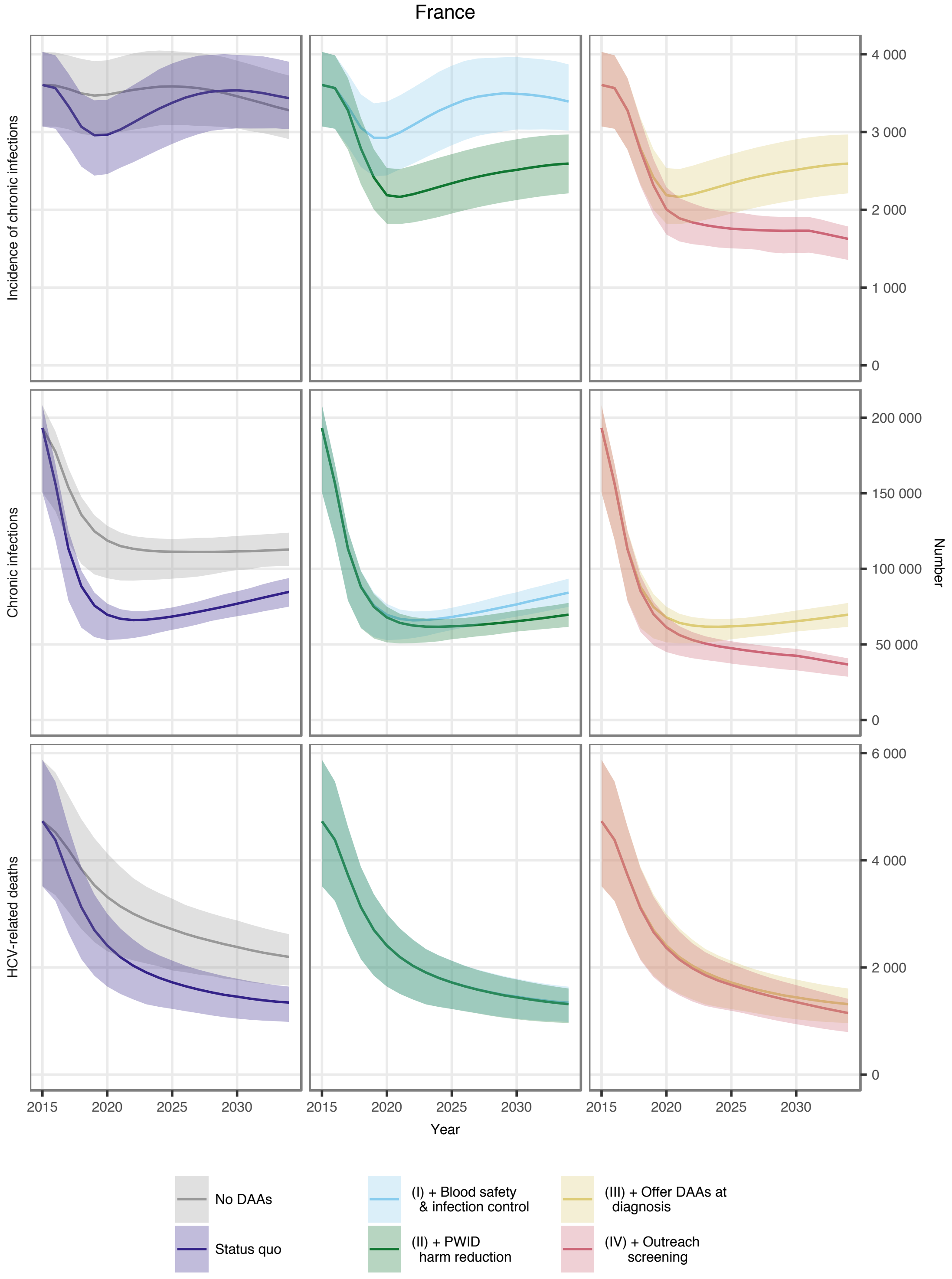

France
Incidence of chronic infections
Chronic infections
HCV-related deaths
Number
Year
4 000
3 000
2 000
1 000
0
200 000
150 000
100 000
50 000
0
6 000
4 000
2 000
0
2015
2020
2025
2030
No DAAs
Status quo
(I) + Blood safety & infection control
(II) + PWID harm reduction
(III) + Offer DAAs at diagnosis
(IV) + Outreach screening

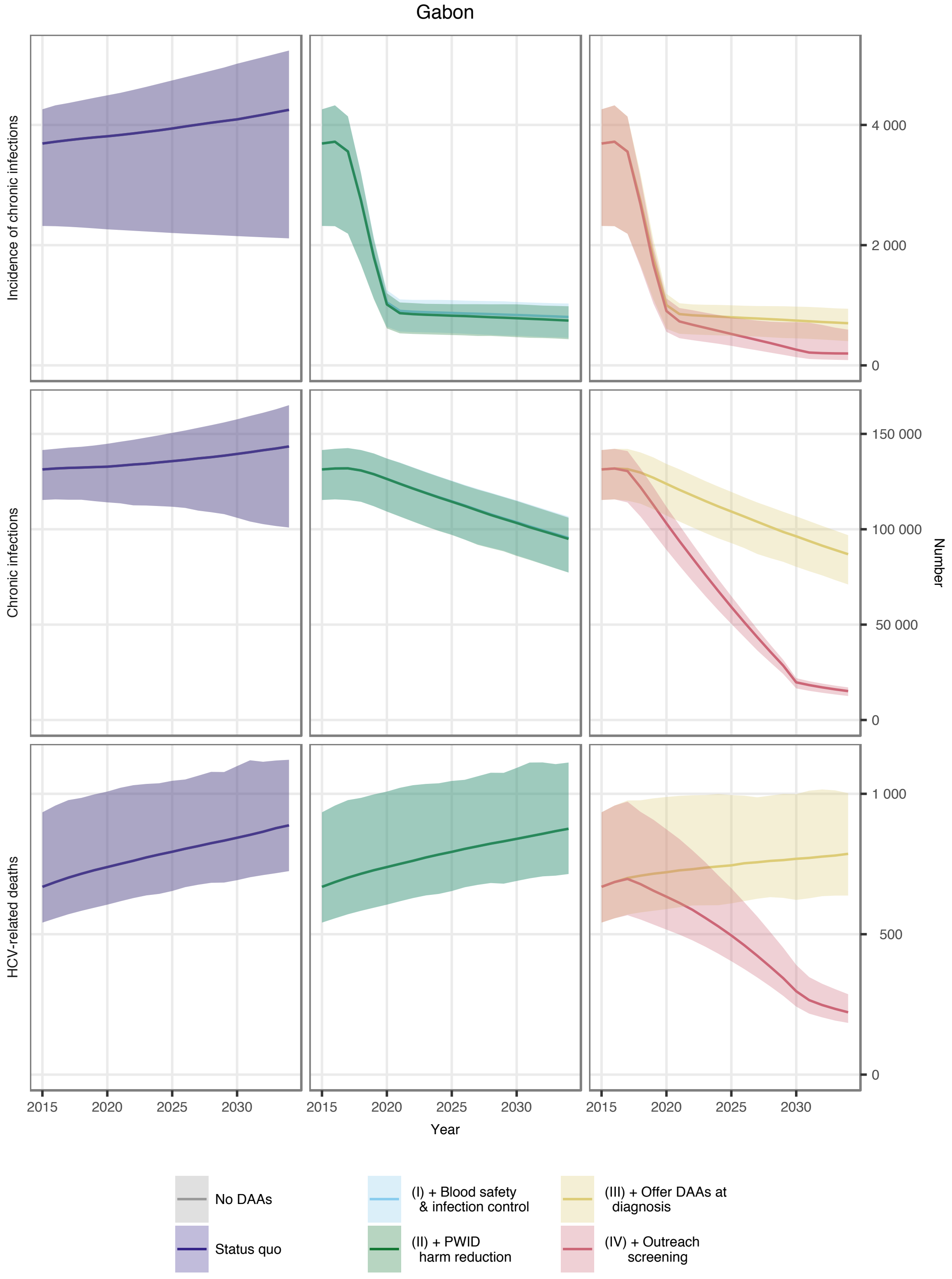

Gabon
Incidence of chronic infections
Chronic infections
HCV-related deaths
Number
4 000
2 000
0
150 000
100 000
50 000
0
1 000
500
0
2015
2020
2025
2030
Year
No DAAs
Status quo
(I) + Blood safety & infection control
(II) + PWID harm reduction
(III) + Offer DAAs at diagnosis
(IV) + Outreach screening

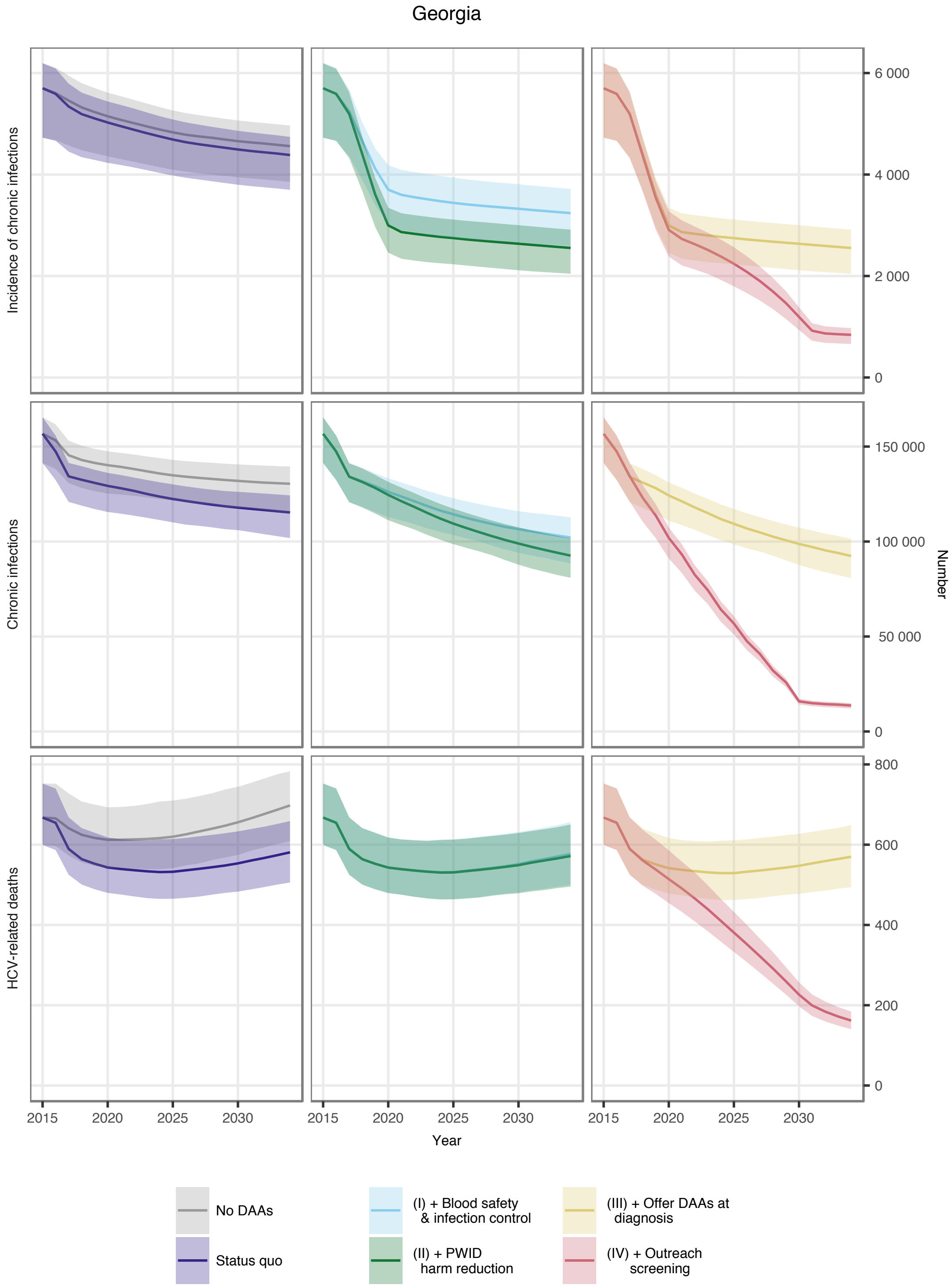

Georgia
Incidence of chronic infections
Chronic infections
HCV-related deaths
Number
6 000
4 000
2 000
0
150 000
100 000
50 000
0
800
600
400
200
0
2015
2020
2025
2030
Year
No DAAs
Status quo
(I) + Blood safety & infection control
(II) + PWID harm reduction
(III) + Offer DAAs at diagnosis
(IV) + Outreach screening

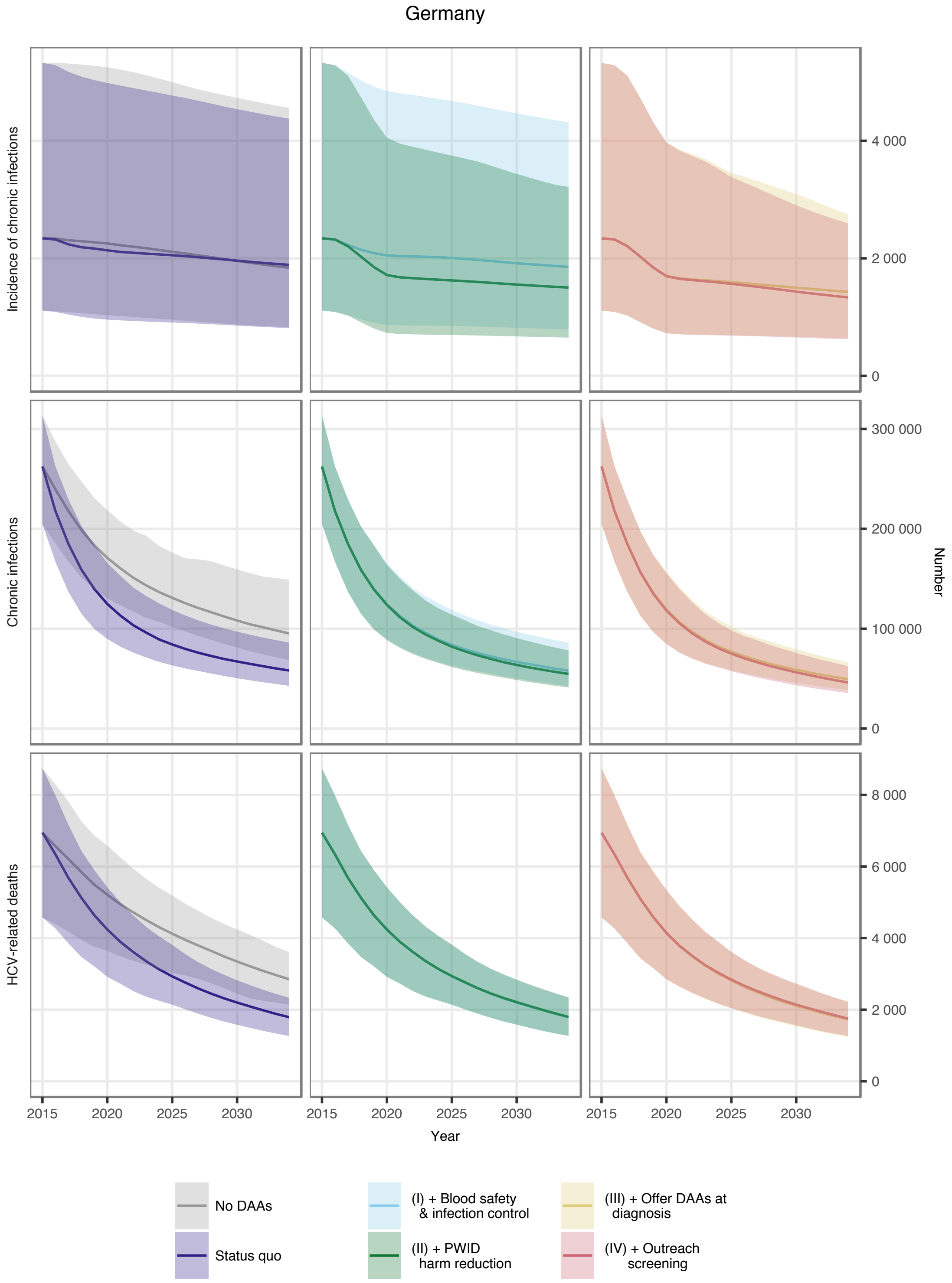

Germany
Incidence of chronic infections
Chronic infections
HCV-related deaths
Number
0
2 000
4 000
100 000
200 000
300 000
6 000
8 000
2015
2020
2025
2030
Year
No DAAs
Status quo
(I) + Blood safety & infection control
(II) + PWID harm reduction
(III) + Offer DAAs at diagnosis
(IV) + Outreach screening

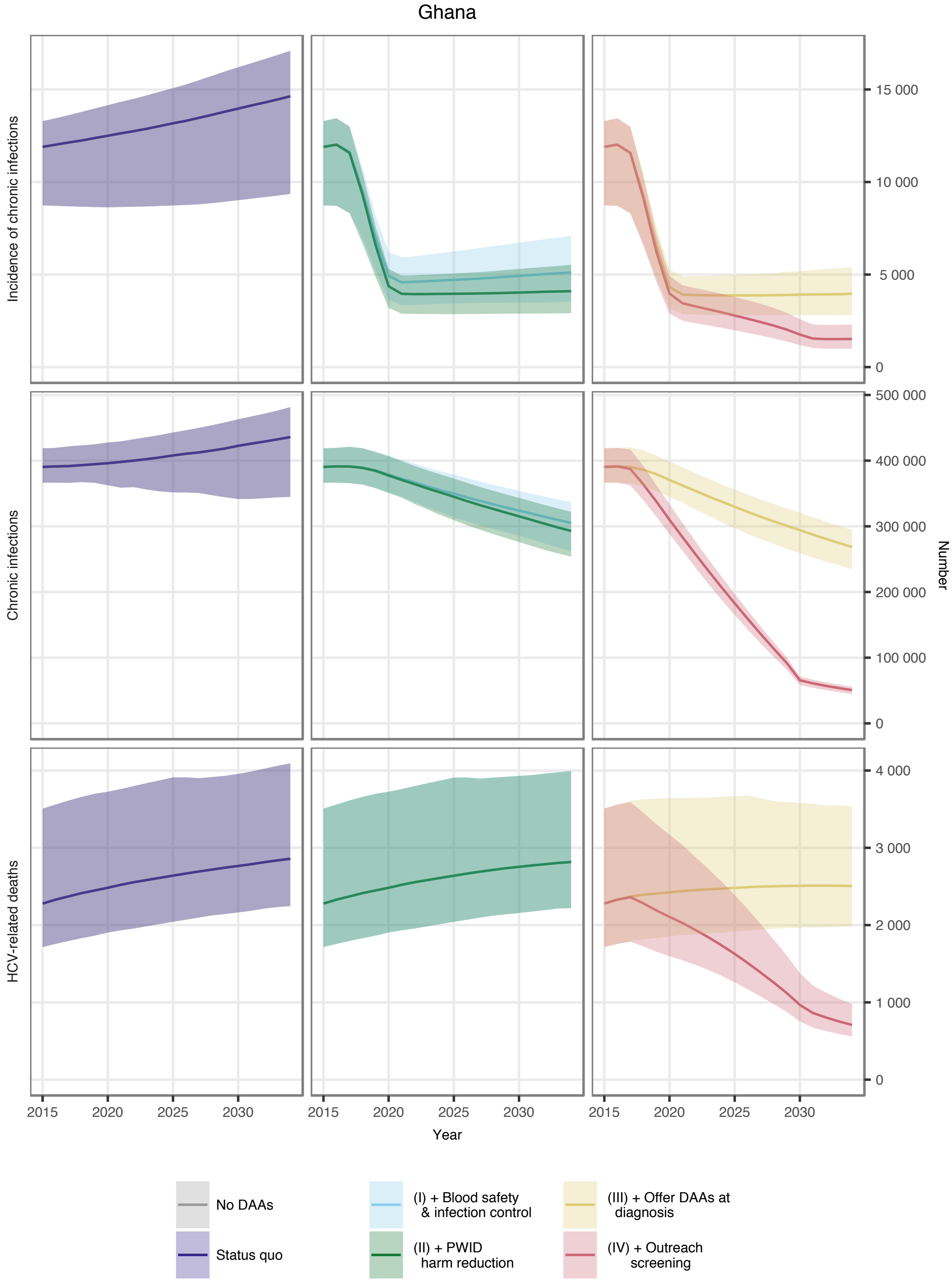
Ghana
Incidence of chronic infections
Chronic infections
HCV-related deaths
Number
Year
15 000
10 000
5 000
0
500 000
400 000
300 000
200 000
100 000
0
4 000
3 000
2 000
1 000
0
2015
2020
2025
2030
No DAAs
Status quo
(I) + Blood safety & infection control
(II) + PWID harm reduction
(III) + Offer DAAs at diagnosis
(IV) + Outreach screening

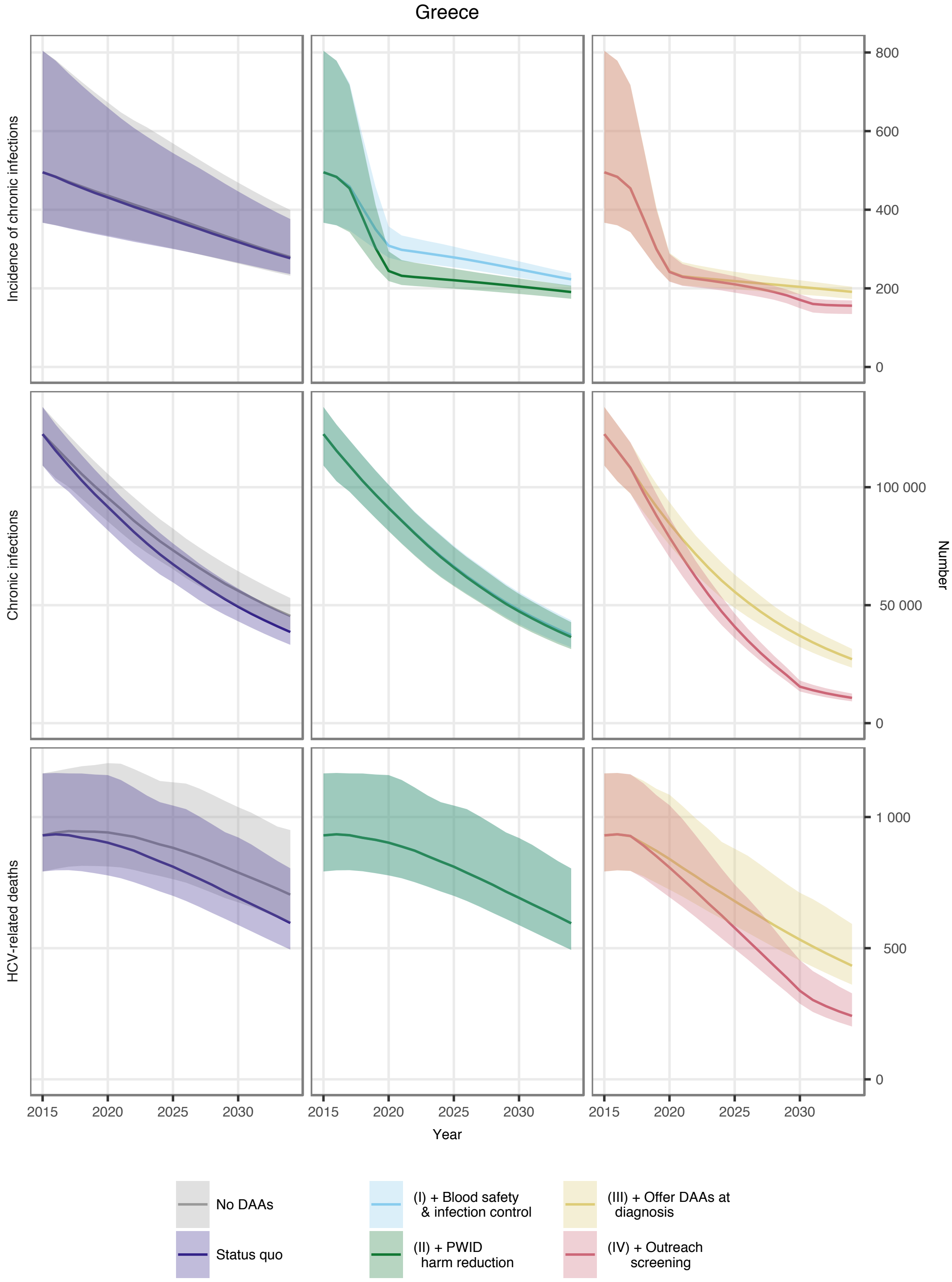
Greece
Incidence of chronic infections
Chronic infections
HCV-related deaths
Number
Year
2015
2020
2025
2030
0
200
400
600
800
50 000
100 000
500
1 000
No DAAs
Status quo
(I) + Blood safety & infection control
(II) + PWID harm reduction
(III) + Offer DAAs at diagnosis
(IV) + Outreach screening

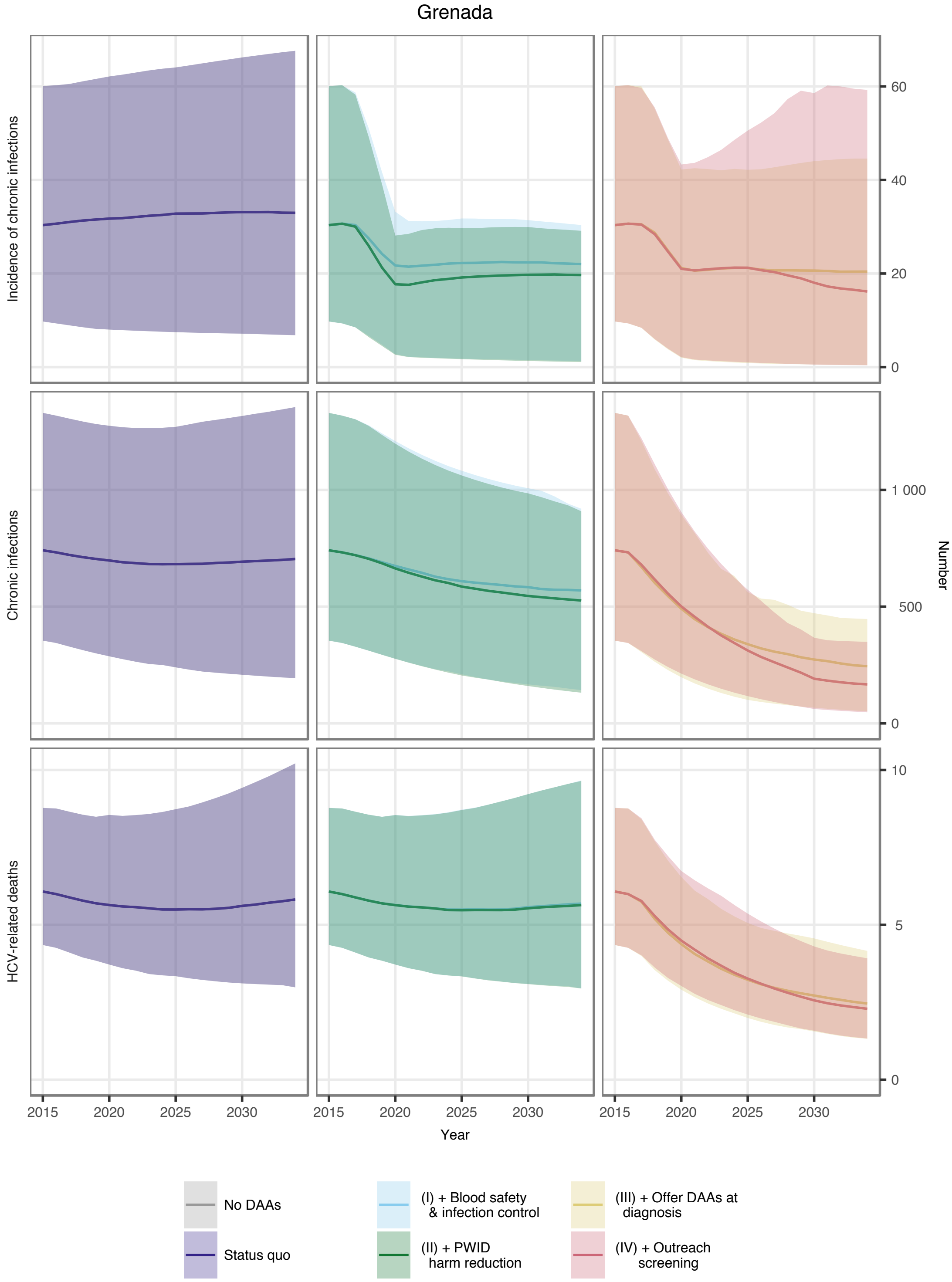
Grenada
Incidence of chronic infections
Chronic infections
HCV-related deaths
Number
60
40
20
0
1 000
500
0
10
5
0
2015
2020
2025
2030
Year
No DAAs
Status quo
(I) + Blood safety & infection control
(II) + PWID harm reduction
(III) + Offer DAAs at diagnosis
(IV) + Outreach screening

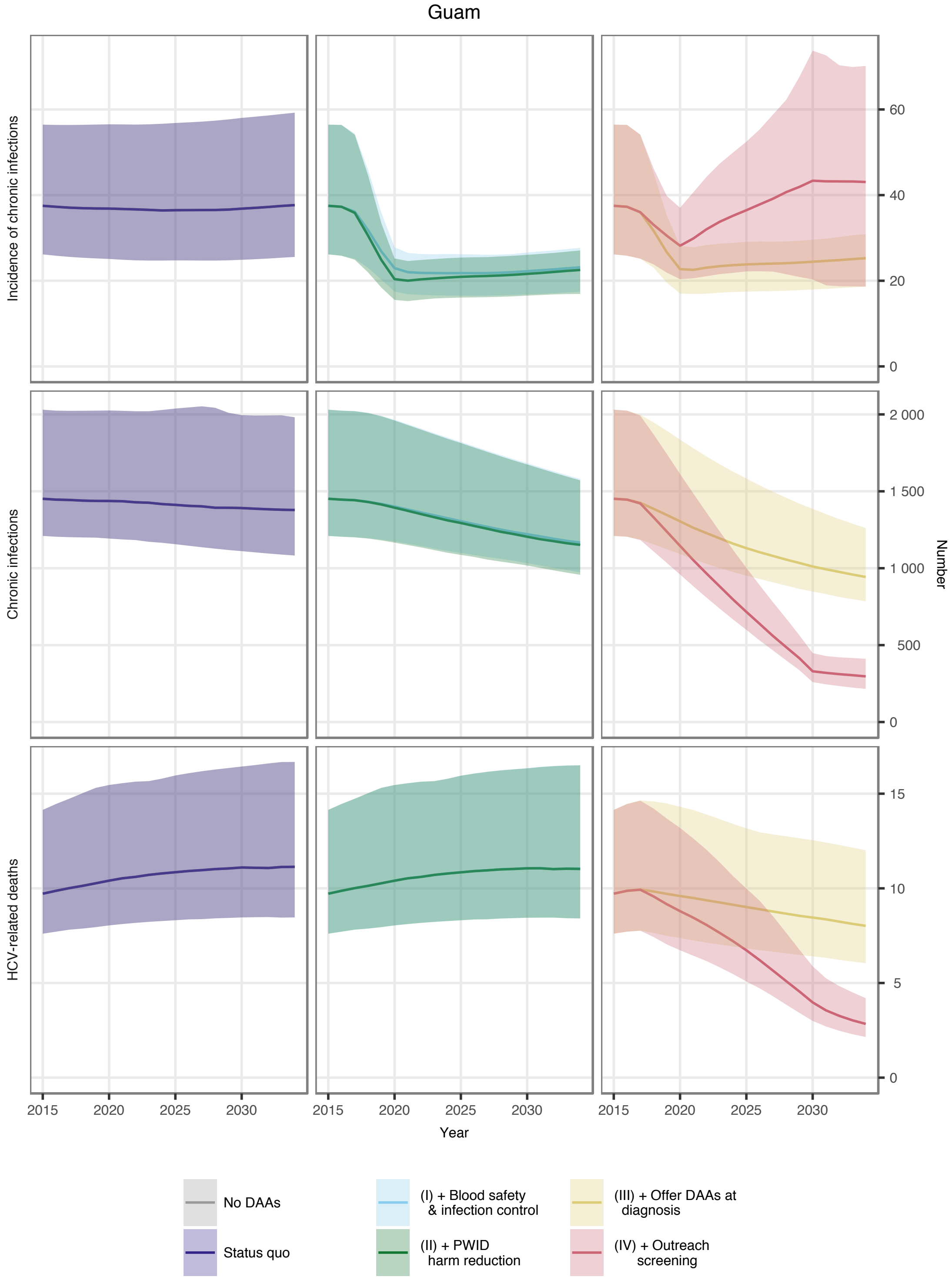
Guam
Incidence of chronic infections
Chronic infections
HCV-related deaths
Number
60
40
20
0
2 000
1 500
1 000
500
0
15
10
5
0
2015
2020
2025
2030
Year
No DAAs
Status quo
(I) + Blood safety & infection control
(II) + PWID harm reduction
(III) + Offer DAAs at diagnosis
(IV) + Outreach screening

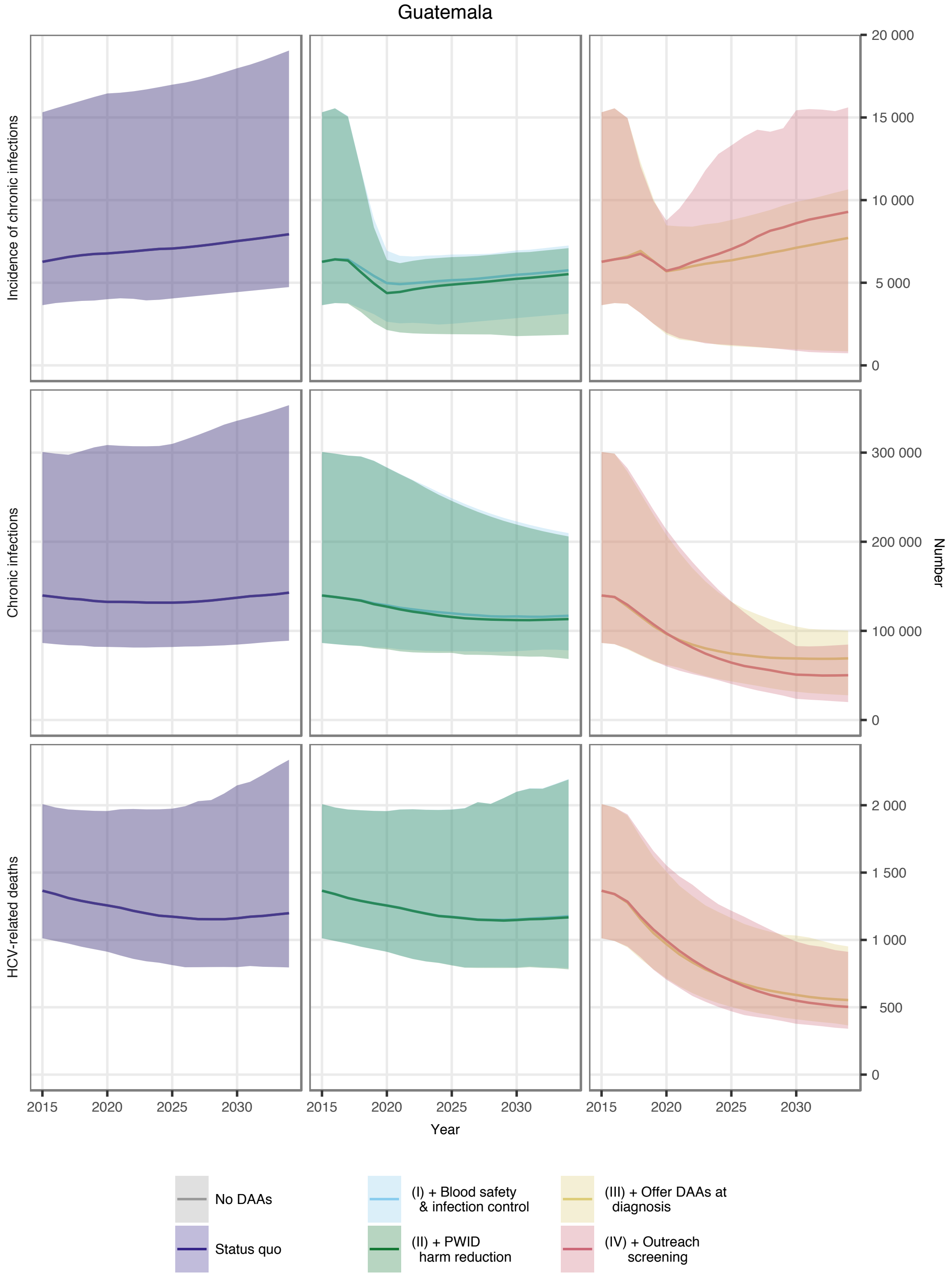
Guatemala
Incidence of chronic infections
Chronic infections
HCV-related deaths
Number
20 000
15 000
10 000
5 000
0
300 000
200 000
100 000
0
2 000
1 500
1 000
500
0
2015
2020
2025
2030
Year
No DAAs
Status quo
(I) + Blood safety & infection control
(II) + PWID harm reduction
(III) + Offer DAAs at diagnosis
(IV) + Outreach screening

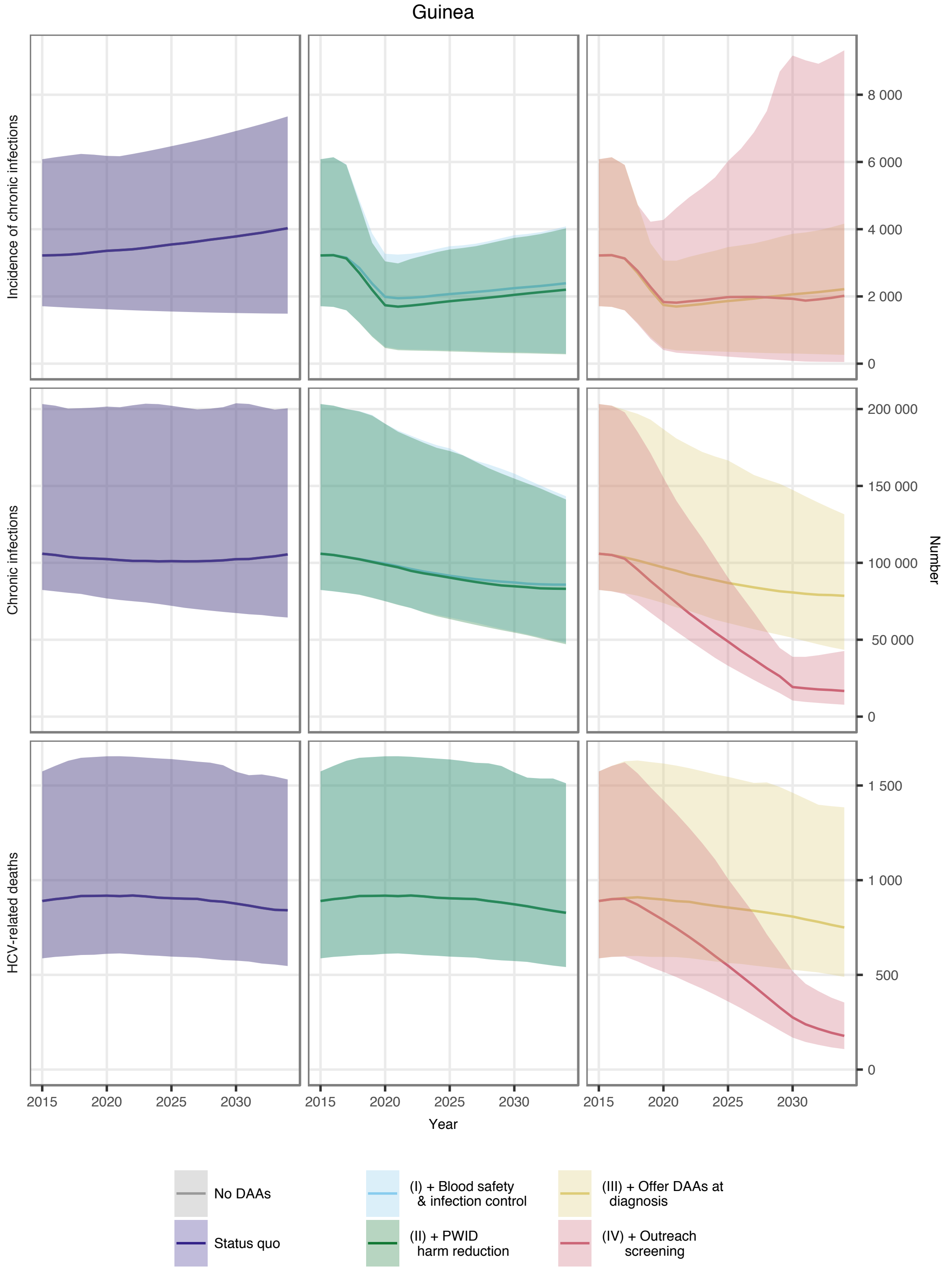

Guinea
Incidence of chronic infections
Chronic infections
HCV-related deaths
Number
Year
0
2 000
4 000
6 000
8 000
0
50 000
100 000
150 000
200 000
0
500
1 000
1 500
2015
2020
2025
2030
No DAAs
Status quo
(I) + Blood safety & infection control
(II) + PWID harm reduction
(III) + Offer DAAs at diagnosis
(IV) + Outreach screening

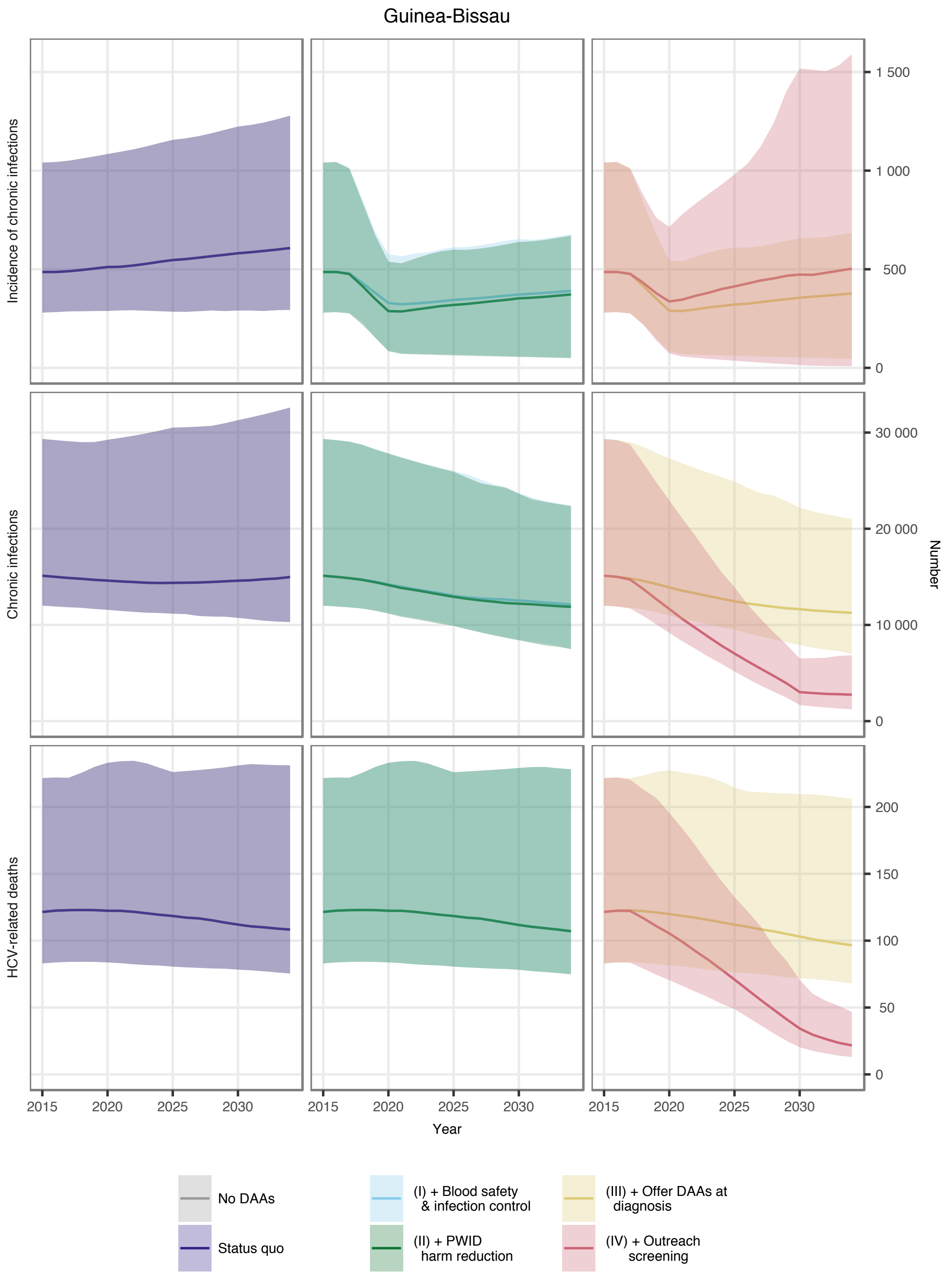

Guinea-Bissau
Incidence of chronic infections
Chronic infections
HCV-related deaths
Number
1 500
1 000
500
0
30 000
20 000
10 000
0
200
150
100
50
0
2015
2020
2025
2030
Year
No DAAs
Status quo
(I) + Blood safety & infection control
(II) + PWID harm reduction
(III) + Offer DAAs at diagnosis
(IV) + Outreach screening

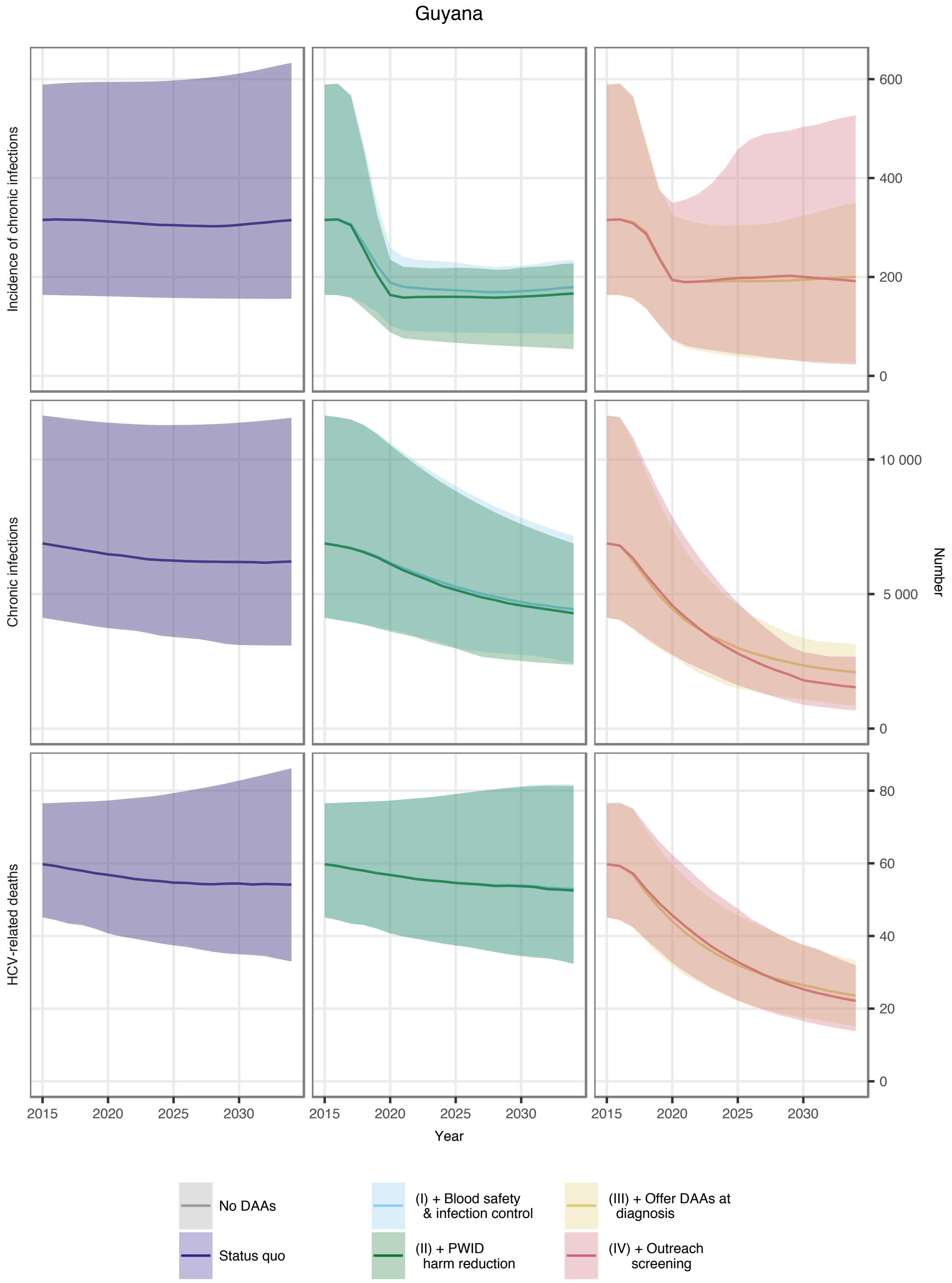

Guyana
Incidence of chronic infections
Chronic infections
HCV-related deaths
Number
600
400
200
0
10 000
5 000
0
80
60
40
20
0
2015
2020
2025
2030
Year
No DAAs
Status quo
(I) + Blood safety & infection control
(II) + PWID harm reduction
(III) + Offer DAAs at diagnosis
(IV) + Outreach screening

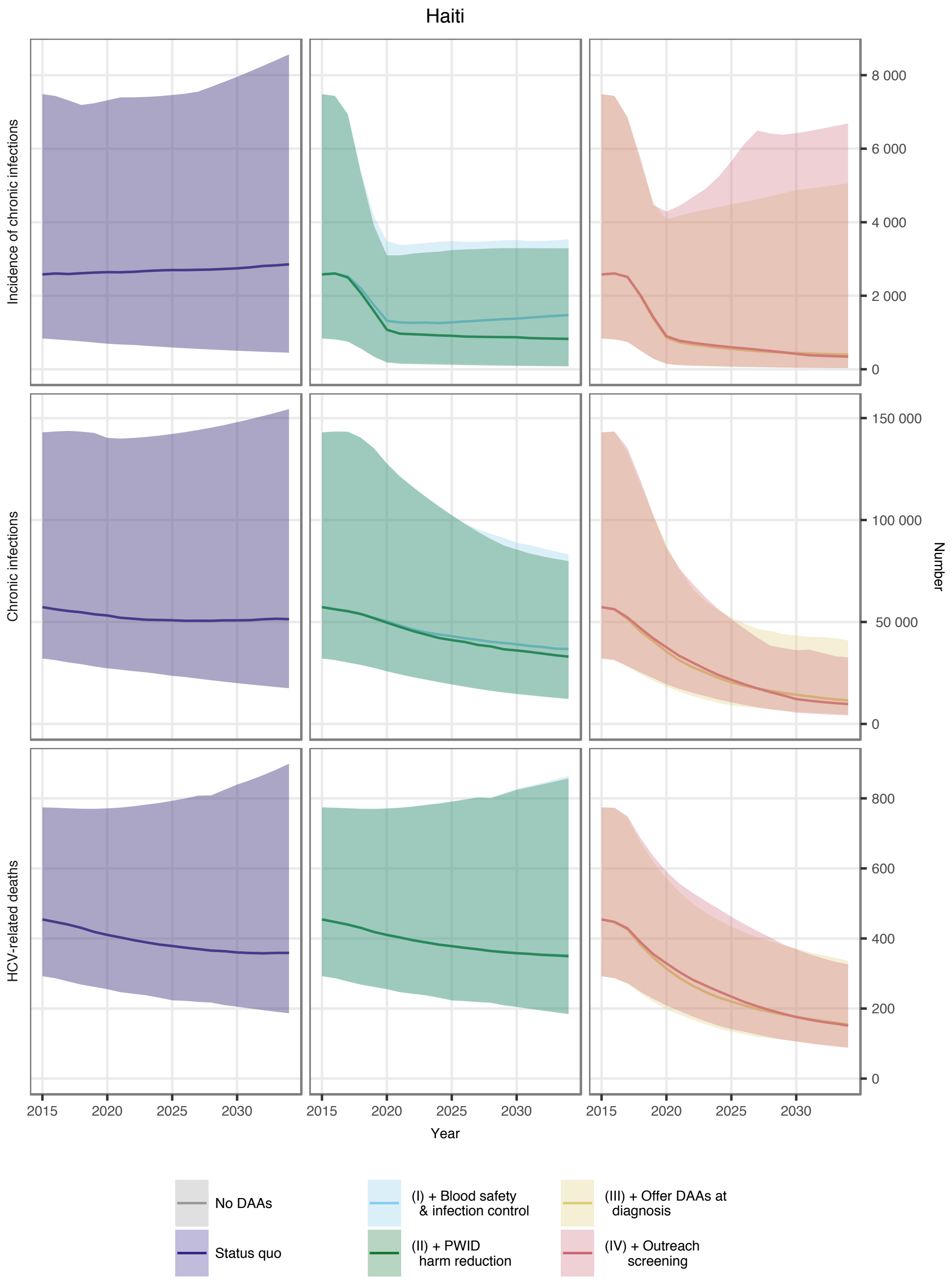

Haiti
Incidence of chronic infections
Chronic infections
HCV-related deaths
8 000
6 000
4 000
2 000
0
150 000
100 000
50 000
0
800
600
400
200
0
Number
2015
2020
2025
2030
Year
No DAAs
Status quo
(I) + Blood safety & infection control
(II) + PWID harm reduction
(III) + Offer DAAs at diagnosis
(IV) + Outreach screening

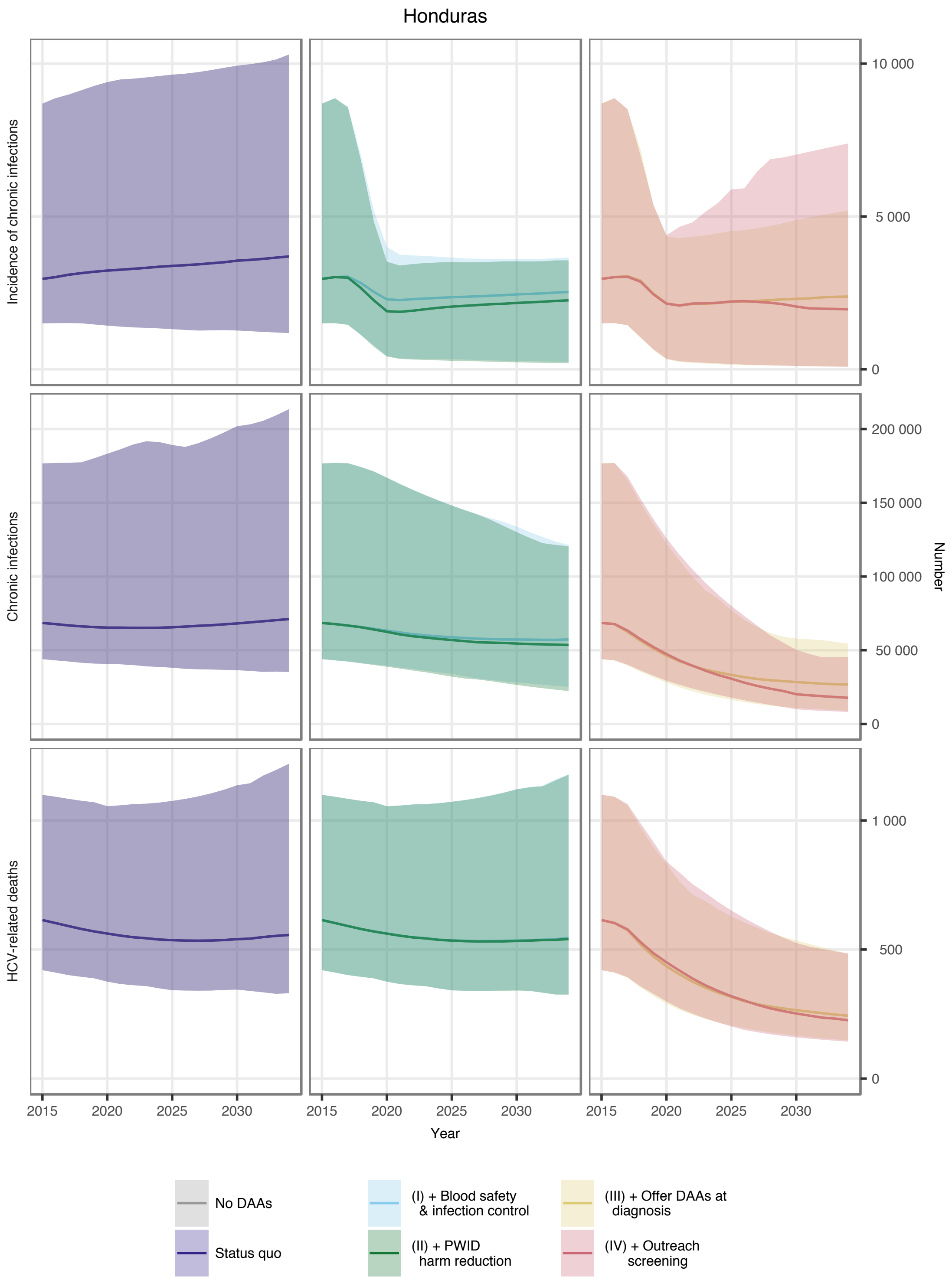
Honduras
Incidence of chronic infections
Chronic infections
HCV-related deaths
Number
10 000
5 000
0
200 000
150 000
100 000
50 000
0
1 000
500
0
2015
2020
2025
2030
Year
No DAAs
Status quo
(I) + Blood safety & infection control
(II) + PWID harm reduction
(III) + Offer DAAs at diagnosis
(IV) + Outreach screening

Hong Kong Special Administrative Region of China

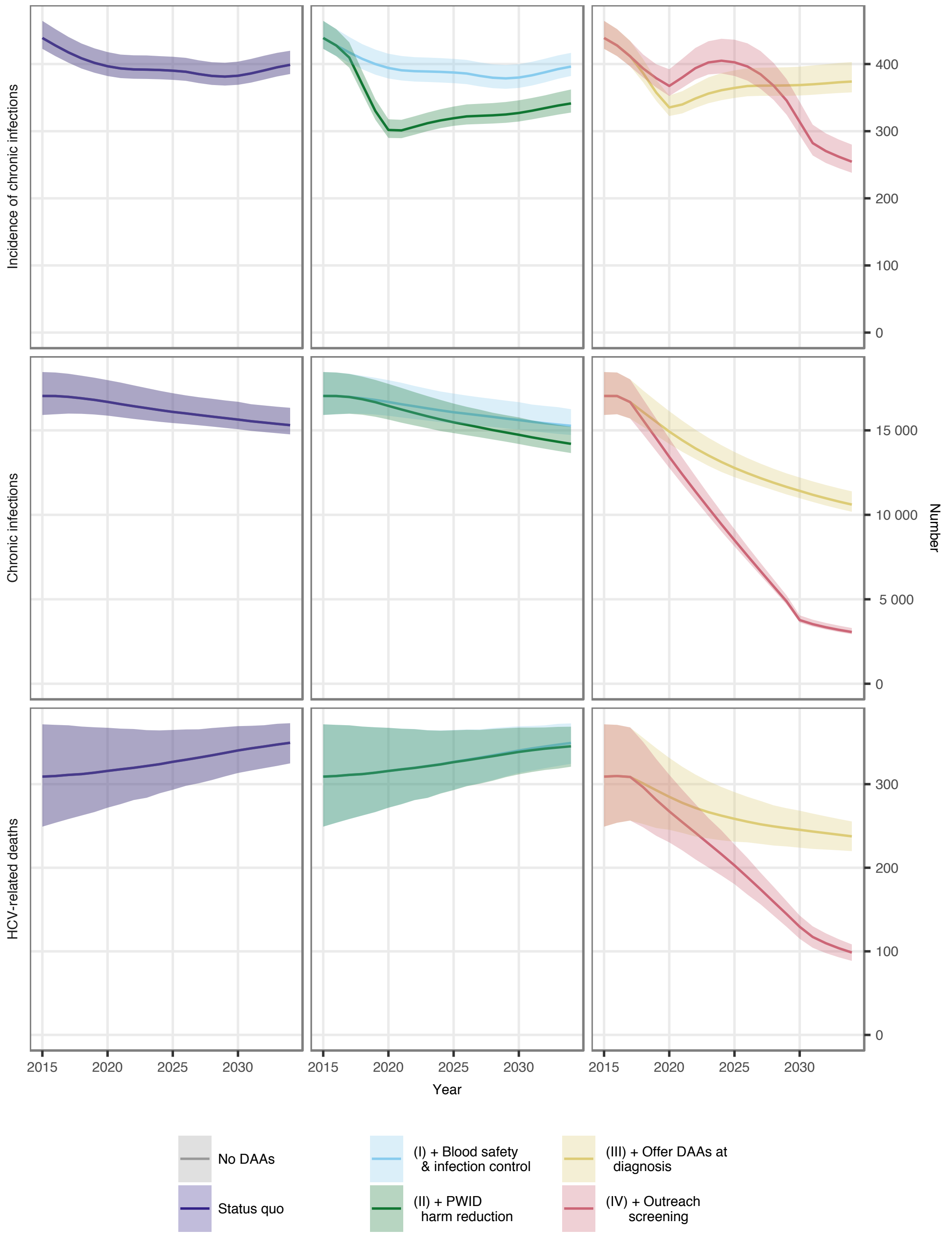

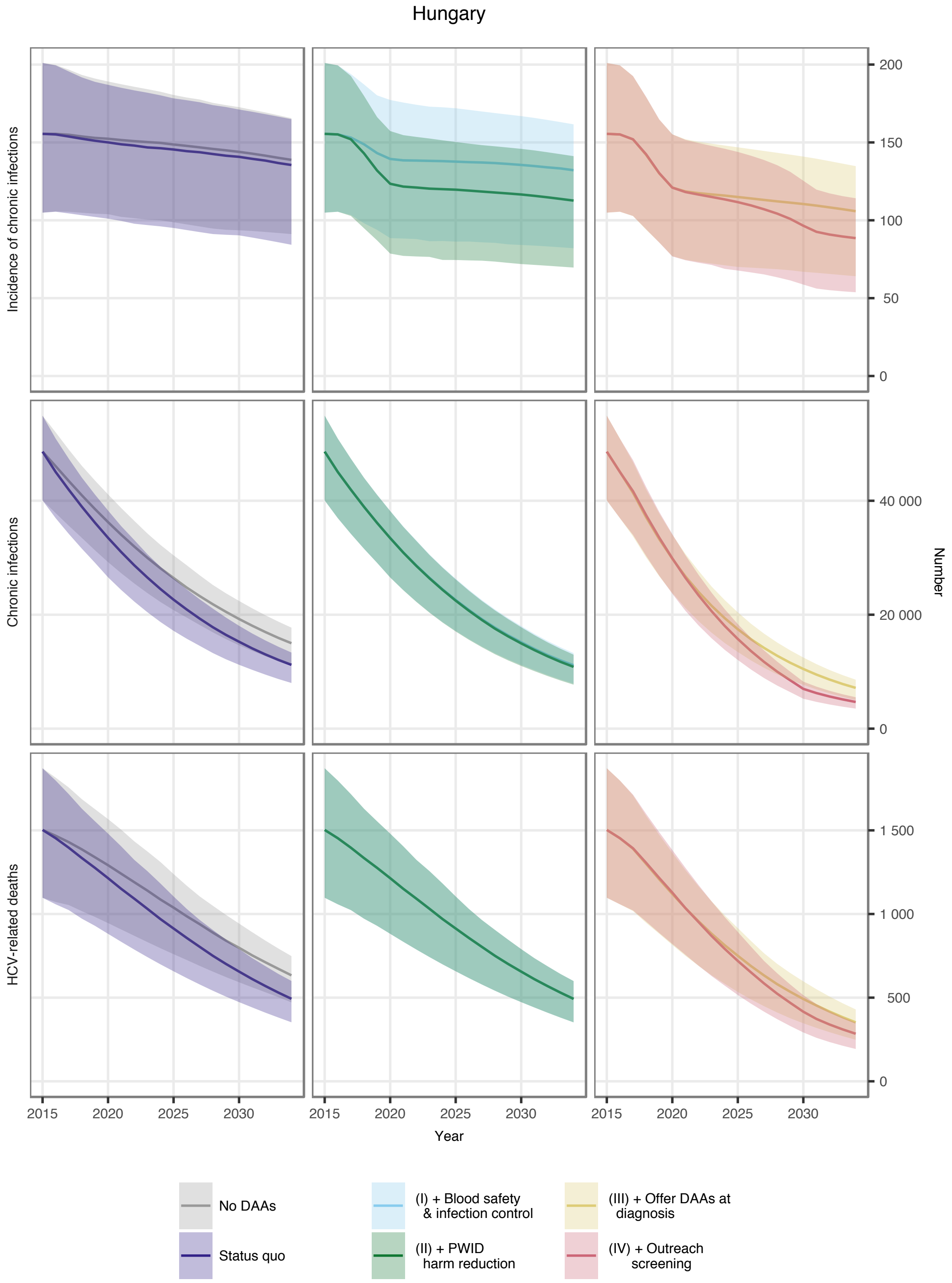
Hungary
Incidence of chronic infections
Chronic infections
HCV-related deaths
Number
Year
200
150
100
50
0
40 000
20 000
0
1 500
1 000
500
0
2015
2020
2025
2030
No DAAs
Status quo
(I) + Blood safety & infection control
(II) + PWID harm reduction
(III) + Offer DAAs at diagnosis
(IV) + Outreach screening

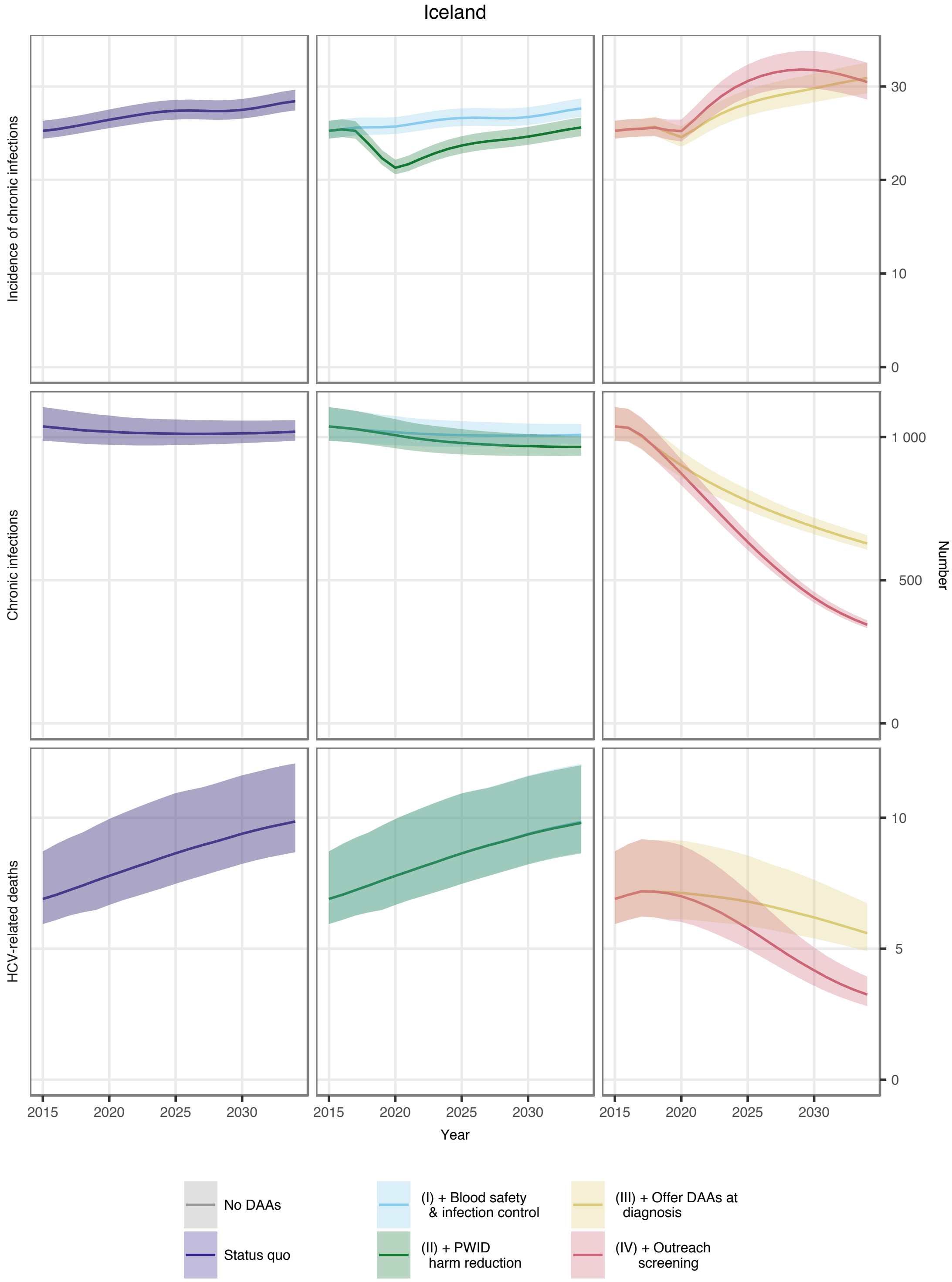
Iceland
Incidence of chronic infections
Chronic infections
HCV-related deaths
Number
Year
2015
2020
2025
2030
0
10
20
30
500
1 000
5
No DAAs
Status quo
(I) + Blood safety & infection control
(II) + PWID harm reduction
(III) + Offer DAAs at diagnosis
(IV) + Outreach screening

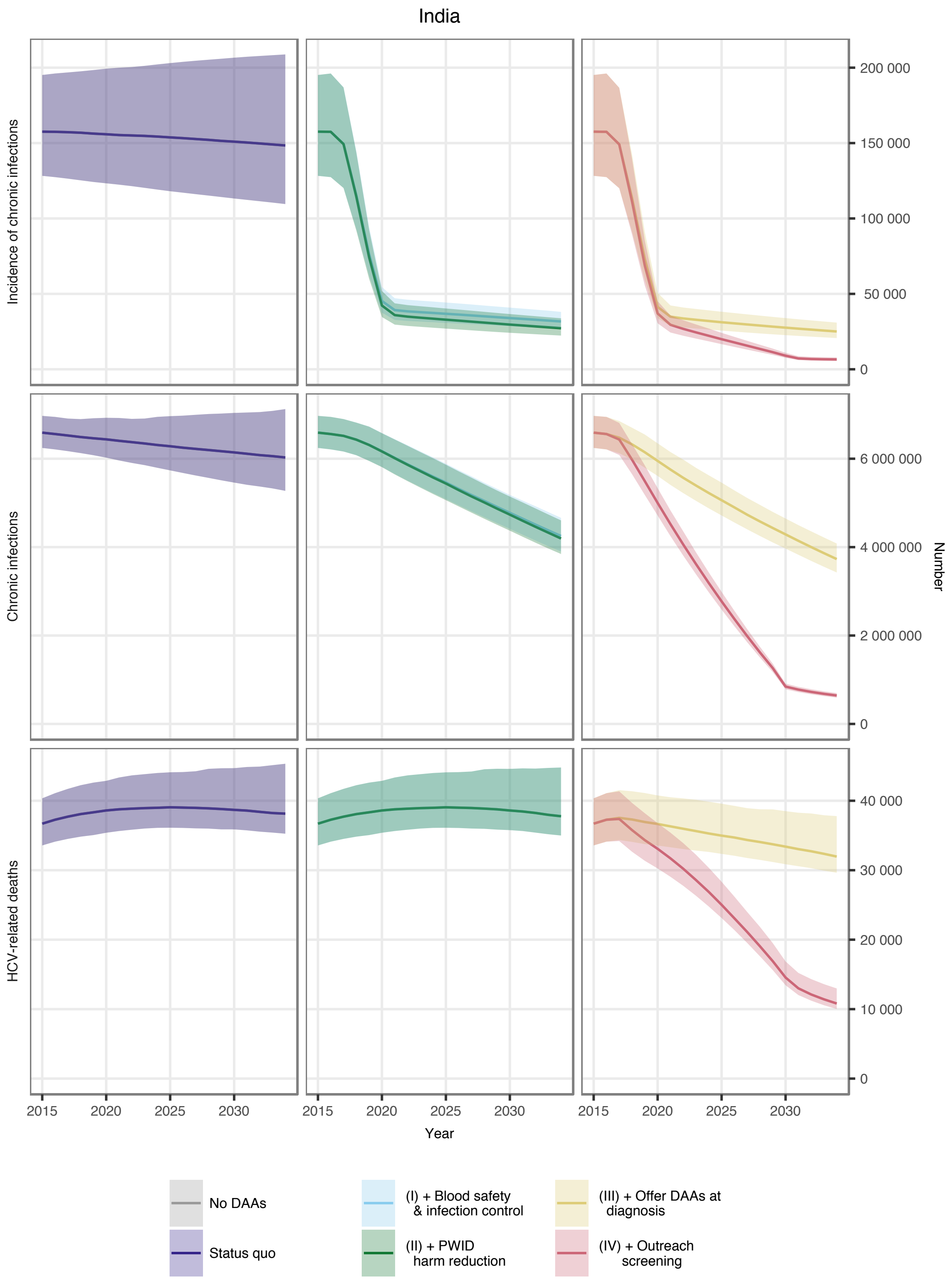

India
Incidence of chronic infections
Chronic infections
HCV-related deaths
Number
200 000
150 000
100 000
50 000
0
6 000 000
4 000 000
2 000 000
0
40 000
30 000
20 000
10 000
0
2015
2020
2025
2030
Year
No DAAs
Status quo
(I) + Blood safety & infection control
(II) + PWID harm reduction
(III) + Offer DAAs at diagnosis
(IV) + Outreach screening

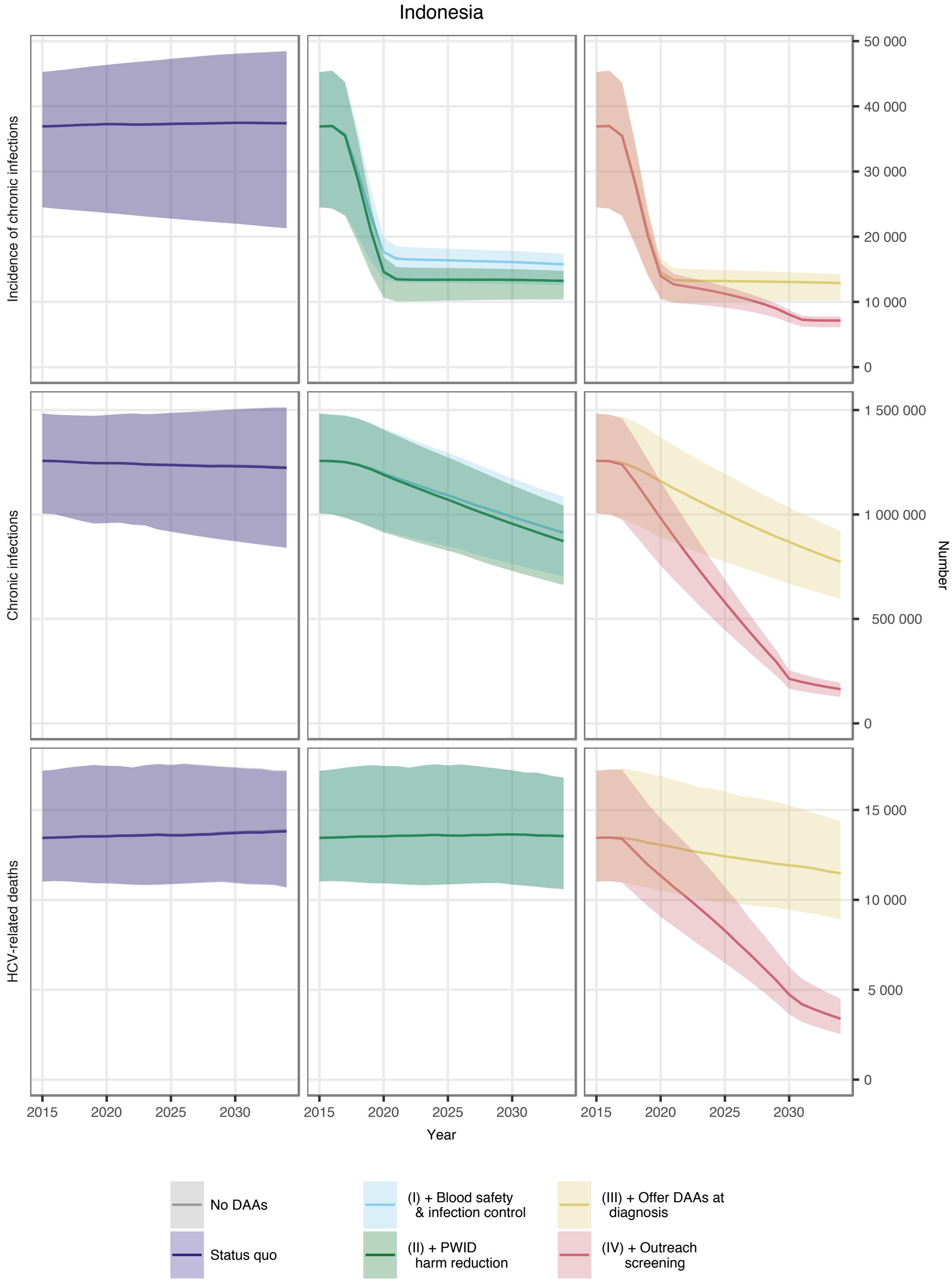
Indonesia
Incidence of chronic infections
Chronic infections
HCV-related deaths
Number
Year
50 000
40 000
30 000
20 000
10 000
0
1 500 000
1 000 000
500 000
0
15 000
10 000
5 000
0
2015
2020
2025
2030
No DAAs
Status quo
(I) + Blood safety & infection control
(II) + PWID harm reduction
(III) + Offer DAAs at diagnosis
(IV) + Outreach screening

Iran, Islamic Republic of

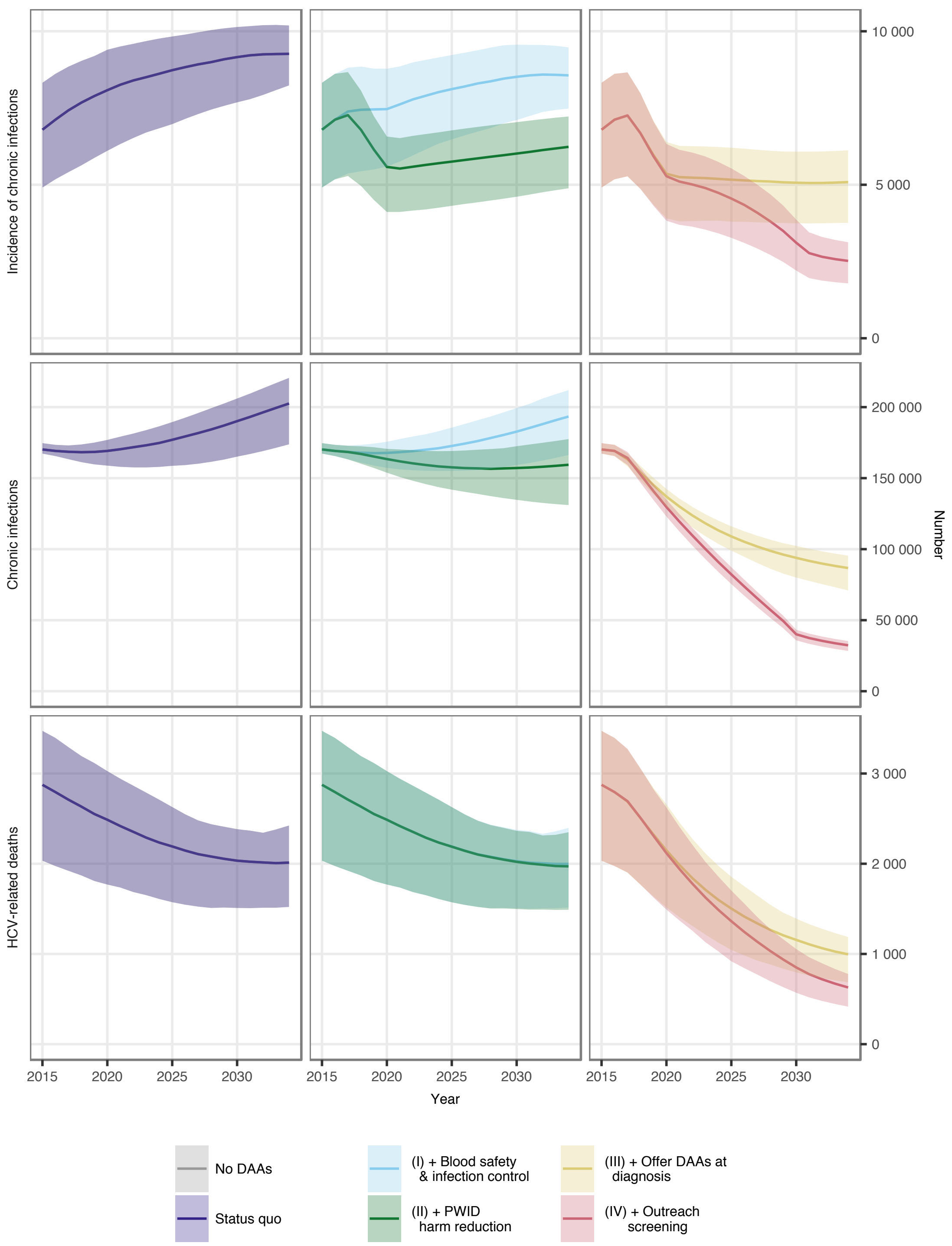

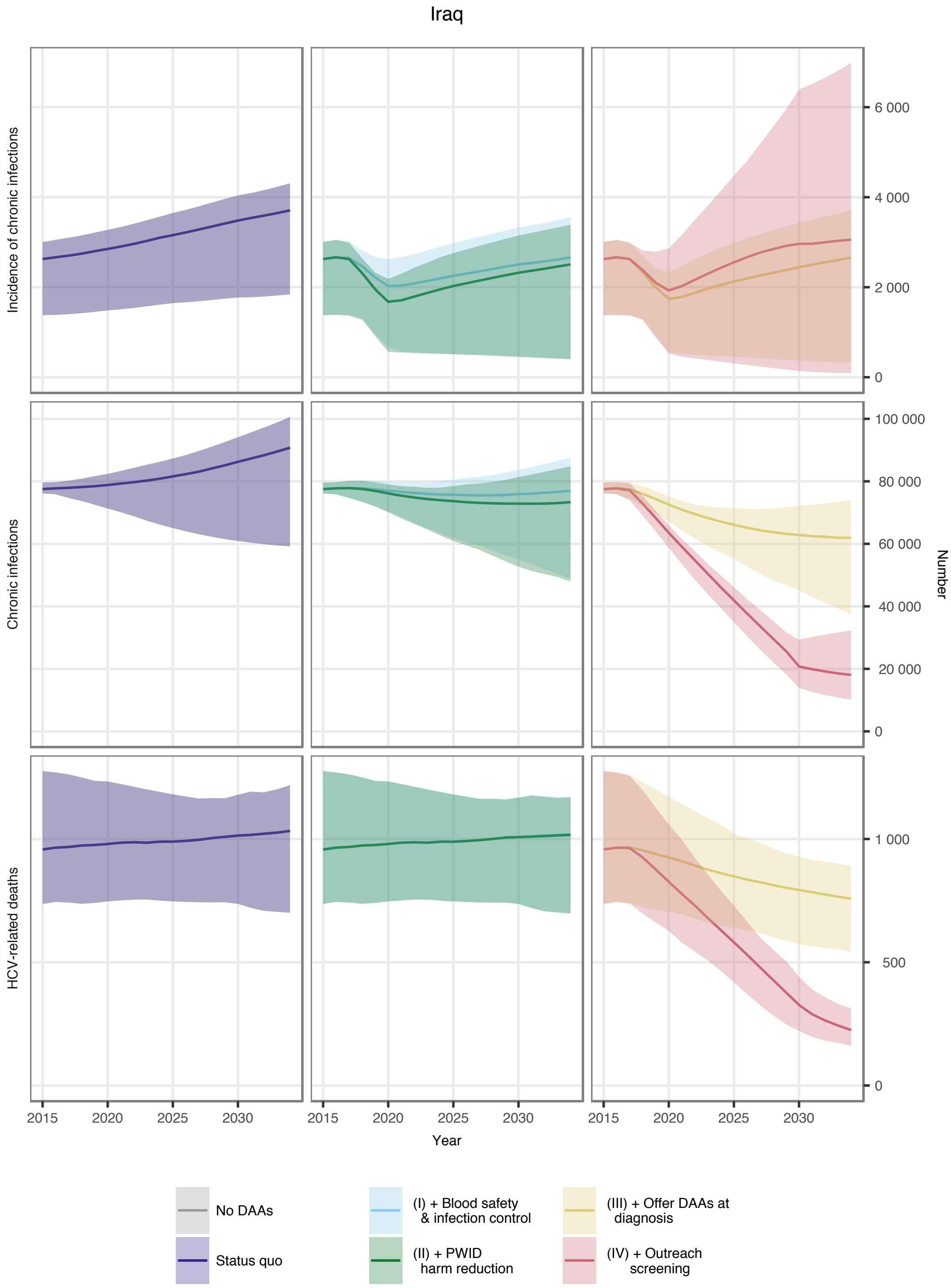
Iraq
Incidence of chronic infections
Chronic infections
HCV-related deaths
Number
6 000
4 000
2 000
0
100 000
80 000
60 000
40 000
20 000
0
1 000
500
0
2015
2020
2025
2030
Year
No DAAs
Status quo
(I) + Blood safety & infection control
(II) + PWID harm reduction
(III) + Offer DAAs at diagnosis
(IV) + Outreach screening

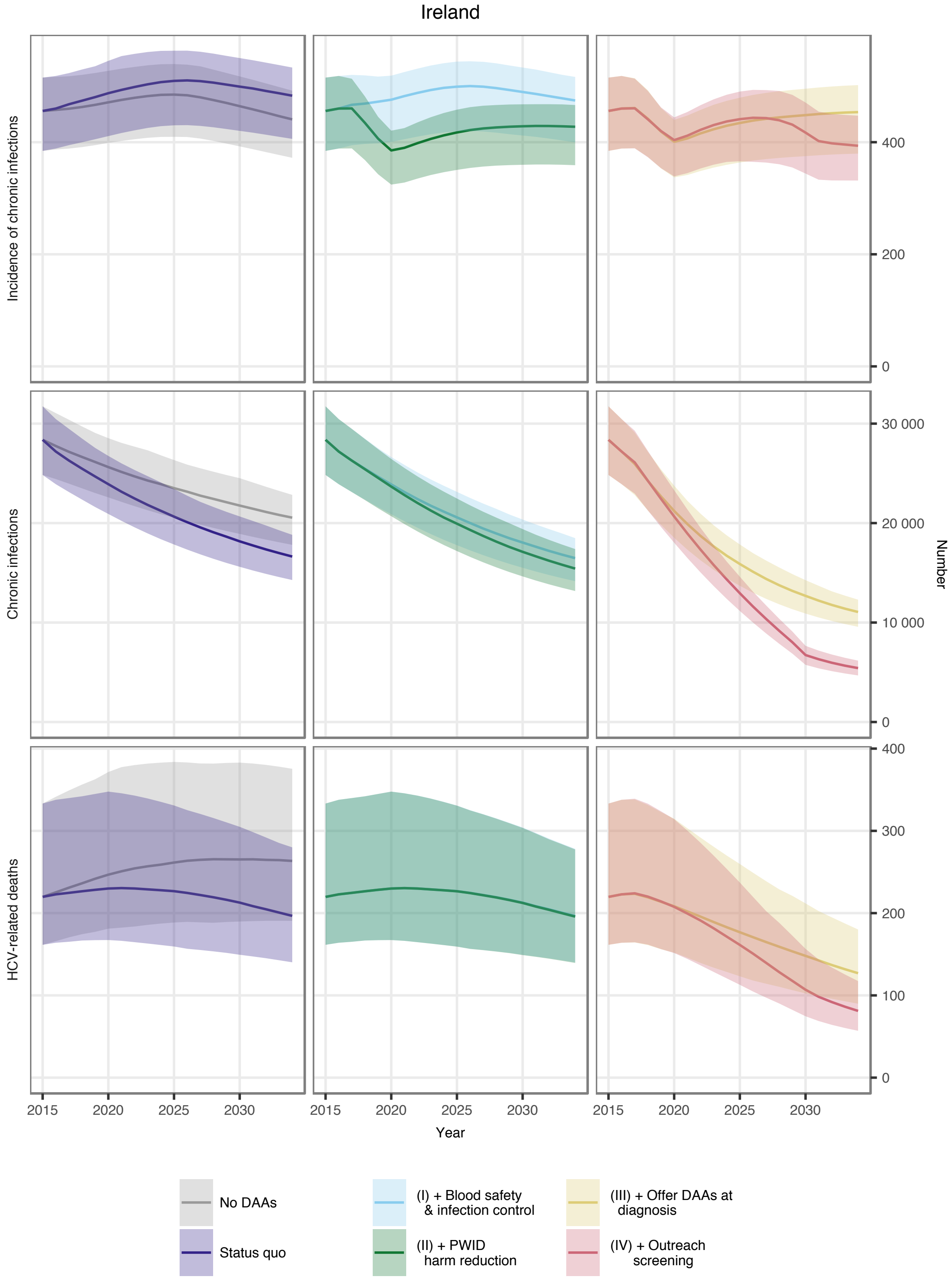
Ireland
Incidence of chronic infections
Chronic infections
HCV-related deaths
Number
0
200
400
10 000
20 000
30 000
100
300
2015
2020
2025
2030
Year
No DAAs
Status quo
(I) + Blood safety & infection control
(II) + PWID harm reduction
(III) + Offer DAAs at diagnosis
(IV) + Outreach screening

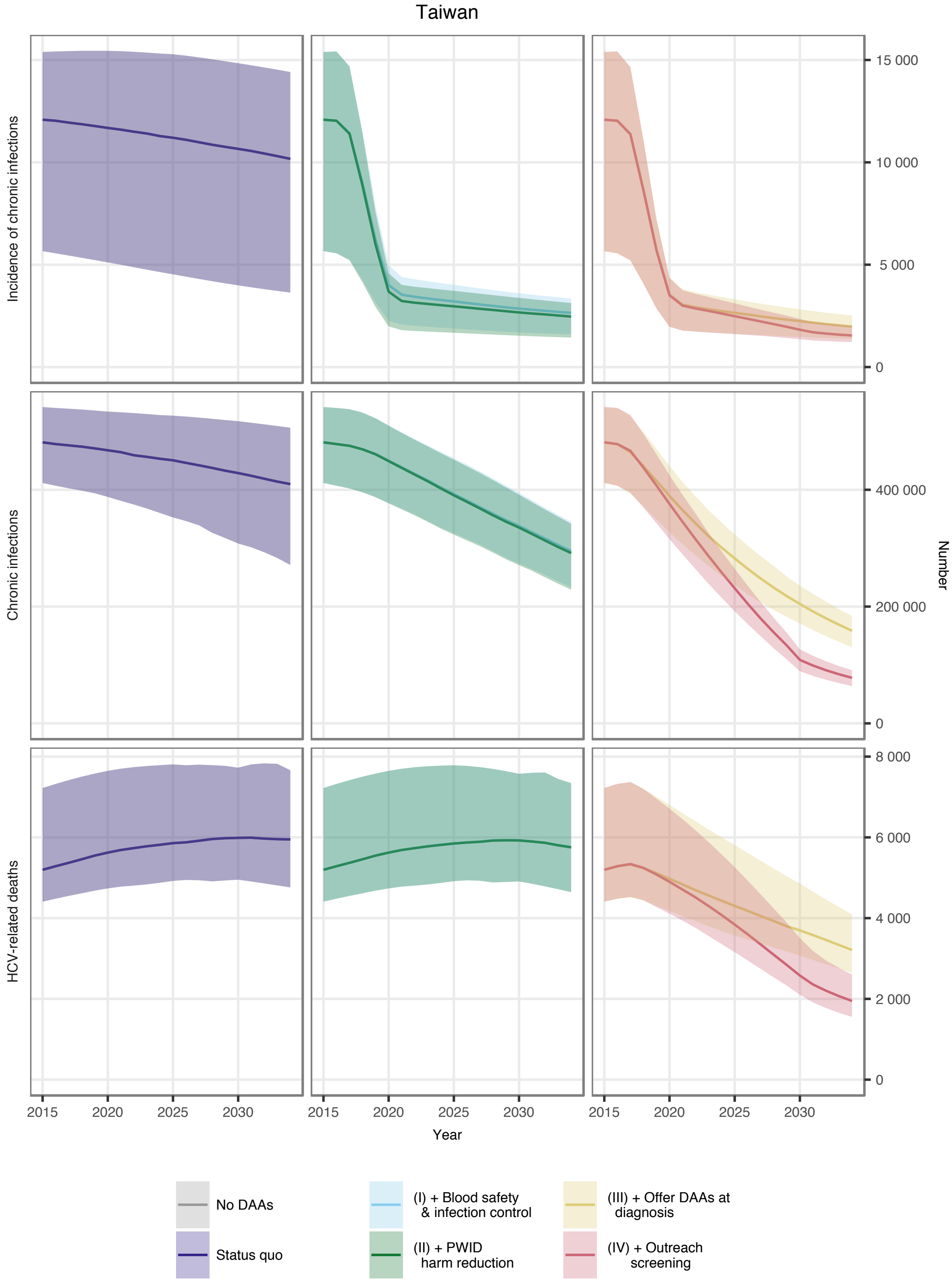
Taiwan
Incidence of chronic infections
Chronic infections
HCV-related deaths
Number
15 000
10 000
5 000
0
400 000
200 000
0
8 000
6 000
4 000
2 000
0
2015
2020
2025
2030
Year
No DAAs
Status quo
(I) + Blood safety & infection control
(II) + PWID harm reduction
(III) + Offer DAAs at diagnosis
(IV) + Outreach screening

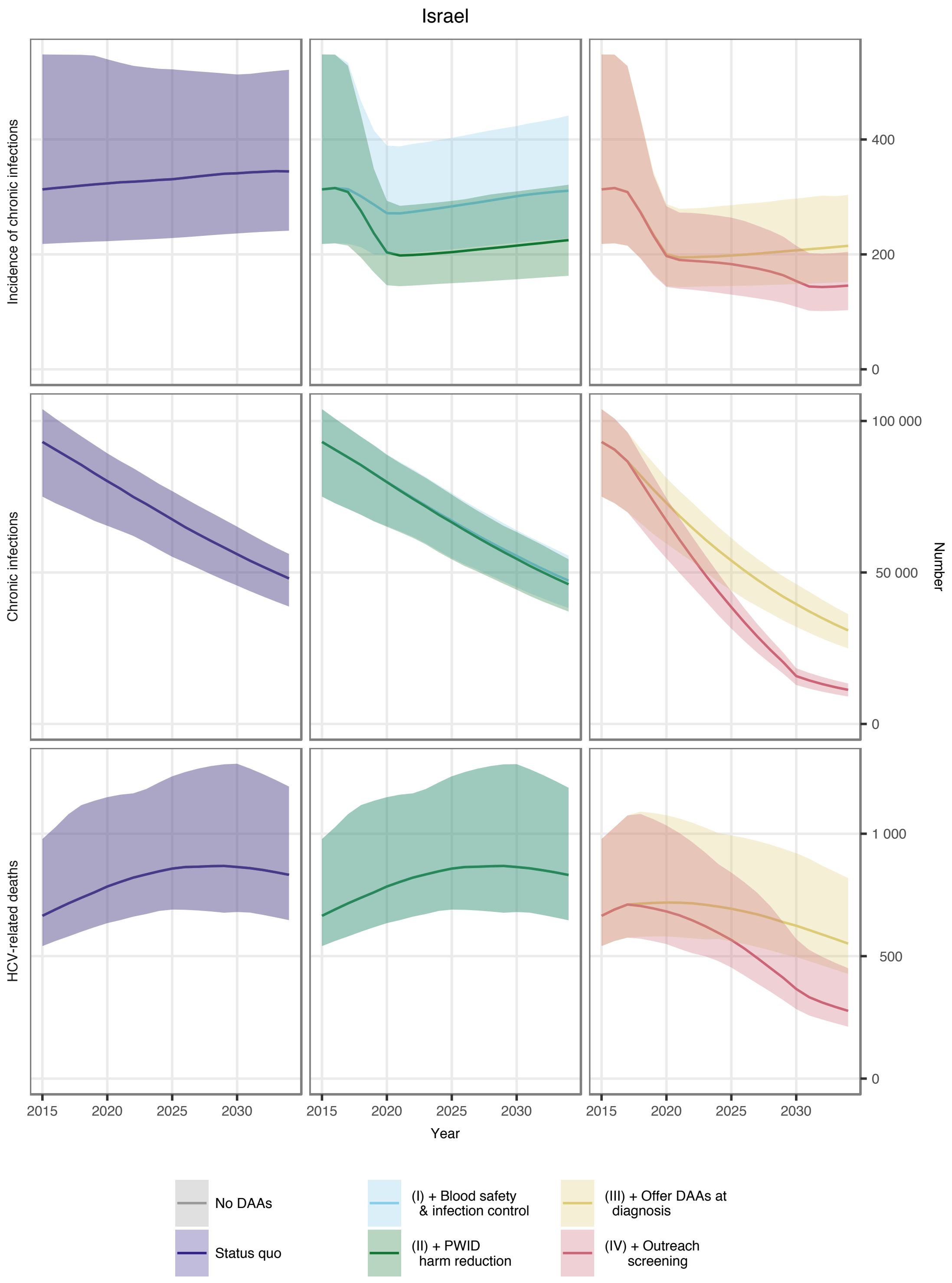
Israel
Incidence of chronic infections
Chronic infections
HCV-related deaths
Number
400
200
0
100 000
50 000
0
1 000
500
0
2015
2020
2025
2030
Year
No DAAs
Status quo
(I) + Blood safety & infection control
(II) + PWID harm reduction
(III) + Offer DAAs at diagnosis
(IV) + Outreach screening

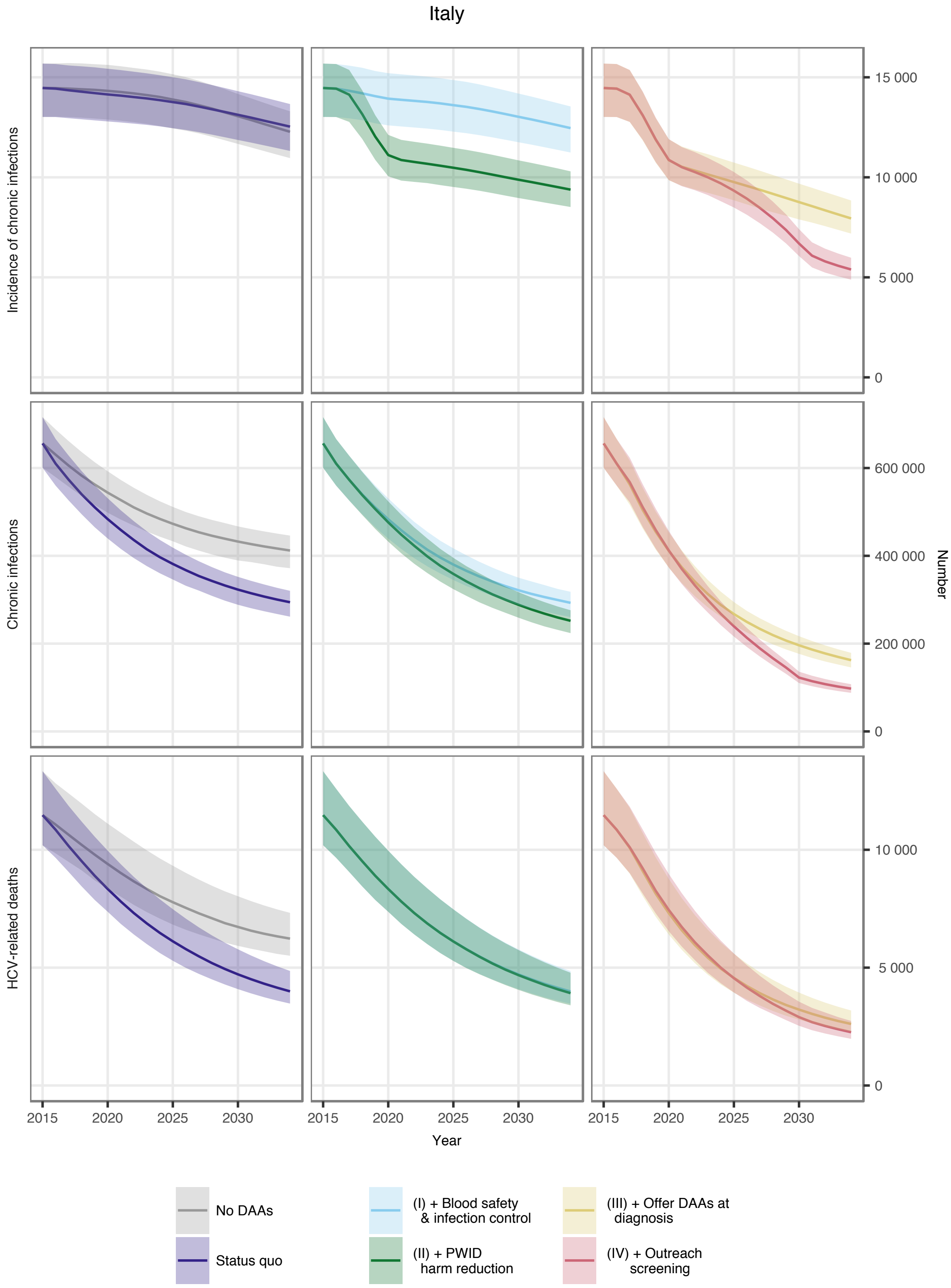

Italy
Incidence of chronic infections
Chronic infections
HCV-related deaths
Number
Year
15 000
10 000
5 000
0
600 000
400 000
200 000
0
10 000
5 000
0
2015
2020
2025
2030
No DAAs
Status quo
(I) + Blood safety & infection control
(II) + PWID harm reduction
(III) + Offer DAAs at diagnosis
(IV) + Outreach screening

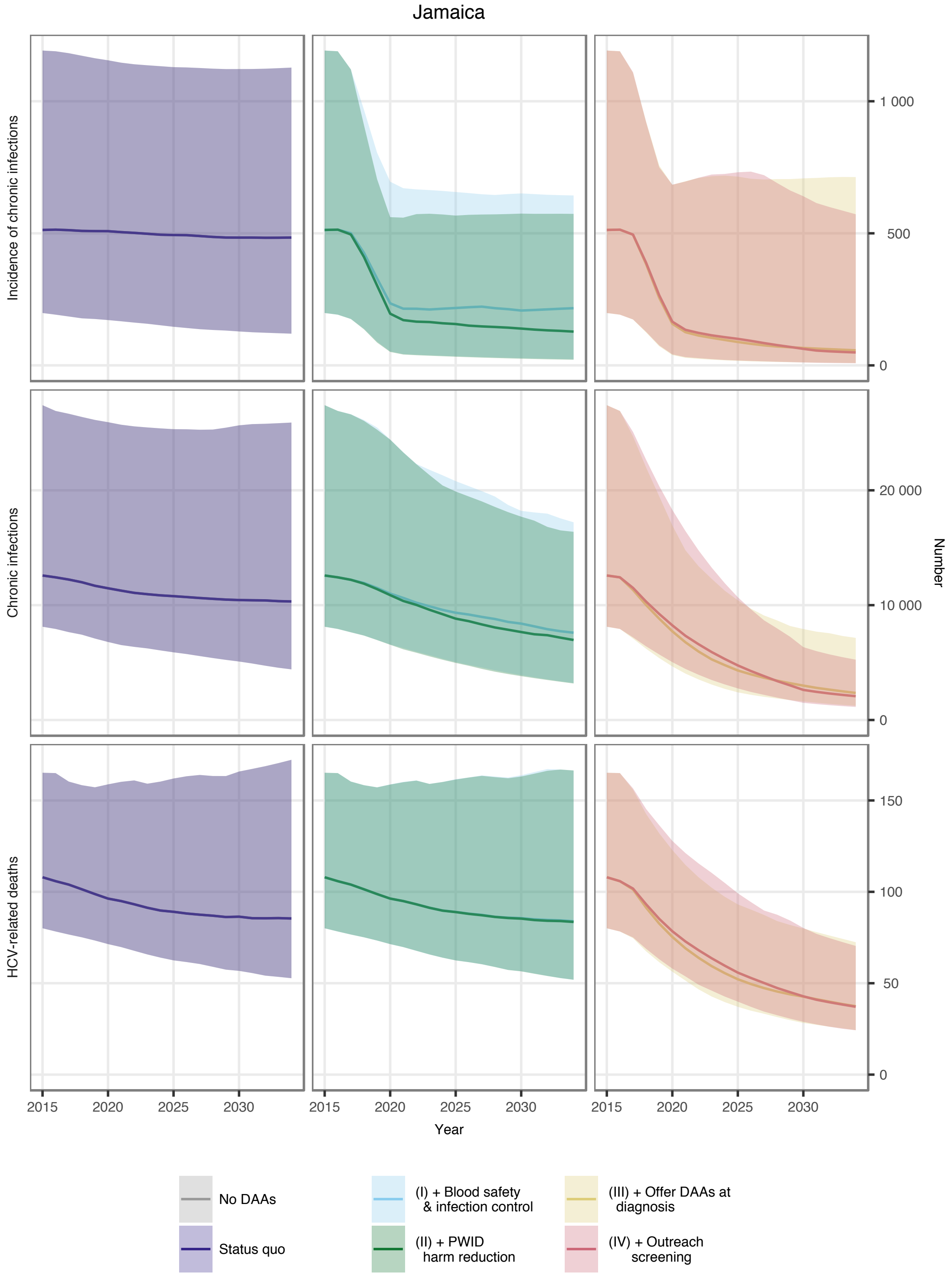

Jamaica
Incidence of chronic infections
Chronic infections
HCV-related deaths
Number
Year
2015
2020
2025
2030
1 000
500
0
20 000
10 000
0
150
100
50
0
No DAAs
Status quo
(I) + Blood safety & infection control
(II) + PWID harm reduction
(III) + Offer DAAs at diagnosis
(IV) + Outreach screening

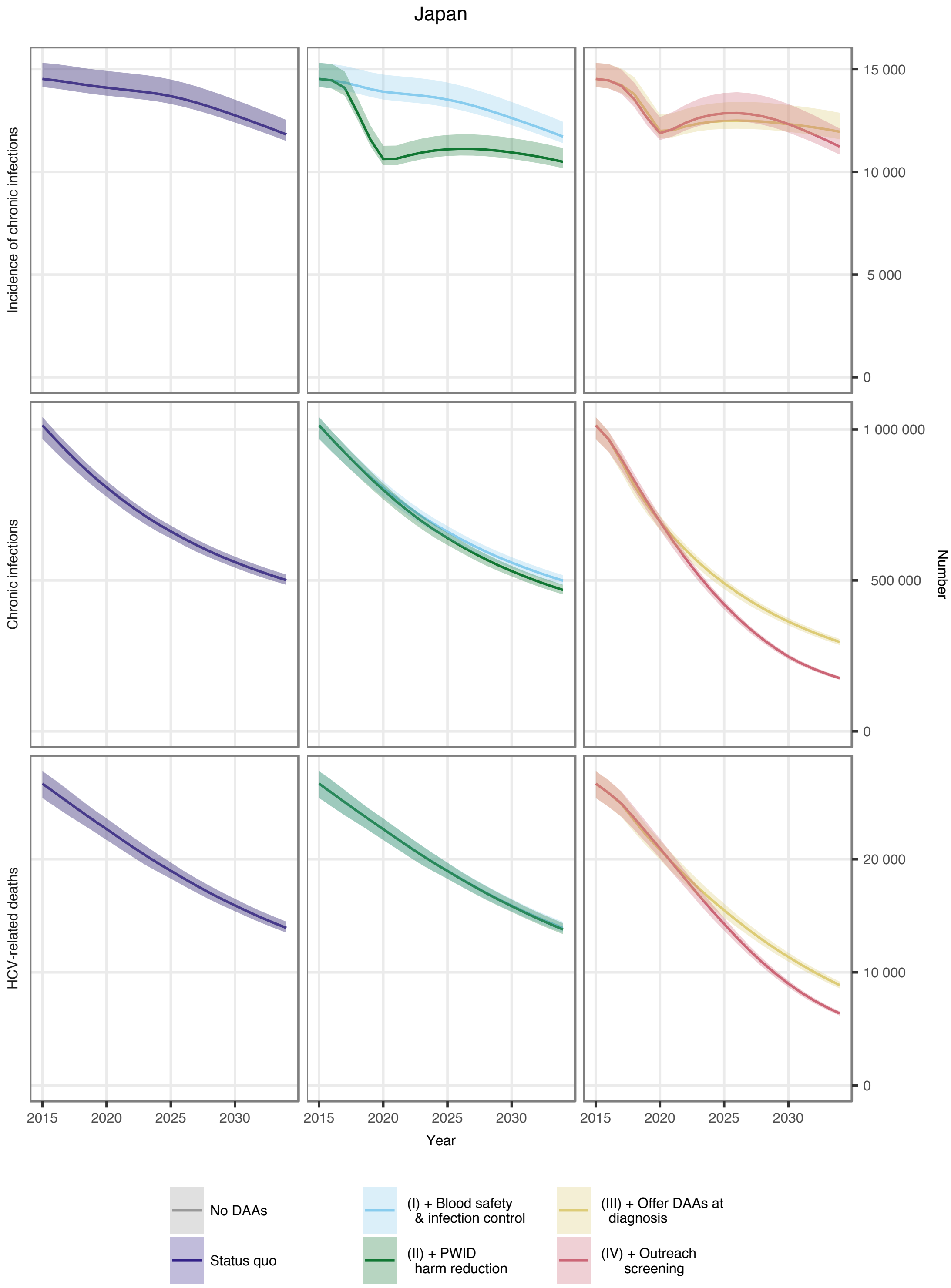
Japan
Incidence of chronic infections
Chronic infections
HCV-related deaths
Number
Year
15 000
10 000
5 000
0
1 000 000
500 000
0
20 000
10 000
0
2015
2020
2025
2030
No DAAs
Status quo
(I) + Blood safety & infection control
(II) + PWID harm reduction
(III) + Offer DAAs at diagnosis
(IV) + Outreach screening

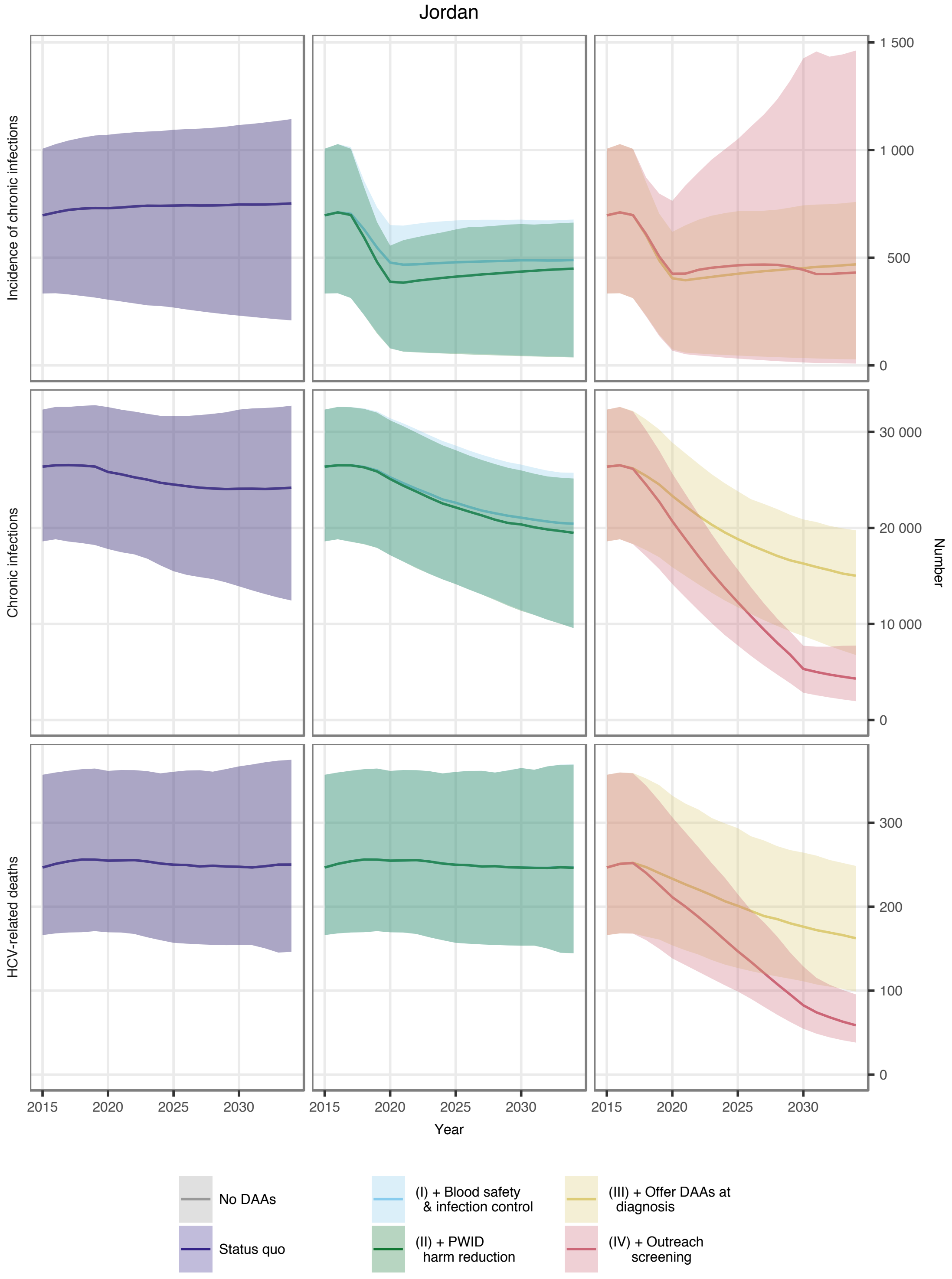

Jordan
Incidence of chronic infections
Chronic infections
HCV-related deaths
Number
1 500
1 000
500
0
30 000
20 000
10 000
0
300
200
100
0
2015
2020
2025
2030
Year
No DAAs
Status quo
(I) + Blood safety & infection control
(II) + PWID harm reduction
(III) + Offer DAAs at diagnosis
(IV) + Outreach screening

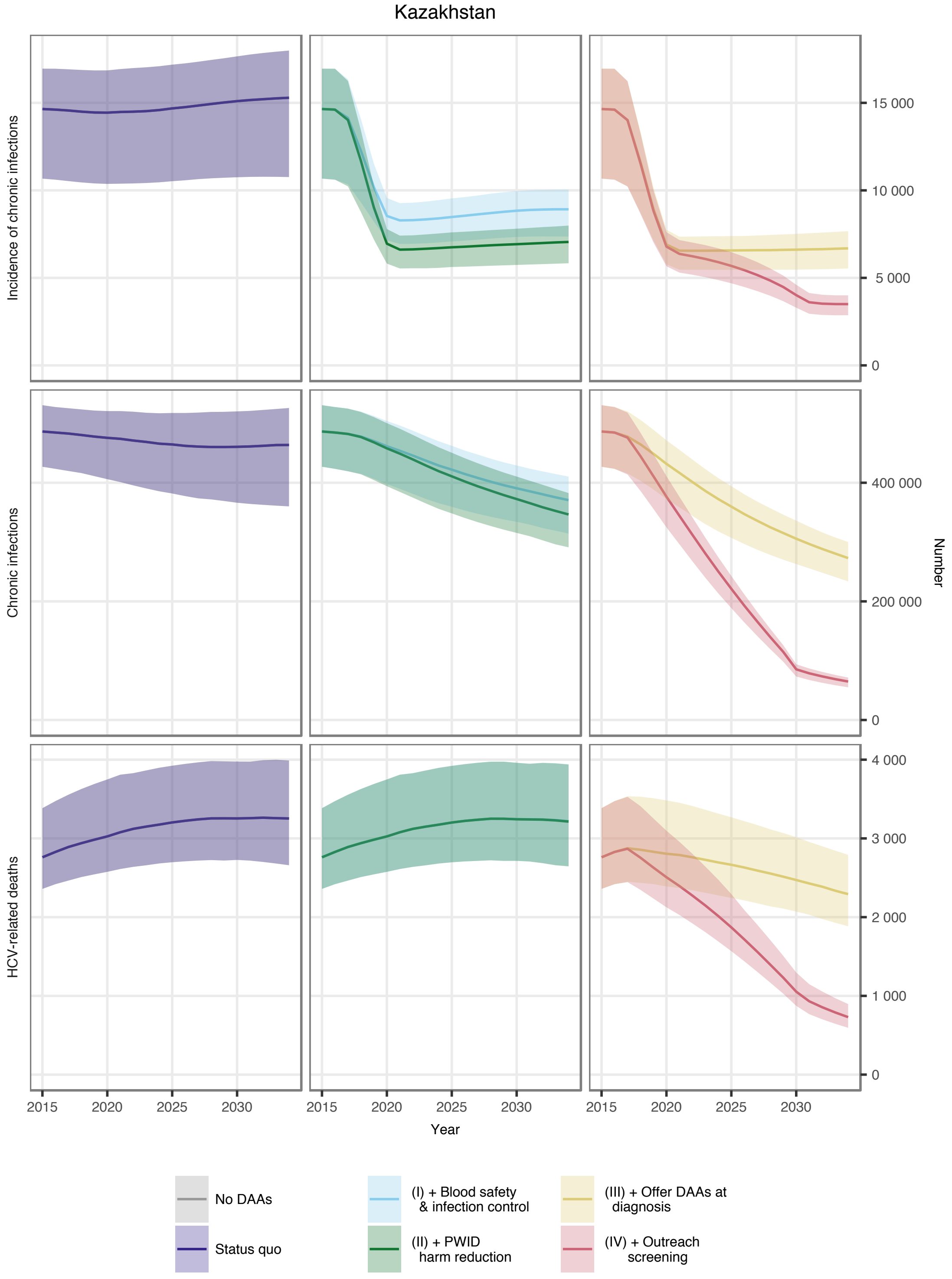
Kazakhstan
Incidence of chronic infections
Chronic infections
HCV-related deaths
Number
15 000
10 000
5 000
0
400 000
200 000
0
4 000
3 000
2 000
1 000
0
2015
2020
2025
2030
Year
No DAAs
Status quo
(I) + Blood safety & infection control
(II) + PWID harm reduction
(III) + Offer DAAs at diagnosis
(IV) + Outreach screening

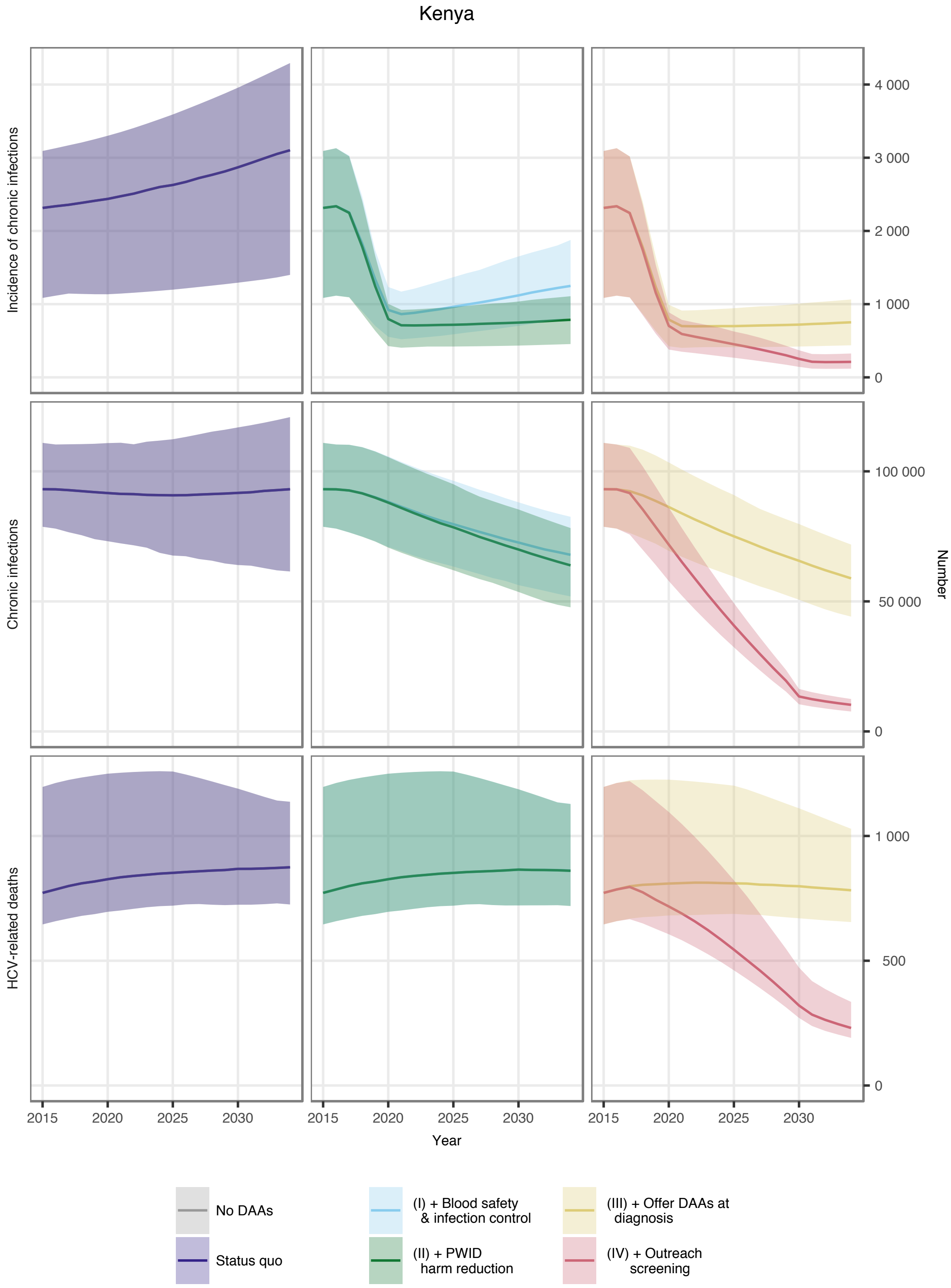
Kenya
Incidence of chronic infections
Chronic infections
HCV-related deaths
4 000
3 000
2 000
1 000
0
100 000
50 000
0
1 000
500
0
Number
2015
2020
2025
2030
Year
No DAAs
Status quo
(I) + Blood safety & infection control
(II) + PWID harm reduction
(III) + Offer DAAs at diagnosis
(IV) + Outreach screening

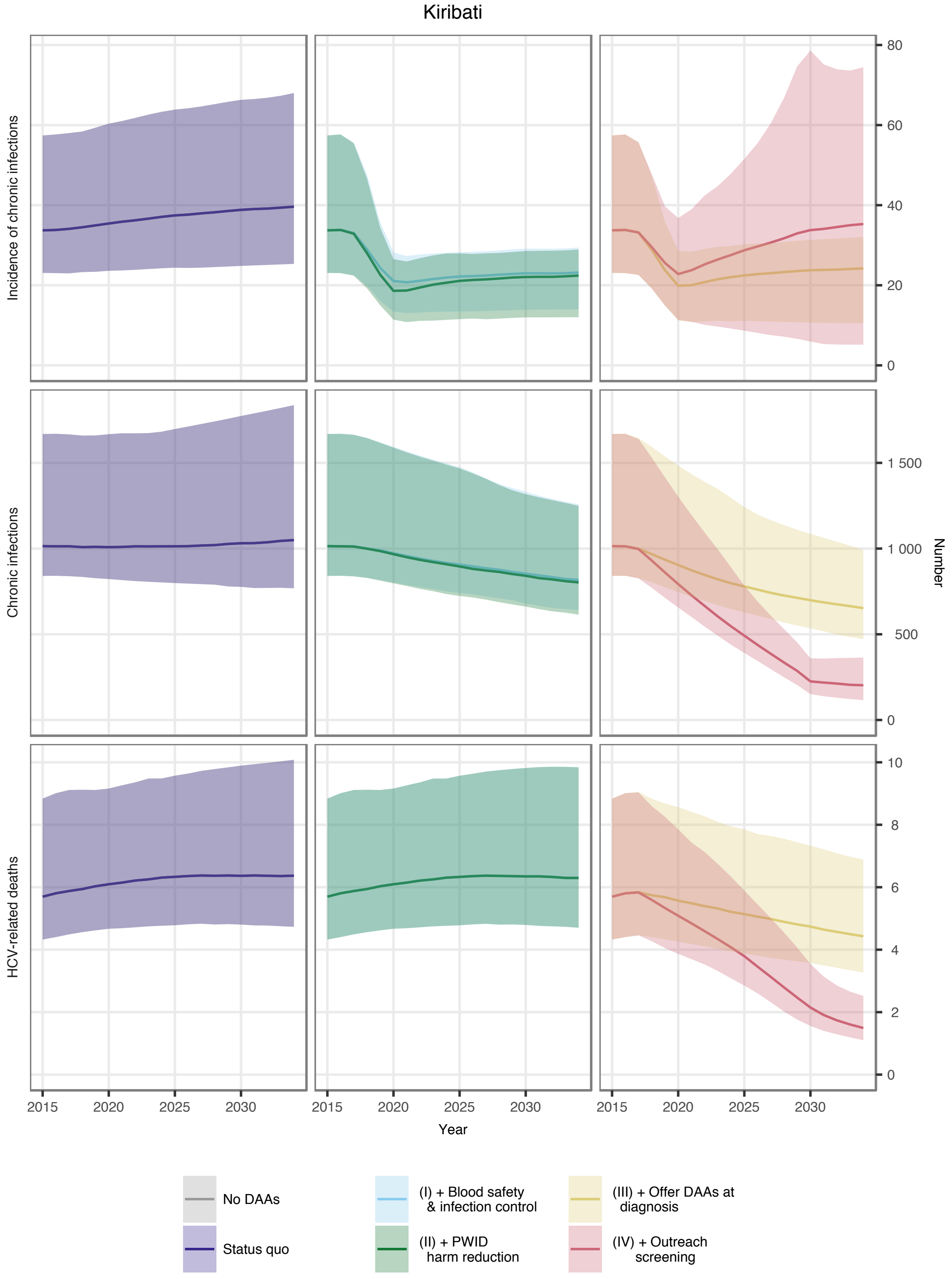

Kiribati
Incidence of chronic infections
Chronic infections
HCV-related deaths
Number
Year
0
20
40
60
80
500
1 000
1 500
2
4
6
8
10
2015
2020
2025
2030
No DAAs
Status quo
(I) + Blood safety & infection control
(II) + PWID harm reduction
(III) + Offer DAAs at diagnosis
(IV) + Outreach screening

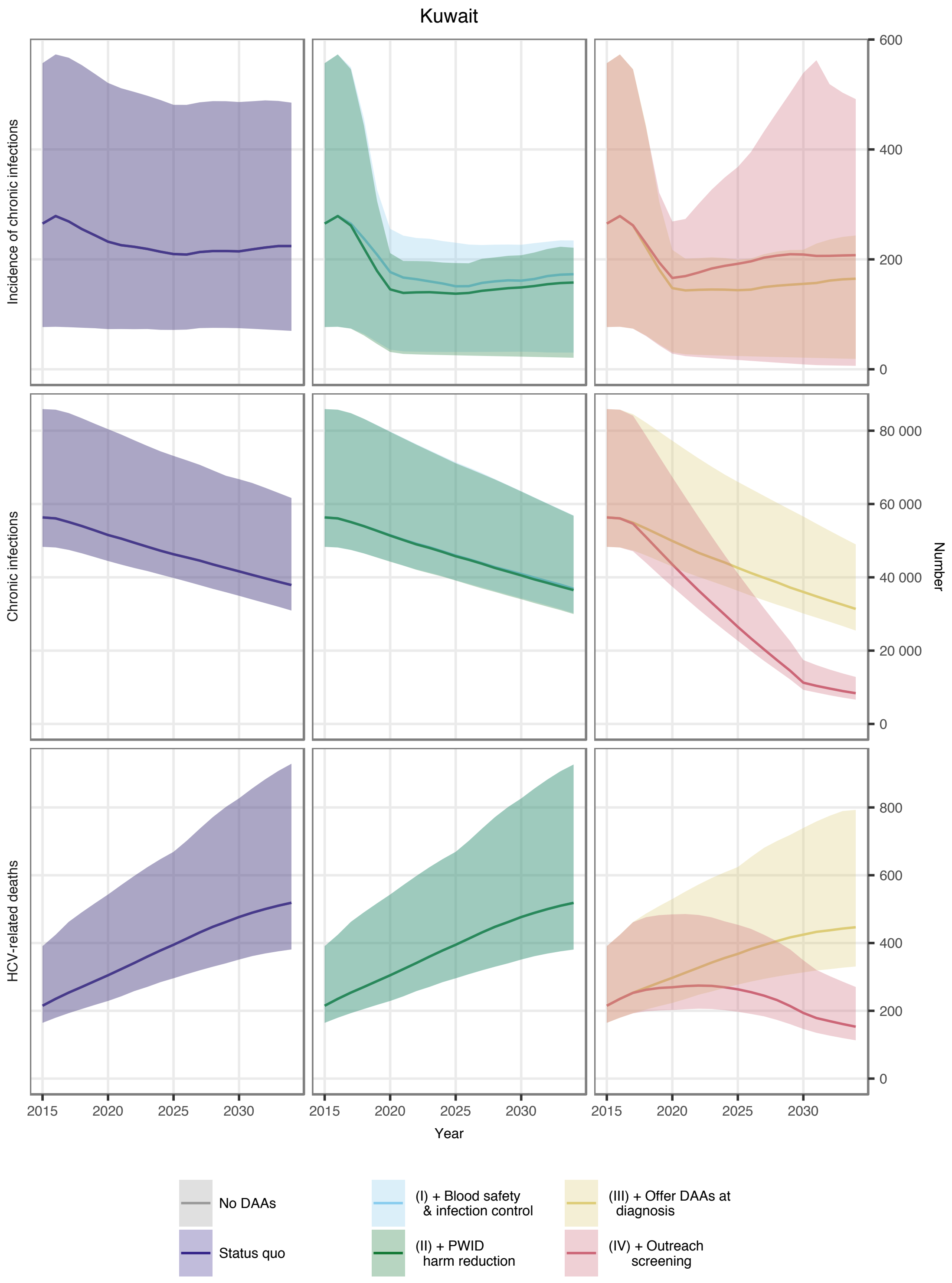
Kuwait
Incidence of chronic infections
Chronic infections
HCV-related deaths
Number
Year
No DAAs
Status quo
(I) + Blood safety & infection control
(II) + PWID harm reduction
(III) + Offer DAAs at diagnosis
(IV) + Outreach screening

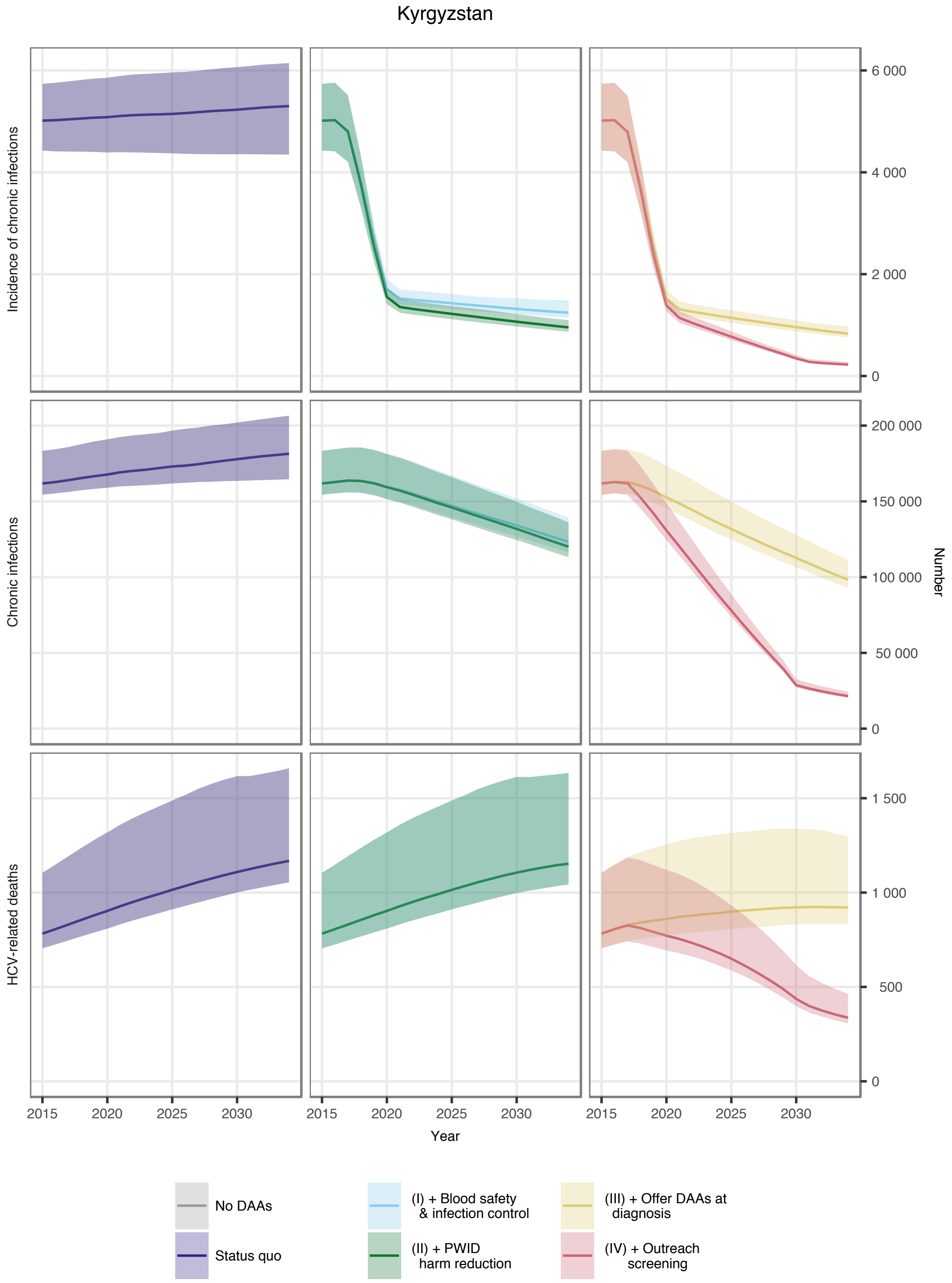
Kyrgyzstan
Incidence of chronic infections
Chronic infections
HCV-related deaths
Number
Year
6 000
4 000
2 000
0
200 000
150 000
100 000
50 000
0
1 500
1 000
500
0
2015
2020
2025
2030
No DAAs
Status quo
(I) + Blood safety & infection control
(II) + PWID harm reduction
(III) + Offer DAAs at diagnosis
(IV) + Outreach screening

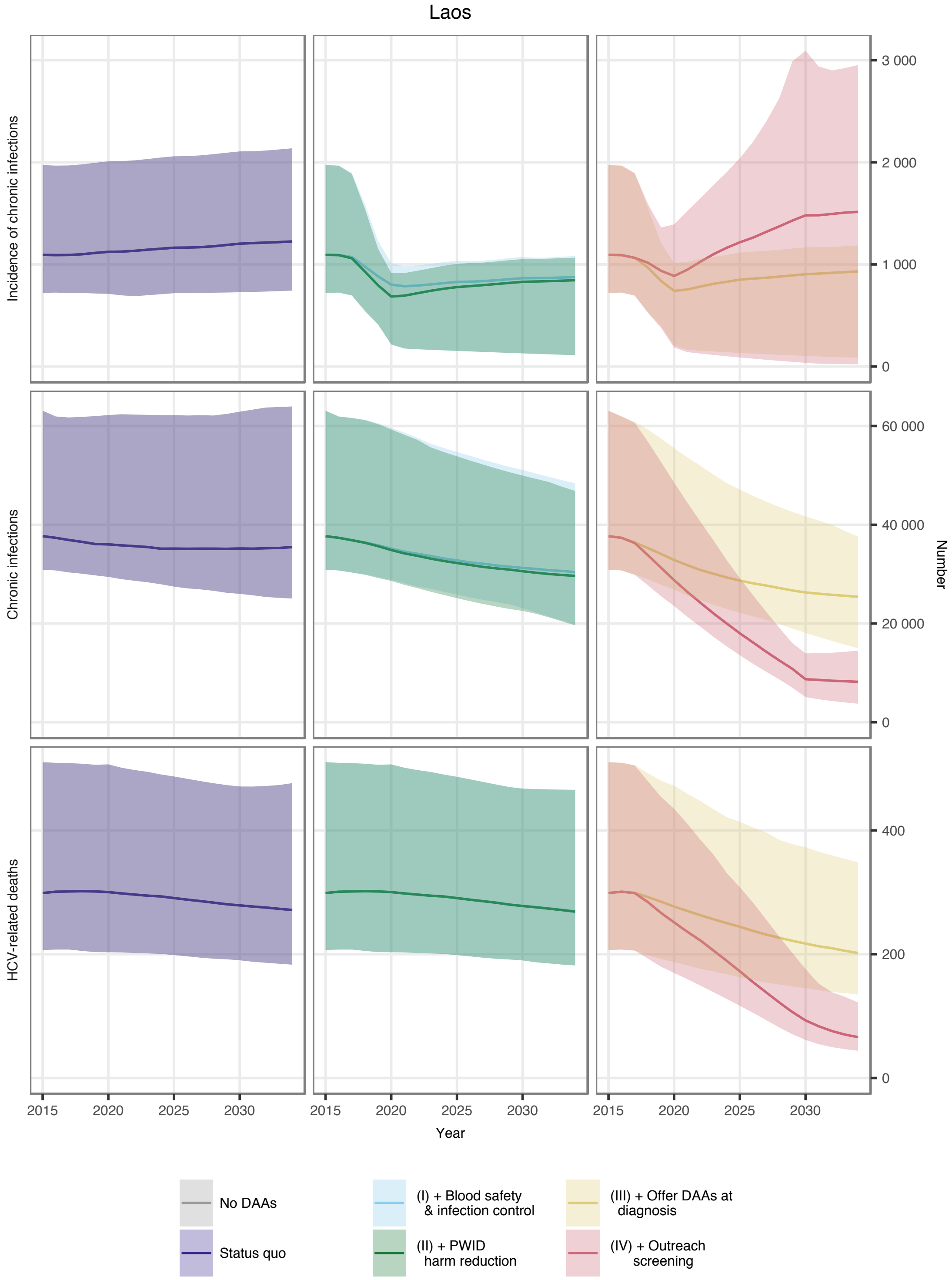
Laos
Incidence of chronic infections
Chronic infections
HCV-related deaths
Number
0
1 000
2 000
3 000
0
20 000
40 000
60 000
0
200
400
2015
2020
2025
2030
Year
No DAAs
Status quo
(I) + Blood safety & infection control
(II) + PWID harm reduction
(III) + Offer DAAs at diagnosis
(IV) + Outreach screening

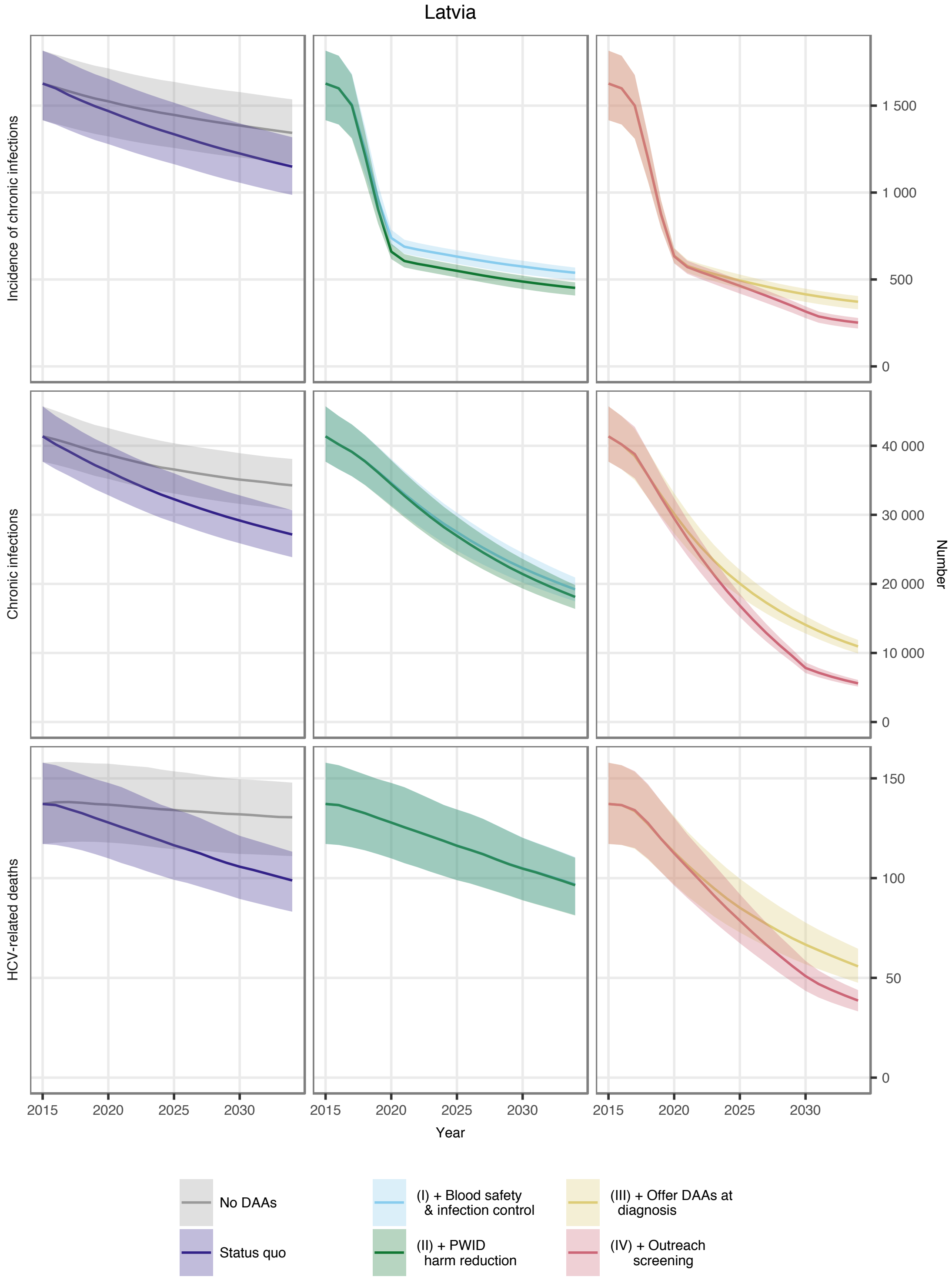
Latvia
Incidence of chronic infections
Chronic infections
HCV-related deaths
1 500
1 000
500
0
40 000
30 000
20 000
10 000
0
150
100
50
0
Number
2015
2020
2025
2030
Year
No DAAs
Status quo
(I) + Blood safety & infection control
(II) + PWID harm reduction
(III) + Offer DAAs at diagnosis
(IV) + Outreach screening

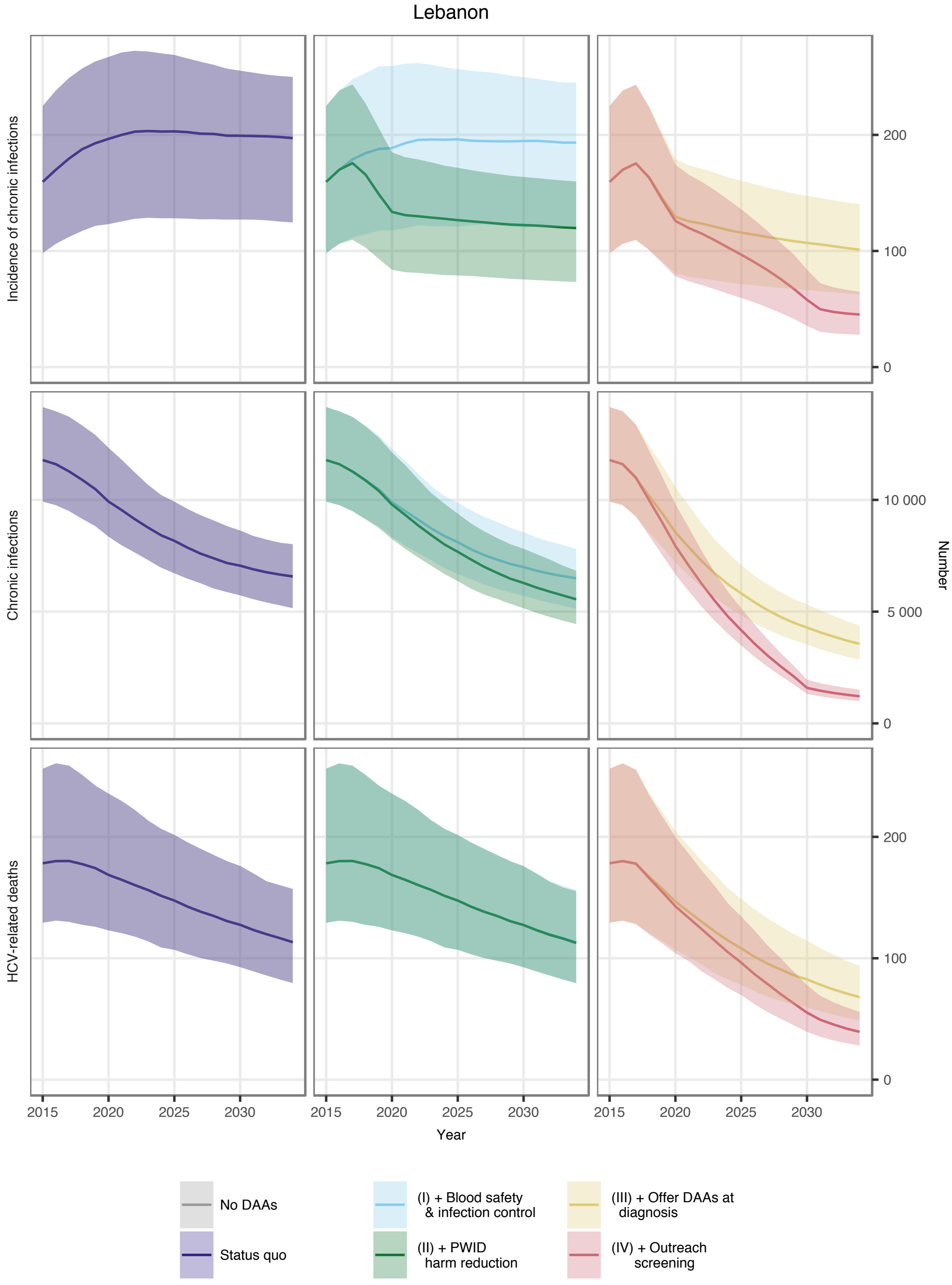
Lebanon
Incidence of chronic infections
Chronic infections
HCV-related deaths
Number
Year
0
100
200
0
5 000
10 000
2015
2020
2025
2030
No DAAs
Status quo
(I) + Blood safety & infection control
(II) + PWID harm reduction
(III) + Offer DAAs at diagnosis
(IV) + Outreach screening

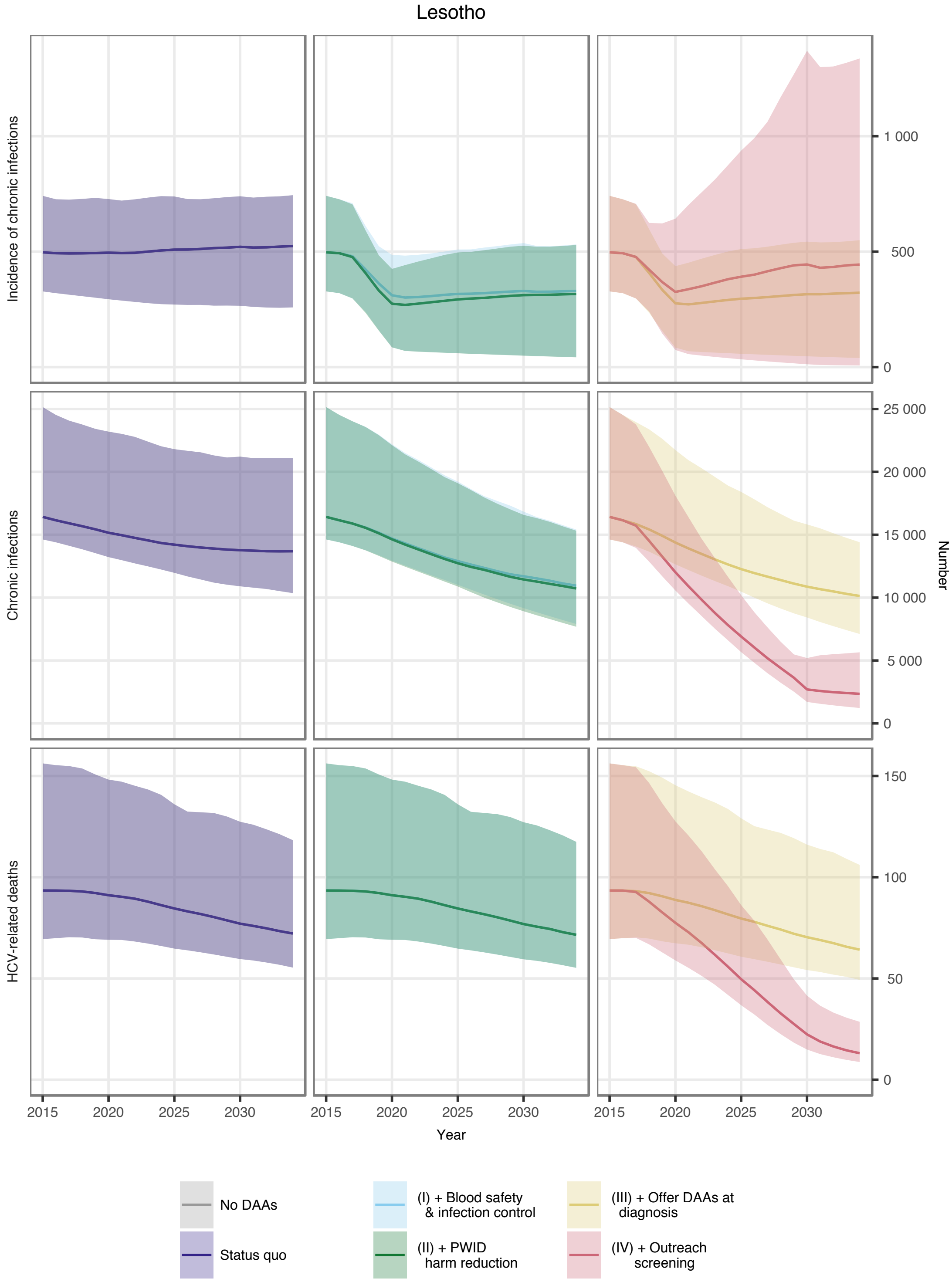

Lesotho
Incidence of chronic infections
Chronic infections
HCV-related deaths
Number
1 000
500
0
25 000
20 000
15 000
10 000
5 000
0
150
100
50
0
2015
2020
2025
2030
Year
No DAAs
Status quo
(I) + Blood safety & infection control
(II) + PWID harm reduction
(III) + Offer DAAs at diagnosis
(IV) + Outreach screening

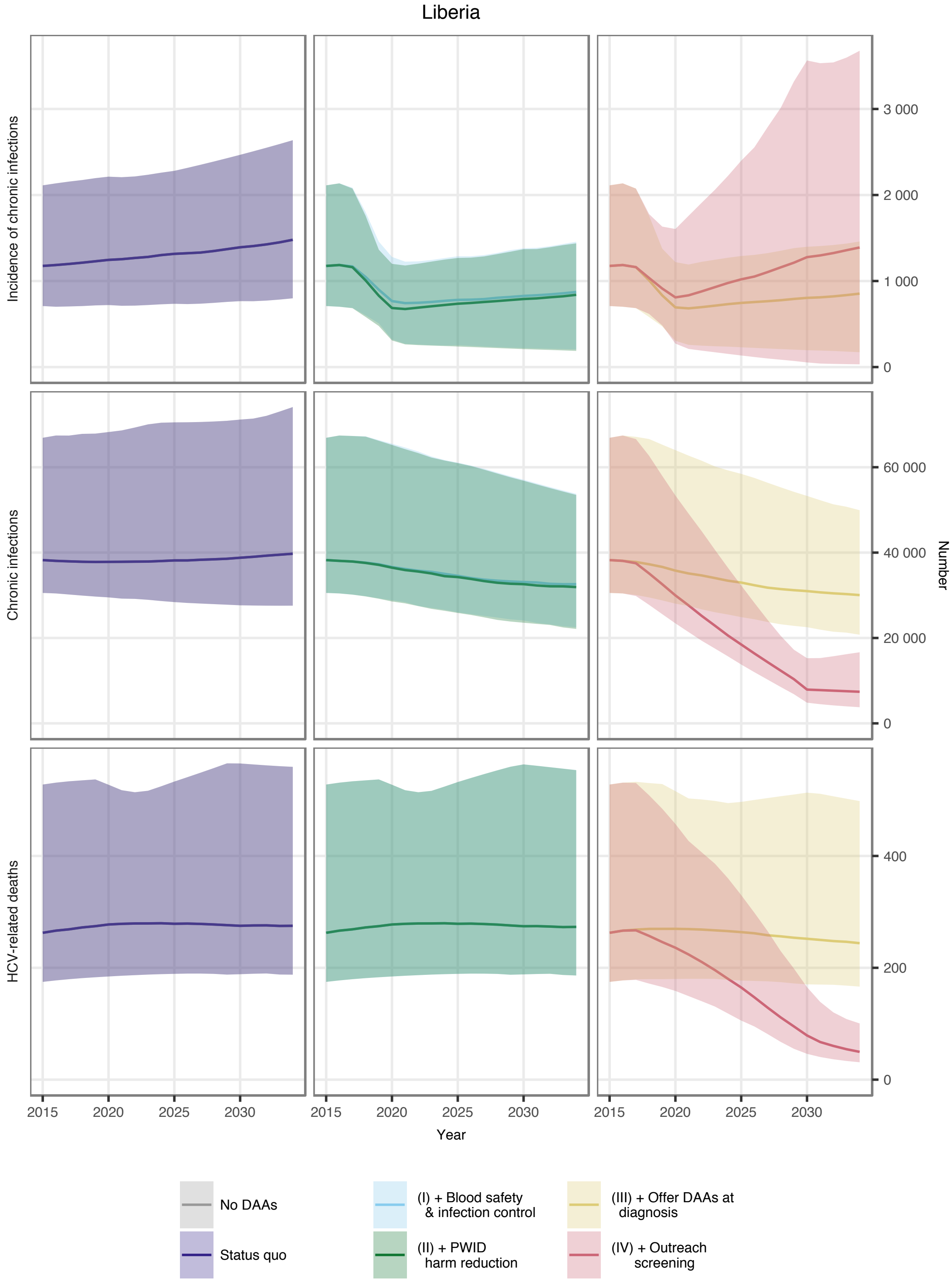

Liberia
Incidence of chronic infections
Chronic infections
HCV-related deaths
Number
3 000
2 000
1 000
0
60 000
40 000
20 000
0
400
200
0
2015
2020
2025
2030
Year
No DAAs
Status quo
(I) + Blood safety & infection control
(II) + PWID harm reduction
(III) + Offer DAAs at diagnosis
(IV) + Outreach screening

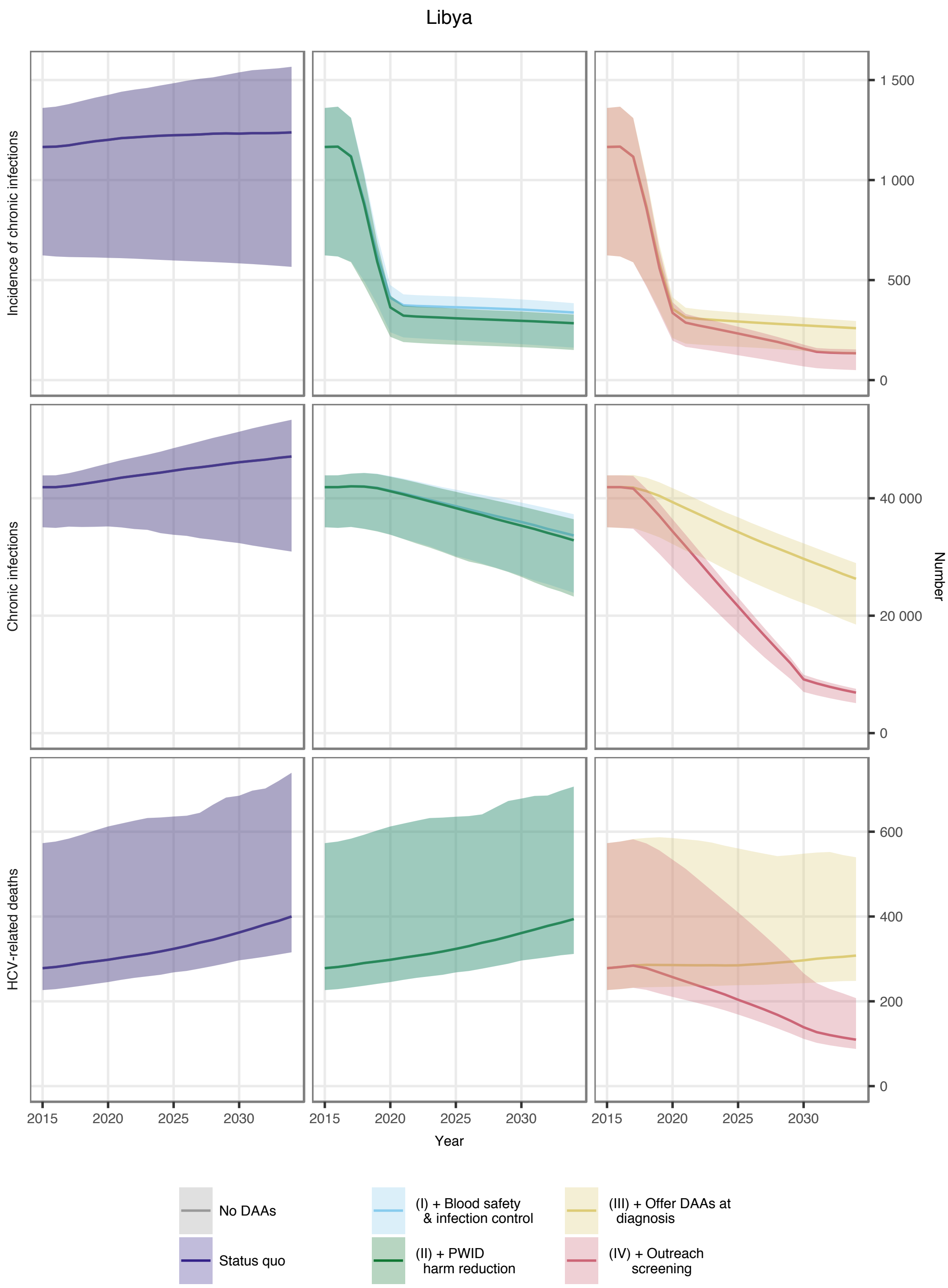

Libya
Incidence of chronic infections
Chronic infections
HCV-related deaths
Number
Year
1 500
1 000
500
0
40 000
20 000
0
600
400
200
0
2015
2020
2025
2030
No DAAs
Status quo
(I) + Blood safety & infection control
(II) + PWID harm reduction
(III) + Offer DAAs at diagnosis
(IV) + Outreach screening

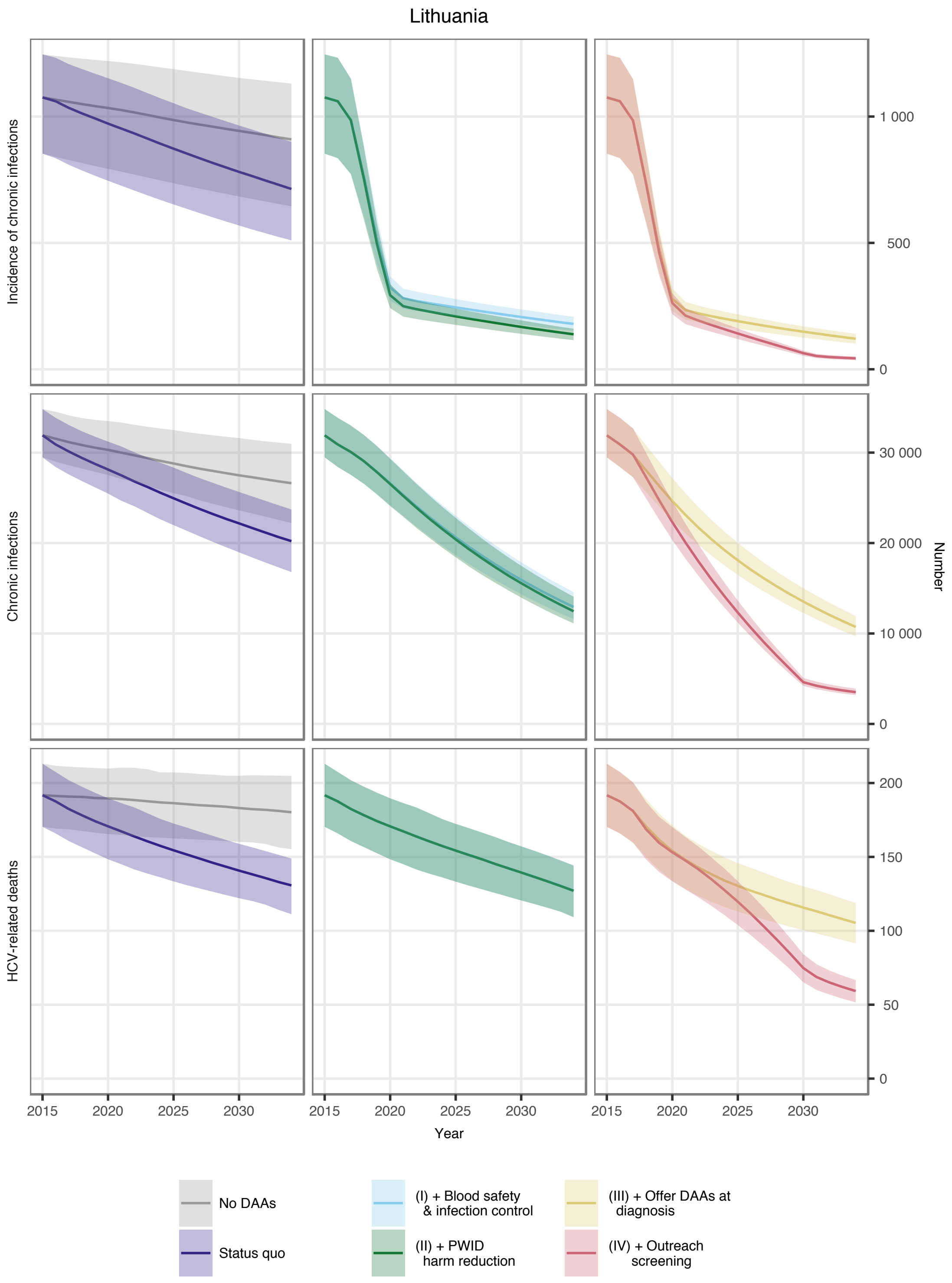

Lithuania
Incidence of chronic infections
Chronic infections
HCV-related deaths
Number
Year
0
500
1 000
10 000
20 000
30 000
50
100
150
200
2015
2020
2025
2030
No DAAs
Status quo
(I) + Blood safety & infection control
(II) + PWID harm reduction
(III) + Offer DAAs at diagnosis
(IV) + Outreach screening

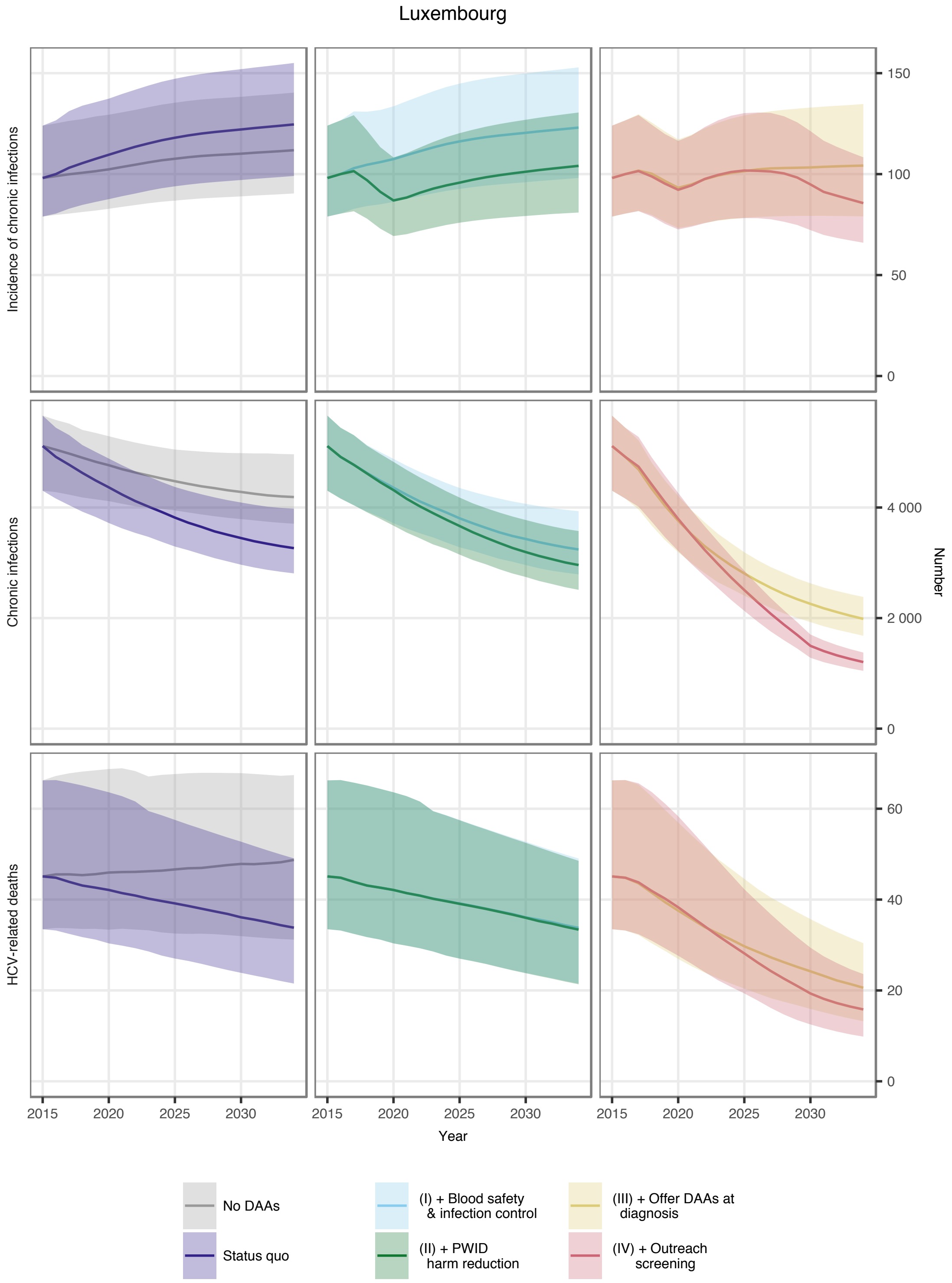

Luxembourg
Incidence of chronic infections
Chronic infections
HCV-related deaths
Number
Year
150
100
50
0
4 000
2 000
0
60
40
20
0
2015
2020
2025
2030
No DAAs
Status quo
(I) + Blood safety & infection control
(II) + PWID harm reduction
(III) + Offer DAAs at diagnosis
(IV) + Outreach screening

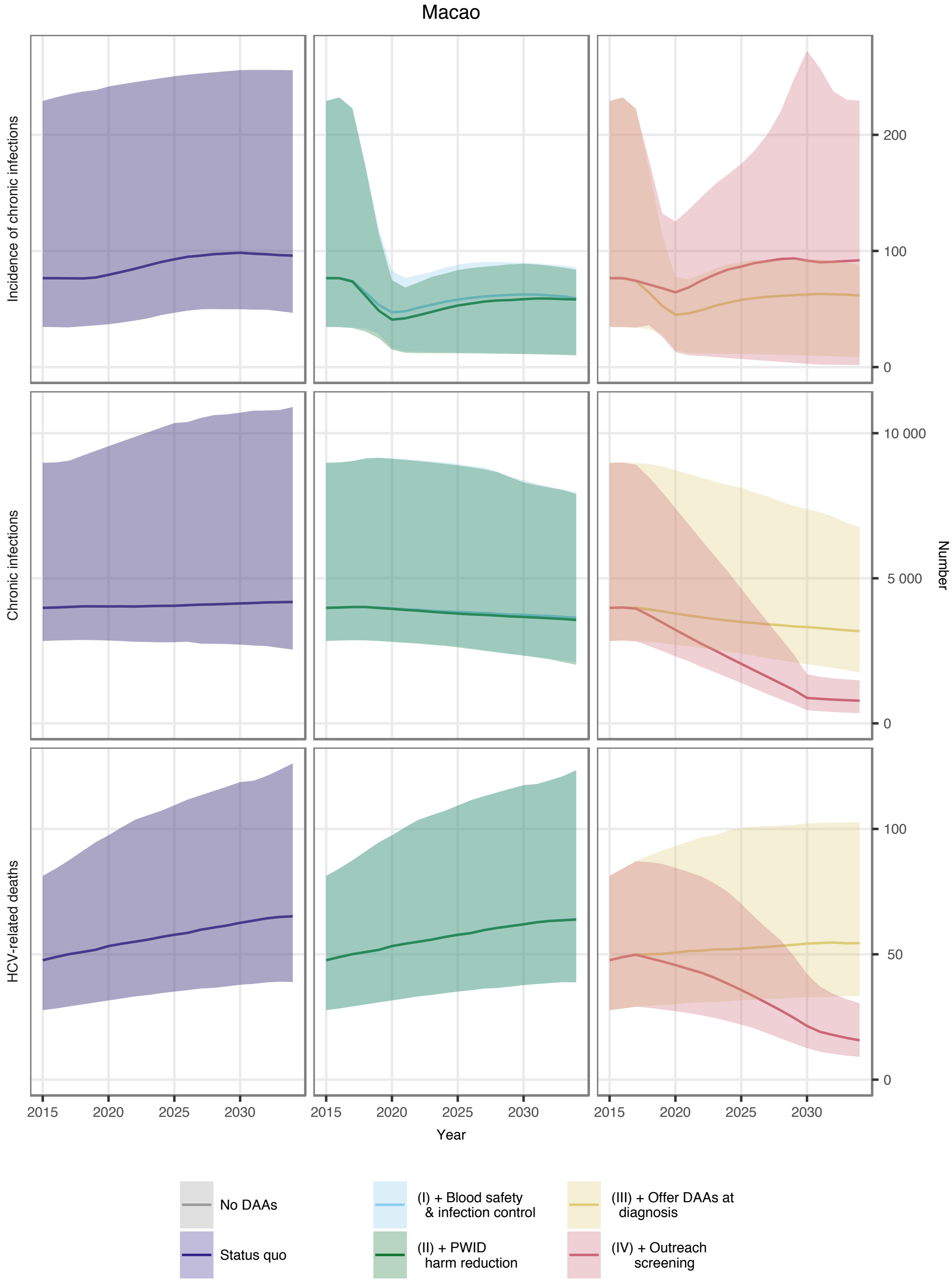
Macao
Incidence of chronic infections
Chronic infections
HCV-related deaths
Number
200
100
0
10 000
5 000
0
100
50
0
2015
2020
2025
2030
Year
No DAAs
Status quo
(I) + Blood safety & infection control
(II) + PWID harm reduction
(III) + Offer DAAs at diagnosis
(IV) + Outreach screening

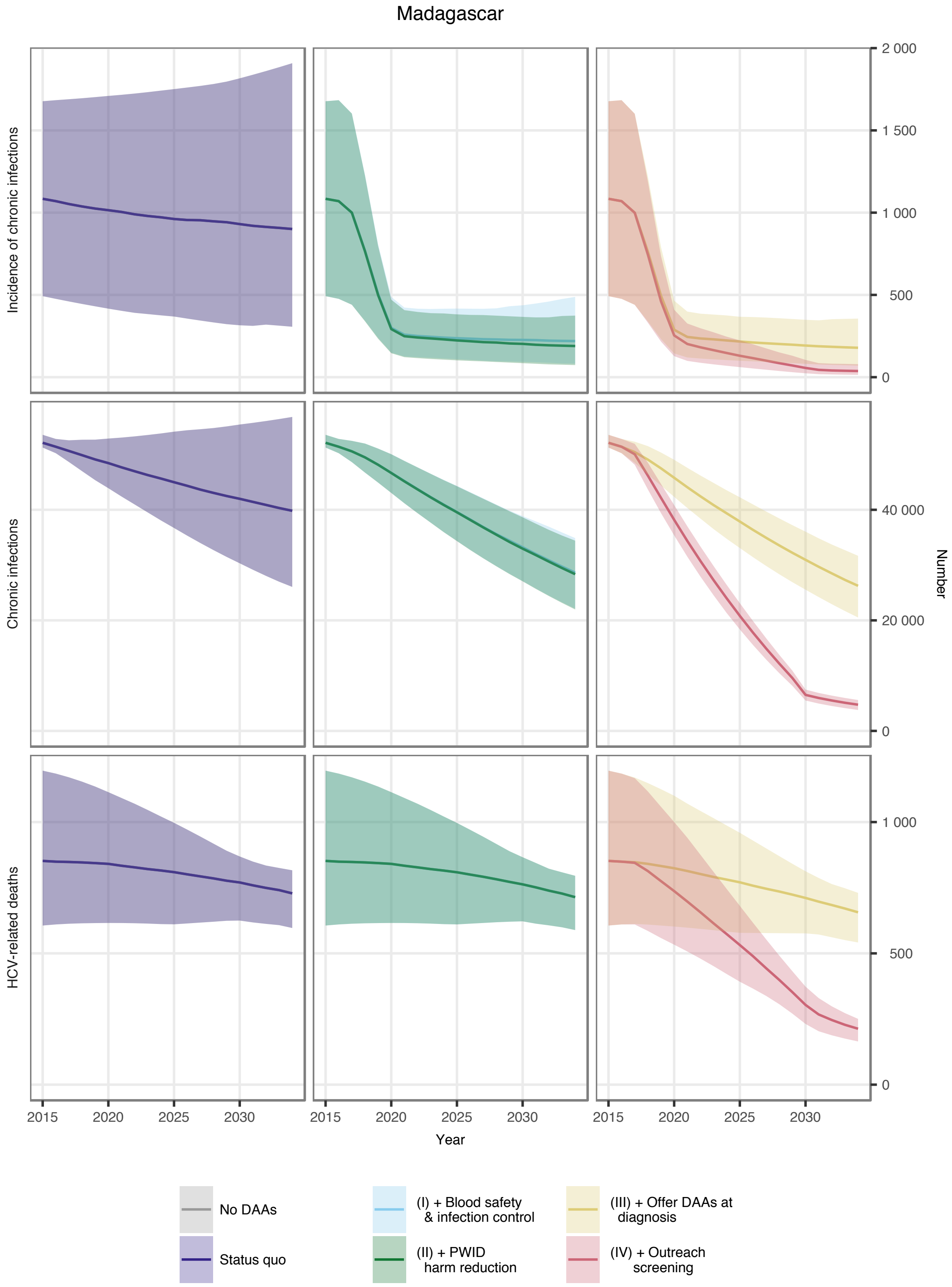
Madagascar
Incidence of chronic infections
Chronic infections
HCV-related deaths
Number
Year
2 000
1 500
1 000
500
0
40 000
20 000
0
1 000
500
0
2015
2020
2025
2030
No DAAs
Status quo
(I) + Blood safety & infection control
(II) + PWID harm reduction
(III) + Offer DAAs at diagnosis
(IV) + Outreach screening

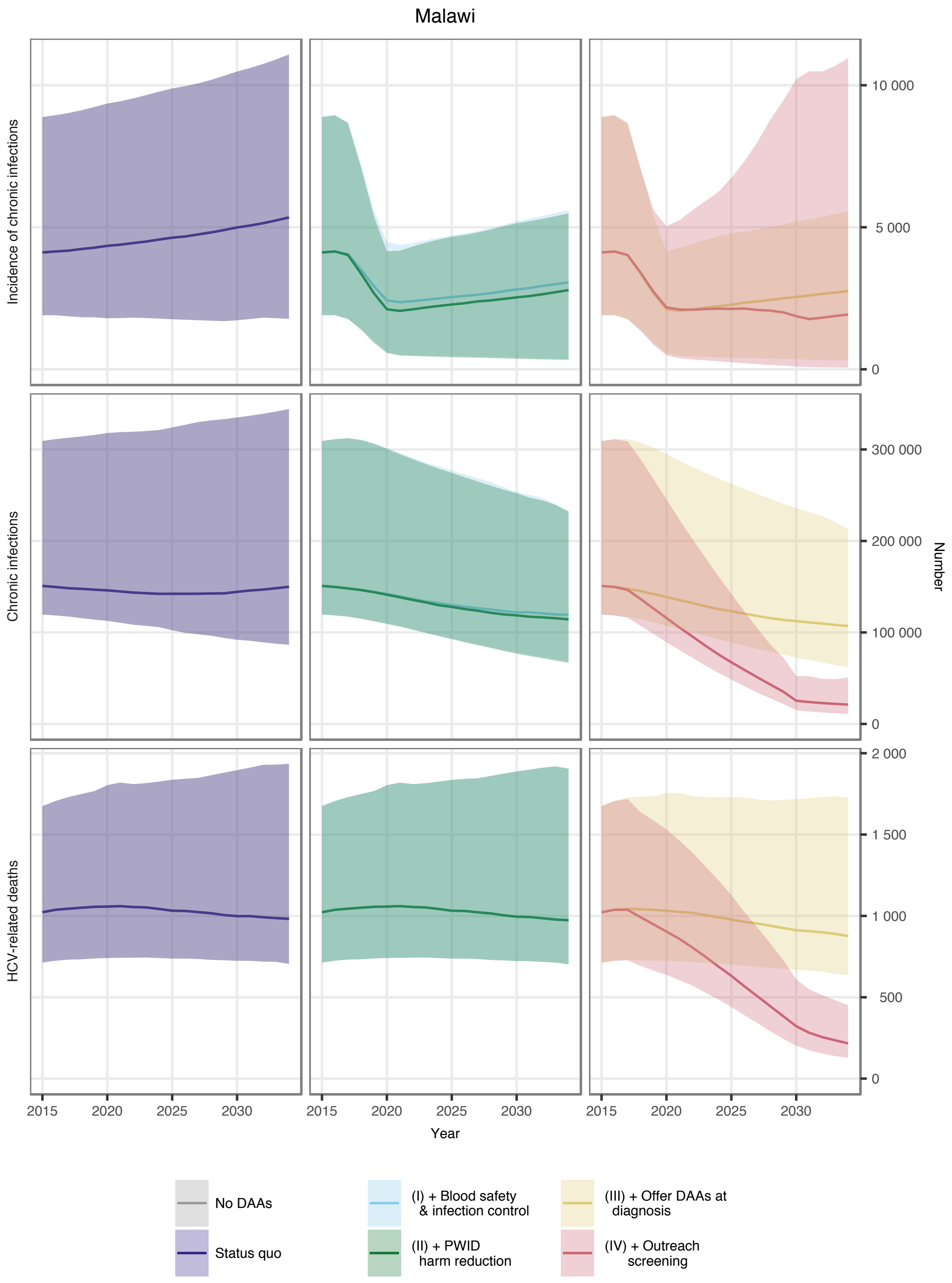
Malawi
Incidence of chronic infections
Chronic infections
HCV-related deaths
10 000
5 000
0
300 000
200 000
100 000
0
2 000
1 500
1 000
500
0
Number
2015
2020
2025
2030
Year
No DAAs
Status quo
(I) + Blood safety & infection control
(II) + PWID harm reduction
(III) + Offer DAAs at diagnosis
(IV) + Outreach screening

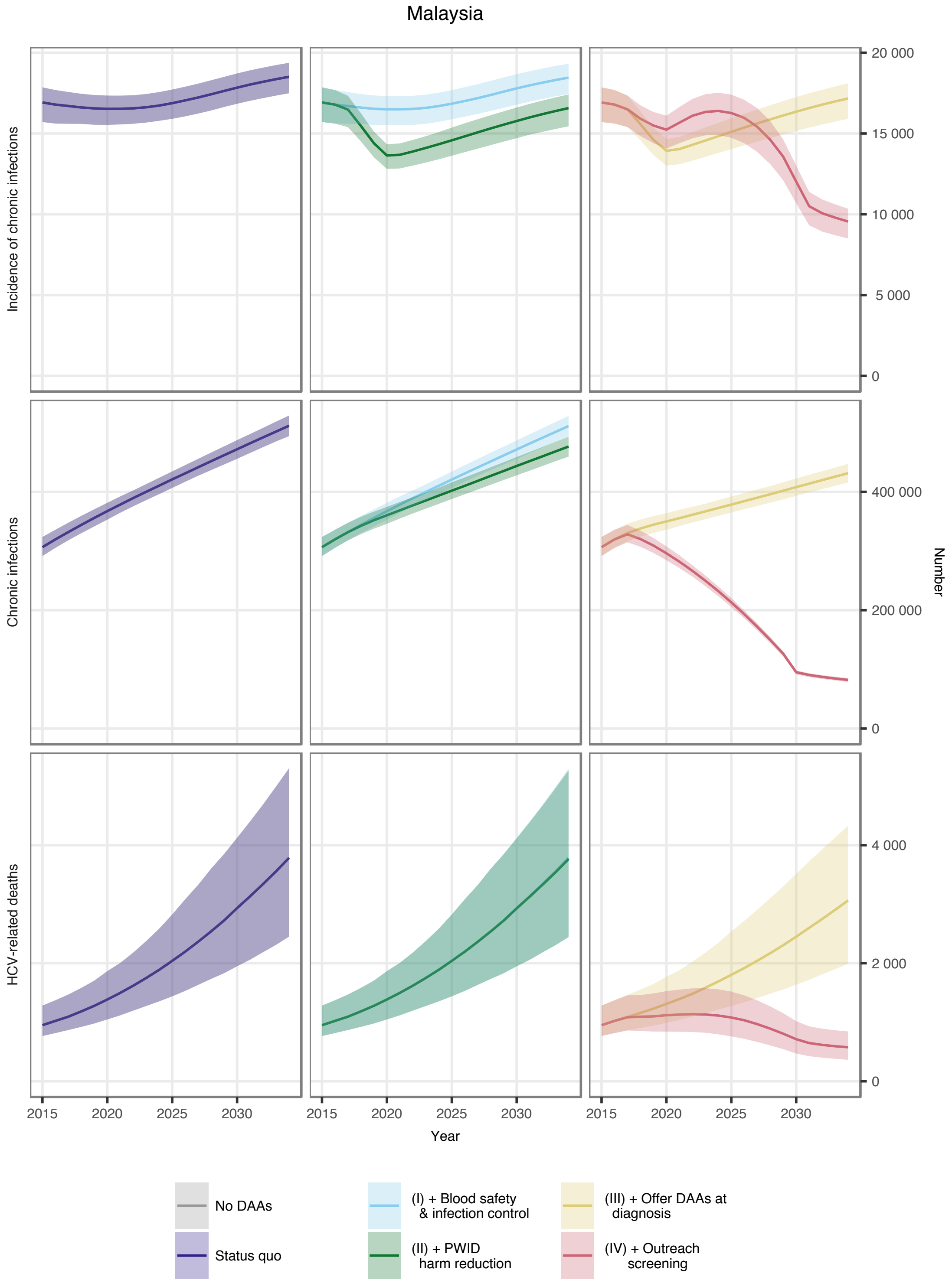
Malaysia
Incidence of chronic infections
Chronic infections
HCV-related deaths
Number
20 000
15 000
10 000
5 000
0
400 000
200 000
0
4 000
2 000
0
2015
2020
2025
2030
Year
No DAAs
Status quo
(I) + Blood safety & infection control
(II) + PWID harm reduction
(III) + Offer DAAs at diagnosis
(IV) + Outreach screening

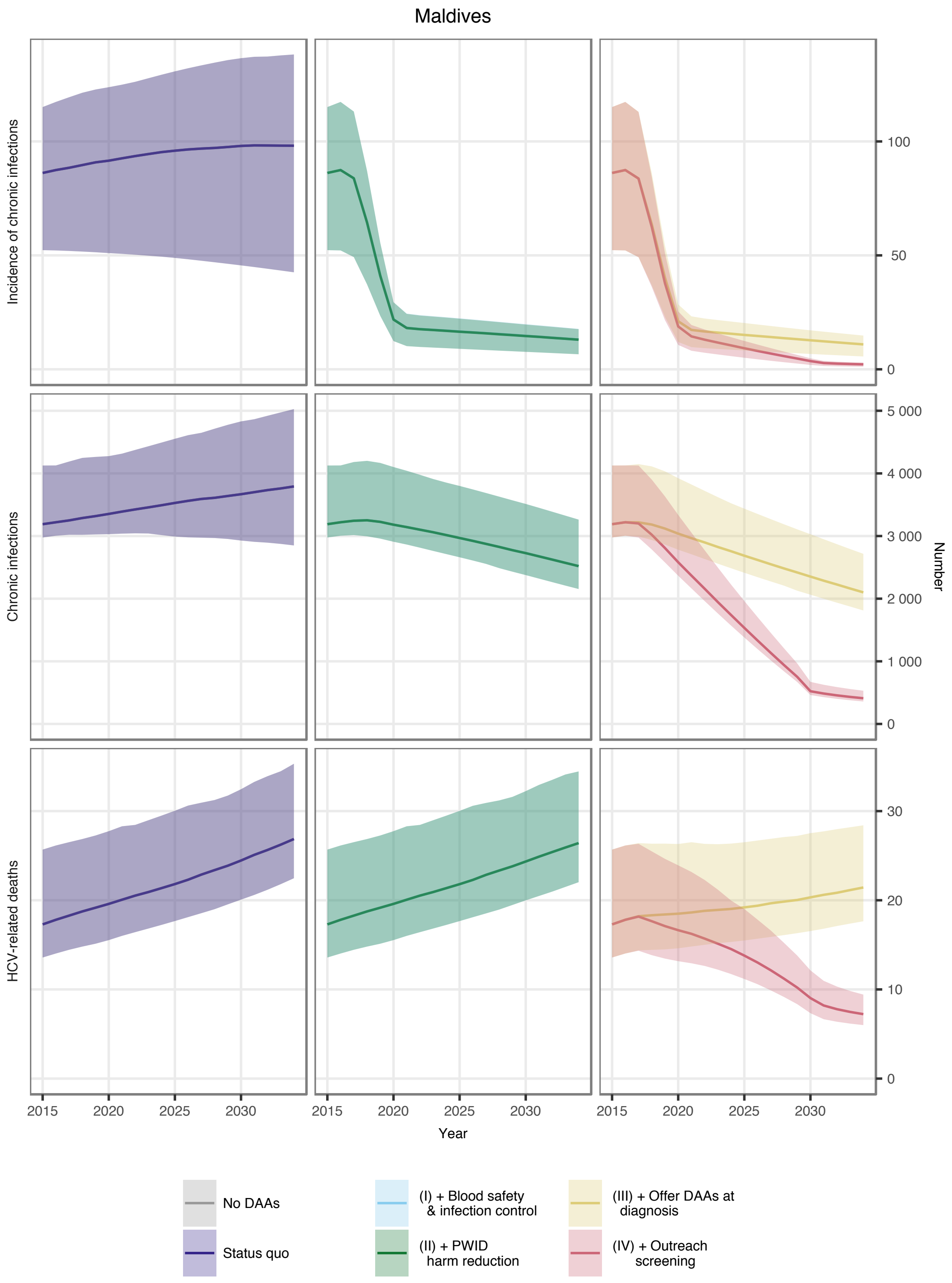

Maldives
Incidence of chronic infections
Chronic infections
HCV-related deaths
Number
Year
2015
2020
2025
2030
0
50
100
1 000
2 000
3 000
4 000
5 000
10
20
30
No DAAs
Status quo
(I) + Blood safety & infection control
(II) + PWID harm reduction
(III) + Offer DAAs at diagnosis
(IV) + Outreach screening

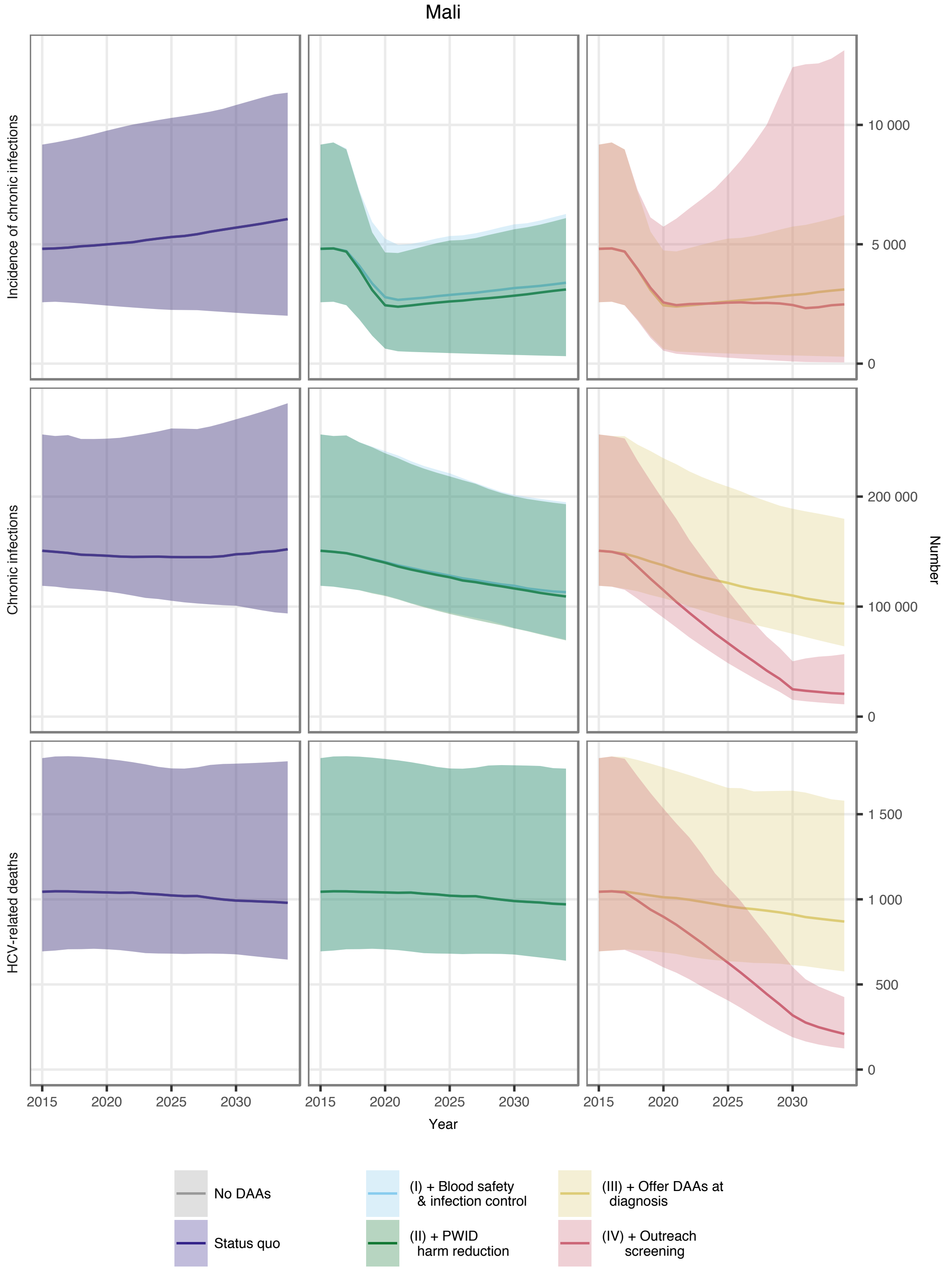

Mali
Incidence of chronic infections
Chronic infections
HCV-related deaths
Number
10 000
5 000
0
200 000
100 000
0
1 500
1 000
500
0
2015
2020
2025
2030
Year
No DAAs
Status quo
(I) + Blood safety & infection control
(II) + PWID harm reduction
(III) + Offer DAAs at diagnosis
(IV) + Outreach screening

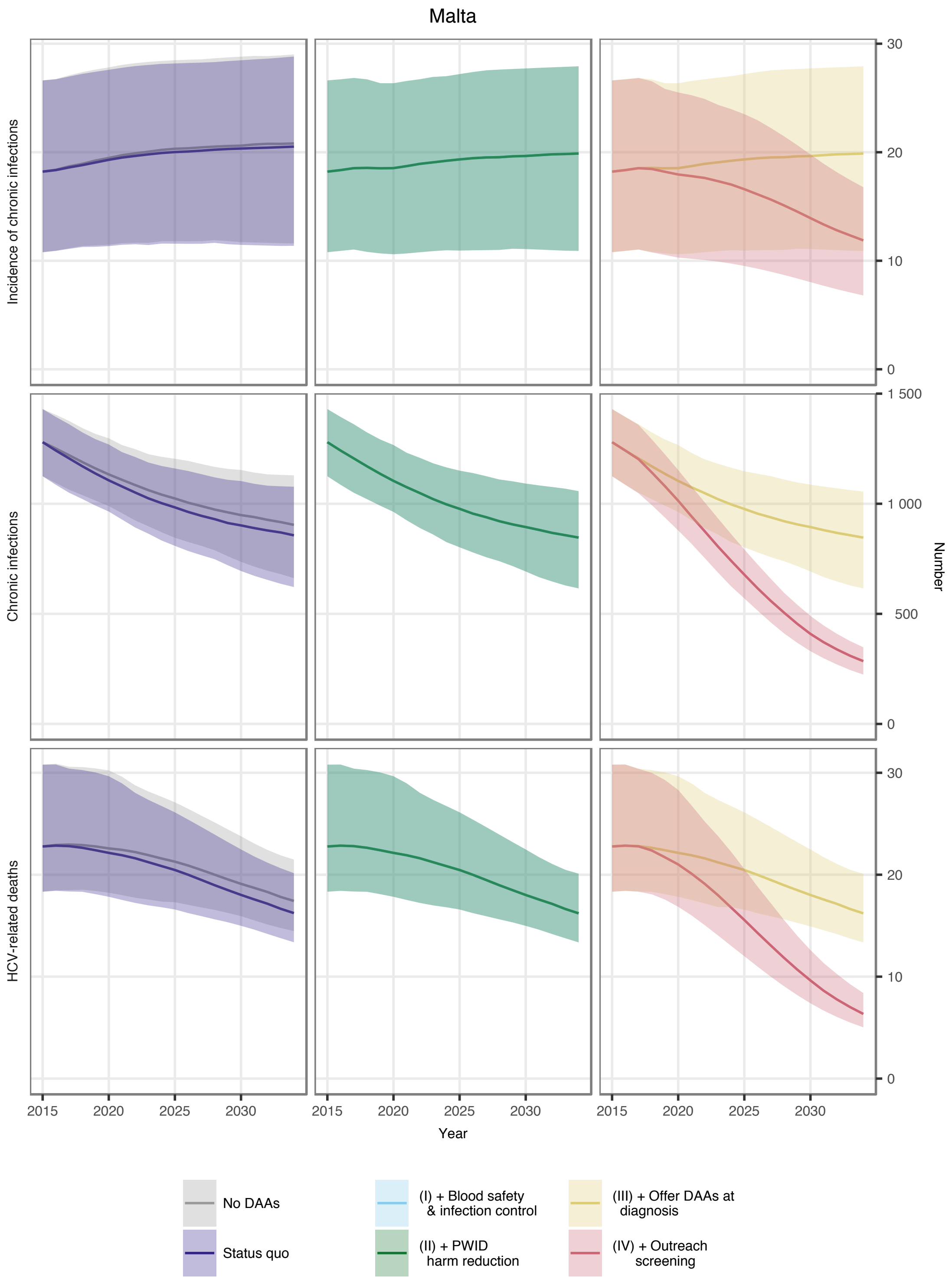
Malta
Incidence of chronic infections
Chronic infections
HCV-related deaths
Number
Year
0
10
20
30
500
1 000
1 500
2015
2020
2025
2030
No DAAs
Status quo
(I) + Blood safety & infection control
(II) + PWID harm reduction
(III) + Offer DAAs at diagnosis
(IV) + Outreach screening

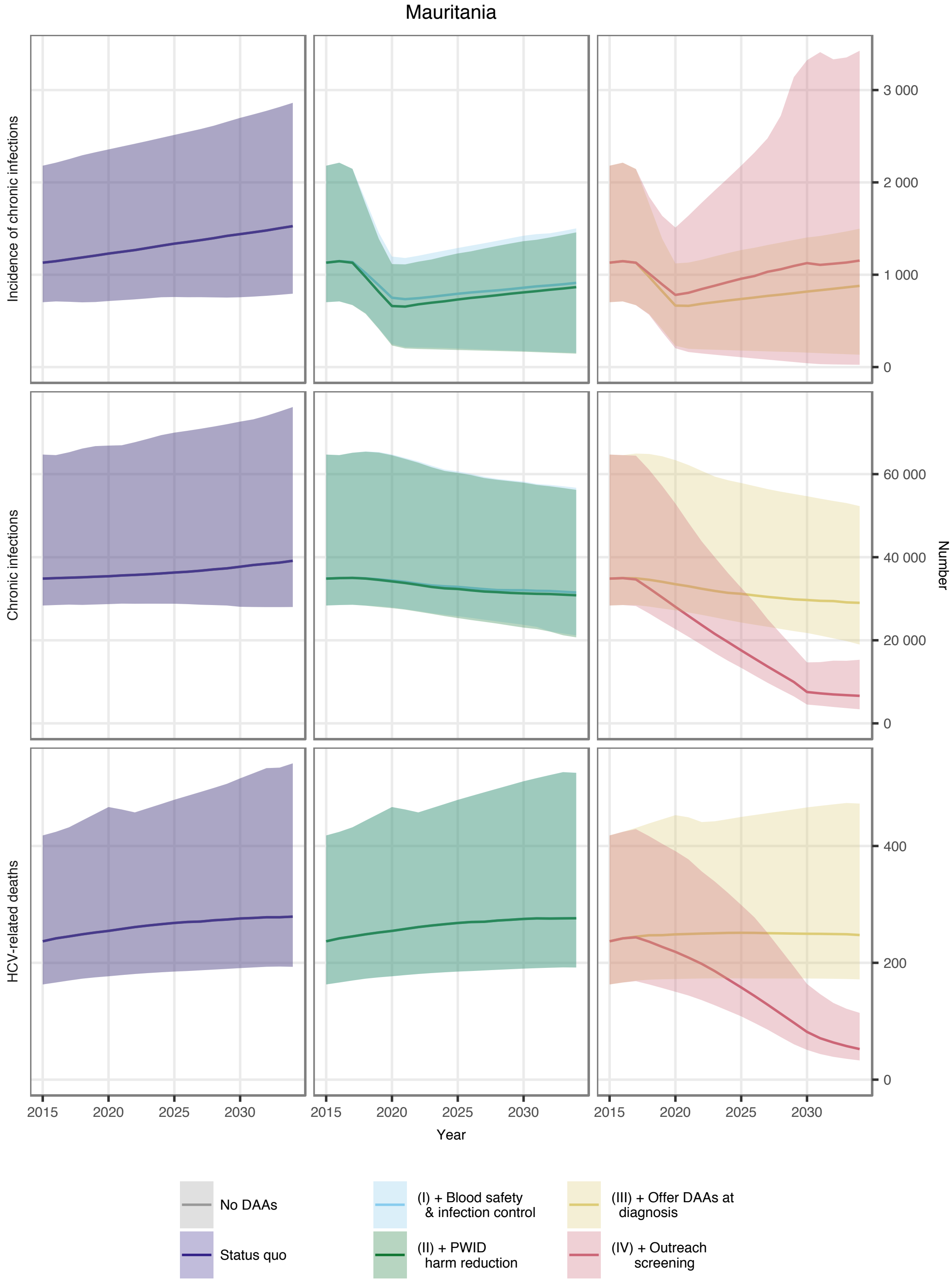
Mauritania
Incidence of chronic infections
Chronic infections
HCV-related deaths
Number
3 000
2 000
1 000
0
60 000
40 000
20 000
0
400
200
0
2015
2020
2025
2030
Year
No DAAs
Status quo
(I) + Blood safety & infection control
(II) + PWID harm reduction
(III) + Offer DAAs at diagnosis
(IV) + Outreach screening

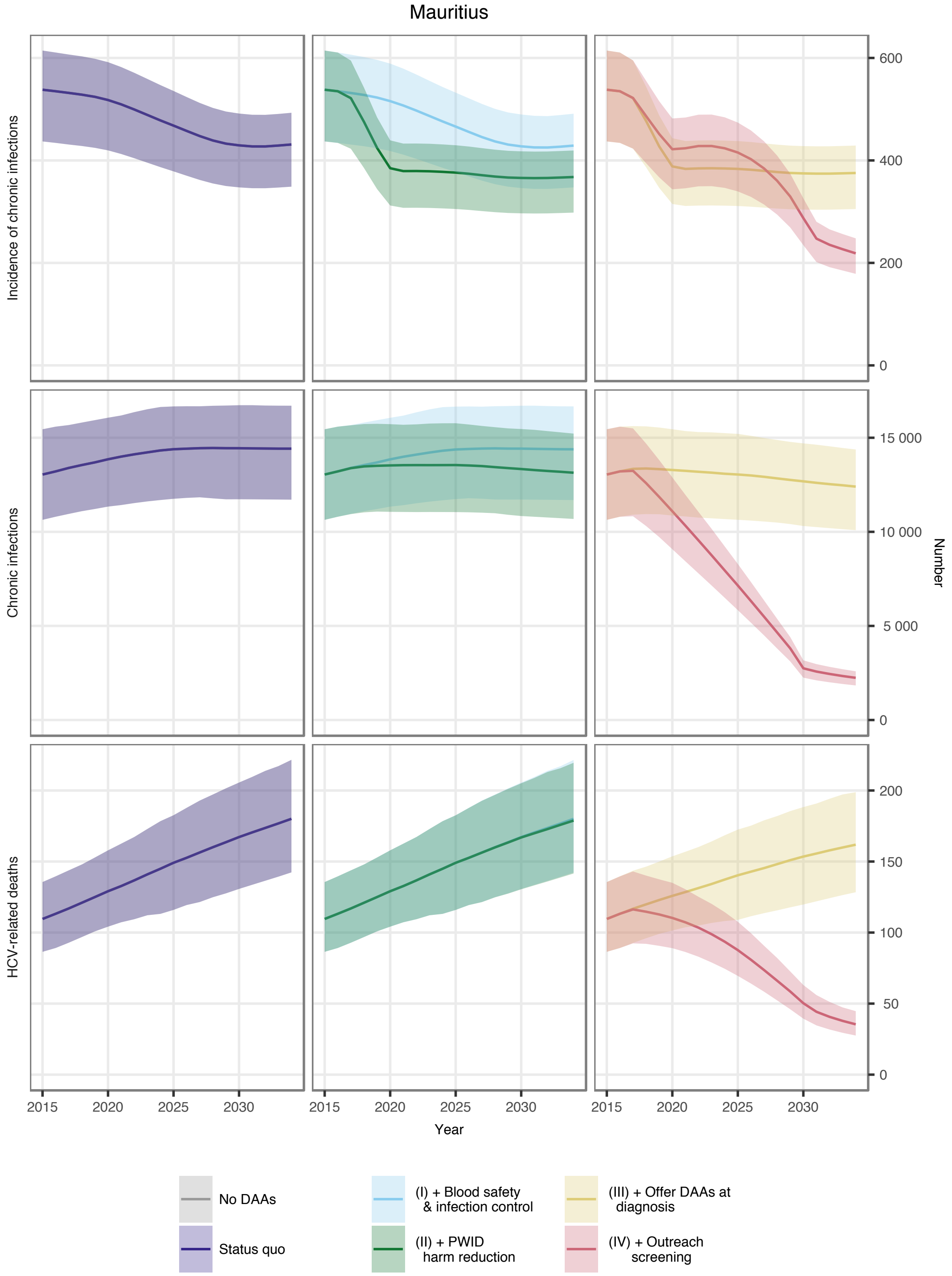
Mauritius
Incidence of chronic infections
Chronic infections
HCV-related deaths
Number
0
200
400
600
0
5 000
10 000
15 000
0
50
100
150
200
2015
2020
2025
2030
Year
No DAAs
Status quo
(I) + Blood safety & infection control
(II) + PWID harm reduction
(III) + Offer DAAs at diagnosis
(IV) + Outreach screening

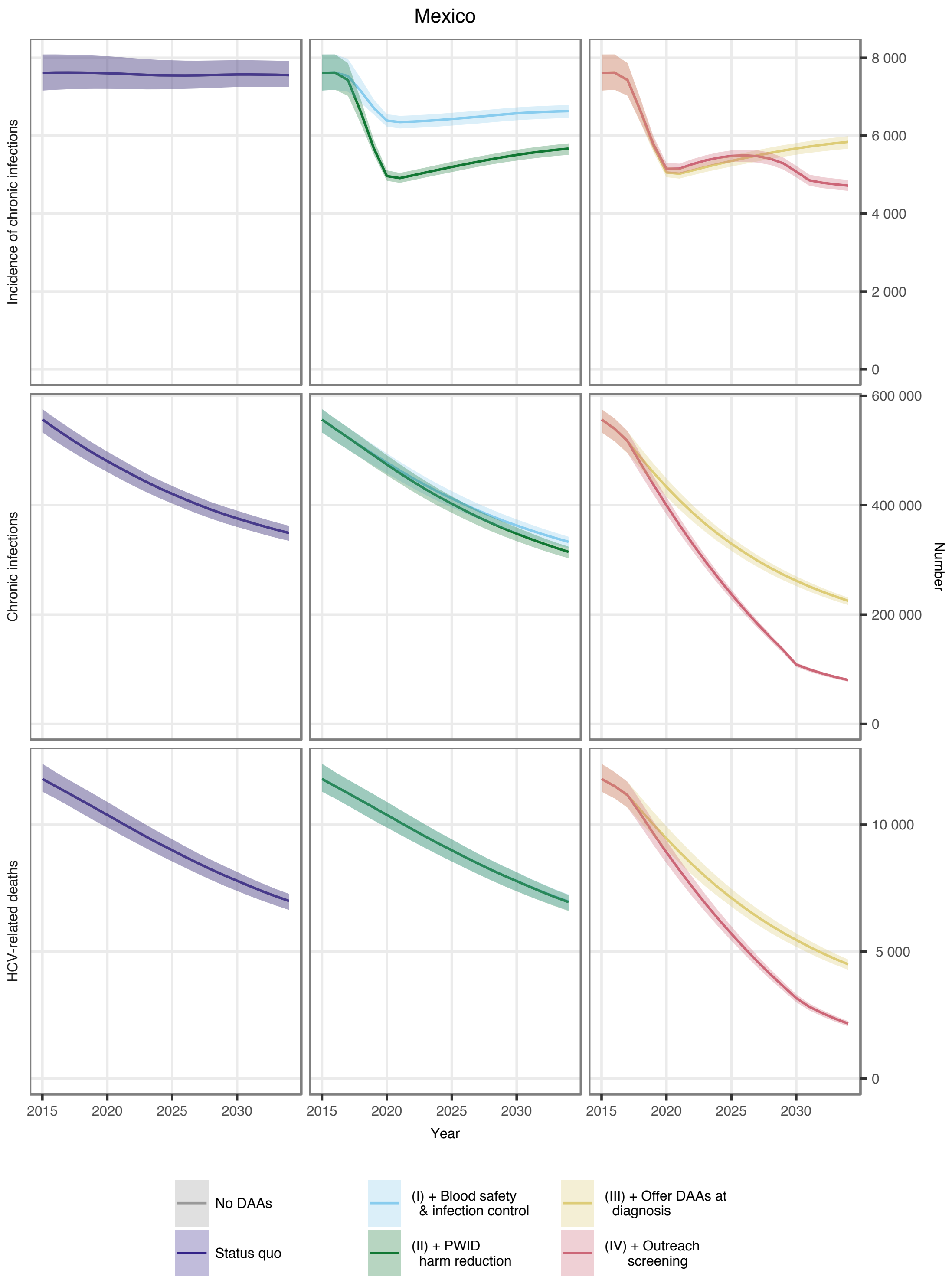
Mexico
Incidence of chronic infections
Chronic infections
HCV-related deaths
Number
Year
8 000
6 000
4 000
2 000
0
600 000
400 000
200 000
0
10 000
5 000
0
2015
2020
2025
2030
No DAAs
Status quo
(I) + Blood safety & infection control
(II) + PWID harm reduction
(III) + Offer DAAs at diagnosis
(IV) + Outreach screening

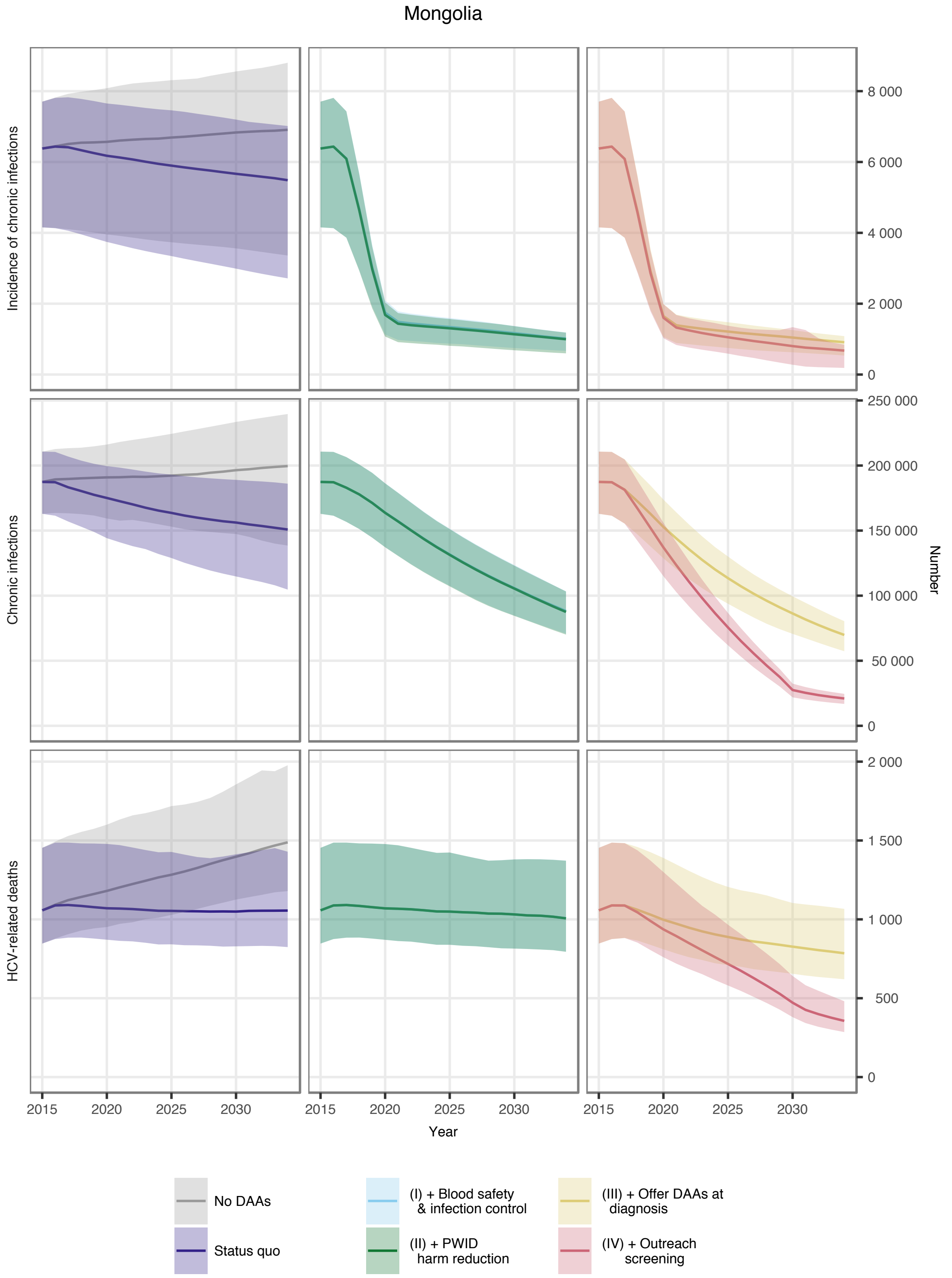
Mongolia
Incidence of chronic infections
Chronic infections
HCV-related deaths
8 000
6 000
4 000
2 000
0
250 000
200 000
150 000
100 000
50 000
0
2 000
1 500
1 000
500
0
Number
2015
2020
2025
2030
Year
No DAAs
Status quo
(I) + Blood safety & infection control
(II) + PWID harm reduction
(III) + Offer DAAs at diagnosis
(IV) + Outreach screening

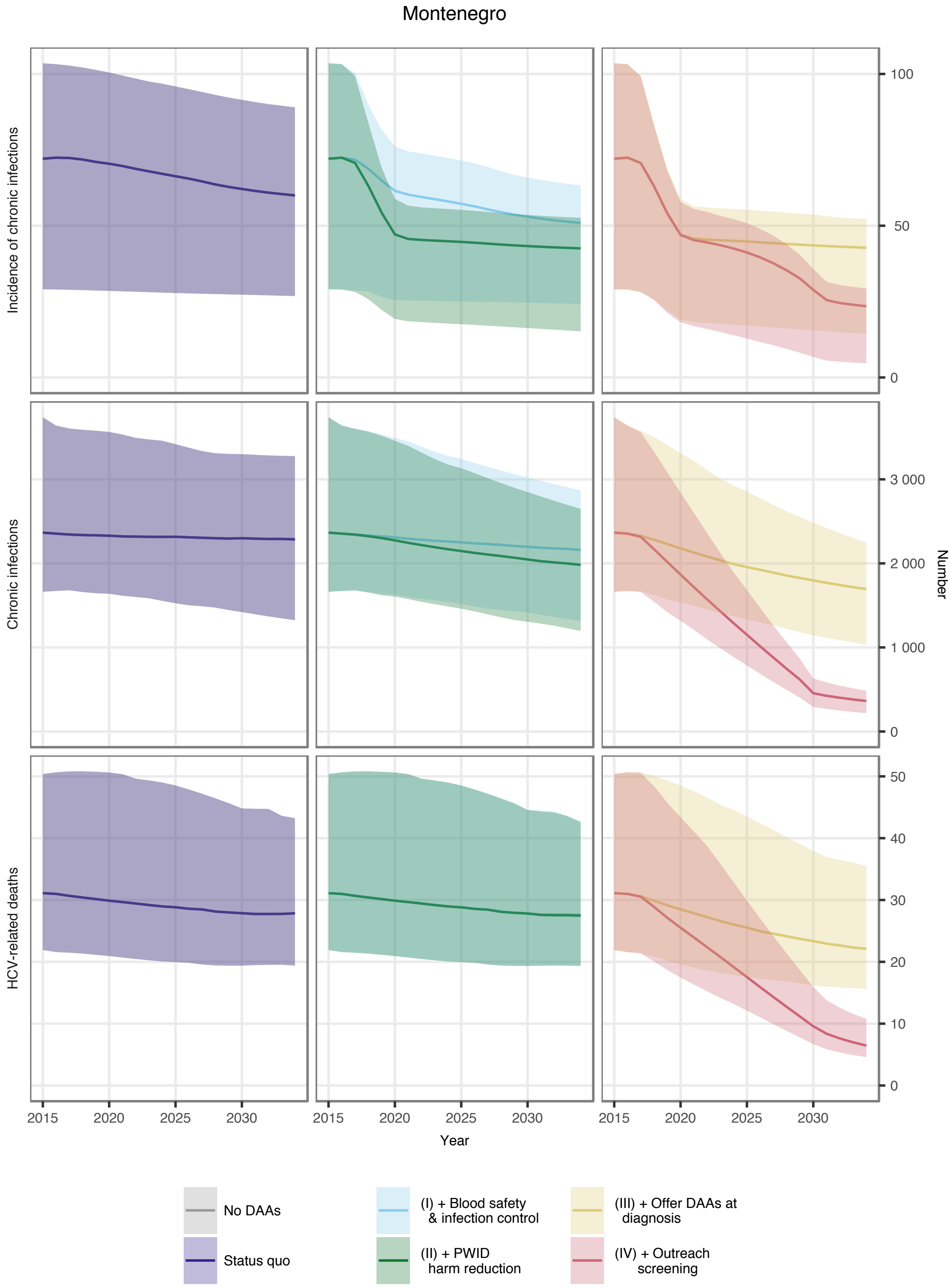
Montenegro
Incidence of chronic infections
Chronic infections
HCV-related deaths
Number
Year
100
50
0
3 000
2 000
1 000
0
50
40
30
20
10
0
2015
2020
2025
2030
No DAAs
Status quo
(I) + Blood safety & infection control
(II) + PWID harm reduction
(III) + Offer DAAs at diagnosis
(IV) + Outreach screening

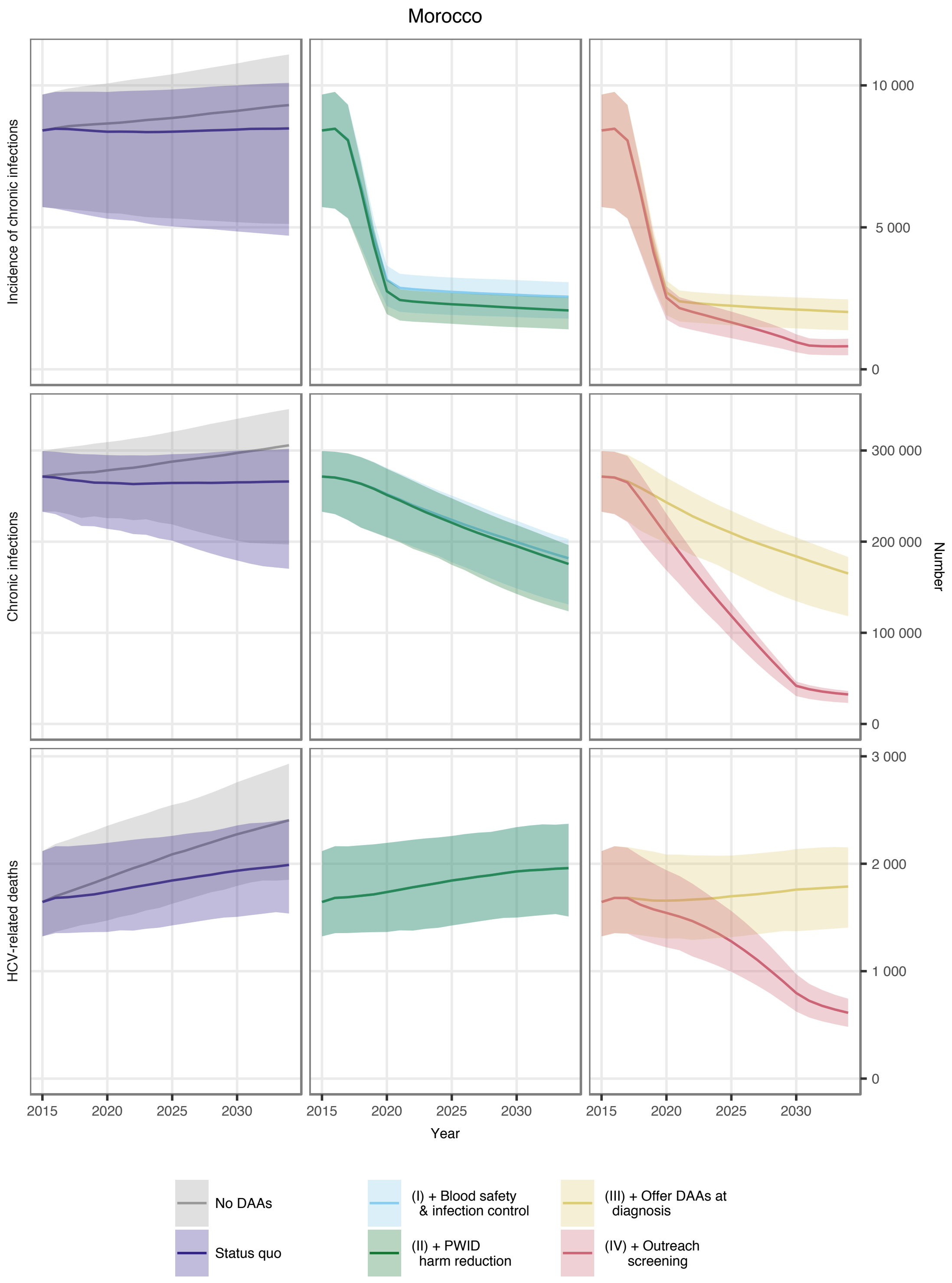
Morocco
Incidence of chronic infections
Chronic infections
HCV-related deaths
Number
10 000
5 000
0
300 000
200 000
100 000
0
3 000
2 000
1 000
0
2015
2020
2025
2030
Year
No DAAs
Status quo
(I) + Blood safety & infection control
(II) + PWID harm reduction
(III) + Offer DAAs at diagnosis
(IV) + Outreach screening

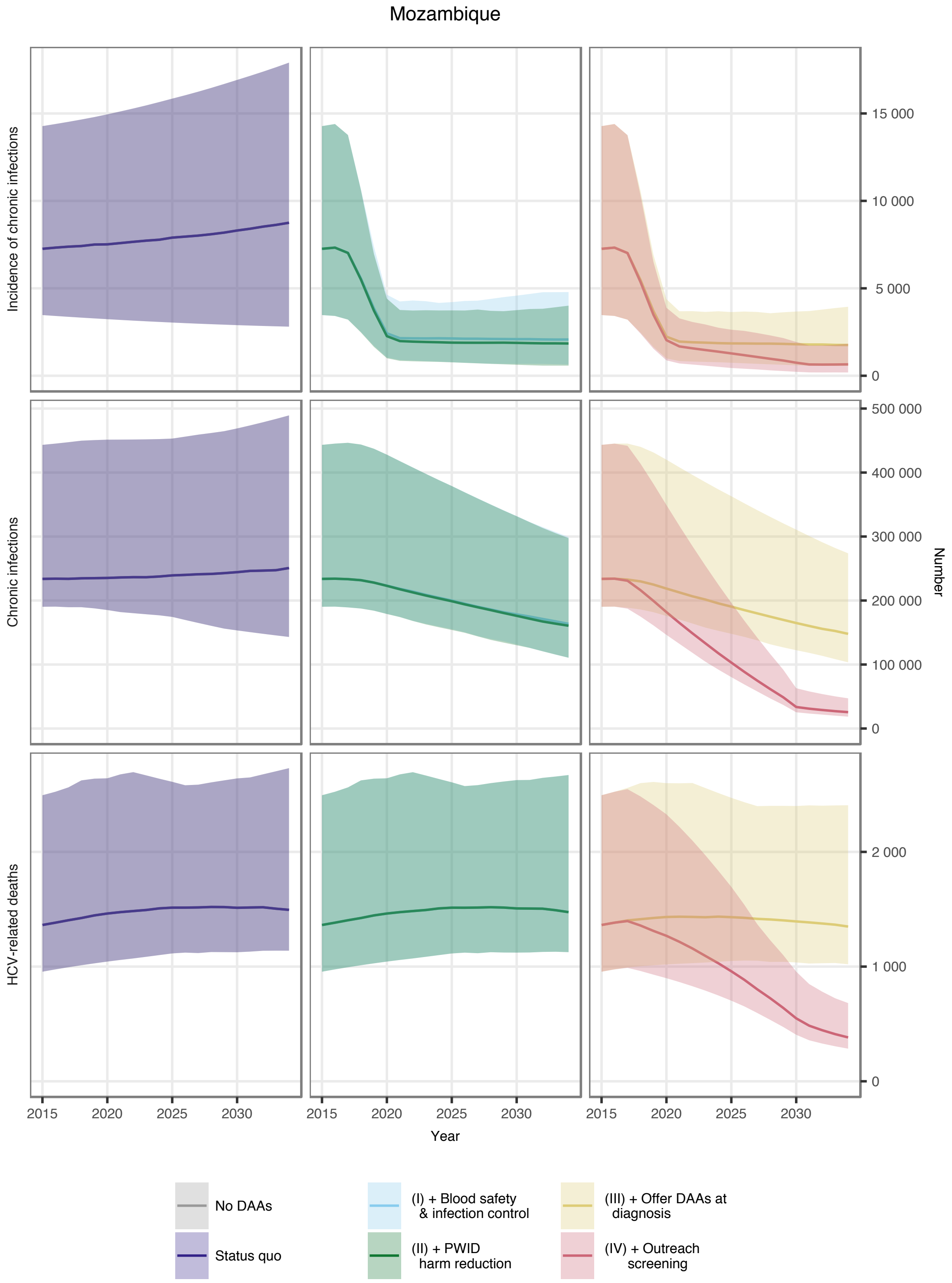

Mozambique
Incidence of chronic infections
Chronic infections
HCV-related deaths
Number
15 000
10 000
5 000
0
500 000
400 000
300 000
200 000
100 000
0
2 000
1 000
0
2015
2020
2025
2030
Year
No DAAs
Status quo
(I) + Blood safety & infection control
(II) + PWID harm reduction
(III) + Offer DAAs at diagnosis
(IV) + Outreach screening

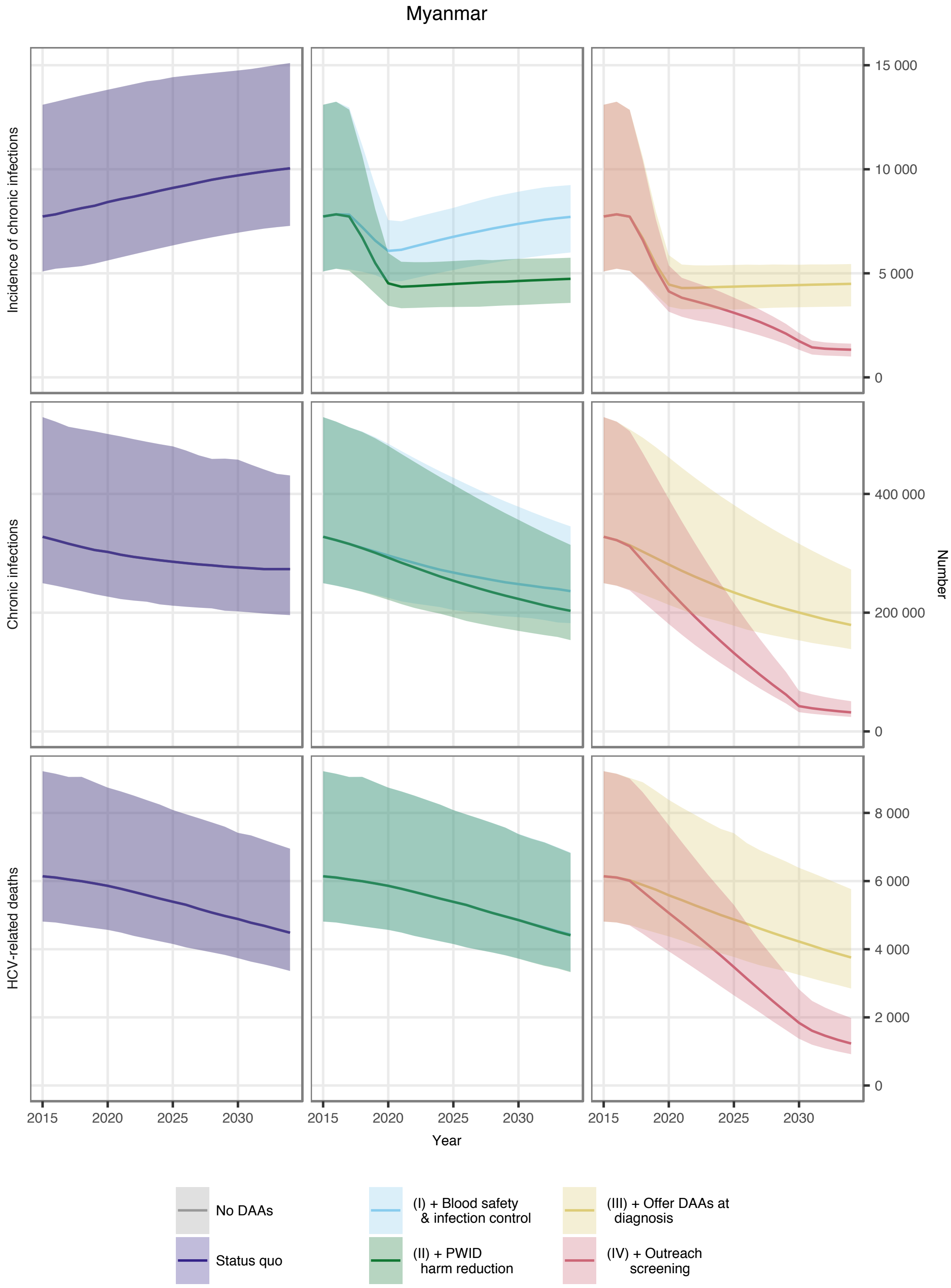
Myanmar
Incidence of chronic infections
Chronic infections
HCV-related deaths
15 000
10 000
5 000
0
400 000
200 000
0
8 000
6 000
4 000
2 000
0
Number
2015
2020
2025
2030
Year
No DAAs
Status quo
(I) + Blood safety & infection control
(II) + PWID harm reduction
(III) + Offer DAAs at diagnosis
(IV) + Outreach screening

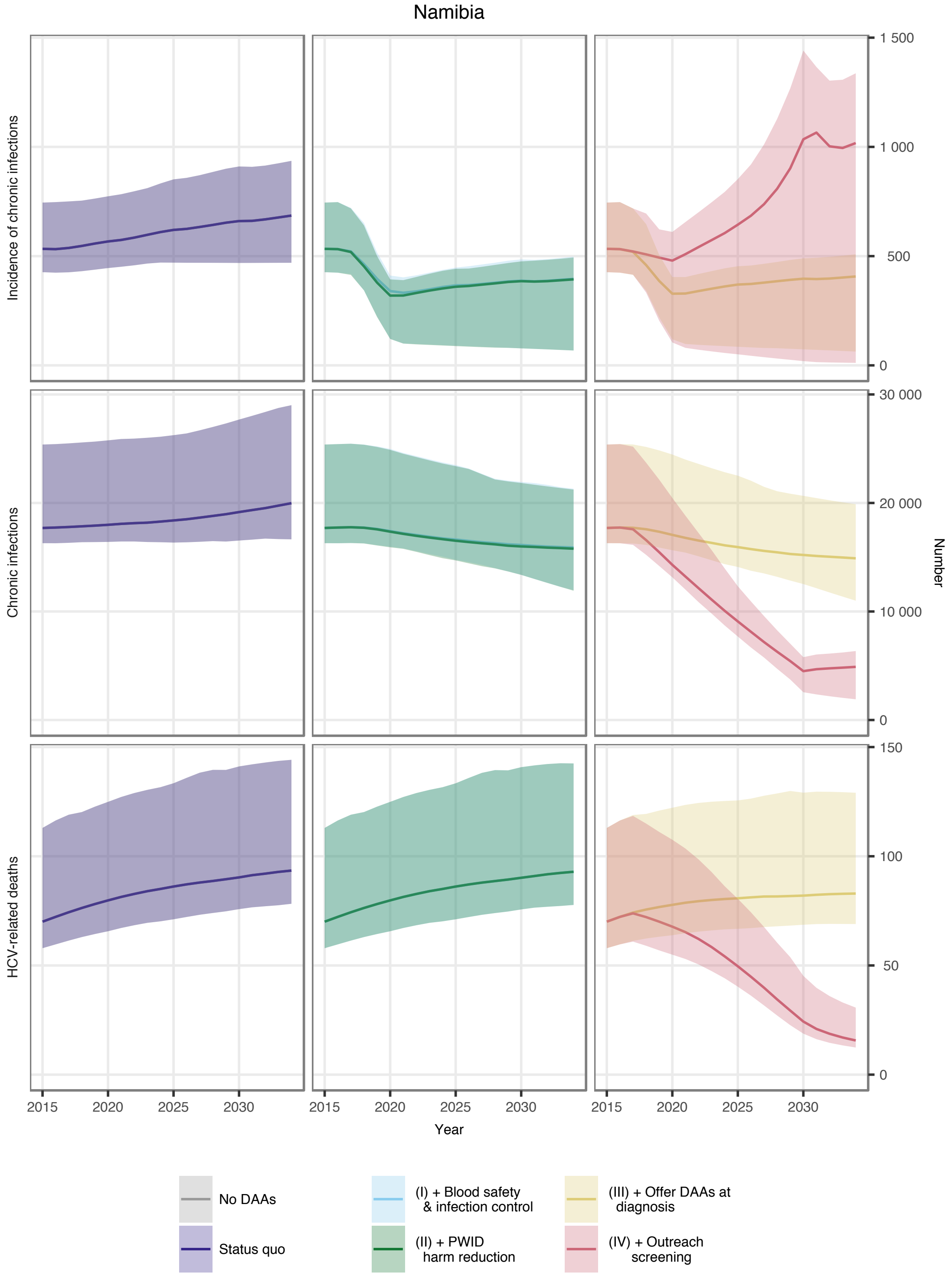
Namibia
Incidence of chronic infections
Chronic infections
HCV-related deaths
Number
Year
2015
2020
2025
2030
0
500
1 000
1 500
0
10 000
20 000
30 000
0
50
100
150
No DAAs
Status quo
(I) + Blood safety & infection control
(II) + PWID harm reduction
(III) + Offer DAAs at diagnosis
(IV) + Outreach screening

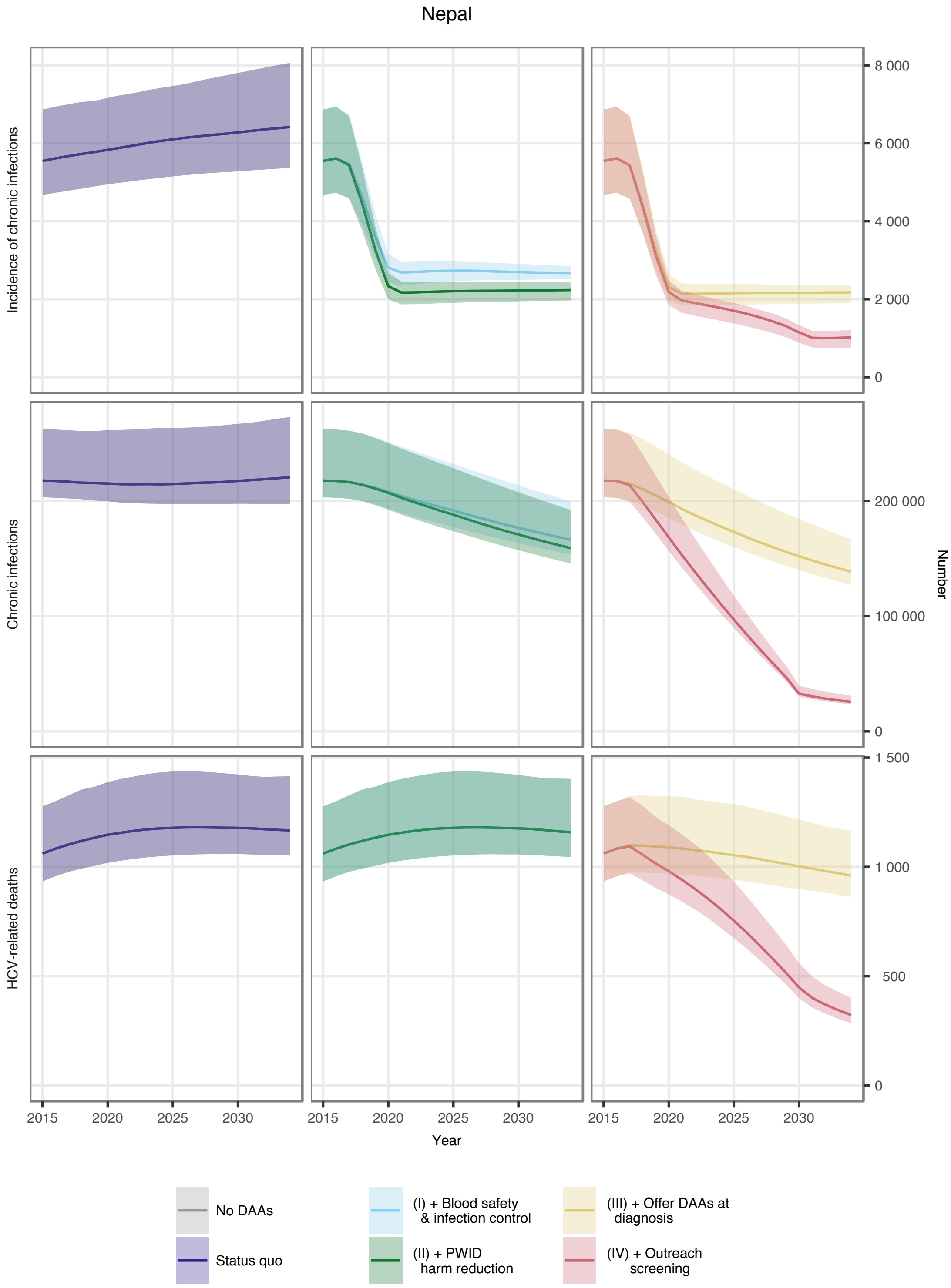
Nepal
Incidence of chronic infections
Chronic infections
HCV-related deaths
8 000
6 000
4 000
2 000
0
200 000
100 000
0
1 500
1 000
500
0
Number
2015
2020
2025
2030
Year
No DAAs
Status quo
(I) + Blood safety & infection control
(II) + PWID harm reduction
(III) + Offer DAAs at diagnosis
(IV) + Outreach screening

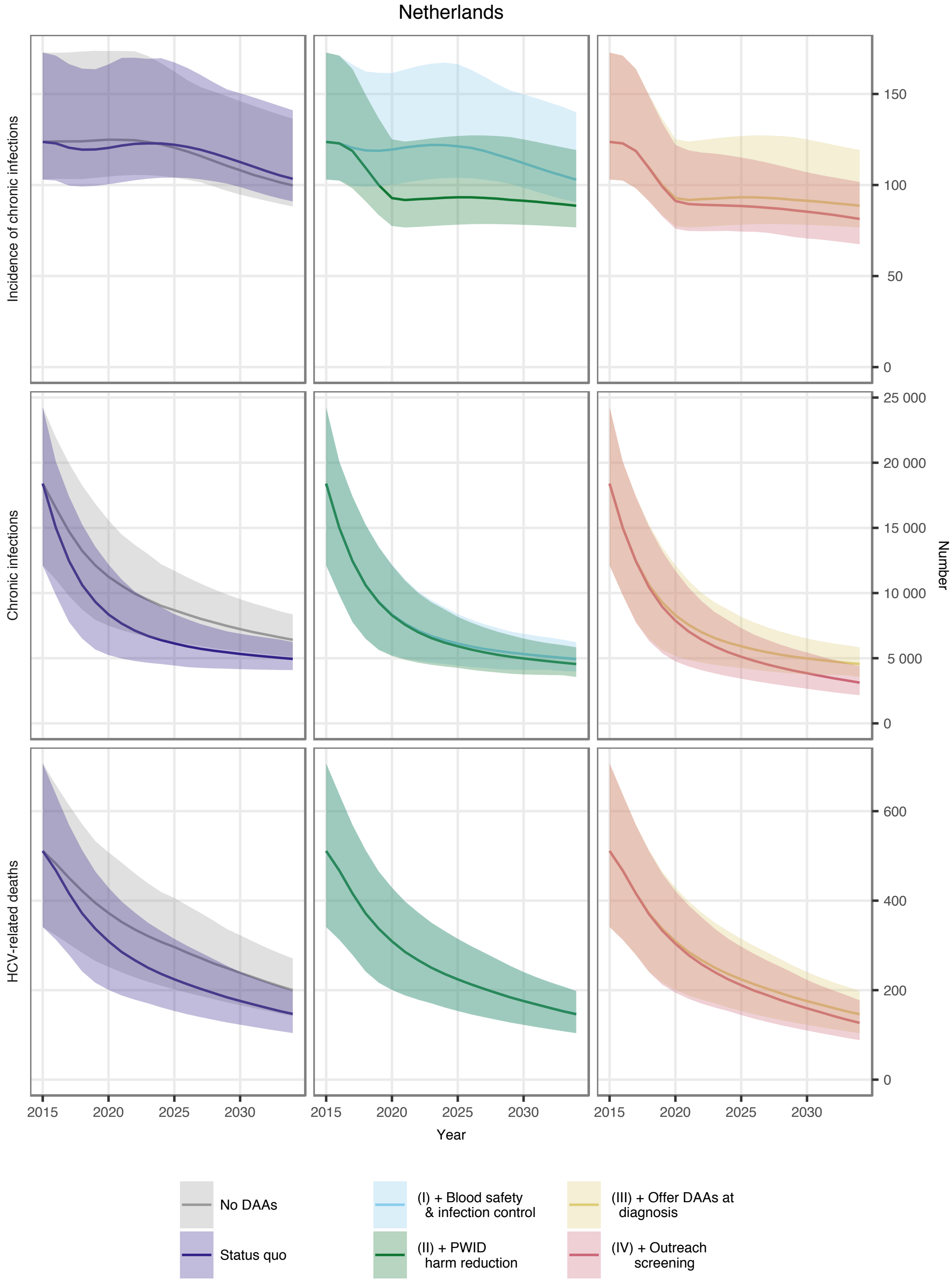

Netherlands
Incidence of chronic infections
Chronic infections
HCV-related deaths
Number
Year
0
50
100
150
0
5 000
10 000
15 000
20 000
25 000
0
200
400
600
2015
2020
2025
2030
No DAAs
Status quo
(I) + Blood safety & infection control
(II) + PWID harm reduction
(III) + Offer DAAs at diagnosis
(IV) + Outreach screening

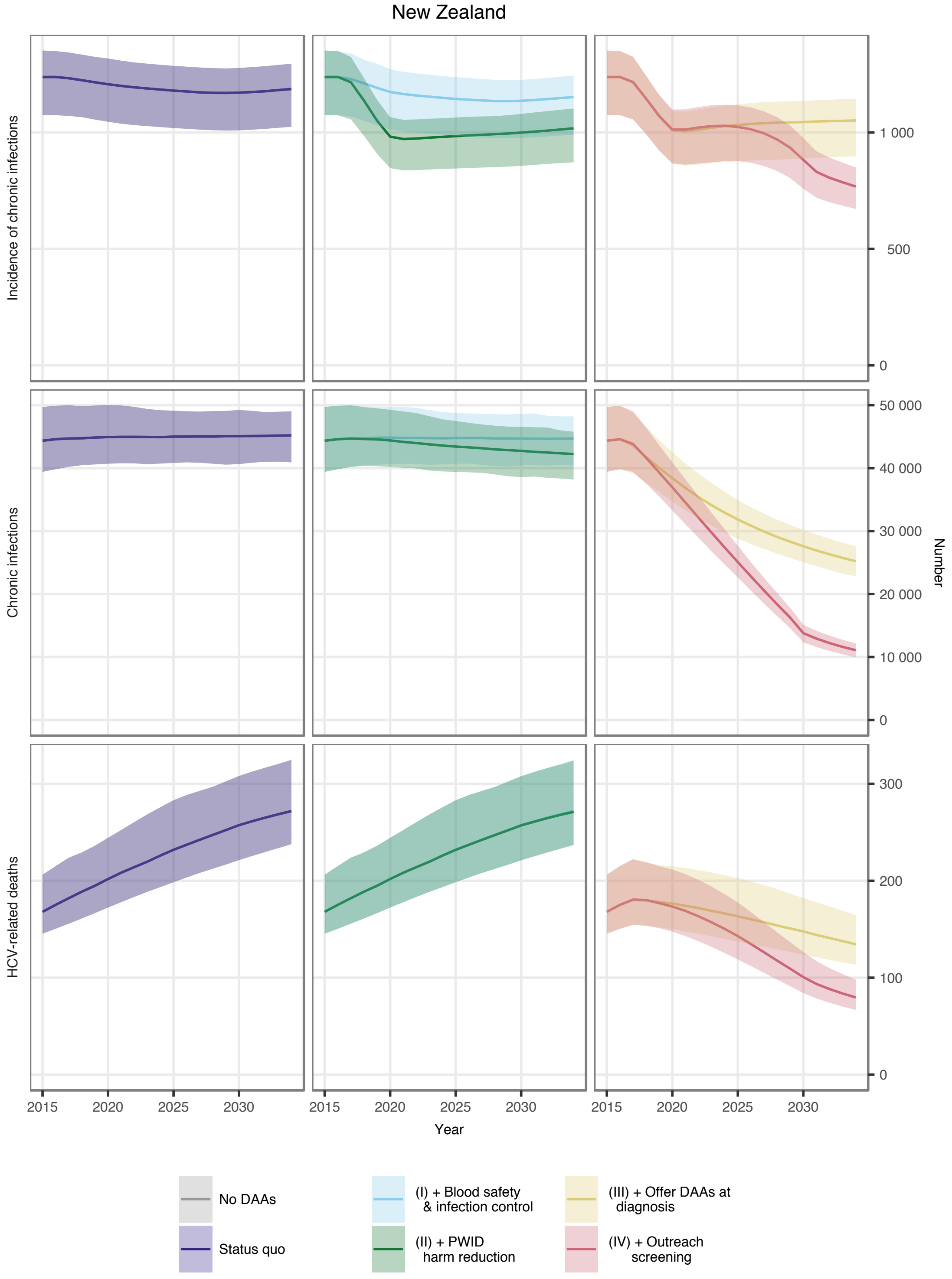
New Zealand
Incidence of chronic infections
Chronic infections
HCV-related deaths
Number
1 000
500
0
50 000
40 000
30 000
20 000
10 000
0
300
200
100
0
2015
2020
2025
2030
Year
No DAAs
Status quo
(I) + Blood safety & infection control
(II) + PWID harm reduction
(III) + Offer DAAs at diagnosis
(IV) + Outreach screening

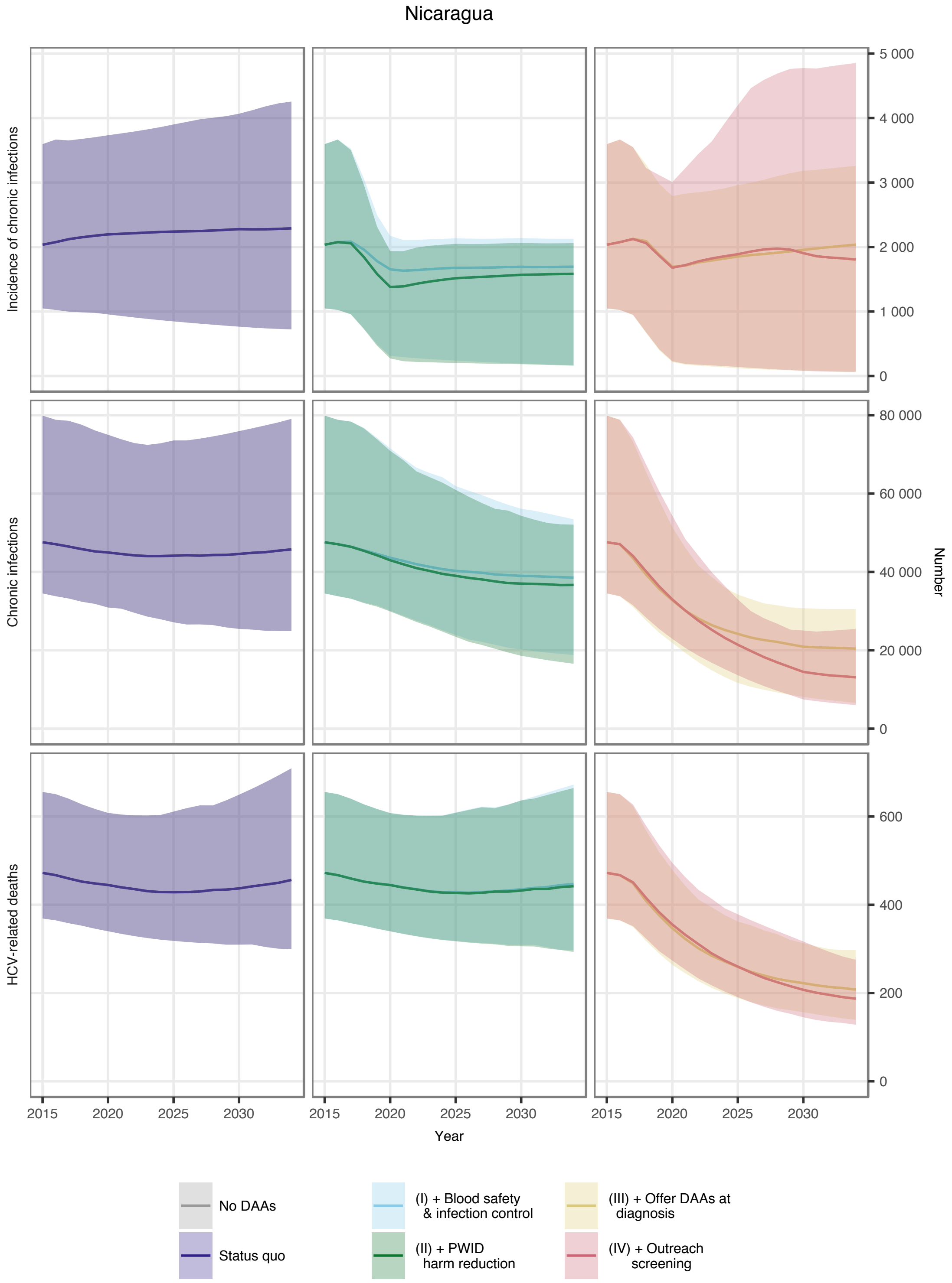
Nicaragua
Incidence of chronic infections
Chronic infections
HCV-related deaths
Number
5 000
4 000
3 000
2 000
1 000
0
80 000
60 000
40 000
20 000
0
600
400
200
0
2015
2020
2025
2030
Year
No DAAs
Status quo
(I) + Blood safety & infection control
(II) + PWID harm reduction
(III) + Offer DAAs at diagnosis
(IV) + Outreach screening

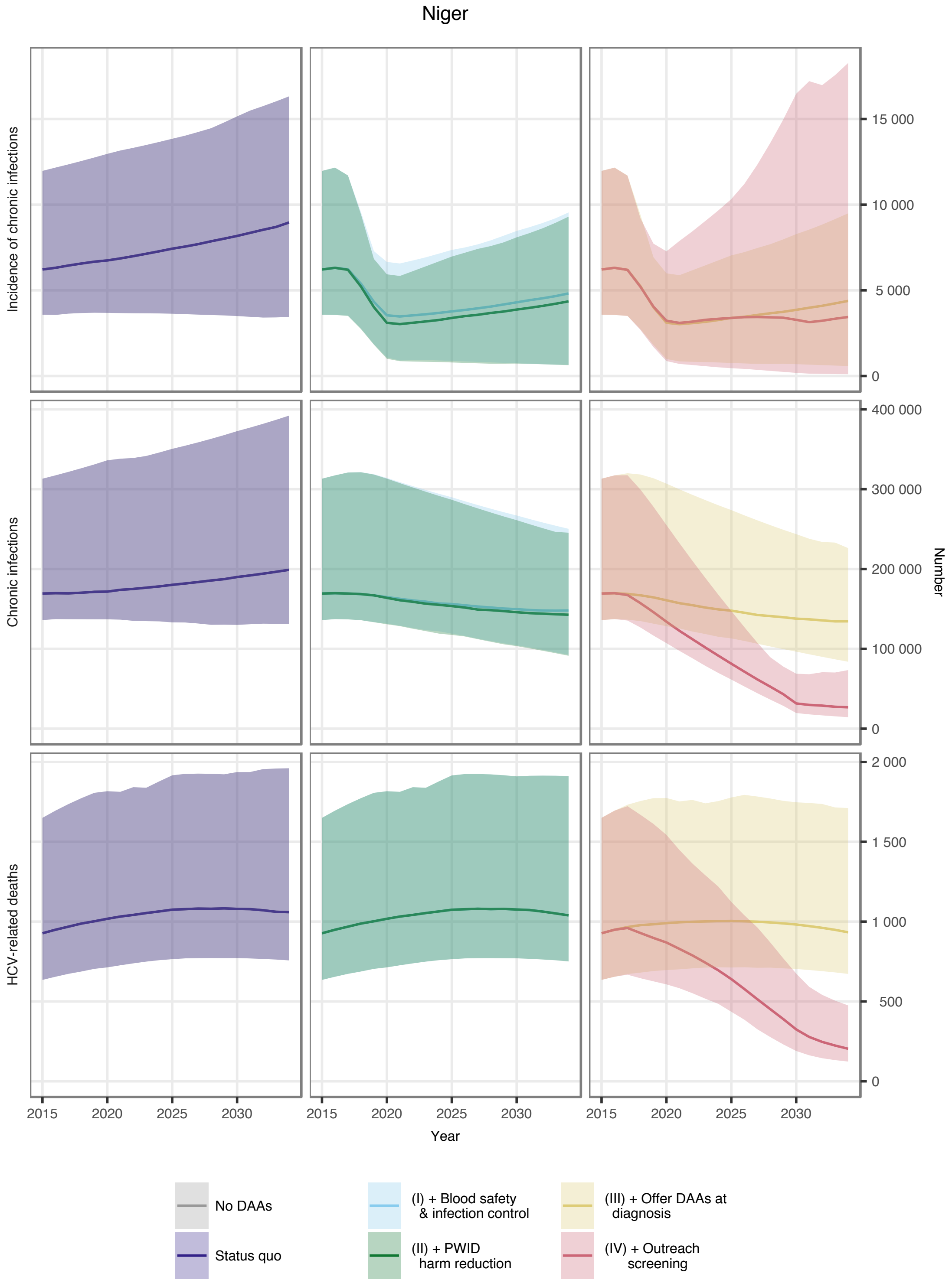
Niger
Incidence of chronic infections
Chronic infections
HCV-related deaths
Number
Year
2015
2020
2025
2030
0
5 000
10 000
15 000
100 000
200 000
300 000
400 000
500
1 000
1 500
2 000
No DAAs
Status quo
(I) + Blood safety & infection control
(II) + PWID harm reduction
(III) + Offer DAAs at diagnosis
(IV) + Outreach screening

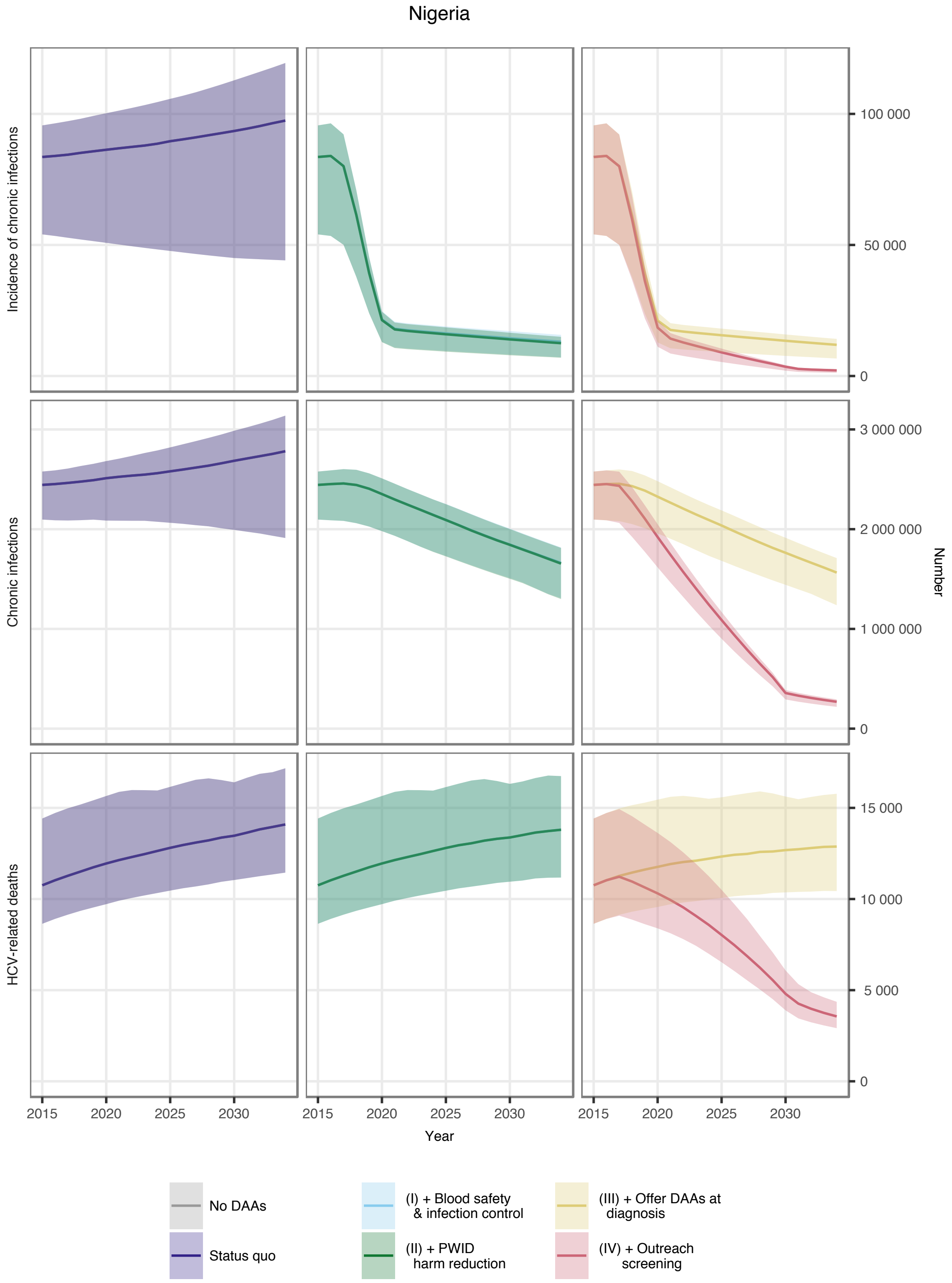
Nigeria
Incidence of chronic infections
Chronic infections
HCV-related deaths
100 000
50 000
0
3 000 000
2 000 000
1 000 000
0
15 000
10 000
5 000
0
Number
2015
2020
2025
2030
Year
No DAAs
Status quo
(I) + Blood safety & infection control
(II) + PWID harm reduction
(III) + Offer DAAs at diagnosis
(IV) + Outreach screening

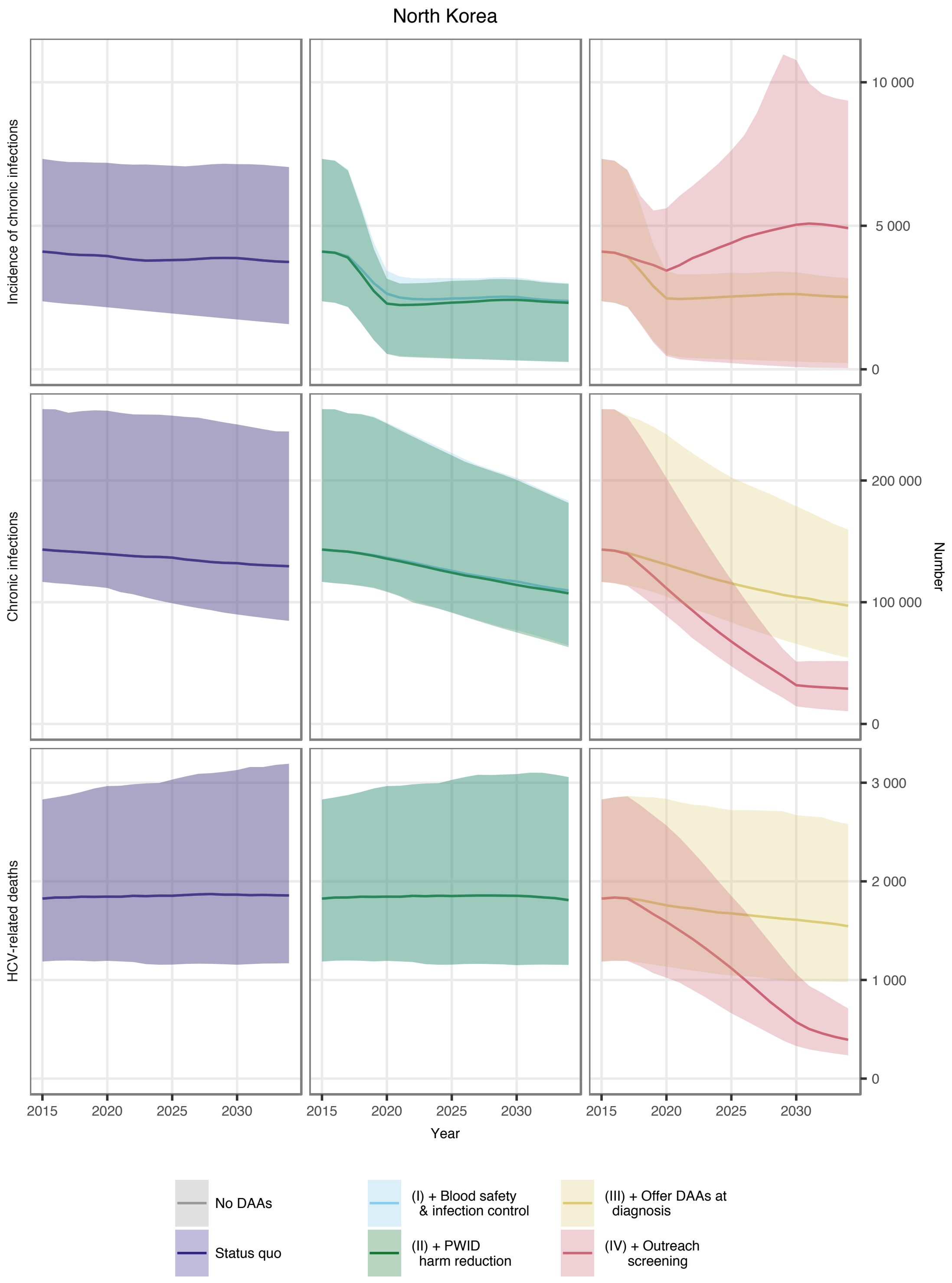
North Korea
Incidence of chronic infections
Chronic infections
HCV-related deaths
Number
Year
10 000
5 000
0
200 000
100 000
0
3 000
2 000
1 000
0
2015
2020
2025
2030
No DAAs
Status quo
(I) + Blood safety & infection control
(II) + PWID harm reduction
(III) + Offer DAAs at diagnosis
(IV) + Outreach screening

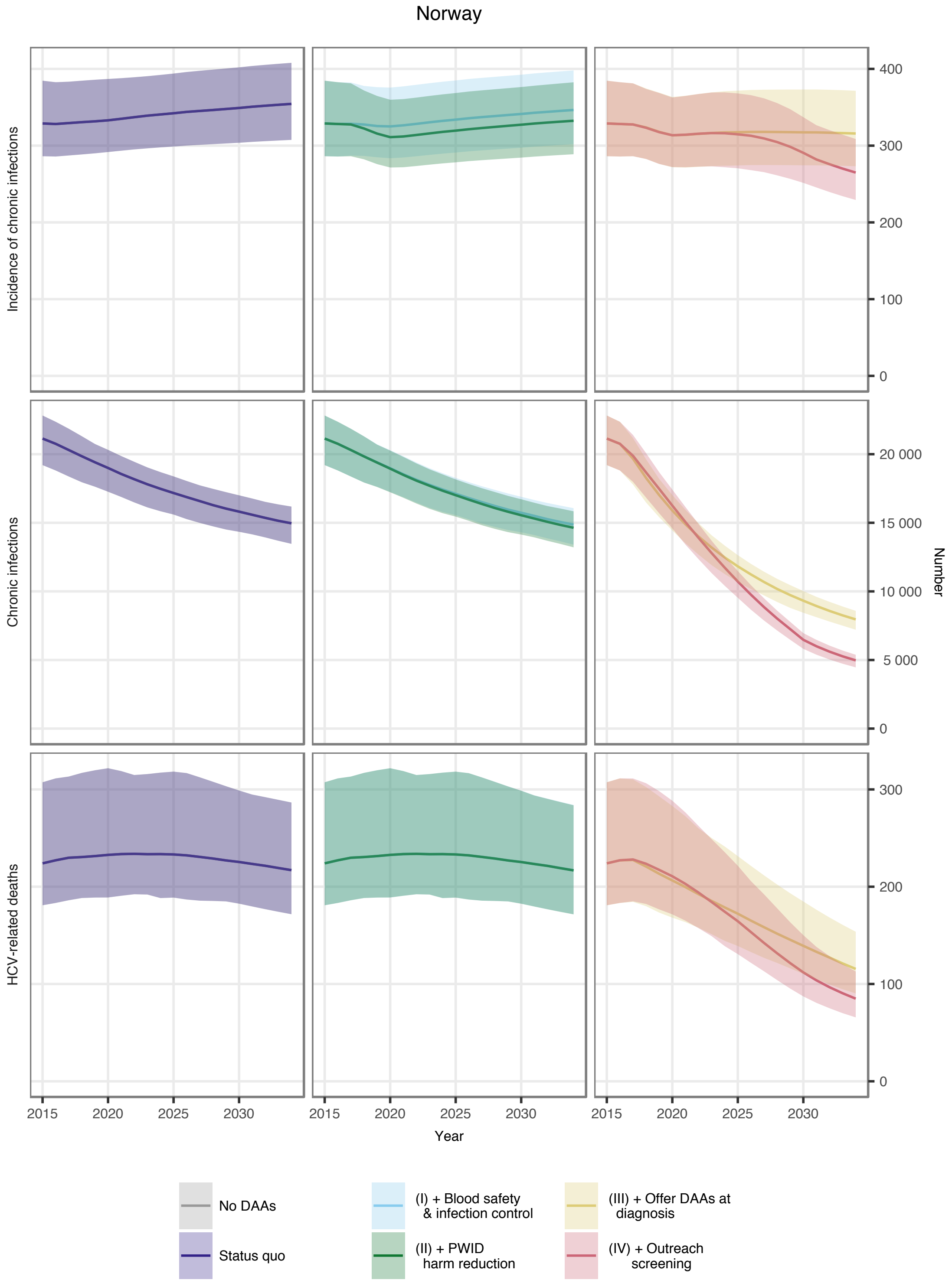

Norway
Incidence of chronic infections
Chronic infections
HCV-related deaths
Number
Year
2015
2020
2025
2030
0
100
200
300
400
5 000
10 000
15 000
20 000
No DAAs
Status quo
(I) + Blood safety & infection control
(II) + PWID harm reduction
(III) + Offer DAAs at diagnosis
(IV) + Outreach screening

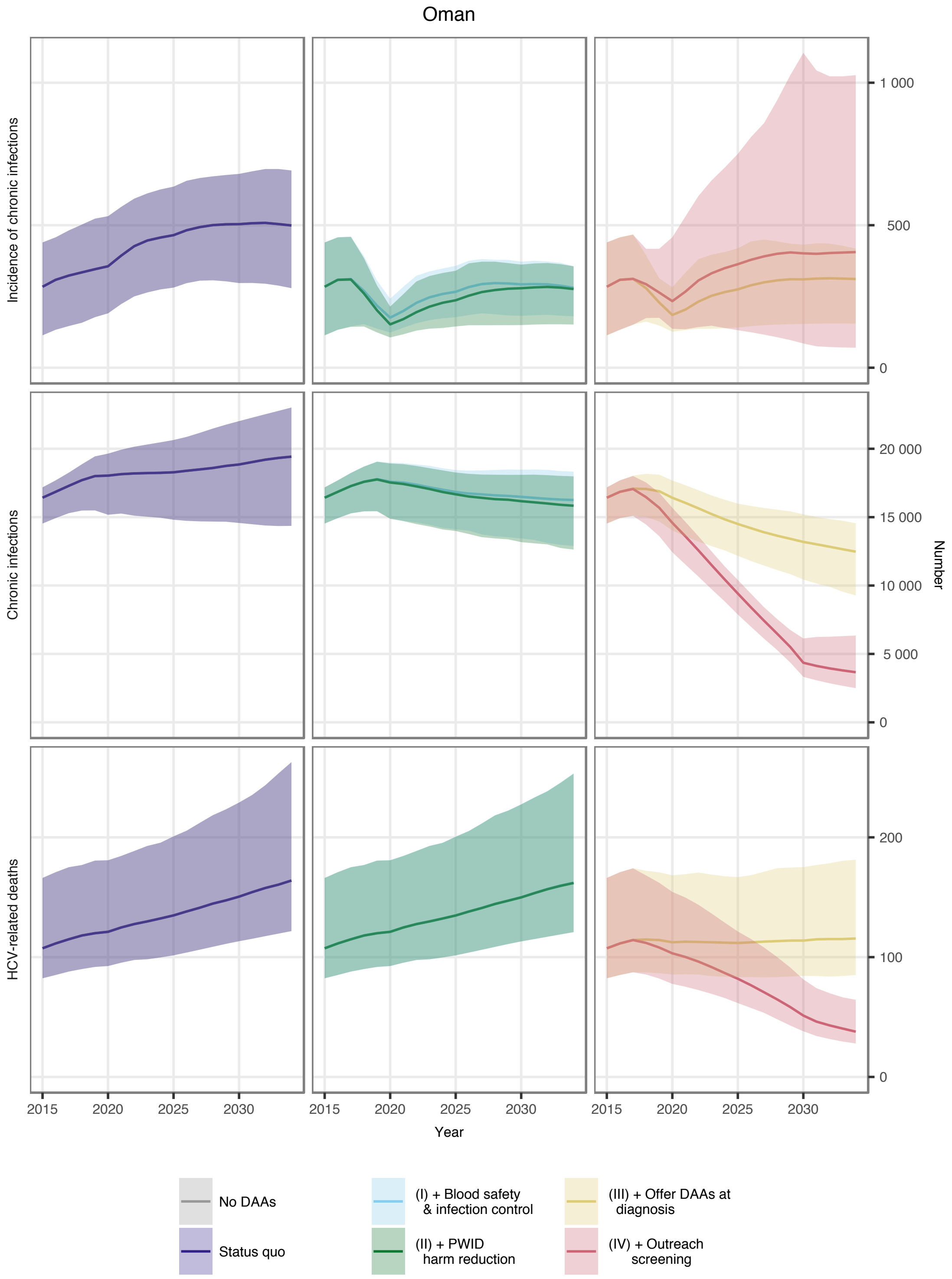

Oman
Incidence of chronic infections
Chronic infections
HCV-related deaths
Number
Year
0
500
1 000
5 000
10 000
15 000
20 000
100
200
2015
2020
2025
2030
No DAAs
Status quo
(I) + Blood safety & infection control
(II) + PWID harm reduction
(III) + Offer DAAs at diagnosis
(IV) + Outreach screening

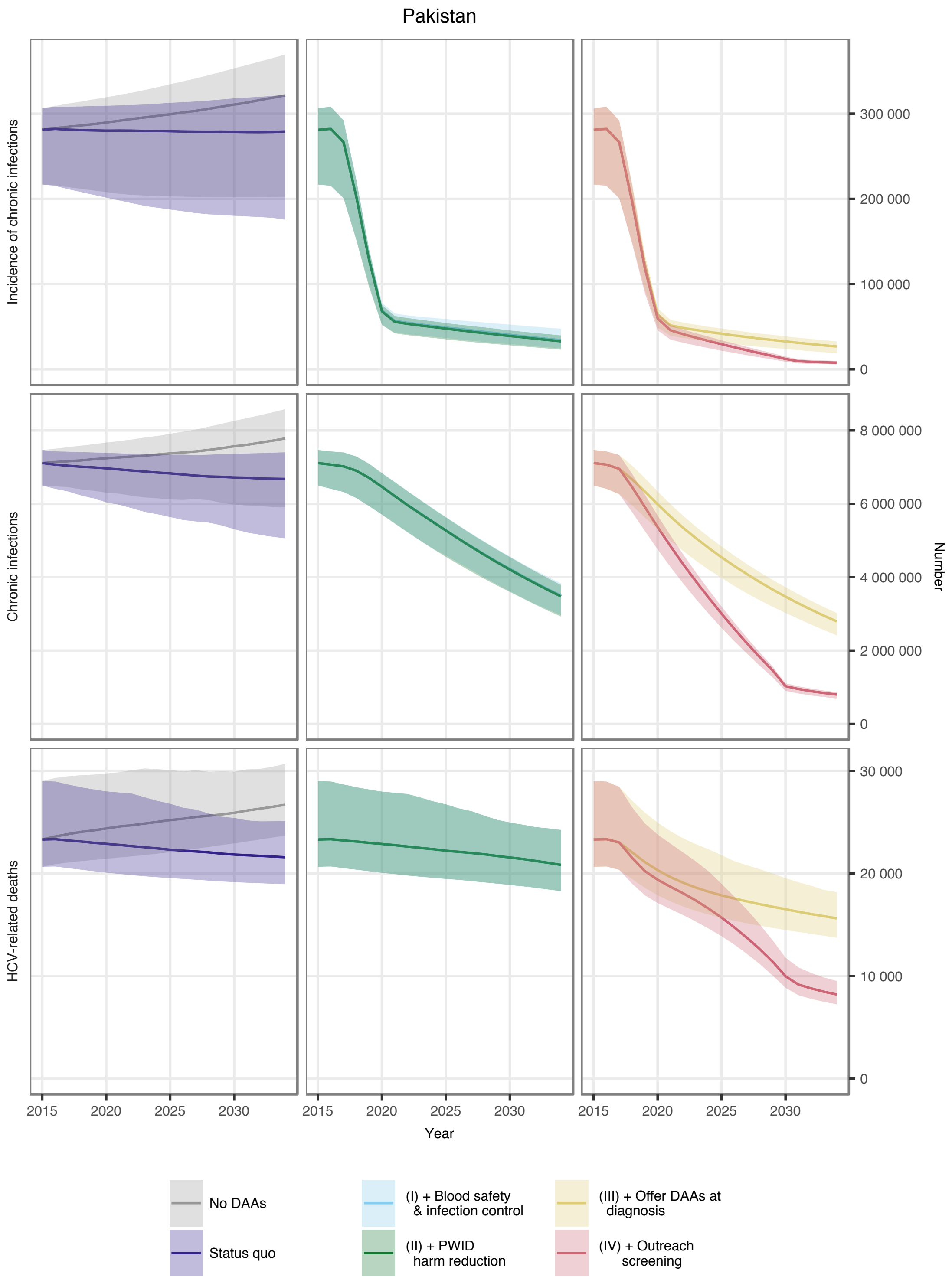
Pakistan
Incidence of chronic infections
Chronic infections
HCV-related deaths
300 000
200 000
100 000
0
8 000 000
6 000 000
4 000 000
2 000 000
0
30 000
20 000
10 000
0
Number
2015
2020
2025
2030
Year
No DAAs
Status quo
(I) + Blood safety & infection control
(II) + PWID harm reduction
(III) + Offer DAAs at diagnosis
(IV) + Outreach screening

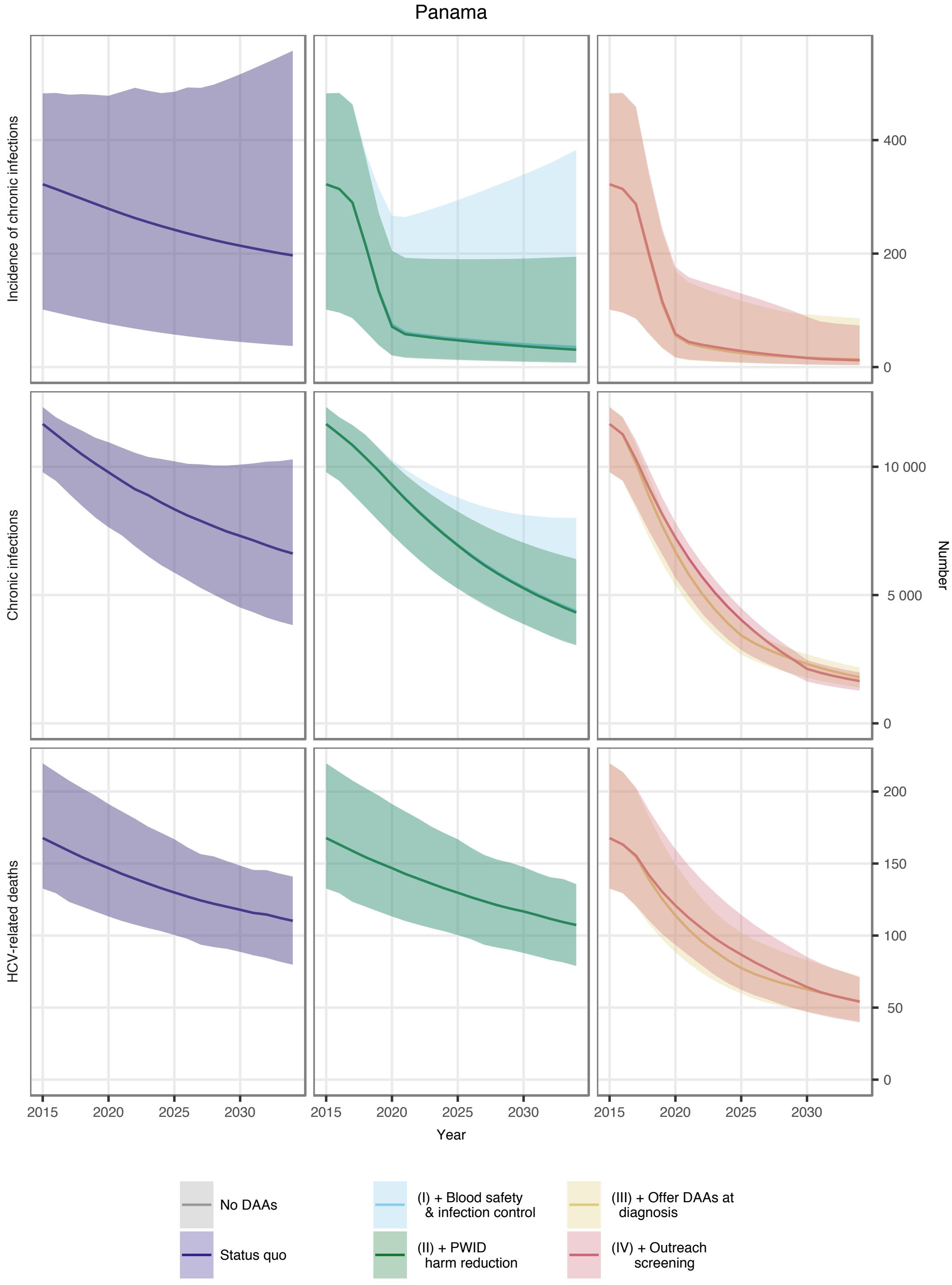
Panama
Incidence of chronic infections
Chronic infections
HCV-related deaths
Number
400
200
0
10 000
5 000
0
200
150
100
50
0
2015
2020
2025
2030
Year
No DAAs
Status quo
(I) + Blood safety & infection control
(II) + PWID harm reduction
(III) + Offer DAAs at diagnosis
(IV) + Outreach screening

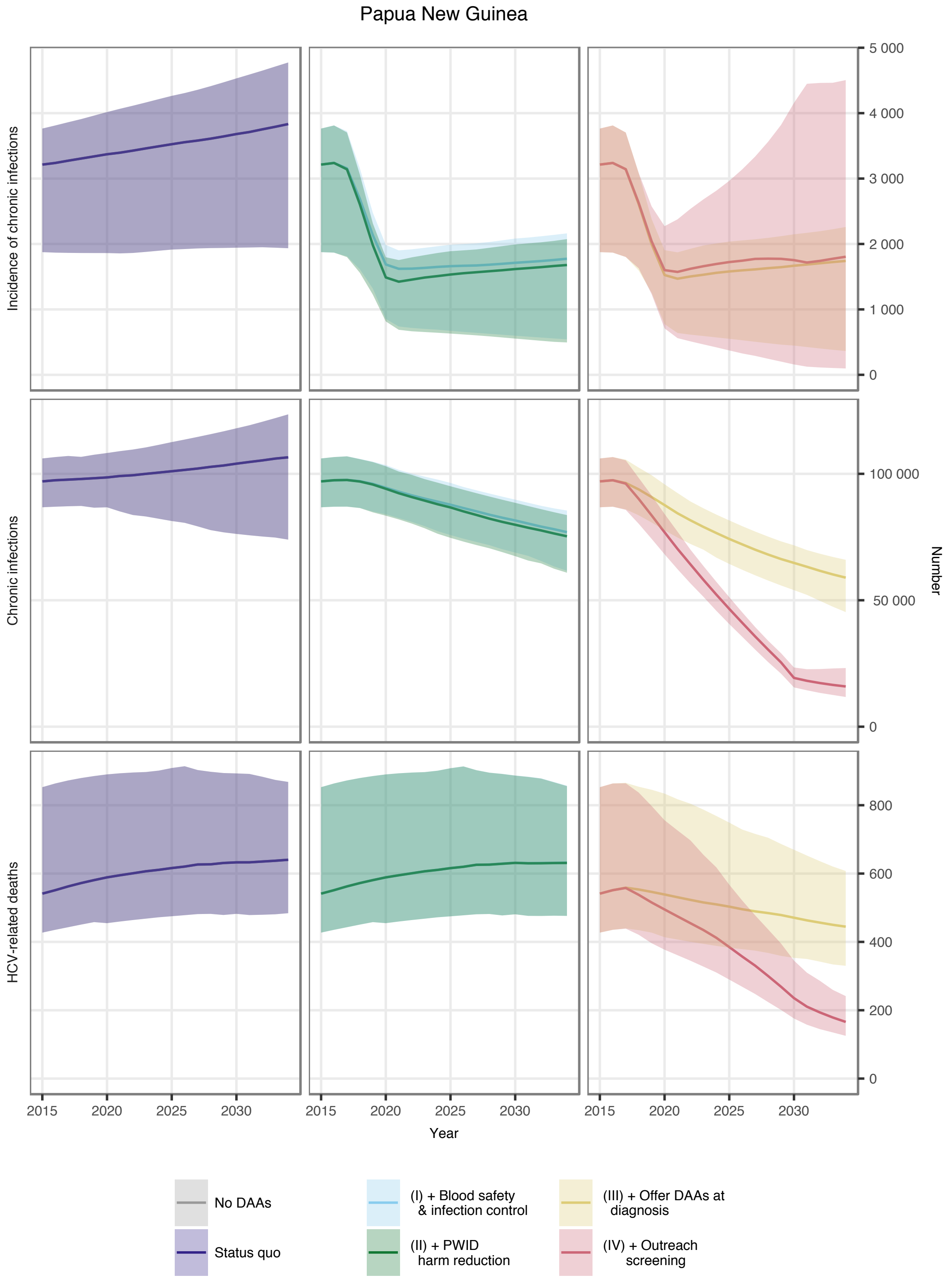

Papua New Guinea
Incidence of chronic infections
Chronic infections
HCV-related deaths
Number
Year
5 000
4 000
3 000
2 000
1 000
0
100 000
50 000
0
800
600
400
200
0
2015
2020
2025
2030
No DAAs
Status quo
(I) + Blood safety & infection control
(II) + PWID harm reduction
(III) + Offer DAAs at diagnosis
(IV) + Outreach screening

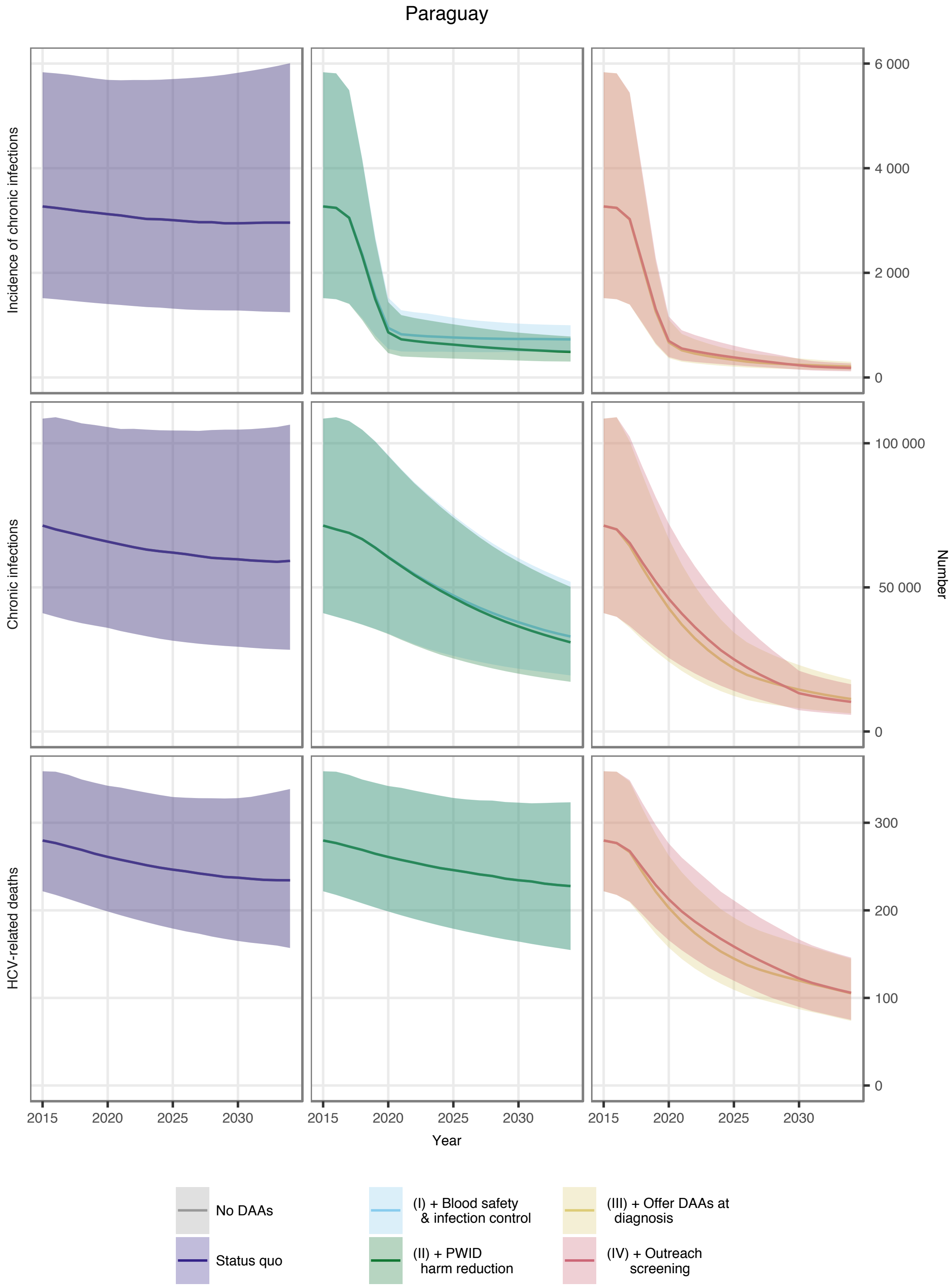
Paraguay
Incidence of chronic infections
Chronic infections
HCV-related deaths
Number
6 000
4 000
2 000
0
100 000
50 000
0
300
200
100
0
2015
2020
2025
2030
Year
No DAAs
Status quo
(I) + Blood safety & infection control
(II) + PWID harm reduction
(III) + Offer DAAs at diagnosis
(IV) + Outreach screening

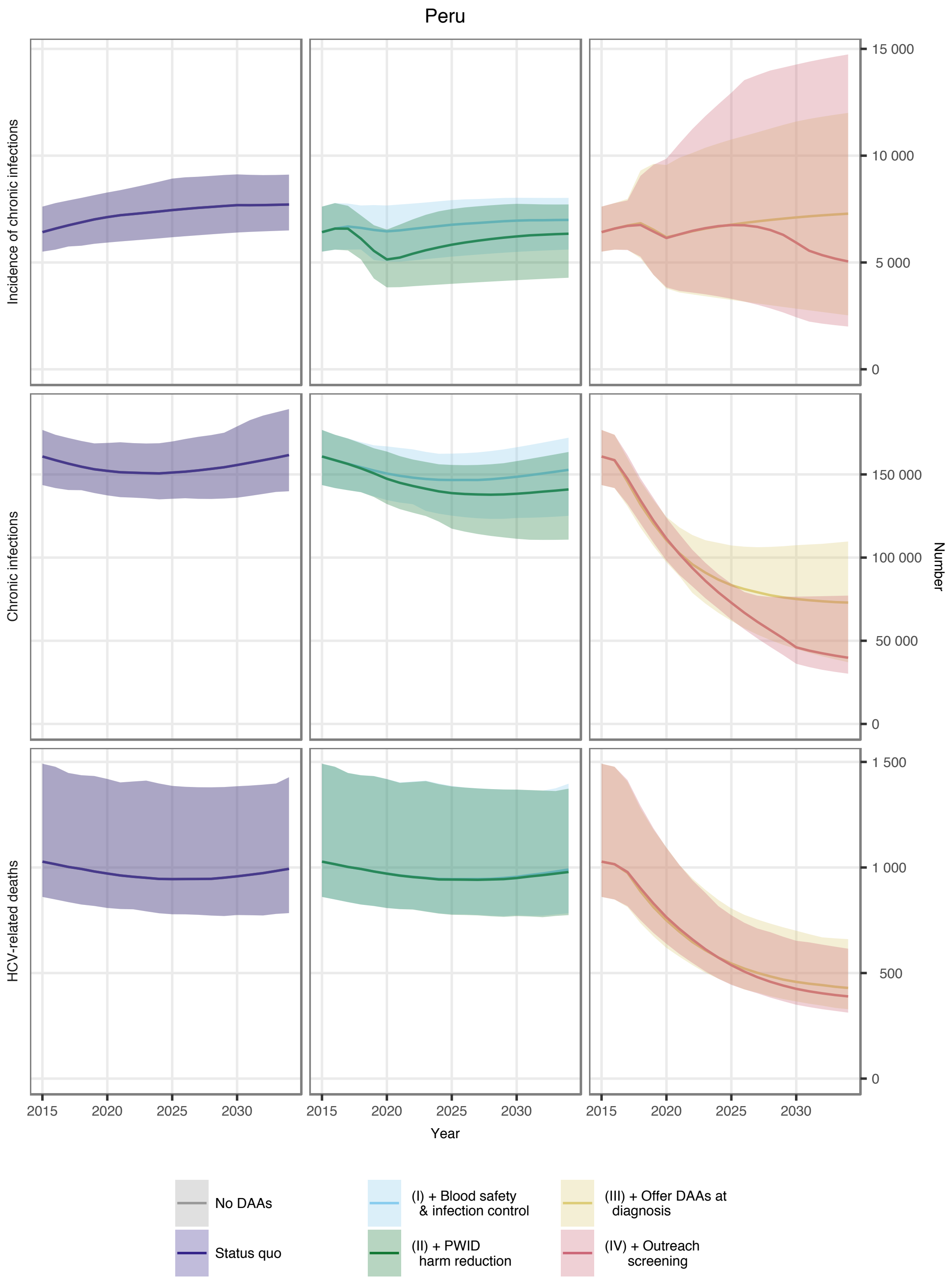
Peru
Incidence of chronic infections
Chronic infections
HCV-related deaths
Number
15 000
10 000
5 000
0
150 000
100 000
50 000
0
1 500
1 000
500
0
2015
2020
2025
2030
Year
No DAAs
Status quo
(I) + Blood safety & infection control
(II) + PWID harm reduction
(III) + Offer DAAs at diagnosis
(IV) + Outreach screening

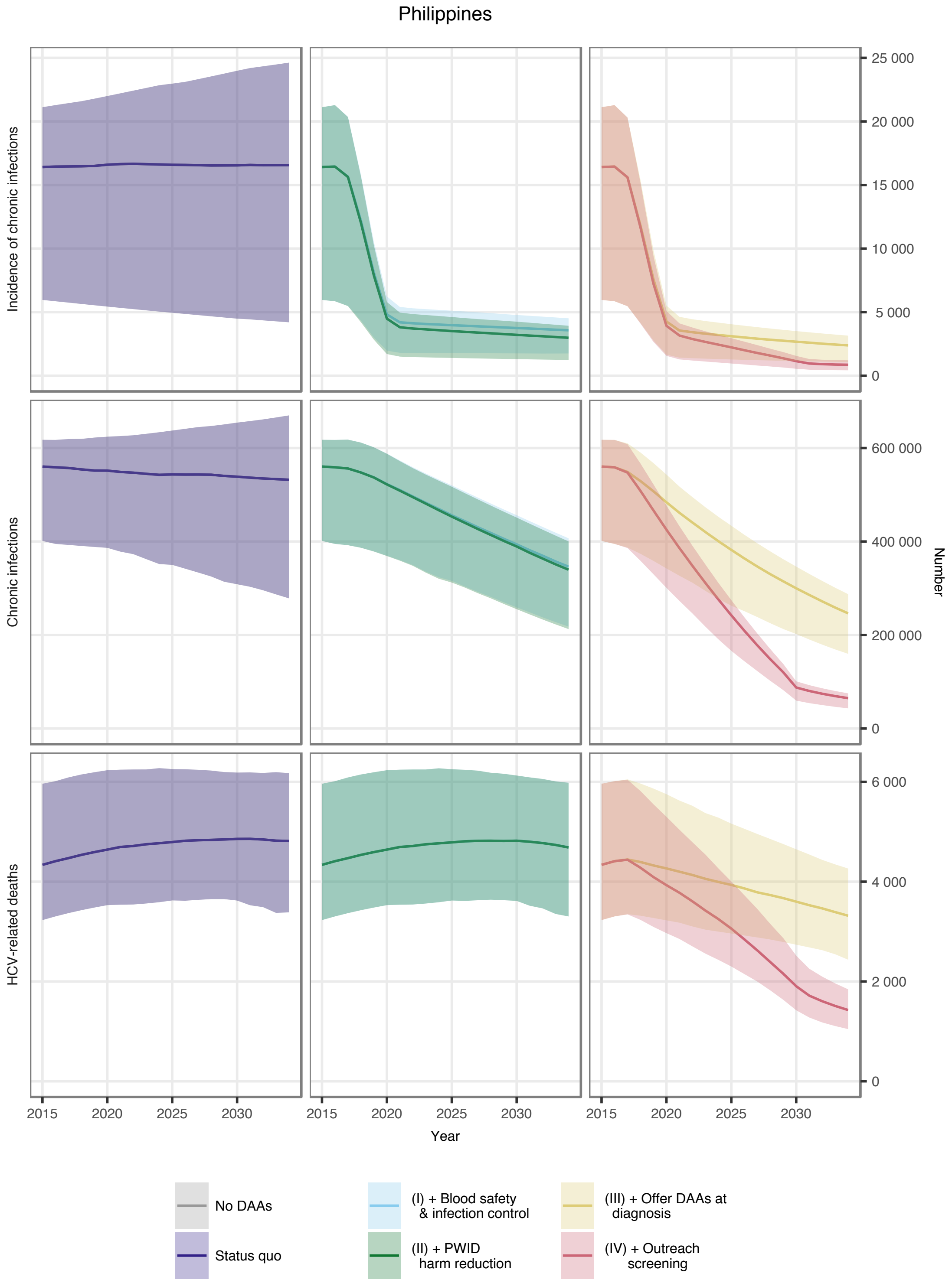

Philippines
Incidence of chronic infections
Chronic infections
HCV-related deaths
25 000
20 000
15 000
10 000
5 000
0
600 000
400 000
200 000
0
6 000
4 000
2 000
0
Number
2015
2020
2025
2030
Year
No DAAs
Status quo
(I) + Blood safety & infection control
(II) + PWID harm reduction
(III) + Offer DAAs at diagnosis
(IV) + Outreach screening

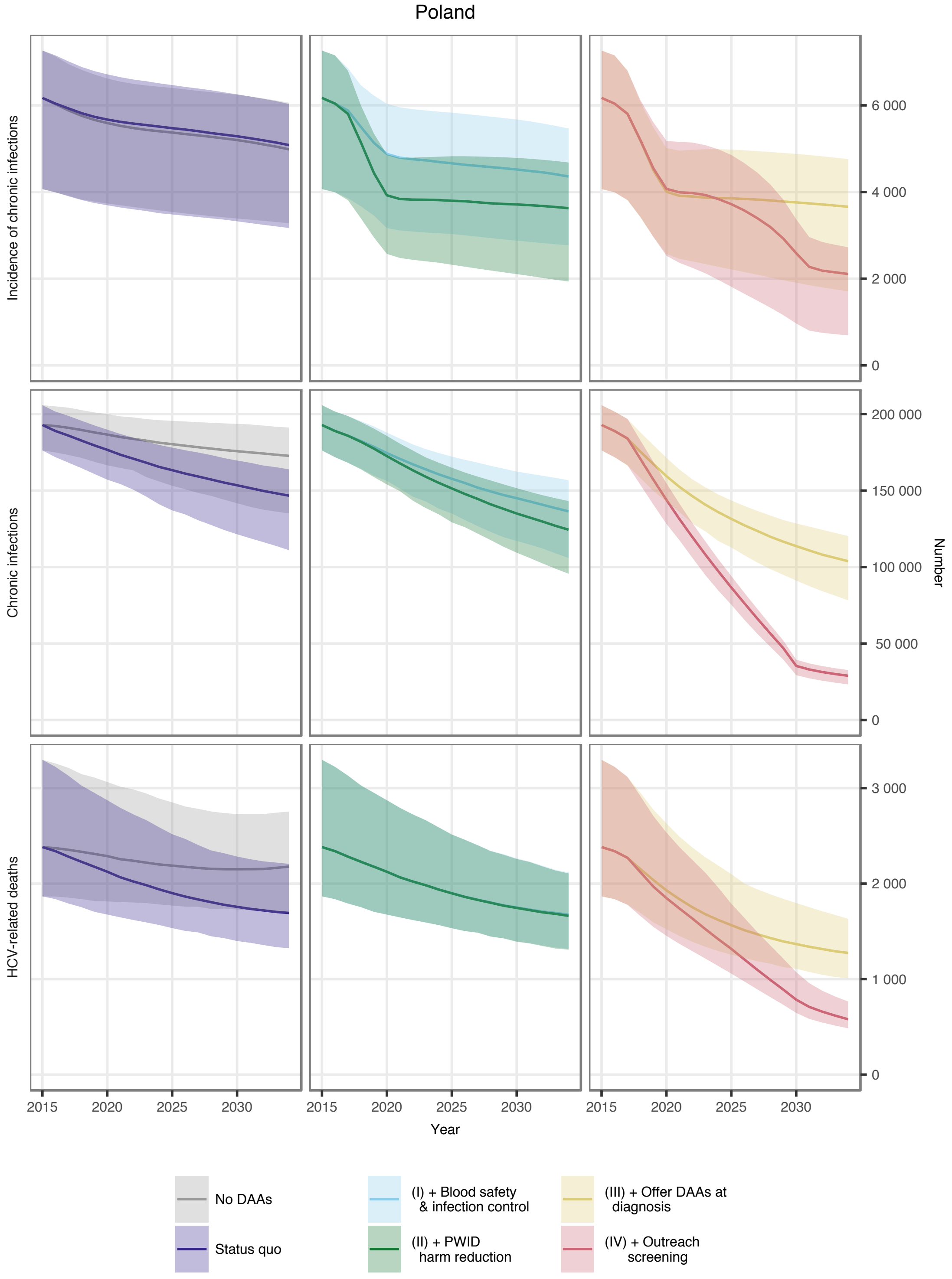

Poland
Incidence of chronic infections
Chronic infections
HCV-related deaths
Number
6 000
4 000
2 000
0
200 000
150 000
100 000
50 000
0
3 000
2 000
1 000
0
2015
2020
2025
2030
Year
No DAAs
Status quo
(I) + Blood safety & infection control
(II) + PWID harm reduction
(III) + Offer DAAs at diagnosis
(IV) + Outreach screening

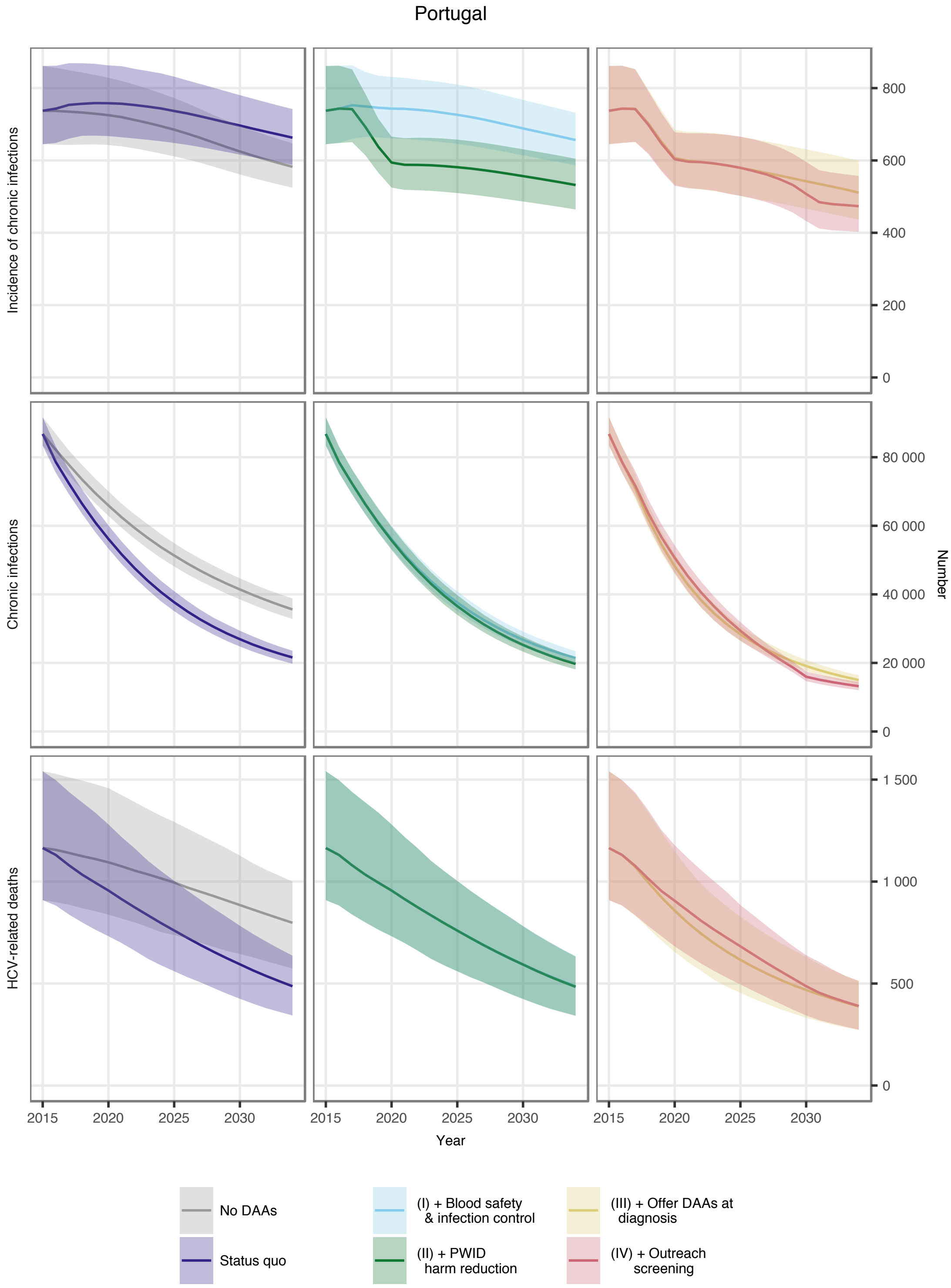
Portugal
Incidence of chronic infections
Chronic infections
HCV-related deaths
Number
0
200
400
600
800
0
20 000
40 000
60 000
80 000
0
500
1 000
1 500
2015
2020
2025
2030
Year
No DAAs
Status quo
(I) + Blood safety & infection control
(II) + PWID harm reduction
(III) + Offer DAAs at diagnosis
(IV) + Outreach screening

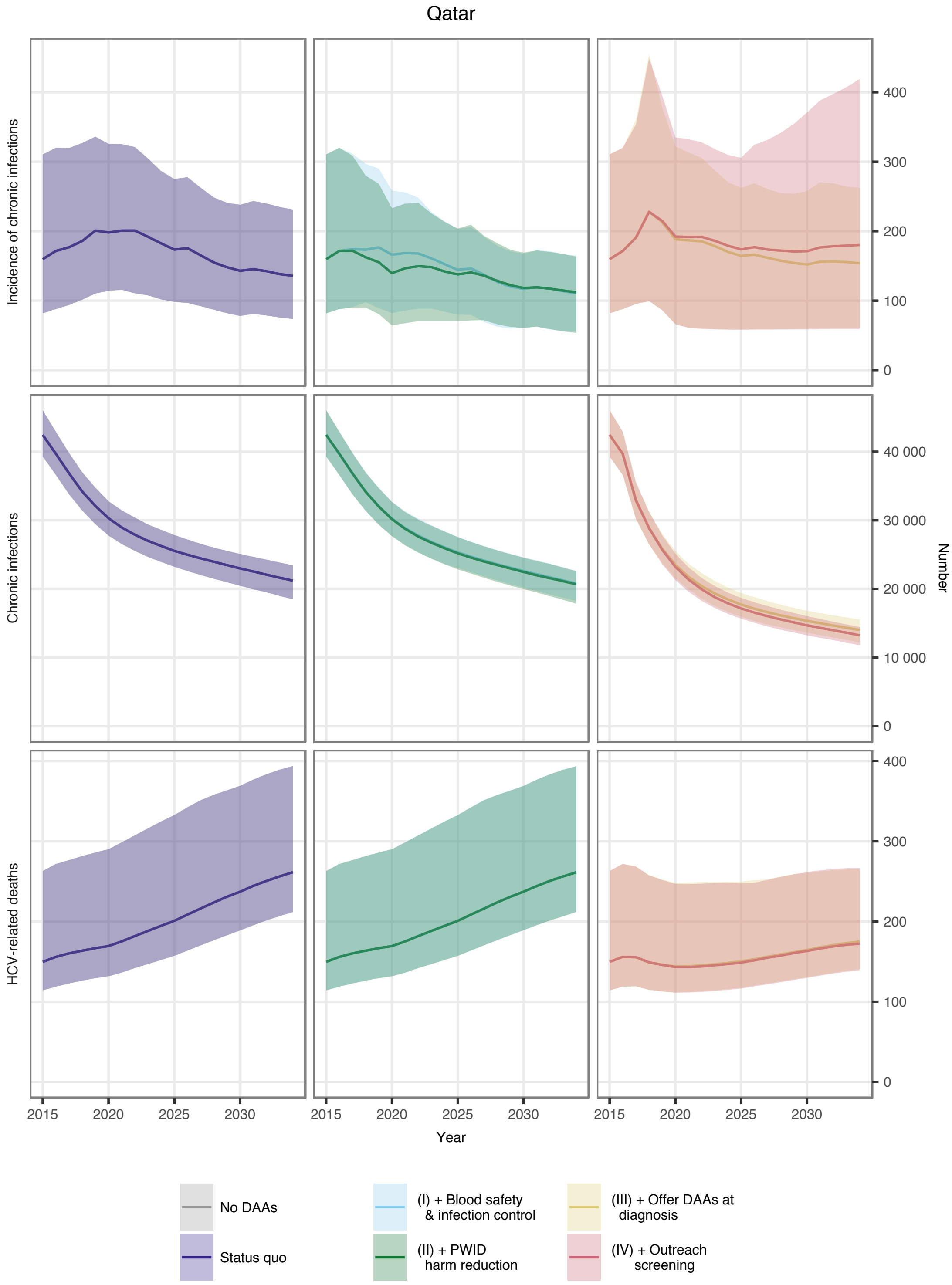

Qatar
Incidence of chronic infections
Chronic infections
HCV-related deaths
Number
Year
2015
2020
2025
2030
0
100
200
300
400
10 000
20 000
30 000
40 000
No DAAs
Status quo
(I) + Blood safety & infection control
(II) + PWID harm reduction
(III) + Offer DAAs at diagnosis
(IV) + Outreach screening

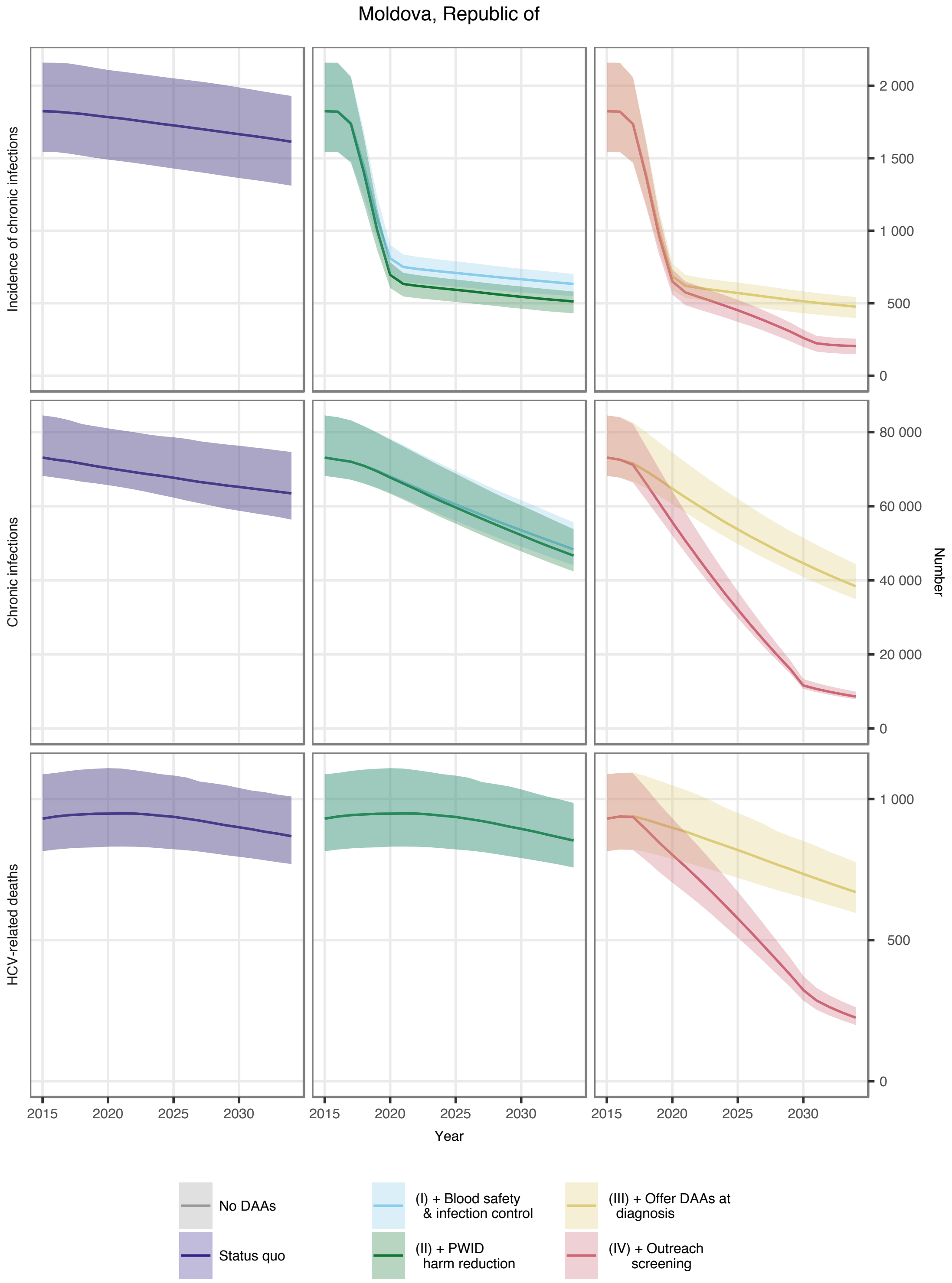
Moldova, Republic of
Incidence of chronic infections
Chronic infections
HCV-related deaths
Number
Year
2015
2020
2025
2030
0
500
1 000
1 500
2 000
20 000
40 000
60 000
80 000
No DAAs
Status quo
(I) + Blood safety & infection control
(II) + PWID harm reduction
(III) + Offer DAAs at diagnosis
(IV) + Outreach screening

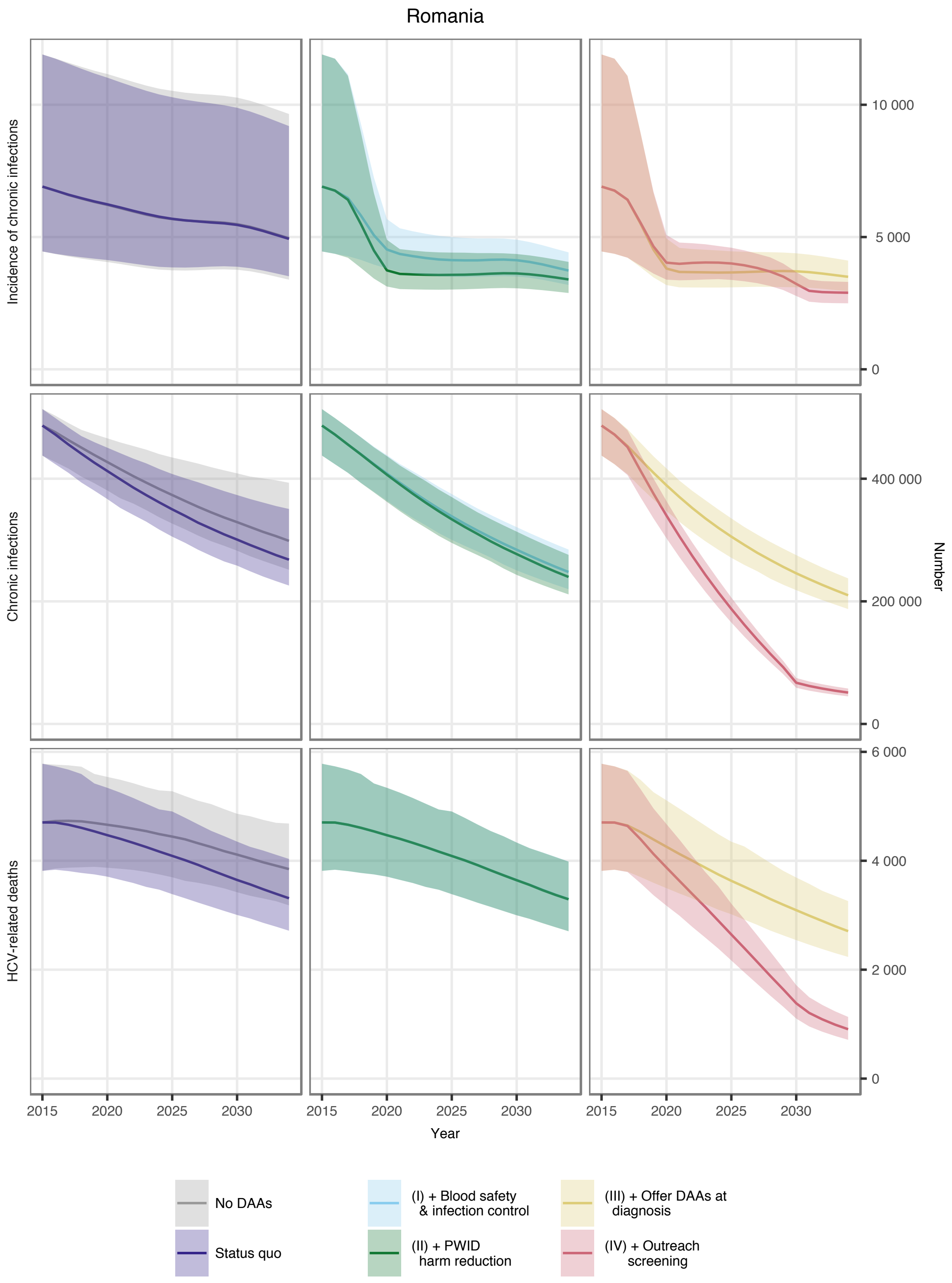
Romania
Incidence of chronic infections
Chronic infections
HCV-related deaths
10 000
5 000
0
400 000
200 000
0
6 000
4 000
2 000
0
Number
2015
2020
2025
2030
Year
No DAAs
Status quo
(I) + Blood safety & infection control
(II) + PWID harm reduction
(III) + Offer DAAs at diagnosis
(IV) + Outreach screening

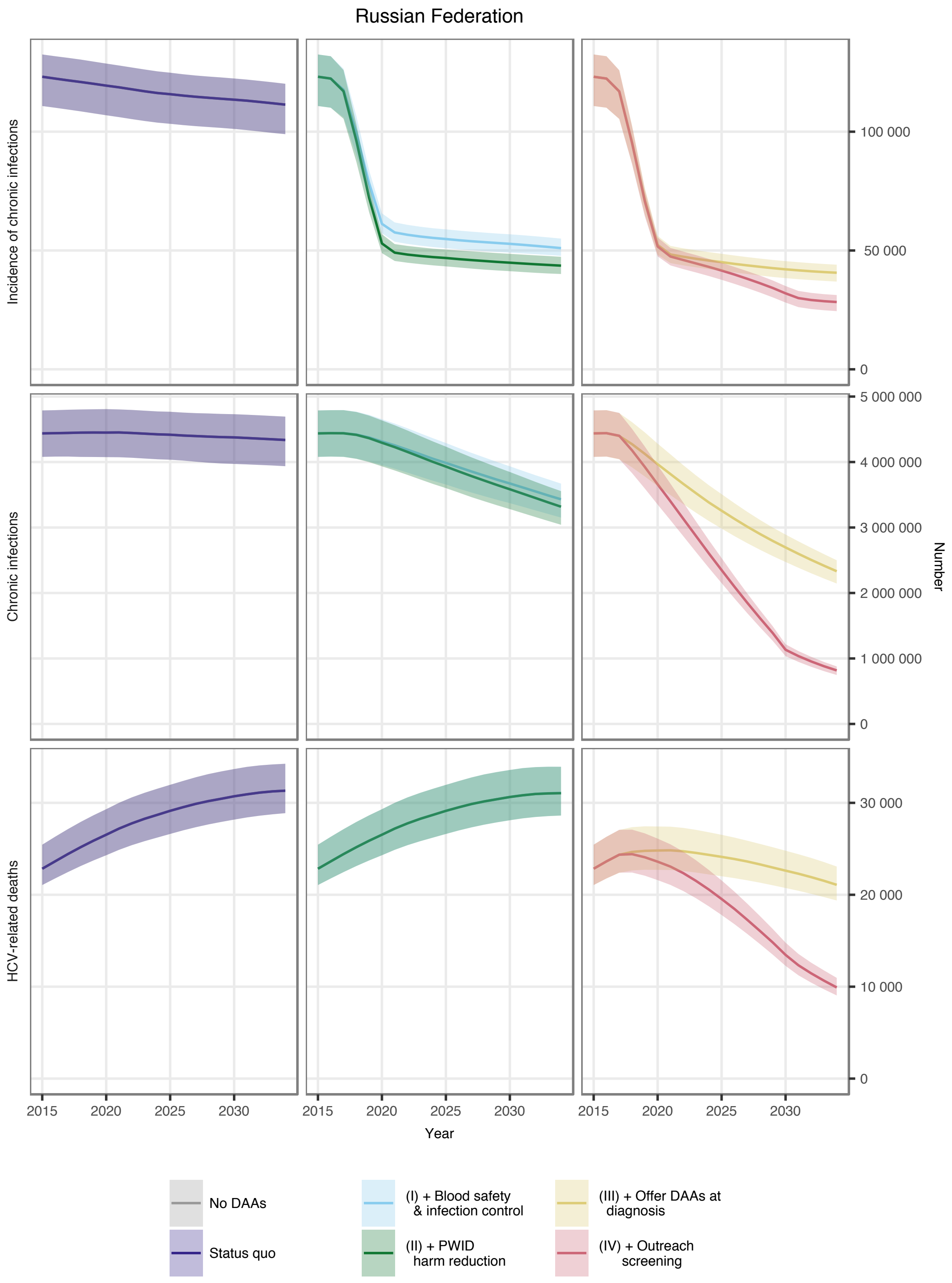

Russian Federation
Incidence of chronic infections
Chronic infections
HCV-related deaths
Number
100 000
50 000
0
5 000 000
4 000 000
3 000 000
2 000 000
1 000 000
0
30 000
20 000
10 000
0
2015
2020
2025
2030
Year
No DAAs
Status quo
(I) + Blood safety & infection control
(II) + PWID harm reduction
(III) + Offer DAAs at diagnosis
(IV) + Outreach screening

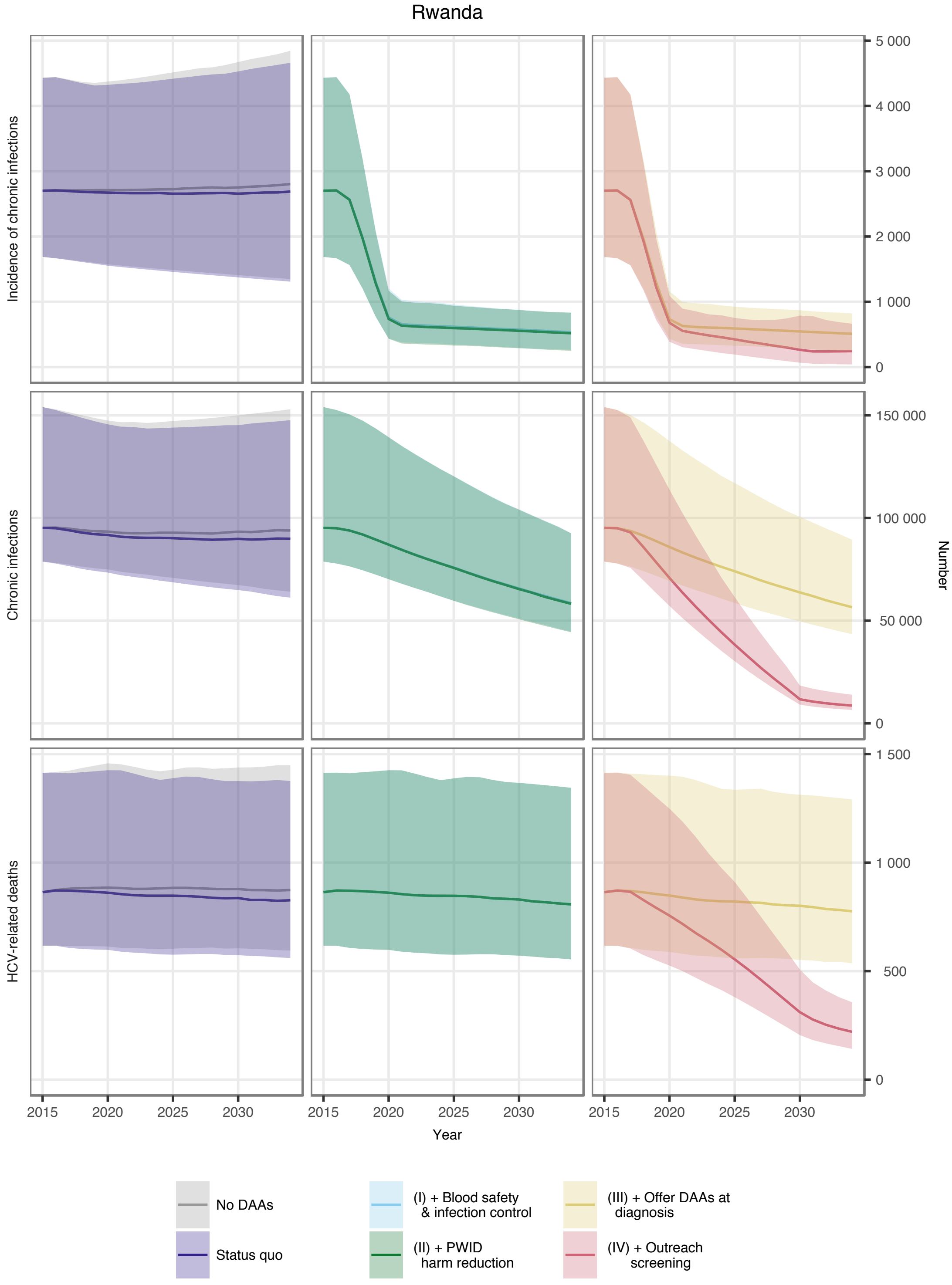
Rwanda
Incidence of chronic infections
Chronic infections
HCV-related deaths
Number
Year
5 000
4 000
3 000
2 000
1 000
0
150 000
100 000
50 000
0
1 500
1 000
500
0
2015
2020
2025
2030
No DAAs
Status quo
(I) + Blood safety & infection control
(II) + PWID harm reduction
(III) + Offer DAAs at diagnosis
(IV) + Outreach screening

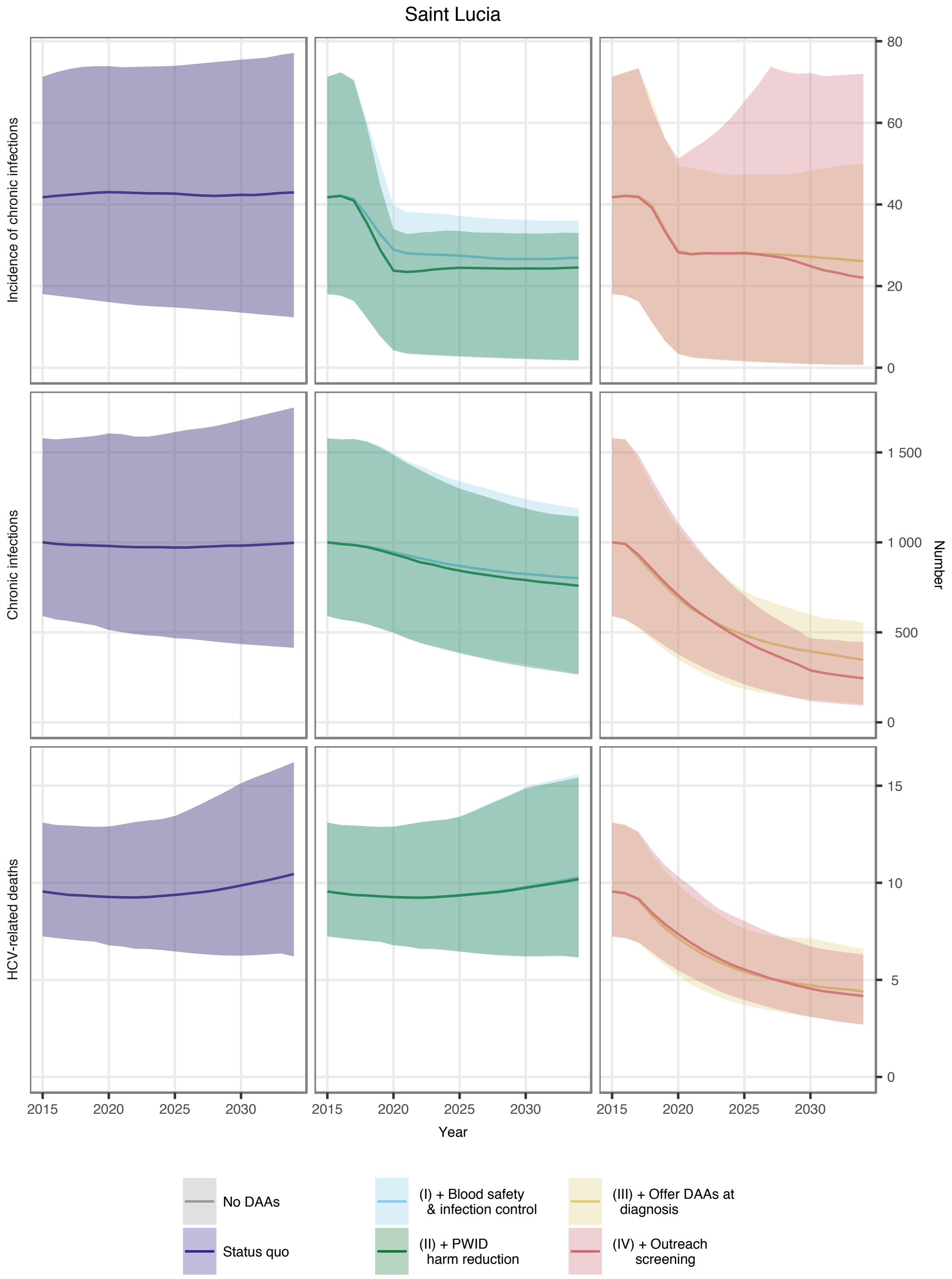
Saint Lucia
Incidence of chronic infections
Chronic infections
HCV-related deaths
Number
Year
80
60
40
20
0
1 500
1 000
500
0
15
10
5
0
2015
2020
2025
2030
No DAAs
Status quo
(I) + Blood safety & infection control
(II) + PWID harm reduction
(III) + Offer DAAs at diagnosis
(IV) + Outreach screening

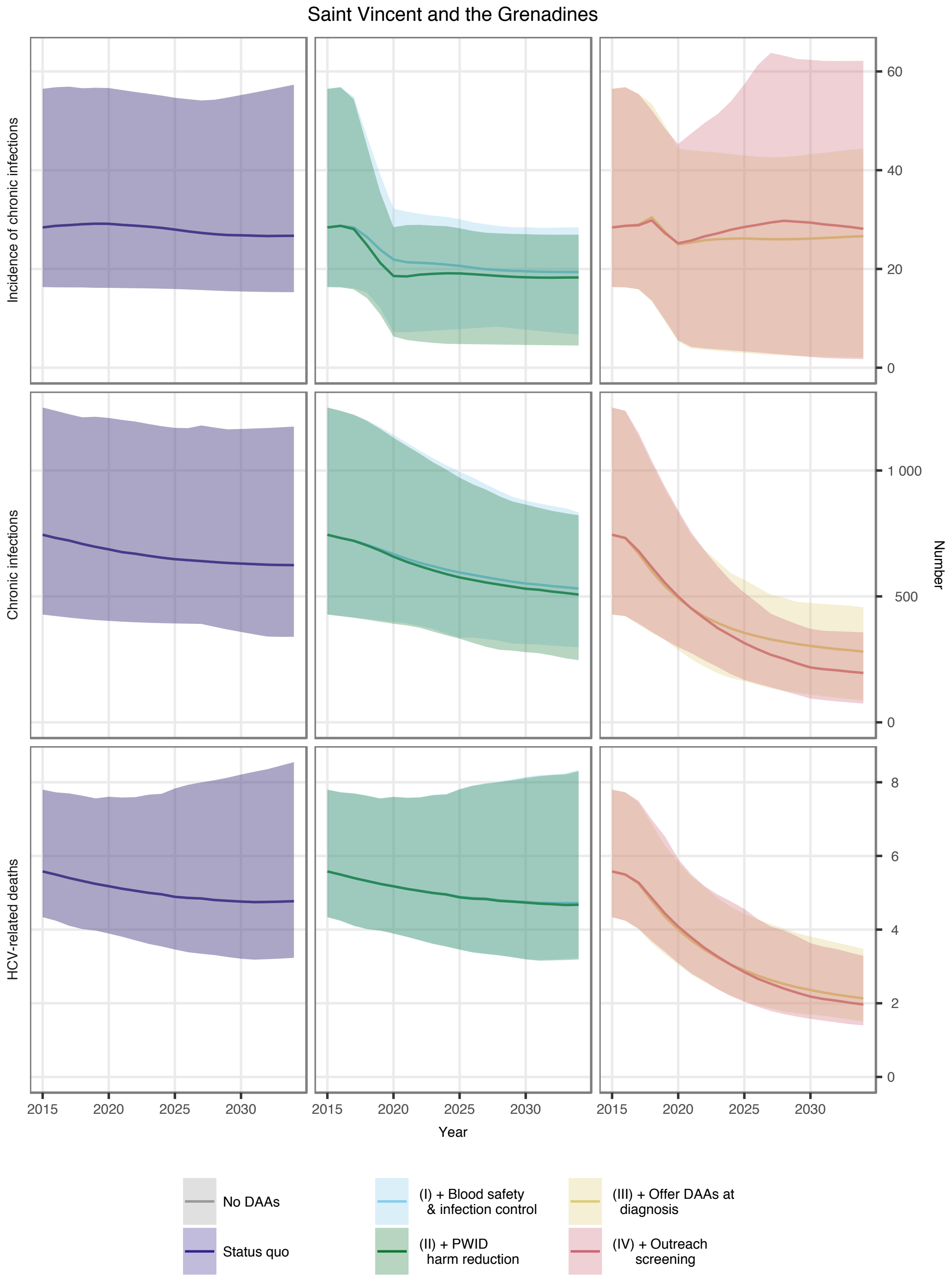

Saint Vincent and the Grenadines
Incidence of chronic infections
Chronic infections
HCV-related deaths
Number
Year
0
20
40
60
0
500
1 000
0
2
4
6
8
2015
2020
2025
2030
No DAAs
Status quo
(I) + Blood safety & infection control
(II) + PWID harm reduction
(III) + Offer DAAs at diagnosis
(IV) + Outreach screening

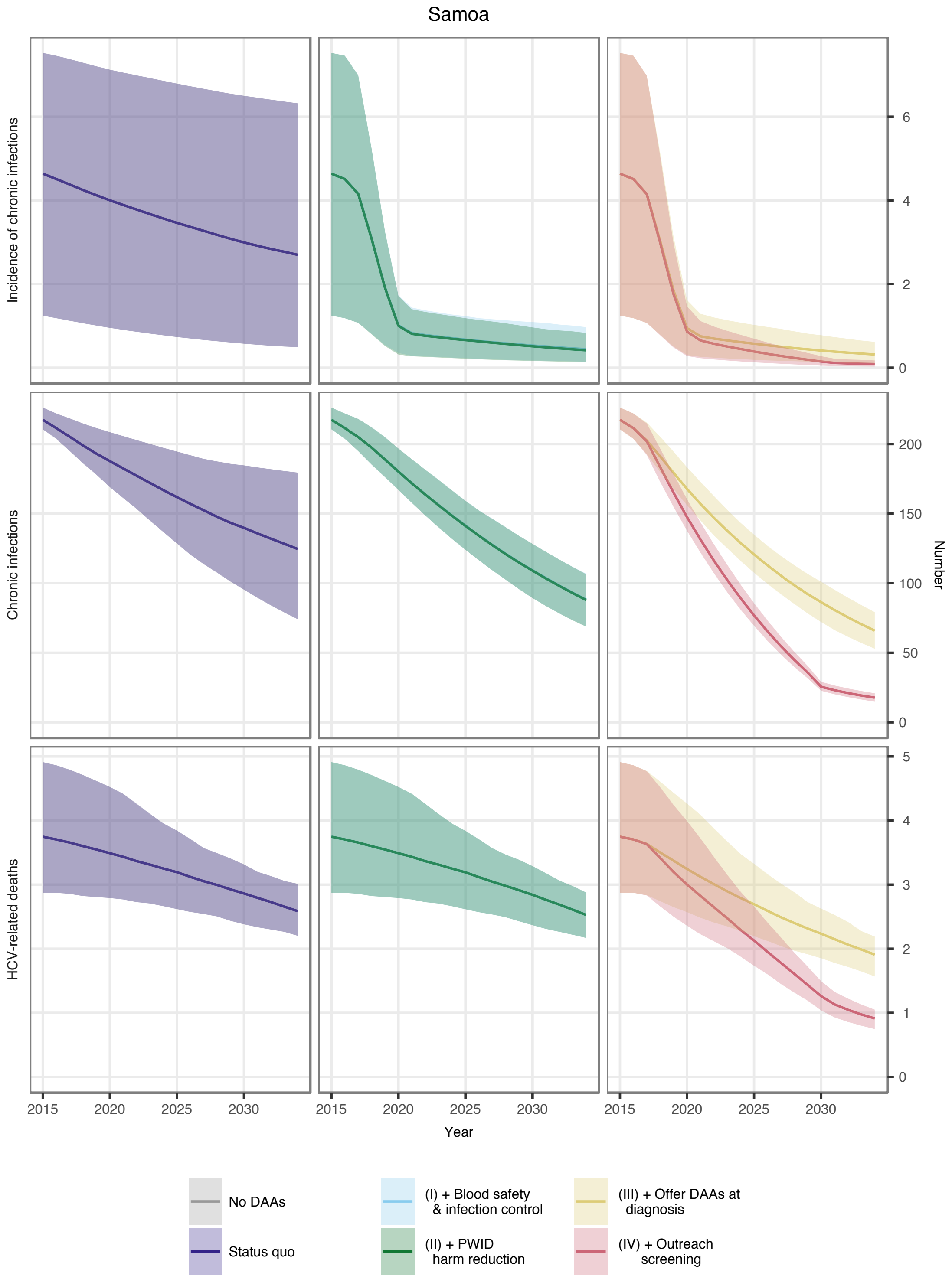
Samoa
Incidence of chronic infections
Chronic infections
HCV-related deaths
Number
Year
2015
2020
2025
2030
0
2
4
6
0
50
100
150
200
0
1
2
3
4
5
No DAAs
Status quo
(I) + Blood safety & infection control
(II) + PWID harm reduction
(III) + Offer DAAs at diagnosis
(IV) + Outreach screening

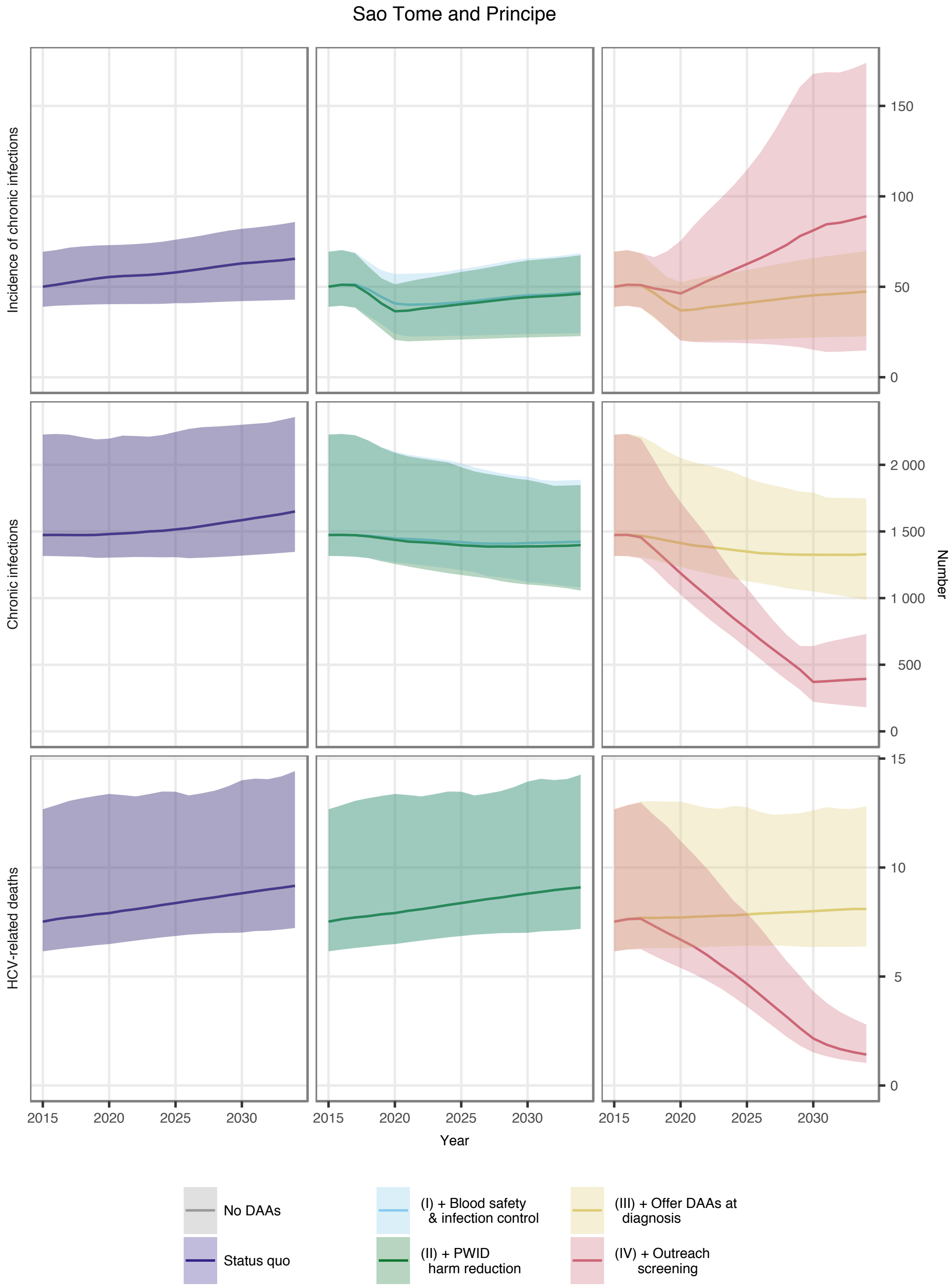
Sao Tome and Principe
Incidence of chronic infections
Chronic infections
HCV-related deaths
Number
Year
150
100
50
0
2 000
1 500
1 000
500
0
15
10
5
0
2015
2020
2025
2030
No DAAs
Status quo
(I) + Blood safety & infection control
(II) + PWID harm reduction
(III) + Offer DAAs at diagnosis
(IV) + Outreach screening

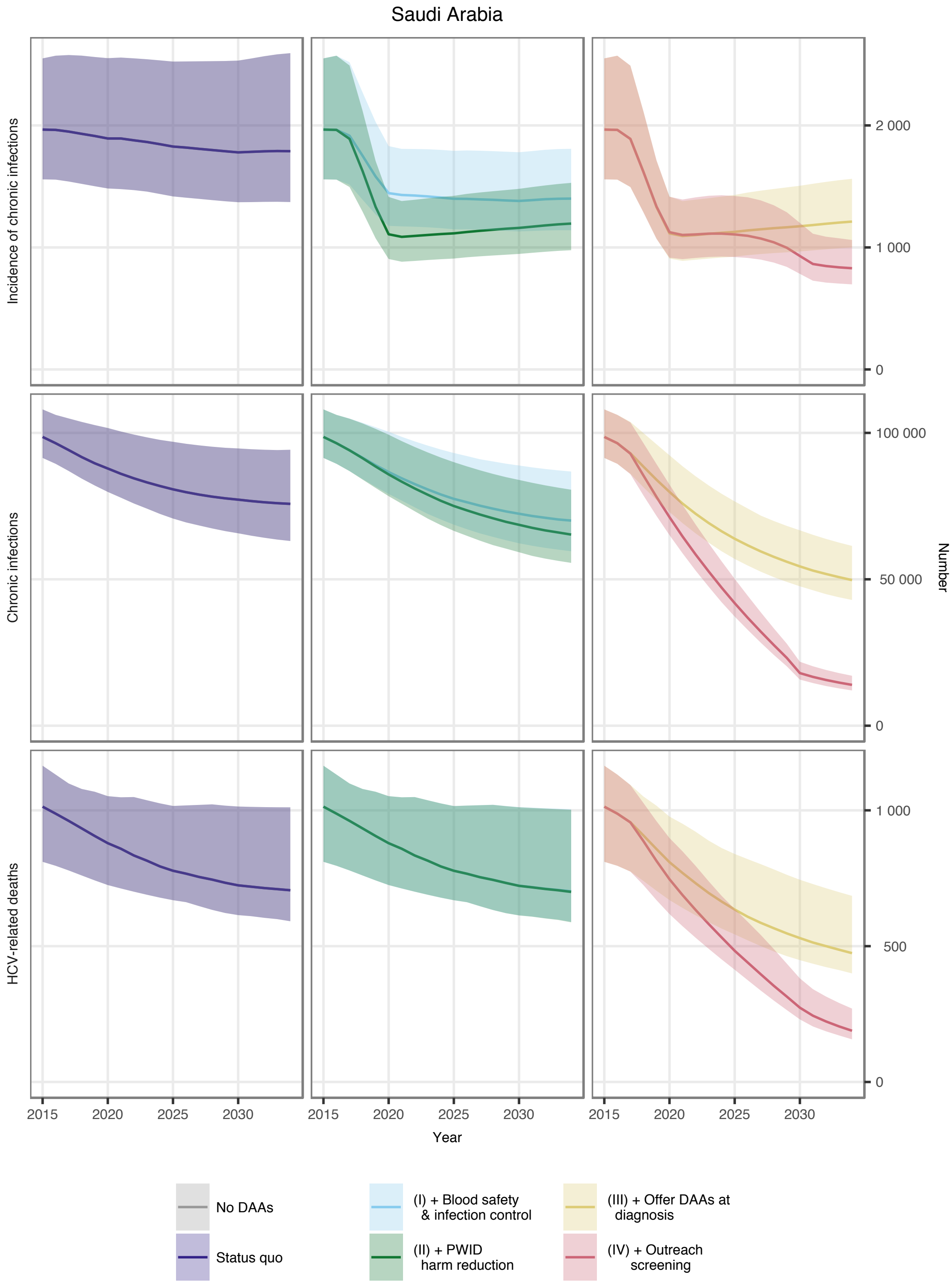
Saudi Arabia
Incidence of chronic infections
Chronic infections
HCV-related deaths
Number
2 000
1 000
0
100 000
50 000
0
1 000
500
0
2015
2020
2025
2030
Year
No DAAs
Status quo
(I) + Blood safety & infection control
(II) + PWID harm reduction
(III) + Offer DAAs at diagnosis
(IV) + Outreach screening

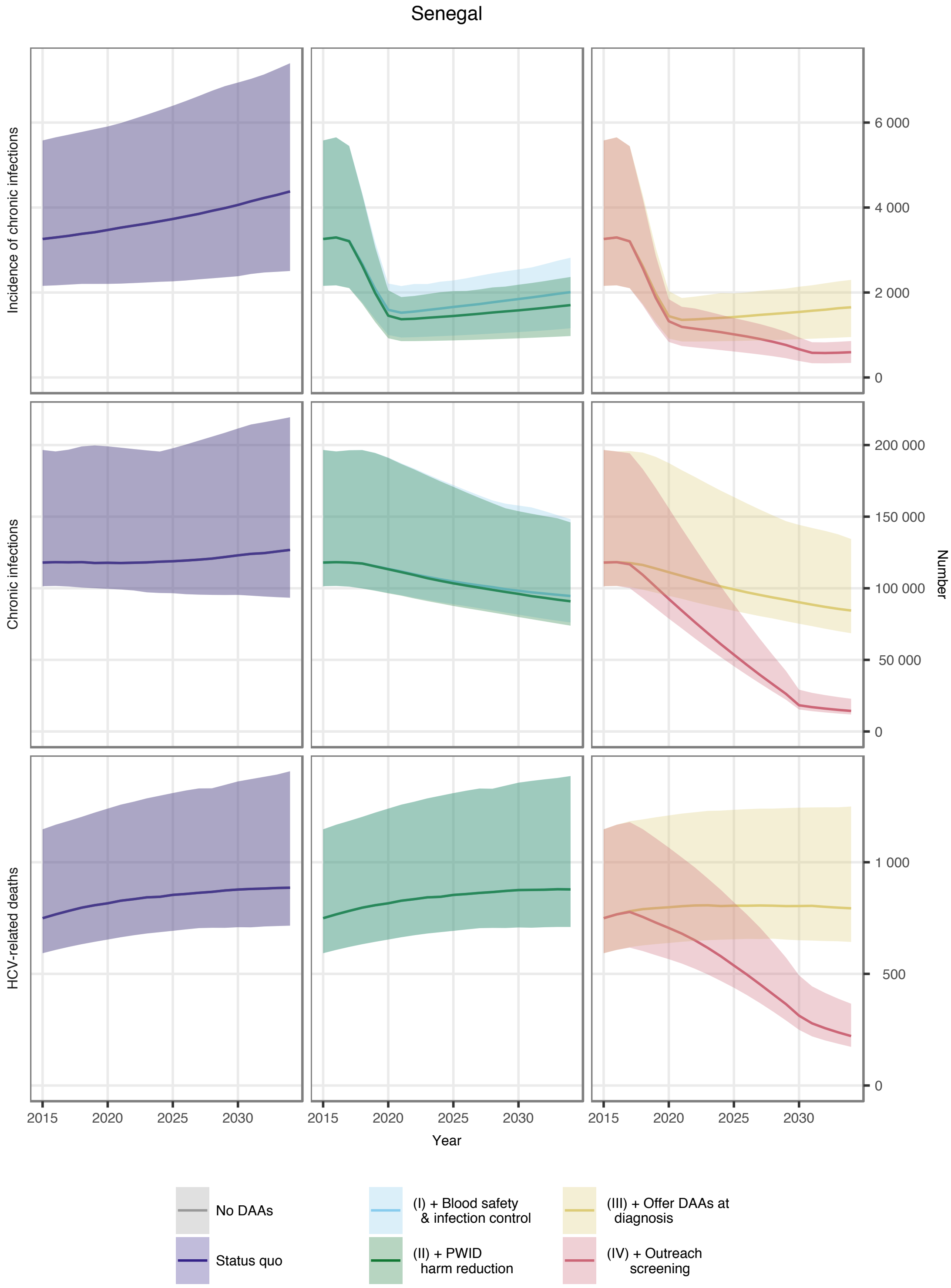

Senegal
Incidence of chronic infections
Chronic infections
HCV-related deaths
Number
Year
2015
2020
2025
2030
6 000
4 000
2 000
0
200 000
150 000
100 000
50 000
0
1 000
500
0
No DAAs
Status quo
(I) + Blood safety & infection control
(II) + PWID harm reduction
(III) + Offer DAAs at diagnosis
(IV) + Outreach screening

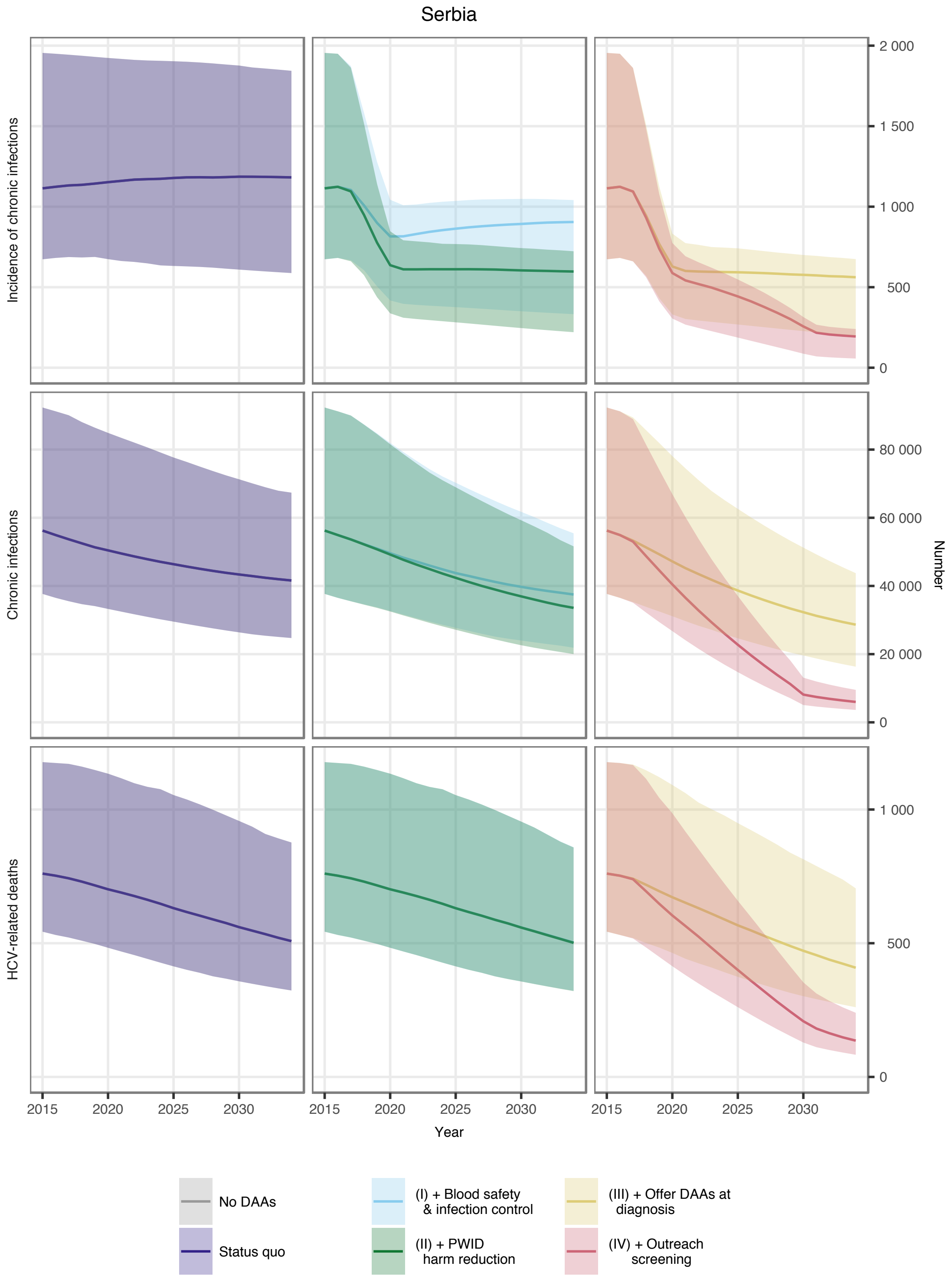
Serbia
Incidence of chronic infections
Chronic infections
HCV-related deaths
Number
2 000
1 500
1 000
500
0
80 000
60 000
40 000
20 000
0
1 000
500
0
2015
2020
2025
2030
Year
No DAAs
Status quo
(I) + Blood safety & infection control
(II) + PWID harm reduction
(III) + Offer DAAs at diagnosis
(IV) + Outreach screening

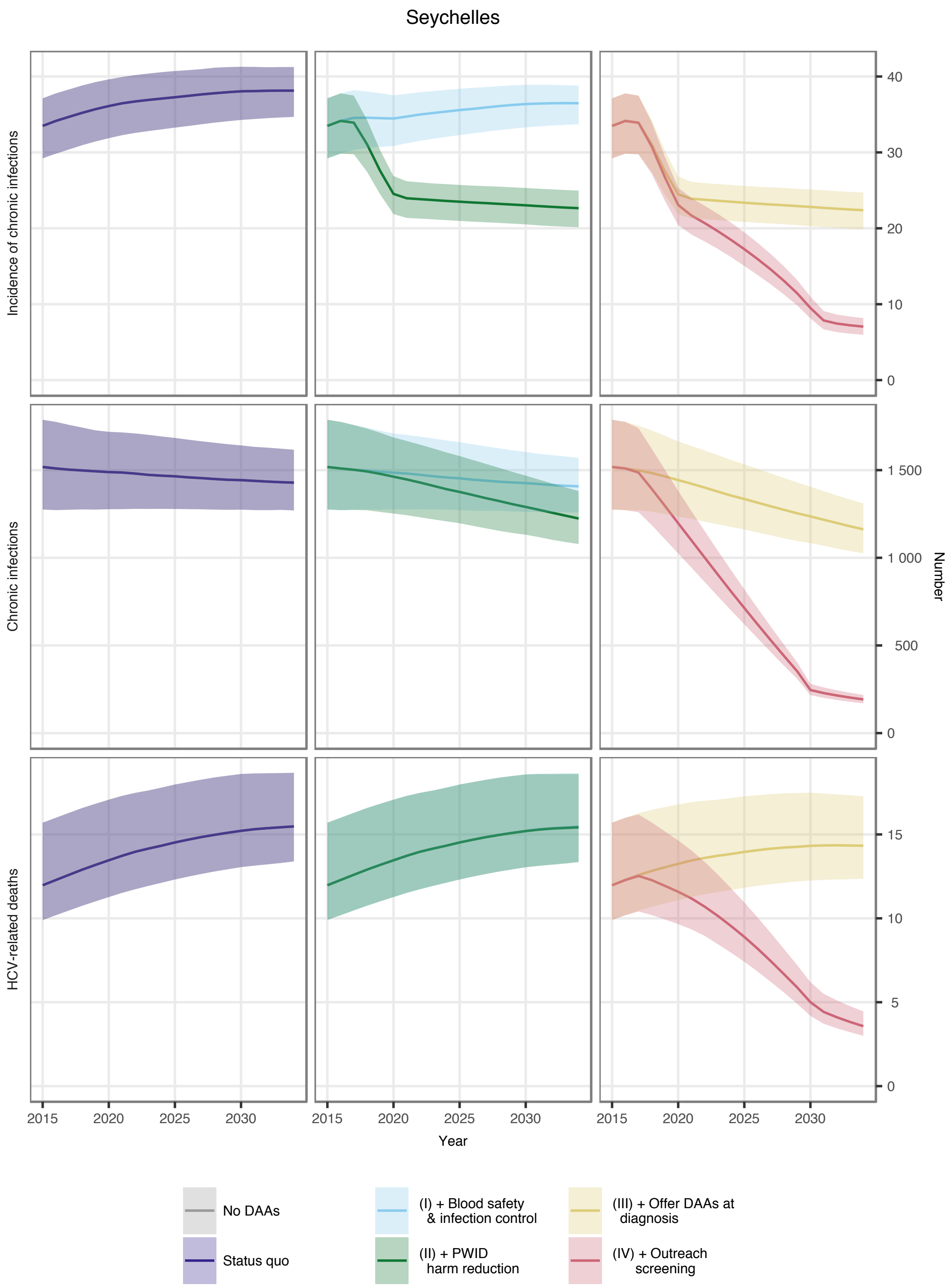
Seychelles
Incidence of chronic infections
Chronic infections
HCV-related deaths
Number
Year
2015
2020
2025
2030
0
10
20
30
40
500
1 000
1 500
5
15
No DAAs
Status quo
(I) + Blood safety & infection control
(II) + PWID harm reduction
(III) + Offer DAAs at diagnosis
(IV) + Outreach screening

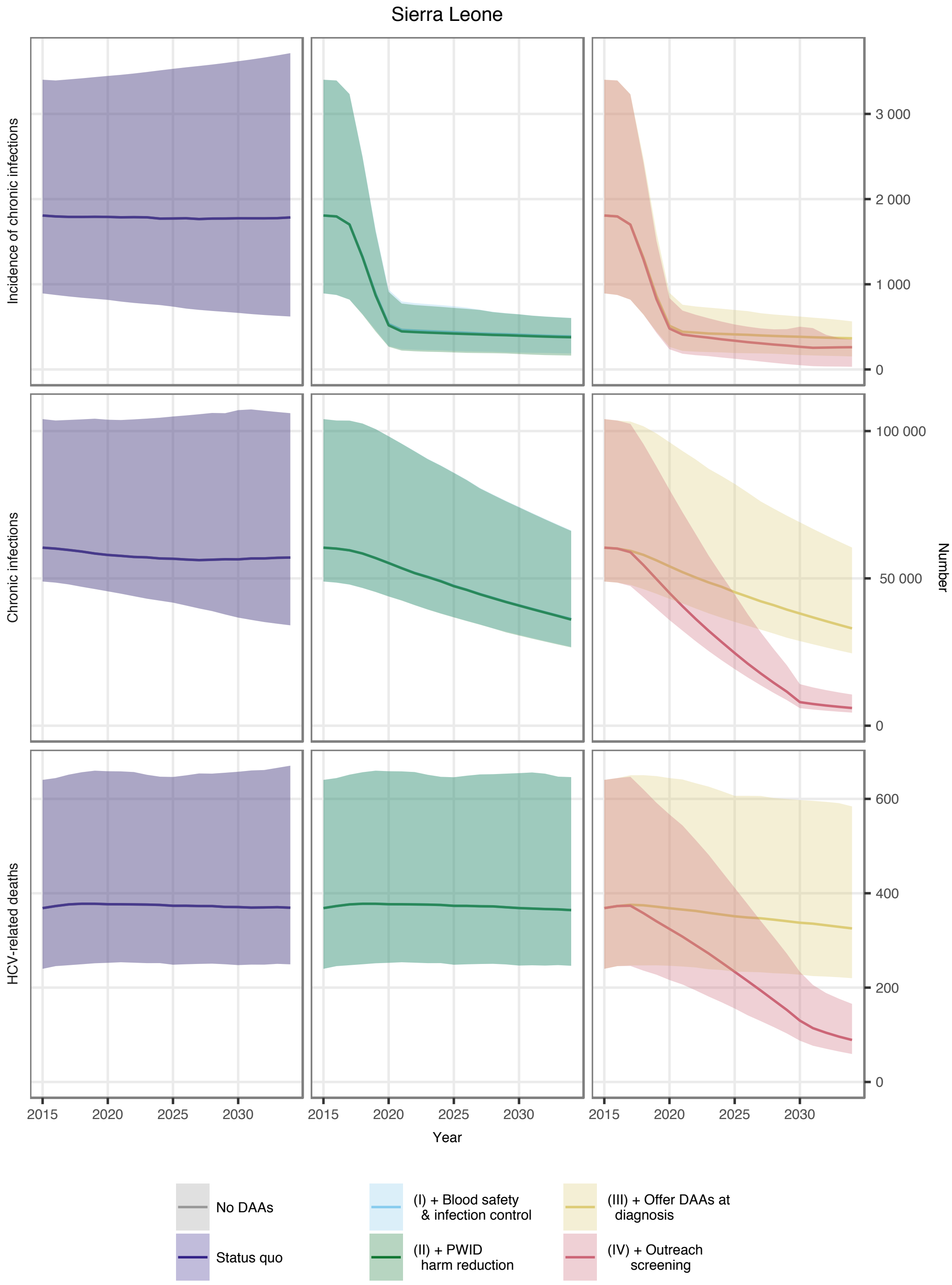

Sierra Leone
Incidence of chronic infections
Chronic infections
HCV-related deaths
Number
3 000
2 000
1 000
0
100 000
50 000
0
600
400
200
0
2015
2020
2025
2030
Year
No DAAs
Status quo
(I) + Blood safety & infection control
(II) + PWID harm reduction
(III) + Offer DAAs at diagnosis
(IV) + Outreach screening

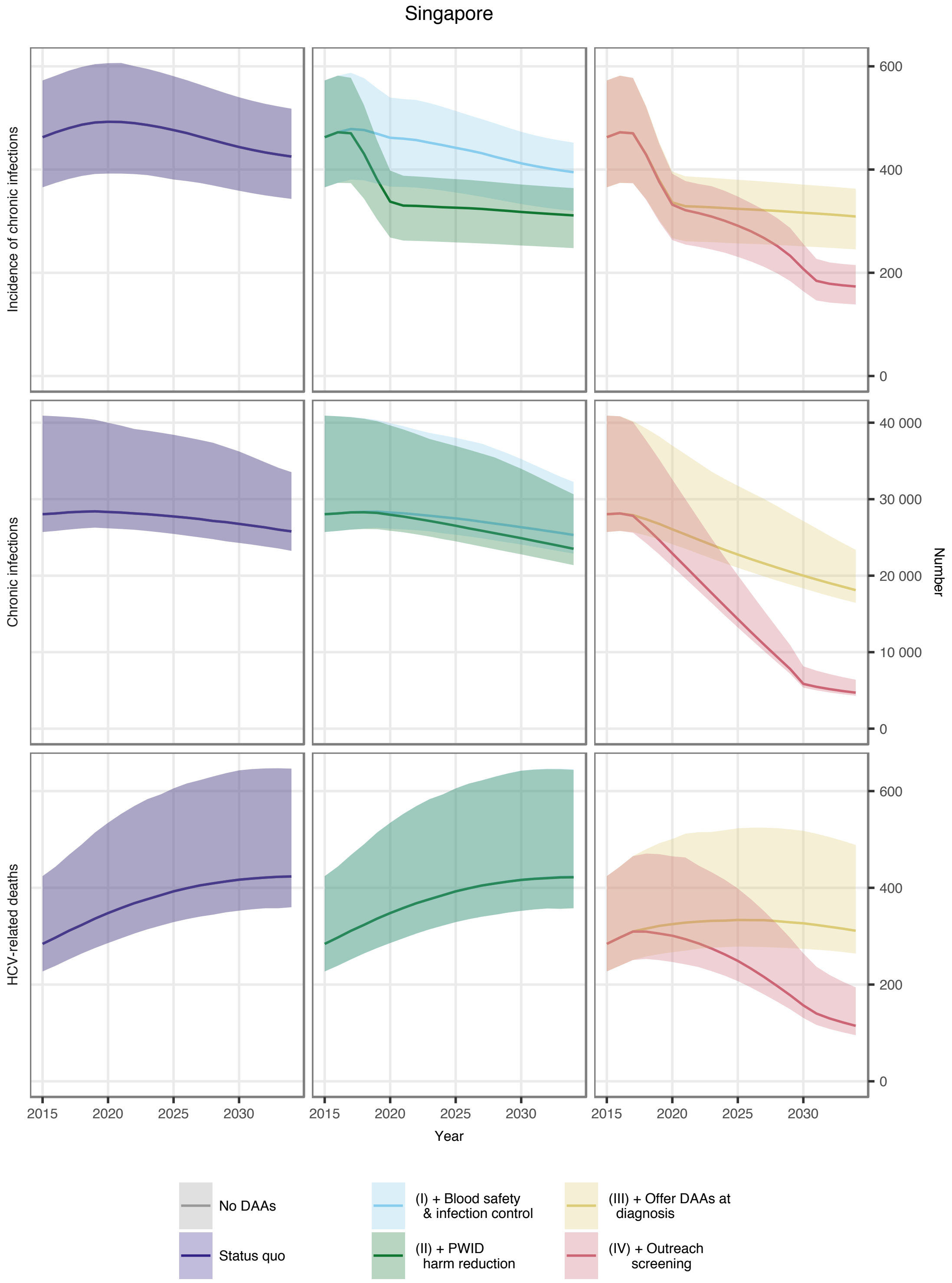
Singapore
Incidence of chronic infections
Chronic infections
HCV-related deaths
Number
Year
2015
2020
2025
2030
0
200
400
600
0
10 000
20 000
30 000
40 000
No DAAs
Status quo
(I) + Blood safety & infection control
(II) + PWID harm reduction
(III) + Offer DAAs at diagnosis
(IV) + Outreach screening

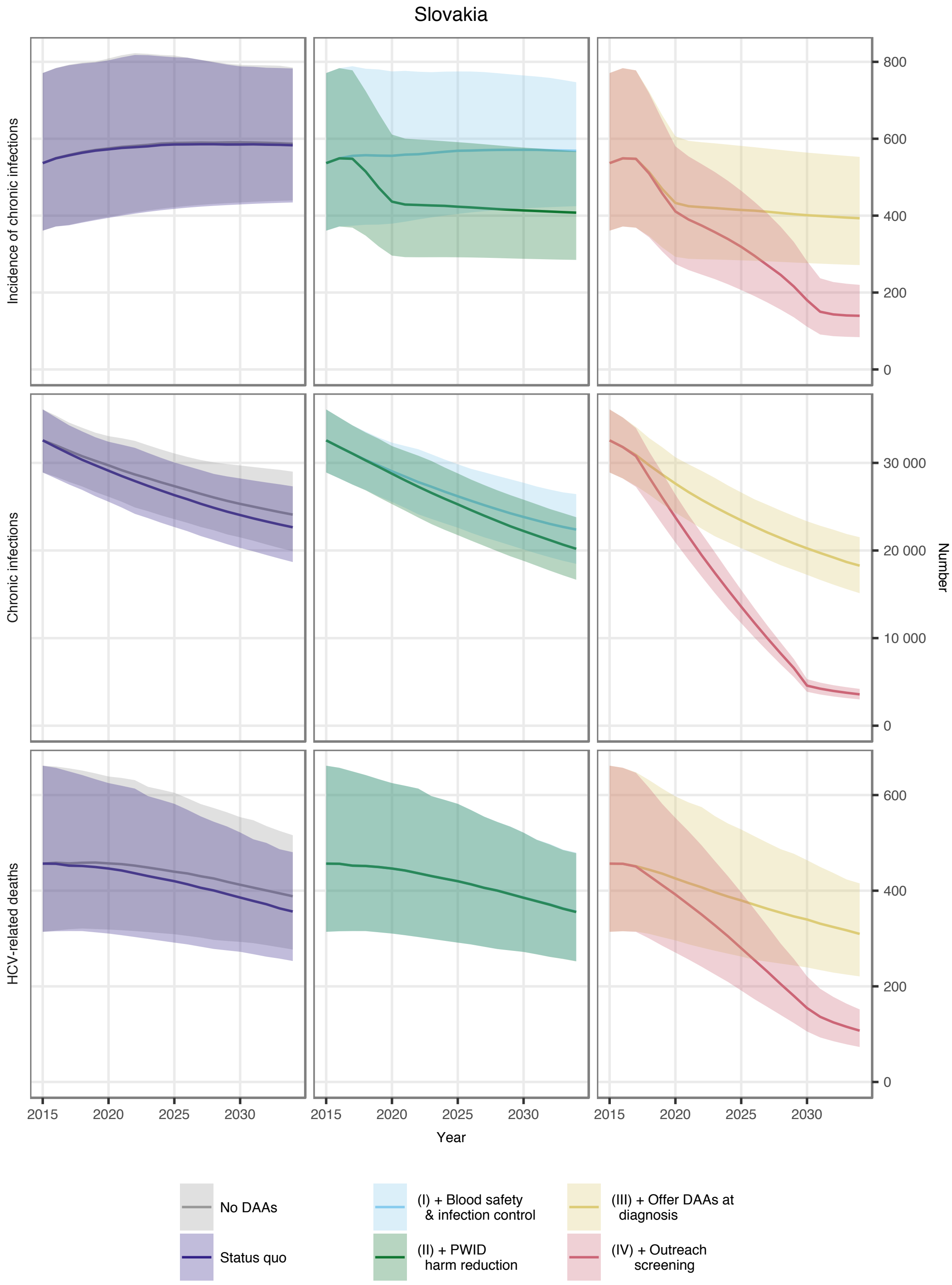
Slovakia
Incidence of chronic infections
Chronic infections
HCV-related deaths
Number
800
600
400
200
0
30 000
20 000
10 000
0
600
400
200
0
2015
2020
2025
2030
Year
No DAAs
Status quo
(I) + Blood safety & infection control
(II) + PWID harm reduction
(III) + Offer DAAs at diagnosis
(IV) + Outreach screening

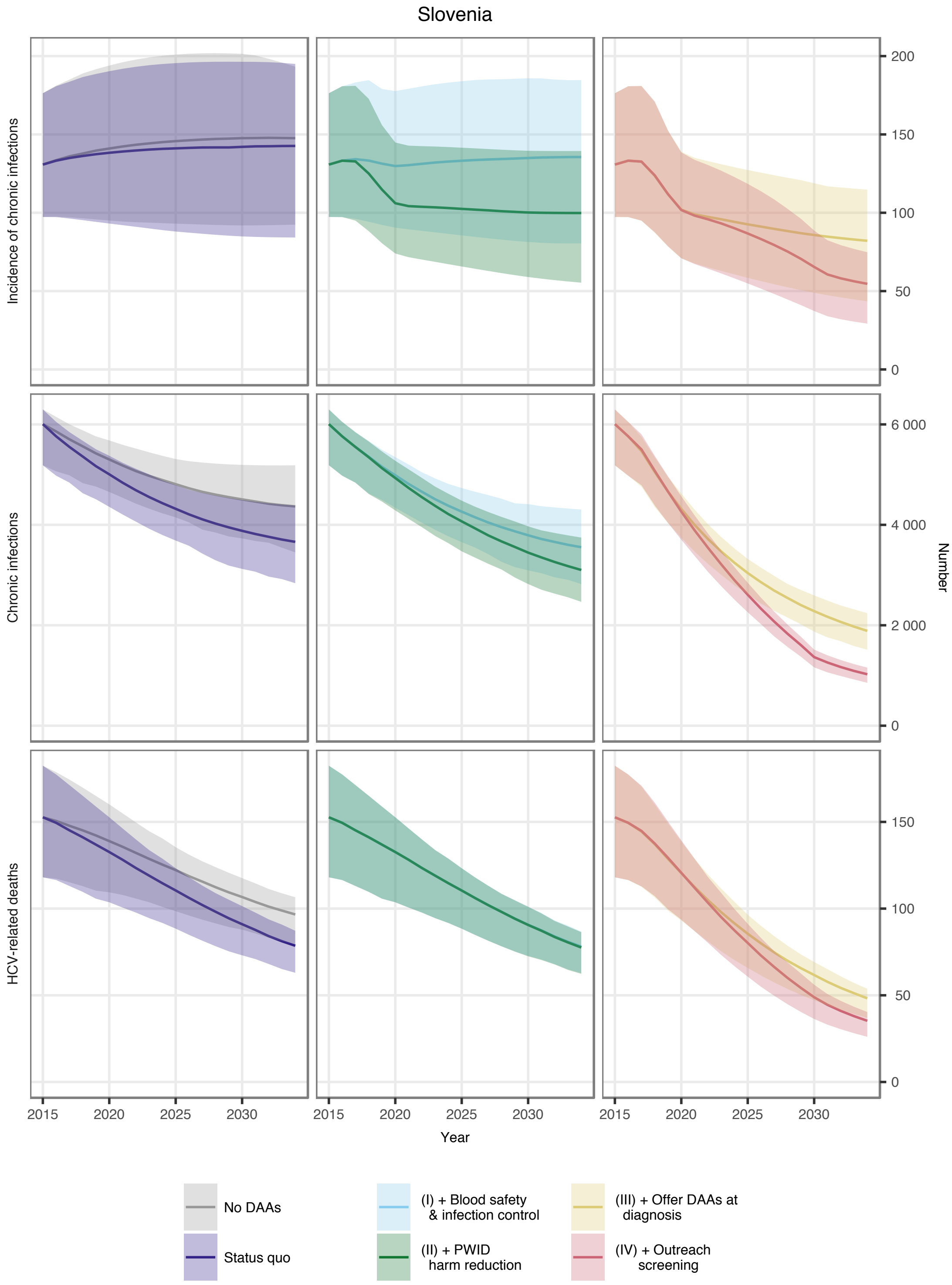

Slovenia
Incidence of chronic infections
Chronic infections
HCV-related deaths
Number
Year
0
50
100
150
200
0
2 000
4 000
6 000
0
50
100
150
2015
2020
2025
2030
No DAAs
Status quo
(I) + Blood safety & infection control
(II) + PWID harm reduction
(III) + Offer DAAs at diagnosis
(IV) + Outreach screening

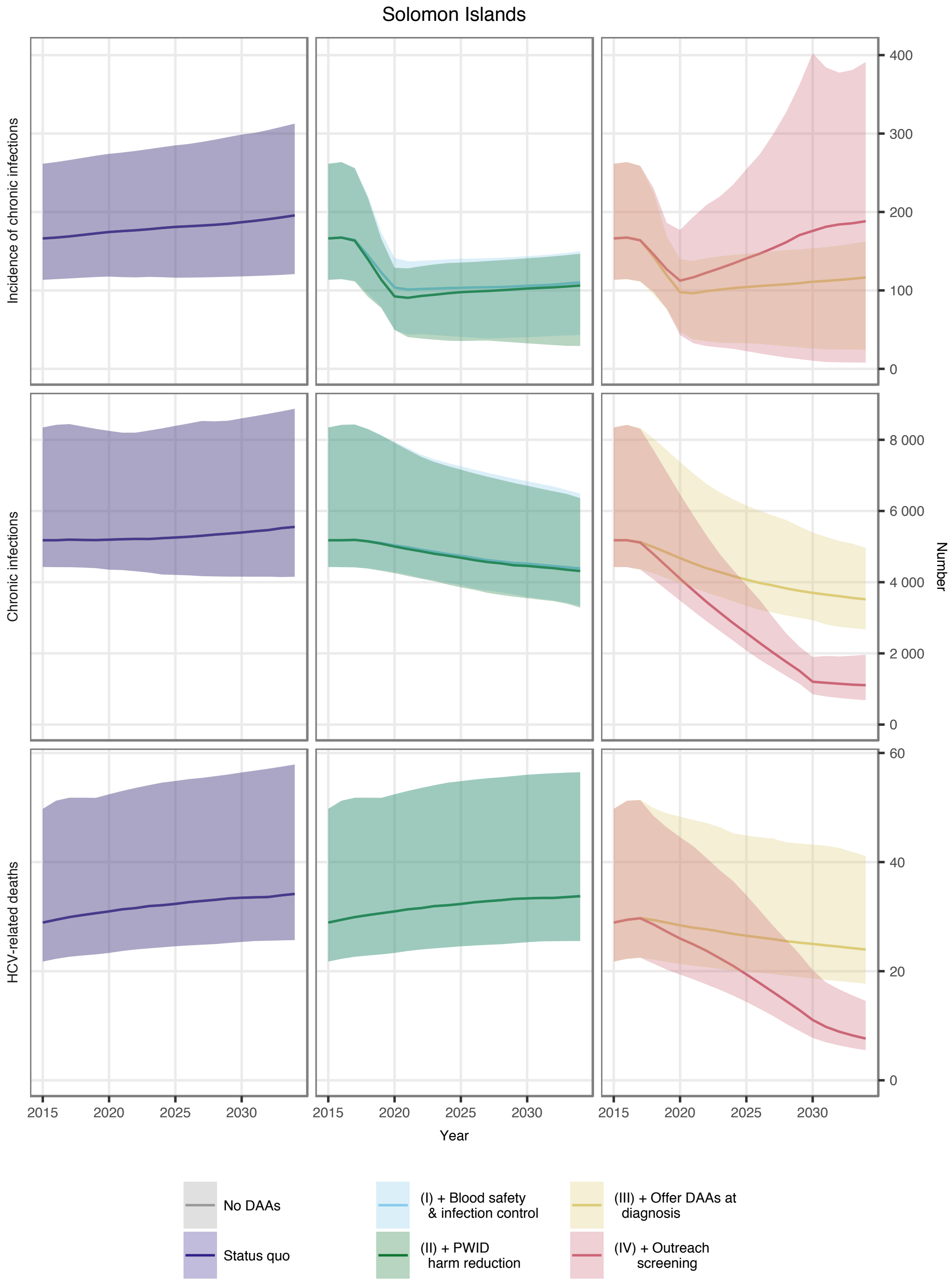

Solomon Islands
Incidence of chronic infections
Chronic infections
HCV-related deaths
Number
Year
0
100
200
300
400
2 000
4 000
6 000
8 000
20
40
60
2015
2020
2025
2030
No DAAs
Status quo
(I) + Blood safety & infection control
(II) + PWID harm reduction
(III) + Offer DAAs at diagnosis
(IV) + Outreach screening

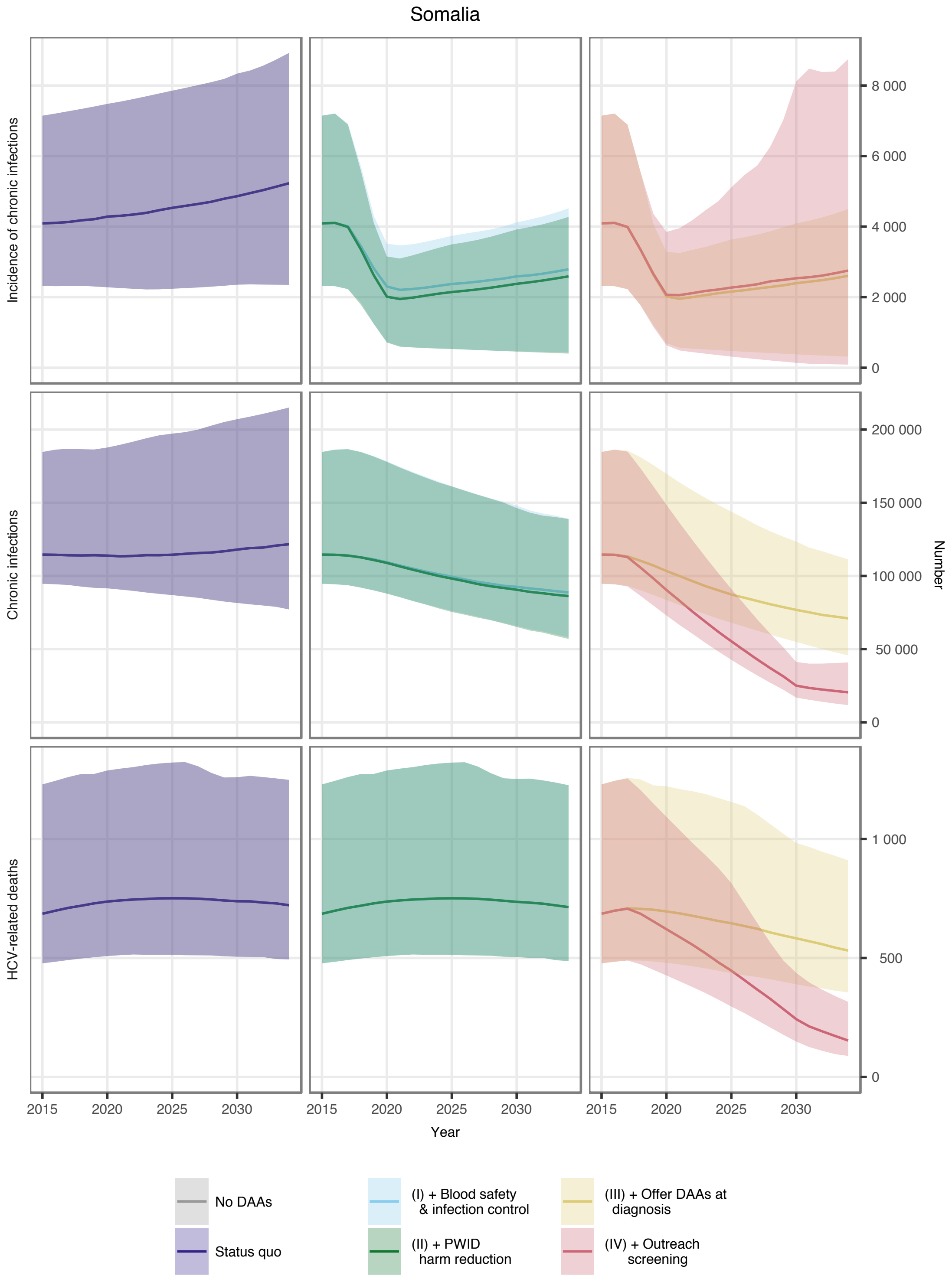
Somalia
Incidence of chronic infections
Chronic infections
HCV-related deaths
Number
8 000
6 000
4 000
2 000
0
200 000
150 000
100 000
50 000
0
1 000
500
0
2015
2020
2025
2030
Year
No DAAs
Status quo
(I) + Blood safety & infection control
(II) + PWID harm reduction
(III) + Offer DAAs at diagnosis
(IV) + Outreach screening

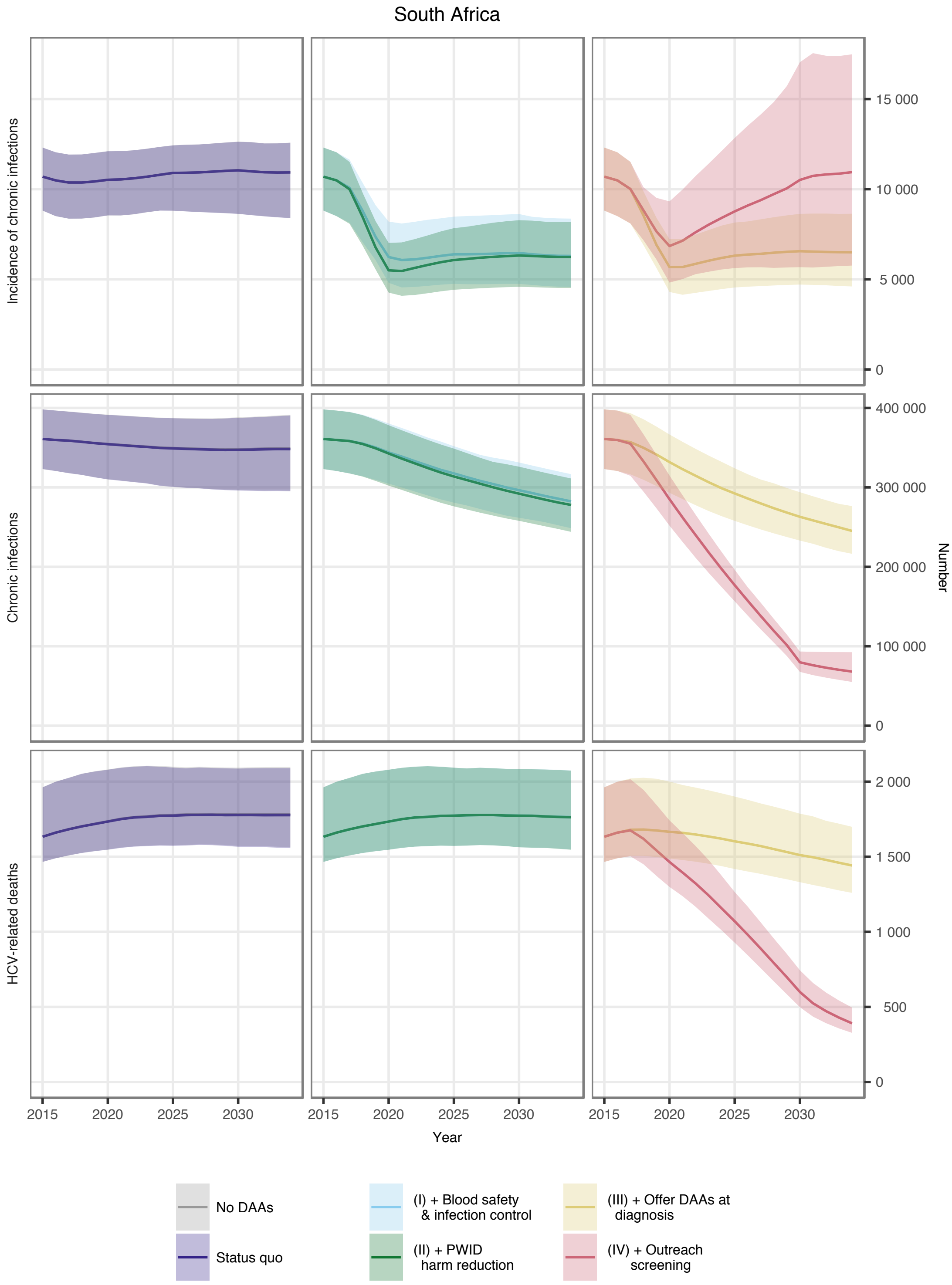
South Africa
Incidence of chronic infections
Chronic infections
HCV-related deaths
Number
15 000
10 000
5 000
0
400 000
300 000
200 000
100 000
0
2 000
1 500
1 000
500
0
2015
2020
2025
2030
Year
No DAAs
Status quo
(I) + Blood safety & infection control
(II) + PWID harm reduction
(III) + Offer DAAs at diagnosis
(IV) + Outreach screening

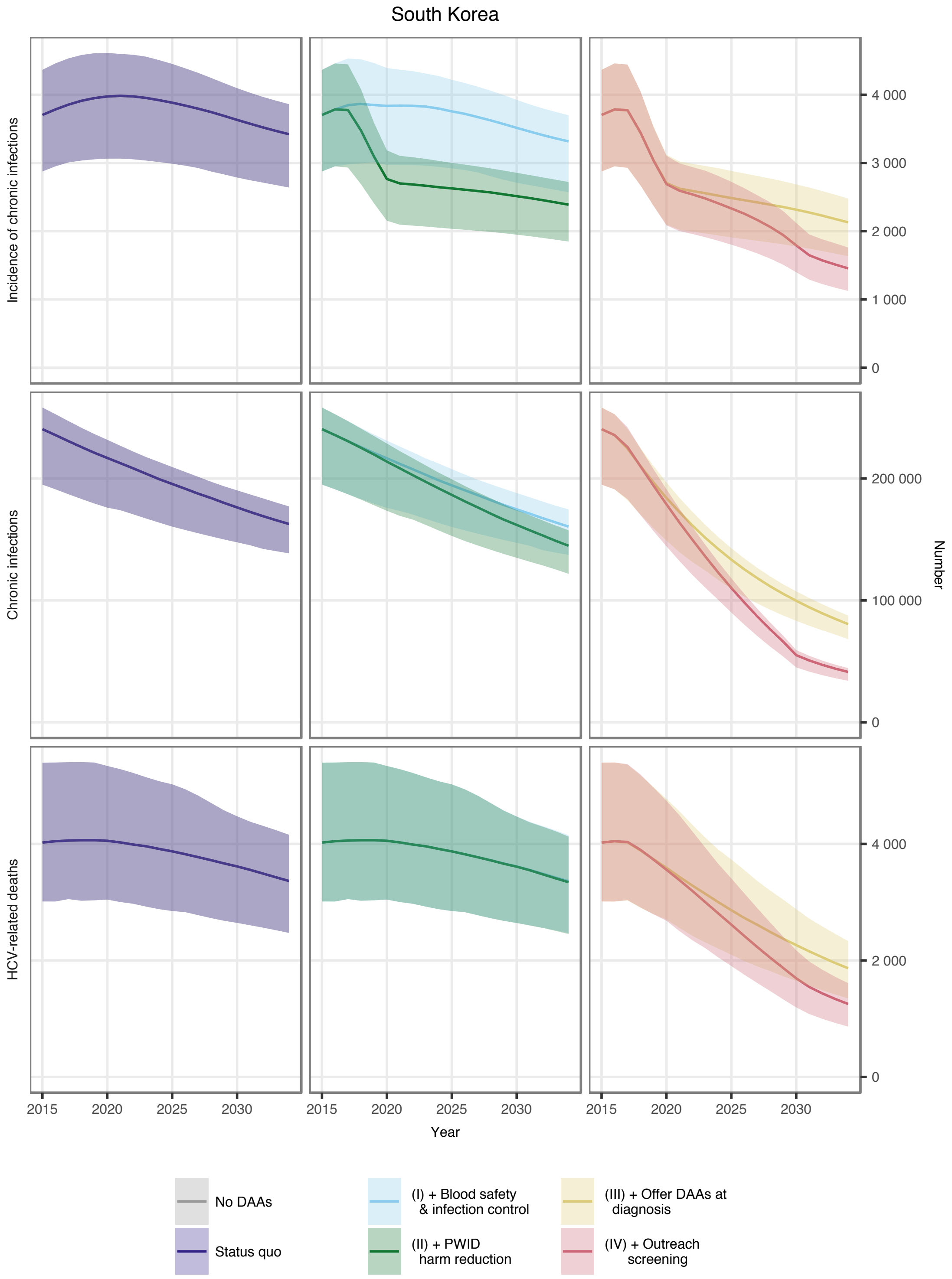
South Korea
Incidence of chronic infections
Chronic infections
HCV-related deaths
Number
Year
0
1 000
2 000
3 000
4 000
0
100 000
200 000
0
2 000
4 000
2015
2020
2025
2030
No DAAs
Status quo
(I) + Blood safety & infection control
(II) + PWID harm reduction
(III) + Offer DAAs at diagnosis
(IV) + Outreach screening

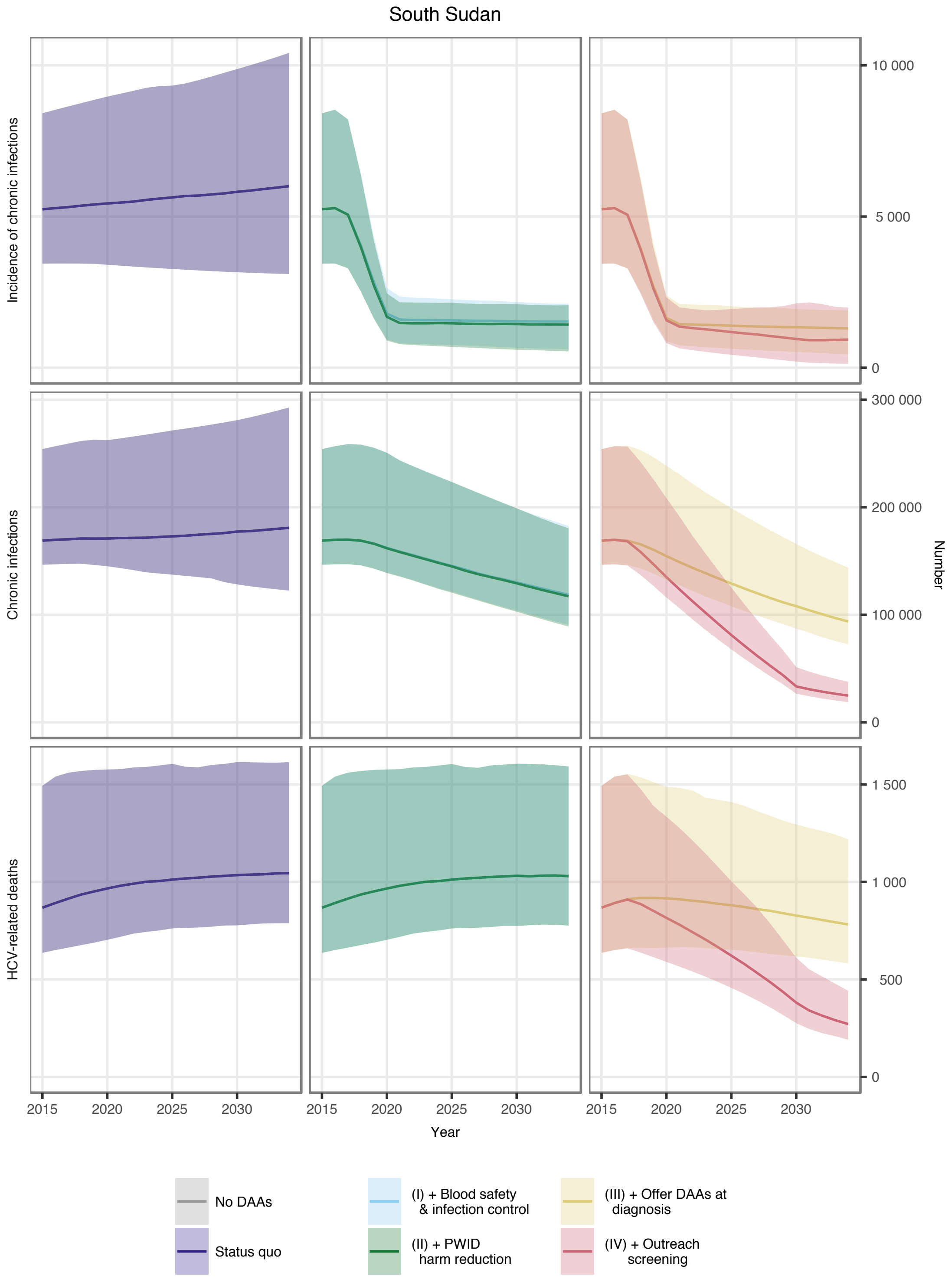
South Sudan
Incidence of chronic infections
Chronic infections
HCV-related deaths
10 000
5 000
0
300 000
200 000
100 000
0
1 500
1 000
500
0
Number
2015
2020
2025
2030
Year
No DAAs
Status quo
(I) + Blood safety & infection control
(II) + PWID harm reduction
(III) + Offer DAAs at diagnosis
(IV) + Outreach screening

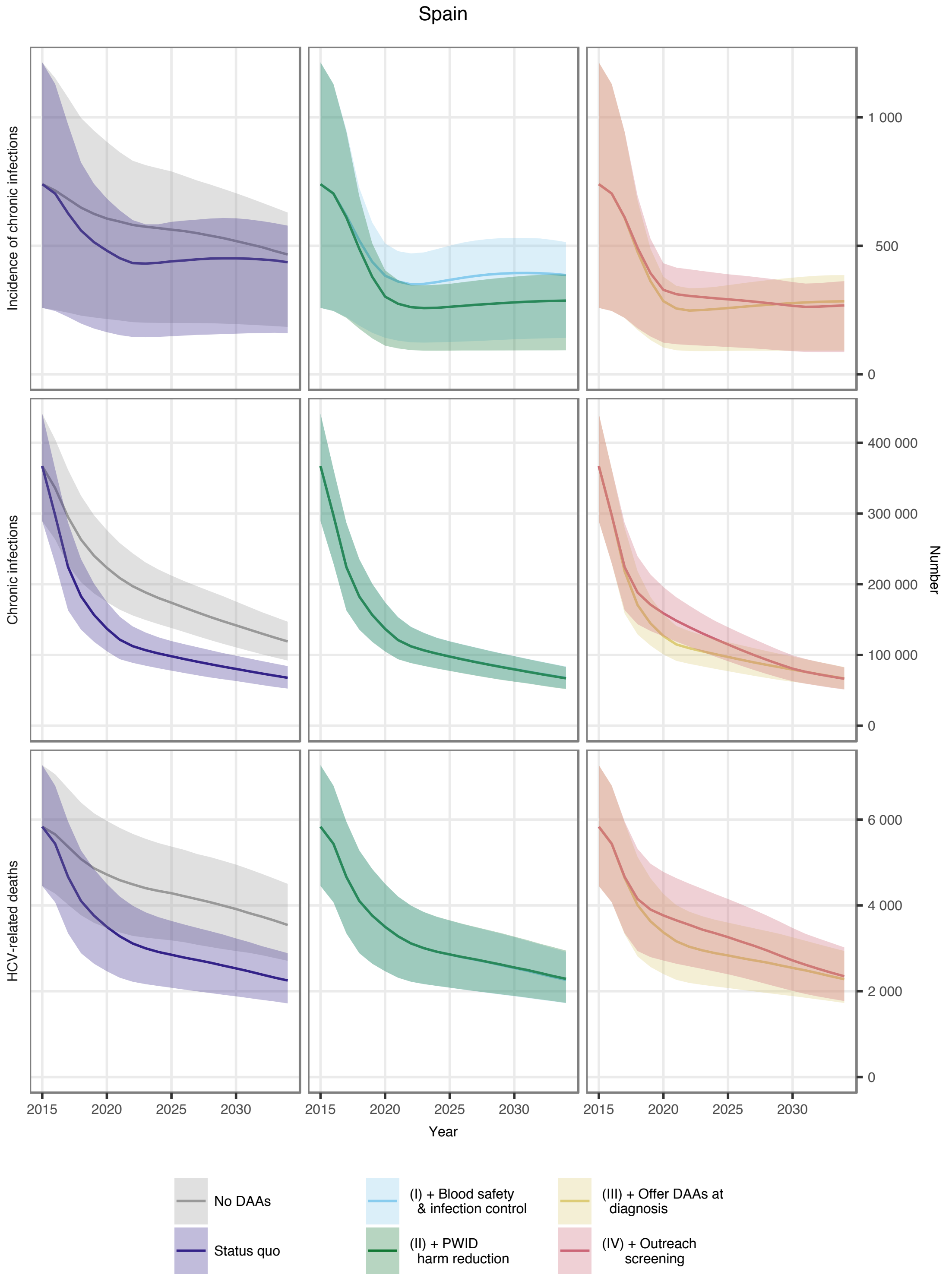
Spain
Incidence of chronic infections
Chronic infections
HCV-related deaths
1 000
500
0
400 000
300 000
200 000
100 000
0
6 000
4 000
2 000
0
Number
2015
2020
2025
2030
Year
No DAAs
Status quo
(I) + Blood safety & infection control
(II) + PWID harm reduction
(III) + Offer DAAs at diagnosis
(IV) + Outreach screening

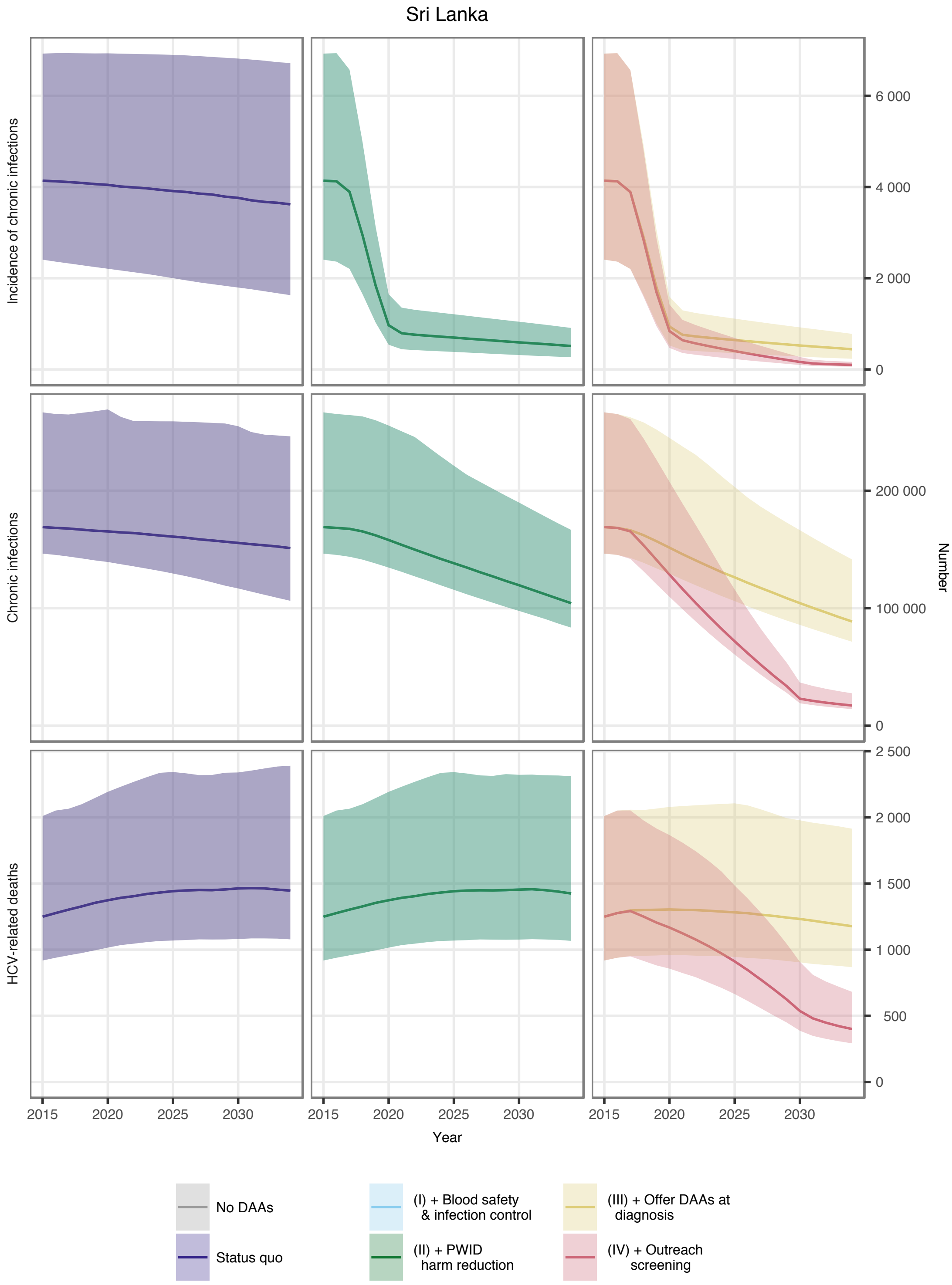
Sri Lanka
Incidence of chronic infections
Chronic infections
HCV-related deaths
Number
Year
2015
2020
2025
2030
6 000
4 000
2 000
0
200 000
100 000
0
2 500
2 000
1 500
1 000
500
0
No DAAs
Status quo
(I) + Blood safety & infection control
(II) + PWID harm reduction
(III) + Offer DAAs at diagnosis
(IV) + Outreach screening

Palestine, State of

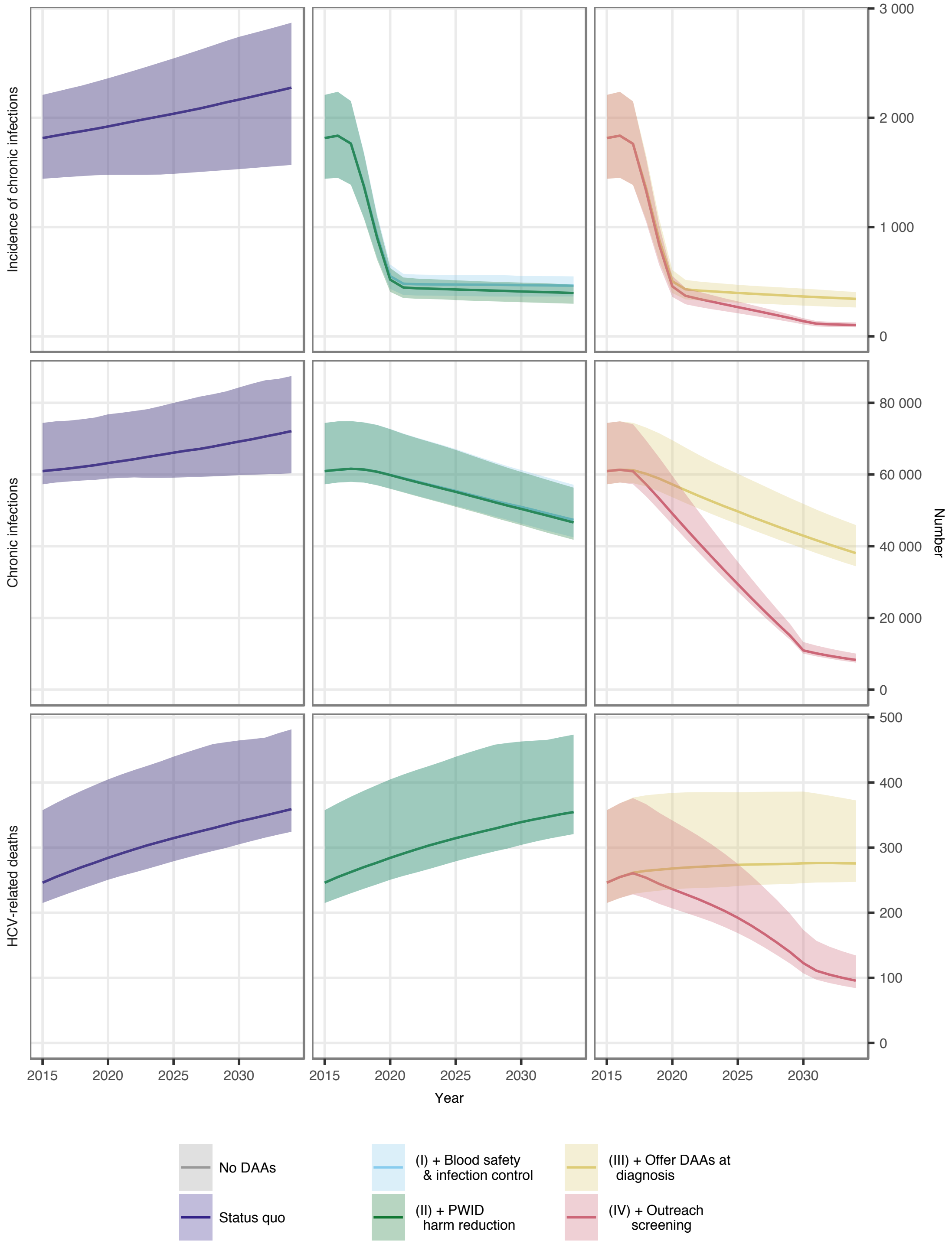

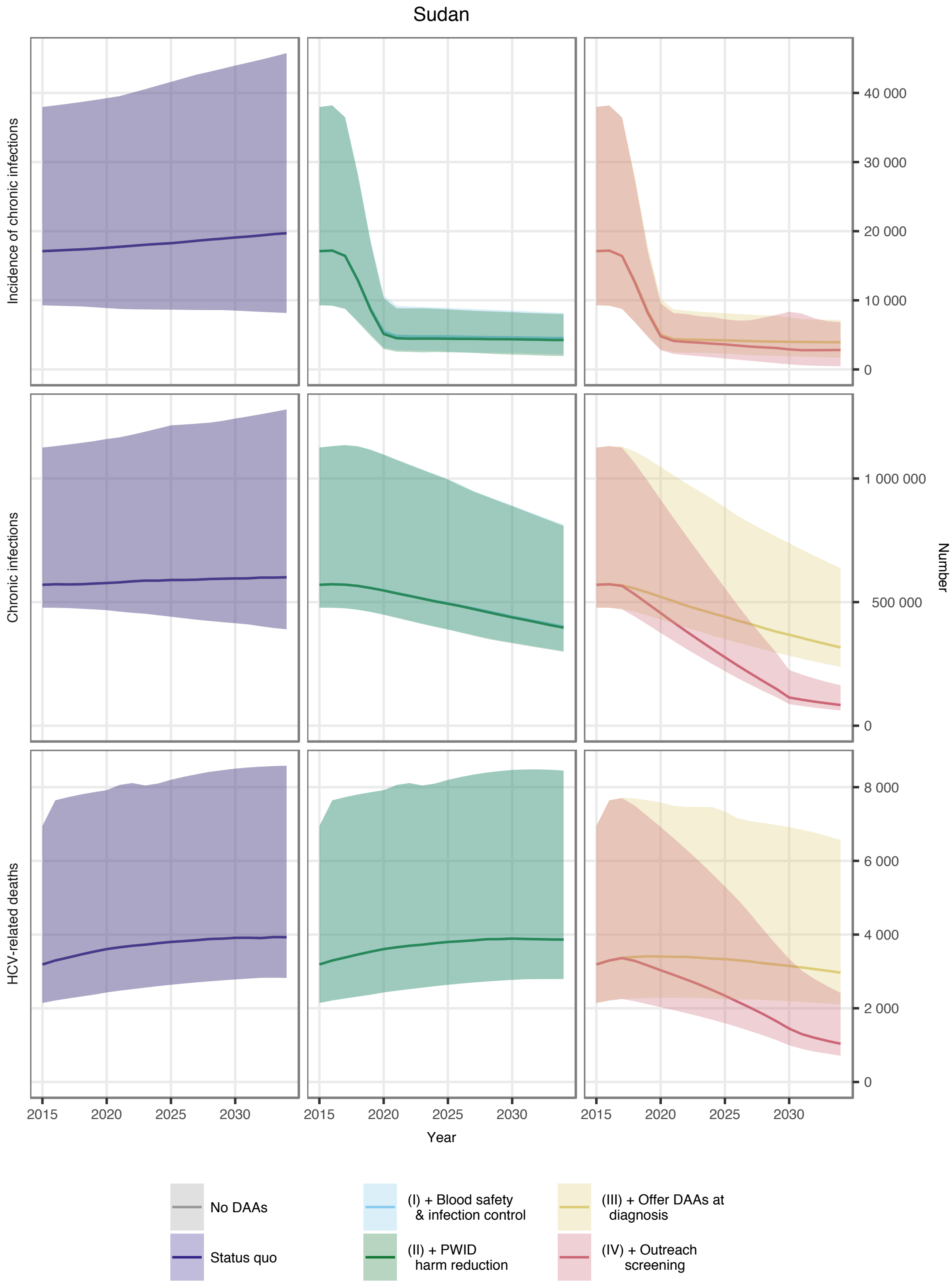

Sudan
Incidence of chronic infections
Chronic infections
HCV-related deaths
Number
40 000
30 000
20 000
10 000
0
1 000 000
500 000
0
8 000
6 000
4 000
2 000
0
2015
2020
2025
2030
Year
No DAAs
Status quo
(I) + Blood safety & infection control
(II) + PWID harm reduction
(III) + Offer DAAs at diagnosis
(IV) + Outreach screening

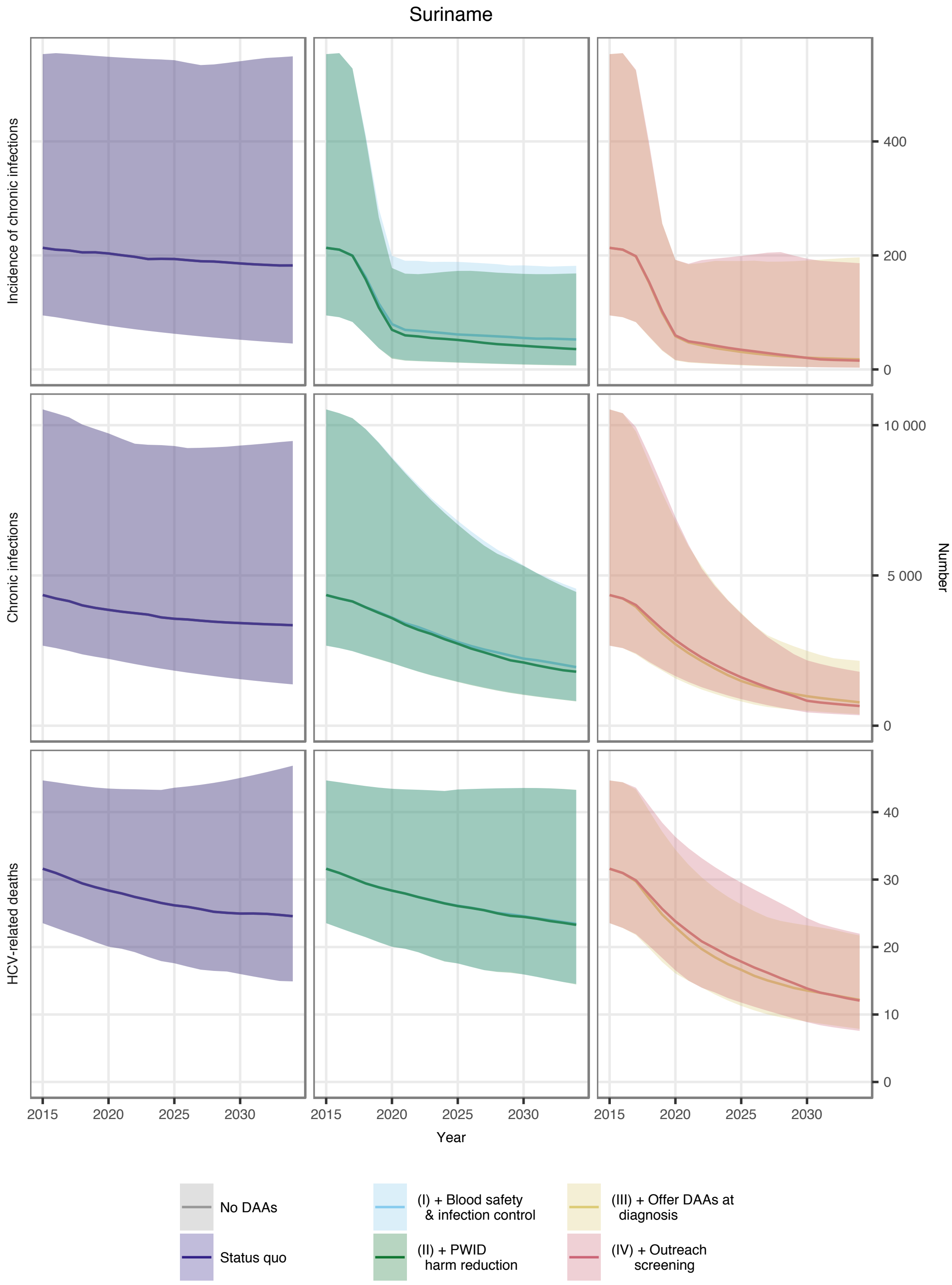
Suriname
Incidence of chronic infections
Chronic infections
HCV-related deaths
Number
Year
2015
2020
2025
2030
0
200
400
5 000
10 000
10
20
30
40
No DAAs
Status quo
(I) + Blood safety & infection control
(II) + PWID harm reduction
(III) + Offer DAAs at diagnosis
(IV) + Outreach screening

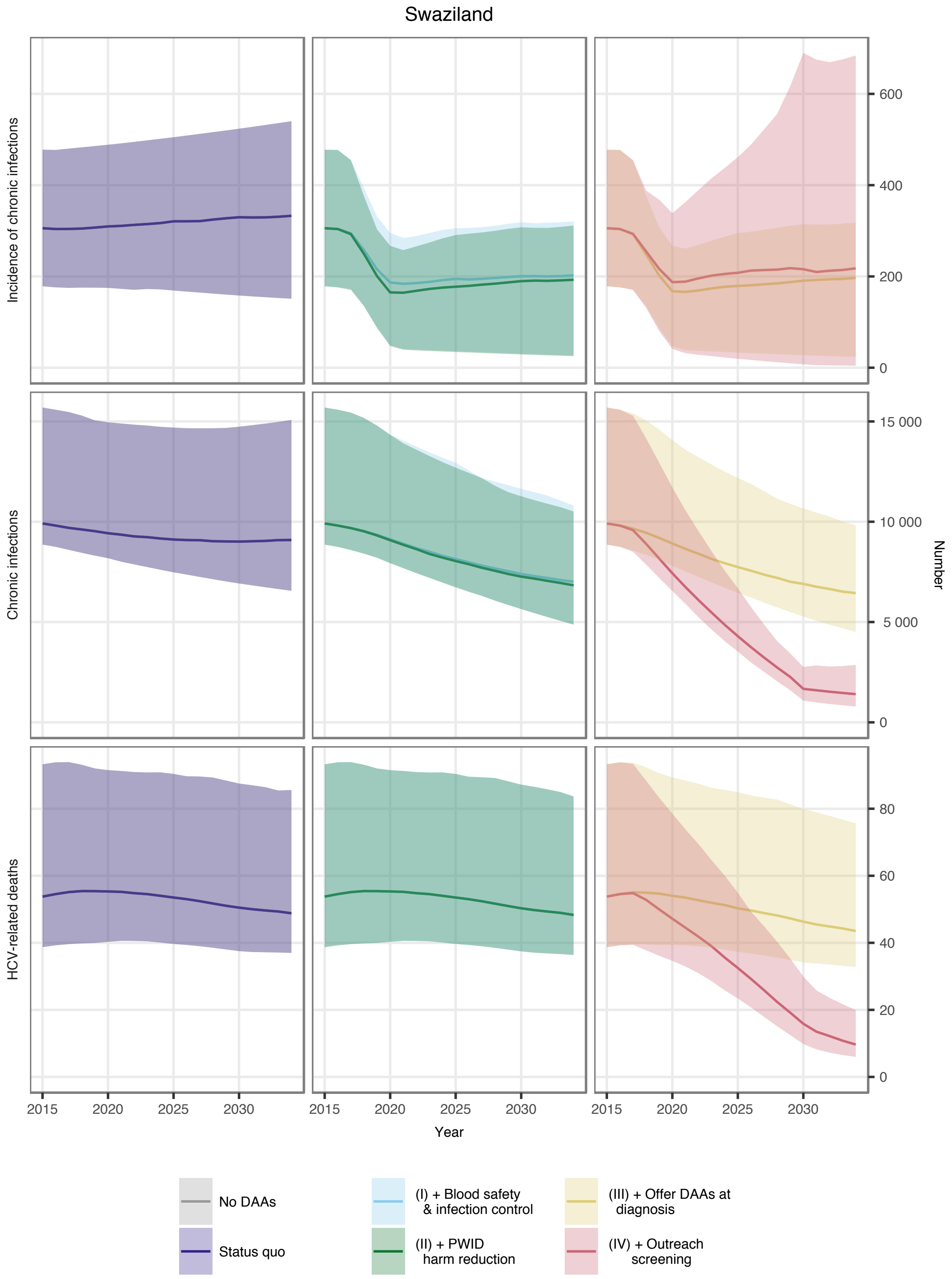

Swaziland
Incidence of chronic infections
Chronic infections
HCV-related deaths
Number
Year
2015
2020
2025
2030
0
200
400
600
0
5 000
10 000
15 000
0
20
40
60
80
No DAAs
Status quo
(I) + Blood safety & infection control
(II) + PWID harm reduction
(III) + Offer DAAs at diagnosis
(IV) + Outreach screening

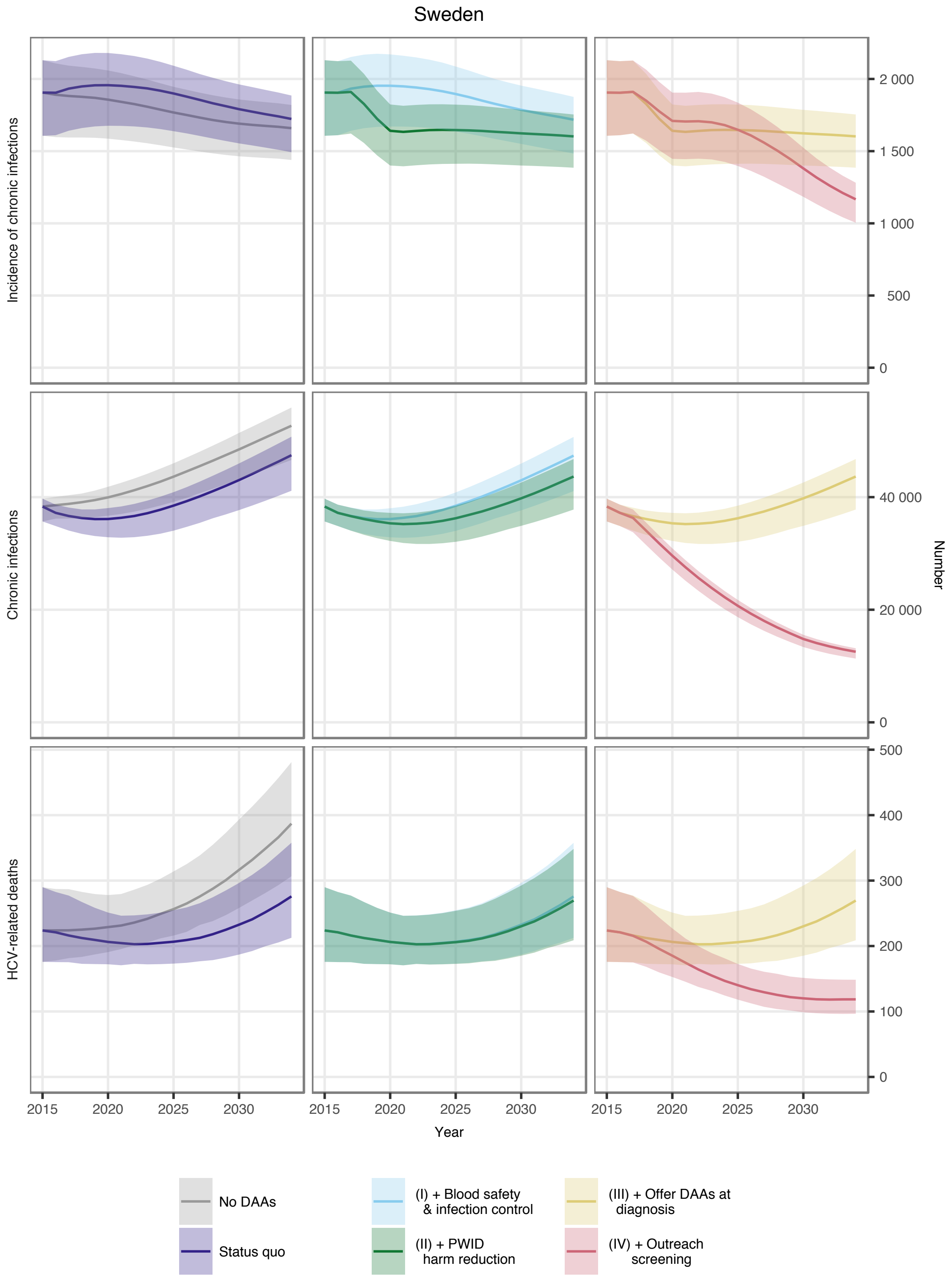
Sweden
Incidence of chronic infections
Chronic infections
HCV-related deaths
Number
Year
2015
2020
2025
2030
0
500
1 000
1 500
2 000
20 000
40 000
100
200
300
400
No DAAs
Status quo
(I) + Blood safety & infection control
(II) + PWID harm reduction
(III) + Offer DAAs at diagnosis
(IV) + Outreach screening

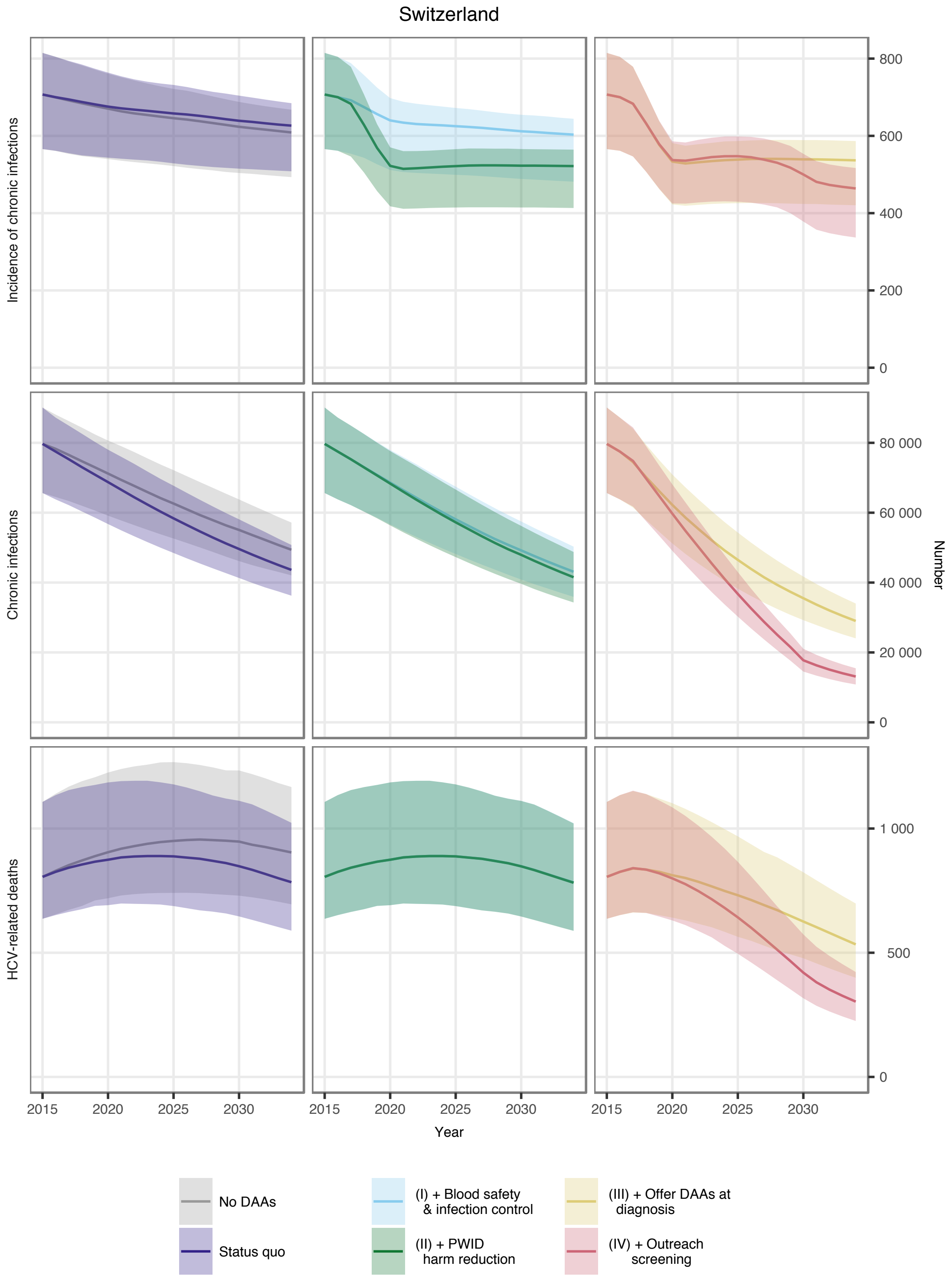

Switzerland
Incidence of chronic infections
Chronic infections
HCV-related deaths
Number
Year
2015
2020
2025
2030
800
600
400
200
0
80 000
60 000
40 000
20 000
0
1 000
500
0
No DAAs
Status quo
(I) + Blood safety & infection control
(II) + PWID harm reduction
(III) + Offer DAAs at diagnosis
(IV) + Outreach screening

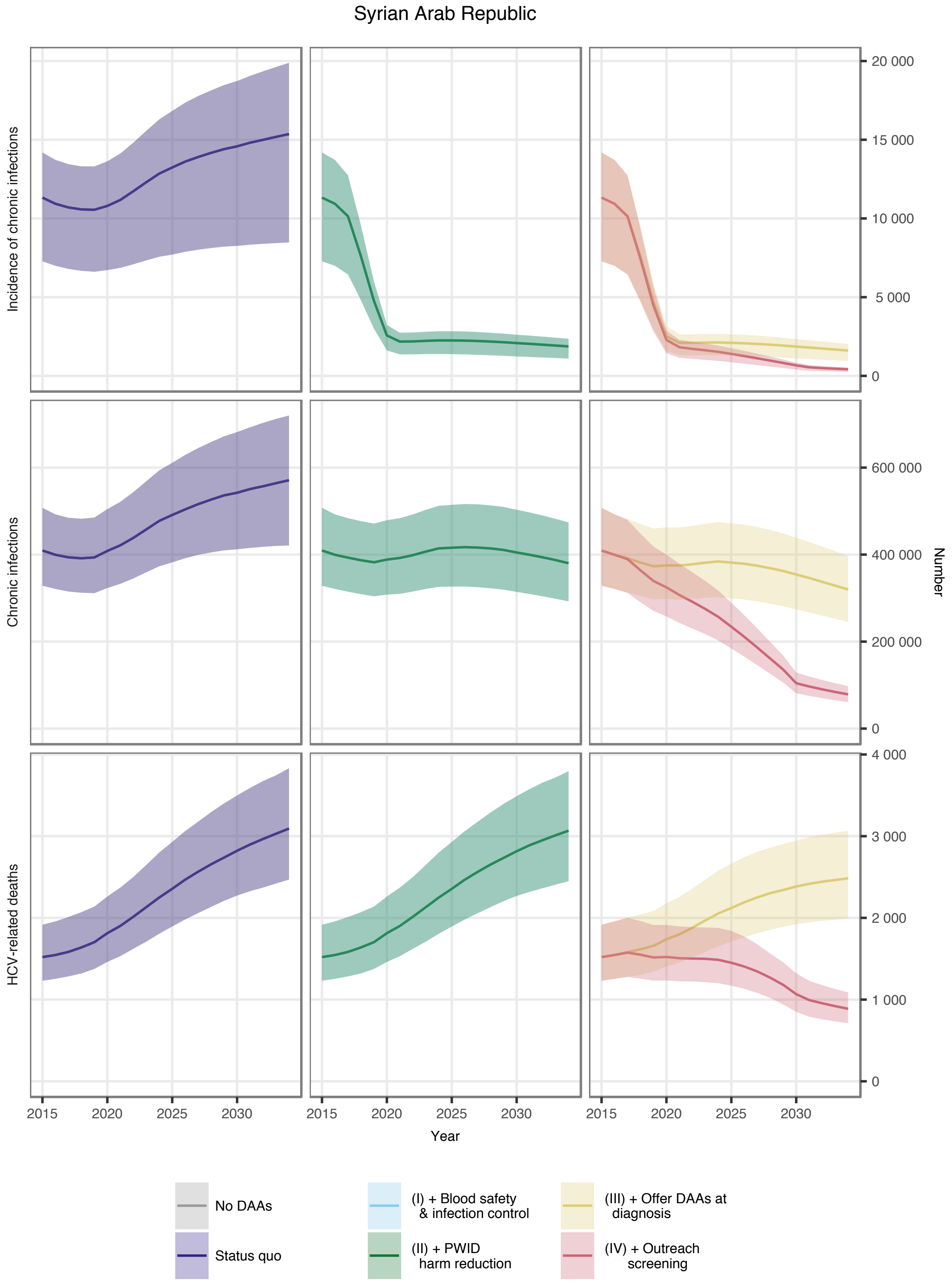

Syrian Arab Republic
Incidence of chronic infections
Chronic infections
HCV-related deaths
Number
20 000
15 000
10 000
5 000
0
600 000
400 000
200 000
0
4 000
3 000
2 000
1 000
0
2015
2020
2025
2030
Year
No DAAs
Status quo
(I) + Blood safety & infection control
(II) + PWID harm reduction
(III) + Offer DAAs at diagnosis
(IV) + Outreach screening

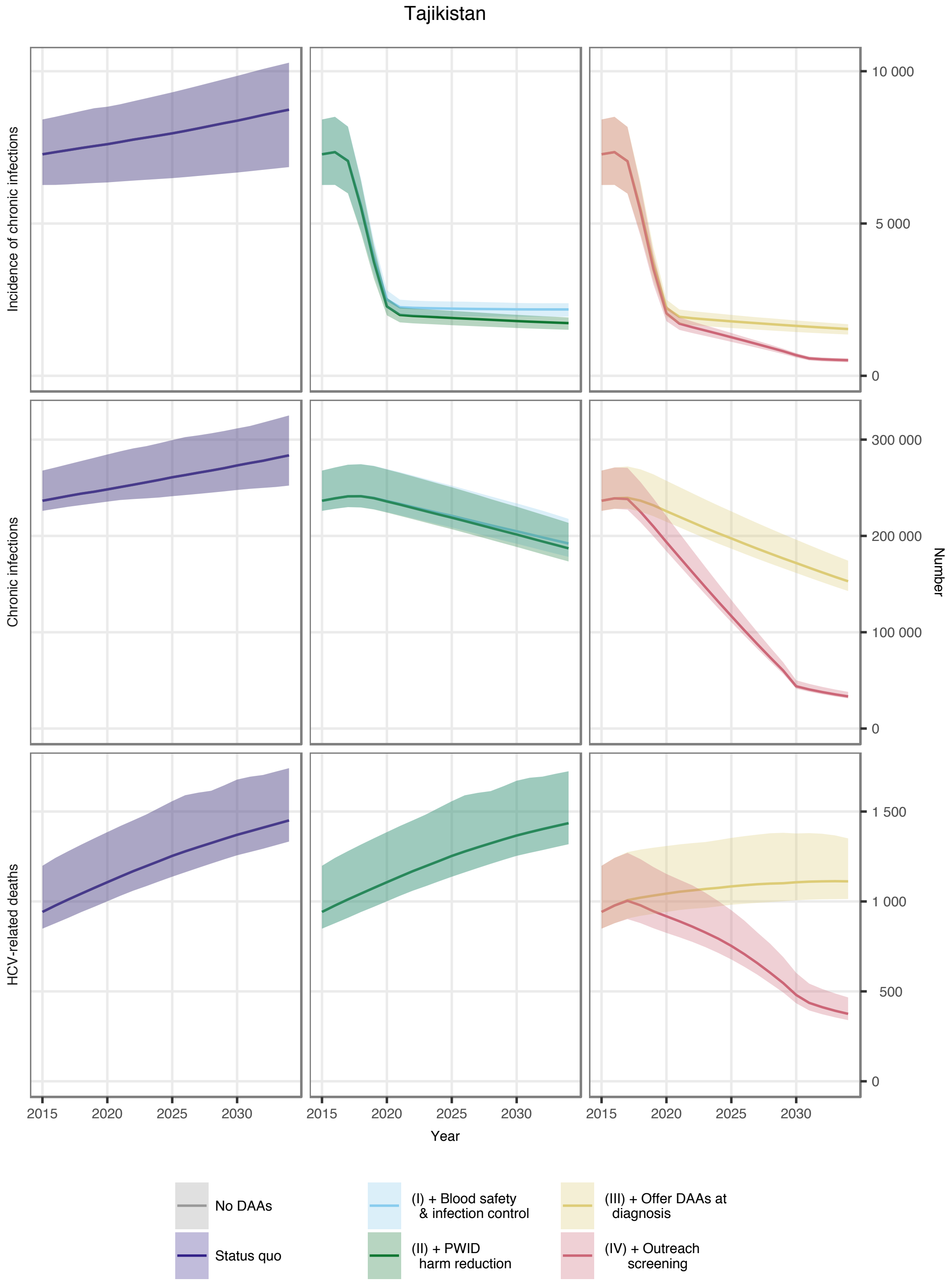

Tajikistan
Incidence of chronic infections
Chronic infections
HCV-related deaths
10 000
5 000
0
300 000
200 000
100 000
0
1 500
1 000
500
0
Number
2015
2020
2025
2030
Year
No DAAs
Status quo
(I) + Blood safety & infection control
(II) + PWID harm reduction
(III) + Offer DAAs at diagnosis
(IV) + Outreach screening

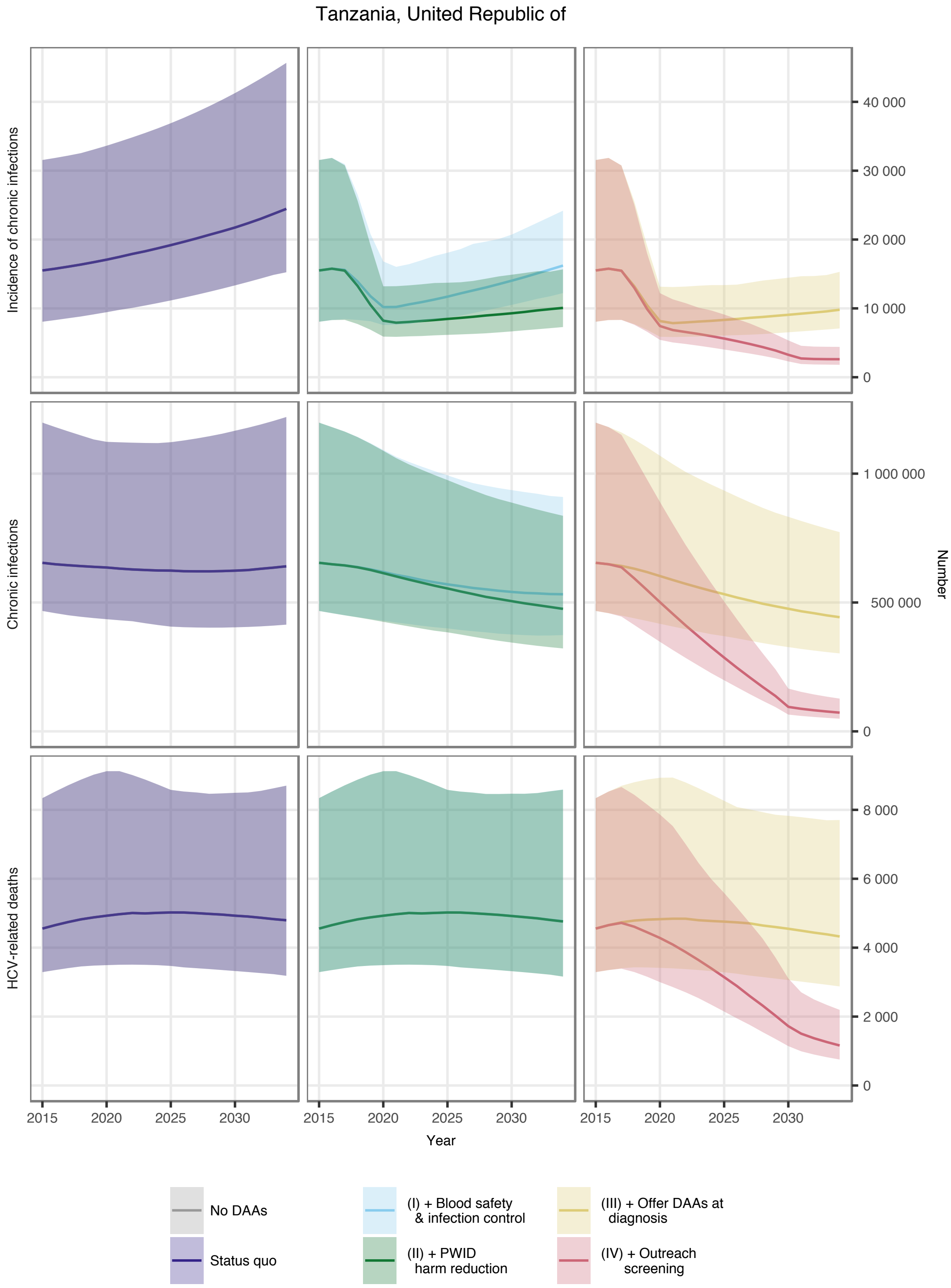

Tanzania, United Republic of
Incidence of chronic infections
Chronic infections
HCV-related deaths
Number
40 000
30 000
20 000
10 000
0
1 000 000
500 000
0
8 000
6 000
4 000
2 000
0
2015
2020
2025
2030
Year
No DAAs
Status quo
(I) + Blood safety & infection control
(II) + PWID harm reduction
(III) + Offer DAAs at diagnosis
(IV) + Outreach screening

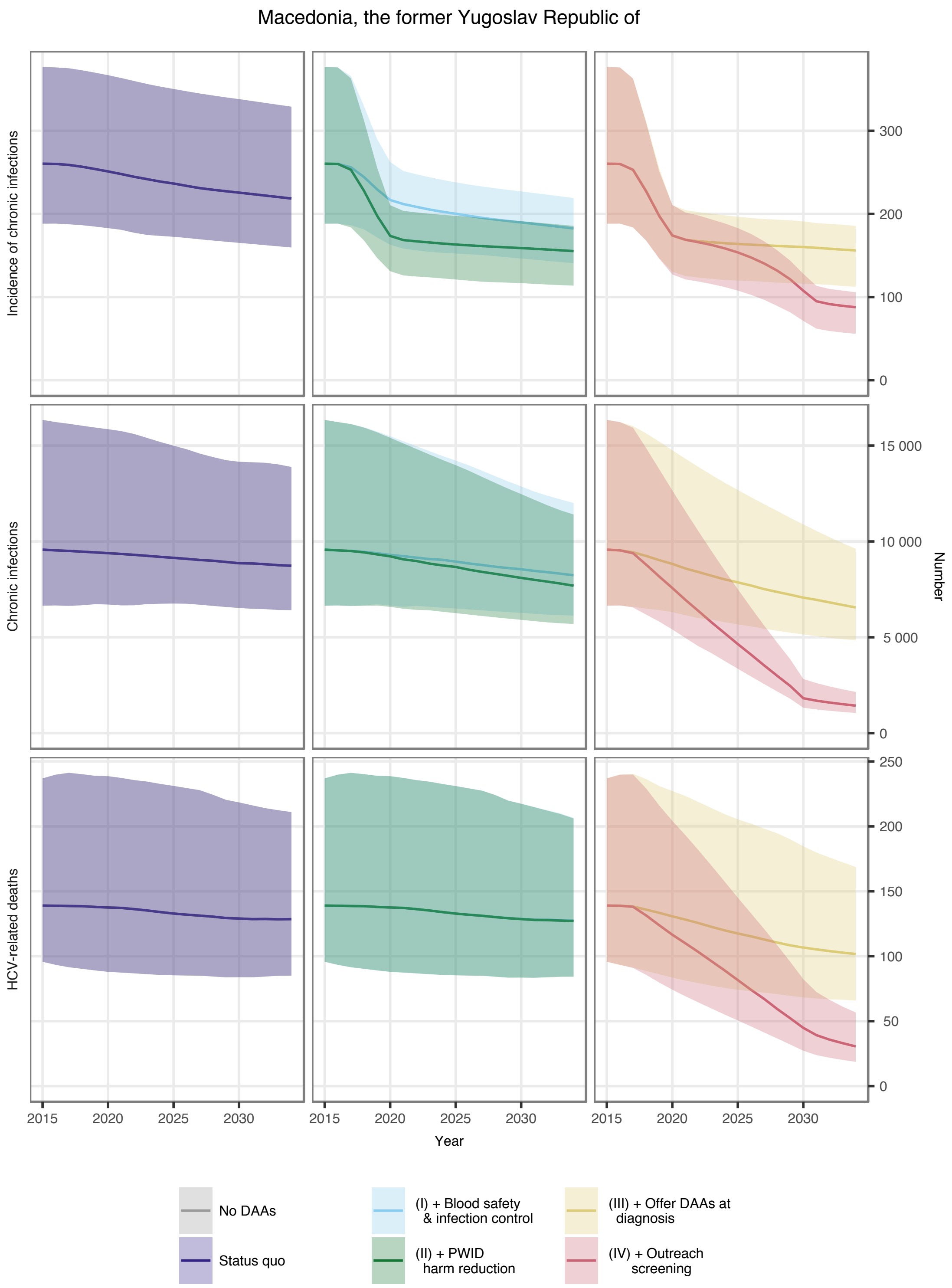
Macedonia, the former Yugoslav Republic of
Incidence of chronic infections
Chronic infections
HCV-related deaths
Number
Year
0
100
200
300
5 000
10 000
15 000
50
150
250
2015
2020
2025
2030
No DAAs
Status quo
(I) + Blood safety & infection control
(II) + PWID harm reduction
(III) + Offer DAAs at diagnosis
(IV) + Outreach screening

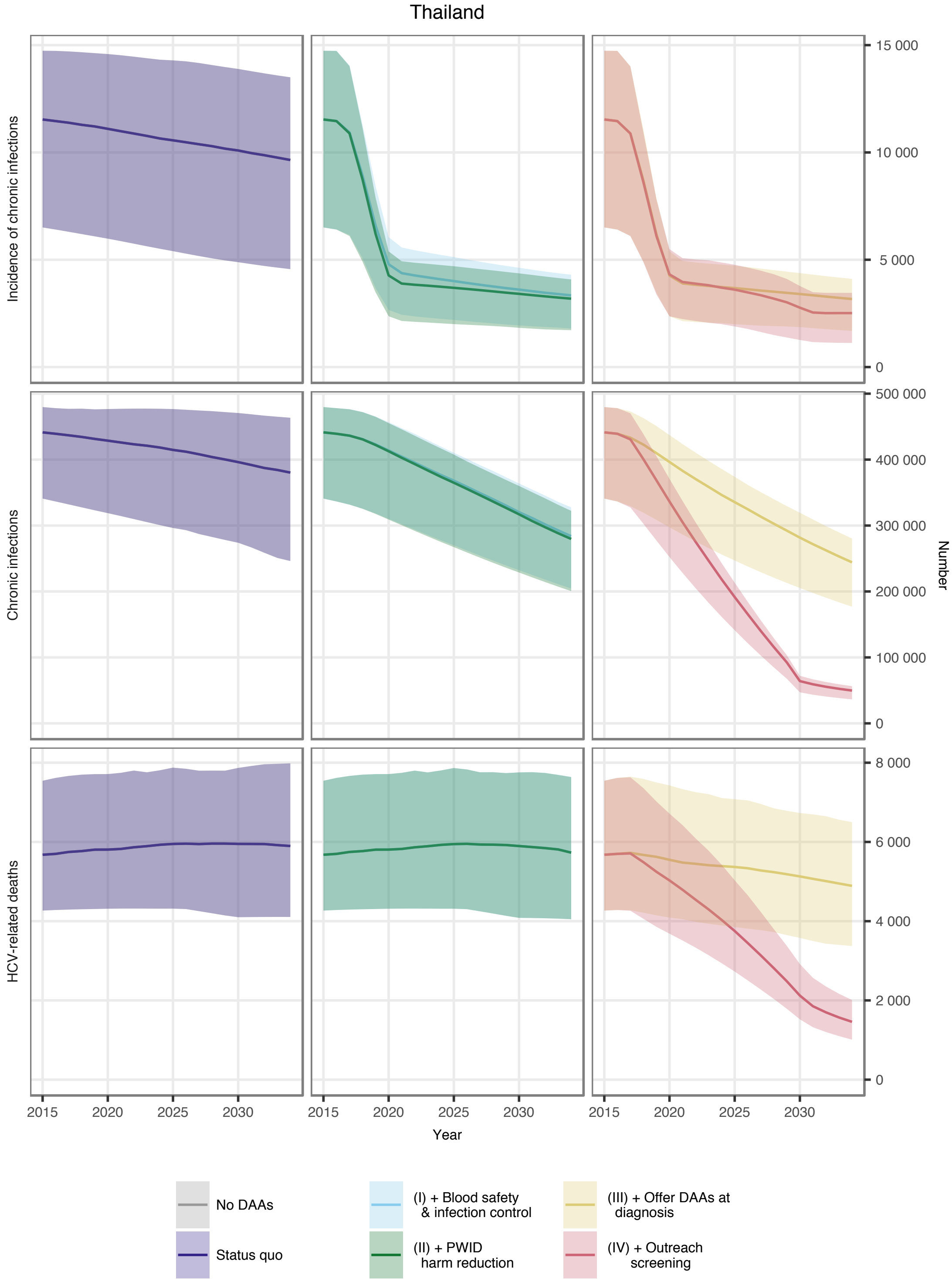
Thailand
Incidence of chronic infections
Chronic infections
HCV-related deaths
Number
15 000
10 000
5 000
0
500 000
400 000
300 000
200 000
100 000
0
8 000
6 000
4 000
2 000
0
2015
2020
2025
2030
Year
No DAAs
Status quo
(I) + Blood safety & infection control
(II) + PWID harm reduction
(III) + Offer DAAs at diagnosis
(IV) + Outreach screening

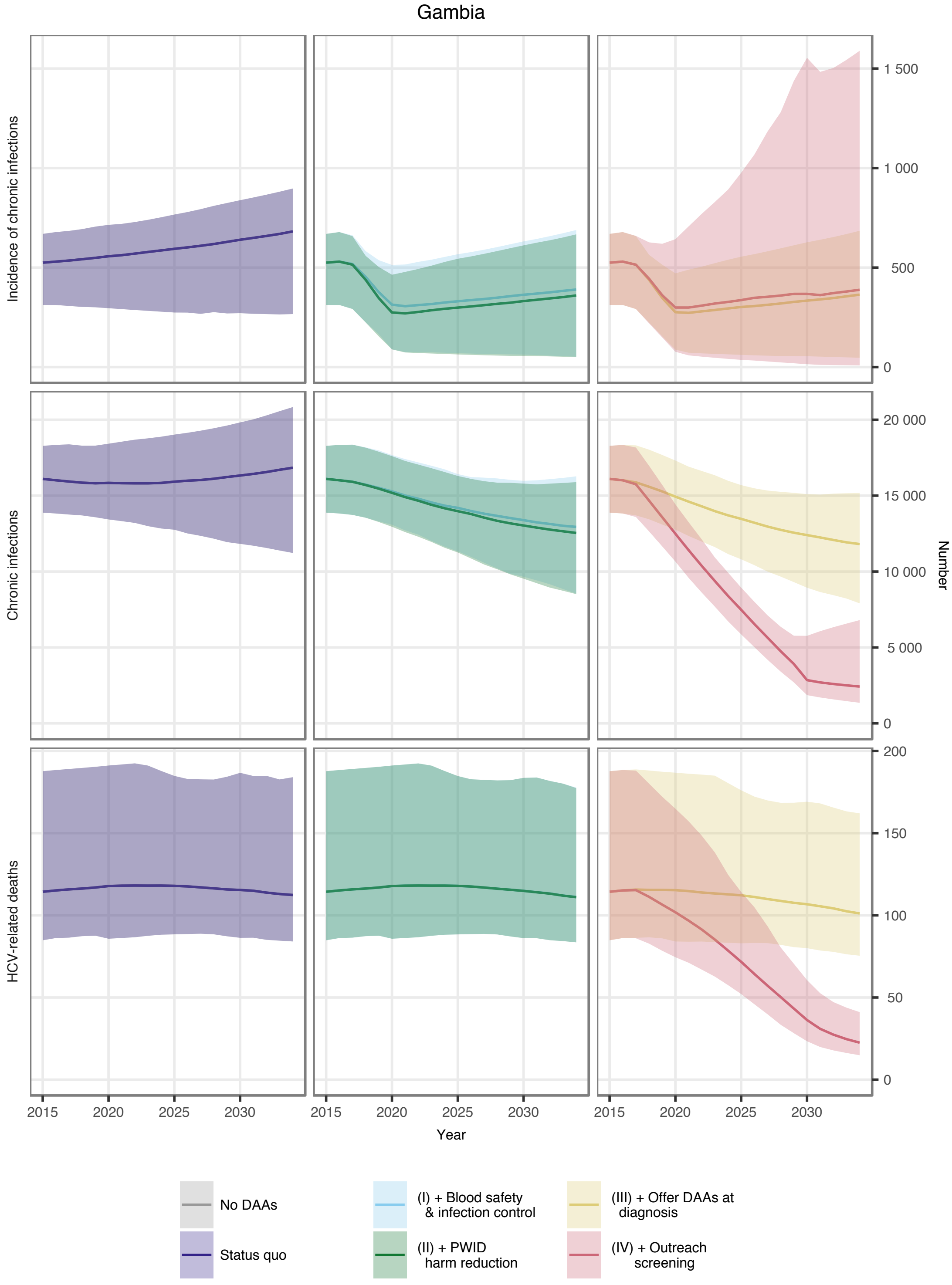

Gambia
Incidence of chronic infections
Chronic infections
HCV-related deaths
Number
1 500
1 000
500
0
20 000
15 000
10 000
5 000
0
200
150
100
50
0
2015
2020
2025
2030
Year
No DAAs
Status quo
(I) + Blood safety & infection control
(II) + PWID harm reduction
(III) + Offer DAAs at diagnosis
(IV) + Outreach screening

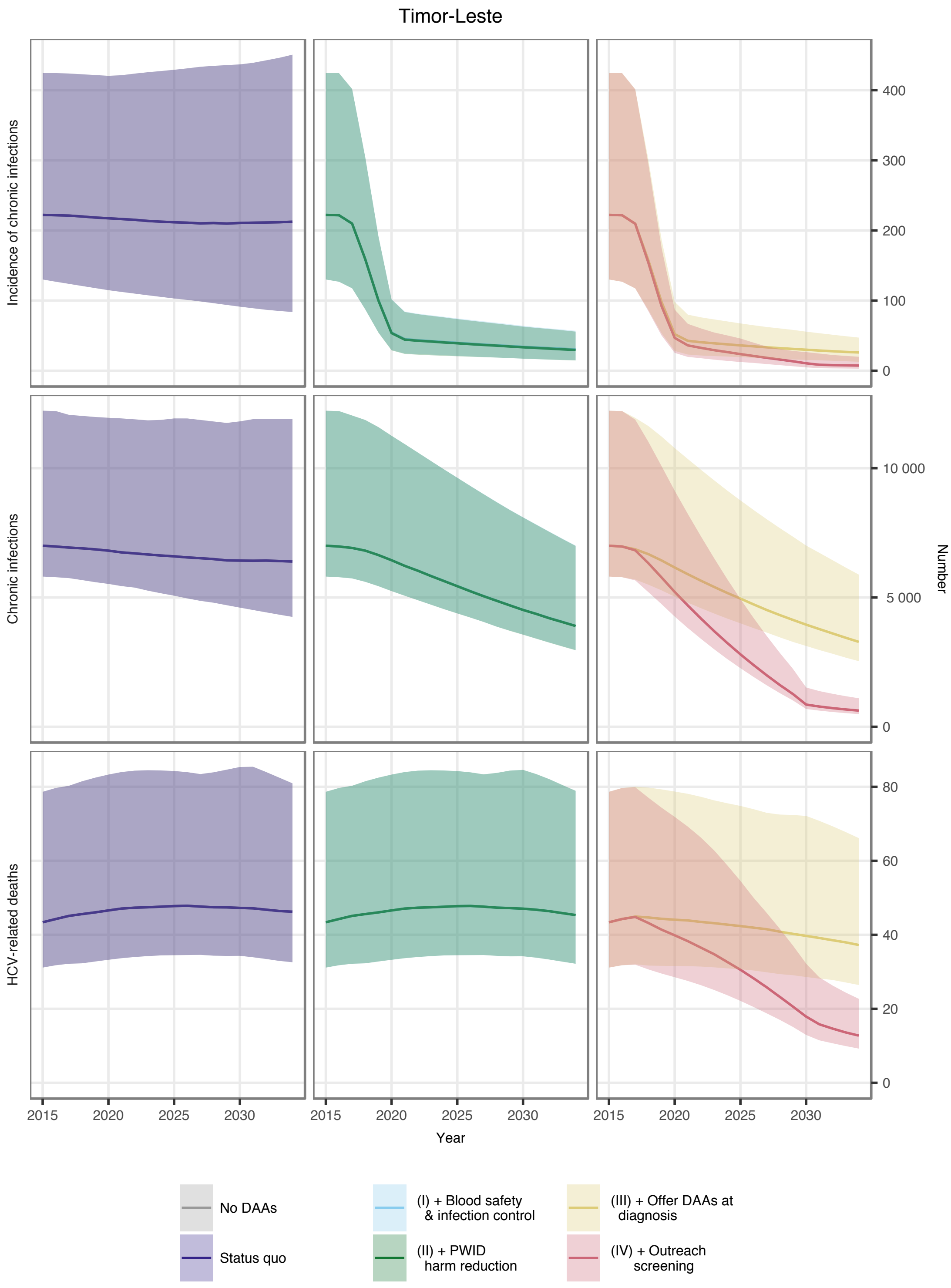

Timor-Leste
Incidence of chronic infections
Chronic infections
HCV-related deaths
Number
Year
0
100
200
300
400
0
5 000
10 000
0
20
40
60
80
2015
2020
2025
2030
No DAAs
Status quo
(I) + Blood safety & infection control
(II) + PWID harm reduction
(III) + Offer DAAs at diagnosis
(IV) + Outreach screening

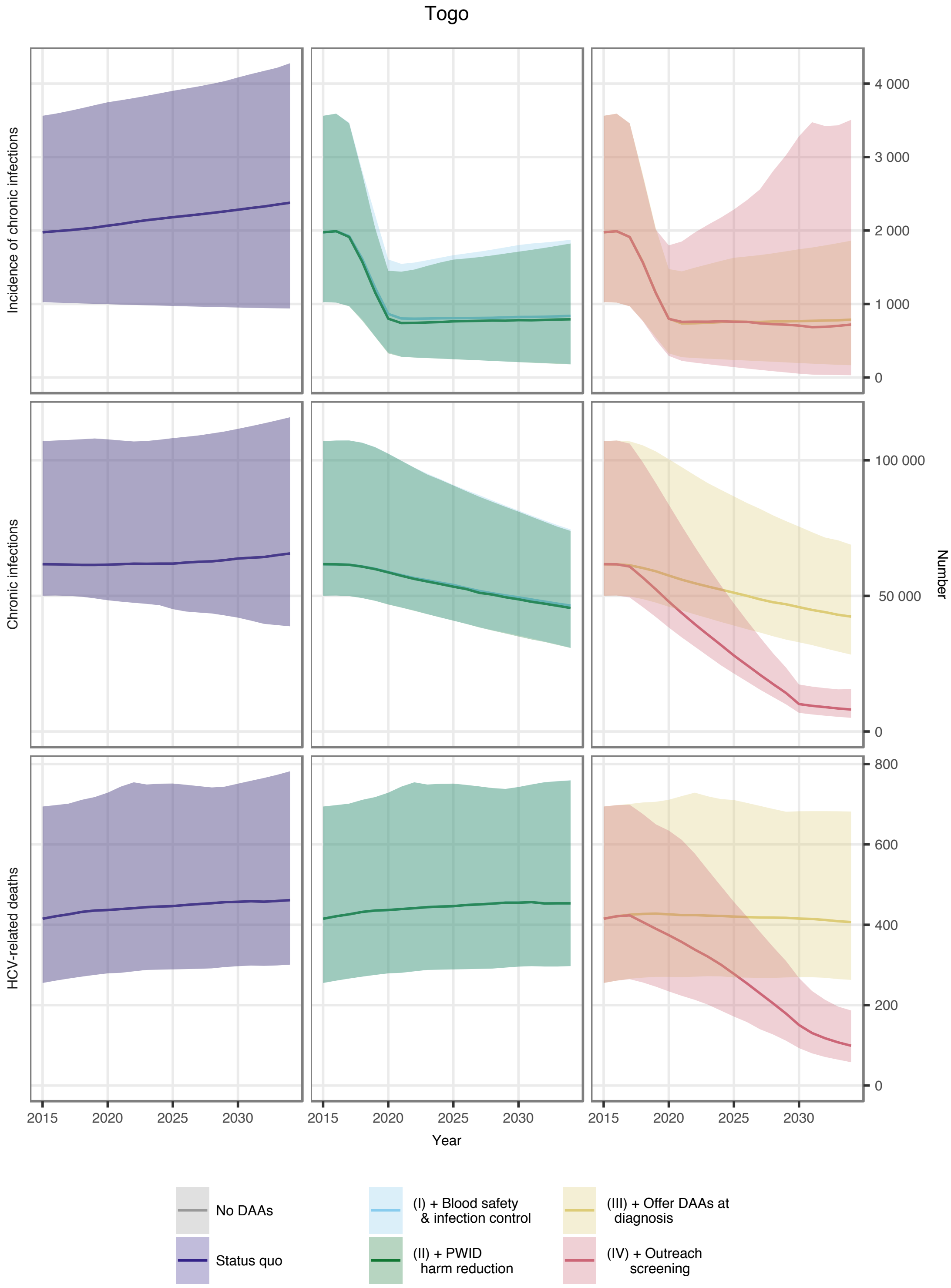
Togo
Incidence of chronic infections
Chronic infections
HCV-related deaths
Number
4 000
3 000
2 000
1 000
0
100 000
50 000
0
800
600
400
200
0
2015
2020
2025
2030
Year
No DAAs
Status quo
(I) + Blood safety & infection control
(II) + PWID harm reduction
(III) + Offer DAAs at diagnosis
(IV) + Outreach screening

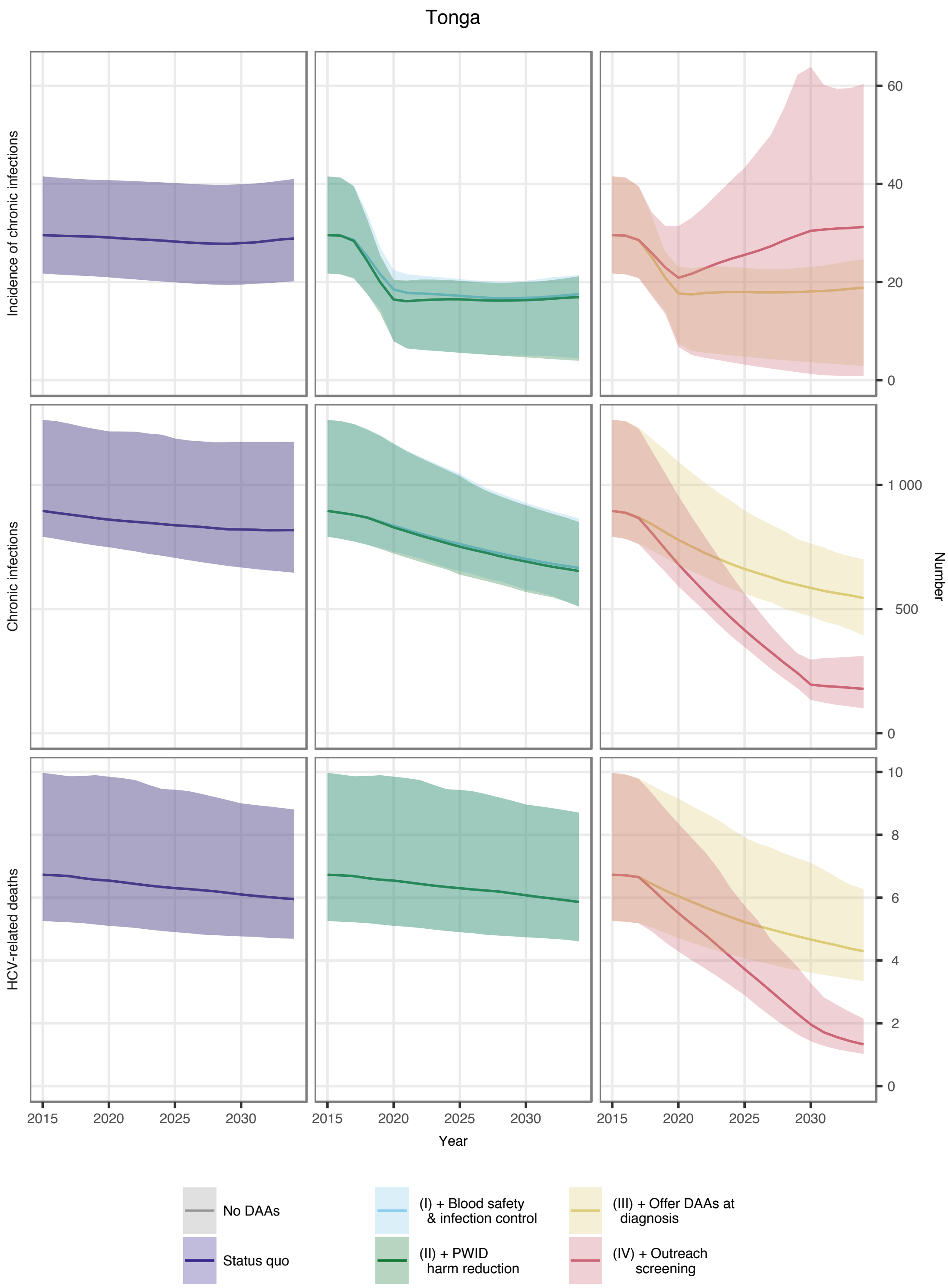
Tonga
Incidence of chronic infections
Chronic infections
HCV-related deaths
Number
Year
2015
2020
2025
2030
0
20
40
60
500
1 000
2
4
6
8
10
No DAAs
Status quo
(I) + Blood safety & infection control
(II) + PWID harm reduction
(III) + Offer DAAs at diagnosis
(IV) + Outreach screening

Trinidad and Tobago

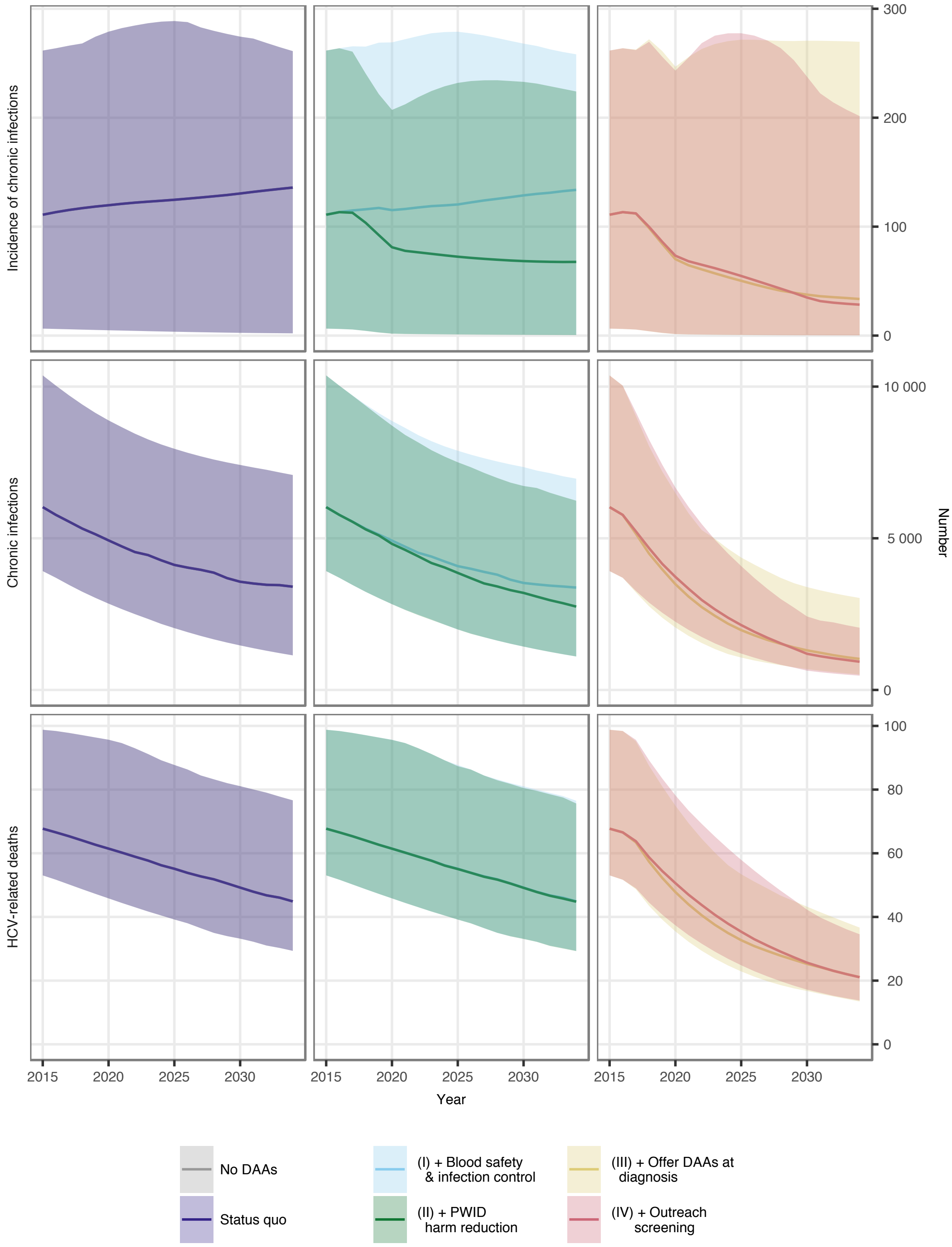

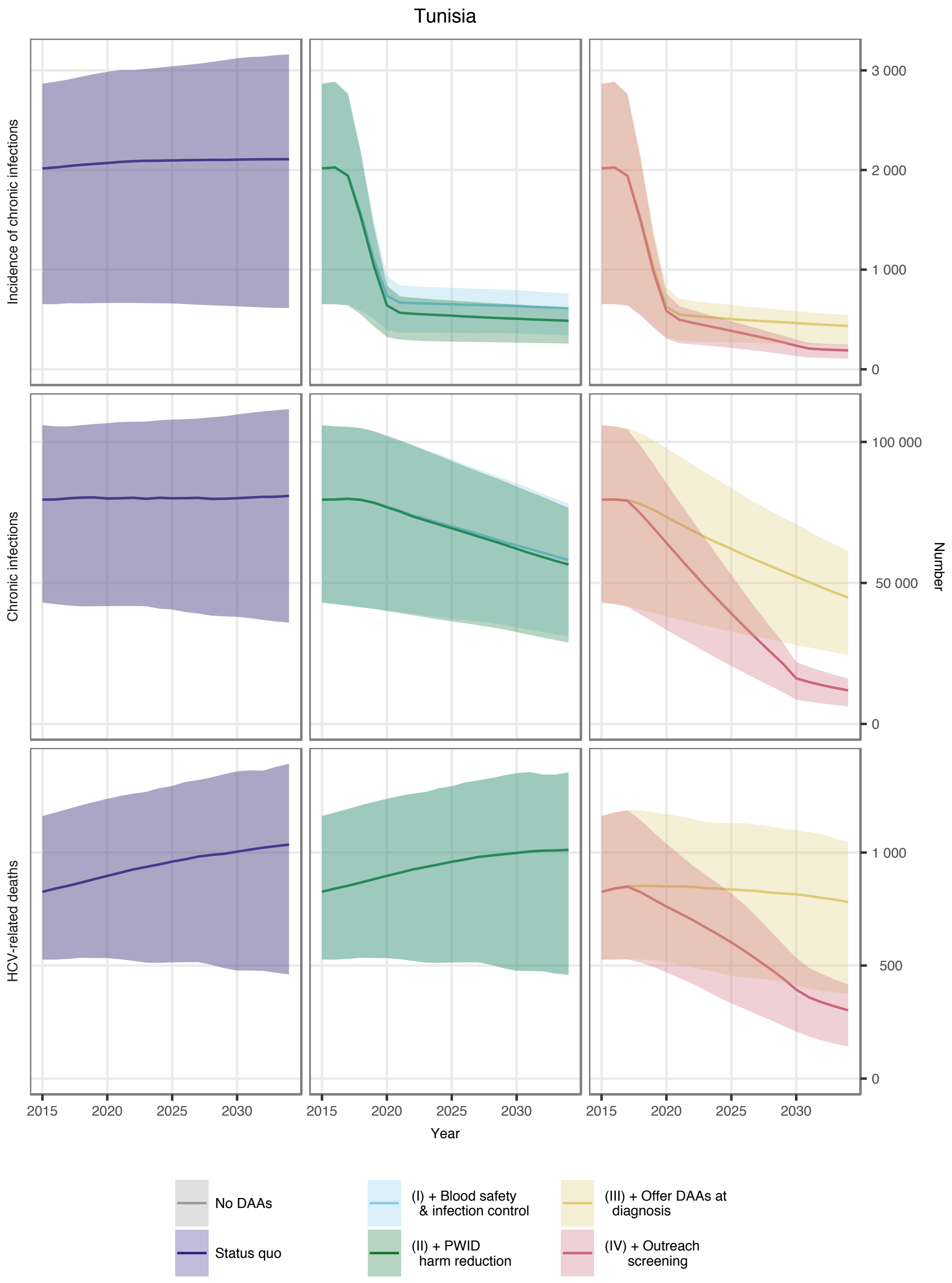
Tunisia
Incidence of chronic infections
Chronic infections
HCV-related deaths
Number
3 000
2 000
1 000
0
100 000
50 000
0
1 000
500
0
2015
2020
2025
2030
Year
No DAAs
Status quo
(I) + Blood safety & infection control
(II) + PWID harm reduction
(III) + Offer DAAs at diagnosis
(IV) + Outreach screening

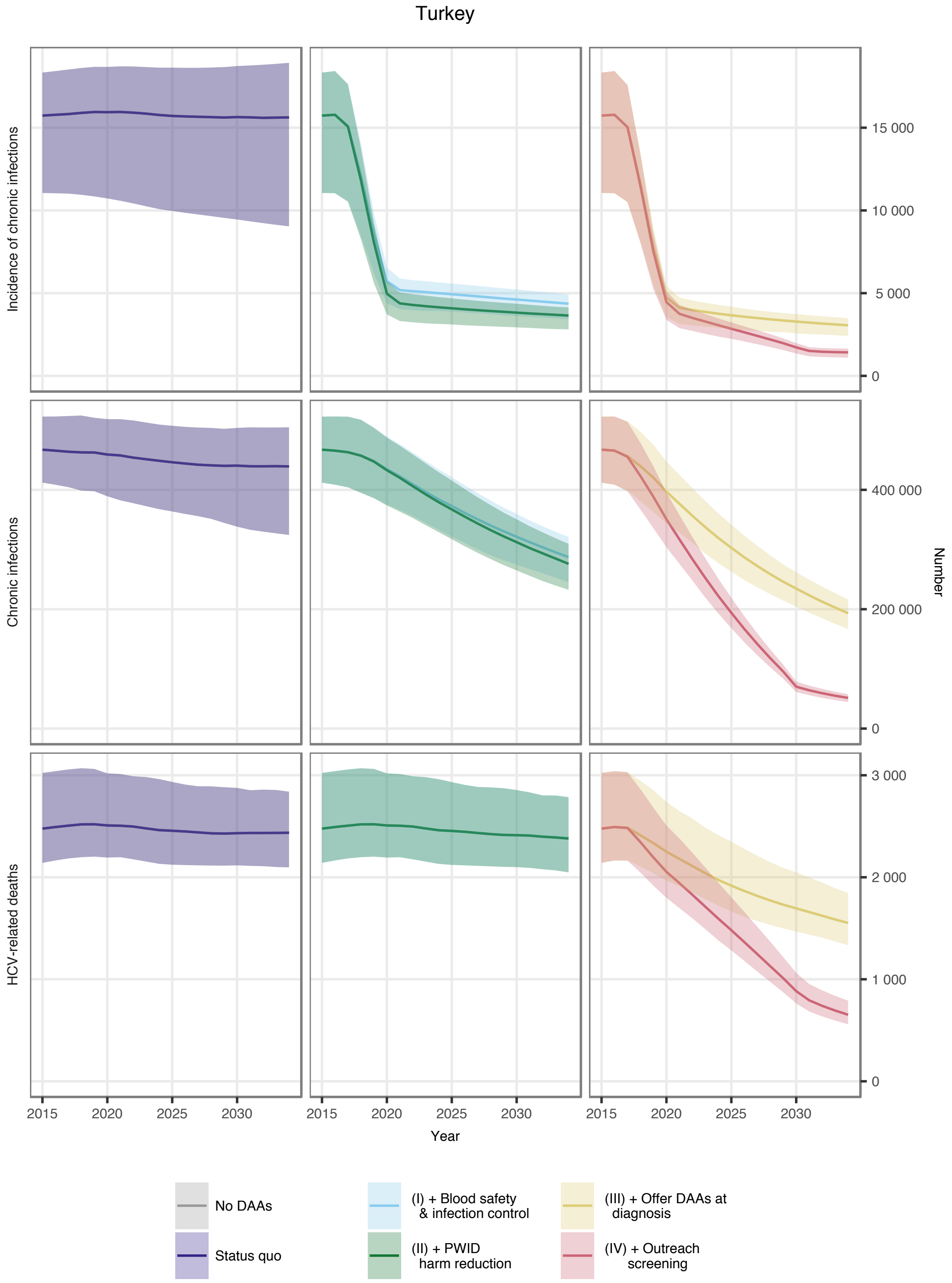
Turkey
Incidence of chronic infections
Chronic infections
HCV-related deaths
Number
15 000
10 000
5 000
0
400 000
200 000
0
3 000
2 000
1 000
0
2015
2020
2025
2030
Year
No DAAs
Status quo
(I) + Blood safety & infection control
(II) + PWID harm reduction
(III) + Offer DAAs at diagnosis
(IV) + Outreach screening

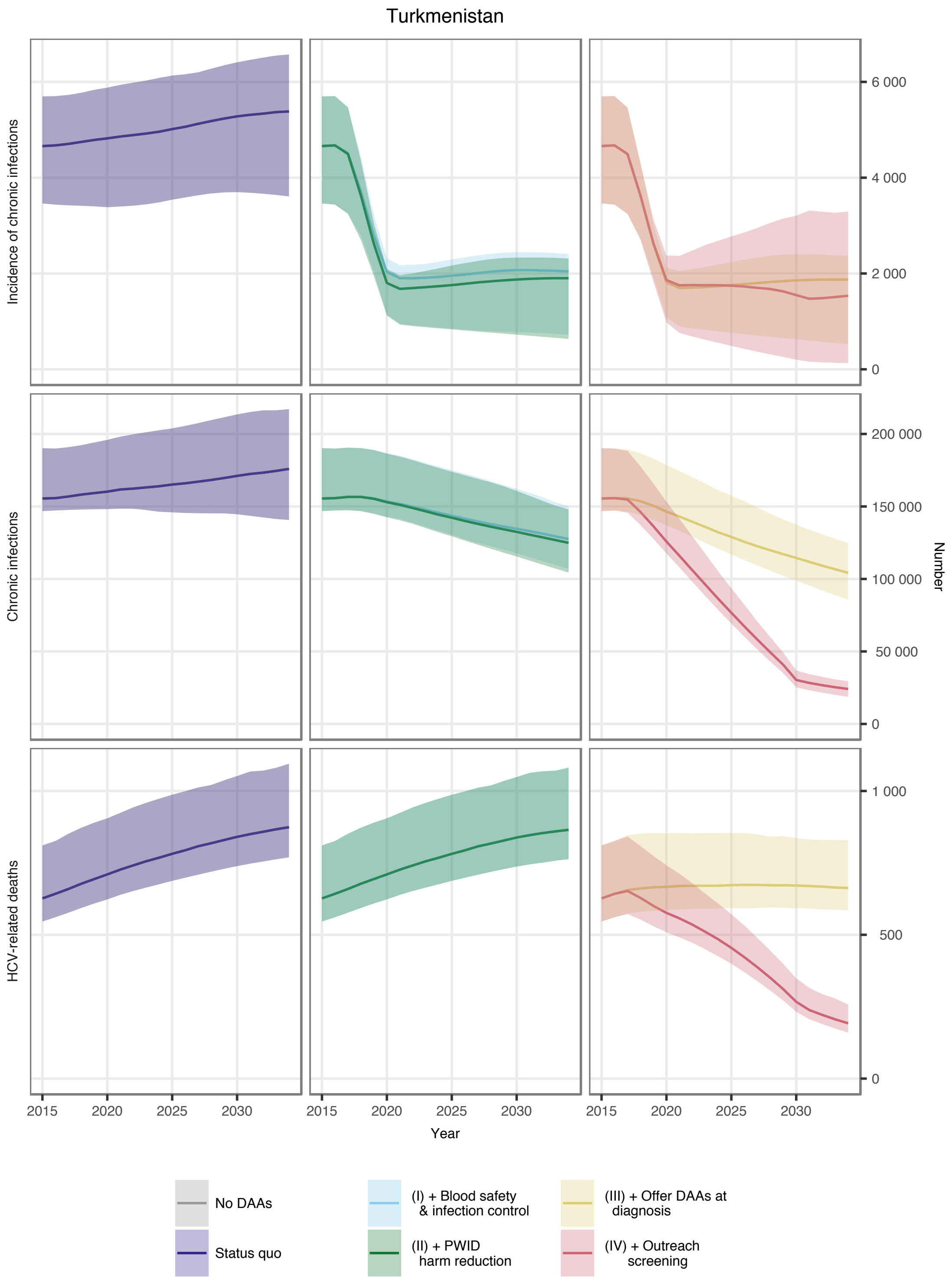

Turkmenistan
Incidence of chronic infections
Chronic infections
HCV-related deaths
Number
6 000
4 000
2 000
0
200 000
150 000
100 000
50 000
0
1 000
500
0
2015
2020
2025
2030
Year
No DAAs
Status quo
(I) + Blood safety & infection control
(II) + PWID harm reduction
(III) + Offer DAAs at diagnosis
(IV) + Outreach screening

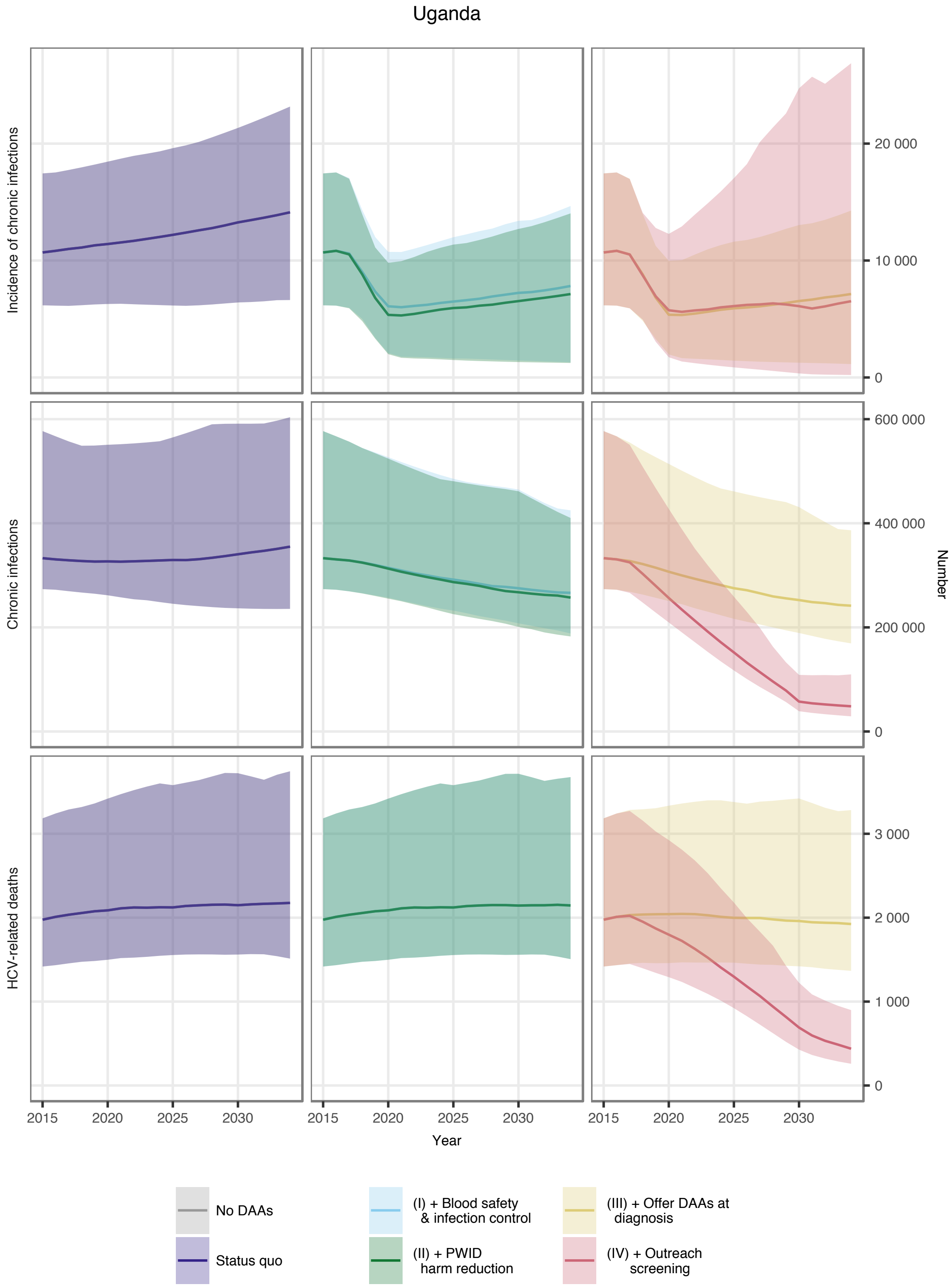
Uganda
Incidence of chronic infections
Chronic infections
HCV-related deaths
Number
Year
0
10 000
20 000
200 000
400 000
600 000
1 000
2 000
3 000
2015
2020
2025
2030
No DAAs
Status quo
(I) + Blood safety & infection control
(II) + PWID harm reduction
(III) + Offer DAAs at diagnosis
(IV) + Outreach screening

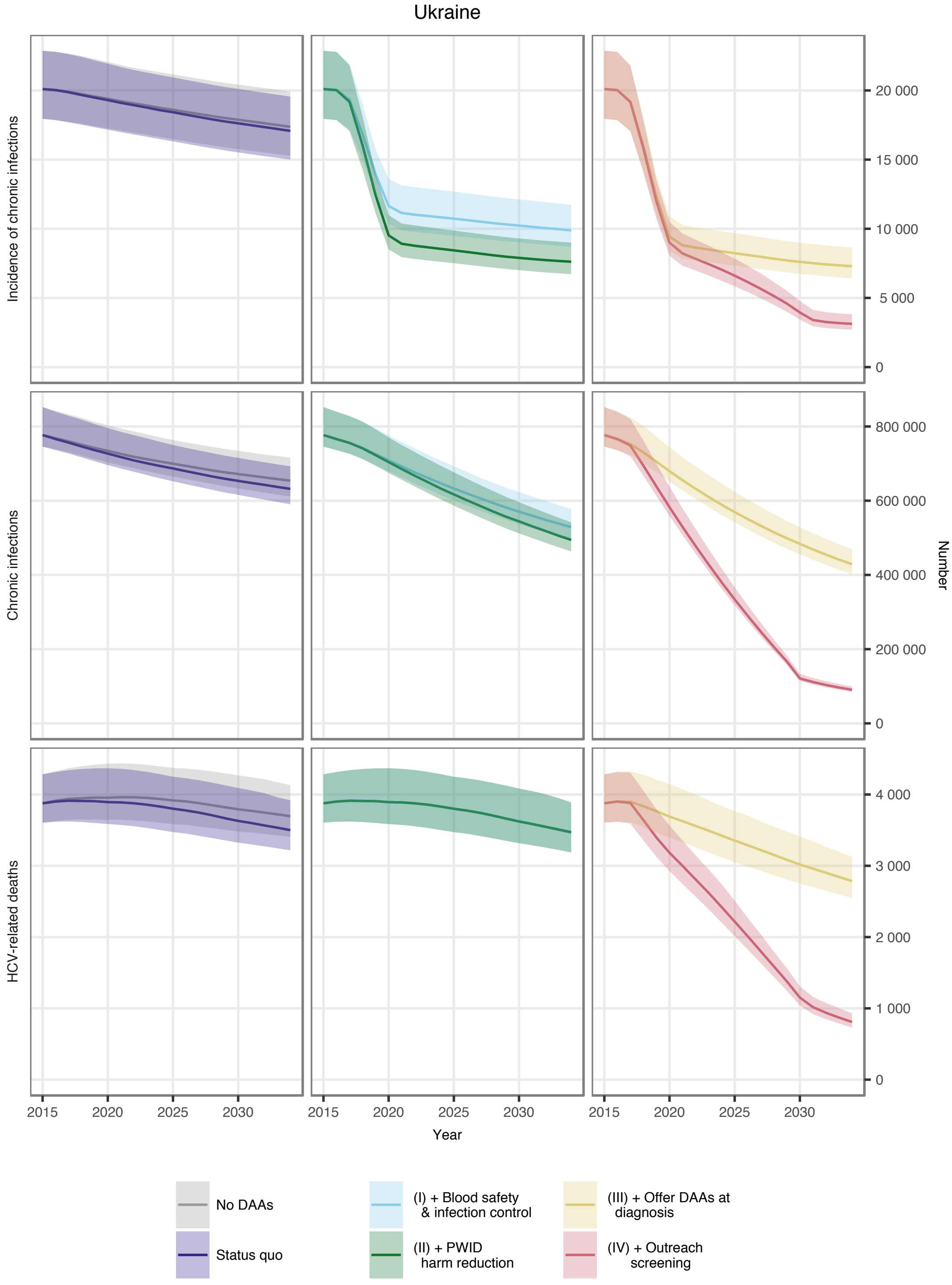
Ukraine
Incidence of chronic infections
Chronic infections
HCV-related deaths
Number
Year
20 000
15 000
10 000
5 000
0
800 000
600 000
400 000
200 000
0
4 000
3 000
2 000
1 000
0
2015
2020
2025
2030
No DAAs
Status quo
(I) + Blood safety & infection control
(II) + PWID harm reduction
(III) + Offer DAAs at diagnosis
(IV) + Outreach screening

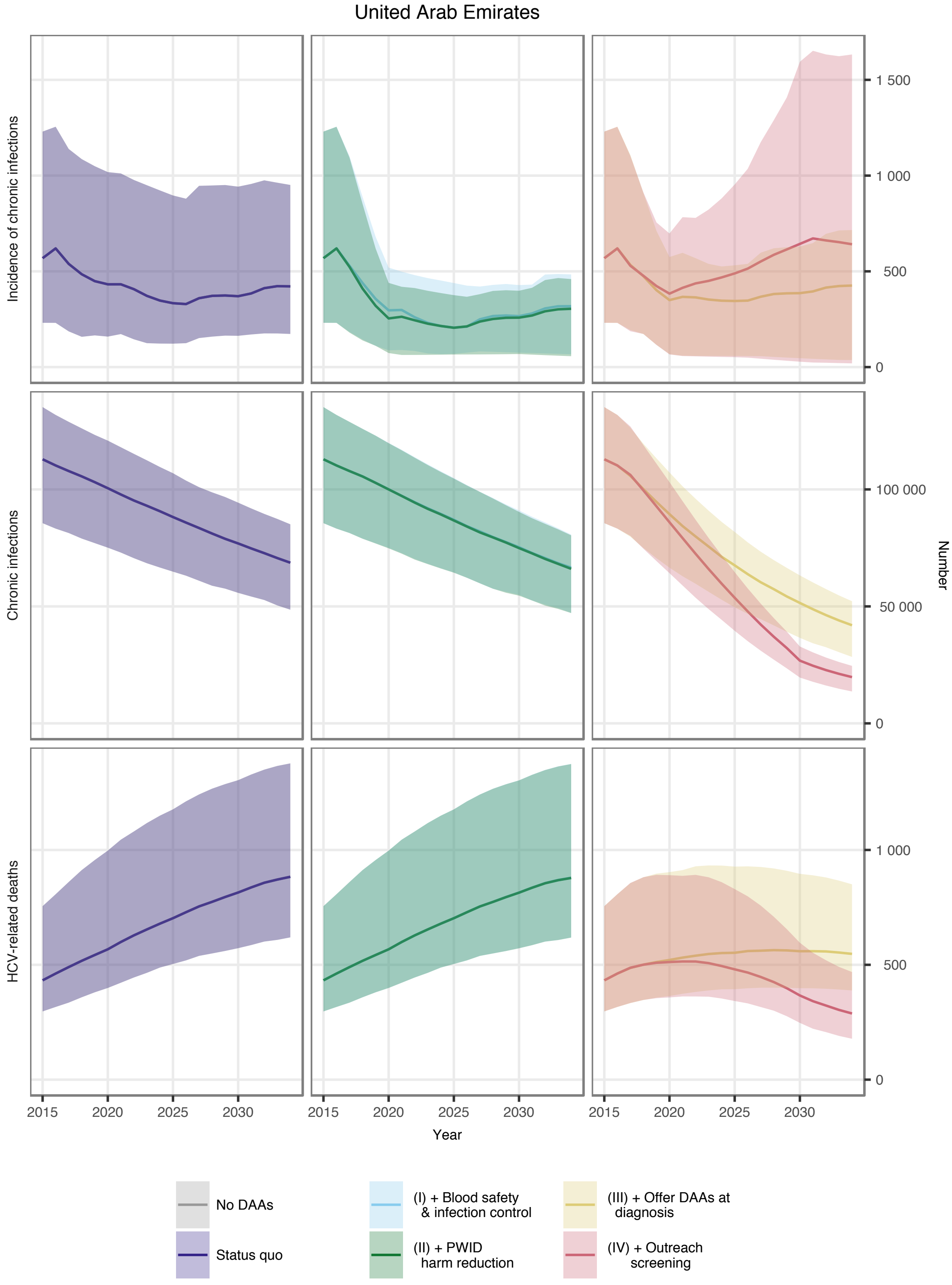
United Arab Emirates
Incidence of chronic infections
Chronic infections
HCV-related deaths
Number
Year
1 500
1 000
500
0
100 000
50 000
0
1 000
500
0
2015
2020
2025
2030
No DAAs
Status quo
(I) + Blood safety & infection control
(II) + PWID harm reduction
(III) + Offer DAAs at diagnosis
(IV) + Outreach screening

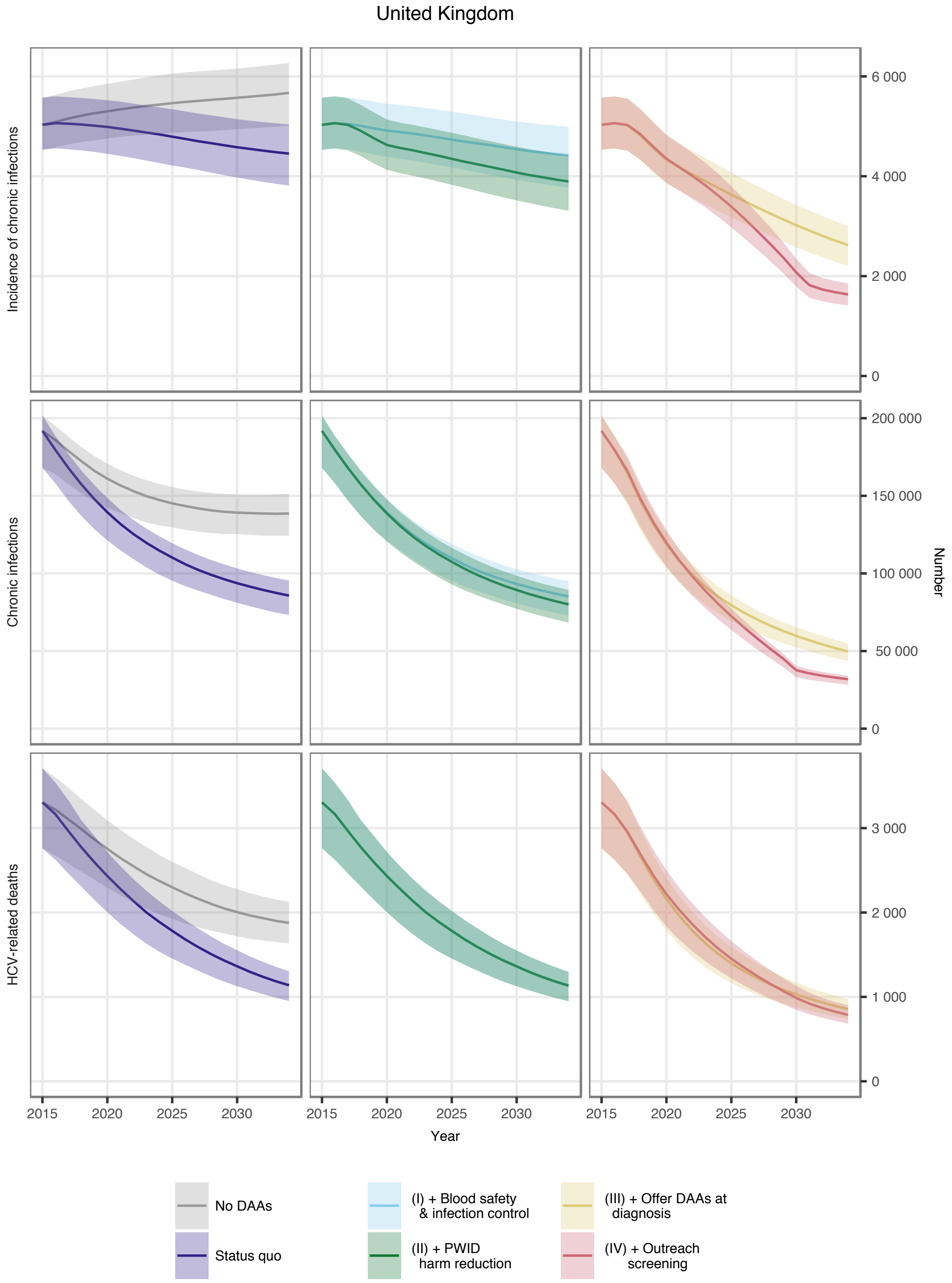
United Kingdom
Incidence of chronic infections
Chronic infections
HCV-related deaths
Number
Year
6 000
4 000
2 000
0
200 000
150 000
100 000
50 000
0
3 000
2 000
1 000
0
2015
2020
2025
2030
No DAAs
Status quo
(I) + Blood safety & infection control
(II) + PWID harm reduction
(III) + Offer DAAs at diagnosis
(IV) + Outreach screening

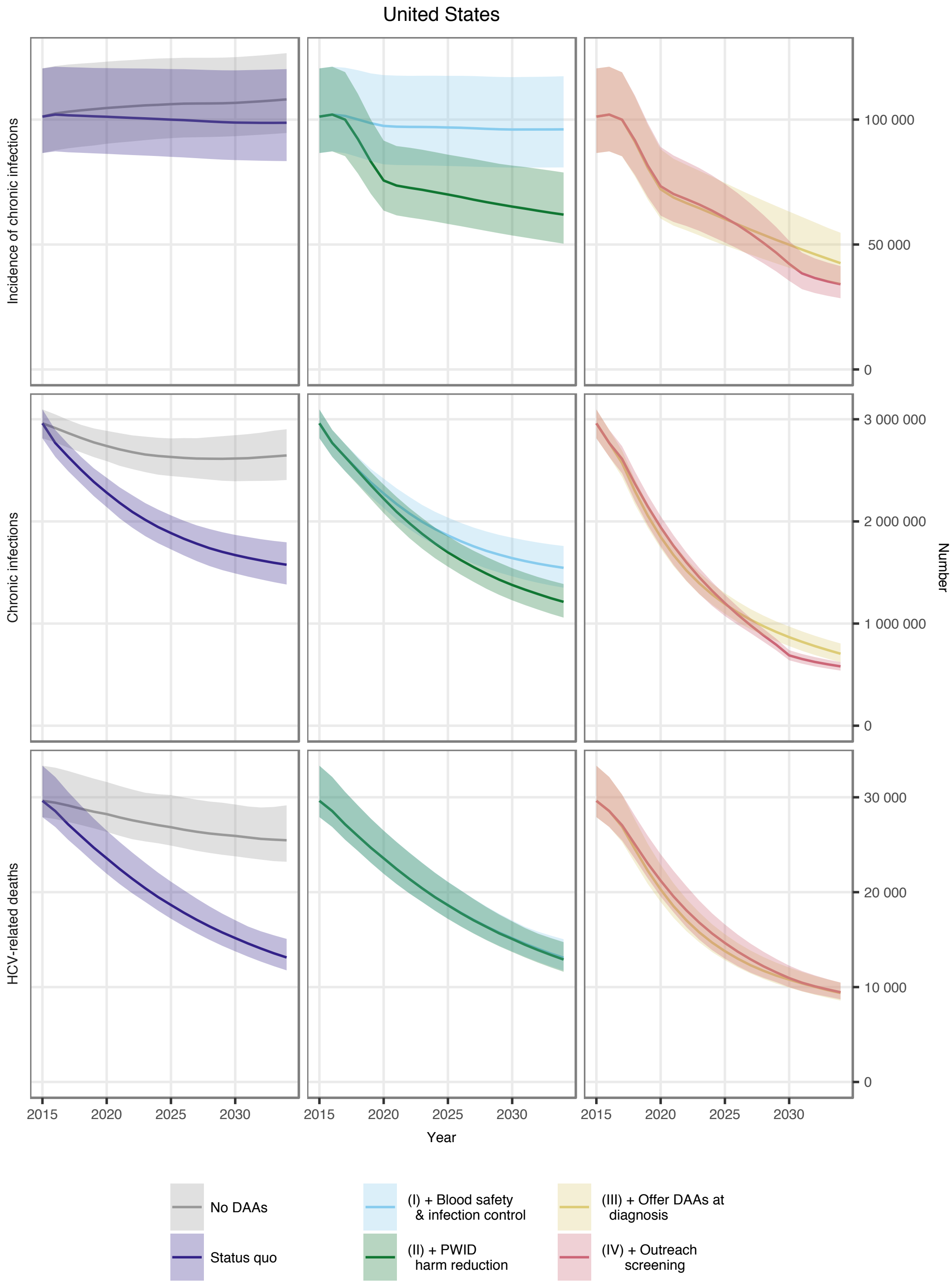
United States
Incidence of chronic infections
Chronic infections
HCV-related deaths
100 000
50 000
0
3 000 000
2 000 000
1 000 000
0
30 000
20 000
10 000
0
Number
2015
2020
2025
2030
Year
No DAAs
Status quo
(I) + Blood safety & infection control
(II) + PWID harm reduction
(III) + Offer DAAs at diagnosis
(IV) + Outreach screening

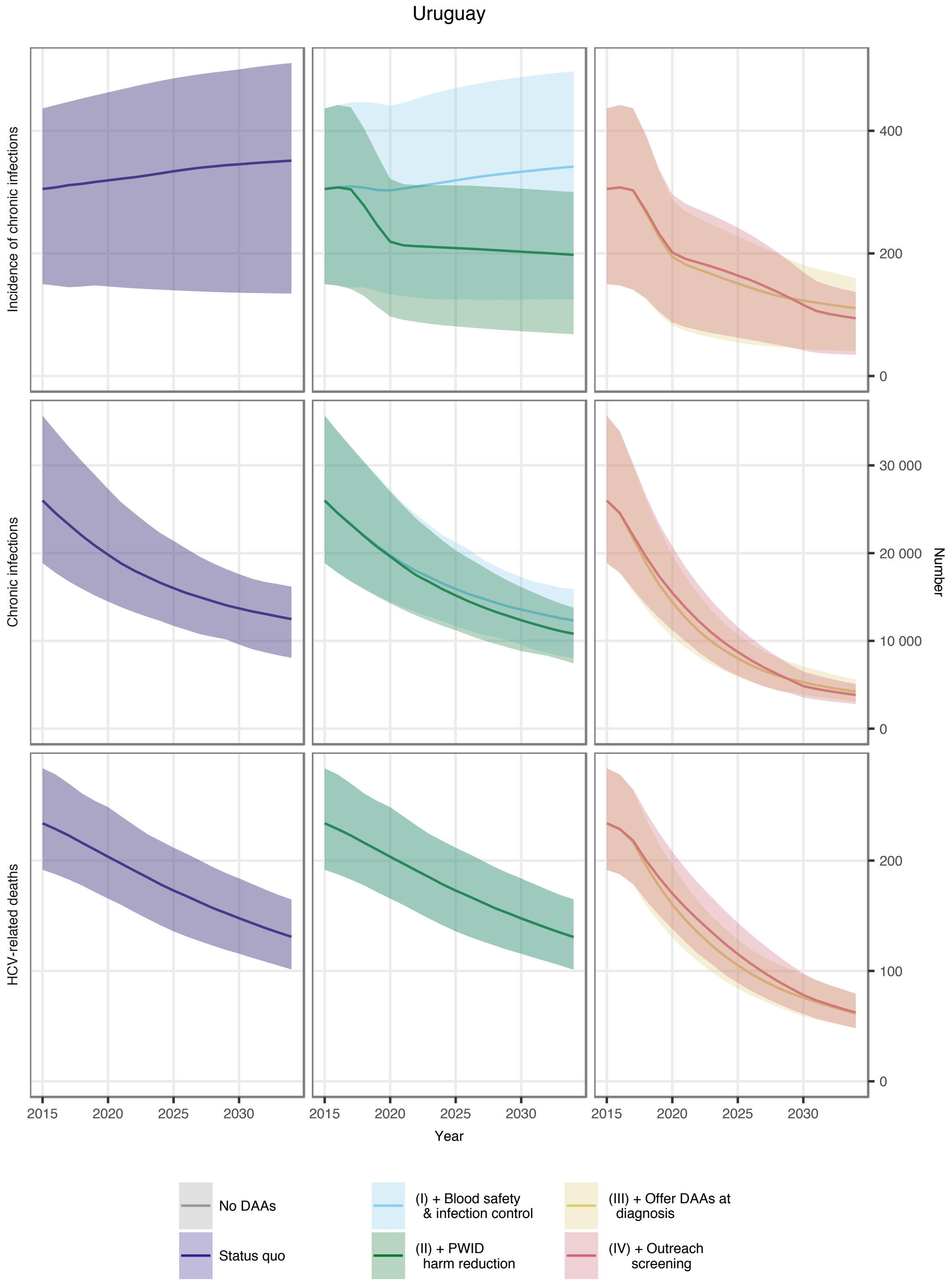

Uruguay
Incidence of chronic infections
Chronic infections
HCV-related deaths
Number
Year
0
200
400
10 000
20 000
30 000
100
2015
2020
2025
2030
No DAAs
Status quo
(I) + Blood safety & infection control
(II) + PWID harm reduction
(III) + Offer DAAs at diagnosis
(IV) + Outreach screening

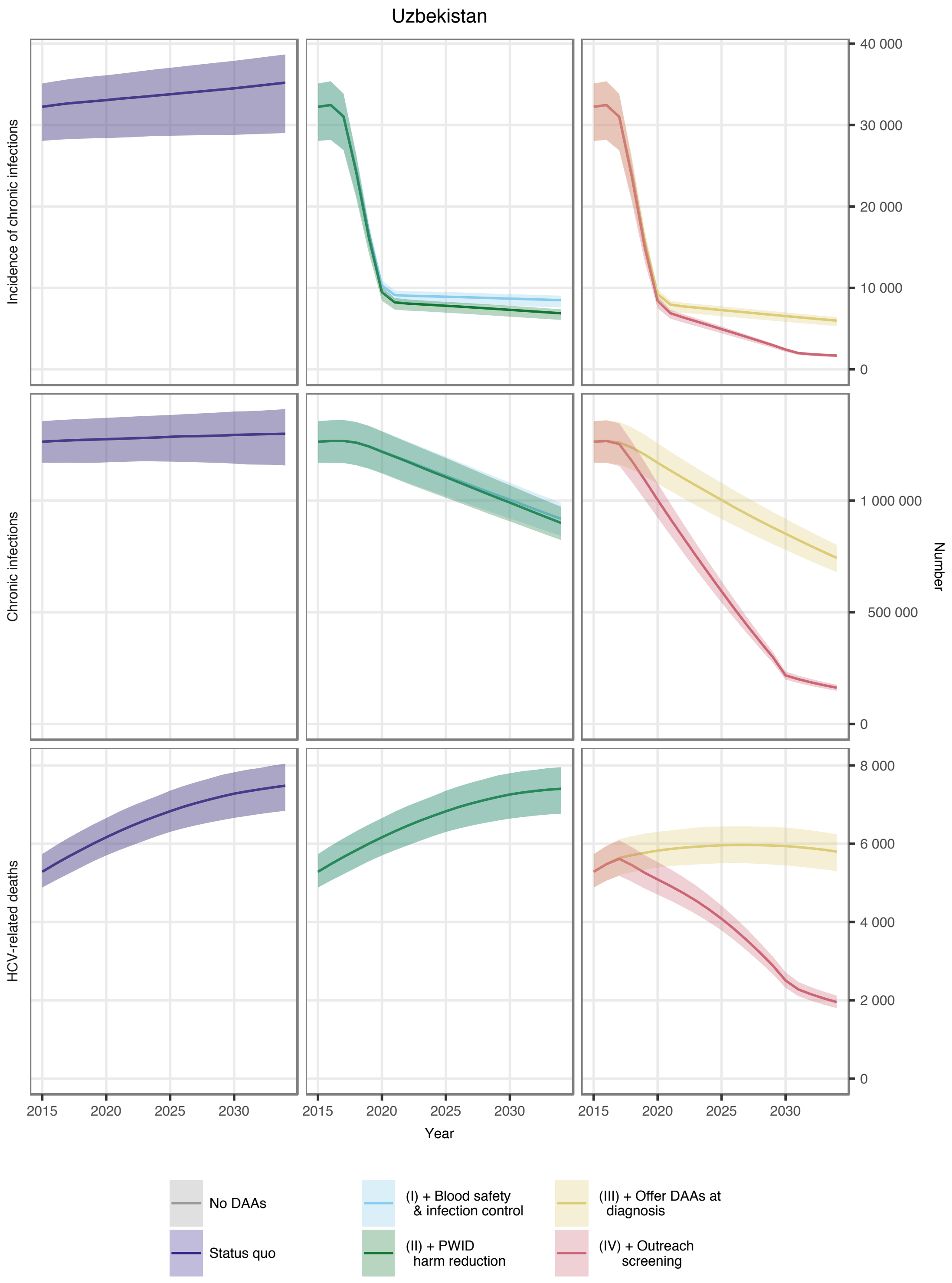

Uzbekistan
Incidence of chronic infections
Chronic infections
HCV-related deaths
Number
40 000
30 000
20 000
10 000
0
1 000 000
500 000
0
8 000
6 000
4 000
2 000
0
2015
2020
2025
2030
Year
No DAAs
Status quo
(I) + Blood safety & infection control
(II) + PWID harm reduction
(III) + Offer DAAs at diagnosis
(IV) + Outreach screening

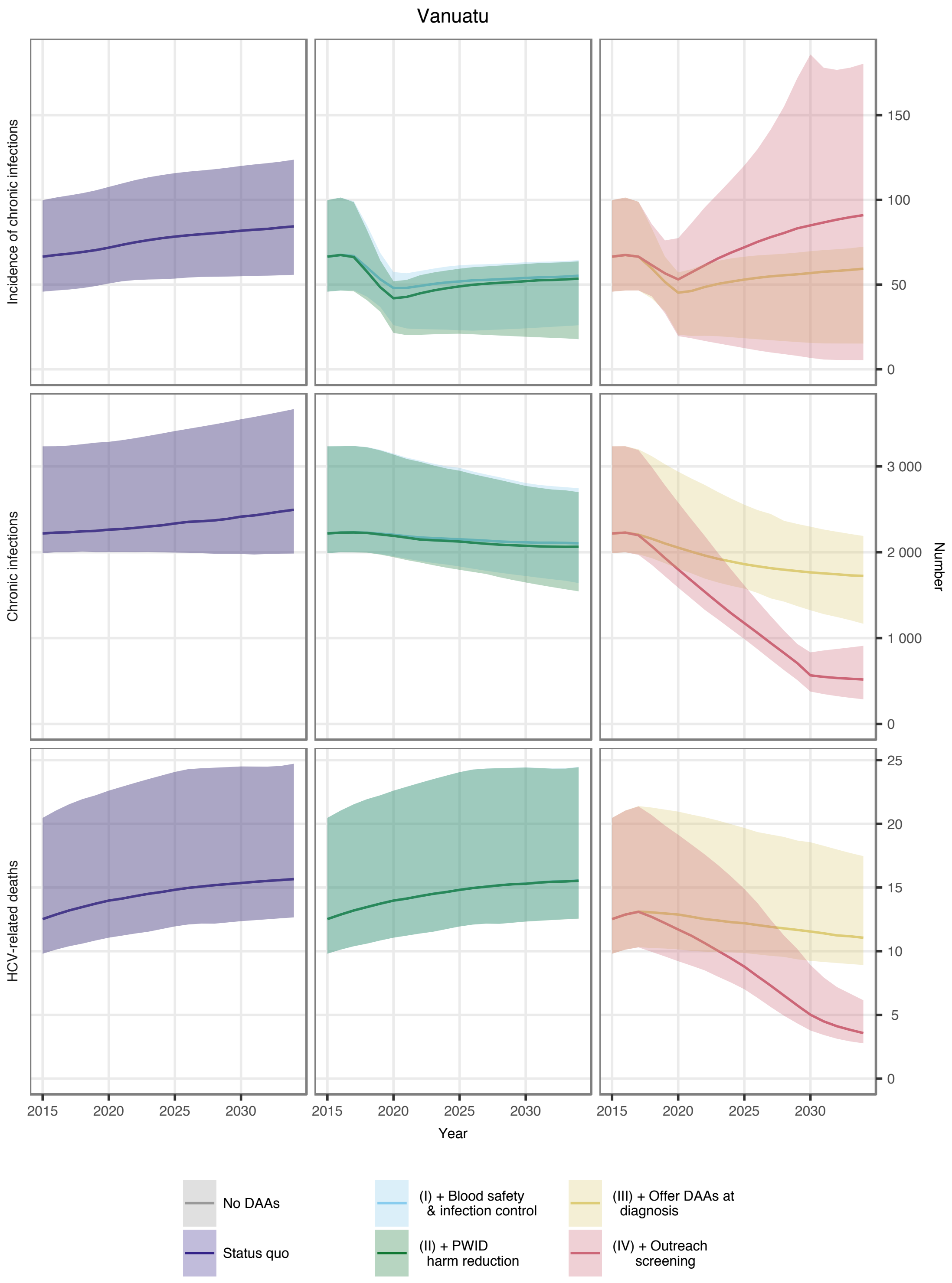
Vanuatu
Incidence of chronic infections
Chronic infections
HCV-related deaths
Number
Year
2015
2020
2025
2030
0
50
100
150
0
1 000
2 000
3 000
0
5
10
15
20
25
No DAAs
Status quo
(I) + Blood safety & infection control
(II) + PWID harm reduction
(III) + Offer DAAs at diagnosis
(IV) + Outreach screening

Venezuela, Bolivarian Republic of

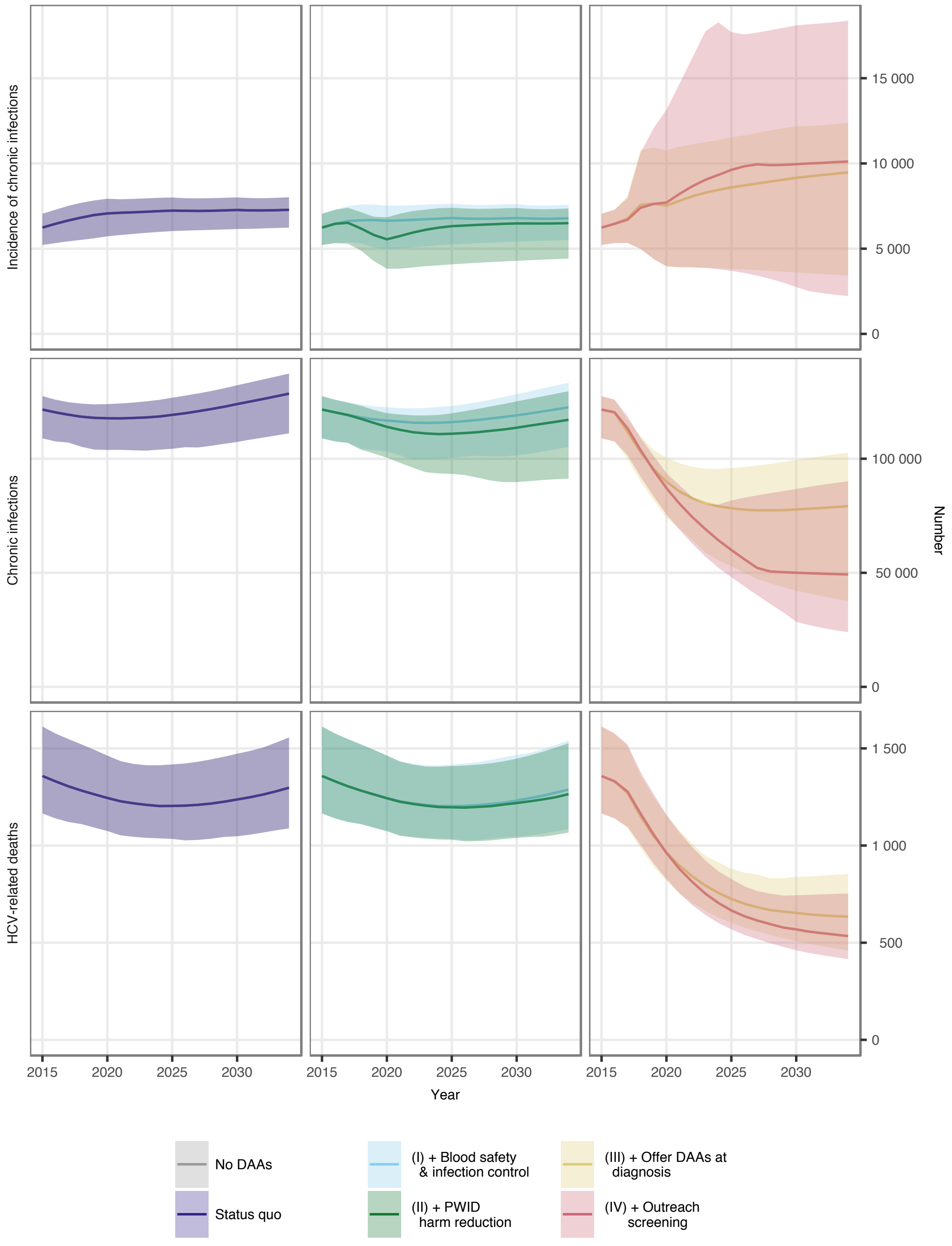

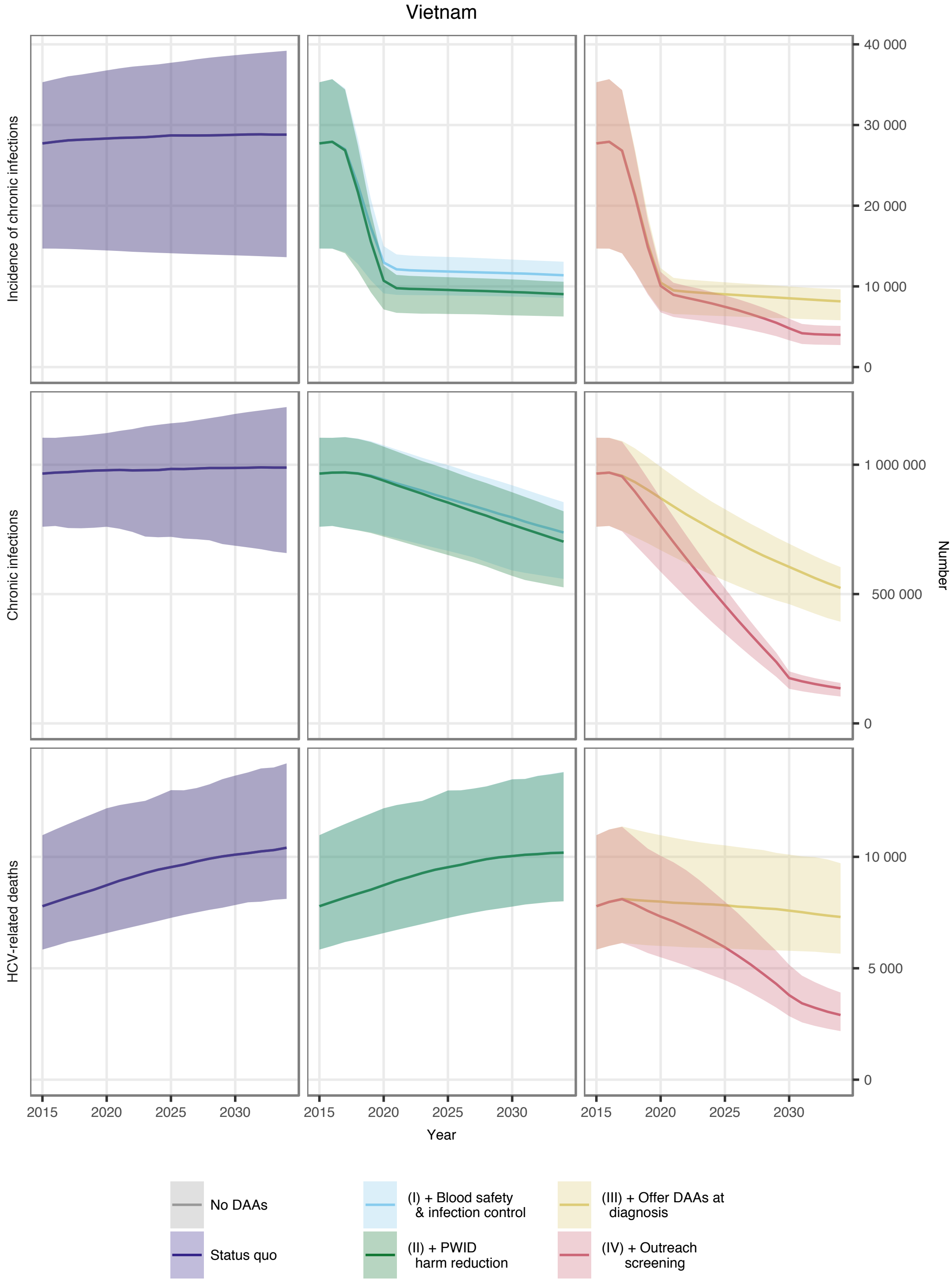
Vietnam
Incidence of chronic infections
Chronic infections
HCV-related deaths
Number
40 000
30 000
20 000
10 000
0
1 000 000
500 000
0
10 000
5 000
0
2015
2020
2025
2030
Year
No DAAs
Status quo
(I) + Blood safety & infection control
(II) + PWID harm reduction
(III) + Offer DAAs at diagnosis
(IV) + Outreach screening

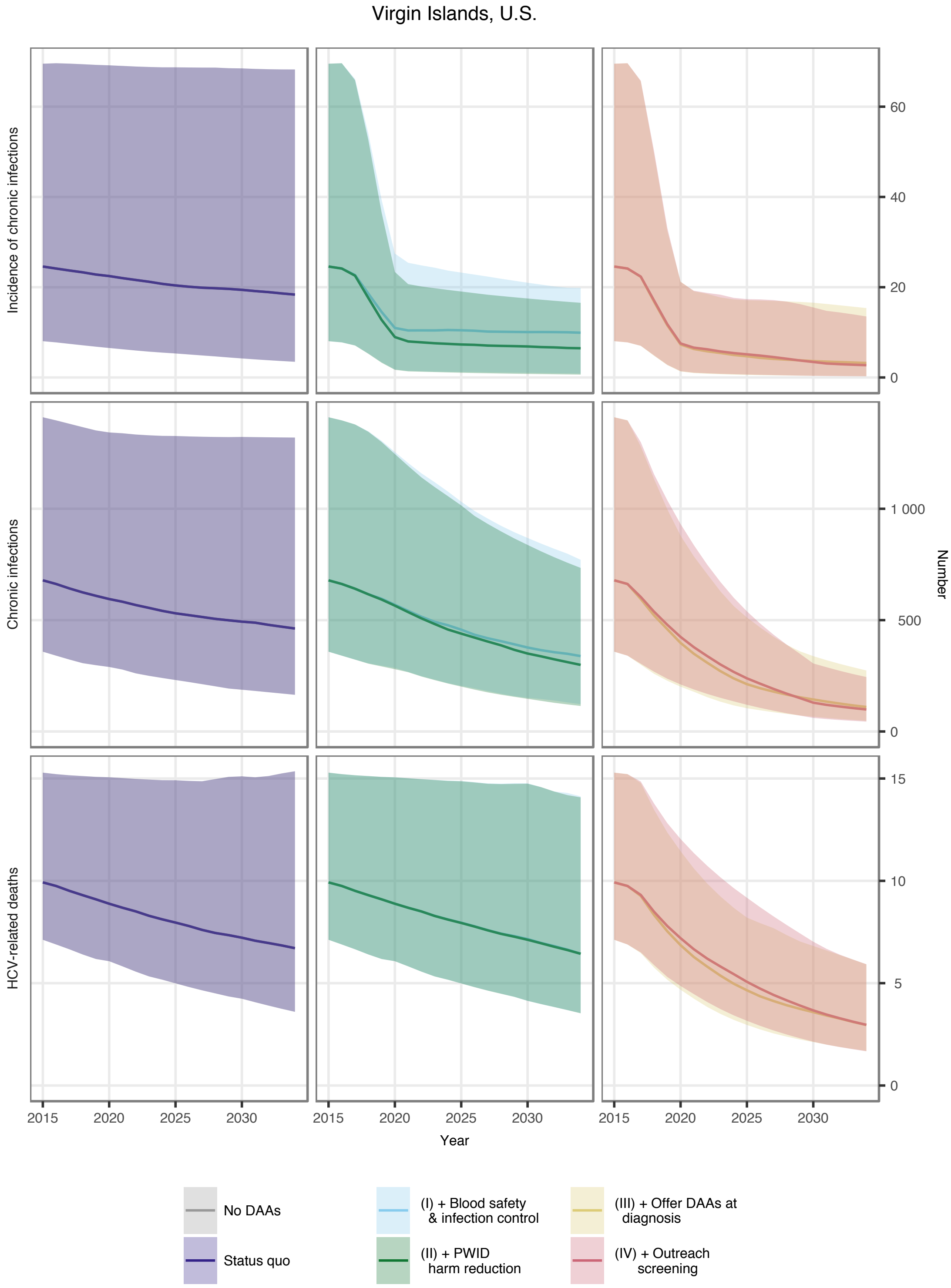

Virgin Islands, U.S.
Incidence of chronic infections
Chronic infections
HCV-related deaths
Number
Year
0
20
40
60
0
500
1 000
0
5
10
15
2015
2020
2025
2030
No DAAs
Status quo
(I) + Blood safety & infection control
(II) + PWID harm reduction
(III) + Offer DAAs at diagnosis
(IV) + Outreach screening

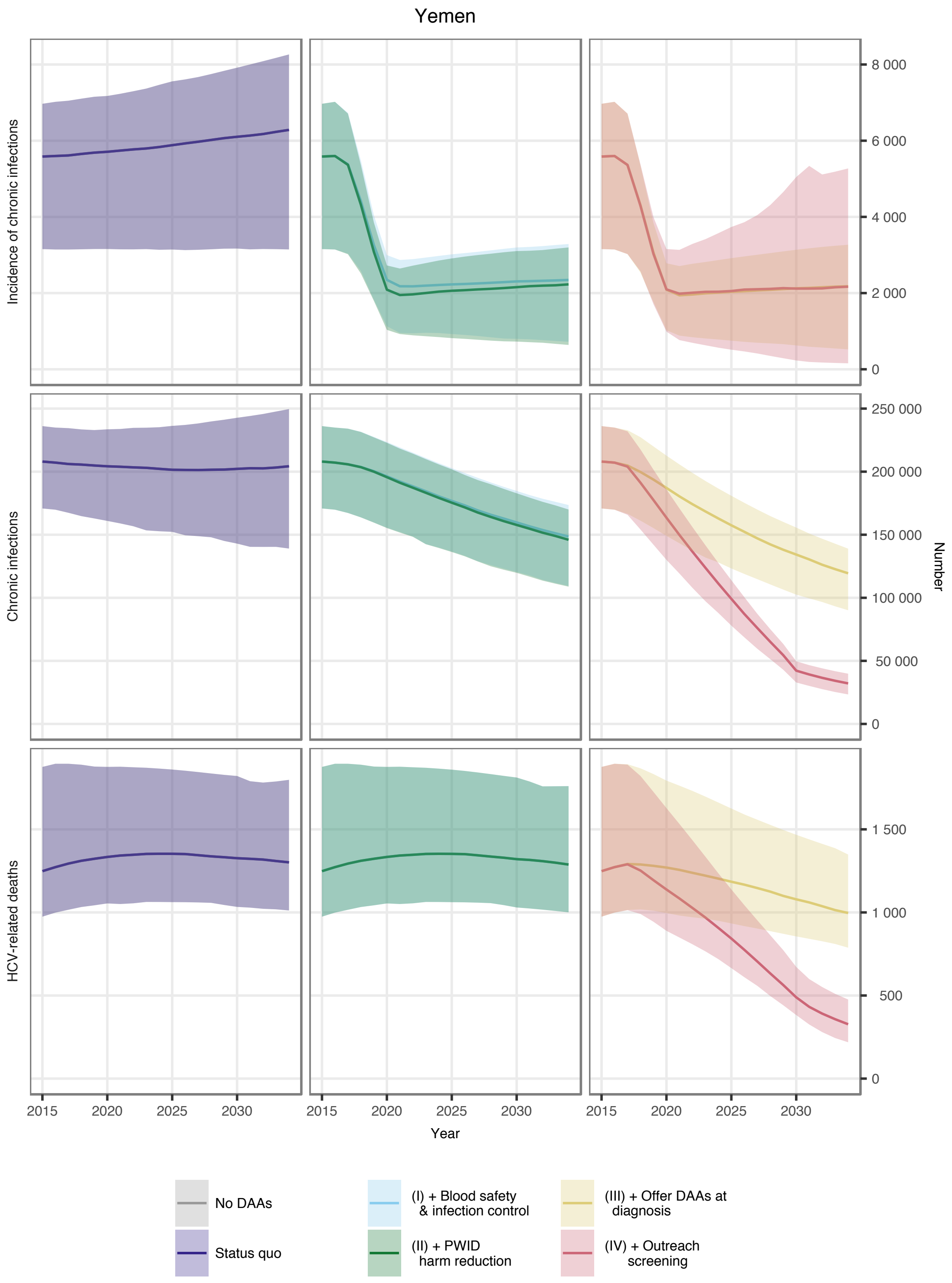
Yemen
Incidence of chronic infections
Chronic infections
HCV-related deaths
Number
8 000
6 000
4 000
2 000
0
250 000
200 000
150 000
100 000
50 000
0
1 500
1 000
500
0
2015
2020
2025
2030
Year
No DAAs
Status quo
(I) + Blood safety & infection control
(II) + PWID harm reduction
(III) + Offer DAAs at diagnosis
(IV) + Outreach screening

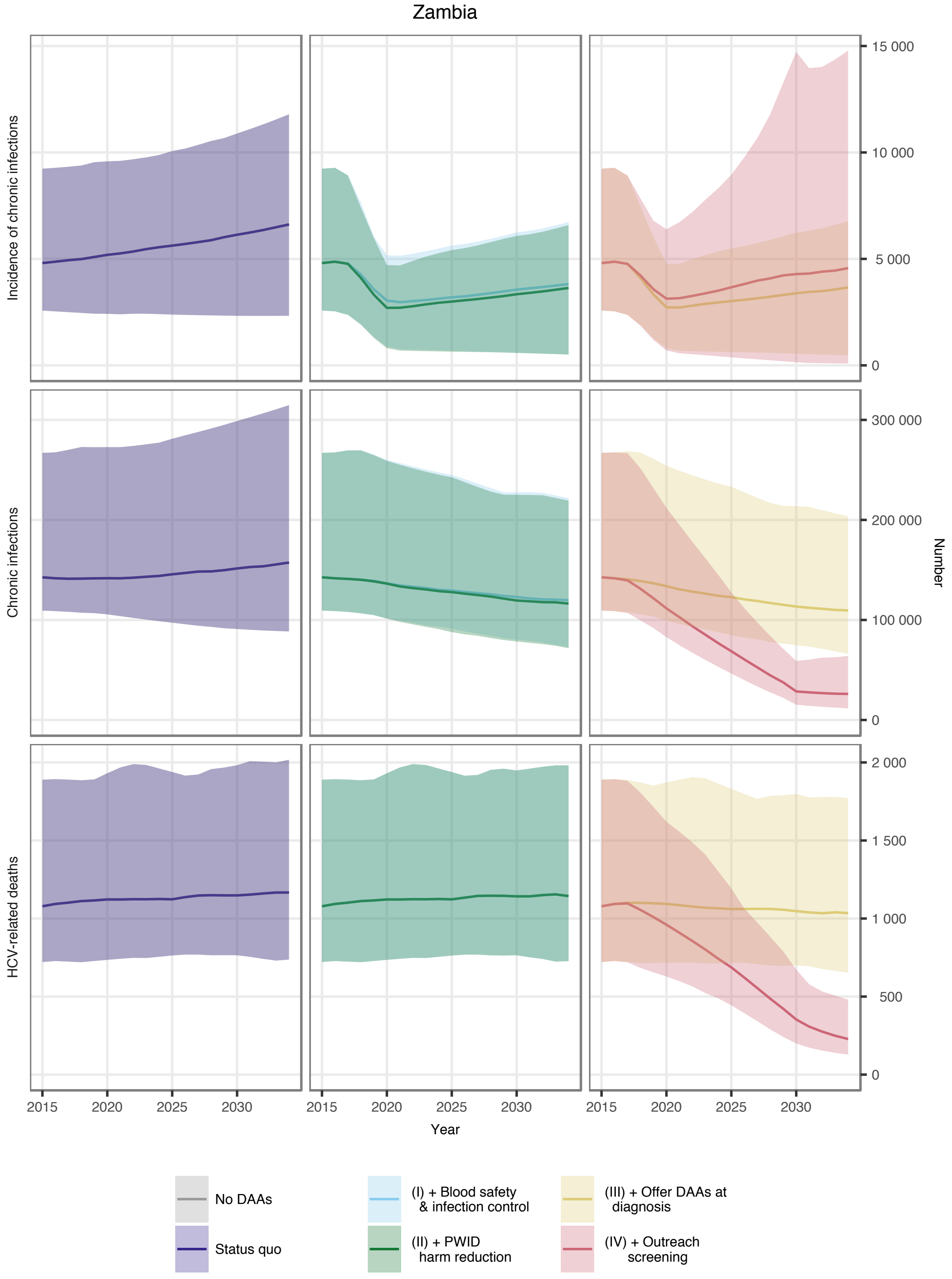
Zambia
Incidence of chronic infections
Chronic infections
HCV-related deaths
Number
Year
15 000
10 000
5 000
0
300 000
200 000
100 000
0
2 000
1 500
1 000
500
0
2015
2020
2025
2030
No DAAs
Status quo
(I) + Blood safety & infection control
(II) + PWID harm reduction
(III) + Offer DAAs at diagnosis
(IV) + Outreach screening

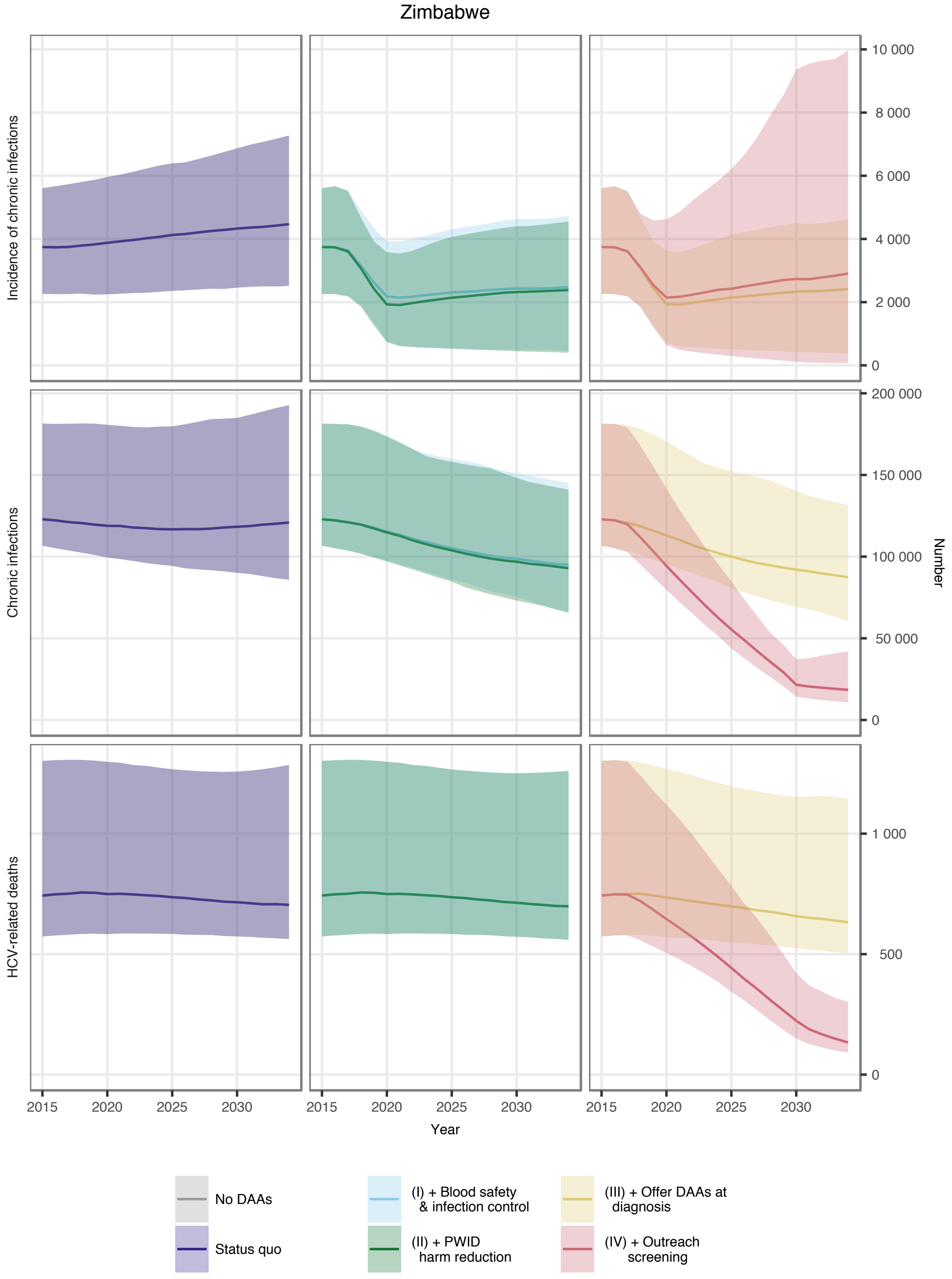

Zimbabwe
Incidence of chronic infections
Chronic infections
HCV-related deaths
Number
Year
10 000
8 000
6 000
4 000
2 000
0
200 000
150 000
100 000
50 000
0
1 000
500
0
2015
2020
2025
2030
No DAAs
Status quo
(I) + Blood safety & infection control
(II) + PWID harm reduction
(III) + Offer DAAs at diagnosis
(IV) + Outreach screening

References

1 Chen SL, Morgan TR. The natural history of hepatitis C virus (HCV) infection. *Int J Med Sci* 2006; **3**: 47–52.

2 Seeff LB. Natural history of chronic hepatitis C. *Hepatology* 2002; **36**: s35–46.

3 Westbrook RH, Dusheiko G. Natural history of hepatitis C. *J Hepatol* 2014; **61**: s58–68.

4 Messina JP, Humphreys I, Flaxman A, *et al.* Global distribution and prevalence of hepatitis C virus genotypes. *Hepatology* 2015; **61**: 77–87.

5 Bedossa P. Intraobserver and interobserver variations in liver biopsy interpretation in patients with chronic hepatitis C. *Hepatology* 1994; **20**: 15–20.

6 Alter MJ. Epidemiology of hepatitis C virus infection. *World J Gastroenterol* 2007; **13**: 2436.

7 Shepard CW, Finelli L, Alter MJ. Global epidemiology of hepatitis C virus infection. *Lancet Infect Dis* 2005; **5**: 558–567.

8 Mathers BM, Degenhardt L, Phillips B, *et al.* Global epidemiology of injecting drug use and HIV among people who inject drugs: a systematic review. *Lancet* 2008; **372**: 1733–45.

9 Martin NK, Vickerman P, Grebely J, *et al.* Hepatitis C virus treatment for prevention among people who inject drugs: modeling treatment scale-up in the age of direct-acting antivirals. *Hepatology* 2013; **58**: 1598–609.

10 Horyniak D, Dietze P, Degenhardt L, *et al.* The relationship between age and risky injecting behaviours among a sample of Australian people who inject drugs. *Drug Alcohol Depend* 2013; **132**: 541–6.

11 Browne R, Asboe D, Gilleece Y, *et al.* Increased numbers of acute hepatitis C infections in HIV positive homosexual men; is sexual transmission feeding the increase? *Sex Transm Infect* 2004; **80**: 326–7.

12 Danta M, Brown D, Bhagani S, *et al.* Recent epidemic of acute hepatitis C virus in HIV-positive men who have sex with men linked to high-risk sexual behaviours. *Aids* 2007; **21**: 983–991.

13 Bradshaw D, Matthews G, Danta M. Sexually transmitted hepatitis C infection: the new epidemic in MSM? *Curr Opin Infect Dis* 2012; **26**: 66–72.

14 Jordan AE, Perlman DC, Neurer J, Smith DJ, Des Jarlais DC, Hagan H. Prevalence of hepatitis C virus infection among HIV+ men who have sex with men: a systematic review and meta-analysis. *Int J STD AIDS* 2017; **28**: 145–59.

15 The Polaris Observatory HCV Collaborators. Global prevalence and genotype distribution of hepatitis C virus infection in 2015: a modelling study. *Lancet Gastroenterol Hepatol* 2017; **2**: 161–176.

16 Vandelli C, Renzo F, Romanò L, *et al.* Lack of evidence of sexual transmission of hepatitis C among monogamous couples: results of a 10-year prospective follow-up study. *Am J Gastroenterol* 2004; **99**: 855–9.

17 Purcell DW, Johnson CH, Lansky A, *et al.* Estimating the population size of men who have sex with men in the United States to obtain HIV and syphilis rates. *Open AIDS J* 2012; **6**: 98.

18 Beyrer C, Baral SD, van Griensven F, *et al.* Global epidemiology of HIV infection in men who have sex with men. *The Lancet* 2012; **380**: 367–77.

19 United Nations Population Division. World Population Prospects - Population Division - United Nations. 2017. https://esa.un.org/unpd/wpp/ (accessed Nov 30, 2017).

20 Degenhardt L, Peacock A, Colledge S, *et al.* Global prevalence of injecting drug use and sociodemographic characteristics and prevalence of HIV, HBV, and HCV in people who inject drugs: a multistage systematic review. *Lancet Glob Health* 2017.

21 Sherman AC, Sherman KE. Extrahepatic manifestations of hepatitis C infection: navigating CHASM. *Curr HIV/AIDS Rep* 2015; **12**: 353–61.

22 Cacoub P, Gragnani L, Comarmond C, Zignego AL. Extrahepatic manifestations of chronic hepatitis C virus infection. *Dig Liver Dis* 2014; **46**: s165–73.

23 El-Kamary SS, Jhaveri R, Shardell MD. All-cause, liver-related, and non-liver-related mortality among HCV-infected individuals in the general US population. *Clin Infect Dis* 2011; **53**: 150–7.

24 Innes HA, McDonald SA, Dillon JF, *et al.* Toward a more complete understanding of the association between a hepatitis C sustained viral response and cause-specific outcomes. *Hepatology* 2015; **62**: 355–64.

25 World Health Organization. Monitoring and evaluation for viral hepatitis B and C: recommended indicators and framework. 2016 http://apps.who.int/iris/bitstream/10665/204790/1/9789241510288_eng.pdf (accessed July 21, 2017).

26 Cacoub P, Comarmond C, Domont F, Savey L, Desbois AC, Saadoun D. Extrahepatic manifestations of chronic hepatitis C virus infection. *Ther Adv Infect Dis* 2016; **3**: 3–14.

27 Mathers BM, Degenhardt L, Bucello C, Lemon J, Wiessing L, Hickman M. Mortality among people who inject drugs: a systematic review and meta-analysis. *Bull World Health Organ* 2013; **91**: 102–23.

28 United Nations Office on Drugs and Crime. Booklet 2: Global overview of drug demand and supply. In: World Drug Report 2017. Vienna, Austria: United Nations Publication, 2017. https://www.unodc.org/wdr2017/field/Booklet_2_HEALTH.pdf (accessed June 20, 2018).

29 Degenhardt L, Bucello C, Mathers B, *et al.* Mortality among regular or dependent users of heroin and other opioids: a systematic review and meta-analysis of cohort studies. *Addiction* 2011; **106**: 32–51.

30 United Nations Office on Drugs and Crime. World Drug Report 2015. Vienna, Austria: United Nations Publication, 2015 https://www.unodc.org/documents/wdr2015/World_Drug_Report_2015.pdf (accessed June 20, 2018).

31 Chhatwal J, He T, Lopez-Olivo MA. Systematic review of modelling approaches for the cost effectiveness of hepatitis C treatment with direct-acting antivirals. *PharmacoEconomics* 2016; **34**: 551–67.

32 Salomon JA, Weinstein MC, Hammitt JK, Goldie SJ. Empirically calibrated model of hepatitis C virus infection in the United States. *Am J Epidemiol* 2002; **156**: 761–73.

33 Razavi H, ElKhoury AC, Elbasha E, *et al.* Chronic hepatitis C virus (HCV) disease burden and cost in the United States. *Hepatology* 2013; **57**: 2164–2170.

34 Townsend R, McEwan P, Kim R, Yuan Y. Structural frameworks and key model parameters in cost-effectiveness analyses for current and future treatments of chronic hepatitis C. *Value Health* 2011; **14**: 1068–77.

35 Hernandez MD, Sherman KE. HIV/hepatitis C coinfection natural history and disease progression, a review of the most recent literature. *Curr Opin HIV AIDS* 2011; **6**: 478–82.

36 Bräu N, Salvatore M, Ríos-Bedoya CF, *et al.* Slower fibrosis progression in HIV/HCV-coinfected patients with successful HIV suppression using antiretroviral therapy. *J Hepatol* 2006; **44**: 47–55.

37 UNAIDS. Fact sheet - Latest statistics on the status of the AIDS epidemic. http://www.unaids.org/en/resources/fact-sheet (accessed May 11, 2018).

38 Thomson EC, Fleming VM, Main J, *et al.* Predicting spontaneous clearance of acute hepatitis C virus in a large cohort of HIV-1-infected men. *Gut* 2011; **60**: 837–45.

39 Thein H-H, Yi Q, Dore GJ, Krahn MD. Estimation of stage-specific fibrosis progression rates in chronic hepatitis C virus infection: A meta-analysis and meta-regression. *Hepatology* 2008; **48**: 418–31.

40 Yi Q, Wang PP, Krahn M. Improving the accuracy of long-term prognostic estimates in hepatitis C virus infection. *J Viral Hepat* 2004; **11**: 166–174.

41 Razavi H, Waked I, Sarrazin C, *et al.* The present and future disease burden of hepatitis C virus (HCV) infection with today's treatment paradigm. *J Viral Hepat* 2014; **21**: 34–59.

42 Sweeting MJ, De Angelis D, Neal KR, *et al.* Estimated progression rates in three United Kingdom hepatitis C cohorts differed according to method of recruitment. *J Clin Epidemiol* 2006; **59**: 144–52.

43 Fattovich G, Giustina G, Degos F, *et al.* Morbidity and mortality in compensated cirrhosis type C: a retrospective follow-up study of 384 patients. *Gastroenterology* 1997; **112**: 463–72.

44 D'Amico G, Garcia-Tsao G, Pagliaro L. Natural history and prognostic indicators of survival in cirrhosis: A systematic review of 118 studies. *J Hepatol* 2006; **44**: 217–31.

45 Diekmann O, Heesterbeek JAP. Mathematical epidemiology of infectious diseases: model building, analysis and interpretation. John Wiley & Sons, 2000.

46 Ko Y-C, Ho M-S, Chiang T-A, Chang S-J, Chang P-Y. Tattooing as a risk of hepatitis C virus infection. *J Med Virol* 1992; **38**: 288–291.

47 Hayes MO, Harkness GA. Body piercing as a risk factor for viral hepatitis: an integrative research review. *Am J Infect Control* 2001; **29**: 271–4.

48 Mele A, Corona R, Tosti ME, *et al.* Beauty treatments and risk of parenterally transmitted hepatitis: results from the hepatitis surveillance system in Italy. *Scand J Infect Dis* 1995; **27**: 441–4.

49 Scheinmann R, Hagan H, Lelutiu-Weinberger C, *et al.* Non-injection drug use and hepatitis C virus: a systematic review. *Drug Alcohol Depend* 2007; **89**: 1–12.

50 Murphy EL, Bryzman SM, Glynn SA, *et al.* Risk factors for hepatitis C virus infection in United States blood donors. *Hepatology* 2000; **31**: 756–62.

51 Urbanus AT, van de Laar TJ, Stolte IG, *et al.* Hepatitis C virus infections among HIV-infected men who have sex with men: an expanding epidemic. *AIDS* 2009; **23**: F1–7.

52 Giraudon I, Ruf M, Maguire H, *et al.* Increase in diagnosed newly acquired hepatitis C in HIV-positive men who have sex with men across London and Brighton, 2002-2006: is this an outbreak? *Sex Transm Infect* 2008; **84**: 111–5.

53 Bottieau E, Apers L, Van Esbroeck M, Vandenbruaene M, Florence E. Hepatitis C virus infection in HIV-infected men who have sex with men: sustained rising incidence in Antwerp, Belgium, 2001-2009. 2010.

54 Martin NK, Vickerman P, Dore GJ, Hickman M. The hepatitis C virus epidemics in key populations (including people who inject drugs, prisoners and MSM): the use of direct-acting antivirals as treatment for prevention. *Curr Opin HIV AIDS* 2015; **10**: 374–80.

55 Shah SM, Shapshak, Paul, Rivers, James E., *et al.* Detection of HIV-1 DNA in needle/syringes, paraphernalia, and washes from shooting galleries in Miami: a preliminary laboratory report. *J Acquir Immune Defic Syndr* 1996; **11**: 301–6.

56 Anderson RM, May RM, Anderson B. Infectious diseases of humans: dynamics and control. Wiley Online Library, 1992.

57 Wasley A, Alter MJ. Epidemiology of hepatitis C: geographic differences and temporal trends. *Semin Liver Dis* 2000; **20**: 1–16.

58 Prati D. Transmission of hepatitis C virus by blood transfusions and other medical procedures: A global review. *J Hepatol* 2006; **45**: 607–16.

59 World Bank. Health expenditure per capita. 2017. http://data.worldbank.org/indicator/SH.XPD.PCAP (accessed Aug 3, 2017).

60 Lavanchy D. Evolving epidemiology of hepatitis C virus. *Clin Microbiol Infect* 2011; **17**: 107–15.

61 Hagan H, Pouget ER, Des Jarlais DC. A Systematic Review and Meta-Analysis of Interventions to Prevent Hepatitis C Virus Infection in People Who Inject Drugs. *J Infect Dis* 2011; **204**: 74–83.

62 Platt L, Minozzi S, Reed J, *et al.* Needle and syringe programmes and opioid substitution therapy for preventing HCV transmission among people who inject drugs: findings from a Cochrane Review and meta-analysis: OST and NSP to prevent HCV transmission. *Addiction* 2017; published online Oct 23. DOI:10.1111/add.14012.

63 Bruggmann P, Grebely J. Prevention, treatment and care of hepatitis C virus infection among people who inject drugs. *Int J Drug Policy* 2015; **26**: S22–6.

64 Grebely J, Dore GJ. Prevention of hepatitis C virus in injecting drug users: a narrow window of opportunity. *J Infect Dis* 2011; **203**: 571–4.

65 Gore SM, Bird AG. Study size and documentation to detect injection-related hepatitis C in prison. *QJM* 1998; **91**: 353–7.

66 World Health Organization, UNODC, UNAIDS. WHO, UNODC, UNAIDS technical guide for countries to set targets for universal access to HIV prevention, treatment and care for injecting drug users - 2012 revision. 2012.

67 Mathers BM, Degenhardt L, Ali H, *et al.* HIV prevention, treatment, and care services for people who inject drugs: a systematic review of global, regional, and national coverage. *The Lancet* 2010; **375**: 1014–1028.

68 Larney S, Peacock A, Leung J, *et al.* Global, regional, and country-level coverage of interventions to prevent and manage HIV and hepatitis C among people who inject drugs: a systematic review. *Lancet Glob Health* 2017; **5**: e1208–e1220.

69 Micallef JM, Macdonald V, Jauncey M, *et al.* High incidence of hepatitis C virus reinfection within a cohort of injecting drug users. *J Viral Hepat* 2007; **14**: 413–8.

70 Aitken CK, Lewis J, Tracy SL, *et al.* High incidence of hepatitis C virus reinfection in a cohort of injecting drug users. *Hepatology* 2008; **48**: 1746–52.

71 Mehta SH, Cox A, Hoover DR, *et al.* Protection against persistence of hepatitis C. *The Lancet* 2002; **359**: 1478–83.

72 Midgard H, Bjøro B, Mæland A, *et al.* Hepatitis C reinfection after sustained virological response. *J Hepatol* 2016; **64**: 1020–6.

73 Dore GJ, Altice F, Litwin AH, *et al.* Elbasvir–grazoprevir to treat hepatitis C virus infection in persons receiving opioid agonist therapy: a randomized trial. *Ann Intern Med* 2016; **165**: 625.

74 World Hepatitis Alliance. Hepatitis C virus reinfection is uncommon after being cured with DAAs. 2017; published online May 22. http://www.worldhepatitisalliance.org/latest-news/infohep/3140568/hepatitis-c-virus-reinfection-uncommon-after-being-cured-daas (accessed Aug 2, 2018).

75 Scott N, Hellard M, McBryde ES. Modeling hepatitis C virus transmission among people who inject drugs: assumptions, limitations and future challenges. *Virulence* 2016; **7**: 201–8.

76 Fraser H, Martin NK, Brummer-Korvenkontio H, *et al.* Model projections on the impact of HCV treatment in the prevention of HCV transmission among people who inject drugs in Europe. *J Hepatol* 2018; **68**: 402–11.

77 Marin J-M, Robert CP. Bayesian Essentials with R. New York, NY: Springer New York, 2014 http://link.springer.com/10.1007/978-1-4614-8687-9 (accessed Nov 15, 2016).

78 Gelman A, Carlin JB, Stern HS, Rubin DB. Bayesian Data Analysis, 3rd edn. Boca Raton, FL: Chapman & Hall/CRC, 2014.

79 Raftery AE, Bao L. Estimating and projecting trends in HIV/AIDS generalized epidemics using incremental mixture importance sampling. *Biometrics* 2010; **66**: 1162–73.

80 Leigh Johnson, Rob Dorrington, Thomas Rehle, *et al.* THEMBISA version 1.0: A model for evaluating the impact of HIV/AIDS in South Africa. 2014. http://www.publichealth.uct.ac.za/sites/default/files/image_tool/images/108/THEMBISA%20version%201.0.pdf (accessed April 15, 2016).

81 Eaton JW, Hallett TB. Why the proportion of transmission during early-stage HIV infection does not predict the long-term impact of treatment on HIV incidence. *Proc Natl Acad Sci* 2014; **111**: 16202–7.

82 De Maesschalck R, Jouan-Rimbaud D, Massart DL. The Mahalanobis distance. *Chemom Intell Lab Syst* 2000; **50**: 1–18.

83 Yee BE, Nguyen NH, Zhang B, *et al.* Sustained virological response and its treatment predictors in hepatitis C virus genotype 4 compared to genotypes 1, 2, and 3: a meta-analysis. *BMJ Open Gastroenterol* 2015; **2**: e000049.

84 Nguyen MH, Keeffe EB. Prevalence and treatment of hepatitis C virus genotypes 4, 5, and 6. *Clin Gastroenterol Hepatol* 2005; **3**: s97–101.

85 Bunchorntavakul C. Hepatitis C genotype 6: A concise review and response-guided therapy proposal. *World J Hepatol* 2013; **5**: 496.

86 Strader DB, Wright T, Thomas DL, Seeff LB. Diagnosis, management, and treatment of hepatitis C. *Hepatology* 2004; **39**: 1147–71.

87 World Health Organization. Guidelines for the screening, care and treatment of persons with hepatitis C infection. World Health Organization, 2014 http://apps.who.int/iris/bitstream/10665/111747/5/9789241548755_mon.pdf (accessed July 25, 2017).

88 Gambato M, Lens S, Navasa M, Forns X. Treatment options in patients with decompensated cirrhosis, pre-and post-transplantation. *J Hepatol* 2014; **61**: S120–31.

89 Pol S. Lack of evidence of an effect of direct acting antivirals on the recurrence of hepatocellular carcinoma. *J Hepatol* 2016. http://www.natap.org/2016/HCV/PIIS0168827816302598.pdf (accessed Oct 13, 2017).

90 Forns X, Lee SS, Valdes J, *et al.* Glecaprevir plus pibrentasvir for chronic hepatitis C virus genotype 1, 2, 4, 5, or 6 infection in adults with compensated cirrhosis (EXPEDITION-1): a single-arm, open-label, multicentre phase 3 trial. *Lancet Infect Dis* 2017; **17**: 1062–8.

91 Wyles D, Wedemeyer H, Ben-Ari Z, *et al.* Grazoprevir, Ruzasvir, and Uprifosbuvir for HCV After NS5A Treatment Failure. *Hepatology*; : n/a-n/a.

92 Wyles D, Poordad F, Wang S, *et al.* Glecaprevir/Pibrentasvir for HCV Genotype 3 Patients with Cirrhosis and/or Prior Treatment Experience: A Partially Randomized Phase III Clinical Trial. *Hepatology*; : n/a-n/a.

93 Lens S, Fernández I, Rodríguez-Tajes S, *et al.* Interferon-free therapy in elderly patients with advanced liver disease. *Am J Gastroenterol* 2017; **112**: 1400.

94 Dore GJ, Altice F, Litwin AH, *et al.* Elbasvir–grazoprevir to treat hepatitis C virus infection in persons receiving opioid agonist therapy: a randomized trial. *Ann Intern Med* 2016; published online Aug 9. DOI:10.7326/M16-0816.

95 Grebley J, Puoti M, Wedemeyer H, *et al.* FRI-236-Safety and efficacy of Ombitasvir, Paritaprevir/Ritonavir and Dasabuvir with or without ribavirin in chronic hepatitis C patients receiving opioid substitution therapy: a pooled analysis across 12 clinical trials. *J Hepatol* 2017; **66**: S514.

96 Wyles D, Bräu N, Kottilil S, *et al.* Sofosbuvir and velpatasvir for the treatment of hepatitis C virus in patients coinfected with human immunodeficiency virus type 1: an open-label, phase 3 study. *Clin Infect Dis* 2017; **65**: 6–12.

97 Naggie S, Cooper C, Saag M, *et al.* Ledipasvir and sofosbuvir for HCV in patients coinfected with HIV-1. *N Engl J Med* 2015; **373**: 705–13.

98 Wyles DL, Ruane PJ, Sulkowski MS, *et al.* Daclatasvir plus sofosbuvir for HCV in patients coinfected with HIV-1. *N Engl J Med* 2015; **373**: 714–25.

99 World Health Organization. Global report on access to hepatitis C treatment. Focus on overcoming barriers. 2016 http://apps.who.int/iris/handle/10665/250625 (accessed Feb 10, 2017).

100 Liver EA for S of, others. EASL recommendations on treatment of hepatitis C 2015. *J Hepatol* 2015; **63**: 199.

101 World Health Organization. WHO guidelines on hepatitis B and C testing. 2017 http://apps.who.int/iris/bitstream/10665/254621/1/9789241549981-eng.pdf (accessed July 10, 2017).

102 George SL, Bacon BR, Brunt EM, Mihindukulasuriya KL, Hoffmann J, Di Bisceglie AM. Clinical, virologic, histologic, and biochemical outcomes after successful HCV therapy: A 5-year follow-up of 150 patients. *Hepatology* 2009; **49**: 729–38.

103 Lee YA, Friedman SL. Reversal, maintenance or progression: What happens to the liver after a virologic cure of hepatitis C? *Antiviral Res* 2014; **107**: 23–30.

104 Nahon P, Bourcier V, Layese R, *et al.* Eradication of hepatitis C virus infection in patients with cirrhosis reduces risk of liver and non-liver complications. *Gastroenterology* 2017; **152**: 142–156.

105 Bruno S, Di Marco V, Iavarone M, *et al.* Survival of patients with HCV cirrhosis and sustained virologic response is similar to the general population. *J Hepatol* 2016; **64**: 1217–23.

106 Cheung MCM, Walker AJ, Hudson BE, *et al.* Outcomes after successful direct acting antiviral therapy for patients with chronic hepatitis C and decompensated cirrhosis. *J Hepatol* 2016; published online July. DOI:10.1016/j.jhep.2016.06.019.

107 Bruno S, Di Marco V, Iavarone M, *et al.* Improved survival of patients with hepatocellular carcinoma and compensated hepatitis C virus-related cirrhosis who attained sustained virological response. *Liver Int* 2017; **37**: 1526–34.

108 Sibley A, Han KH, Abourached A, *et al.* The present and future disease burden of hepatitis C virus infections with today's treatment paradigm - volume 3. *J Viral Hepat* 2015; **22**: 21–41.

109 Hatzakis A, Chulanov V, Gadano AC, *et al.* The present and future disease burden of hepatitis C virus (HCV) infections with today's treatment paradigm - volume 2. *J Viral Hepat* 2015; **22**: 26–45.

110 Chan HLY, Chen CJ, Omede O, *et al.* The present and future disease burden of hepatitis C virus infections with today's treatment paradigm: Volume 4. *J Viral Hepat* 2017; **24**: 25–43.

111 World Health Organization. Global hepatitis report, 2017. 2017 http://apps.who.int/iris/bitstream/10665/255016/1/9789241565455-eng.pdf?ua=1 (accessed April 28, 2017).

112 Kuo G, Choo QL, Alter HJ, *et al.* An assay for circulating antibodies to a major etiologic virus of human non-A, non-B hepatitis. *Science* 1989; **244**: 362–4.

113 Choo Q, Kuo G, Weiner A, Overby L, Bradley D, Houghton M. Isolation of a cDNA clone derived from a blood-borne non-A, non-B viral hepatitis genome. *Science* 1989; **244**: 359–62.

114 Manns MP, McHutchison JG, Gordon SC, *et al.* Peginterferon alfa-2b plus ribavirin compared with interferon alfa-2b plus ribavirin for initial treatment of chronic hepatitis C: a randomised trial. *Lancet* 2001; **358**: 958–65.

115 Razavi H, Robbins S, Zeuzem S, *et al.* Hepatitis C virus prevalence and level of intervention required to achieve the WHO targets for elimination in the European Union by 2030: a modelling study. *Lancet Gastroenterol Hepatol* 2017; **2**: 325–36.

116 World Health Organization. Progress report on access to hepatitis C treatment: focus on overcoming barriers in low-and middle-income countries. 2018 http://apps.who.int/iris/bitstream/handle/10665/260445/WHO-CDS-HIV-18.4-eng.pdf;jsessionid=7A16961E82328637E7FEF0372A082E09?sequence=1 (accessed May 2, 2018).

117 Martin NK, Hickman M, Hutchinson SJ, Goldberg DJ, Vickerman P. Combination interventions to prevent HCV transmission among people who inject drugs: modeling the impact of antiviral treatment, needle and syringe programs, and opiate substitution therapy. *Clin Infect Dis* 2013; **57**: S39–45.

118 Gountas I, Sypsa V, Anagnostou O, *et al.* Treatment and primary prevention in people who inject drugs for chronic hepatitis C infection: is elimination possible in a high-prevalence setting? *Addiction* 2017; **112**: 1290–9.

119 Scott N, McBryde ES, Thompson A, Doyle JS, Hellard ME. Treatment scale-up to achieve global HCV incidence and mortality elimination targets: a cost-effectiveness model. *Gut* 2016; : gutjnl-2016-311504.

120 World Health Organization. Guidelines for the care and treatment of persons diagnosed with chronic hepatitis C virus infection. 2018 http://apps.who.int/iris/bitstream/handle/10665/273174/9789241550345-eng.pdf.

121 World Health Organization. Global health sector strategy on viral hepatitis 2016–2021. 2016 http://apps.who.int/iris/handle/10665/246177 (accessed April 1, 2017).

122 Nelson PK, Mathers BM, Cowie B, *et al.* Global epidemiology of hepatitis B and hepatitis C in people who inject drugs: results of systematic reviews. *Lancet* 2011; **378**: 571–83.

123 European Monitoring Centre for Drugs and Addiction. European drug report: trends and developments. 2017 http://www.emcdda.europa.eu/system/files/publications/4541/TDAT17001ENN.pdf (accessed Dec 20, 2017).

124 ISO/IEC. ISO International Standard ISO/IEC 14882:2014(E) – Programming Language C++. Geneva, Switzerland: International Organization for Standardization (ISO), 2014 http://www.open-std.org/jtc1/sc22/wg21/docs/papers/2014/n4296.pdf (accessed Sept 5, 2017).

125 Wickham H. ggplot2: Elegant Graphics for Data Analysis. Springer-Verlag New York, 2009 http://ggplot2.org.

126 South A. rworldmap: A New R package for Mapping Global Data. *R J* 2011; **3**: 35–43.

127 R Core Team. R: A Language and Environment for Statistical Computing. Vienna, Austria: R Foundation for Statistical Computing, 2016 https://www.R-project.org/.

128 Wickham H, Francois R, Henry L, Müller K. dplyr: A Grammar of Data Manipulation. 2017 https://CRAN.R-project.org/package=dplyr.

129 Wickham H, Bryan J. readxl: Read Excel Files. 2017 https://CRAN.R-project.org/package=readxl.

130 Dragulescu AA. xlsx: Read, write, format Excel 2007 and Excel 97/2000/XP/2003 files. 2014 https://CRAN.R-project.org/package=xlsx.

131 R-bloggers. The Paul Tol 21-color salute. R-Bloggers. 2013; published online Feb 27. https://www.r-bloggers.com/the-paul-tol-21-color-salute/ (accessed Sept 5, 2017).

132 Auguie B. gridExtra: Miscellaneous Functions for 'Grid' Graphics. 2016 https://CRAN.R-project.org/package=gridExtra.

133 Wickham H. scales: Scale Functions for Visualization. 2016 https://CRAN.R-project.org/package=scales.

134 Gagolewski M. R package stringi: Character string processing facilities. 2017 http://www.gagolewski.com/software/stringi/.

135 Wickham H. The Split-Apply-Combine Strategy for Data Analysis. *J Stat Softw* 2011; **40**: 1–29.

136 Wickham H, Hester J, Francois R. readr: Read Rectangular Text Data. 2017 https://CRAN.R-project.org/package=readr.

137 Mokdad AA, Lopez AD, Shahraz S, *et al.* Liver cirrhosis mortality in 187 countries between 1980 and 2010: a systematic analysis. *BMC Med* 2014; **12**: 1.

138 Institute for Health Metrics and Evaluation (IHME). GBD Results Tool. 2017. http://ghdx.healthdata.org/gbd-results-tool (accessed Oct 13, 2017).

139 Stanaway JD, Flaxman AD, Naghavi M, *et al.* The global burden of viral hepatitis from 1990 to 2013: findings from the Global Burden of Disease study 2013. *Lancet* 2016; **388**: 1081–1088.

140 Institute for Health Metrics and Evaluation (IHME). GBD 2015 geographies. 2015. http://www.healthdata.org/sites/default/files/files/Projects/GBD/GBDRegions_countries.pdf (accessed Sept 13, 2017).

141 United Nations Population Division. World Population Prospects - Population Division - United Nations. 2015. https://esa.un.org/unpd/wpp/ (accessed June 17, 2016).

142 Ferrero S, Lungaro P, Bruzzone BM, Gotta C, Bentivoglio G, Ragni N. Prospective study of mother-to-infant transmission of hepatitis C virus: a 10-year survey (1990–2000). *Acta Obstet Gynecol Scand* 2003; **82**: 229–234.

143 Grebely J, Page K, Sacks-Davis R, *et al.* The effects of female sex, viral genotype, and IL28B genotype on spontaneous clearance of acute hepatitis C virus infection. *Hepatology* 2014; **59**: 109–20.

144 Micallef JM, Kaldor JM, Dore GJ. Spontaneous viral clearance following acute hepatitis C infection: a systematic review of longitudinal studies. *J Viral Hepat* 2006; **13**: 34–41.

145 Vogt M, Lang T, Frösner G, *et al.* Prevalence and clinical outcome of hepatitis C infection in children who underwent cardiac surgery before the implementation of blood-donor screening. *N Engl J Med* 1999; **341**: 866–870.

146 Dienstag JL, Ghany MG, Morgan TR, *et al.* A prospective study of the rate of progression in compensated, histologically advanced chronic hepatitis C. *Hepatology* 2011; **54**: 396–405.

147 Kato Y, Nakata K, Nagataki S, *et al.* Risk of hepatocellular carcinoma in patients with cirrhosis in Japan. Analysis of infectious hepatitis viruses. *Cancer* 1994; **74**: 2234–8.

148 Planas R, Ballesté B, Antonio Álvarez M, *et al.* Natural history of decompensated hepatitis C virus-related cirrhosis. A study of 200 patients. *J Hepatol* 2004; **40**: 823–30.

149 Altekruse SF, McGlynn KA, Reichman ME. Hepatocellular carcinoma incidence, mortality, and survival trends in the United States from 1975 to 2005. *J Clin Oncol* 2009; **27**: 1485–91.

150 Shiratori Y, Shiina S, Imamura M, *et al.* Characteristic difference of hepatocellular carcinoma between hepatitis B-and C-viral infection in Japan. *Hepatology* 1995; **22**: 1027–1033.

151 Ohmer S, Honegger J. New prospects for the treatment and prevention of hepatitis C in children. *Curr Opin Pediatr* 2016; **28**: 93–100.

152 United Nations Office on Drugs and Crime (UNODC). UNODC database. https://data.unodc.org/#state:0 (accessed Sept 8, 2017).
